# Efficacy and tolerability of pharmacological interventions for schizophrenia non-responsive to prior treatment: a systematic review and network meta-analysis

**DOI:** 10.1016/j.eclinm.2025.103291

**Published:** 2025-06-07

**Authors:** Myrto Samara, Andreas S. Lappas, Elisavet Pinioti, Eleni Glarou, Iwo Fober, Christos Christogiannis, Spyridon Siafis, Nikos Christodoulou, Bartosz Helfer, Dimitris Mavridis, Stefan Leucht

**Affiliations:** a Department of Psychiatry and Psychotherapy, TUM School of Medicine and Health, Munich, Germany; b Department of Psychiatry, Faculty of Medicine, University of Thessaly, Larisa, Greece; c Department of Geriatric Psychiatry, Aneurin Bevan University Health Board, United Kingdom; d Centre for Trials Research, Cardiff University, Cardiff, United Kingdom; e Division of Population Medicine, School of Medicine, Cardiff University, Cardiff, United Kingdom; f Meta Research Centre, University of Wroclaw, Wroclaw, Poland; g Institute of Psychology, University of Wroclaw, Wroclaw, Poland; h Department of Primary Education, School of Education, University of Ioannina, Ioannina, Greece; i Developmental EPI (Evidence Synthesis, Prediction, Implementation) Lab, Centre for Innovation in Mental Health, School of Psychology, Faculty of Environmental and Life Sciences, University of Southampton, Southampton, United Kingdom

**Keywords:** Resistance, Clozapine, Combination, Augmentation, Antipsychotics, Polypharmacy

## Abstract

**Background:**

Treatment-resistant schizophrenia (TRS) poses significant challenges for both clinicians and patients. This systematic review and network meta-analysis (NMA) aimed to compare the efficacy and tolerability of all available pharmacotherapy options.

**Methods:**

We systematically searched MEDLINE, Cochrane Central, Embase, PsycINFO, ClinicalTrials.gov, WHO trials registry, and FDA website through March 2025 for randomised controlled trials (RCTs) comparing pharmacological treatments for TRS. NMA estimated pooled effects, with the primary outcome being overall symptom change. Secondary outcomes included treatment response, individual symptom domains, discontinuation, adverse events, quality of life, and functioning. Effect sizes were reported as standardized mean differences (SMDs) for continuous outcomes and odds ratios (ORs) for dichotomous outcomes, with 95% confidence intervals (CIs). Meta-regression and sensitivity analyses explored variability in findings.

**Findings:**

150 RCTs with 11,375 patients examined 78 drug options or placebo. Clozapine showed superior efficacy for overall symptoms compared to haloperidol, chlorpromazine, quetiapine, and sulpiride (SMDs 0.35 to 1.00). It slightly outperformed olanzapine for positive symptoms (SMD 0.19; 95% CI 0.00 to 0.37) and risperidone for response rates (OR 0.64; 95% CI 0.41 to 1.01). Clozapine combinations with amisulpride, duloxetine, memantine, mirtazapine, topiramate, and ziprasidone improved overall symptoms more than clozapine monotherapy (SMDs −1.53 to −0.51). In a similar vein, clozapine combinations with amisulpride, lamotrigine, and topiramate reduced positive symptoms more than monotherapy (SMDs −1.13 to −0.54), while with duloxetine, memantine, and ziprasidone negative symptoms (SMDs −1.98 to −0.99). Some antipsychotic combinations may outperform monotherapy, but data on non-clozapine combinations were limited. Higher baseline severity was associated with higher clozapine efficacy. Confidence in most estimates was low or very low.

**Interpretation:**

Clozapine remains the gold standard, outperforming several antipsychotics, while specific combinations may offer added benefits but require careful risk-benefit evaluation. Networks sparsity increases the likelihood of chance findings for estimates based on single studies. These results emphasise the need for personalised treatment, further research comparing non-clozapine antipsychotic combinations to high-dose clozapine monotherapy, and studies on long-term outcomes.

**Funding:**

None.

**Research in context**

**Evidence before this study**

We searched PubMed for network meta-analyses on the treatment of resistant schizophrenia from database inception to the 4th of March 2025. We found some relevant systematic reviews either on antipsychotic monotherapy options or examining specific combinations. Overall, we found no comprehensive network meta-analysis comparing all pharmacological options for the treatment of resistant schizophrenia across several efficacy and safety outcomes.

**Added value of this study**

To our knowledge, this analysis is the largest network meta-analysis for treatment resistant schizophrenia. It was based on 150 studies including 11,375 participants randomly assigned to 78 pharmacological options or placebo. The primary outcome was reduction of overall symptoms of schizophrenia, but we also examined reduction in positive, negative and depressive symptoms, as well as response rates, dropouts, quality of life, functioning and specific side effects. Clozapine outperformed several antipsychotics including chlorpromazine, haloperidol, quetiapine, and sulpiride. Certain combinations with clozapine, such as amisulpride, duloxetine, memantine, mirtazapine, topiramate, and ziprasidone, improved overall schizophrenia symptoms more than clozapine monotherapy. In combination with clozapine, amisulpride, lamotrigine, and topiramate were particularly effective in reducing positive symptoms, while duloxetine, mirtazapine, memantine, and ziprasidone provided benefits for negative symptoms. The combination of clozapine with topiramate was associated with more dropouts due to any cause and due to side effects, indicating lower tolerability. Due to the scarcity of data on certain drug options, comparisons involving them were often highly uncertain, and the evidence was of low or very low quality. Conclusions regarding the primary outcome remained largely unchanged after adjustments for potential effect moderators and sensitivity analyses, except for baseline severity, which showed a positive association with clozapine’s efficacy.

**Implications of all the available evidence**

Despite a large evidence base of randomized clinical trials for the treatment of resistant schizophrenia, significant gaps remain. Many drug options are supported by only one or two studies, limiting confidence in the estimates. Nevertheless, specific combinations, particularly combinations of antipsychotics, demonstrate superior efficacy compared to antipsychotic monotherapy, without evidence of compromised safety. These findings underscore the pressing need for further research and should guide decision-making processes and the development of clinical guidelines internationally.

## Introduction

Non-response to treatment is a major and common challenge in managing patients with schizophrenia. Approximately 40% of patients do not achieve even minimal response to antipsychotics in randomised controlled trials (RCTs),^1^ with rates reaching up to 75% in routine clinical practice.^2^ Suboptimal outcomes contribute to impaired socio-occupational functioning and quality of life,^3^^,^^4^ and significant socioeconomic costs.^5^

Clozapine remains the gold standard for treatment-resistant schizophrenia (TRS).^6^^,^^7^ Although highly effective for many patients, it is not universally suitable due to potential inefficacy,^8^ tolerability challenges,^9^ or safety risks. These limitations may prompt clinicians to consider alternatives, despite the scarcity of robust evidence supporting other options. Additionally, concerns about severe adverse effects—such as agranulocytosis and myocarditis—and the requirement for frequent monitoring often discourage both clinicians and patients from its use.^10^, ^11^, ^12^ Even when prescribed, clozapine is frequently combined with another antipsychotic as a last-resort strategy.^13^^,^^14^ Data from six Belgian hospitals indicated that more than 70% of patients treated with clozapine were also prescribed a second antipsychotic.^13^

Previous meta-analyses have primarily focused on either monotherapy options^15^^,^^16^ or specific drug combinations,^17^, ^18^, ^19^, ^20^, ^21^, ^22^ rather than evaluating comparative efficacy and safety of all pharmacological interventions in cases of antipsychotic non-response. However, in real-world clinical practice, clinicians often employ a range of pharmacological strategies, including both monotherapies and combination therapies. Therefore, we conducted a comprehensive network meta-analysis (NMA) including all available drug treatments for TRS, specifically antipsychotic monotherapy or antipsychotic combinations with any other drug. This approach aims to provide a more comprehensive overview of the evidence supporting available pharmacotherapy options.

## Methods

### Search strategy and selection criteria

This NMA was conducted in accordance with the PRISMA reporting guidelines^23^^,^^24^ (Appendix 1). The review protocol was published in OSF Preregistration (https://doi.org/10.17605/OSF.IO/4AJUR) and is presented in Appendix 2. We searched the following sources without any language restrictions from database inception until March 4, 2025: MEDLINE (via Ovid), the Cochrane Central Register of Controlled Trials (CENTRAL), Embase, PsycINFO, the clinical trials register ClinicalTrials.gov, the WHO International Clinical Trials Registry Platform (ICTRP), and the US Food and Drug Administration website. The search strategy is available in Appendix 3. The reference lists of included studies, as well as previously published reviews by our team^15^^,^^16^ and others,^18^, ^19^, ^20^^,^^25^ were screened for additional studies. Three reviewers (EP, EG, and IF) independently screened the search results, retrieved full-text articles, and examined these against our inclusion criteria. In case of conflicts, another reviewer (ASL, MS) was consulted.

We included only RCTs, excluding cluster randomised trials, trials with duration less than three weeks^15^^,^^16^ and trials with a high risk of bias in sequence generation or allocation concealment.^26^

We included participants with TRS or related psychoses, such as schizophreniform or schizoaffective disorders. Patients were included based on the study-defined criteria for treatment resistance or non-response and having a diagnosis of schizophrenia given by any means of operationalised criteria (e.g., DSM, ICD) or a clinical diagnosis. There were no restrictions in terms of age, sex or ethnicity. Criteria for treatment resistance with varying levels of rigor were addressed in subgroup analyses (see below).

We included all antipsychotic medication available worldwide, at any dose and in any form of administration, including long-acting injectables (LAIs), either as monotherapy or in combination with any other drug, and placebo.

### Data collection and outcomes

Three reviewers (EP, EG, and IF) independently extracted and entered data into electronic forms in Excel sheets. Differences were discussed, and if consensus was not reached, a senior reviewer (MS, ASL, SL) was contacted. Study authors were contacted when necessary.

The primary outcome was the change in overall schizophrenia symptoms, as measured by rating scales such as the Positive and Negative Syndrome Scale (PANSS),^27^ the Brief Psychiatric Rating Scale (BPRS),^28^ or any other validated scale. Secondary outcomes included clinically relevant treatment response as defined by the authors of the trials, the average change in scores on a positive symptom scale, a negative symptom scale, and a depressive symptom scale. We also assessed all-cause discontinuation, discontinuation due to inefficacy, discontinuation due to adverse events, the total number of patients experiencing adverse events, and the average change in scores on quality-of-life and functioning scales. Major side effects were examined as individual outcomes. These included the use of antiparkinsonian medication, weight gain, sedation, prolactin levels, and QTc prolongation. Odds ratios (ORs) were reported for dichotomous outcomes and standardized mean differences (SMDs) for continuous outcomes, accompanied by their corresponding confidence intervals (CIs) and prediction intervals (PIs). Criteria for treatment resistance were based on the following groups: 1) one previously failed antipsychotic trial, based on historical information prior to entering the study; 2) at least 2 previously failed antipsychotic trials, based on historical information prior to entering the study; 3) a combination of retrospective (historical information prior to entering the study) and prospective (failed trials as part of the study design) criteria for treatment resistance; 4) no or partial response to Clozapine (Ultra TRS). Missing standard deviations (SDs) were estimated from test statistics.^26^

Three reviewers (EP, EG, and IF) independently assessed the risk of bias, using the Cochrane Handbook for Systematic Reviews of Interventions.^26^ The overall risk of bias was classified as high, moderate, or low.^29^ The confidence in estimates of the primary outcome were assessed using the Confidence in Network Meta-Analysis (CINeMA) framework and on-line application.^30^^,^^31^

### Ethics

As a systematic review and meta-analysis of previously published studies, this research did not require additional ethical approval or informed consent.

### Statistics

We performed a network meta-analysis combining direct and indirect comparisons across eligible studies^32^^,^^33^ (Appendix 8). Data were combined using a random-effects model in continuous and binary outcomes, while for binary we also applied a variety of common-effect models specifically designed to handle meta-analyses with few number of events in some trials^34^ (Appendix 12). Clozapine monotherapy served as the reference. We used P-scores for ranking interventions.^35^ The transitivity assumption was evaluated by comparing the distribution of potential effect modifiers (publication year, sample size, baseline severity, mean age, and percentage of male participants) across studies grouped by comparison (Appendix 9). We assessed network consistency by applying both local and global methods. We employed the node-splitting approach by Dias and colleagues^36^ for local inconsistency and the design-by-treatment model as described by Higgins and colleagues^37^ for global consistency. Additionally, we present the net-heat plot to detect closed loops of inconsistency.^38^ We explored residual heterogeneity through several meta-regressions (resistance level, symptom type, age, duration of illness, sex, baseline severity of illness, treatment concept, dose, trial duration, publication year, sponsorship, and country) and sensitivity analyses (excluding studies with small samples, lack of double-blinding, non-operationalized diagnostic criteria, patients who may have been considered treatment-resistant due to intolerance, high risk of bias, and missing data assumptions; applying a fixed-effect model; and an extreme sensitivity analysis excluding open label studies, intolerant patients and those with low and medium stringency resistance criteria). Post hoc, we decided to examine whether a combination of antipsychotics was superior to antipsychotic monotherapy in a pairwise meta-analysis. We used contour-enhanced funnel plots and the trim-and-fill method for the primary outcome to investigate the presence of small-study effects, whereby small studies give different results from the large studies for any comparison against clozapine. All analyses were done in R version 4.3.3.^39^

### Role of the funding source

There was no funding source for this study.

## Results

**Fig. 1:**
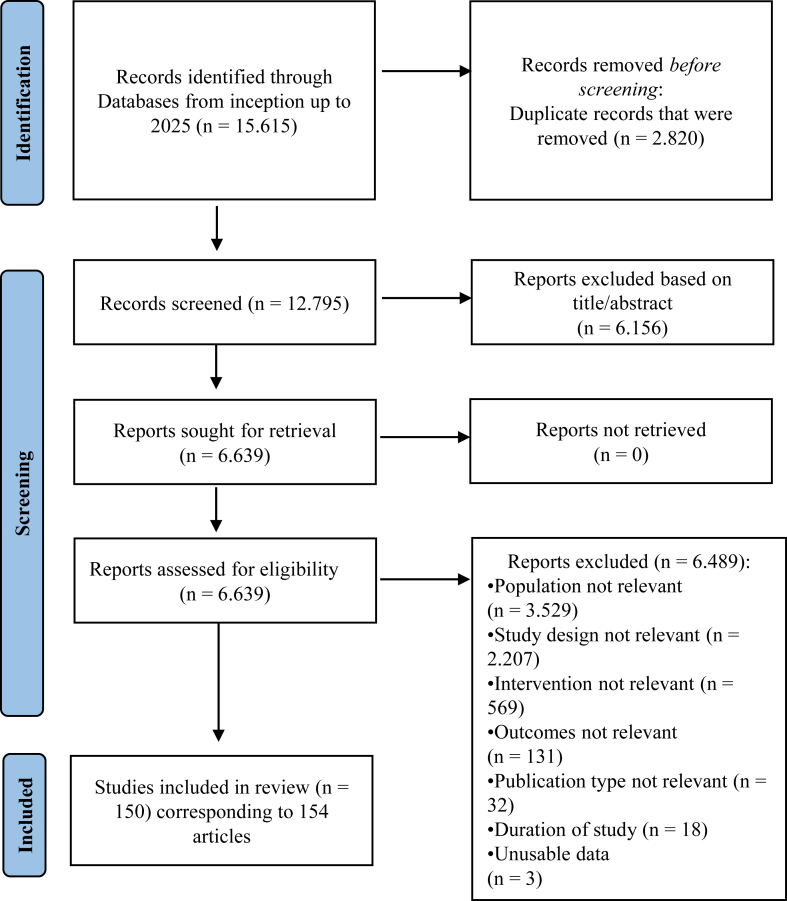
Study flow diagram.

The PRISMA flow diagram and table of included studies are presented in Fig. 1 and Appendix 4–6. 154 studies, reporting on 150 individual RCTs with a total of 11,375 randomised participants and examining 78 different drug options or placebo were included. The studies were published between 1958 and 2024. Since race and ethnicity data were not available, the region of origin for each study is provided instead. Most of the studies were conducted in the USA (52 studies). 127 studies employed a double-blind, 14 a single-blind, and seven an open label design, while only two studies^40^^,^^41^ did not provide any information regarding blinding. Study duration varied from 4 to 52 weeks, with a median of 12 weeks. The majority of the studies involved adults of working age (38.01 years), while only 3 included children and adolescent populations.^42^, ^43^, ^44^ The number of males in 142 RCTs that provided information was 7818 out of 11,076 (71%). A total of five RCTs included only male participants,^45^, ^46^, ^47^, ^48^, ^49^ while three included only female participants.^50^, ^51^, ^52^ The vast majority of included studies (89.33%) used operationalised criteria (e.g., DSM, ICD) for the diagnosis of schizophrenia, while 9 studies (6%) reported a clinical diagnosis^45^, ^46^, ^47^^,^^51^, ^52^, ^53^, ^54^, ^55^, ^56^ and seven studies (4.67%) did not specify how the diagnosis was made.^57^, ^58^, ^59^, ^60^, ^61^, ^62^, ^63^ All studies included TRS patients, utilising various criteria for the definition of resistance. A total of 69 RCTs evaluated antipsychotic monotherapy (including six with placebo arms^47^^,^^51^^,^^52^^,^^54^^,^^55^^,^^64^), while 81 RCTs assessed combinations of antipsychotics with other medications.

**Fig. 2:**
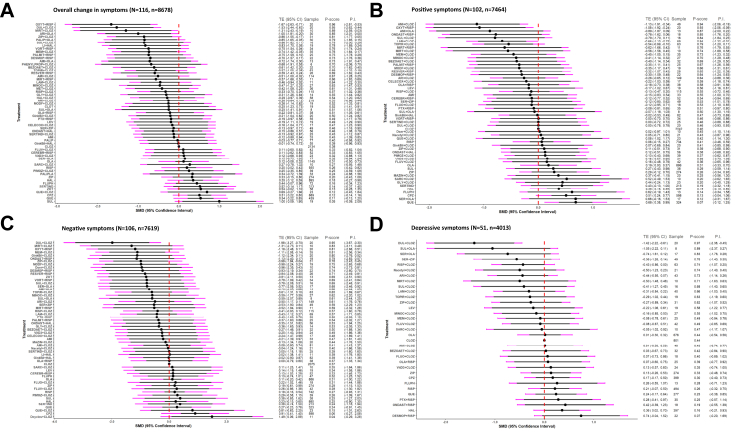
Change in efficacy continuous outcomes. (A) Overall change in symptoms. (B) Positive symptoms. (C) Negative symptoms. (D) Depressive symptoms. All treatments were compared to clozapine monotherapy as the reference. Treatments are ordered by efficacy based on P-scores, with the most efficacious shown at the top and the least at the bottom. The forest plot displays standardized mean differences (SMDs) with 95% confidence intervals (CI; grey bars) and prediction intervals (PI; purple bars). Grey bars crossing the vertical (y) axis indicate no statistically significant difference from clozapine monotherapy. TE: treatment effect (SMD); AMI: amisulpride; ARI: aripiprazole; BEZOAET: benzoate sodium; CELECOX: celecoxib; CEREBR: cerebrolysin; CLOZ: clozapine; CLOT: clotiapine; CPZ: chlorpromazine; DESMOP: desmopressin; DUL: duloxetine; FLUO: fluoxetine; FLUPHLA: fluphenazine decanoate; FLUV: Fluvoxamine; GinkBil: *Ginkgo biloba*; GLY: glycine; HAL: haloperidol; LAM: lamotrigine; LEV: levomepromazine; LI: lithium; MEM: memantine; MET: metformin; MINOC: minocycline; MIRT: mirtazapine; MODF: modafinil; ONDAST: ondansetron; OLA: olanzapine; OXYT: Oxytocin; PALIP: paliperidone; PALMIT: palmitoylethanolamide; PBO: placebo; PHENYLPROP: phenylpropanolamine; PIMOZ: pimozide; PTX: pentoxifylline; QUE: quetiapine; RESVER: resveratrol; RISP: risperidone; SARC: sarcosine; SER: sertraline; SERTIND: sertindole; SUL: sulpiride; TOPIR: topiramate; VitB6: vitamin B6; VitD3: vitamin D3; VORT: vortioxetine; ZIP: ziprasidone.

116 studies with 8678 participants reported useable data for change in overall symptoms (Appendix 10.1). Intranasal oxytocin plus risperidone, duloxetine plus clozapine, mirtazapine plus clozapine, aripiprazole plus olanzapine, paliperidone plus olanzapine, ziprasidone plus clozapine, memantine plus clozapine, topiramate plus clozapine, and amisulpride plus clozapine reduced overall symptoms more than clozapine monotherapy (Fig. 2A, Table 1), with SMDs ranging between −1.67 (95%CI −2.63, −0.71) for intranasal oxytocin plus risperidone to −0.51 (−1.00, −0.02) for amisulpride plus clozapine. Haloperidol, chlorpromazine, quetiapine, and sulpiride were less efficacious than clozapine, with SMDs ranging from 0.35 (0.12, 0.59) for haloperidol to 1.00 for sulpiride (0.09, 1.90). However, most PIs were wide, indicating important uncertainty about treatment effects in future studies, and confidence in most estimates was low or very low.

**Table 1:** Overall change in symptoms.

Treatment	Effect size (95% CI)	Prediction interval	Overall confidence
	Compared to clozapine monotherapy		
Oxytocin + Risperidone	−1.67 (−2.63, −0.71)	−2.81, −0.53	Very Low
Duloxetine + Clozapine	−1.53 (−2.44, −0.63)	−2.62, −0.44	Moderate
Mirtazapine + Clozapine	−1.31 (−2.43, −0.19)	−2.59, −0.03	Low
Aripiprazole + Olanzapine	−1.00 (−1.81, −0.20)	−2.01, 0.00	Low
Ziprasidone + Clozapine	−0.86 (−1.55, −0.17)	−1.77, 0.05	Low
Paliperidone + Olanzapine	−0.85 (−1.65, −0.05)	−1.85, 0.15	Low
Memantine + Clozapine	−0.74 (−1.42, −0.05)	−1.64, 0.17	Moderate
Topiramate + Clozapine	−0.57 (−1.05, −0.08)	−1.33, 0.19	Low
Amisulpride + Clozapine	−0.51 (−1.00, −0.02)	−1.28, 0.25	Low
Haloperidol	0.35 (0.12, 0.59)	−0.27, 0.98	Low
Chlorpromazine	0.51 (0.22, 0.80)	−0.14, 1.16	Low
Quetiapine	0.54 (0.22, 0.85)	−0.13, 1.20	Moderate
Sulpiride	1.00 (0.09, 1.90)	−0.09, 2.09	Moderate

Negative effect sizes indicate that other treatments are superior to clozapine, while positive effect sizes indicate that clozapine is superior to other treatments.

95% CI: Confidence Interval; Overall Confidence estimates are based on CINeMA.

Secondary outcomes were reported less frequently except dropouts due to any reason. 102 studies with 7464 participants presented useable data for reduction of positive symptoms. Amisulpride plus clozapine, intranasal oxytocin plus risperidone, lamotrigine plus clozapine, and topiramate plus clozapine reduced positive symptoms more than clozapine monotherapy with SMDs ranging from −1.13 (−1.91, −0.34) for amisulpride plus clozapine to −0.54 (−0.99, −0.10) for topiramate plus clozapine. Clozapine was superior to chlorpromazine, haloperidol, quetiapine, and marginally to olanzapine, with SMDs ranging from 0.19 (0.00, 0.37) for olanzapine to 0.68 (0.38; 0.99) for quetiapine (Fig. 2B).

106 studies with 7619 participants presented useable data for reduction of negative symptoms. Duloxetine plus clozapine, memantine plus clozapine, and ziprasidone plus clozapine demonstrated a greater reduction in negative symptoms compared to clozapine monotherapy. The SMDs ranged from −1.98 (−3.27, −0.70) for duloxetine plus clozapine to −0.99 (−1.94, −0.04) for ziprasidone plus clozapine. In contrast, the combination of D-cycloserine with clozapine, and chlorpromazine were less efficacious than clozapine, with SMDs 1.49 (0.09, 2.89) and 0.91 (0.41, 1.40) respectively (Fig. 2C).

51 studies with 4013 participants reported useable data for depressive symptoms. The combination of duloxetine with clozapine, and to a lesser extent clozapine with risperidone, showed a greater reduction in depressive symptoms compared to clozapine monotherapy, with SMDs of −1.42 (−2.22, −0.61) and −0.43 (−0.86, 0.00), respectively. In contrast, haloperidol was less efficacious than clozapine, with an SMD of 0.36 (0.02, 0.70) (Fig. 2D).

**Fig. 3:**
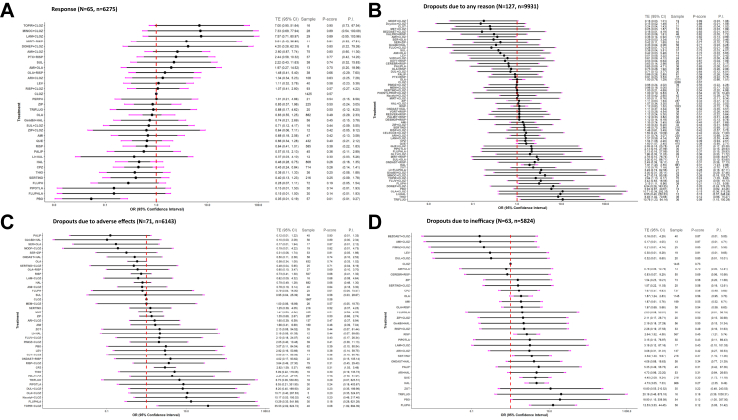
Dichotomous outcomes. (A) Response. (B) Dropouts due to any reason. (C) Dropouts due to adverse effects. (D) Dropouts due to inefficacy. All treatments were compared to clozapine monotherapy as the reference. Treatments are ordered by efficacy based on P-scores, with the most efficacious shown at the top and the least at the bottom. The forest plot displays odds ratios (ORs) with 95% confidence intervals (CI; grey bars) and prediction intervals (PI; purple bars). Grey bars crossing the vertical (y) axis indicate no statistically significant difference from clozapine monotherapy. TE = treatment effect (OR). AMI: amisulpride; ARI: aripiprazole; BEZOAET: benzoate sodium; CELECOX: celecoxib; CEREBR: cerebrolysin; CLOZ: clozapine; CLOT: clotiapine; CPZ: chlorpromazine; DESMOP: desmopressin; DUL: duloxetine; FLUO: fluoxetine; FLUPHLA: fluphenazine decanoate; FLUV: Fluvoxamine; GinkBil: *Ginkgo biloba*; GLY: glycine; HAL: haloperidol; LAM: lamotrigine; LEV: levomepromazine; LI: lithium; MEM: memantine; MET: metformin; MINOC: minocycline; MIRT: mirtazapine; MODF: modafinil; ONDAST: ondansetron; OLA: olanzapine; OXYT: Oxytocin; PALIP: paliperidone; PALMIT: palmitoylethanolamide; PBO: placebo; PHENYLPROP: phenylpropanolamine; PIMOZ: pimozide; PTX: pentoxifylline; QUE: quetiapine; RESVER: resveratrol; RISP: risperidone; SARC: sarcosine; SER: sertraline; SERTIND: sertindole; SUL: sulpiride; TOPIR: topiramate; VitB6: vitamin B6; VitD3: vitamin D3; VORT: vortioxetine; ZIP: ziprasidone.

65 studies with 6275 participants reported study-defined response rates with varied cut-off definitions. Clozapine demonstrated higher response rates than haloperidol, chlorpromazine, and placebo, with ORs ranging from 0.05 (0.01, 0.19) for placebo to 0.46 (0.28, 0.75) for haloperidol. It was also marginally superior to risperidone (0.64; 0.41, 1.01) (Fig. 3A).

127 studies with 9931 participants reported useable data for drop-outs due to any reason. Only amisulpride plus clozapine showed lower drop-out rates due to any reason compared to clozapine with OR 0.36 (0.14, 0.92). On the contrary, chlorpromazine, quetiapine, haloperidol, topiramate plus clozapine, fluphenazine, thioridazine, and trifluoperazine, presented higher drop-out rates due to any reason with OR ranging from 1.54 (1.08, 2.20) for chlorpromazine to 10.76 (1.23, 94.14) for trifluoperazine (Fig. 3B).

71 studies with 6143 participants reported useable data for drop-outs due to any adverse effects. Chlorpromazine, and topiramate plus clozapine showed higher drop-out rates due to adverse effects compared to clozapine (ORs 2.92 and 35.53 respectively), while olanzapine had slightly lower rates (OR 0.58; 0.34, 1.00) (Fig. 3C).

63 studies with 5824 participants reported useable data for drop-outs due to inefficacy. Olanzapine, risperidone, sertindole, quetiapine, haloperidol, fluphenazine, and placebo showed higher drop-out rates due to inefficacy compared to clozapine, with OR ranging from 1.87 (1.24, 2.83) for olanzapine to 12.53 (3.53, 44.45) for fluphenazine (Fig. 3D).

22 studies with 2506 participants provided data for total number of participants experiencing adverse effects. Compared to clozapine, placebo and olanzapine had fewer participants with adverse effects (OR 0.07 and 0.40 respectively) while amisulpride plus clozapine had more (OR 3.45) (Appendix 10.9).

As for specific adverse effects, clozapine use necessitated less anti-Parkinson medication use than risperidone, fluphenazine, haloperidol, and zotepine (ORs from 0.03 to 0.31). It however caused more sedation than amisulpride, ziprasidone, sertindole, quetiapine, olanzapine, haloperidol, risperidone, and chlorpromazine (ORs from 1.72 to 6.67); more weight gain than ziprasidone, haloperidol, chlorpromazine, aripiprazole plus clozapine, quetiapine, and risperidone (SMDs from 0.32 to 0.78); higher prolactin levels than aripiprazole plus clozapine, and ziprasidone plus clozapine (SMDs 0.96 and 3.37 respectively) and lower compared to haloperidol, amisulpride plus clozapine, zotepine, olanzapine plus risperidone, risperidone, risperidone plus clozapine, and sulpiride plus clozapine (SMDs from −0.69 to −3.15). Lastly, clozapine caused more QTc prolongation than risperidone plus clozapine (SMD 1.31)(Appendixes 10.10–10.14).

No difference was shown between other treatments and clozapine for quality of life and functioning (Appendixes 10.15 and 10.16).

46 studies (30.67%) were judged as having an overall low risk of bias, while 75 studies (50%) were judged as having an overall moderate risk of bias and 29 (19.33%) having an overall high risk of bias. The risk of bias summary plot and assessment per individual study are presented in the Appendix 7. Heterogeneity was low to moderate for most outcomes, moderate to high for change in overall symptoms, and high for negative symptoms, functioning and QTc prolongation. Heterogeneity and inconsistency assessments are presented in the Appendix 13. Judgements about confidence in most estimates (CINeMA) for the primary outcome ranged from very low to moderate (Appendix 14).

**Fig. 4:**
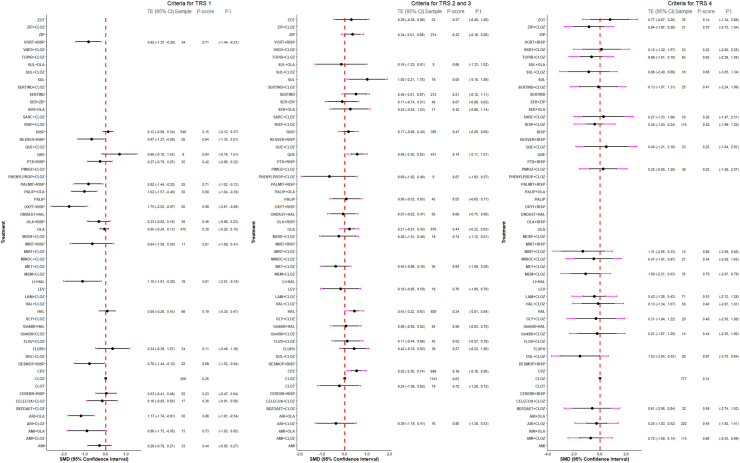
Forest plots comparing treatments versus clozapine monotherapy for the primary outcome (overall symptom change) are presented separately for treatment-resistance subgroups. Criteria for treatment resistance: TRS-1: one previously failed antipsychotic trial; TRS 2 and 3: at least 2 previously failed antipsychotic trials; TRS-4: no or partial response to clozapine (Ultra TRS). An SMD below 0 favors the treatment listed on the left, while an SMD above 0 favors clozapine monotherapy. SMD = standardised mean difference. AMI: amisulpride; ARI: aripiprazole; BEZOAET: benzoate sodium; CELECOX: celecoxib; CEREBR: cerebrolysin; CLOZ: clozapine; CLOT: clotiapine; CPZ: chlorpromazine; DESMOP: desmopressin; DUL: duloxetine; FLUO: fluoxetine; FLUPHLA: fluphenazine decanoate; FLUV: Fluvoxamine; GinkBil: *Ginkgo biloba*; GLY: glycine; HAL: haloperidol; LAM: lamotrigine; LEV: levomepromazine; LI: lithium; MEM: memantine; MET: metformin; MINOC: minocycline; MIRT: mirtazapine; MODF: modafinil; ONDAST: ondansetron; OLA: olanzapine; OXYT: Oxytocin; PALIP: paliperidone; PALMIT: palmitoylethanolamide; PBO: placebo; PHENYLPROP: phenylpropanolamine; PIMOZ: pimozide; PTX: pentoxifylline; QUE: quetiapine; RESVER: resveratrol; RISP: risperidone; SARC: sarcosine; SER: sertraline; SERTIND: sertindole; SUL: sulpiride; TOPIR: topiramate; VitB6: vitamin B6; VitD3: vitamin D3; VORT: vortioxetine; ZIP: ziprasidone.

**Fig. 5:**
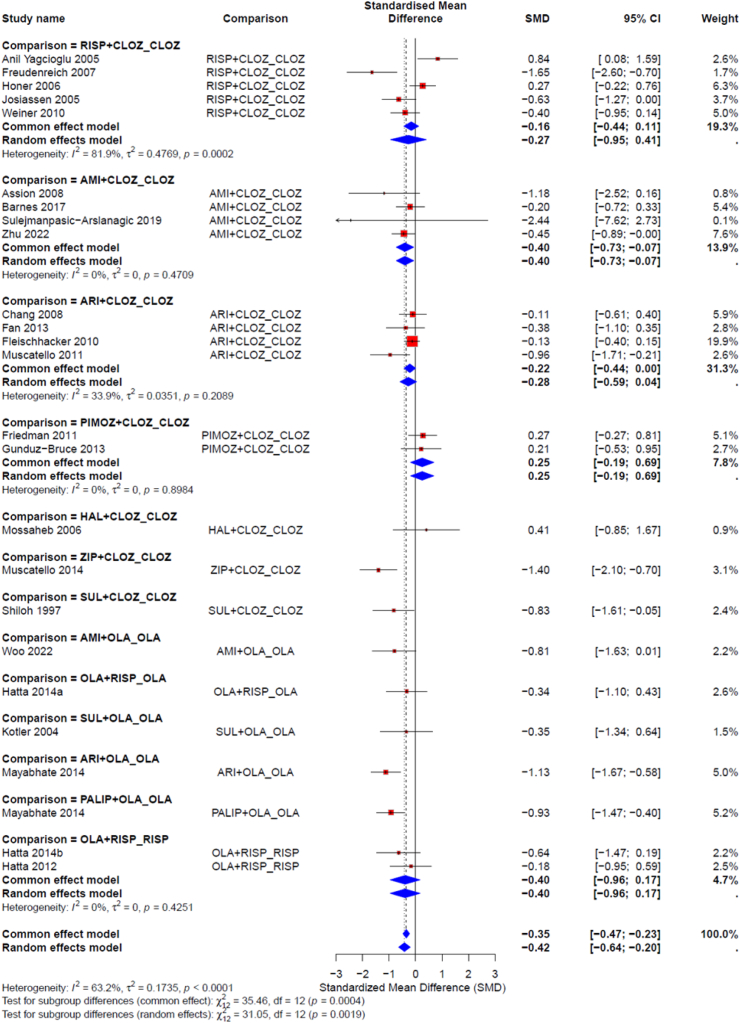
Pairwise meta-analysis of all antipsychotic combination trials versus antipsychotic monotherapy trials per comparison. The size of squares reflects the weight attributed to each study. The diamonds illustrate the summary effect sizes for individual comparisons and for the combined analysis of all interventions. The centre of each diamond indicates the summary effect size, and the width of the diamond represents the confidence interval’s (CI) range. The last comparison (combined) presents the summary effect size for the pairwise meta-analysis of all antipsychotic combination trials versus all antipsychotic monotherapy trials. Error bars indicate 95% CI; SMD is standardized mean difference. AMI: amisulpride; ARI: aripiprazole; CLOZ: clozapine; HAL: haloperidol; OLA: olanzapine; PALIP: paliperidone; PIMOZ: pimozide; RISP: risperidone; SUL: sulpiride; ZIP: ziprasidone.

Multiple meta-regression, subgroup and sensitivity analyses did not substantially alter the results apart from baseline illness severity which showed that higher baseline severity was associated with higher clozapine efficacy (Appendixes 15 and 16). For the degree of treatment resistance, four subgroups of studies were formed based on the reported criteria (as described in the Methods section). No significant differences in efficacy were found among antipsychotics within any of these subgroups, likely due to the limited data available per group and comparison (Fig. 4, Appendix 15.1). The most notable difference was that the subgroup with the more relaxed criteria for treatment resistance included drug combinations such as oxytocin plus risperidone, amisulpride plus olanzapine, aripiprazole plus olanzapine, and paliperidone plus olanzapine, which had no data in any of the other subgroups. Grouping treatments by their primary mechanism of action showed that combining an antipsychotic with an antiepileptic or combining two antipsychotics was more effective than using an antipsychotic alone (Appendix 15.3), but trials with antiepileptics were very heterogeneous (Appendix 17.3). Combinations of antipsychotics demonstrated superiority over antipsychotic monotherapy, even in conventional pairwise meta-analysis (Fig. 5).

A comparison of changes in overall symptoms between all treatments and clozapine, using a contour-enhanced funnel plot and Egger’s test, indicated that smaller trials tended to exaggerate treatment efficacy compared to clozapine (Appendix 18). Excluding the quartile of studies with the smallest sample sizes did not alter the results or the treatment hierarchy (Appendix 16.1).

## Discussion

To our knowledge, this study is the largest NMA on the pharmacological treatment of TRS, evaluating 78 different drug options or placebo, including both monotherapy and combination treatments. The analysis included 150 RCTs with 11,375 participants with varying levels of treatment resistance and examined multiple efficacy and safety outcomes. Clozapine was more efficacious than some other antipsychotics. However, a few of its combinations were superior to clozapine monotherapy. Data on non-clozapine combinations were scarce.

Clozapine monotherapy outperformed chlorpromazine, quetiapine, and haloperidol in treating total symptoms, in line with previous meta-analyses.^15^^,^^16^ Additionally, clozapine showed slight superiority over olanzapine for positive symptoms and a marginally higher response rate compared to risperidone. Further supporting these findings, clozapine outperformed fluphenazine, haloperidol, quetiapine, olanzapine, risperidone, sertindole, and placebo in terms of dropouts due to inefficacy.

Combinations of clozapine with amisulpride, duloxetine, memantine, mirtazapine, topiramate, and ziprasidone enhanced its efficacy. For the antidepressants duloxetine and mirtazapine, as well as memantine and ziprasidone, the improvements were primarily in negative symptoms, while combinations with amisulpride, lamotrigine, and topiramate demonstrated superiority over clozapine monotherapy in reducing positive symptoms.

All-cause discontinuation can serve as an indicator of both efficacy and tolerability. Among treatment options, only the combination of amisulpride with clozapine outperformed clozapine monotherapy. While combining topiramate with clozapine demonstrated greater efficacy than clozapine alone in improving total and positive symptoms, it was associated with a higher dropout rate due to any reason as well as more adverse events. Additionally, all-cause discontinuation was higher for chlorpromazine, fluphenazine, haloperidol, quetiapine, thioridazine, and trifluoperazine compared to clozapine monotherapy.

Antipsychotic combinations demonstrated promising results, even in conventional pairwise meta-analysis (Fig. 5). The combination of clozapine with amisulpride, in particular, showed potential benefits across multiple outcomes. Similarly, combinations of clozapine with aripiprazole, ziprasidone, and sulpiride also appeared promising; however, the limited number of trials and participants underscores the need for further research. Data remain unavailable for other partial D2 agonists. While it is plausible that combining antipsychotics with distinct receptor profiles may yield beneficial effects—and large cohort studies have suggested that such combinations might reduce rehospitalization risk^65^—high-quality RCT evidence is still lacking.

Non-clozapine combinations, particularly olanzapine with aripiprazole or paliperidone, showed potential superiority to clozapine monotherapy. However, this finding is based on limited data from a single study of 90 participants with a very loose definition of treatment resistance, i.e., having failed only one prior antipsychotic trial.^66^ The question of whether combinations of non-clozapine antipsychotics could serve as an alternative to clozapine remains underexplored in RCTs, despite clinicians often favoring this approach before initiating clozapine.^67^^,^^68^ Up to date, most RCTs exploring polytherapy have focused on the combination of clozapine with other antipsychotics in highly treatment-resistant cases. Meanwhile, real-world effectiveness studies using extensive registry-based data highlight some benefits of olanzapine combinations with other antipsychotics.^65^^,^^69^

An observational study of 1543 acute schizophrenia patients reported an 89.8% response rate to non-clozapine antipsychotic combinations after two failed trials,^70^ far exceeding the 40% response to clozapine reported among RCTs.^8^ The discrepancy between clinical guidelines, which unanimously recommend clozapine monotherapy following two antipsychotic failures, and everyday clinical practice, where antipsychotic polytherapy is frequently employed before a clozapine trial,^14^^,^^67^^,^^71^^,^^72^ suggests that combining non-clozapine antipsychotics may be a valuable strategy for patients who do not respond to monotherapy. Despite the limited number of RCTs per comparison, our data tend to support this for combinations of olanzapine with other antipsychotics like amisulpride, aripiprazole and paliperidone. However, there is also evidence suggesting that delaying clozapine treatment may increase the clozapine non-response rate in TRS patients.^73^, ^74^, ^75^ Therefore, advocating for the consideration of combination interventions before a clozapine trial should be approached cautiously and balanced against the potential drawbacks of delaying clozapine initiation. This is particularly important given the limited evidence on the efficacy and safety of combination treatments, highlighting the need for further research to clarify their role in TRS management.

The augmentation of risperidone with oxytocin also demonstrated greater efficacy in reducing both total and positive symptoms of schizophrenia compared to clozapine monotherapy. However, this result was based on a single small-scale trial with a high risk of bias involving patients unresponsive to risperidone alone,^76^ which limits the generalizability and robustness of the findings. Over the past two decades, there has been significant interest in intranasal oxytocin as an adjunctive therapy for various psychiatric disorders.^77^, ^78^, ^79^ However, recent meta-analyses in schizophrenia, including up to 10 RCTs, have not provided convincing evidence that intranasal oxytocin improves core schizophrenia symptoms.^80^^,^^81^ Therefore, this result should be interpreted with great caution in the absence of large, well-designed RCTs.

Due to several limitations, our study cannot be considered definitive. Meta-analyses of RCTs have consistently struggled to demonstrate clozapine’s superiority over most second generation antipsychotics (SGAs),^15^^,^^16^ despite real-world evidence and clinical experience suggesting otherwise.^82^^,^^83^ While expectation bias in open-label RCTs might contribute to findings in favor of clozapine, real-world observational studies and population-based register studies have validated these results, even for hard endpoints such as hospitalization, mortality rates^82^^,^^83^ and inpatient days.^84^ Although clozapine shows a rapid response profile, similar to other antipsychotics but of greater magnitude,^85^ longer trial durations (6–12 months) might be needed to detect differences in outcomes like functioning and quality of life.^86^ Most RCTs, however, are of short duration, and outcomes such as quality of life, functioning, suicide, and aggression—where clozapine often excels—are frequently unexamined in RCTs, as seen in our analysis. Real-world evidence, by contrast, tends to focus on these important outcomes.

Moreover, increased monitoring for clozapine-treated patients (e.g., regular blood tests) could contribute some bias in the observational studies. Effect sizes might be inflated since patients with better adherence profiles and supportive environment are often prescribed clozapine, while for the remaining patients an LAI might be preferred. Another potential explanation for the lack of clear superiority of clozapine in RCTs is that patients who would benefit most from clozapine—those with severe treatment-resistant disease,^15^ acute suicidality,^87^^,^^88^ or high aggression^89^^,^^90^ — are often underrepresented in blinded RCTs, introducing a sampling bias.

Dose-response meta-analyses have indicated that increasing doses of certain antipsychotics, such as clozapine and olanzapine, can improve efficacy.^91^ Most guidelines emphasise achieving adequate clozapine plasma concentrations (>350 ng/mL), especially in cases of non-response, to mitigate pseudoresistance.^92^^,^^93^ However, plasma levels were not reported in most studies included in our analysis. Although the meta-regression using dose as a moderator variable found no effect on treatment efficacy, the statistical power of this analysis was notably low. In recent RCTs, clozapine dosing has decreased. In our analysis, the median dose for trials comparing clozapine to other SGAs was 343 mg, whereas for trials comparing clozapine to FGAs, it was 525 mg. Notably, continuously increasing efficacy beyond 600 mg/day has been shown,^94^ but RCT data are too limited to draw a definitive conclusion.^84^^,^^95^

In clinical practice, due to limited effective alternatives, clinicians often continue clozapine treatment, waiting, increasing to the highest tolerable dose or augmenting as necessary, frequently adding anticonvulsants—primarily valproic acid—to mitigate seizure risk of high dosages. Similarly, clinicians may re-challenge patients with clozapine multiple times, while in an RCT setting, such patients would typically drop out and not re-enter. While some patients may not respond adequately without plasma levels >550 ng/mL, the benefits must be balanced against the heightened risk of adverse effects.^96^ Consequently, dose variability may serve as a confounding factor in our findings.

Our inclusion of diverse definitions of TRS, ranging from partial non-response to a single antipsychotic to ultra-resistant cases, also means that patients differed in illness severity. Our goal was to provide a comprehensive overview of pharmacological interventions for TRS as it is managed in real-world clinical practice, where treatment resistance exists on a spectrum, and patients often become increasingly resistant as the disease progresses,^97^ but this decision also introduced complexity in the interpretation of the results. Subgroup analyses based on levels of resistance did not reveal significant differences from the main analysis, though this may stem from limited data per intervention. Individual antipsychotics might still perform differently depending on level of resistance. Moreover, baseline illness severity was identified as a predictor of clozapine efficacy in meta-regression analyses. As recently recommended,^98^ standardized definitions of TRS would improve consistency, comparability and reproducibility across studies, and thereby allow clinical guidelines to provide more targeted recommendations for truly treatment-resistant cases.

Direct evidence for many interventions was derived from only a few studies—sometimes just a single study—leading to two key limitations: (a) limited statistical power to detect potential differences and (b) a high risk of outlier results due to a lack of closed loops in the network. This reduces the benefits of the NMA design and increases the risk of high effect sizes due to chance. For example, this applies to combinations of clozapine with antidepressants and anticonvulsants, as well as specific combinations like oxytocin with risperidone and olanzapine with either aripiprazole or paliperidone. These combinations, although ranked highly in the efficacy hierarchy, were primarily supported by indirect evidence. The wide PIs further highlight the uncertainty surrounding the true treatment effects in future studies. Thus, interpretation requires caution, considering the number of trials and participants for each intervention together with the overall confidence in estimates.

Additional RCTs with longer durations, including outcomes such as functioning and quality of life, and comparing antipsychotic augmentation strategies with high-dose monotherapy, are urgently needed. Furthermore, non-clozapine antipsychotic combinations should be directly compared to clozapine, particularly in studies using LAIs, which may offer greater adherence benefits and have shown superior efficacy in cohort studies. It is also noteworthy that only four out of the 150 included studies investigated the combination of an LAI antipsychotic with another drug, and none examined the combination of an LAI with clozapine—a strategy that warrants further exploration and discussion given its potential to improve adherence and outcomes in TRS.

While clinical guidelines generally discourage antipsychotic polypharmacy, routine clinical practice often involves combining medications to address specific clinical needs. Although meta-analyses of low-quality studies have yielded mixed results and RCT data remain scarce, real-world evidence suggests potential benefits. In light of this, clozapine monotherapy continues to be the gold standard for TRS; however, guidance on next steps in cases of clozapine non-response remains uncertain. Therefore, the consideration of the patient’s clinical profile and specific drug combinations should be individualized and nuanced. Clinicians must carefully weigh the potential benefits of combination strategies against the risk of adverse effects. In resource-limited settings, the financial and logistical burden of frequent monitoring and polypharmacy should also be factored into clinical decision-making.

## Contributors

MS conceptualized and planned the study, with input from ASL, BH, SS, NC, and SL. MS and ASL managed and coordinated the research activity planning and execution. EP, EG, and IF screened the literature and extracted the data. CC and DM did the statistical analysis. MS wrote the first draft of the report with important input from ASL and SL. All authors critically reviewed and commented on the manuscript. Αll authors read and approved the final version of the manuscript. All authors had full access to all the data in the study and had final responsibility for the decision to submit for publication.

## Data sharing statement

The data that support the findings of this study are available from the corresponding author, Prof. Myrto Samara, upon reasonable request.

## Declaration of interests

MS has received honoraria as a consultant/advisor and/or for lectures from Recordati, Lundbeck and Viatris. SL has received honoraria as a consultant/advisor and/or for lectures from Angelini, Böhringer Ingelheim, Geodon and Richter, Janssen, Johnson&Johnson, Lundbeck, LTS Lohmann, MSD, Otsuka, Recordati, Sanofi Aventis, Sandoz, Sunovion, TEVA, Eisai, Rovi, Medichem, and Mitsubishi. All other authors declare no financial relationships with commercial interests.

## Appendix ASupplementary data

Appendixes 1–19
