# Supplementary material for: Efficacy and tolerability of pharmacological interventions for schizophrenia non-responsive to prior treatment: a systematic review and network meta-analysis
Source: eClinicalMedicine. 2025 Jun 7;84:103291. doi: 10.1016/j.eclinm.2025.103291 (PMC12173738; doi:10.1016/j.eclinm.2025.103291)
Supplement: Appendixes 1–19 [file mmc1.pdf]

Appendix

Table of Contents

Appendix 1. PRISMA checklist ... 5
Appendix 2. Protocol of the systematic review and network meta-analysis ... 9
'Network meta-analysis of drug options in case of non-response in schizophrenia' ... 9
Contributors ... 9
Abstract ... 9
2.1. Study information ... 10
2.2. Design plan ... 10
2.3. Variables ... 11
2.4. Analysis plan ... 11
2.5. Other ... 13
Appendix 3. Search strategy ... 14
3.1. Sample search strategy for Embase ... 14
Appendix 4. PRISMA flow diagram ... 16
Appendix 5. Included studies: Monotherapy (Table S 5) ... 17
Appendix 6. Included studies: Add-on (Table S 6) ... 34
Appendix 7. Risk of bias: Summary and traffic plots (Figure S 7.1.; Figure S 7.2.) ... 53
Appendix 8: Statistical methods in detail ... 58
Appendix 9. Transitivity assessment ... 59
9.1. Baseline severity of treatment resistance schizophrenia ... 59
9.2. Year of publication ... 60
9.3. Mean age of participants ... 61
9.4. Percentage of male participants ... 62
Appendix 10. Complete list of graphs and plots for all outcomes ... 63
10.1. Overall change in symptoms (Figure S 10.1a) ... 63
10.2. Positive symptoms (Figure S 10.2a; Figure S 10.2b; Figure S 10.2c) ... 64
10.3. Negative symptoms (Figure S 10.3a; Figure S 10.3b; Figure S 10.3c) ... 66
10.4. Depressive symptoms (Figure S 10.4a; Figure S 10.4b; Figure S 10.4c) ... 68
10.5. Response rates (Figure S 10.5a; Figure S 10.5b; Figure S 10.5c) ... 70
10.6. Drop-outs due to any reason (Figure S 10.6a; Figure S 10.6b; Figure S 10.6c) ... 72
10.7. Drop-outs due to any adverse effects (Figure S 10.7a; Figure S 10.7b; Figure S 10.7c) 74
10.8. Drop-outs due to inefficacy (Figure S 10.8a; Figure S 10.8b; Figure S 10.8c) ... 76
10.9. Total number of participants with adverse effects (Figure S 10.9a; Figure S 10.9b; Figure S 10.9c) ... 78

10.10. Antiparkinsonian medication use (Figure S 10.10a; Figure S 10.10b; Figure S 10.10c) 80
10.11. Sedation (Figure S 10.11a; Figure S 10.11b; Figure S 10.11c) 82
10.12. Weight gain (Figure S 10.12a; Figure S 10.12b; Figure S 10.12c) 84
10.13. Prolactin elevation (Figure S 10.13a; Figure S 10.13b; Figure S 10.13c) 86
10.14. QTc prolongation (Figure S 10.14a; Figure S 10.14b; Figure S 10.14c) 88
10.15. Quality of life (Figure S 10.15a; Figure S 10.15b; Figure S 10.15c) 90
10.16. Social functioning (Figure S 10.16a; Figure S 10.16b; Figure S 10.16c) 92
Appendix 11. League tables: Primary and secondary outcomes 94
11.1. League table: Overall symptoms 95
11.2. League table: Positive symptoms 97
11.3. League table: Negative symptoms 99
11.4. League table: Depressive symptoms 101
11.5. League table: Response rates 103
11.6. League table: Drop-outs due to any reason 105
11.7. League table: Drop-outs due to any adverse effects 107
11.8. League table: Drop-outs due to inefficacy 109
11.9. League table: Total number of participants with adverse effects 111
11.10. League table: Antiparkinsonian medication use 112
11.11. League table: Sedation 113
11.12. League table: Weight gain 114
11.13. League table: Prolactin elevation 115
11.14. League table: QTc prolongation 116
11.15. League table: Quality of life 117
11.16. League table: Social functioning 118
Appendix 12. Other models for dichotomous outcomes 119
12.1. Other models for dichotomous outcomes: Response rates 119
12.2. Other models for dichotomous outcomes: Drop-outs due to any reason 121
12.3. Other models for dichotomous outcomes: Drop-outs due to any adverse effect 123
12.4. Other models for dichotomous outcomes: Drop-outs due to inefficacy 125
12.5. Other models for dichotomous outcomes: Total number of participants with adverse effects 127
12.6. Other models for dichotomous outcomes: Antiparkinsonian medication use 128
12.7. Other models for dichotomous outcomes: Sedation 129
Appendix 13. Summary of statistical evaluation of heterogeneity and consistency for the network meta-analysis 130
Appendix 14. Confidence evaluation in network meta-analysis: CINeMA 131

14.1. Reasons for downgrading 131
Domain 1: Within-study bias 131
Domain 2: Reporting bias 131
Domain 3: Indirectness 131
Domain 4: Imprecision 131
Domain 5: Heterogeneity 131
Domain 6: Incoherence 132
Overall confidence 132
14.2. CINeMA rating for all comparisons: Overall symptoms primary outcome 133
Appendix 15. Sub-group and meta-regression analysis 234
15.1. Sub-group: Criteria of treatment-resistant definitions 234
15.1.1. Sub-group: Treatment-resistant criteria – Group 1 versus Groups 2 to 4 234
15.1.2. Sub-group: Treatment-resistant criteria – Groups 1 to 2 versus Group 3 to 4 . 235
15.1.3. Sub-group: Treatment-resistant criteria – Groups 1 to 3 versus Groups 4 (ultra resistant) 236
15.2. Sub-group: Residual symptoms 237
15.3. Sub-group: Treatment groups based on their main therapeutic concept (Figure 15.3.1.; Figure 15.3.2) 238
15.4. Sub-group: Sponsorship: Sponsored vs non-sponsored 240
15.5. Meta-regression: Baseline severity 241
15.6. Meta-regression: Publication date 241
15.7. Meta-Regression*:* Age 242
15.8. Meta-regression: Percentage of males 242
15.9. Meta-regression: Dose ratio 243
15.10. Meta-regression: Duration of illness 243
15.11. Meta-regression: Duration of trial 244
15.12. Multivariable meta-regression analyses 244
Appendix 16. Sensitivity analyses 245
16.1. Exclusion of the first quartile of trials with the smallest sample sizes (Figure S 16.1a; Figure S 16.1b) 245
16.2. Double blind randomised control trials only (Figure S 16.2a; Figure S 16.2b) 247
16.3. Exclusion of randomised control trials that did not employ operationalized criteria for schizophrenia diagnosis (Figure S 16.3a; Figure S 16.3b) 249
16.4. Exclusion of randomised control trials including intolerant patients (Figure S 16.4a; Figure S 16.4b) 251
16.5. Exclusion of randomised control trials with high risk of bias (Figure S 16.5a; Figure S 16.5b) 253

16.6. Assumption of missing data (Figure S 16.6a; Figure S 16.6b) 255

16.7. Country: Trials from developed countries only (Figure S 16.7a; Figure S 16.7b) 257

16.8. Common-effect model (Figure S 16.8) 259

16.9. Extreme sensitivity analysis excluding the following studies: Open-label, intolerant patients, and low and medium stringency of resistance criteria (Figure S 16.9a; Figure S 16.9b) 260

Appendix 17. Pairwise meta-analysis of drug groups 262

17.1. Combination of clozapine with another antipsychotic vs clozapine monotherapy 262

17.2. Combination of olanzapine with another antipsychotic versus antipsychotic monotherapy 263

17.3. Antipsychotics augmentation with antiepileptics versus antipsychotic monotherapy 263

Appendix 18. Assessment of publication bias: Contour-enhanced funnel plot 264

Appendix 19. References 265

Appendix 1. PRISMA checklist

Section/Topic	Item #	Checklist Item	Reported on Page #
**TITLE**			
**Title**	1	Identify the report as a systematic review *incorporating a network meta-analysis (or related form of meta-analysis).*	1
**ABSTRACT**			
**Structured summary**	2	Provide a structured summary including, as applicable:<br>**Background:** main objectives<br>**Methods:** data sources; study eligibility criteria, participants, and interventions; study appraisal; and *synthesis methods, such as network meta-analysis.*<br>**Results:** number of studies and participants identified; summary estimates with corresponding confidence/credible intervals; *treatment rankings may also be discussed. Authors may choose to summarize pairwise comparisons against a chosen treatment included in their analyses for brevity.*<br>**Discussion/Conclusions:** limitations; conclusions and implications of findings.<br>**Other:** primary source of funding; systematic review registration number with registry name.	2
**INTRODUCTION**			
**Rationale**	3	Describe the rationale for the review in the context of what is already known, *including mention of why a network meta-analysis has been conducted.*	5
**Objectives**	4	Provide an explicit statement of questions being addressed, with reference to participants, interventions, comparisons, outcomes, and study design (PICOS).	5,<br>Appendix 2
**METHODS**			
**Protocol and registration**	5	Indicate whether a review protocol exists and if and where it can be accessed (e.g., Web address); and, if available, provide registration information, including registration number.	Appendix 2, OSF (https://doi.org/10.17605/OSF.IO/4AJUR)
**Eligibility criteria**	6	Specify study characteristics (e.g., PICOS, length of follow-up) and report characteristics (e.g., years considered, language, publication status) used as criteria for eligibility, giving rationale. *Clearly describe eligible treatments included in the treatment network, and note whether any have been clustered or merged into the same node (with justification).*	5 & 6,<br>Appendix 2
**Information sources**	7	Describe all information sources (e.g., databases with dates of coverage, contact with study authors to identify additional studies) in the search and date last searched.	5,<br>Appendix 3
**Search**	8	Present full electronic search strategy for at least one database, including any limits used, such that it could be repeated.	5,<br>Appendix 3

**Study selection**	9	State the process for selecting studies (i.e., screening, eligibility, included in systematic review, and, if applicable, included in the meta-analysis).	5 & 6, Appendix 2
**Data collection process**	10	Describe method of data extraction from reports (e.g., piloted forms, independently, in duplicate) and any processes for obtaining and confirming data from investigators.	6, Appendix 2
**Data items**	11	List and define all variables for which data were sought (e.g., PICOS, funding sources) and any assumptions and simplifications made.	6 & 7, Appendix 2
**Geometry of the network**	**S1**	Describe methods used to explore the geometry of the treatment network under study and potential biases related to it. This should include how the evidence base has been graphically summarized for presentation, and what characteristics were compiled and used to describe the evidence base to readers.	6 & 7, Appendix 2 & 8
**Risk of bias within individual studies**	12	Describe methods used for assessing risk of bias of individual studies (including specification of whether this was done at the study or outcome level), and how this information is to be used in any data synthesis.	6, Appendix 2 & 7
**Summary measures**	13	State the principal summary measures (e.g., risk ratio, difference in means). *Also describe the use of additional summary measures assessed, such as treatment rankings and surface under the cumulative ranking curve (SUCRA) values, as well as modified approaches used to present summary findings from meta-analyses.*	6, Appendix 2 & 8
**Planned methods of analysis**	14	Describe the methods of handling data and combining results of studies for each network meta-analysis. This should include, but not be limited to:<br>• *Handling of multi-arm trials;*<br>• *Selection of variance structure;*<br>• *Selection of prior distributions in Bayesian analyses; and*<br>• *Assessment of model fit.*	6 & 7, Appendix 2 & 8
**Assessment of Inconsistency**	**S2**	Describe the statistical methods used to evaluate the agreement of direct and indirect evidence in the treatment network(s) studied. Describe efforts taken to address its presence when found.	6 & 7, Supplement 2, 8 & 13
**Risk of bias across studies**	15	Specify any assessment of risk of bias that may affect the cumulative evidence (e.g., publication bias, selective reporting within studies).	7, Supplement 14 & 18
**Additional analyses**	16	Describe methods of additional analyses if done, indicating which were pre-specified. This may include, but not be limited to, the following:<br>• Sensitivity or subgroup analyses;<br>• Meta-regression analyses;<br>• *Alternative formulations of the treatment network; and*<br>• *Use of alternative prior distributions for Bayesian analyses (if applicable).*	6 & 7, Appendix 2, 15 & 16

RESULTS†			
**Study selection**	17	Give numbers of studies screened, assessed for eligibility, and included in the review, with reasons for exclusions at each stage, ideally with a flow diagram.	7, Appendix 4, PRISMA
**Presentation of network structure**	**S3**	Provide a network graph of the included studies to enable visualization of the geometry of the treatment network.	Figure 1, Appendix 10
**Summary of network geometry**	**S4**	Provide a brief overview of characteristics of the treatment network. This may include commentary on the abundance of trials and randomized patients for the different interventions and pairwise comparisons in the network, gaps of evidence in the treatment network, and potential biases reflected by the network structure.	7, Appendix 10
**Study characteristics**	18	For each study, present characteristics for which data were extracted (e.g., study size, PICOS, follow-up period) and provide the citations.	7, Appendix 5 & 6
**Risk of bias within studies**	19	Present data on risk of bias of each study and, if available, any outcome level assessment.	Appendix 7
**Results of individual studies**	20	For all outcomes considered (benefits or harms), present, for each study: 1) simple summary data for each intervention group, and 2) effect estimates and confidence intervals. *Modified approaches may be needed to deal with information from larger networks.*	Figure 2, 3, 4, 5 Appendix 10 & 17
**Synthesis of results**	21	Present results of each meta-analysis done, including confidence/credible intervals. *In larger networks, authors may focus on comparisons versus a particular comparator (e.g. placebo or standard care), with full findings presented in an Appendix. League tables and forest plots may be considered to summarize pairwise comparisons.* If additional summary measures were explored (such as treatment rankings), these should also be presented.	Figure 2, 3, 4, 5 Appendix 10, 11 (League tables), & 17
**Exploration for inconsistency**	**S5**	Describe results from investigations of inconsistency. This may include such information as measures of model fit to compare consistency and inconsistency models, *P* values from statistical tests, or summary of inconsistency estimates from different parts of the treatment network.	9, Appendix 13
**Risk of bias across studies**	22	Present results of any assessment of risk of bias across studies for the evidence base being studied.	9, Appendix 14
**Results of additional analyses**	23	Give results of additional analyses, if done (e.g., sensitivity or subgroup analyses, meta-regression analyses, *alternative network geometries studied, alternative choice of prior distributions for Bayesian analyses,* and so forth).	9 & 10, Appendix 12, 15 & 16

**DISCUSSION**			
**Summary of evidence**	24	Summarize the main findings, including the strength of evidence for each main outcome; consider their relevance to key groups (e.g., healthcare providers, users, and policy-makers).	10, 11
**Limitations**	25	Discuss limitations at study and outcome level (e.g., risk of bias), and at review level (e.g., incomplete retrieval of identified research, reporting bias). *Comment on the validity of the assumptions, such as transitivity and consistency. Comment on any concerns regarding network geometry (e.g., avoidance of certain comparisons).*	11, 12
**Conclusions**	26	Provide a general interpretation of the results in the context of other evidence, and implications for future research.	12, 13
**FUNDING**			
**Funding**	27	Describe sources of funding for the systematic review and other support (e.g., supply of data); role of funders for the systematic review. This should also include information regarding whether funding has been received from manufacturers of treatments in the network and/or whether some of the authors are content experts with professional conflicts of interest that could affect use of treatments in the network.	2, 14

Appendix 2. Protocol of the systematic review and network meta-analysis

'Network meta-analysis of drug options in case of non-response in schizophrenia'

Contributors
Myrto Samara, Andreas Lappas, Elisavet Pinioti, Eleni Glarou, Iwo Fober, Spyridon Siafis, Nikolaos Christodoulou, Bartosz Helfer, Stefan Leucht

Abstract

Introduction In the context of treatment-resistant schizophrenia, clozapine stands as the standard treatment. Nonetheless, its underutilization is linked to the numerous adverse effects it presents, leading clinicians to often prefer a combination of non-clozapine antipsychotics. Furthermore, there are instances where even clozapine proves to be inefficacious. Therefore, it is imperative to investigate whether alternative treatment options could demonstrate efficacy. **Method and analysis** This meta-analysis will adhere to the Preferred Reporting Items for Systematic Reviews and Meta-Analyses Protocols guidelines. A systematic search will be conducted in MEDLINE, Cochrane Central Register of Controlled Trials, Embase, PsycINFO, US National Institute of Health Ongoing Trials Register (ClinicalTrials.gov), and the World Health Organization International Clinical Trials Registry Platform (ICTRP) (www.who.int/ictrp) for randomized controlled trials examining antipsychotics alone or in combination with other drugs for patients with treatment-resistant schizophrenia. Article screening and data extraction will be independently performed by at least two authors. The data will be synthesized using a network-meta-analysis that incorporates all interventions. Subgroup and sensitivity analyses will be employed to explore potential heterogeneity. **Ethics and dissemination** No ethical review is required for this systematic review. The study's results will be presented at major international psychiatric conferences and published in a peer-reviewed psychiatric journal.

Keywords: nonresponse, augmentation, combination, antipsychotic

Registration type: OSF Preregistration

Date registered: August 9, 2024

Date created: August 9, 2024

Associated project: osf.io/7jd64

Internet Archive link: https://archive.org/details/osf-registrations-4ajur-v1

Category: Project

Registration DOI: https://doi.org/10.17605/OSF.IO/4AJUR

Subjects:

- Mental and Social Health
- Medicine and Health Sciences
- Psychiatric and Mental Health
- Psychiatry and Psychology

License: CC-By Attribution 4.0 International

2.1. Study information

Hypotheses

Antipsychotic drugs constitute the cornerstone of schizophrenia treatment, with their efficacy well-documented in numerous randomized controlled trials (RCTs) [1]. However, despite adequate antipsychotic pharmacotherapy, up to 43% of schizophrenia patients do not even minimally improve in short-term RCTs, and approximately 67% fail to achieve symptom remission [2]. Some individuals display treatment resistance from the onset of psychosis [3,4], while others develop resistance over time [5], with prevalence rates ranging from 15% to 76%, depending on the examined population [6]. Clozapine is presently considered the gold standard for refractory patients, although a comprehensive network meta-analysis has questioned its superiority over other second-generation antipsychotics, mainly olanzapine [7]. Additionally, clozapine is grossly underutilized, which is largely attributed to its side-effect profile [8]. In clinical practice, alternative strategies to clozapine, such as combining antipsychotics or augmenting with mood stabilizers, antiepileptics, or antidepressants, are often preferred. Nevertheless, the evidence base supporting these approaches remains unclear. Additionally, when clozapine is initiated, it is commonly paired directly with another antipsychotic [9] before an adequate trial of clozapine monotherapy, as suggested by guidelines. Clinicians combine clozapine with numerous antipsychotics, despite the fact that, in a cohort study, only the combination with aripiprazole has shown some evidence of greater efficacy than clozapine monotherapy [7,9–11]. Therefore, identifying any intervention with equal or superior efficacy to clozapine monotherapy for refractory patients is of paramount importance. This systematic review and network meta-analysis aims to examine the comparative efficacy and safety of all antipsychotic drugs, either as monotherapy, or in combinations with another antipsychotic or any other drug. Notably, this inclusive approach may uncover old "clinical pearls", such as perphenazine and sulpiride, which exhibited promising results in effectiveness studies [12,13], or novel combinations, even with non-psychiatric drugs, that could prove efficacious. Moreover, any definition of treatment resistance or nonresponse will be considered allowing us to explore whether the degree of resistance modifies treatment hierarchies. Such a comprehensive attempt to include all antipsychotic drugs and their combinations in patients who do not respond to antipsychotics in a review is unprecedented.

2.2. Design plan

Study type

Meta-Analysis - A systematic review of published studies.

Blinding

No blinding is involved in this study.

Is there any additional blinding in this study?

No

Study design

Network Meta-analysis

Randomization

Does not apply

Sampling plan

Existing data

Registration prior to analysis of the data

Explanation of existing data

No response

Data collection procedures

We will run a literature search of the following databases: MEDLINE (via Ovid), CENTRAL (Cochrane Central Register of Controlled Trials), Embase, PsycINFO, US National Institute of Health Ongoing Trials Register (ClinicalTrials.gov), and the World Health Organization International Clinical Trials Registry Platform (ICTRP) (www.who.int/ictrp). Reference lists of previously published systematic reviews on the topic [14–16] as well as previous reviews from our team [7,11] will be inspected to ensure that no eligible articles are missed. We will also manually review the references of all identified studies and we will contact the first/corresponding author of each included study for missing information and information regarding unpublished trials. We will document any response.

Sample size
Does not apply

Sample size rationale
No response

Stopping rule
No response

2.3. Variables

Manipulated variables
Does not apply

Measured variables

Primary outcome

Average change or endpoint in score on a general mental state scale (e.g., PANSS, BPRS). As not all studies use the same scales, we will apply the following hierarchy: (i) mean change of the PANSS total score from baseline to endpoint; (ii) if this is not available, we will use the mean change of the BPRS; (iii) if this is also not available, we will use the mean values at endpoint of either the PANSS or the BPRS. The results of other rating scales will only be used if the instrument has been published in a peer-reviewed journal.

Secondary outcomes

1. Clinically relevant response to treatment as defined by the trials (dichotomous). The following hierarchy of the response definitions will be applied: at least 20% reduction of the baseline score of the PANSS, 20% reduction of the BPRS or 20% reduction of any other global schizophrenia rating scale, at least "minimally improved" (score of 3) on the Clinical Global-Impressions-Improvement Scale (CGI). We choose this cut-off because even minimal improvement can be clinically important for treatment-resistant patients. If none of these definitions is available, we will use the original authors' primary definition.
2. Dropout due to any reason, as a means of global assessment of acceptability.

Other (secondary) outcomes will include: (i) average change or endpoint in score on a positive symptom scale;(ii) average change or endpoint in score on a negative symptom scale; (iii) average change or endpoint in score on a depressive symptom scale; (iv) average change or endpoint score on quality of life scales; (v) average change or endpoint score on functioning scales; (vi) general tolerability (total number of patients with adverse events, dichotomous outcome); (vii) specific reasons for dropping out (due to inefficacy of treatment and due to adverse events); (viii) specific adverse events, i.e. use of antiparkinsonian medication (dichotomous outcome), weight gain (kg, continuous outcome), sedation (dichotomous outcome), prolactin levels (ng/mL, continuous outcome), and QTc prolongation (continuous outcome).

Indices
No response

2.4. Analysis plan

Statistical models

1. Conventional pair wise meta-analyses will precede the network meta-analyses, which is a widely accepted strategy.
2. A comprehensive network-meta-analysis of all interventions examined is the proposed strategy for information synthesis. Due to the potential heterogeneity of interventions and comparators, transitivity and heterogeneity might be a concern. In this case, separate networks for individual categories of interventions may be presented.
3. For binary (dichotomous) outcomes we will calculate a standard estimation of the odds ratio (OR) and its 95% confidence interval (CI). This will be supplemented by calculations of the number needed to treat for an additional beneficial outcome (NNTB) and the number needed to treat for an additional harmful outcome (NNTH), with their CIs.
4. For continuous outcomes we will estimate mean differences (MD) between groups. We prefer not to calculate effect size measures such as standardized mean difference (SMD). However, if different scales are

used among the studies, and even if these are relatively similar to each other, we will presume that there is a small difference in measurement, and we will calculate SMDs instead of MDs.

5. Heterogeneity will be investigated by visual inspection of the forest plots, by applying the χ^2 test and calculating the I^2 value. Reasons for heterogeneity will be explored. We will use a random-effects model, assuming equal heterogeneity across all comparisons.
6. A key assumption is that the network is "transitive", which indicates that there is an agreement between direct and indirect evidence on the same comparisons. Limiting our sample to treatment-resistant patients increases transitivity. We will also assess transitivity from an epidemiological point of view, by comparing the distribution of possible effect modifiers (i.e. key study characteristics such as publication year, baseline severity, age, percentage male) across studies grouped by comparison. Furthermore, we will perform statistical evaluations of the network consistency, using the design-by-treatment test, and the node-splitting approach (where direct and indirect effect sizes are compared for each comparison in the network). In case of significant inconsistency, we will investigate all possible culprits. Small or moderate amounts of inconsistency will again be further explored by network meta-regression and subgroup analyses using the effect modifiers listed below. If reasonable transitivity can't be assumed, we will only present the pair-wise meta-analytic evaluations. We will estimate the probability for each intervention to be ranked at each possible place, given the relative effect sizes as estimated in NMA. We will obtain a hierarchy of the competing interventions using p-scores.
7. Small-study effects and publication bias for each treatment pair will be assessed using a contour-enhanced funnel plot if at least ten studies are available.

Analysis of subgroups or subsets

We will only perform subgroup/meta-regression analysis for the primary outcome, depending on availability of data. Subgroup analyses (categorical variables) and meta-regressions (continuous variables) will be used to explore possible sources of heterogeneity and inconsistency.

1. Gender - male vs. female
2. Age, especially below 18 years old vs over 65 years old vs. the rest
3. Criteria for treatment resistance and ultra-treatment resistance: until recently, there was no universally accepted definition for treatment resistance. In 2017, the Treatment-Resistant Schizophrenia: Treatment Response and Resistance in Psychosis (TRRIP) consensus criteria were published [17]. However, as highlighted by the authors of this work, more than half of the studies included did not use operationalised criteria. Moreover, the criteria used were different among 95% of the studies, and adherence was not documented in 95% of the studies. We therefore anticipate that the definitions will vary considerably across the included studies and that we won't be able to find sufficient data that would allow us to use even the minimum requirements of the TRIPP consensus criteria as our reference point for the purposes of this work. Based on the above, the results of the primary outcomes will be analysed based on the following three broad levels:
   - Relaxed criteria: definition of non-response without a specification. This will also include studies that included "partially resistant" patients.
   - Intermediate criteria: failure of response to at least two previous antipsychotics.
   - Strict criteria: combination of retrospective and prospective criteria for treatment resistance similar to those used by the pivotal clozapine study by Kane et al. [18] that is no period of good functioning or significant symptomatic relief within preceding 5 years despite at least two 6 weeks courses of antipsychotics; currently BPRS score ≥ 45, score of ≥ 4 on at least two positive symptoms, CGI ≥ 4; and prospectively a failure to respond to 6-week trial of haloperidol [18]
   - Ultra TRS (UTRS): failure to respond to clozapine, however this is identified in the studies
4. Severity of illness at baseline (PANSS or BPRS score at baseline)
5. Dose of the antipsychotics in olanzapine-equivalents according to McAdam et al. [19]
6. Treatment groups according to their main therapeutic concept e.g. antidepressants as add-on treatment, antipsychotic combinations etc
7. Publication date (older studies tend to report larger differences)
8. Studies with predominant specific symptoms (e.g. positive, negative, catatonic)

Sensitivity analysis

We will only perform subgroup analysis for the primary outcomes, depending on availability of data.

1. Exclusion of non-double- blind studies (open and single-blind studies)
2. Exclusion of studies that didn't use operationalised diagnostic criteria

3. Assumptions for missing data: we will exclude studies using completer analyses only
4. Risk of bias: We will analyse the effects of excluding trials judged as having an overall high risk of bias
5. Common-effect model: we will synthesise data using a common-effect model to evaluate whether this alters the significance of the results
6. Studies sponsored by pharmaceutical industry vs. not
7. Exclusion of studies considering failure of a drug trial due to intolerance in their definition of TRS
8. A most extreme sensitivity analysis excluding the following studies: open-label, studies with intolerant patients and studies with low and medium stringency of criteria for resistance.
9. Exclusion of trials from less developed countries (https://www.bmj.com/content/346/bmj.f707)

Transformations
No response

Inference criteria
No response

Data exclusion
No response

Missing data
No response

Exploratory analysis
No response

2.5. Other

Other
No response

Appendix 3. Search strategy

3.1. Sample search strategy for Embase

The search strategy for Embase is detailed below. Variations of this search strategy were tailored to meet the specific requirements of each of the additional databases, namely Ovid Medline, APA PsycInfo, the Cochrane Central Register of Controlled Trials (CENTRAL). Medline, Embase and APA PsychInfo were searched via Ovid. The WHO International Clinical Trials Registry Platform (ICTRP) and the clinical trials register ClinicalTrials.gov were searched individually, using treatment-resistant schizophrenia and all its variations as a keyword.

Database: Embase <1974 to 2025 March 4
Search Strategy:

----

1 randomized controlled trial/
2 controlled clinical trial/
3 or/1-2
4 random$.ti,ab.
5 randomization/
6 intermethod comparison/
7 placebo.ti,ab.
8 (compare or compared or comparison).ti.
9 ((evaluated or evaluate or evaluating or assessed or assess) and (compare or compared or comparing or comparison)).ab.
10 (open adj label).ti,ab.
11 ((double or single or doubly or singly) adj (blind or blinded or blindly)).ti,ab.
12 double blind procedure/
13 parallel group$1.ti,ab.
14 (crossover or cross over).ti,ab.
15 ((assign$ or match or matched or allocation) adj5 (alternate or group$1 or intervention$1 or patient$1 or subject$1 or participant$1)).ti,ab.
16 (assigned or allocated).ti,ab.
17 (controlled adj7 (study or design or trial)).ti,ab.
18 (volunteer or volunteers).ti,ab.
19 human experiment/
20 trial.ti.
21 or/4-20
22 21 or 3
23 (random$ adj sampl$ adj7 (cross section$ or questionnaire$1 or survey$ or database$1)).ti,ab. not (comparative study/ or controlled study/ or randomi?ed controlled.ti,ab. or randomly assigned.ti,ab.)
24 Cross-sectional study/ not (randomized controlled trial/ or controlled clinical study/ or controlled study/ or randomi?ed controlled.ti,ab. or control group$1.ti,ab.)
25 (((case adj control$) and random$) not randomi?ed controlled).ti,ab.
26 (Systematic review not (trial or study)).ti.
27 (nonrandom$ not random$).ti,ab.
28 Random field$.ti,ab.
29 (random cluster adj3 sampl$).ti,ab.
30 (review.ab. and review.pt.) not trial.ti.
31 we searched.ab. and (review.ti. or review.pt.)
32 update review.ab.
33 (databases adj4 searched).ab.
34 (rat or rats or mouse or mice or swine or porcine or murine or sheep or lambs or pigs or piglets or rabbit or rabbits or cat or cats or dog or dogs or cattle or bovine or monkey or monkeys or trout or marmoset$1).ti. and animal experiment/
35 Animal experiment/ not (human experiment/ or human/)
36 or/23-35
37 22 not 36
38 exp psychotherapy/ or exp short term psychotherapy/ or exp interpersonal psychotherapy/ or exp psychodynamic psychotherapy/ or exp body psychotherapy/ or psychotherapy.mp. (302070)

39 37 not 38
40 treatment resistant schizophrenia.mp. or exp treatment-resistant schizophrenia/ (2466)
41 ("treatment resist*" or "therapy resist*" or "drug resist*" or "chemical resist" or "treatment refract*" or "treatment fail*" or nonrespon* or non-respon* or "non respon*" or "not respon*" or "no respon*" or "part* respon*" or "partially respon*" or "incomplete respon*" or "incompletely respon*" or unrespon* or "poorly respon* " or "partial* remi*" or "non-remi*" or "non remi*" or "failed to respond" or "failed to improve" or "failure to respon*" or "failure to improve" or "failed medication*" or "fail* therapy" or refractory or resistant or intolerant or intractable or "difficult to treat" or persistent or chronic or "medication resist*" or residual or deficit).ti,ab,tw.
42 (Schizophrenia or schizoaffective or schizophreniform or "schizophrenia-like" or schiz* or delusion* or psycho* or hallucinat* or paranoi*).ti,ab,tw.
43 41 and 42
44 (hebephreni$ or oligophreni$ or "dementia praecox").ti,ab,tw.
45 ((disorgani* or unorgani* or simple or cataton* or undifferentiated or "not otherwise specified") adj5 schiz*).ti,ab,tw.
46 ((negativ* or cognit* or positive) adj5 symptoms adj5 (schizophrenia or schizoaffective or schizophreniform or "schizophrenia-like" or "schizophrenia like" or "schizophrenia spectrum" or schiz* or delusion* or psycho* or hallucinat* or paranoi*)).ti,ab,tw.
47 41 and 46
48 40 or 43 or 44 or 45 or 47
49 39 and 48
50 antipsychotics.mp. or exp neuroleptic agent/
51 (Antipsychotic* or Neuroleptic or Acepromazine or Acetophenazine or Amisulpride or Aripiprazole or Asenapine or Benperidol or Blonanserin or Brexpiprazole or Bromperidol or Butaperazine or Carpipramine or Carpiprazine or Chlorpromazine or Chlorprothixene or Clocapramine or Clopenthixol or Clopentixol or Clothiapine or Clotiapine or Clozapine or Cyamemazine or Cyamepromazine or Dixyrazine or Droperidol or Fluanisone or Flupehenazine or Flupenthixol or Flupentixol or Fluphenazine or Fluspirilen or Fluspirilene or Haloperidol or Iloperidone or Levomepromazine or Levosulpiride or Loxapine or Loxapinsuccinate or Lurasidone or Melperone or Mepazine or Mesoridazine or Methotrimeprazine or Molindone or Moperone or Mosapramine or Olanzapine or Oxypertine or Paliperidone or Penfluridol or Perazine or Periciazine or Pericyazine or Perospirone or Perphenazine or Pimozide or Pipamperone or Pipothiazine or Pipotiazine or Prochlorperazine or Promazine or Promethazine or Prothipendyl or Quetiapine or Remoxipiride or Reserpine or Riospirone or Risperdal or Risperidone or Seroquel or Sertindole or Stelazine or Sulpiride or Sultopride or Thiopropazate or Thioproperazine or Thioridazine or Tiospirone or Thiothixene or Tiapride or Tiotixene or Trifluoperazine or Trifluperidol or trifluoperidol or Triflupromazine or trifluperazine or Veralipride or Ziprasidone or Zotepine or Zuclopenthixol).mp.

52 or/50-51

53 52 and 49

****

Appendix 4. PRISMA flow diagram

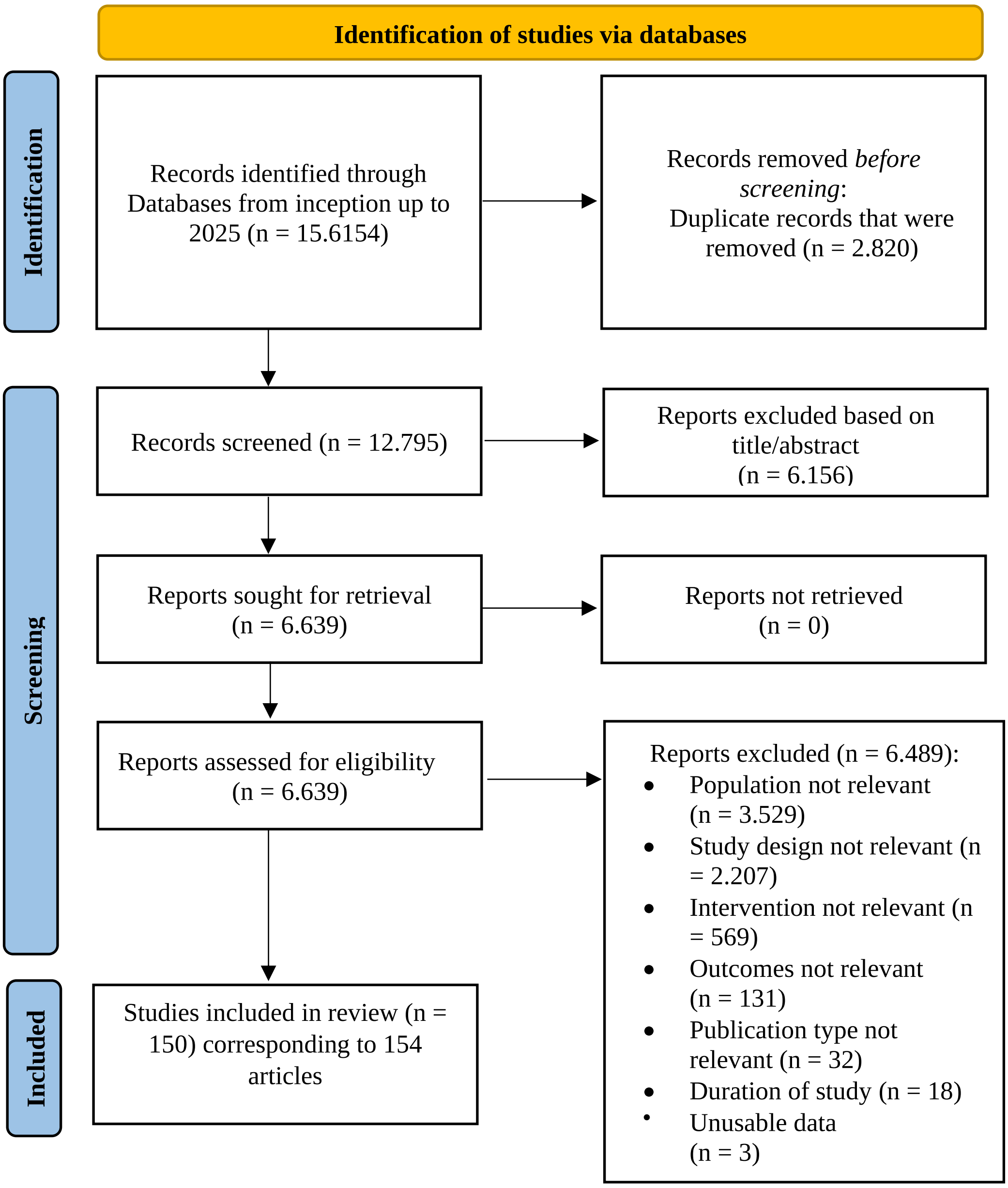

Appendix 5. Included studies: Monotherapy (Table S 5)

Study ID	Study arms; n of participants	Study design	Study duration (weeks)	Country	Mean or range drug doses (mg/d)	Diagnosis	Definition of TRS	Population characteristics
Ahlfors 1980 [20]	CLOPENTHLA, n = 87<br>PERP D, n = 85	parallel double-blind RCT	24	Denmark<br>Finland<br>Norway<br>Sweden	CLOP: 50 to 800 mg/d<br>PERP D: 20 to 600 mg/d	Schizophrenia (classic diagnosis)	*'Hospitalized, mainly classical (Bleuler, 1911) schizophrenic patients were included in the study if they had: 1) a total duration of illness of more than 2 years and 2) unsatisfactory response to present neuroleptic treatment or had been readmitted to hospital because of exacerbation.'*	**Sex:**<br>66.20% males<br>**Mean age (years):**<br>30<br>**Mean duration of illness (years):**<br>n.i.
Altamura 2002 [21]	OLA, n = 13<br>HAL, n = 11	parallel double-blind RCT	14	Canada<br>Italy	OLA: 12.40 mg/d<br>HAL: 12.30 mg/d	Paranoid Schizophrenia (DSM – IV)	*'[…] partial response according to the following criteria: 1. A history of residual negative and/or positive symptoms after at least 6 weeks of therapeutic doses of neuroleptics from different neuroleptic classes; 2. A minimum level of positive and/or negative symptoms at the time of the evaluation of the response as defined by: (a) the BPRS:16 a positive symptoms total score of at least 8 for the items of conceptual disorganization, hallucinations, unusual thought content, and suspiciousness, or a score of at least 4 on any of these items; (b) a total score on the SANS of at least 20, or a score of 2 or more on at least 1 global item on this scale.'*	**Sex:**<br>54.16% males<br>**Mean age (years):**<br>38.84<br>**Mean duration of illness (years):**<br>15.65
AstraZeneca 5077IL/0031 [22]	CPZ, n = 130<br>QUE, n = 130	parallel double-blind RCT	10	Canada<br>USA	CPZ: 50 to 200 mg/d<br>QUE: 25 to 200 mg/d	Schizophrenia (DSM – IV)	*'[…] historical and prospective treatment criteria, were considered resistant to treatment with standard antipsychotics agents.'*	**Sex:**<br>79.20% males<br>**Mean age (years):**<br>40.90<br>**Mean duration of illness (years):**<br>n.i.
AstraZeneca 5077IL/0054 [23]	CPZ, n = 119<br>QUE, n = 117	parallel single-blind RCT	10	n.i.	CPZ: 25 to 50 mg/d<br>QUE: 25 to 200 mg/d	Schizophrenia (DSM – IV)	*'[…] at least two 6-week periods of treatment with different antipsychotic agents (at doses equivalent to or greater than 700 mg/d of chlorpromazine) in the preceding 5 years with no evidence of significant improvement between the 2 treatment periods, and with persistence of at least 1 positive symptoms and 1 or more major areas of functioning (e.g., work, interpersonal relations) markedly below the level achieved prior to this period; a total score of at least 60 on the PANSS; a score of at least 4 (moderately ill) on the CGI severity of illness item.'*	**Sex:**<br>55.90% males<br>**Mean age (years):**<br>39<br>**Mean duration of illness (years):**<br>n.i.

Study ID	Study arms; n of participants	Study design	Study duration (weeks)	Country	Mean or range drug doses (mg/d)	Diagnosis	Definition of TRS	Population characteristics
Azorin 2001 [24]	CLOZ, n = 72<br>RISP, n = 75	parallel double-blind RCT	12	Canada<br>France	CLOZ: 200 to 900 mg/d<br>RISP: 2 to 15 mg/d	Schizophrenia (DSM – IV)	*'[…] 1. The patient's current episode had been treated continually with a neuroleptic for at least the preceding 6 months without significant clinical improvement. 2. The patient had undergone one unsuccessful trial of antipsychotic medication equivalent to 20 mg/day of haloperidol for at least six weeks (less if the patient was experiencing dose-limiting ad verse events) since the onset of the current episode. If several drugs had been prescribed simultaneously, the final equivalence dosage could be calculated by adding the individual equivalencies. 3. The patient had experienced no period of good functioning for at least 24 months despite a sufficient period of use of two antipsychotics from at least two chemical classes, or no period of good functioning for five years despite the use of three antipsychotics. Poor previous treatment response as defined in the current study differs from treatment resistance by having a less stringent criterion for the previous drug history than did the study by Kane et al., enabling patients to be more representative of those seen in current clinical practice compared with previous clinical trials.'*	**Sex:**<br>71.10% males<br>**Mean age (years):**<br>38.84<br>**Mean duration of illness (years):**<br>15.65
Bitter 2004 [25]	CLOZ, n = 72<br>OLA, n = 75	parallel double-blind RCT	18	Hungary<br>South Africa	CLOZ: 216.20 mg/d<br>OLA: 17.20 mg/d	Schizophrenia (DSM – IV)	*'[…] patients had to have failed to respond adequately to standard acceptable treatment with a conventional antipsychotic medication (at least one treatment trial of 4-6 weeks duration at 400-600 mg equivalents of CPZ; Barnes and McEvedy, 1996) caused by the medication'*	**Sex:**<br>59.90% males<br>**Mean age (years):**<br>37.60<br>**Mean duration of illness (years):**<br>n.i.
Bondolfi 1998 [26]	CLOZ, n = 43<br>RISP, n = 43	parallel double-blind RCT	8	France<br>Switzerland	CLOZ: 291.20 mg/d<br>RISP: 6.4 mg/d	Schizophrenia (DSM -III-R)	*'They had previously failed to respond to or were intolerant of at least 2 different classes of antipsychotic drugs given in appropriated doses for at least 4 weeks; defined retrospectively from the patients' files.'*	**Sex:**<br>71% males<br>**Mean age (years):**<br>37.30<br>**Mean duration of illness (years):**<br>11.80
Bozzatello 2019 [27]	PALIP, n = 36<br>PALIP ER, n = 36	parallel open-label RCT	24	Canada<br>Italy	PALIP: 50 to 150 mg/d<br>PALIP ER: 6 to 12 mg/d	Schizophrenia (DSM – V)	*'[…] had a diagnosis stable but symptomatic schizophrenia and were previously unsuccessfully treated with an oral antipsychotic at an adequate therapeutic dose and with a change in Clinical Global Impression–Severity (CGI-S) score of ≤ 1 in the 4 weeks before enrollment.'*	**Sex:**<br>43.10% males<br>**Mean age (years):**<br>46.40<br>**Mean duration of illness (years):**<br>n.i.

Study ID	Study arms; n of participants	Study design	Study duration (weeks)	Country	Mean or range drug doses (mg/d)	Diagnosis	Definition of TRS	Population characteristics
Breier 1999a [28]	CLOZ, n = 14<br>RISP, n = 15	parallel double-blind RCT	6	USA	CLOZ: 403.60 mg/d<br>RISP: 5.90 mg/d	Chronic Schizophrenia (DSM – IV)	*'[…] partial response to neuroleptics: 1) a history of residual positive and/or negative symptoms after at least a 6-week trial of a therapeutic dose of a neuroleptic agent, 2) at least a minimum level of positive and/or negative symptoms at the time of evaluation for the study, and 3) at least a minimum level of positive and/or negative symptoms after a prospective trial of at least 2 weeks of fluphenazine, 20 mg/day (with dose adjustments between 10 mg/day and 30 mg/day allowed in or der to optimize outcome). The minimum positive symptom level was a total score of at least 8 for the four BPRS positive symptom items (conceptual disorganization, hallucinations, unusual thought content, and suspiciousness). The minimum negative symptom level was a total score on the SANS of at least 20.'*	**Sex:**<br>65.50% males<br>**Mean age (years):**<br>34.96<br>**Mean duration of illness (years):**<br>12.45
Breier 1999b [29]	HAL, n = 174<br>OLA, n = 352	parallel double-blind RCT	6	USA	HAL: 10.0 mg/d<br>OLA: 11.10 mg/d	Schizophrenia or Schizophreniform or Schizoaffective Disorder (DSM-III-R)	*'[…] failure to respond to at least one neuroleptic over a period of at least 8 weeks during the previous 2 years; 2) BPRS total score of at least 24 (18-item version, each item scored 0 to 6); and 3) BPRS positive score of at least 8 or scores of at least 4 on any of the following BPRS items: conceptual disorganization, suspiciousness, hallucinatory behavior, and unusual thought content. BPRS scores from the first visit while patients were still being treated with their prior antipsychotic drug were used in the classification.'*	**Sex:**<br>63.90% males<br>**Mean age (years):**<br>38.27<br>**Mean duration of illness (years):**<br>15.33
Browne 1988 [30]	HAL, n = 6<br>PBO, n = 5	parallel double-blind RCT	20	Ireland	HAL: 10 to 40 mg/d<br>PBO: NA	Schizophrenia (Feighner's criteria, 1972) [31]	*'[…] chronic schizophrenic in-patients who had persistent positive symptoms, despite having previously had adequate trial of conventional doses of neuroleptic medication […]'*	**Sex:**<br>45.45% males<br>**Mean age (years):**<br>40.88<br>**Mean duration of illness (years):**<br>17.49
Buchanan 1998 [32]	CLOZ, n = 38<br>HAL, n = 37	parallel double-blind RCT	10	USA	CLOZ: 463.80 mg/d<br>HAL: 25.20 mg/d	Schizophrenia or Schizoaffective Disorder (DSM-III-R)	*'Patients were required to meet retrospective and prospective criteria for partial response to conventional neuroleptics. The retrospective criteria were 1) a history of residual positive and/or negative symptoms after at least two 6-week trials of therapeutic dosages of conventional neuroleptics from at least two different classes and 2) a minimum level of positive and/or negative symptoms at the time of evaluation for participation in the study. The minimum positive symptom level was a total score of at least 8 on the BPRS items for conceptual disorganization, hallucinations, unusual thought content, and suspiciousness (item scores on the BPRS range from 1 to 7) or a score of at least 4 on any one of the items'*	**Sex:**<br>61% males<br>**Mean age (years):**<br>40.56<br>**Mean duration of illness (years):**<br>19.91

Study ID	Study arms; n of participants	Study design	Study duration (weeks)	Country	Mean or range drug doses (mg/d)	Diagnosis	Definition of TRS	Population characteristics
Buchanan 2005 [33]	HAL, n = 34<br>OLA, n = 29	parallel double-blind RCT	16	USA	HAL: 18.30 mg/d<br>OLA: 20.30 mg/d	Schizophrenia or Schizoaffective Disorder (DSM – IV)	*'Patients were required to meet retrospective and prospective criteria for partial response to conventional antipsychotics. Retrospective criteria were 1) a history of residual positive and/or negative symptoms after at least two 6-week trials of therapeutic doses of conventional antipsychotics from at least two different classes; and 2) a minimum level of positive and/or negative symptoms at the time of evaluation for participation in the study. The minimum positive symptom level was a total score of 8 or more on the four BPRS positive symptom items or a score of 4 or more on any one of the items.'*	**Sex:**<br>73% males<br>**Mean age (years):**<br>44.33<br>**Mean duration of illness (years):**<br>21.20
Chen 2012 [34]	OLA, n = 16<br>RISP, n = 16	parallel single-blind RCT	8	Tawain	OLA: 4.30 mg/d<br>RISP: 17.80 mg/d	Schizophrenia (DSM – IV)	*'[…] 'inadequate response' as PANSS total score ≧60, at least 1 positive item score ≧4, and CGI-S score ≧4 after 6 weeks of FGA treatment with chlorpromazine equivalent (CPE) ≧500 mg/day.'*	**Sex:**<br>29.88% males<br>**Mean age (years):**<br>37.30<br>**Mean duration of illness (years):**<br>10.45
Chowdhury 1999 [35]	CLOZ, n = 30<br>RISP, n = 30	parallel RCT	16	India	CLOZ: 342.86 mg/d<br>RISP: 5.80 mg/d	Schizophrenia and it subtypes (ICD-10)	*'[…] schizophrenia for more than 6 months and received at least one full course (i.e., at least 6 weeks) treatment with conventional neuroleptics agents (either chlorpromazine 600-800 mg/day, haloperidol or trifluoperazine in doses equivalent to 600-800 mg/day of chlorpromazine) without adequate response.'*	**Sex:**<br>75% males<br>**Mean age (years):**<br>31.37<br>**Mean duration of illness (years):**<br>12.46
Claus 1992 [36]	HAL, n = 21<br>RISP, n = 21	parallel double-blind RCT	12	Belgium	HAL: 10.30 mg/d<br>RISP: 12 mg/d	Schizophrenia (DSM-III-R)	*'Despite optimization of conventional neuroleptic treatment, they still presented positive, negative and/or extrapyramidal symptoms'*	**Sex:**<br>66.60% males<br>**Mean age (years):**<br>37.70<br>**Mean duration of illness (years):**<br>n.i.

Study ID	Study arms; n of participants	Study design	Study duration (weeks)	Country	Mean or range drug doses (mg/d)	Diagnosis	Definition of TRS	Population characteristics
Conley 1998 [37]	CPZ, n = 42<br>OLA, n = 42	parallel double-blind RCT	8	USA	CPZ: 1173 mg/d<br>OLA: 25 mg/d	Schizophrenia (DSM-III-R)	*'[…] all subjects met criteria for treatment resistance. These criteria were as follows: 1) at least two periods of treatment in the preceding 5 years with an antipsychotic drug (from at least two different chemical classes, excluding haloperidol), at dosages ≥1000mg/d of chlorpromazine equivalents, for 6 weeks without significant symptomatic relief; 2) no period of good functioning within the past 5 years; and 3) severity of psychopathology indicated by a total score of 45 or more on the BPRS (items rated 1–7), a CGI severity score of 4 or more, and a score of 4 or more on at least two of the BPRS psychosis items. These criteria differ from the criteria in the Kane et al. study (9) in that those investigators used three retrospective treatment failures, which could include haloperidol. Subjects in the present study could have had prior clozapine treatment but could not have demonstrated resistance to clozapine.'*	**Sex:**<br>73.80% males<br>**Mean age (years):**<br>42.77<br>**Mean duration of illness (years):**<br>21.31
Conley 2003 [38]	CLOZ, n = 5<br>OLA, n = 8	crossover double-blind RCT	12	USA	CLOZ: 50 to 450 mg/d<br>OLA: 30 to 50 mg/d	Schizophrenia (DSM – IV)	*'[…] treatment-resistant schizophrenia […] Response to olanzapine or chlorpromazine was defined a priori as a 20% improvement in total BPRS score and a final BPRS score of ,35 or a 1 point improvement on the CGI-S.'*	**Sex:**<br>61.50% males<br>**Mean age (years):**<br>37.58<br>**Mean duration of illness (years):**<br>n.i.
Conley 2005 [39]	QUE, n = 12<br>RISP, n= 13<br>FLUPH, n = 13	parallel double-blind RCT	12	USA	QUE: 463.60 mg/d<br>RISP: 4.31 mg/d<br>FLUPH: 13.20 mg/d	Schizophrenia (DSM – IV)	*'[…]met the following criteria for treatment resistance: (1) persistent positive psychotic symptoms at study entry ('moderate' severity [≥4 points on a 1–7 point scale] on 2 of 4 psychosis items on the BPRS); (2) persistent global illness severity (BPRS total score ≥45 points on the 18-item scale and a CGI score of ≥4 points [moderately ill]); (3) 2 prior failed treatment trials with 2 different antipsychotics at doses of at least 600mg/d chlorpromazine equivalents, each of at least 6 weeks duration; and (4) no stable period of good social/occupational functioning within the previous 5 years'*	**Sex:**<br>79% males<br>**Mean age (years):**<br>45.10<br>**Mean duration of illness (years):**<br>n.i.
Daniel 1996 [40]	CLOZ, n = 10<br>RISP, n = 10	parallel single-blind RCT	6	USA	CLOZ: 375 mg/d<br>RISP: 6.10 mg/d	Schizophrenia or Schizoaffective Disorder (DSM – IV)	*'All patients were clinically stable on clozapine regimens at the time of screening) I9 were outpatients, and one was an inpatient), and each had experienced multiple past treatment failures or intolerance of conventional antipsychotic side effects.'*	**Sex:**<br>35% males<br>**Mean age (years):**<br>33.80<br>**Mean duration of illness (years):**<br>22.70

Study ID	Study arms; n of participants	Study design	Study duration (weeks)	Country	Mean or range drug doses (mg/d)	Diagnosis	Definition of TRS	Population characteristics
Dean 1958 [41]	CPZ, n = 9<br>PBO, n = 9	parallel double-blind RCT	15	USA	CPZ: 2000 to 3000 mg/d<br>PBO: NA	Schizophrenia (clinical diagnosis)	*'[…] they had failed to respond satisfactorily to past treatment, because of an absence of handicapping physical defects, and because of either a work record outside the hospital or an interested family, or both.'*	**Sex:**<br>100% females<br><br>**Mean age (years):**<br>37<br><br>**Mean duration of illness (years):**<br>n.i.
Emsley 2000 [42]	QUE, n = 143<br>HAL, n = 145	parallel double-blind RCT	8	South Africa<br>UK	QUE: 600 mg/d<br>HAL: 20 mg/d	Schizophrenia (DSM – IV)	*'[…] history of unsuccessful therapy for schizophrenia and had shown either a partial response or no response to 1 month of treatment with the conventional antipsychotic.'*	**Sex:**<br>70.50% males<br><br>**Mean age (years):**<br>38.25<br><br>**Mean duration of illness (years):**<br>n.i.
Geller 2005 [43]	CLOT, n = 21<br>CPZ, n = 20	cross-over double-blind RCT	12	Israel	CLOT: 40 mg/d<br>CPZ: 100 mg/d	Schizophrenia (n.i.)	*'[…] schizophrenia who were severe chronic active psychotic hospitalized patients with a history of nonresponse to at least 3 neuroleptics. 'Severe chronic active psychosis' was defined as symptoms that required inpatient care or closed hostel care for at least 50% of the patient's life over the past 5 years, despite adequate compliance with both pharmacologic and psychosocial therapies.'*	**Sex:**<br>75.90% males<br><br>**Mean age (years):**<br>44.20<br><br>**Mean duration of illness (years):**<br>19.90
Hall 1968 [44]	FLUPH, n = 25<br>HAL, n = 25	parallel double-blind RCT	12	USA	FLUPH: 17.86 mg/d<br>HAL: 6.95 mg/d	Schizophrenia (clinical diagnosis)	*'[…] who had been largely resistant to a number of chemotherapy regimes […]'*	**Sex:**<br>100% males<br><br>**Mean age (years):**<br>45<br><br>**Mean duration of illness (years):**<br>n.i.
Herken 1999 [45]	CLOZ, n= 17<br>SUL, n= 19	parallel RCT	9	Turkey	CLOZ: 521 mg/d<br>SUL: 905 mg/d	Schizophrenia (DSM – IV)	*'[…] were diagnosed with chronic schizophrenia according to DSM-IV diagnostic criteria, who were resistant to classical antipsychotic therapy […]'*	**Sex:**<br>67.10% males<br><br>**Mean age (years):**<br>36.42<br><br>**Mean duration of illness (years):**<br>7.32

Study ID	Study arms; n of participants	Study design	Study duration (weeks)	Country	Mean or range drug doses (mg/d)	Diagnosis	Definition of TRS	Population characteristics
Heres 2022 [46]	AMI, n = 71<br>OLA, n = 71	parallel double-blind RCT	6	Germany	AMI: 649 mg/d<br>OLA: 15.90 mg/d	Schizophrenia or Schizophreniform or Schizoaffective Disorder (DSM – IV)	*'[…] nonresponsive to 2 weeks to AMI or OLA […]'*	**Sex:**<br>51.40% males<br><br>**Mean age (years):**<br>39.60<br><br>**Mean duration of illness (years):**<br>n.i.
Hong 1997 [47]	CLOZ, n = 21<br>CPZ, n = 19	parallel double-blind RCT	12	China	CLO: 543 mg/d<br>CPZ: 1163 mg/d	Schizophrenia (DSM – IV)	*'Treatment-refractory was defined as persistent severe psychotic symptoms for at least 6 months while the patient received adequate neuroleptic treatment (from at least two different chemical classes ant at dosages equivalent ≥ 1000mg/d CPZ).'*	**Sex:**<br>35% males<br><br>**Mean age (years):**<br>38.70<br><br>**Mean duration of illness (years):**<br>n.i.
Honigfeld 1984 [48]	CLOZ, n = 75<br>CPZ, n = 76	parallel double-blind RCT	4	USA	CLO: 417 mg/d<br>CPZ: 795 mg/d	Schizophrenia (DSM – II)	*'[…] considered treatment resistant as evidenced by either poor response to previous therapy (an average prior psychotic drug use of 3-4 different drugs per patient, ranging up to 7-9), or a documented history of sensitivity to extrapyramidal side effects with at t least two prior antipsychotic drugs or a history of tardive dyskinesia.'*	**Sex:**<br>60.90% males<br><br>**Mean age (years):**<br>30<br><br>**Mean duration of illness (years):**<br>n.i.
Howard 1974 [49]	HAL, n = 17<br>THIO, n = 16<br>PBO, n = 13	parallel double-blind RCT	12	USA	HAL: n.i.<br>THIO: n.i.<br>PBO: NA	Chronic psychosis (clinical diagnosis)	*'[…] treatment resistant, and hopeless chronic psychotics […] 'all-or-non' response to the study medications; i.e. the patient was no longer psychotic and no longer re quired custodial care […]'*	**Sex:**<br>100% females<br><br>**Mean age (years):**<br>45.95<br><br>**Mean duration of illness (years):**<br>20

Study ID	Study arms; n of participants	Study design	Study duration (weeks)	Country	Mean or range drug doses (mg/d)	Diagnosis	Definition of TRS	Population characteristics
Kahn 2018 [50]	AMI, n = 47<br>OLA, n = 47	parallel double-blind RCT	4	European countries Israel	AMI: 590.90 mg/d<br>OLA: 15.60 mg/d	Schizophrenia or Schizophreniform or Schizoaffective Disorder (DSM – IV)	*'[…] patients with (first-episode) schizophrenia who do not respond to their initial antipsychotic treatment (response has been defined differently in the various studies; we used remission in the current study.'*	**Sex:** 53.76% males<br>**Mean age (years):** 24.75<br>**Mean duration of illness (years):** n.i.
Kane 1988 [51]	CLOZ, n = 126<br>CPZ, n = 142	parallel double-blind RCT	6	USA	CLOZ: 600 to 900 mg/d<br>CPZ: 1000 to 1800 mg/d	Schizophrenia (DSM – III)	*'The criteria for being classified as refractory to treatment included the following: (1) at least three periods of treatment in the preceding five years with neuroleptic agents (from at least two different chemical classes) at dosages equivalent to or greater than 1000 mg/d of chlorpromazine for a period of six weeks, each without significant symptomatic relief, and (2) no period of good functioning within the preceding five years.'*	**Sex:** 80% males<br>**Mean age (years):** 35.90<br>**Mean duration of illness (years):** 15.30
Kane 2001 [52]	CLOZ, n = 37<br>HAL, n = 34	parallel double-blind RCT	29	USA	CLOZ: 523 mg/d<br>HAL: 18.90 mg/d	Schizophrenia or Schizoaffective Disorder (DSM-III-R)	*'Poor or poor response was defined by documented treatment failure in 2 trials of conventional antipsychotics at dosages equivalent to or greater than chlorpromazine hydrochloride, 600 mg/d for at least 6 weeks (high-dose qualification). Patients for whom a low-dose trial could not be documented received prospective dose reduction for 4 weeks or less if clinical worsening was seen. Only patients who met symptom criteria for inclusion after such treatments (a rating of at least moderate on 1 of the following 4 BPRS items […]'*	**Sex:** 70.50% males<br>**Mean age (years):** 40.52<br>**Mean duration of illness (years):** 17
Kane 2006 [53]	CPZ, n = 154<br>ZIP, n = 152	parallel double-blind RCT	12	USA	CPZ: 743.60 mg/d<br>ZIP: 153.80 mg/d	Schizophrenia (DSM-III-R)	*'[…] treatment resistant if they had had three or more 6-week periods of treatment in the preceding 5 years with at least 2 different antipsychotic agents without experiencing significant symptomatic improvement or a period of good functioning. Additional entry criteria were a total score ≥45 on the BPRS; a score ≥4 (moderate) on at least two PANSS core psychosis items; and a score ≥4 on the CGI-S scale. This definition of treatment resistance history is similar to the Kane criteria adapted by Conley et al. (1998) in a trial of olanzapine in subjects with treatment-resistant schizophrenia.'*	**Sex:** 73.53% males<br>**Mean age (years):** 34.95<br>**Mean duration of illness (years):** 11.50

Study ID	Study arms; n of participants	Study design	Study duration (weeks)	Country	Mean or range drug doses (mg/d)	Diagnosis	Definition of TRS	Population characteristics
Kane 2007 [54]	ARI, n = 154<br>PERP, n = 146	parallel double-blind RCT	6	Canada<br>USA	ARI: 28.80 mg/d<br>PERP: 39.10 mg/d	Schizophrenia (DSM – IV)	*'Treatment resistance was defined as failure to experience satisfactory symptom relief despite at least 2 periods of treatment (each lasting at least 6 weeks) with adequate doses of antipsychotics agents (one of which had to be a typical antipsychotic) during the 2 years prior to the study. In addition, patients should not have experienced satisfactory symptom relief with their most recent course of antipsychotic therapy. Patients also had to meet the following disease severity criteria: a PANSS total score ≥4 on least 2 the items of conceptual disorganisation, suspiciousness, hallucinatory behavior, or delusions and a CGI-S score ≥4.'*	**Sex:**<br>69% males<br>**Mean age (years):**<br>42.10<br>**Mean duration of illness (years):**<br>19.30
Kane 2011 [55]	RISP, n = 105<br>SERTIND, n = 216	parallel double-blind RCT	12	Canada<br>USA	RISP: 9 mg/d<br>SERTIND: 18.10 mg/d	Schizophrenia (DSM – IV)	*'[…] had failed 1 adequate antipsychotic treatment in the previous 6 months.'*	**Sex:**<br>77.57% males<br>**Mean age (years):**<br>38.80<br>**Mean duration of illness (years):**<br>16.70
Kinon 1993 [56]	FLUPH, n = 34<br>HAL, n = 13	parallel double-blind RCT	4	USA	FLUPH: 48,24 mg/d<br>HAL: 20 mg/d	Schizophrenia or Schizophreniform or Schizoaffective Disorder (DSM-III-R)	*'treatment-resistant […] non responsive to 1 prospective trial […]'*	**Sex:**<br>n.i.<br>**Mean age (years):**<br>29.40<br>**Mean duration of illness (years):**<br>n.i.
Kinon 2010 [57]	OLA, n = 186<br>RISP, n = 192	parallel double-blind RCT	12	USA	OLA: 10 to 20 mg/d<br>RISP: 2 to 6 mg/d	Schizophrenia or Schizophreniform or Schizoaffective Disorder (DSM – IV)	*'treatment-resistant […] non responsive to 1 prospective trial […]'*	**Sex:**<br>72.40% males<br>**Mean age (years):**<br>41.90<br>**Mean duration of illness (years):**<br>18

Study ID	Study arms; n of participants	Study design	Study duration (weeks)	Country	Mean or range drug doses (mg/d)	Diagnosis	Definition of TRS	Population characteristics
Kluge 2007 [58]	CLOZ, n = 15<br>OLA, n = 15	parallel double-blind RCT	6	Germany	CLOZ: 266.70 mg/d<br>OLA: 21.20 mg/d	Schizophrenia or Schizophreniform or Schizoaffective Disorder (DSM – IV)	*'[…] clozapine non responsive / intolerant [...]'*	**Sex:** 40% males<br>**Mean age (years):** 34.75<br>**Mean duration of illness (years):** n.i.
Kumar 2017 [59]	CLOZ, n= 24<br>QUET, n= 29	parallel open-label RCT	14	India	CLOZ: 322.50 mg/d<br>QUET: 790 mg/d	Schizophrenia (ICD – 10)	*'[…] No clinical response with 2 different antipsychotics used separately in the dose range of 400–600mg of CPZ per day or equivalents for 6 weeks; no period of good social or occupational functioning at least in last 1 year, and a minimum CGIs scale rating of 4 (moderately ill); BPRS total score >45, and a score of >4 on 2 out of 4 positive items.'*	**Sex:** 67.50% males<br>**Mean age (years):** 39.40<br>**Mean duration of illness (years):** n.i.
Kumra 1996 [60]	CLOZ, n = 10<br>HAL, n = 11	parallel double-blind RCT	6	USA	CLOZ: 176 mg/d<br>HAL: 16 mg/d	Schizophrenia (DSM – III)	*'[…] non responsive to at least two typical neuroleptics [...]'*	**Sex:** 52.38% males<br>**Mean age (years):** 13.73<br>**Mean duration of illness (years):** n.i.
Kumra 2008 [61]	CLOZ, n = 18<br>OLA, n = 21	parallel double-blind RCT	12	USA	CLOZ: 403.10 mg/d<br>OLA: 26.20 mg/d	Schizophrenia Or Schizoaffective Disorder (K-SADS-PL)	*'[…] failure of two prior antipsychotic treatments.'*	**Sex:** n.i.<br>**Mean age (years):** 15.65<br>**Mean duration of illness (years):** n.i.

Study ID	Study arms; n of participants	Study design	Study duration (weeks)	Country	Mean or range drug doses (mg/d)	Diagnosis	Definition of TRS	Population characteristics
Lal 2006 [62]	CPZ, n = 19<br>LEV, n = 19	parallel double-blind RCT	30	Canada	CPZ: 813 mg/d<br>LEV: 762 mg/d	Schizophrenia (DSM-III-R)	*'Criteria for TRS were based on those of Kane et al and in the present trial consisted of (a) historical criteria: (i) at least 3 periods of treatment with neuroleptics from at least 2 different chemical classes for a period of at least 6 weeks at a dose of at least 1000 mg CPZ equivalents/d, (ii) no good period of functioning in the past 5 years; of (b) severity criteria: (i) total BPRS score of at least 45 (18-item version rated 1–7; 1 = absence, 7 = severe) during screening and on each occasion when assessed every 2 weeks during the 6-week baseline phase, (ii) a CGI-S rating of at least 4 or more (4 = moderately ill) during screening and on each assessment during the baseline phase of the study, (iii) item score of at least 4 (4 = moderate) on 2 of the following BPRS items: conceptual disorganization, suspiciousness, hallucinatory behaviour and unusual thought content (4-item psychosis cluster)4 during screening and on each assessment during the baseline phase; and of (c) prospective criteria: failure to show clinical improvement in an open prospective trial with HAL (up to 60 mg/d in liquid form) plus BT (up to 6 mg/d) to confirm TRS.'*	**Sex:**<br>63,16% males<br><br>**Mean age (years):**<br>38.95<br><br>**Mean duration of illness (years):**<br>19
Lin 2013 [63]	CLOZ, n = 24<br>ZOT, n = 35	parallel single-blind RCT	12	Tawain	CLOZ: 377.10 mg/d<br>ZOT: 397.10 mg/d	Schizophrenia (DSM – IV)	*'[…] had been maintained on clozapine for at least 6 months, and whose baseline Clinical Global Impression -Severity (CGI-S) score was at least 3, […]'*	**Sex:**<br>n.i.<br><br>**Mean age (years):**<br>n.i.<br><br>**Mean duration of illness (years):**<br>n.i.
Marjerrison 1964 [64]	TRIFLUO, n = 16<br>PBO, n = 34	parallel double-blind RCT	30	Canada<br>USA	TRIFLUO: 28 mg/d<br>PBO: NA	Chronic Psychosis (clinical diagnosis)	*'[…] highly treatment-resistive, long-term patients […]'*	**Sex:**<br>42% males<br><br>**Mean age (years):**<br>48<br><br>**Mean duration of illness (years):**<br>16.50

Study ID	Study arms; n of participants	Study design	Study duration (weeks)	Country	Mean or range drug doses (mg/d)	Diagnosis	Definition of TRS	Population characteristics
Mc Gurk 2005 [65]; Schooler 2016 [66]	CLOZ, n = 53<br>RISP, n = 54	parallel double-blind RCT	29	USA	CLOZ: 500 to 800 mg/d<br>RISP: 6 to 12 mg/d	Schizophrenia Or Schizoaffective Disorder (DSM – IV)	*'Partial or poor response was defined by failure in at least 1 trial first-generation antipsychotic medication at a dose equivalent to or greater than 600mg/d of chlorpromazine for 6 weeks (high-dose qualification) and failure at a dose equivalent to 250-500 mg/d for 4 weeks (low-dose qualification.'*	**Sex:** 78.50% males<br>**Mean age (years):** 42<br>**Mean duration of illness (years):** 19.50
McCreadie 1977 [67]	CPZ, n = 10<br>HAL, n = 10	parallel double-blind RCT	12	UK	CPZ: 100 to 600 mg/d<br>HAL: 15 to 100 mg/d	Schizophrenia (clinical diagnosis)	*'[…] drug resistant chronic schizophrenics […] had had many previous courses of psychotropic medication (they had had on average 3 to 2 different major tranquillizers) […]'*	**Sex:** 100% males<br>**Mean age (years):** 52<br>**Mean duration of illness (years):** n.i.
McEvoy 2006 [13,68–70]	CLO, n = 49<br>OLA, n = 19<br>QUET, n = 15<br>RISP, n = 16	parallel double-blind *(CLO; QUE; RISP)* /open-blind *(OLA)* RCT	26	USA	CLO: 332.10 mg/d<br>OLA: 23.40 mg/d<br>QUET: 642.90 mg/d<br>RISP: 4.8 mg/d<br>*(modal doses)*	Schizophrenia (DSM – IV)	*'[…] did not respond to prior atypical antipsychotic treatment […]'*	**Sex:** 81% males<br>**Mean age (years):** 39.70<br>**Mean duration of illness (years):** n.i.
Meltzer 2008 [71]	CLOZ, n = 21<br>OLA, n = 19	parallel double-blind RCT	26	USA	CLOZ: 564 mg/d<br>OLA: 33.60 mg/d	Schizophrenia or Schizoaffective Disorder (DSM – IV)	*'[…] have a documented history of treatment resistant schizophrenia based on the criteria of Kande et al. […] despite 2 or more trials of typical or atypical antipsychotic drugs from different chemical classes, with usually adequate doses for at least 6 weeks.'*	**Sex:** 67.5% males<br>**Mean age (years):** 36.80<br>**Mean duration of illness (years):** 15.60

Study ID	Study arms; n of participants	Study design	Study duration (weeks)	Country	Mean or range drug doses (mg/d)	Diagnosis	Definition of TRS	Population characteristics
Mercer 1997 [72]	CPZ, n = 12<br>RISP, n = 15	parallel single-blind RCT	9	UK	CPZ: 500 mg/d<br>RISP: 8 mg/d	Schizophrenia (DSM-III-R)	*'[…] criteria for schizophrenia; treatment resistance meeting the definition of Level 4, 5 or 6 according to the scheme for treatment resistance of May et al. (1988); the duration of treatment resistance meeting criteria for at least Level 4 being at least 6 months and not more than 5 years'*	**Sex:**<br>81.48% males<br>**Mean age (years):**<br>40.18<br>**Mean duration of illness (years):**<br>n.i.
Meyer - Lindenberg 1997 [73]	CLOZ, n = 25<br>ZOT, n = 25	parallel double-blind RCT	6	Germany	CLOZ: 150 to 450 mg/d<br>ZOT: 150 to 450 mg/d	Schizophrenia (DSM-III-R)	*'[…] clozapine, patients were only included if they had been treated before for at least three weeks each with two conventional neuroleptics in effective doses without a satisfactory result or with intolerable side-effects.*	**Sex:**<br>69.23% males<br>**Mean age (years):**<br>33.15<br>**Mean duration of illness (years):**<br>n.i.
Moresco 2004 [74]	CLOZ, n = 12<br>OLA, n = 11	parallel double-blind RCT	8	Italy	CLOZ: 325.40 mg/d<br>OLA: 18.30 mg/d	Schizophrenia (DSM – IV)	*'[…] lack of clinical response to two previous neuroleptic treatments with two different classes of antipsychotics, with a duration of at least 6 weeks each, given at an appropriate dosage (at least 500 mg chlorpromazine equivalent).'*	**Sex:**<br>73.33% males<br>**Mean age (years):**<br>35.80<br>**Mean duration of illness (years):**<br>n.i.
Naber 2005 [75(p200)]	CLOZ, n = 57<br>OLA, n = 57	parallel double-blind RCT	26	Germany	CLOZ: 209 mg/d<br>OLA: 16.20 mg/d	Schizophrenia (DSM – IV)	*'All participants had a documented history that they had either failed to respond to at least one antipsychotic other than clozapine and olanzapine or had experienced intolerable side effects during there prior antipsychotic treatments.'*	**Sex:**<br>61% males<br>**Mean age (years):**<br>34<br>**Mean duration of illness (years):**<br>7.10
Rosenheck 1997 [76]	CLOZ, n = 205<br>HAL, n = 218	parallel double-blind RCT	52	USA	CLOZ: 552 mg/d<br>HAL: 28 mg/d<br>*(average daily dose)*	Schizophrenia (DSM-III-R)	*'[…] refractoriness, defined as persisting psychotic symptoms despite adequate treatment trials of two or more antipsychotic drugs at 1000-mg chlorpromazine equivalents; severe symptoms; indicated by scores on the BPRS and CGI; and serious social dysfunction for the previous two years.'*	**Sex:**<br>97.64% males<br>**Mean age (years):**<br>43.56<br>**Mean duration of illness (years):**<br>21.25

Study ID	Study arms; n of participants	Study design	Study duration (weeks)	Country	Mean or range drug doses (mg/d)	Diagnosis	Definition of TRS	Population characteristics
Rosenheck 1997 [76]	CLOZ, n = 205<br>HAL, n = 218	parallel double-blind RCT	52	USA	CLOZ: 552 mg/d<br>HAL: 28 mg/d<br>*(average daily dose)*	Schizophrenia (DSM-III-R)	*'[…] refractoriness, defined as persisting psychotic symptoms despite adequate treatment trials of two or more antipsychotic drugs at 1000-mg chlorpromazine equivalents; severe symptoms; indicated by scores on the BPRS and CGI; and serious social dysfunction for the previous two years.'*	**Sex:**<br>97.64% males<br><br>**Mean age (years):**<br>43.56<br><br>**Mean duration of illness (years):**<br>21.25
Sacchetti 2009 [77]	CLOZ, n = 74<br>ZIP, n = 73	parallel double-blind RCT	18	Italy	CLOZ: 130 mg/d<br>ZIP: 346 mg/d	Schizophrenia (DSM – IV)	*'[…] resistant and/ or intolerant to at least 3 acute cycles with different antipsychotic treatments in the previous 5 years.'*	**Sex:**<br>69.20% males<br><br>**Mean age (years):**<br>39.95<br><br>**Mean duration of illness (years):**<br>13.75
Schiele 1961, 06602 [78]	CPZ, n = 20<br>THIO, n = 20<br>TRIFLUO, n = 20<br>PBO, n = 20	parallel double-blind RCT	16	USA	CPZ: 894 mg/d<br>THIO: 958 mg/d<br>TRIFLUO: 35 mg/d<br>PBO: NA	Schizophrenia (clinical diagnosis)	*'[…] previous treatment of this group of patients had been extensive. Virtually all of them had had […] insulin treatment. From these and other treatments they had obtained only limited or temporary benefit […] all previously been on ataractic medications, from which many of them had attained and maintained moderate degrees of improvement. For the 11 months immediately preceding the study, the patients had been on the following medications: chlorpromazine […], mepazine […], trifluoperazine […], prochlorperazine […]*	**Sex:**<br>100% males<br><br>**Mean age (years):**<br>40.60<br><br>**Mean duration of illness (years):**<br>n.i.
Schlosberg 1978 [79]	PIPOTLA, n = 30<br>FLU, n = 30<br>PBO, n = 15	parallel double-blind RCT	36	Israel	PIPOTLA: 27.50 mg/d<br>FLU: 27.50 mg/d<br>PBO: NA	Schizophrenia (clinical diagnosis)	*'[…] chronic schizophrenic patients highly resistant to treatment […]'*	**Sex:**<br>n.i.<br><br>**Mean age (years):**<br>41.65<br><br>**Mean duration of illness (years):**<br>17.12

Study ID	Study arms; n of participants	Study design	Study duration (weeks)	Country	Mean or range drug doses (mg/d)	Diagnosis	Definition of TRS	Population characteristics
See 1999 [80]	HAL, n = 10<br>RISP, n = 10	parallel double-blind RCT	5	Kuwait	HAL: 15 to 30 mg/d<br>RISP: 4 to 6 mg/d	Schizophrenia (DSM – IV)	*'[…] defined as being partial responders to previous antipsychotic drug treatment based on their history and current status as determined by the participating psychiatrists at the time of the study.'*	**Sex:**<br>70% males<br><br>**Mean age (years):**<br>35.60<br><br>**Mean duration of illness (years):**<br>10.65
Shalev 1993 [81]	HAL, n = 18<br>LEV, n = 21<br>PERP, n = 21	parallel double-blind RCT	4	USA	HAL: 20 mg/d<br>LEV: 300 mg/d<br>PERP: 32 mg/d	Schizophrenia (DSM – III)	*'[…] chronic patients with previous failure to treatment […]'*	**Sex:**<br>41.60% males<br><br>**Mean age (years):**<br>33<br><br>**Mean duration of illness (years):**<br>n.i.
Shaw 2006 [82]	CLOZ, n = 12<br>OLA, n = 13	parallel double-blind RCT	8	USA	CLOZ: 327 mg/d<br>OLA: 18.10 mg/d	Schizophrenia (DSM – IV; unmodified)	*'[…] failure to respond to 2 antipsychotic medications (typical or atypical) used at adequate doses 100-mg chlorpromazine equivalents and for adequate duration (4 weeks unless terminate owing to intolerable adverse effects). Failure was defined as in sufficient response with persistence of symptoms significantly impairing the child's functioning according to child, parental, medical, and school reports or intolerable adverse effects.'*	**Sex:**<br>60% males<br><br>**Mean age (years):**<br>12.27<br><br>**Mean duration of illness (years):**<br>3.20
Sirota 2006 [83]	QUE, n = 19<br>OLA, n = 21	parallel single-blind RCT	12	Israel	QUE: 16 mg/d OLA: 637.20 mg/d	Schizophrenia (DSM – IV)	*'[…] had not adequately responded to previous medications, defined as a lack of response to at least two conventional antipsychotics (e.g. perphenazine and haloperidol) at a dose of 400–600 mg chlorpromazine equivalent, for a period of 4 to 6 weeks.'*	**Sex:**<br>80% males<br><br>**Mean age (years):**<br>37.20<br><br>**Mean duration of illness (years):**<br>14.54

Study ID	Study arms; n of participants	Study design	Study duration (weeks)	Country	Mean or range drug doses (mg/d)	Diagnosis	Definition of TRS	Population characteristics
Smith 2001 [84]	HAL, n = 18<br>OLA, n = 20	parallel double-blind RCT	8	USA	HAL: 37.90 mg/d<br>OLA: 19.90 mg/d	Schizophrenia or Schizoaffective psychosis (n.i.)	*'Refractoriness was defined as a poor clinical response to at least two typical neuroleptics, with current active positive and/or severe negative symptoms which impacted on functioning and prevented discharge; patients had to be continuously hospitalized for at least 1 yr.'*	**Sex:**<br>91.20% males<br><br>**Mean age (years):**<br>43<br><br>**Mean duration of illness (years):**<br>25.40
Suzuki 2007 [85]	OLA, n = 15<br>QUE, n = 10<br>RISP, n = 12	parallel open-label RCT	4	Japan	OLA: 18,3 mg/d<br>QUE: 564 mg/d<br>RISP: 5.47 mg/d	Schizophrenia (DSM – IV)	*'[…] non-responsive to one prospective trial […]'*	**Sex:**<br>44.87% males<br><br>**Mean age (years):**<br>44.90<br><br>**Mean duration of illness (years):**<br>17
Tollefson 2001 [86]	CLOZ, n = 90<br>OLA, n = 90	parallel double-blind RCT	18	European countries South Africa	CLOZ: 303.60 mg/d<br>OLA: 20.50 mg/d	Schizophrenia (DSM – IV)	*'All study participants were required to have a documented history that they were clinically resistant to previous antipsychotic treatments. Resistance was defined as a lack of satisfactory clinical response to at least 2 previous oral neuroleptic treatments, each of a different chemical class, given for a duration of at least 6 weeks at an appropriate daily dosage equivalent to at least 500 mg chlorpromazine, or to the maximum daily dosage when intolerable side effects had been documented.'*	**Sex:**<br>63.80% males<br><br>**Mean age (years):**<br>38.60<br><br>**Mean duration of illness (years):**<br>15.80
Toru 1972 [87]	CPZ, n = 38<br>SUL, n = 38	parallel double-blind RCT	8	Japan	CPZ: 150 to 600 mg/d<br>SUL: 300 to 1200 mg/d	Schizophrenia (clinical diagnosis)	*'Patients who were thought not responsive to conventional psychotropic drugs were entered into the trial.'*	**Sex:**<br>77% males<br><br>**Mean age (years):**<br>35<br><br>**Mean duration of illness (years):**<br>n.i.

Study ID	Study arms; n of participants	Study design	Study duration (weeks)	Country	Mean or range drug doses (mg/d)	Diagnosis	Definition of TRS	Population characteristics
Volavka 2002 [88]	CLOZ, n = 40<br>HAL, n = 37<br>OLA, n = 39<br>RISP, n = 41	parallel double-blind RCT	14	USA	CLOZ: 526.60 mg/d<br>HAL: 25.70 mg/d<br>OLA: 30.40 mg/d<br>RISP: 11.60 mg/d	Schizophrenia or Schizoaffective Disorder (DSM – IV)	*'[…] suboptimal response to previous treatment, […] (1) suboptimal response was persistent positive symptoms […] after at least 6 contiguous weeks of treatment, presently or documented in the past, with one or more typical antipsychotics at doses ≥600 mg/ day in chlorpromazine equivalents […] (2) poor level of functioning over the past 2 years, defined by the lack of competitive employment or enrolment in an academic or vocational program and not having age-expected interpersonal relations with someone outside the biological family of origin with whom ongoing regular contacts were maintained.[…] patients required to had to have a baseline total score ≥60 on the Positive and Negative Syndrome Scale.'*	**Sex:**<br>84.70% males<br><br>**Mean age (years):**<br>40.80<br><br>**Mean duration of illness (years):**<br>19.50
Wahlbeck 2000 [89]	CLOZ, n = 11<br>RISP, n = 9	parallel rater-blind RCT	10	Finland	CLOZ: 385 mg/d<br>RISP: 7.80 mg/d	Schizophrenia (DSM – IV)	*'[…] persistent psychotic symptoms for at least 6 months, while the patient received antipsychotic treatment from at least 2 different chemical classes (1000 mg/d chlorpromazine equivalence) for a period at least 6 weeks each, […] non-response or intolerance to haloperidol.'*	**Sex:**<br>52.60% males<br><br>**Mean age (years):**<br>36.20<br><br>**Mean duration of illness (years):**<br>12.83
Wang 2022 [90]	OLA, n = 41<br>PALIP ER, n = 45	parallel double-blind RCT	12	China	OLA: 20.49 mg/d<br>PALI: 10.73 mg/d	Schizophrenia (DSM – IV)	*'Inclusion criterion of resistance was defined as the treatment failure due to an inadequate response after at least 2 conventional and standardized antipsychotic regimens (each treatment trial of a different chemical category, at least 6 weeks duration at 400 to 600 mg/d - equivalents of chlorpromazine), or intolerance to the standardized acceptable antipsychotic regimen because of adverse effects.'*	**Sex:**<br>77.90% males<br><br>**Mean age (years):**<br>33.26<br><br>**Mean duration of illness (years):**<br>10.19
Wirshing 1999 [91]	HAL, n = 33<br>RISP, n = 34	parallel single-blind RCT	8	USA	HAL: 19.40 mg/d<br>RISP: 7.50 mg/d	Schizophrenia (DSM-III-R)	*'All subjects met treatment-refractory criteria, which entailed a failure to respond to or an inability to tolerate at least three 6-week epochs of treatment within the preceding 5 years with antipsychotic medications from at least two different chemical classes, at daily doses equivalent to or greater than 1000 mg of chlorpromazine. These were the same criteria used by Kane et al. in their study that established the efficacy of clozapine in treatment-refractory individuals.'*	**Sex:**<br>82.09% males<br><br>**Mean age (years):**<br>40.50<br><br>**Mean duration of illness (years):**<br>19.10

Abbreviations:
AMI: amisulpride; **BPRS:** Brief Psychiatric Rating Scale; **CLOPENTHLA:** clopenthixol long-acting; **CLOT:** clotiapine; **CLOZ:** clozapine; **CPZ:** chlorpromazine; **DSM:** Diagnostic and Statistical Manual for Mental Disorders; **FLUPH:** fluphenazine; **CGI-S:** Clinical Global Impressions – Severity; **HAL:** haloperidol; **ICD:** International Classification of Diseases; **K-SADS-PL:** Kiddie-Schedule for Affective Disorders and Schizophrenia for School-Age Children–Present and Lifetime; **mg/d:** milligrams per day; **LEV:** levomepromazine; **n**: number; **NA:** not applicable; **N.I.:** no information; **OLA:** olanzapine; **PALIP:** paliperidone; **PALIP ER:** paliperidone extended release; **PANSS:** Positive and Negative Syndrome Scale; **PBO:** placebo; **PERP:** perphenazine**; PIPOTLA:** pipotiazine long-acting; **QUE:** quetiapine; **RCT:** randomised controlled trial; **RISP:** risperidone; **SANS:** Scale for the Assessment of Negative Symptoms; **SUL:** sulpiride; **THIO:** thioridazine; **TRIFLUO:** trifluoperazine; **TRS:** treatment-resistance; **ZIP:** ziprasidone

Appendix 6. Included studies: Add-on (Table S 6)

Study ID	Study arms; n of participants	Study design	Study duration (weeks)	Country	Mean or range drug doses (mg/d)	Diagnosis	Definition of TRS	Population characteristics
Afshar 2008 [92]	TOPIR, n = 16<br>PBO, n = 16<br><br>Add on **Clozapine**	parallel double-blind RCT	8	Iran	TOPIR: 25 to 300 mg/d<br>PBO: NA<br><br>CLOZ: n.i.	Schizophrenia (DSM-IV-TR)	*'Poor clinical outcome in spite of long-term treatment with several types of antipsychotic medications and under treatment with clozapine at a maximum tolerable dose for the previous 2 months.'*	**Sex:**<br>62.50% males<br><br>**Mean age (years):**<br>37.90<br><br>**Mean duration of illness (years):**<br>17.90
Aliyev 2010 [93]	LAM, n = 175<br>PBO, n = 175<br><br>Add on **Haloperidol Decanoate**	parallel single-blind RCT	12	Azerbaijan	LAM: 25 to 100 mg/d<br>PBO: NA<br><br>HAL: 7.14 mg/d	Schizophrenia (DSM – IV)	*'Lack of improvement after implementing at least one strategy targeted at "poor responders" to the initial antipsychotic treatment.'*	**Sex:**<br>62.29% males<br><br>**Mean age (years):**<br>32<br><br>**Mean duration of illness (years):**<br>7
Anil Yagcioglu 2005 [94]	RISP, n= 16<br>PBO, n= 14<br><br>Add on **Clozapine**	parallel double-blind RCT	6	Turkey	RISP: 2 to 6 mg/d<br>PBO: NA<br><br>CLOZ (RISP): 515.60 mg/d<br>CLOZ (PBO): 414.30 mg/d	Schizophrenia or Schizoaffective Disorder (DSM – IV)	*'[....] psychopathology was no more than partially responsive to clozapine treatment/had persistent positive symptoms, to at least 2 trials of adequate duration and dose of antipsychotic drugs other than clozapine.'*	**Sex:**<br>60% males<br><br>**Mean age (years):**<br>33.40<br><br>**Mean duration of illness (years):**<br>12.25
Assion 2008 [95]	AMI, n = 13<br>PBO, n = 3<br><br>Add on **Clozapine**	parallel double-blind RCT	6	Germany	AMI: 543.75 mg/d<br>PBO: NA<br><br>CLOZ: 300 mg/d	Chronic Schizophrenia (DSM – IV)	*'Patients who had already received clozapine for at least three months on a stable dose and were only partially or even nonrespondent, [...] at least moderately ill (CGI score ≥ 4).'*	**Sex:**<br>75% males<br><br>**Mean age (years):**<br>43.10<br><br>**Mean duration of illness (years):**<br>n.i.

Study ID	Study arms; n of participants	Study design	Study duration (weeks)	Country	Mean or range drug doses (mg/d)	Diagnosis	Definition of TRS	Population characteristics
Barnes 2017 [96]	AMI, n = 35<br>PBO, n = 33<br><br>Add on **Clozapine**	parallel double-blind RCT	12	UK	AMI: 400 to 800 mg/d<br>PBO: NA<br><br>CLOZ: n.i. mg/d	Schizophrenia (DSM – IV)	*'[…] unresponsive, at a criterion level of persistent symptom severity (as used by Honer et al.30) and impaired social function, to an adequate trial of clozapine monotherapy in terms of dosage, duration and adherence […]'*	**Sex:**<br>69.10% males<br><br>**Mean age (years):**<br>39.5<br><br>**Mean duration of illness (years):**<br>n.i.
Behdani 2011 [97]	TOPIR, n = 40<br>PBO, n = 40<br><br>Add on **Clozapine**	parallel double-blind RCT	17	Iran	TOPIR: 200 to 300 mg/d<br>PBO: NA<br><br>CLOZ: n.i.	Schizophrenia (DSM-IV-TR)	*'All were continuously hospitalized for at least six months in chronic wards; in addition, their symptoms were resistant to at least two different antipsychotic therapy trials other than clozapine.'*	**Sex:**<br>85% males<br><br>**Mean age (years):**<br>46.03<br><br>**Mean duration of illness (years):**<br>n.i.
Biederman 1979 [98]	LI, n = 8<br>PBO, n = 5<br><br>Add on **Haloperidol**	parallel double-blind RCT	5	Israel	LI: 1830 mg/d<br>PBO: NA<br><br>HAL(LI): 40 mg/d<br>HAL(PBO): 46 mg/d	Schizoaffective, manic type (RDC)	*'[…] patients with affective symptoms in the context of a severe chronic schizophrenic illness that existed even between superimposed affective episodes; patients without sufficient affective symptoms for Schizoaffective diagnosis, i.e., chronic schizophrenics.'*	**Sex:**<br>n.i.<br><br>**Mean age (years):**<br>n.i.<br><br>**Mean duration of illness (years):**<br>n.i.
Boggeto 1995 [99]	AMI, n = 11<br>FLUO, n = 9<br><br>Add on **Haloperidol**	parallel open-label RCT	8	Italy	AMI: 100 mg/d<br>FLUO: 20 mg/d<br><br>HAL (AMI): 2.2 mg/d<br>HAL (FLUO): 2.4 mg/d	Chronic Schizophrenia (DSM-III-R)	*'The examined subjects had to be treated with haloperidol only and their medication regimen had remained unchanged for at least 6 months before they entered the study. The existence of a definite negative syndrome had to be imperative, demonstrated objectively by a finding o a marked degree (minimum score: 4 of a least 2 items of the 5 on the SANS.'*	**Sex:**<br>55.55% males<br><br>**Mean age (years):**<br>29.49<br><br>**Mean duration of illness (years):**<br>3.39

Study ID	Study arms; n of participants	Study design	Study duration (weeks)	Country	Mean or range drug doses (mg/d)	Diagnosis	Definition of TRS	Population characteristics
Borovicka 2002 [100]	PHENYLPROP, n = 8<br>PBO, n = 8<br><br>Add on **Clozapine**	parallel double-blind RCT	12	USA	PHENYLPROP: 75 mg/d<br>PBO: NA<br><br>CLOZ (PHENYLPROP): 506 mg/d<br>CLOZ(PBO): 431 mg/d	Schizophrenia (DSM – IV)	*'Patients met DSM-IV criteria for schizophrenia and were treatment resistant intolerant or prior to initiation of clozapine treatment [Kane 1988 referred].'*	**Sex:**<br>87.50% males<br><br>**Mean age (years):**<br>44.16<br><br>**Mean duration of illness (years):**<br>n.i.
Buchanan 1996 [101]	FLUO, n = 18<br>PBO, n = 15<br><br>Add on **Clozapine**	parallel double-blind RCT	8	USA	FLUO: 48.90 mg/d<br>PBO: NA<br><br>CLOZ (FLUO): 457.40 mg/d<br>CLOZ (PBO): 511.70 mg/d	Schizophrenia or Schizoaffective Disorder (DSM-III-R)	*'At least 6 months of clozapine treatments with doses of at least 300 mg/day and minimum level of residual (positive or negative symptoms (positive: BPRS positive symptoms total score of at least 8 for the items of conceptual disorganization, suspiciousness, hallucinatory behaviour, and unusual thought content or a score of at least 4 on any one of these items; negative: total score on SANS of at least 20 or a score of 2 or more on at least one global item on this scale). Criteria for partial response to conventional antipsychotic: residual positive or negative symptoms after two trials of drugs from 2 different classes, failure to respond to a 6-week, open-label prospective trial of fluphenazine, participation in a 10-week, double-blind, parallel-groups comparison of haloperidol and clozapine.'*	**Sex:**<br>69.70% males<br><br>**Mean age (years):**<br>34.98<br><br>**Mean duration of illness (years):**<br>15.93
Carpenter 2000 [102]	MAZIN, n = 20<br>PBO, n = 20<br><br>Add on **Clozapine**	crossover double-blind RCT	16	USA	MAZIN: 2.90 mg/d<br>PBO: 17 mg/d<br><br>CLOZ: 496 mg/d<br>*(baseline)*	Schizophrenia or Schizoaffective Disorder (DSM-III-R)	*'Patients were relatively chronic and stable outpatients and were required to exhibit a minimum level of negative symptoms. The minimum negative symptom level was a total score of at least 20 on the Scale for the Assessment of Negative Symptoms, or SANS (Andreasen, 1984), or a score of at least 2 on at least one SANS global item.'*	**Sex:**<br>75% males<br><br>**Mean age (years):**<br>40<br><br>**Mean duration of illness (years):**<br>18
Chang 2008 [103]	ARI, n = 29<br>PBO, n = 32<br><br>Add on **Clozapine**	parallel double-blind RCT	8	South Korea	ARI: 15.50 mg/d<br>PBO: NA<br><br>CLOZ (ARI): 304.30 mg/d<br>CLOZ (PBO): 290.60 mg/d	Schizophrenia (DSM – IV)	*'[…] documented treatment failure prior to clozapine treatment; clozapine treatment for more than I year with at least 8 weeks at a stable daily dose of 400 mg or more; plateau of clinical response to clozapine […]'*	**Sex:**<br>78.69% males<br><br>**Mean age (years):**<br>32.41<br><br>**Mean duration of illness (years):**<br>n.i.

Study ID	Study arms; n of participants	Study design	Study duration (weeks)	Country	Mean or range drug doses (mg/d)	Diagnosis	Definition of TRS	Population characteristics
Chen 2013 [104]	MET, n = 28<br>PBO, n = 27<br><br>Add on **Clozapine**	parallel double-blind RCT	24	Tawain	MET: 500 to 1500 mg/d<br>PBO: NA<br><br>CLOZ(MET): 252.70 mg/d<br>CLOZ(PBO): 282.40 mg/d	Schizophrenia or Schizoaffective disorder (DSM – IV)	*'The current trial of adjunctive metformin for clozapine-treated patients with schizophrenia […]'*	**Sex:**<br>50.90% males<br><br>**Mean age (years):**<br>41.60<br><br>**Mean duration of illness (years):**<br>n.i.
Cipriani 2013 [105]; Barbui 2011 [106]	ARI, n = 53<br>HAL, n = 53<br><br>Add on **Clozapine**	parallel open-label RCT	48	Italy	ARI: 11.80 mg/d<br>HAL: 2.80 mg/d<br><br>CLOZ (HAL): 395 mg/d<br>CLOZ (ARI): 421 mg/d	Schizophrenia (DSM – IV)	*'partially resistant to clozapine monotherapy […] presence of positive symptoms (delusions, hallucinations, abnormal behavior, and clinical diagnosis) […]'*	**Sex:**<br>65% males<br><br>**Mean age (years):**<br>40.90<br><br>**Mean duration of illness (years):**<br>16
De Lucena 2009 [107]	MEM, n = 10<br>PBO, n = 11<br><br>Add on **Clozapine**	parallel double-blind RCT	12	Brazil	MEM: 5 to 20 mg/d<br>PBO: NA<br><br>CLOZ (MEM): 540 mg/d<br>CLOZ (PBO): 659 mg/d	Schizophrenia (DSM – IV)	'*Treatment-refractory, on clozapine, with partial remission of negative symptoms.'*	**Sex:**<br>90.48% males<br><br>**Mean age (years):**<br>34.67<br><br>**Mean duration of illness (years):**<br>18.56
Doruk 2008 [108]	GinkBil, n = 23<br>PBO, n = 23<br><br>Add on **Clozapine**	parallel open-label RCT	12	Turkey	GinkBil: 120 mg/d<br>PBO: NA<br><br>CLOZ (GinkBil): 415 mg/d<br>CLOZ (PBO): 409.10 mg/d	Schizophrenia (DSM – IV)	*'All patients had been treated with at least two kinds of antipsychotic for 6 months or longer, and had shown no response. Clozapine was then administered to these patients with treatment-resistant schizophrenia.<br>Although improvement was observed with clozapine, these patients remained symptomatic [mean BPRS=59.7±9.1, range=39–76].'*	**Sex:**<br>64.29% males<br><br>**Mean age (years):**<br>30.85<br><br>**Mean duration of illness (years):**<br>9.40

Study ID	Study arms; n of participants	Study design	Study duration (weeks)	Country	Mean or range drug doses (mg/d)	Diagnosis	Definition of TRS	Population characteristics
Evins 2000 [109]	GLY, n = 14<br>PBO, n = 13<br><br>Add on **Clozapine**	parallel double-blind RCT	8	USA	GLY: 60 g<br>PBO: NA<br><br>CLOZ: 455 mg/d	Schizophrenia (DSM – IV)	*'Inclusion criteria included a score of 27 or greater on the Scale for SANS, treatment with a stable dose of clozapine for at least 4 weeks.'*	**Sex:**<br>78% males<br><br>**Mean age (years):**<br>39<br><br>**Mean duration of illness (years):**<br>16
Fan 2013 [110]	ARI, n = 20<br>PBO, n = 18<br><br>Add on **Clozapine**	parallel double-blind RCT	8	USA	ARI: 15 mg/d<br>PBO: NA<br><br>CLOZ (ARI): 397 mg/d<br>CLOZ (PBO): 400 mg/d	Schizophrenia or Schizoaffective Disorder (DSM – IV)	*'[…] treatment with clozapine for at least 1 year.'*	**Sex:**<br>73.30% males<br><br>**Mean age (years):**<br>44.20<br><br>**Mean duration of illness (years):**<br>n.i.
Fleischhacker 2010 [111]	ARI, n = 108<br>PBO, n = 99<br><br>Add on **Clozapine**	parallel double-blind RCT	16	European countries South Africa	ARI: 5 to 15 mg mg/d<br>PBO: NA<br><br>CLOZ(ARI): 384 mg/d<br>CLOZ(PBO): 363 mg/d<br>*(baseline only)*	Schizophrenia (DSM-IV-TR)	*'Included patients were not optimally controlled while on clozapine.'*	**Sex:**<br>64.73% males<br><br>**Mean age (years):**<br>38.99<br><br>**Mean duration of illness (years):**<br>13.46
Freudenreich 2007 [112]	RISP, n = 11<br>PBO, n = 13<br><br>Add on **Clozapine**	parallel double-blind RCT	6	USA	RISP: 4 mg/d<br>PBO: NA<br><br>CLOZ: 456 mg/d	Schizophrenia (DSM – IV)	*'Subjects had schizophrenia as their primary diagnosis, and displayed stable residual psychiatric symptoms as defined by a PANSS score greater than 60.'*	**Sex:**<br>87.50% males<br><br>**Mean age (years):**<br>42.30<br><br>**Mean duration of illness (years):**<br>20.60
Freudenreich 2009 [113]	MODF, n = 19<br>PBO, n = 20<br><br>Add on **Clozapine**	parallel double-blind RCT	8	USA	MODF: 300 mg/d<br>PBO: NA<br><br>CLOZ (MODF): 379 mg/d<br>CLOZ (PBO): 361 mg/d	Schizophrenia or Schizoaffective Disorder (DSM – IV)	*'[…] modafinil for clozapine-treated schizophrenia patients […] clinically stable for at least 3 months, and had been taking clozapine for at least 6 months, with a stable dose for at least 1 month.'*	**Sex:**<br>77% males<br><br>**Mean age (years):**<br>45.20<br><br>**Mean duration of illness (years):**<br>19.50

Study ID	Study arms; n of participants	Study design	Study duration (weeks)	Country	Mean or range drug doses (mg/d)	Diagnosis	Definition of TRS	Population characteristics
Friedman 2011 [114]	PIMOZ, n = 25<br>PBO, n = 28<br><br>Add on **Clozapine**	parallel double-blind RCT	12	USA	PIMOZ: 6.48 mg/d<br>PBO: NA<br><br>CLOZ (PIMOZ): 518.80 mg/d<br>CLOZ (PBO): 478.10 mg/d	Schizophrenia or Schizoaffective Disorder (DSM – IV)	*'[..] treatment unresponsive to an optimal trial of clozapine monotherapy. Nonresponse to treatment was defined as the presence, at 4 consecutive weekly screening phase ratings, of persistent positive psychotic symptoms characterized by PANSS scores of 4 or higher on at least 2 items from the positive subscale, >60 a PANSS total score and CGI ≥4; constructs were adopted from the US multicenter trial of clozapine (Kane et al, 1988).'*	**Sex:**<br>77.36% males<br><br>**Mean age (years):**<br>44.92<br><br>**Mean duration of illness (years):**<br>n.i.
Genc 2007 [115]	AMI, n =28<br>QUE, n = 28<br><br>Add on **Clozapine**	parallel single-blind RCT	8	Turkey	AMI: 437.03 mg/d<br>QUE: 595.65 mg/d<br><br>CLOZ(AMI): 550 mg/d<br>CLOZ(QUE): 536,95 mg/d	Schizophrenia (DSM – IV)	*'[…] partial response was defined as persistent psychotic symptoms, as evidenced by a total score >45 on the BPRS [on which each of 18 items is scored from 1 to 7] or a rating of moderately ill [>4] on at least 2 of the 4 BPRS positive symptom items.'*	**Sex:**<br>42% males<br><br>**Mean age (years):**<br>37.30<br><br>**Mean duration of illness (years):**<br>15.60
Goff 1999 [116]	Dcyclos, n = 17<br>PBO, n = 17<br><br>Add on **Clozapine**	crossover double-blind RCT	13	USA	Dcyclos: 50 mg/d<br>PBO: NA<br><br>CLOZ: 490.90 mg/d *(completers)*	Schizophrenia (DSM – IV)	*'[…] prominent negative symptoms (SANS total .30) despite treatment with an optimal dose of clozapine for at least 6 months, […]'*	**Sex:**<br>88.20% males<br><br>**Mean age (years):**<br>36.60<br><br>**Mean duration of illness (years):**<br>14.80
Gunduz-Bruce 2013 [117]	PIMOZ, n = 14<br>PBO, n = 14<br><br>Add on **Clozapine**	parallel double-blind RCT	12	USA	PIMOZ: 1 to 4 mg/d<br>PBO: NA<br><br>CLOZ: n.i.	Schizophrenia or Schizoaffective Disorder (DSM – IV)	*'[…] patients with schizophrenia currently receiving clozapine with partial response, […] A minimum BPRS score of 35 and a BPRS psychotic symptom cluster score of at least 8were required.'*	**Sex:**<br>71.43% males<br><br>**Mean age (years):**<br>42.90<br><br>**Mean duration of illness (years):**<br>n.i.

Study ID	Study arms; n of participants	Study design	Study duration (weeks)	Country	Mean or range drug doses (mg/d)	Diagnosis	Definition of TRS	Population characteristics
Hatta 2012 [118]	OLA, n = 13<br>PBO, n = 13<br><br>Add on **Risperidone**	parallel single-blind RCT	8	Japan	OLA: 16,9mg/d<br>PBO: NA<br><br>RISP: 7mg/d	Schizophrenia, or Schizophreniform, or Schizoaffective Disorder (DSM-IV-TR)	*'[…] and early non-responders (CGI >= 4) despite 2 weeks of treatment with risperidone.'*	**Sex:**<br>50% males<br><br>**Mean age (years):**<br>39.35<br><br>**Mean duration of illness (years):**<br>n.i.
Hatta 2014a [119]	RISP, n = 14<br>PBO, n = 13<br><br>Add on **Olanzapine**	parallel single-blind RCT	10	Japan	RISP: 9 mg/d<br>PBO: NA<br><br>OLA: 16,1mg/d	Schizophrenia or Schizophreniform or Schizoaffective Disorder (DSM-IV-TR)	*'[…] non-responders to risperidone in acute-phases schizophrenia […] CGI-I score >=4 after 2 weeks of risperidone treatment.'*	**Sex:**<br>66.66% males<br><br>**Mean age (years):**<br>40.14<br><br>**Mean duration of illness (years):**<br>12.30
Hatta 2014b [119]	OLA, n = 11<br>PBO, n = 13<br><br>Add on **Risperidone**	parallel single-blind RCT	10	Japan	OLA: 17.50 mg/d<br>PBO: NA<br><br>RISP: 8.10 mg/d	Schizophrenia or Schizophreniform or Schizoaffective Disorder (DSM-IV-TR)	*'[…] non-responders to olanzapine in acute-phases schizophrenia […] CGI-I score >=4 after 2 weeks of olanzapine treatment.'*	**Sex:**<br>13.60% males<br><br>**Mean age (years):**<br>47<br><br>**Mean duration of illness (years):**<br>16.40
Honer 2006 [120]	RISP, n = 34<br>PBO, n = 34<br><br>Add on **Clozapine**	parallel double-blind RCT	8	Canada<br>China<br>Germany<br>UK	RISP: 2.94 mg/d<br>PBO: NA<br><br>CLOZ: 492 mg/d	Schizophrenia Or Schizoaffective Disorder (DSM – IV)	*'[…] the indication of poor response to other antipsychotic agents; treatment for at least 12 weeks at a stable dose of 400mg or more per day, unless the size of the dose was limited by side effects; a total score of 80 or greater at baseline on the PANSS […]'*	**Sex:**<br>74% males<br><br>**Mean age (years):**<br>37.20<br><br>**Mean duration of illness (years):**<br>15
Hosseini 2014 [121]	DESMOP, n = 22<br>PBO, n = 22<br><br>Add on **Risperidone**	parallel double-blind RCT	8	Iran	DESMOP: 10 to 20 mcg/d<br>PBO:NA<br><br>RISP(DESMOP): 5.20 mg/d<br>RISP(PBO): 5.40 mg/d	Schizophrenia (DSM-IV-TR)	*'[…] chronic schizophrenia, minimum score of 60 PANSS, […] treated with a stable dose of risperidone for at least four weeks and had been partially stabilized (<20% change on the PANSS total score in 2 subsequent visits 1 week apart) prior to entry.'*	**Sex:**<br>81.82% males<br><br>**Mean age (years):**<br>33.89<br><br>**Mean duration of illness (years):**<br>9.09

Study ID	Study arms; n of participants	Study design	Study duration (weeks)	Country	Mean or range drug doses (mg/d)	Diagnosis	Definition of TRS	Population characteristics
Ibrahim 2019 [122]	VALPRO, n = 46<br>PBO, n = 48<br><br>Add on **Risperidone**	parallel double-blind RCT	18	Egypt	VALPRO: 50 to 100 mg/mL<br>PBO: NA<br><br>RISP: n.i.	Schizophrenia Or Schizoaffective Disorder (DSM – IV)	*'[…] receiving a stable dose of risperidone for a month or more; score 4 or more on one or more items of the Positive and Negative Syndrome Scale (Kay et al.,1987).'*	**Sex:**<br>60.60% males<br><br>**Mean age (years):**<br>25.64<br><br>**Mean duration of illness (years):**<br>n.i. (<4)
Josiassen 2005 [123]	RISP, n = 20<br>PBO, n = 20<br><br>Add on **Clozapine**	parallel double-blind RCT	12	USA	RISP: 4.43 mg/d<br>PBO: NA<br><br>CLOZ: 396.90 mg/d *(baseline)*	Schizophrenia or Schizoaffective Disorder (DSM – IV)	*'[…] had, before treatment with clozapine, documented treatment failure after two antipsychotics approved by the U.S. Food and Drug Administration were administered for an adequate duration in a sufficient dose (6 or more weeks of 1000mg/d of chlorpromazine equivalents); demonstrated a documented failure to show a satisfactory clinical response to an adequate trial of clozapine (3 or more months of at least 600mg/d of oral clozapine or a plasma drug level of 350ng/ml or higher); and 5) had persistent psychotic symptoms, as evidenced by either a total score of at least 45 on the BPRS or a rating of moderately ill (4 or more) on at least two of the four BPRS positive symptom items (hallucinatory behavior, conceptual disorganization, unusual thought content, and suspiciousness).'*	**Sex:**<br>87.50% males<br><br>**Mean age (years):**<br>40.35<br><br>**Mean duration of illness (years):**<br>22.10
Kelly 2015 [124]	MINOC, n = 29<br>PBO, n = 23<br><br>Add on **Clozapine**	parallel double-blind RCT	10	USA	MINOC: 50 to 100 mg/b.i.d.<br>PBO: NA<br><br>CLOZ (MINOC): 423.1 mg/d<br>CLOZ (PBO): 433.7 mg/d	Schizophrenia or Schizoaffective disorder (DSM-IV-TR)	*'Participants had been taking clozapine for at least 6 months prior to study screening with past dose of at least 200mg/day and had achieved a serum clozapine level of >350ng/ml. Patients had persistent positive symptoms, defined by 1) BPRS total score 45 or CGI severity score 4; and 2) BPRS positive symptom item total score of 8 with a score 4 on at least one individual item.'*	**Sex:**<br>74.51% males<br><br>**Mean age (years):**<br>42.63<br><br>**Mean duration of illness (years):**<br>n.i.
Kotler 2004 [125]	SUL, n = 9<br>OLA (only), n = 8<br><br>Add on **Olanzapine**	parallel open-label RCT	8	Israel	SUL: 600 mg/d<br><br>OLA (SUL): 22.2 mg/d<br>OLA: 22.5 mg/d	Schizophrenia (DSM – IV)	*'[…] a minimum score of at least 70 on the Positive and Negative Syndrome Scale (PANSS) (Kay et al., 1987). All subjects met parameters for treatment-resistance following the criteria of Kane et al. (1988).'*	**Sex:**<br>52.90% males<br><br>**Mean age (years):**<br>31.80<br><br>**Mean duration of illness (years):**<br>11.33

Study ID	Study arms; n of participants	Study design	Study duration (weeks)	Country	Mean or range drug doses (mg/d))	Diagnosis	Definition of TRS	Population characteristics
Krivoy 2017 [126]	VitD3, n = 24<br>PBO, n = 23<br><br>Add on **Clozapine**	parallel double-blind RCT	8	Israel	VitD3: 14.000 IU/d<br>PBO: NA<br><br>CLOZ (VitD3): 414.60 mg/d<br>CLOZ (PBO): 414.10 mg/d	Schizophrenia (n.i.)	*'[…] chronic schizophrenia treated with clozapine for at least 18weeks and being on a stable clozapine dose for at least four weeks prior to enrollment and total severity of psychopathology score, as measured by the PANSS total score above 70.'*	**Sex:**<br>68.08% males<br><br>**Mean age (years):**<br>40.90<br><br>**Mean duration of illness (years):**<br>16.20
Lane 2006 [127]	SARC, n = 10<br>PBO, n = 10<br><br>Add on **Clozapine**	parallel double-blind RCT	6	China	SARC: 2 gr/d<br>PBO: NA<br><br>CLOZ (SARC): 306 mg/d<br>CLOZ (PBO): 305 mg/d	Schizophrenia (DSM – IV)	*'The patients were treatment resistant to at least two different classes of antipsychotics, in doses equal to at least 400-600mg of chlorpromazine per day for at least 8 weeks. Also, patients received adequate trials of clozapine but without satisfactory response with total scores 70 or higher on the PANSS) (Kay et al 1987).'*	**Sex:**<br>70% males<br><br>**Mean age (years):**<br>36.10<br><br>**Mean duration of illness (years):**<br>n.i.
Lang 2023 [128]	SER, n = 17<br>OLA, n = 17<br><br>Add on **Olanzapine**	parallel single-blind RCT	24	China	SER: 7.5 to 10 mg/d<br>OLA: 50 to 100 mg/d	Schizophrenia (DSM – IV)	*'Treatment resistance was determined according to the criteria of Howes et al. (2017): continued psychosis despite at least two treatment courses with typical antipsychotics at a dose equivalent of chlorpromazine = 800 mg/day for 6 weeks and post-treatment failure to reduce the BPRS total score by =20%, a BPRS score of =45, or a CGI-S score by 4.'*	**Sex:**<br>44.12% males<br><br>**Mean age (years):**<br>45.55<br><br>**Mean duration of illness (years):**<br>13.25
Lee 2011 [129]	MIRT, n = 12<br>PBO, n = 9<br><br>Add on **Risperidone**	parallel double-blind RCT	8	South Korea	MIRT: 15 to 30 mg/d<br>PBO: NA<br><br>RISP (MIRT): 3 mg/d<br>RISP(PBO): 4.22 mg/d	Schizophrenia (DSM – IV)	*'[…] stabilized outpatients with schizophrenia undergoing risperidone treatment […] score of at least 4 on the Clinical Global Impressions scale (CGI; Guy, 1982) and stable, with no changes in CGI (as determined by two investigators, SH Lee and TK Choi) or medication dosage, for 8 weeks […]'*	**Sex:**<br>50% males<br><br>**Mean age (years):**<br>35.69<br><br>**Mean duration of illness (years):**<br>6.5

Study ID	Study arms; n of participants	Study design	Study duration (weeks)	Country	Mean or range drug doses (mg/d))	Diagnosis	Definition of TRS	Population characteristics
Lin 2018 [130]	BEZOAET, n = 32[*]<br>PBO, n = 19[*]<br><br>Add on **Clozapine**<br><br>*(only patients receiving clozapine monotherapy included in the analysis)*	parallel double-blind RCT	6	China Tawain	BEZOAET: 1.50 gr/d<br>PBO: NA<br><br>CLOZ (BENZOAET): 270 mg/d<br>CLOZ (PBO): 242.50 mg/d	Schizophrenia (DSM – IV)	*'[…] were resistant to standard treatments of at least two specific antipsychotics before clozapine treatment; 5) were receiving adequate trials of clozapine for more than 12 weeks but without satisfactory response, with a minimum baseline total score of 70 on the PANSS and a minimum baseline total score of 40 on SANS […]'*	**Sex:**<br>68.33% males<br><br>**Mean age (years):**<br>44.80<br><br>**Mean duration of illness (years):**<br>23.21
Lu 2004 [131]	CLOZ, n = 34<br>FLUV, n = 34<br><br>Add on **Clozapine**	parallel open-label RCT	12	China Tawain	CLOZ: 307.4 mg/d<br>FLUV: 50 to 250 mg/d<br><br>CLOZ (FLUV): 130.10 mg/d	Schizophrenia (DSM – IV)	*'[…] treatment-resistant to typical anti psychotics, which was modified from the criteria proposed by Kane et al. (1988), Conley and Kelly (2001) and Howes et al. (2017).'*	**Sex:**<br>29.41% males<br><br>**Mean age (years):**<br>34<br><br>**Mean duration of illness (years):**<br>n.i.
Lu 2018 [132]	CLOZ, n = 43<br>FLUV, n = 42<br><br>Add on **Clozapine**	parallel double-blind RCT	12	Taiwan	CLOZ: 200 mg/d FLUV: 50 mg/d	Schizophrenia (DSM – IV)	*'[…] treatment-resistant to typical antipsychotics, which was modified from the criteria proposed by Kane et al. (1988), Conley and Kelly (2001) and TRRIP Working Group (Howes et al., 2017) […] patients satisfied the TRS criteria for unambiguous lack of improvement when they had at least 6 weeks of continuous treatment with two or more typical or atypical antipsychotics at doses of at least 600 mg of CPZ equivalents, a poor level of functioning over the last 5 years, and persistent psychotic symptoms of at least moderate severity (as indexed by PANSS scores (Kay et al., 1987) on two or more positive subscale measures).'*	**Sex:**<br>71.77% males<br><br>**Mean age (years):**<br>44.89<br><br>**Mean duration of illness (years):**<br>n.i.
Mayabhate 2014 [133]	ARI, n = 30<br>PALIP, n= 30<br>PBO, n = 30<br><br>Add on **Olanzapine**	parallel double-blind RCT	6	India	ARI: 10 mg/d<br>PALIP: 3 mg/d<br>PBO: NA<br><br>OLA: 10 mg/d	Schizophrenia (DSM – IV)	*'[…] all diagnosed patients of schizophrenia by DSM-IV diagnostic criteria that are on olanzapine therapy for at least 6 weeks showing partial or no response to the treatment which is measured in terms of PANSS scale and those found meeting the inclusion criteria were taken into the consideration.'*	**Sex:**<br>63.33% males<br><br>**Mean age (years):**<br>36.46<br><br>**Mean duration of illness (years):**<br>n.i.

Study ID	Study arms; n of participants	Study design	Study duration (weeks)	Country	Mean or range drug doses (mg/d)	Diagnosis	Definition of TRS	Population characteristics
Mico 2011 [134]	DUL, n = 20<br>PBO, n = 20<br><br>Add on **Clozapine**	parallel double-blind RCT	16	Italy	DUL: 60 mg/d<br>PBO: NA<br><br>CLOZ (DUL): 503.30 mg/d<br>CLOZ (PBO): 533.30 mg/d	Schizophrenia (DSM – IV)	*'All patients had been on clozapine monotherapy at the highest tolerable range(450–650mg/day), for at least 1 year; the dose had been stable for at least 1 month before the study and was left unchanged throughout the study. […] Patients scoring 25 or more on the BPRS scale (Overall and Gorham, 1962) at both the screening and baseline evaluation were classified as partial responders or non-responders (Munro et al., 2004).'*	**Sex:**<br>60% males<br><br>**Mean age (years):**<br>34.95<br><br>**Mean duration of illness (years):**<br>6.85
Moazen-Zadeh 2020 [135]	VORT, n = 39<br>PBO, n = 39<br><br>Add on **Risperidone**	parallel double-blind RCT	8	Iran	VORT: 10 mg/b.i.d.<br>PBO: NA<br><br>RISP (VORT): 4.10 mg/d<br>RISP (PBO): 4.15 mg/d	Schizophrenia (DSM – V)	*'Treated with risperidone for at least 8 weeks, stable for a minimum 4 weeks prior to the study, PANSS negative >= 16 before the start of treatment with vortioxetine.'*	**Sex:**<br>69.12% males<br><br>**Mean age (years):**<br>33.66<br><br>**Mean duration of illness (years):**<br>9.03
Modabbernia 2013 [136]	OXYT, n = 20<br>PBO, n = 20<br><br>Add on **Risperidone**	parallel double-blind RCT	8	Iran	OXYT: 20 (5 sprays) to 40 (10 sprays)/b.i.d.<br>PBO: NA<br><br>RISP (OXYT): 5.80 mg/d<br>RISP (PBO): 5.70 mg/d	Schizophrenia (DSM-IV-TR)	*'[…] the patients were required to be treated with a stable dose of risperidone for at least 4 weeks and had been partially stabilized (\20 % change on the Positive and visits 1 week apart) prior to entry […] had significant residual symptoms as defined by a minimum score of 60 on PANSS following treatment with risperidone.'*	**Sex:**<br>82.50% males<br><br>**Mean age (years):**<br>32.75<br><br>**Mean duration of illness (years):**<br>6.35
Mossaheb 2006 [137]	HAL, n = 5<br>PBO, n = 5<br><br>Add on **Clozapine**	parallel double-blind RCT	10	Austria	HAL: 4 mg/d<br>PBO: NA<br><br>CLOZ: 520 mg/d	Schizophrenia (n.i.)	*'[…] resistant to two adequate trials with two different antipsychotics and to a trial with clozapine during a minimum of 6−8 weeks in adequate dosage were included in the study.'*	**Sex:**<br>n.i.<br><br>**Mean age (years):**<br>32.50<br><br>**Mean duration of illness (years):**<br>n.i.

Study ID	Study arms; n of participants	Study design	Study duration (weeks)	Country	Mean or range drug doses (mg/d)	Diagnosis	Definition of TRS	Population characteristics
Muscatello 2010 [138]	TOPIR, n = 30<br>PBO, n = 30<br><br>Add on **Clozapine**	parallel double-blind RCT	24	Italy	TOPIR: 25 to 200 mg/d<br>PBO: NA<br><br>CLOZ (TOPIR): 330.30 mg/d<br>CLOZ (PBO): 327.30 mg/d	Schizophrenia (DSM – IV)	*'[…] met DSM-IV criteria for schizophrenia and demonstrated persistent positive and negative symptoms despite an adequate trial of clozapine, were included in this study.'*	**Sex:**<br>52.67% males<br><br>**Mean age (years):**<br>32.40<br><br>**Mean duration of illness (years):**<br>5.58
Muscatello 2011[139]	ARI, n = 20<br>PBO, n = 20<br><br>Add on **Clozapine**	parallel double-blind RCT	24	Italy	ARI: 10 to 15 mg/d<br>PBO: NA<br><br>CLOZ (ARI): 310.70 mg/d<br>CLOZ (PBO): 341.20 mg/d	Schizophrenia (DSM – IV)	*'[…] met DSM-IV criteria for schizophrenia and demonstrated persistent positive and negative symptoms despite an adequate trial of clozapine, were included in this study.'*	**Sex:**<br>57.50% males<br><br>**Mean age (years):**<br>31.30<br><br>**Mean duration of illness (years):**<br>n.i.
Muscatello 2014 [140]	ZIP, n = 20<br>PBO, n = 20<br><br>Add on **Clozapine**	parallel double-blind RCT	16	Italy	ZIP: 80 mg/d<br>PBO: NA<br><br>CLOZ (ZIP): 428.70 mg/d<br>CLOZ (PBO): 462.50 mg/d	Schizophrenia (DSM – IV)	*'[…] demonstrated persistent positive and negative symptoms despite an adequate trial of clozapine, were included in this study. Patients scoring 25 or more on the BPRS 17 at baseline evaluation were classified as partial-responders or non responders.'*	**Sex:**<br>32.50% males<br><br>**Mean age (years):**<br>35<br><br>**Mean duration of illness (years):**<br>n.i.
Neill 2022 [141]	Nacetyl, n = 42<br>PBO, n = 43<br><br>Add on **Clozapine**	parallel double-blind RCT	52	Australia	Nacetyl: 2gr/d<br>PBO: NA<br><br>CLOZ: n.i.	Schizophrenia or Schizoaffective Disorder (DSM – V)	*'[…] they were on a stable dose of clozapine for at least 6 months, and, despite adequate dosing (serum level of >350 µg/L) they continued to experience residual symptoms, defined as either a score of >4 on two or more PANSS negative items or a total PANSS score ≥60.'*	**Sex:**<br>71.70% males<br><br>**Mean age (years):**<br>39.74<br><br>**Mean duration of illness (years):**<br>18.37
Nielsen 2012 [142]	SERTIND, n = 25<br>PBO, n = 25<br><br>Add on **Clozapine**	parallel double-blind RCT	12	Denmark	SERTIND: 16 mg/d<br>PBO: NA<br><br>CLOZ: 414.50 mg/d	Schizophrenia (ICD – 10)	*'Patients must have been treated with clozapine for at least 6 months at an optimized dose (minimum 150 mg/d).'*	**Sex:**<br>60% males<br><br>**Mean age (years):**<br>42.25<br><br>**Mean duration of illness (years):**<br>29.35

Study ID	Study arms;<br>n of participants	Study design	Study duration (weeks)	Country	Mean or range drug doses (mg/d)	Diagnosis	Definition of TRS	Population characteristics
Potkin 1999 [143]	GLY, n = 12<br>PBO, n = 12<br><br>Add on **Clozapine**	parallel double-blind RCT	12	USA	GLY: 30g/d<br>PBO: NA<br><br>CLOZ (GLY): 589 mg/d<br>CLOZ (PBO): 635 mg/d	Schizophrenia (DSM – III-R)	*'[…] chronically hospitalized (mean=12.4 years, SD=7.2) […]., and were maintained on optimal doses of clozapine (range =400–1200 mg/day) for several months before the start of the trial. Although improvement was observed with clozapine, these patients remained symptomatic (mean BPRS score=43.2, SD=12.2) and required continual hospitalization.'*	**Sex:**<br>87,5 % males<br><br>**Mean age(years):**<br>34.85<br><br>**Mean duration of illness (years):**<br>n.i.
Repo-Tiihonen 2012 [144]	OLA, n = 5<br>PBO, n = 7<br><br>Add on **Clozapine**	crossover double-blind RCT	24	Finland	OLA: n.i.<br>PBO: NA<br><br>CLOZ: n.i.	Schizophrenia (n.i.)	*'Insufficient response to the medication considered was observed when the GAF12 had been rated <25 and, on clinical treatment-resistance reported by each patient's own physician.'*	**Sex:**<br>91.60% males<br><br>**Mean age (years):**<br>47.07<br><br>**Mean duration of illness (years):**<br>n.i.
Samadi 2015 [145]	CELECOX, n = 20<br>PBO, n = 20<br><br>Add on **Clozapine**	parallel double-blind RCT	4	Iran	CELECOX: 200 mg/d<br>PBO: NA<br><br>CLOZ (CELECOX): 88.29 mg/d<br>CLOZ (PBO): 87.28 mg/d	Schizophrenia (DSM – V)	*'[…] treatment resistant schizophrenia (based on research trials, 1-3 trials that used adequate doses of antipsychotic drugs, each lasting for more than 4-6 weeks, have failed to reduce positive symptoms sufficiently) [....]'*	**Sex:**<br>85.70% males<br><br>**Mean age (years):**<br>28.50<br><br>**Mean duration of illness (years):**<br>n.i.
Samadi 2017 [146]	ONDA, n = 18<br>PBO, n = 20<br><br>Add on **Risperidone**	parallel double-blind RCT	12	Iran	ONDA: 4 to 8 mg/d<br>PBO: NA<br><br>RISP: 8 mg/d	Schizophrenia (DSM-IV-TR)	*'All the subjects had been receiving a similar dosage of an atypical antipsychotic medicine (risperidone 4–6 mg/d) for at least 2 months prior to enrollment. The cutoff point for the negative PANSS was >15.13.'*	**Sex:**<br>92.10% males<br><br>**Mean age (years):**<br>40<br><br>**Mean duration of illness (years):**<br>n.i.
Samaei. 2020 [147]	RESVER, n = 26<br>PBO, n = 26<br><br>Add on **Risperidone**	parallel double-blind RCT	8	Iran	RESVER: 200 mg/d<br>PBO NA<br><br>RISP: 4 to 6 mg/d	Schizophrenia (DSM – V)	*'[…] who met the diagnostic criteria for chronic schizophrenia based on the fifth edition of DSM and a minimum disease duration of 2 years (based on the Structured Clinical Interview for DSM-5 Clinical Version (2013).'*	**Sex:**<br>53.30% males<br><br>**Mean age (years):**<br>33.90<br><br>**Mean duration of illness (years):**<br>11

Study ID	Study arms; n of participants	Study design	Study duration (weeks)	Country	Mean or range drug doses (mg/d)	Diagnosis	Definition of TRS	Population characteristics
Salehi 2022 [148]	PALMIT, n= 30<br>PBO, n= 30<br><br>Add on **Risperidone**	parallel double blind RCT	8	Iran	PALMIT: 600 mg/b.i.d.<br>PBO: NA<br><br>RISP: n.i.	Chronic Schizophrenia (DSM – V)	*'Participants had a baseline score ≥15 in negative subscale of PANSS, a baseline score <14 on HDRS, and clinical stability on a stable dose of risperidone for at least 8 weeks prior to the trial, defined as ≤20% change in the total score of PANSS on 2 successive assessments with a 2-week interval. These inclusion criteria were determined to select only those patients with primary negative symptoms.'*	**Sex:**<br>88% males<br><br>**Mean age (years):**<br>35.28<br><br>**Mean duration of illness (years):**<br>9.34
Schulz 1999 [149]	LI, n = 21<br>PBO, n = 20<br><br>Add on **Fluphenazine decanoate**	parallel double-blind RCT	8	USA	LI: 900 mmg/d<br>PBO: NA<br><br>FLUPHLA: 12.50 to 50 mg/d	Schizophrenia or Schizoaffective Disorder (DSM – III)	*'[…] patients to be 'stabilized' before entry into the trial […] up to 6 months of treatment with fluphenazine decanoate with the goal of patient stabilization. Patients were eligible for the lithium study if they failed to fulfill TSS stabilization criteria; stable psychotic symptoms on the BPRS over four weeks [….]'*	**Sex:**<br>82.90% males<br><br>**Mean age (years):**<br>29.42<br><br>**Mean duration of illness (years):**<br>n.i.
Shamabadi 2025 [150]	PTX, n = 37<br>PBO, n = 37<br><br>Add on **Risperidone**	parallel double-blind RCT	8	Iran	PTX: 800 mg/d<br>PBO: NA<br><br>RISP: 4 to 6 mg/d	Schizophrenia (DSM – V)	*'[…] diagnosed with schizophrenia for at least 2 years and received risperidone for at least 2 months […] had a negative subscale score of >15 on the PANSS (Kay et al., 1987) and a total score of <14 ON the HDRS (Hamilton, 1960). Patientsnded to be clinically stable, with a PANSS score change of ≤20% over 2 consecutive visits spaced 2 weeks apart while receiving a stable dose of risperidone […]'*	**Sex:**<br>55.07% males<br><br>**Mean age (years):**<br>36.42<br><br>**Mean duration of illness (years):**<br>13.51
Shi 2022 [151]	SER, n = 53<br>ZIP, n = 62<br><br>Add on **Ziprasidone**	parallel double-blind RCT	24	China	SER: 50 mg/d<br>ZIP: 120 to 160 mg/d<br><br>ZIP: 60 to 80 mg/d	Schizophrenia (DSM – IV)	*'Acutely relapsed patients were required to meet the following inclusion criteria: 1) antipsychotic treatment (at least 2 chemical classes) with a dose equivalence of CPZ ≥800mg/d for 6 weeks, each without significant relief of clinical symptoms, and failure to improve by at least 20% in total BPRS score; 2) a BPRS score ≥45 and a CGI score ≥4 in this evaluation.'*	**Sex:**<br>56% males<br><br>**Mean age (years):**<br>41.30<br><br>**Mean duration of illness (years):**<br>13.54

Study ID	Study arms; n of participants	Study design	Study duration (weeks)	Country	Mean or range drug doses (mg/d)	Diagnosis	Definition of TRS	Population characteristics
Shiloh 1997 [152]	SUL, n = 16<br>PBO, n = 12<br><br>Add on **Clozapine**	parallel double-blind RCT	10	Israel	SUL: 600 mg/d<br>PBO: NA<br><br>CLOZ (SUL): 403.10 mg/d<br>CLOZ (PBO): 445.80 mg/d	Schizophrenia (DSM – IV)	*'Twenty-eight people with schizophrenia, previously unresponsive to typical antipsychotics and only partially responsive to current treatment with clozapine […]'*	**Sex:**<br>67.85% males<br><br>**Mean age (years):**<br>38.92<br><br>**Mean duration of illness (years):**<br>19.98
Shoja-Shafti 2017 [153]	FLUPHLA, n = 12<br>ARI*, n = 12<br><br>Add on **Aripiprazole**<br><br>**continuing current treatment*	parallel single-blind RCT	8	Iran	FLUPHLA: 10 to 20 mg/2 weeks IM<br><br>ARI: 30 mg/d<br>ARI*: 30 mg/d	Schizophrenia (DSM – V)	*'[…] who had shown poor response to aripiprazole (SAPS > 70 at baseline, with maximum dose of 30 mg daily for at least 4 weeks, as inclusion criteria) were entered […]'*	**Sex:**<br>100% males<br><br>**Mean age (years):**<br>34.49<br><br>**Mean duration of illness (years):**<br>7.8
Siris 1991 [154]	IMI, n = 10<br>PBO, n = 17<br><br>Add on **Fluphenazine decanoate**	parallel double-blind RCT	6	USA	IMI: 50 to 150 mg/d<br>PBO: NA<br><br>FLUPHLA: 25 g/cc/week	Schizophrenia or Schizoaffective Disorder (RDC)	*'[…] patients manifested consistent negative symptoms and the negative symptoms did not response to antiparkinsonian trial […]'*	**Sex:**<br>66.60% males<br><br>**Mean age (years):**<br>34<br><br>**Mean duration of illness (years):**<br>n.i.
Siskind 2021 [155]	MET, n= 10<br>PBO, n= 10<br><br>Add on **Clozapine**	parallel double-blind RCT	24	Australia	MET: 500 to 2000 mg/d<br>PBO: NA<br><br>CLOZ: n.i.	Schizophrenia or Schizoaffective Disorder (DSM – IV)	*'The participants were being commenced on clozapine as part of routine clinical care.<br>The criteria for clozapine in Australia are the same as the TRRIP guidelines - inadequate response to at least 2 antipsychotics with ongoing psychotic symptoms and functional deficits.'*	**Sex:**<br>85% males<br><br>**Mean age (years):**<br>33.45<br><br>**Mean duration of illness (years):**<br>n.i.
Stryjer 2004 [156]	DONEP, n = 8<br>PBO, n = 8<br><br>Add on **Clozapine**	crossover double-blind RCT	18	Israel	DONEP: 5 to 10 mg/d<br>PBO: NA<br><br>CLOZ: 466.60 mg/d	Schizophrenia (DSM – IV)	*'[…] treated with a stable tolerated dose of CLOZ as monotherapy for a minimum period of 6 months and still present active psychotic symptomatology, and to have a history of nonresponse to two different antipsychotic drugs of different classes at equivalent CPZ dose of 1000 mg for a period of at least 8 week.'*	**Sex:**<br>66.60% males<br><br>**Mean age (years):**<br>34.80<br><br>**Mean duration of illness (years):**<br>7.6

Study ID	Study arms; n of participants	Study design	Study duration (weeks)	Country	Mean or range drug doses (mg/d)	Diagnosis	Definition of TRS	Population characteristics
Sulejmanpasic-Arslanagic 2019 [157]	AMI, n = 2<br>PBO, n = 2<br><br>Add on **Clozapine**	parallel double-blind RCT	8	Bosnia-Herzegovina	AMI: 450 mg/d<br>PBO: NA<br><br>CLOZ: 450 mg/d	Schizophrenia (n.i.)	*'[…] patients with treatment-resistant schizophrenia […]'*	**Sex:**<br>n.i.<br><br>**Mean age (years):**<br>n.i.<br><br>**Mean duration of illness (years):**<br>n.i.
Talbot 1964 [158]	TRIFLUO, n = 27<br>CPZ, n= 25<br>PBO, n = 25<br><br>Add on **Chlorpromazine** (+TRIFLUO) **or Trifluoperazine** (+CPZ)	parallel double-blind RCT	32	USA	TRIFLUO: 5 mg/b.i.d.<br>CPZ: 300 mg/b.i.d.<br>PBO: NA<br><br>CPZ: 150 mg/b.i.d. (+TRIFLUO)<br>TRIFLUO: 10 mg/b.i.d. (+CPZ)	Schizophrenia (n.i.)	*'[…] residuals symptoms regarding treatment pharmacotherapy […]'*	**Sex:**<br>n.i.<br><br>**Mean age (years):**<br>n.i.<br><br>**Mean duration of illness (years):**<br>n.i.
Tiihonen 2003 [159]	LAM, n = 16<br>PBO, n = 16<br><br>Add on **Clozapine**	crossover double-blind RCT	14	Finland	LAM: 25 to 200 mg/d<br>PBO: NA<br><br>CLOZ: 558 mg/d	Schizophrenia (DSM – IV)	*'[…] non satisfactory response with ongoing clozapine treatment (duration of clozapine treatment at least 6 months). All patients had been previously classified as being non-responders for other antipsychotic drugs after receiving an optimal dose of at least 2 other neuroleptics for at least 3 months.'*	**Sex:**<br>100% males<br><br>**Mean age (years):**<br>38.30<br><br>**Mean duration of illness (years):**<br>13.60
Tsai 1999 [160]	Dser, n = 10<br>PBO, n = 10<br><br>Add on **Clozapine**	parallel double-blind RCT	6	Taiwan	Dser: 30mg/d<br>PBO: NA<br><br>CLOZ (Dser): 363 mg/d<br>CLOZ(PBO): 315mg/d	Schizophrenia (DSM – IV)	*'The patients had not responded to conventional antipsychotics and were classified as treatment resistant according to the definition of Kane et al.'*	**Sex:**<br>55% males<br><br>**Mean age (years):**<br>41<br><br>**Mean duration of illness (years):**<br>20.25
Vayısoğlu 2013 [161]	LAM, n = 17<br>PBO, n = 17<br><br>Add on **Clozapine**	parallel double-blind RCT	12	Turkey	LAM: 35 to 200 mg/d<br>PBO: NA<br><br>CLOZ (LAM): 514.70 mg/d<br>CLOZ(PBO): 426,4mg/d	Schizophrenia (DSM – IV)	*'Patients included were 18–65 years old, with a PANSS score of at least 70 and CGI score of at least 3, receiving 150 - 900 mg/d of clozapine treatment for at least a year, with a stable dose in the previous month.'*	**Sex:**<br>67.64% males<br><br>**Mean age (years):**<br>40.85<br><br>**Mean duration of illness (years):**<br>18.25

Study ID	Study arms; n of participants	Study design	Study duration (weeks)	Country	Mean or range drug doses (mg/d)	Diagnosis	Definition of TRS	Population characteristics
Veerman 2016 [162]	MEM, n = 26<br>PBO, n = 26<br><br>Add on **Clozapine**	crossover double-blind RCT	12	Netherlands	MEM: 10 to 20 mg/d<br>PBO: NA<br><br>CLOZ: 350 mg/d	Schizophrenia (DSM – IV)	*'[..] and failed to achieve remission criteria proposed by Andreasen et al. (2005), [...] conversation. At inclusion, duration of clozapine therapy was at least 6 months level above 350 ng/ml or intolerability to achieve this threshold (Schulte, 2003).'*	**Sex:**<br>75% males<br><br>**Mean age (years):**<br>42.35<br><br>**Mean duration of illness (years):**<br>22.88
Weiner 2010 [163]	RISP, n = 33<br>PBO, n = 36<br><br>Add on **Clozapine**	parallel double-blind RCT	16	USA	RISP: 3.96 mg/d<br>PBO: NA<br><br>CLOZ: n.i.	Schizophrenia or Schizoaffecti ve Disorder (DSM – IV)	*'They met the following treatment-resistance criteria: (1) BPRS; Overall and Gorham, 1962 total score of 45 or CGI severity of illness item score of 4; and (2) BPRS positive symptom item total score of 8, with one or more item rated 4.'*	**Sex:**<br>68.75% males<br><br>**Mean age (years):**<br>46.11<br><br>**Mean duration of illness (years):**<br>n.i.
Wilson 1993 [164]	LI, n = 12<br>PBO, n = 10<br><br>Add on **Haloperidol**	parallel double-blind RCT	14	USA	LI: 1419 mg/d<br>PBO: NA<br><br>HAL (LI): 19 mg/d<br>HAL (PBO): 25.90 mg/d	Schizophrenia (DSM-III-R)	*'persistent psychosis during treatment with conventional antipsychotics for not less than 8 weeks at a dose equivalent of at least 800 mg/day of chlorpromazine by history; [...]'*	**Sex:**<br>58.26% males<br><br>**Mean age (years):**<br>34<br><br>**Mean duration of illness (years):**<br>n.i.
Woo 2022 [165]	AMI=13<br>OLA*=12<br><br>Add on **Olanzapine**<br><br>**continuing current treatment*	parallel, single-blind RCT	4	South Korea	AMI: 550mg/d<br><br>OLA (+AMI): 16.90 mg/d<br>OLA: 19.6 mg/d	Schizophrenia (DSM – V)	*'[...] acute worsening for 3−8 weeks, (4) a CGI-I score 4 or more (unchanged or worse) despite 1−3 weeks of OLA treatment for the current acute episode, and (5) at least mild severity of symptoms as evidenced by a PANSS score of 58 or higher.'*	**Sex:**<br>48% males<br><br>**Mean age (years):**<br>47.66<br><br>**Mean duration of illness (years):**<br>13.88
Xiao Shifu 2012 [166]	CERE, n = 55<br>PBO, n = 54<br><br>Add on **Risperidone**	parallel double-blind RCT	8	China	CEREBR: 30ml in 250ml NS IV/d<br>PBO: NA<br><br>RISP (CEREBR): 3.56 mg/d<br>RISP (PBO): 3.54 mg/d	Schizophrenia (DSM – IV)	*'[...] had a score on the PANSS negative symptom scale greater than that on the positive symptom scale. All patients receiving risperidone monotherapy prior to entering the trial and the dosage of risperidone had been stabilized for at least 1 month prior to the trial.'*	**Sex:**<br>74.25% males<br><br>**Mean age (years):**<br>48<br><br>**Mean duration of illness (years):**<br>n.i.

Study ID	Study arms; n of participants	Study design	Study duration (weeks)	Country	Mean or range drug doses (mg/d)	Diagnosis	Definition of TRS	Population characteristics
Zhang 2001a [167]; Zhang 2001b [168]	GinkBil, n = 43<br>PBO, n = 39<br><br>Add on **Haloperidol**	parallel double-blind RCT	12	China	GinkBil: 360 mg/d<br>PBO: NA<br><br>HAL: 16.60 mg/d	Schizophrenia (ICD – 10)	*'Each patient had been treated with at least two kinds of antipsychotics for a half-year or longer, with seven patients who had been previously treated with haloperidol and had shown no response'*	**Sex:**<br>57.30% males<br><br>**Mean age (years):**<br>42.40<br><br>**Mean duration of illness (years):**<br>21.40
Zhang 2006 [169]	ONDAST, n = 58<br>PBO, n = 63<br><br>Add on **Haloperidol**	parallel double-blind RCT	12	China	ONDAST: 7.80 mg/d<br>PBO: NA<br><br>HAL: 11.40 mg/dd	Schizophrenia (DSM – IV)	*' [...] had a documented treatment-resistant status, as defined by the absence of clinically significant improvement after treatment with at least 2 neuroleptics for 6 weeks or longer at a full dose equivalent to 800 mg/d of CPZ; had persistent negative symptoms or significant cognitive dysfunction, as evidenced by a score of at least 20 on the negative symptom subscale or at least 15 on the cognitive subscale of the PANSS; and had an overall score of at least 70 on the PANSS and ≥4 on the CGI-S.*	**Sex:**<br>72.72% males<br><br>**Mean age (years):**<br>39.80<br><br>**Mean duration of illness (years):**<br>16.20
Zhou 1999 [170]	GinkBil, n = 27<br>PBO, n = 27<br><br>Add on **Haloperidol**	parallel double-blind RCT	12	China	GinkBil: 360 mg/d<br>PBO: NA<br><br>HAL (GinkBil): 16.40 mg/d<br>HAL (PBO): 16.60 mg/d	Schizophrenia (ICD – 10)	*'[...] non response to previous 2 antipsychotics [...]'*	**Sex:**<br>55.56% males<br><br>**Mean age (years):**<br>38.40<br><br>**Mean duration of illness (years):**<br>20.85
Zhu 2022 [171]	AMI, n = 40<br>PBO, n = 40<br><br>Add on **Clozapine**	parallel double-blind RCT	12	China	AMI: 771.40 mg/d<br>PBO:NA<br><br>CLOZ: 443.31 mg/d<br>*(completers dose)*	Schizophrenia (DSM – IV)	*'[...] had received at least two antipsychotic agents with different mechanisms of action, at appropriate doses for a sufficient course of treatment, and had recently received a stable dose of clozapine (i.e., at least 400 mg/d or more for at least 6 months) in order to ensure a reasonable response to clozapine monotherapy [...]'*	**Sex:**<br>51.25% males<br><br>**Mean age (years):**<br>47.28<br><br>**Mean duration of illness (years):**<br>23.39

Study ID	Study arms; n of participants	Study design	Study duration (weeks)	Country	Mean or range drug doses (mg/d)	Diagnosis	Definition of TRS	Population characteristics
Zink 2009 [172]	RISP, n = 12<br>ZIP, n = 12<br><br>Add on **Clozapine**	parallel open-label RCT	6	Germany	RISP: 3.82 mg/d<br>ZIP: 134 mg/d<br><br>CLOZ (RISP): 406.80 mg/d<br>CLOZ (ZIP): 361.40 mg/d	Schizophrenia or Schizoaffective Disorder (DSM – IV)	*'Documented treatment failures with at least two antipsychotic agents before being switched to clozapine. 6) Treatment-resistant symptoms of psychosis under clozapine monotherapy with clinical significance […]'*	**Sex:**<br>58.30% males<br><br>**Mean age (years):**<br>34.54<br><br>**Mean duration of illness (years):**<br>11.56
Zoccali 2007 [173]	LAM, n = 30<br>PBO, n = 30<br><br>Add on **Clozapine**	parallel double-blind RCT	24	Italy	LAM: 25 to 200 mg/d<br>PBO: NA<br><br>CLOZ (LAM): 300 mg/d<br>CLOZ (PBO): 335 mg/d	Schizophrenia (DSM – IV)	*'[…] persistent positive and negative symptoms despite an adequate trial of clozapine, were included in this study.'*	**Sex:**<br>54.90% males<br><br>**Mean age (years):**<br>31.32<br><br>**Mean duration of illness (years):**<br>9.85

Abbreviations:
AMI: amisulpride; **AP:** antipsychotic; **ARI:** aripiprazole; **BEZOAET:** benzoate sodium; **b.i.d.:** twice per day; **BPRS:** Brief Psychiatric Rating Scale; **CELECOX:** celecoxib; **CEREBR:** cerebrolysin; **CGI-I:** Clinical Global Impressions – Improvement; **CGI-S:** Clinical Global Impressions – Severity; **CLOZ:** clozapine; **CPZ:** chlorpromazine; **Dcyclos:** D-cycloserine; **DESMOP:** desmopressin; **DONEP:** donepezil; **DSM:** Diagnostic and Statistical Manual for Mental Disorders; **Dser:** D-serine; **DUL:** duloxetine; **FLUO:** fluoxetine; **FLUPHLA:** fluphenazine decanoate; **FLUV:** fluvoxamine; **GAF:** Global Assessment of Functioning; **GinkBil:** Ginkgo biloba; **GLY:** glycine; **HAL:** haloperidol; **ICD:** International Classification of Diseases; **IM:** intravascular; **IMI:** imipramine; **IV:** intravenous infusion; **LAM:** lamotrigine; **mcg/d:** microgram per day; **LI:** lithium; **MAZIN:** mazindol; **mg/d:** milligrams per day; **MEM:** memantine; **MET**: metformin; **MINOC:** minocycline; **MIRT:** mirtazapine; **MODF:** modafinil; **mg/mL:** milligrams per millilitre; **µmol/l:** micromole per litre; **n**: number; **NA:** not applicable; **Nacetyl:** N-acetylcysteine; **N.I.:** no information; **NS:** normal saline; **ONDAST**: ondansetron; **PALMIT:** palmitoylethanolamide; **PANSS:** Positive and Negative Syndrome Scale; **PTX:** pentoxifylline; **OLA:** olanzapine; **OXYT:** oxytocin; **PALIP:** paliperidone; **PBO:** placebo; **PHENYLPROP:** phenylpropanolamine; **PIMOZ:** pimozide; **QUE:** quetiapine; **q2wk:** every two weeks; **RESVER:** resveratrol; **RCT:** randomised controlled trial; **RDC:** Research Diagnostic Criteria; **RISP:** risperidone; **SANS:** Scale for the Assessment of Negative Symptoms; **SAPS:** Scales for the Assessment of Positive Symptoms; **SARC:** sarcosine; **SER:** sertraline; **SERTIND:** sertindole; **SUL:** sulpiride; **TOPIR:** topiramate; **TRIFLUO:** trifluoperazine; **TRS:** treatment-resistance; **VALPRO:** valproate; **VitB6:** vitamin B6; **VitD3:** vitamin D3; **VORT:** vortioxetine; **ZIP:** ziprasidone

Appendix 7. Risk of bias: Summary and traffic plots (Figure S 7.1.; Figure S 7.2.)

The following plot (Figure S 7.1.) demonstrates the risk of bias assessment for the individual domains.

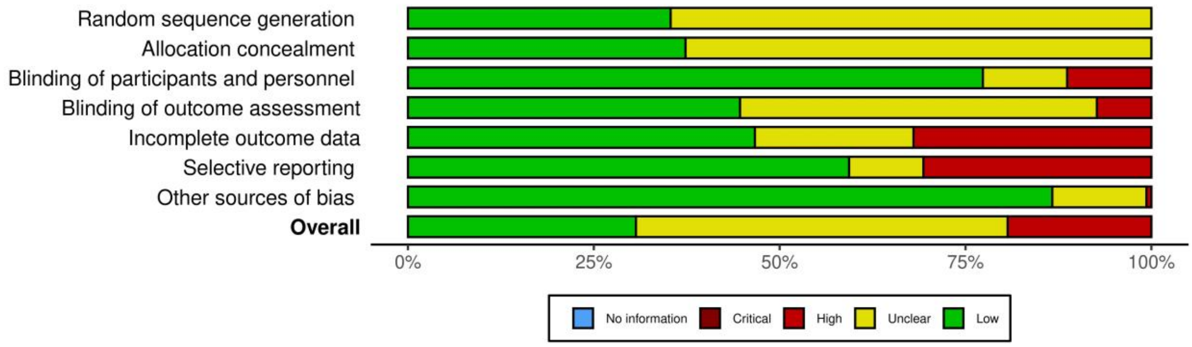

Figure S 7.1. Risk of bias summary plot

The following plot (Figure S 7.2.) demonstrates the risk of bias assessment per individual study.

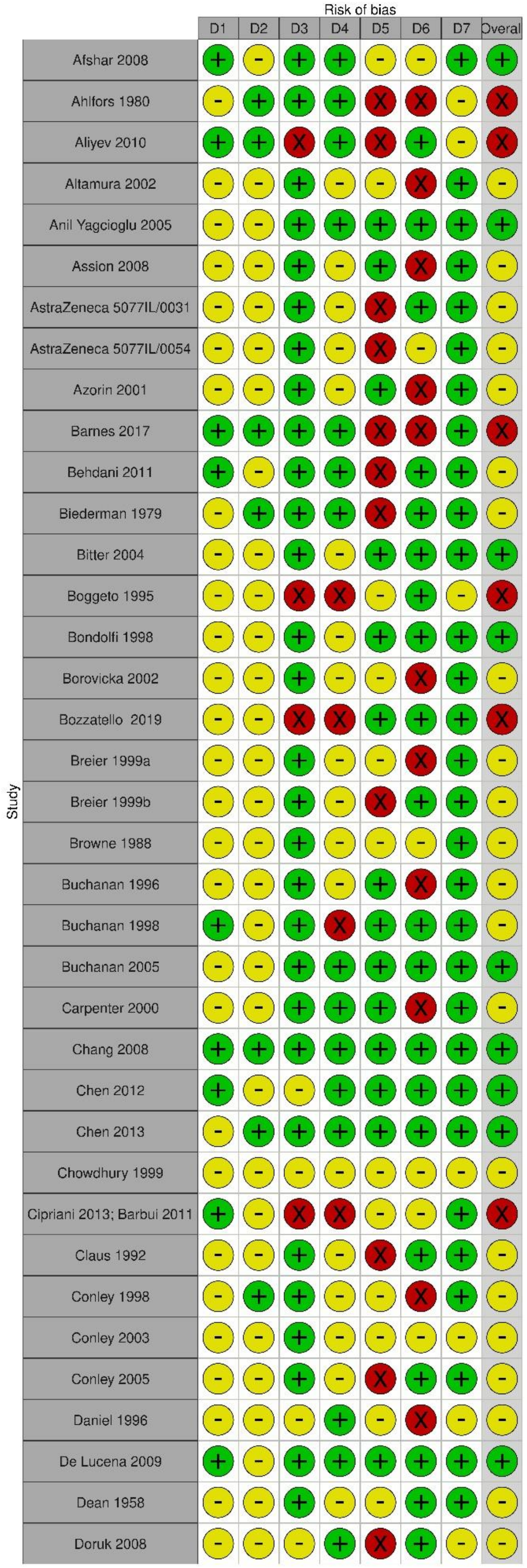

Cont …

Figure S 7.2. Risk of bias assessment for the individual studies (1 of 4)

Risk of bias

Study	D1	D2	D3	D4	D5	D6	D7	Overall
Emsley 2000	-	-	-	+	-	+	+	-
Evins 2000	-	-	+	-	X	+	-	-
Fan 2013	-	-	+	-	-	X	+	-
Fleischhacker 2010	+	+	+	-	X	+	+	-
Freudenreich 2007	-	+	+	-	-	X	-	-
Freudenreich 2009	-	+	+	-	+	+	+	+
Friedman 2011	-	-	+	+	-	+	+	+
Geller 2005	-	-	+	+	X	X	+	X
Genc 2007	-	-	X	+	X	+	+	X
Goff 1999	-	-	+	-	X	X	+	X
Gunduz-Bruce 2013	-	+	+	-	+	+	+	+
Hall 1968	-	-	+	-	-	X	-	-
Hatta 2012	+	+	X	+	+	+	+	-
Hatta 2014a	+	+	X	+	+	+	+	-
Hatta 2014b	+	+	X	+	+	+	+	-
Herken 1999	-	-	-	-	-	-	+	-
Heres 2022	+	+	+	+	X	X	+	X
Honer 2006	+	+	+	+	+	+	+	+
Hong 1997	+	-	+	-	+	+	+	+
Honigfeld 1984	-	-	+	-	X	+	+	-
Hosseini 2014	+	+	+	+	+	+	+	+
Howard 1974	-	-	+	-	+	X	-	-
Ibrahim 2019	-	+	+	+	-	X	+	-
Josiassen 2005	-	-	+	+	+	X	+	-
Kahn 2018	+	+	+	-	+	+	+	+
Kane 1988	-	+	+	-	-	X	+	-
Kane 2001	+	+	+	-	X	+	+	-
Kane 2006	-	-	+	-	+	+	+	+
Kane 2007	-	-	+	-	X	+	+	-
Kane 2011	-	+	+	-	X	+	+	-
Kelly 2015	-	-	+	+	+	+	+	+
Kinon 1993	-	-	+	-	-	-	+	-
Kinon 2010	-	-	+	-	X	X	+	X
Kluge 2007	-	-	+	-	+	+	+	+
Kotler 2004	-	-	X	X	-	+	+	X
Krivoy 2017	+	+	+	+	+	-	+	+
Kumar 2017	+	-	X	X	+	-	+	X

Cont …

Figure S 7.2. Risk of bias assessment for the individual studies (2 of 4)

Risk of bias

Study	D1	D2	D3	D4	D5	D6	D7	Overall
Kumra 1996	+	+	+	-	+	+	+	+
Kumra 2007	-	-	+	+	+	X	+	-
Lal 2006	+	+	+	+	+	+	+	+
Lane 2006	-	+	+	+	+	+	+	+
Lang 2023	+	+	-	X	+	+	+	-
Lee 2011	-	-	+	-	-	+	+	-
Lin 2013	-	-	X	+	+	X	+	X
Lin 2018	-	+	+	+	-	+	+	+
Lu 2004	-	-	X	X	+	X	-	X
Lu 2018	+	+	+	+	+	+	+	+
Marjerrison 1964	-	-	+	-	-	+	+	-
Mayabhate 2014	+	+	+	+	+	+	+	+
Mc Gurk 2005; Schooler 2016	-	-	+	+	+	X	+	-
McCreadie 1977	-	-	+	-	-	+	+	-
McEvoy 2006	-	-	+	+	+	+	+	+
Meltzer 2008	+	+	+	-	X	+	+	-
Mercer 1997	-	-	+	+	X	X	+	X
Meyer-Lindenberg 1997	-	-	+	-	X	X	+	X
Mico 2011	+	+	-	-	+	+	+	+
Moazen-Zadeh 2020	+	+	+	+	-	+	+	+
Modabbernia 2013	+	+	+	+	+	+	+	+
Moresco 2004	-	-	+	-	X	X	+	X
Mossaheb 2006	-	-	-	-	X	-	-	-
Muscatello 2010	+	+	+	-	X	X	+	X
Muscatello 2011	+	+	+	-	X	X	+	X
Muscatello 2014	+	+	+	+	+	+	+	+
Naber 2005	-	-	+	-	+	+	+	+
Neill 2022	+	+	+	+	+	+	+	+
Nielsen 2012	+	+	-	-	+	+	+	+
Potkin 1999	-	+	+	+	X	+	+	-
Repo-Tiihonen 2012	-	+	+	+	X	+	+	-
Rosenheck 1997	-	-	+	-	-	X	-	-
Sacchetti 2009	-	-	+	-	X	+	+	-
Salehi 2022	+	+	+	+	+	X	+	-
Samadi 2015	-	-	+	+	X	+	+	-
Samadi 2017	+	+	+	-	+	+	+	+
Samaei 2020	+	+	+	+	+	X	+	-
Schiele 1961, 06602	-	-	+	+	+	+	-	+

Cont …

Figure S 7.2. Risk of bias assessment for the individual studies (3 of 4)

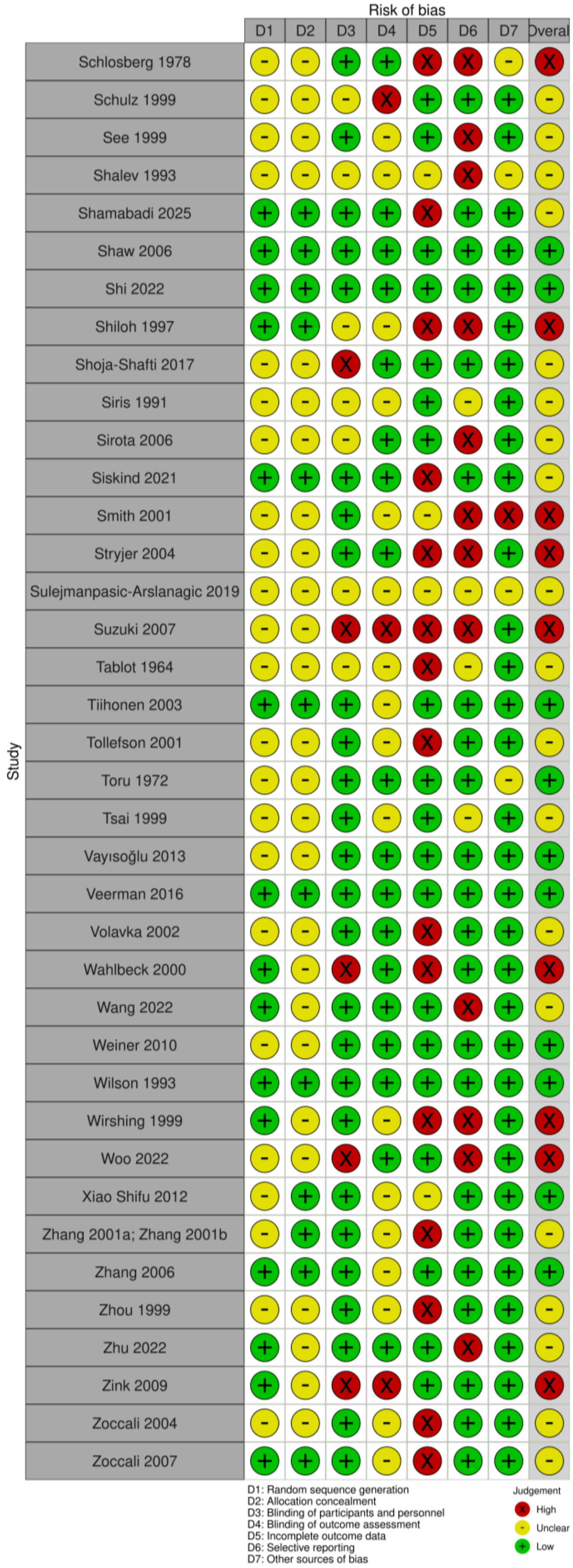

Figure S 7.2. Risk of bias assessment for the individual studies (4 of 4)

Appendix 8: Statistical methods in detail

We fitted frequentist random-effects network meta-analyses with Inverse Variance (IV) method, assuming a common random-effects SD (τ), for all comparisons across the network. We used a random-effects model to account for potential methodological variations across the trials, such as differences in participants, interventions, or outcome definitions. We used the standardized mean difference (SMD) due to different measurements when the outcome was continuous and Odds Ratio (OR) when the outcome was binary. Due to the rarity of the events, we used various common-effect models for dichotomous (i.e., binary) outcomes, including Mantel-Haenszel (MH), non-central hypergeometric distribution with Breslow approximation, and logistic regression with Firth's correction, in addition to the random-effects model[174]. In the two common-effect models we add to the single zero-cell counts the value of 0.5 (continuity correction) when necessary and exclude the double zero-cell count studies[175]. A logistic regression model works with zero-cell counts, so it does not require any correction.

Clozapine is the reference treatment in all forest plots. Our ranking of interventions was based on P-scores as described by Rücker et al.[176]. We display prediction intervals which capture the variability of the data (heterogeneity) and provide a predicted range for the true effect size in a new study. Forest plots depict the information we described in the previous lines. Additional to NMA forest plots, we display the results with a league table where the lower triangle depicts the results of NMA while the upper triangle the results of the pairwise meta-analysis when possible.

We assessed network consistency by applying both local and global methods. We employed the node-splitting approach by Dias et al.[177] for local inconsistency and the design-by-treatment model as described by Higgins et al.[178] for global consistency. Additionally, we present the net-heat plot for detecting[179] close loops of inconsistency.

We performed sensitivity analysis and subgroup analysis in agreement with the protocol of the study. All analyses were done in R version 4.3.3[180]. We fitted our models in R using netmeta[181] version 2.9.0 and create our network plot with multinma[182] version 0.7.1.

We used the Confidence in Network Meta-Analysis (CINeMA) framework to assess the credibility of findings from each network meta-analysis, grading the confidence in each treatment comparison as high, moderate, low or very low; further details on the implementation of CINeMA can be found in the Appendix 14[183].

Appendix 9. Transitivity assessment

Prior to the statistical analysis, we evaluated whether the RCTs included in the NMA were, on average, similar regarding the characteristics that could potentially influence the treatment effects, ensuring the plausibility of the transitivity assumption. Rather than direct comparisons, indirect comparisons are not protected by randomisation and may be influenced by differences between the RCTs. In our analysis, we identified the following parameters as potential confounders based on findings from a previous study[184]: (1) baseline severity of symptoms of treatment resistance schizophrenia, (2) publication year, (3) mean age of participants, and (4) percentage of male participants. To assess the plausibility of the transitivity assumption, we compared the distribution of these potential effect modifiers across studies grouped by comparison.

9.1. Baseline severity of treatment resistance schizophrenia

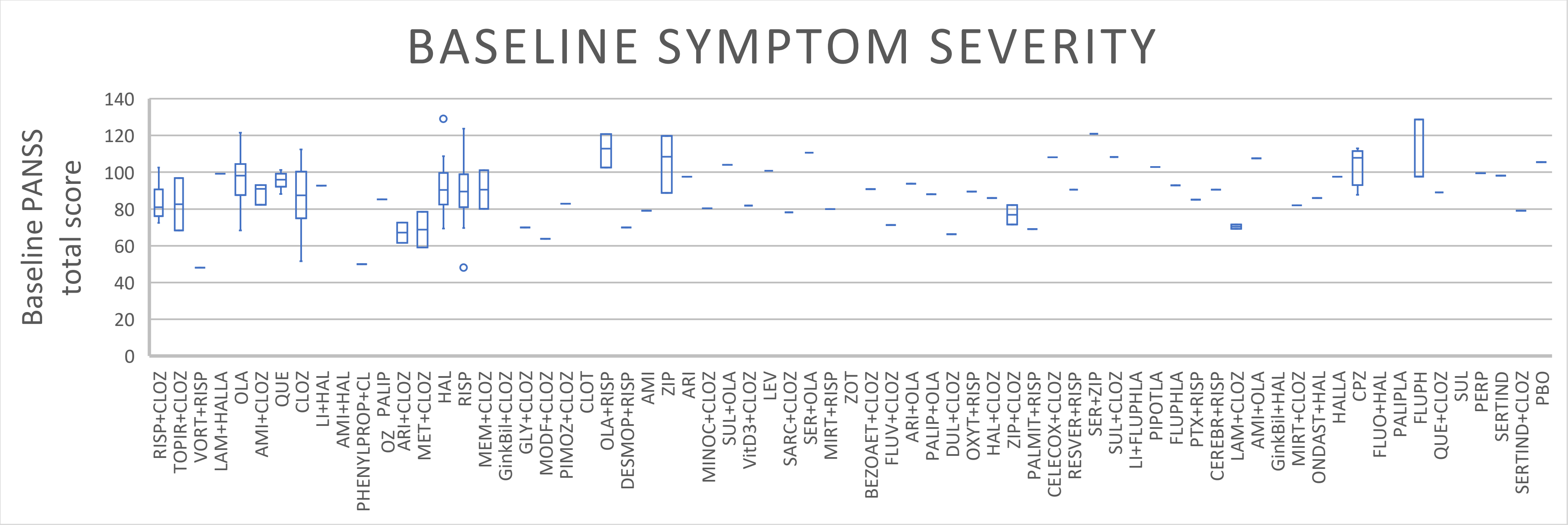

Figure S 9.1. Boxplot for distribution of baseline severity across comparisons
AMI: amisulpride; ARI: aripiprazole; BEZOAET: benzoate sodium; CELECOX: celecoxib; CEREBR: cerebrolysin; CLOZ: clozapine; CLOT: clotiapine; CPZ: chlorpromazine;; DESMOP: desmopressin; DUL: duloxetine; FLUO: fluoxetine; FLUPH: fluphenazine; FLUPHLA: Fluphenazine decanoate; FLUV: Fluvoxamine; GinkBil: Ginkgo biloba; GLY: glycine; HAL: haloperidol; HALLA: haloperidol decanoate; LAM: lamotrigine; LEV: levomepromazine; LI: lithium; MEM: memantine; MET: metformin; MINOC: minocycline; MIRT: mirtazapine; MODF: modafinil; ONDAST: ondansetron; OXYT: oxytocin; PANSS: Positive and Negative Syndrome Scale; PIPOTLA: pipotiazine long-acting; OLA: olanzapine; OXYT: oxytocin; PALIP: paliperidone; PALMIT: palmitoylethanolamide; PBO: placebo; PERP: perphenazine;

PHENPROP: phenylpropanolamine; PIMOZ: pimozide; PTX: pentoxifylline; QUE: quetiapine; RESVER: resveratrol; RISP: risperidone; SARC: sarcosine; SER: sertraline; SERTIND: sertindole; SUL: sulpiride; TOPIR: topiramate; VitB6: vitamin B6; VitD3: vitamin D3; VORT: vortioxetine; ZIP: ziprasidone; ZOT: zotepine.

9.2. Year of publication

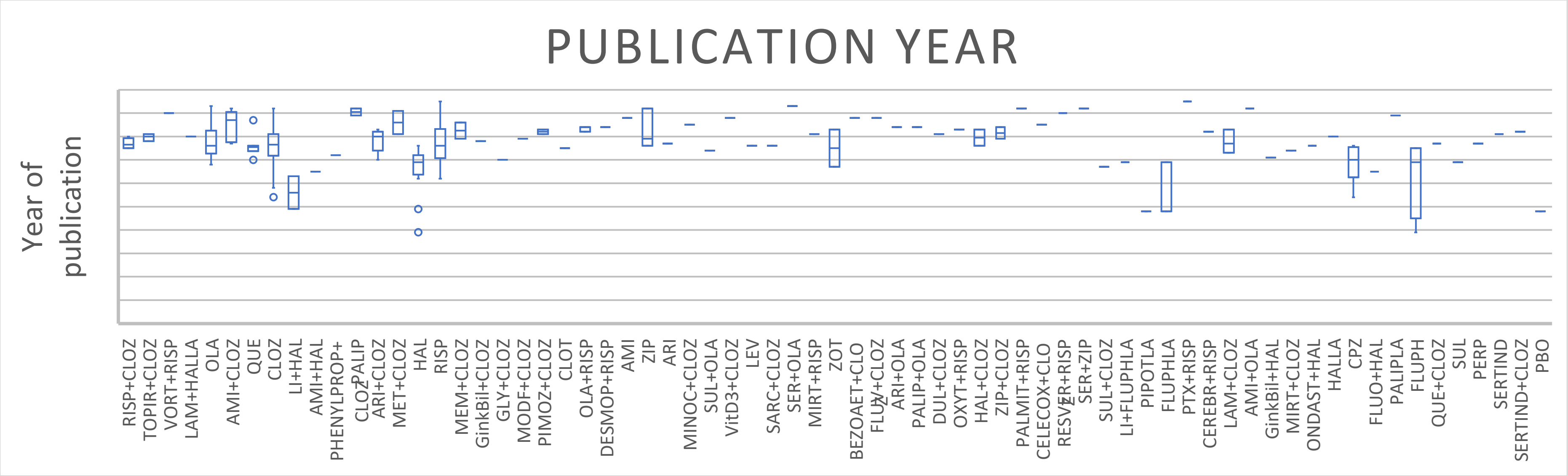

Figure S 9.2. Boxplot for distribution of publication year across comparisons

AMI: amisulpride; ARI: aripiprazole; BEZOAET: benzoate sodium; CELECOX: celecoxib; CEREBR: cerebrolysin; CLOZ: clozapine; CLOT: clotiapine; CPZ: chlorpromazine;; DESMOP: desmopressin; DUL: duloxetine; FLUO: fluoxetine; FLUPH: fluphenazine; FLUPHLA: Fluphenazine decanoate; FLUV: Fluvoxamine; GinkBil: Ginkgo biloba; GLY: glycine; HAL: haloperidol; HALLA: haloperidol decanoate; LAM: lamotrigine; LEV: levomepromazine; LI: lithium; MEM: memantine; MET: metformin; MINOC: minocycline; MIRT: mirtazapine; MODF: modafinil; ONDAST: ondansetron; OXYT: oxytocin; PANSS: Positive and Negative Syndrome Scale; PIPOTLA: pipotiazine long-acting; OLA: olanzapine; OXYT: oxytocin; PALIP: paliperidone; PALMIT: palmitoylethanolamide; PBO: placebo; PERP: perphenazine;PHENPROP: phenylpropanolamine; PIMOZ: pimozide; PTX: pentoxifylline; QUE: quetiapine; RESVER: resveratrol; RISP: risperidone; SARC: sarcosine; SER: sertraline; SERTIND: sertindole; SUL: sulpiride; TOPIR: topiramate; VitB6: vitamin B6; VitD3: vitamin D3; VORT: vortioxetine; ZIP: ziprasidone; ZOT: zotepine.

9.3. Mean age of participants

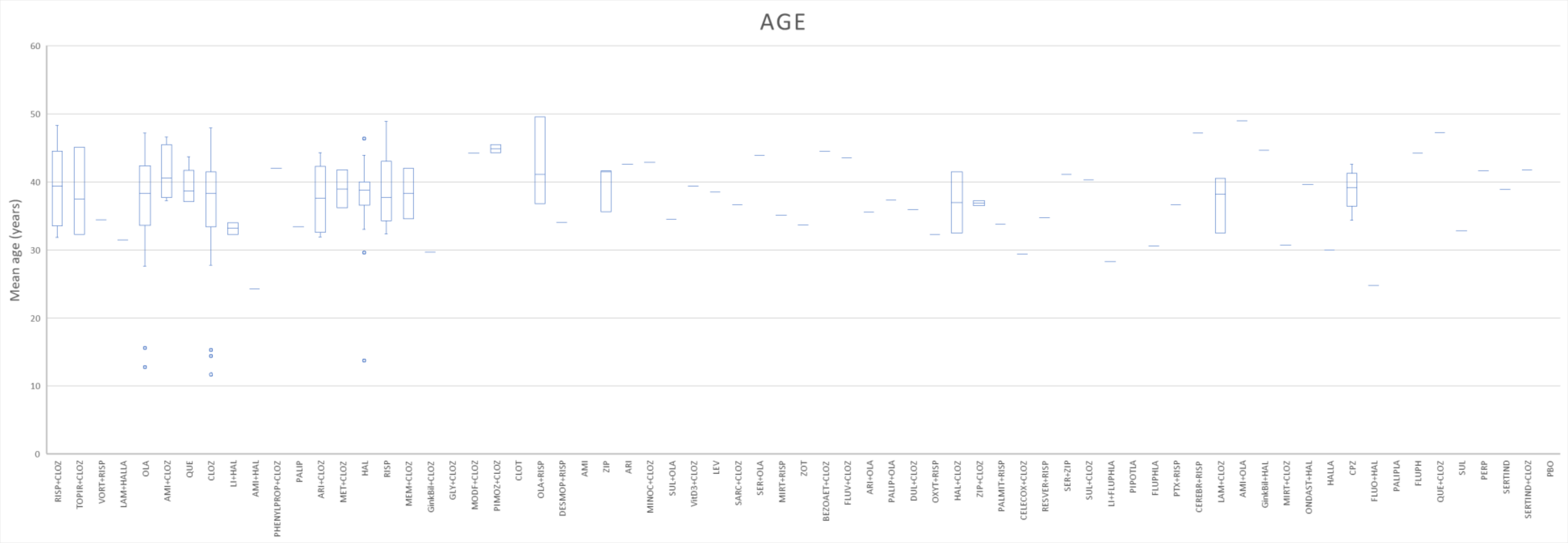

Figure S 9.3. Boxplot for distribution of mean age of the participants across comparisons
AMI: amisulpride; ARI: aripiprazole; BEZOAET: benzoate sodium; CELECOX: celecoxib; CEREBR: cerebrolysin; CLOZ: clozapine; CLOT: clotiapine; CPZ: chlorpromazine;; DESMOP: desmopressin; DUL: duloxetine; FLUO: fluoxetine; FLUPH: fluphenazine; FLUPHLA: Fluphenazine decanoate; FLUV: Fluvoxamine; GinkBil: Ginkgo biloba; GLY: glycine; HAL: haloperidol; HALLA: haloperidol decanoate; LAM: lamotrigine; LEV: levomepromazine; LI: lithium; MEM: memantine; MET: metformin; MINOC: minocycline; MIRT: mirtazapine; MODF: modafinil; ONDAST: ondansetron; OXYT: oxytocin; PANSS: Positive and Negative Syndrome Scale; PIPOTLA: pipotiazine long-acting; OLA: olanzapine; OXYT: oxytocin; PALIP: paliperidone; PALMIT: palmitoylethanolamide; PBO: placebo; PERP: perphenazine; PHENPROP: phenylpropanolamine; PIMOZ: pimozide; PTX: pentoxifylline; QUE: quetiapine; RESVER: resveratrol; RISP: risperidone; SARC: sarcosine; SER: sertraline; SERTIND: sertindole; SUL: sulpiride; TOPIR: topiramate; VitB6: vitamin B6; VitD3: vitamin D3; VORT: vortioxetine; ZIP: ziprasidone; ZOT: zotepine.

9.4. Percentage of male participants

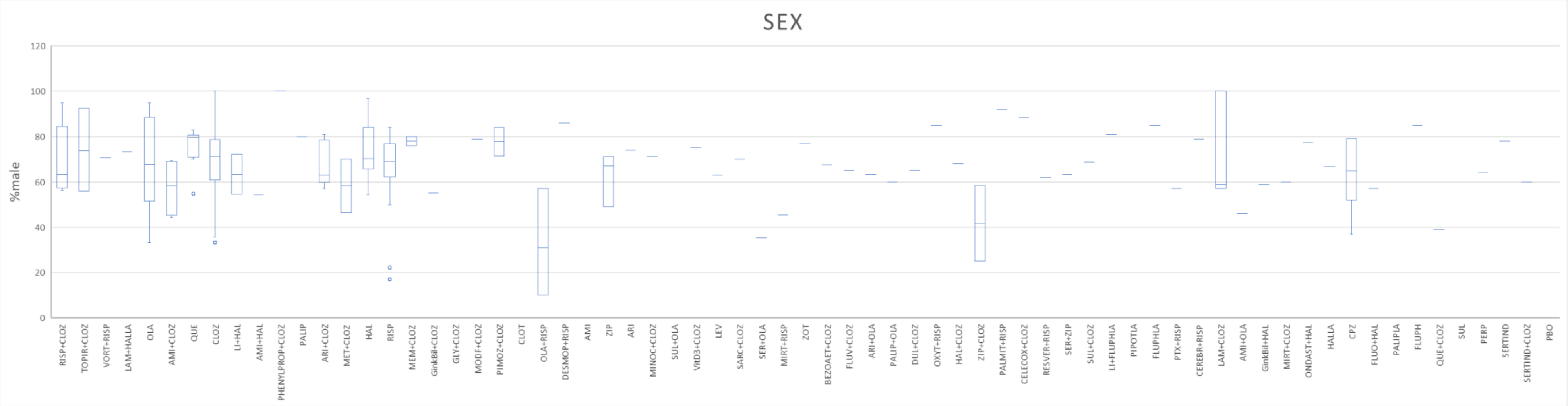

Figure S 9.4. Boxplot for distribution of percentage of male participants across comparisons

AMI: amisulpride; ARI: aripiprazole; BEZOAET: benzoate sodium; CELECOX: celecoxib; CEREBR: cerebrolysin; CLOZ: clozapine; CLOT: clotiapine; CPZ: chlorpromazine;; DESMOP: desmopressin; DUL: duloxetine; FLUO: fluoxetine; FLUPH: fluphenazine; FLUPHLA: Fluphenazine decanoate; FLUV: Fluvoxamine; GinkBil: Ginkgo biloba; GLY: glycine; HAL: haloperidol; HALLA: haloperidol decanoate; LAM: lamotrigine; LEV: levomepromazine; LI: lithium; MEM: memantine; MET: metformin; MINOC: minocycline; MIRT: mirtazapine; MODF: modafinil; ONDAST: ondansetron; OXYT: oxytocin; PANSS: Positive and Negative Syndrome Scale; PIPOTLA: pipotiazine long-acting; OLA: olanzapine; OXYT: oxytocin; PALIP: paliperidone; PALMIT: palmitoylethanolamide; PBO: placebo; PERP: perphenazine; PHENPROP: phenylpropanolamine; PIMOZ: pimozide; PTX: pentoxifylline; QUE: quetiapine; RESVER: resveratrol; RISP: risperidone; SARC: sarcosine; SER: sertraline; SERTIND: sertindole; SUL: sulpiride; TOPIR: topiramate; VitB6: vitamin B6; VitD3: vitamin D3; VORT: vortioxetine; ZIP: ziprasidone; ZOT: zotepine.

Appendix 10. Complete list of graphs and plots for all outcomes

10.1. Overall change in symptoms (Figure S 10.1a)

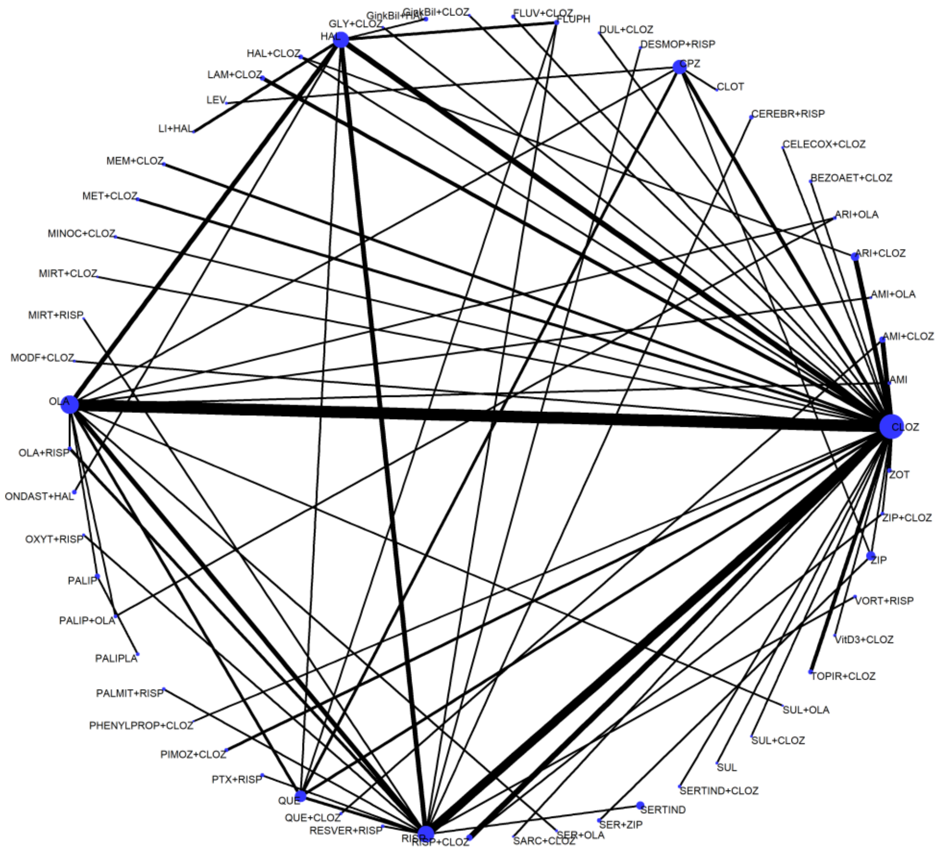

Figure S 10.1a. Network plot for the secondary outcome: Overall change in symptoms

The size of the nodes corresponds to the number of participants assigned to each treatment. Treatments with direct comparisons are linked with a line; its thickness corresponds to the number of trials evaluating the comparison.

AMI: amisulpride; ARI: aripiprazole; BEZOAET: benzoate sodium; CELECOX: celecoxib; CEREBR: cerebrolysin; CLOZ: clozapine; CLOT: clotiapine; CPZ: chlorpromazine; DESMOP: desmopressin; DUL: duloxetine; FLUO: fluoxetine; FLUPHLA: fluphenazine decanoate; FLUV: Fluvoxamine; GinkBil: Ginkgo biloba; GLY: glycine; HAL: haloperidol; LAM: lamotrigine; LEV: levomepromazine; LI: lithium; MEM: memantine; MET: metformin; MINOC: minocycline; MIRT: mirtazapine; MODF: modafinil; ONDAST: ondansetron; OLA: olanzapine; OXYT: Oxytocin; PALIP: paliperidone; PALMIT: palmitoylethanolamide; PBO: placebo; PHENYLPROP: phenylpropanolamine; PIMOZ: pimozide; PTX: pentoxifylline; QUE: quetiapine; RESVER: resveratrol; RISP: risperidone; SARC: sarcosine; SER: sertraline; SERTIND: sertindole; SUL: sulpiride; TOPIR: topiramate; VitB6: vitamin B6; VitD3: vitamin D3; VORT: vortioxetine; ZIP: ziprasidone.

10.2.Positive symptoms (Figure S 10.2a; Figure S 10.2b; Figure S 10.2c)

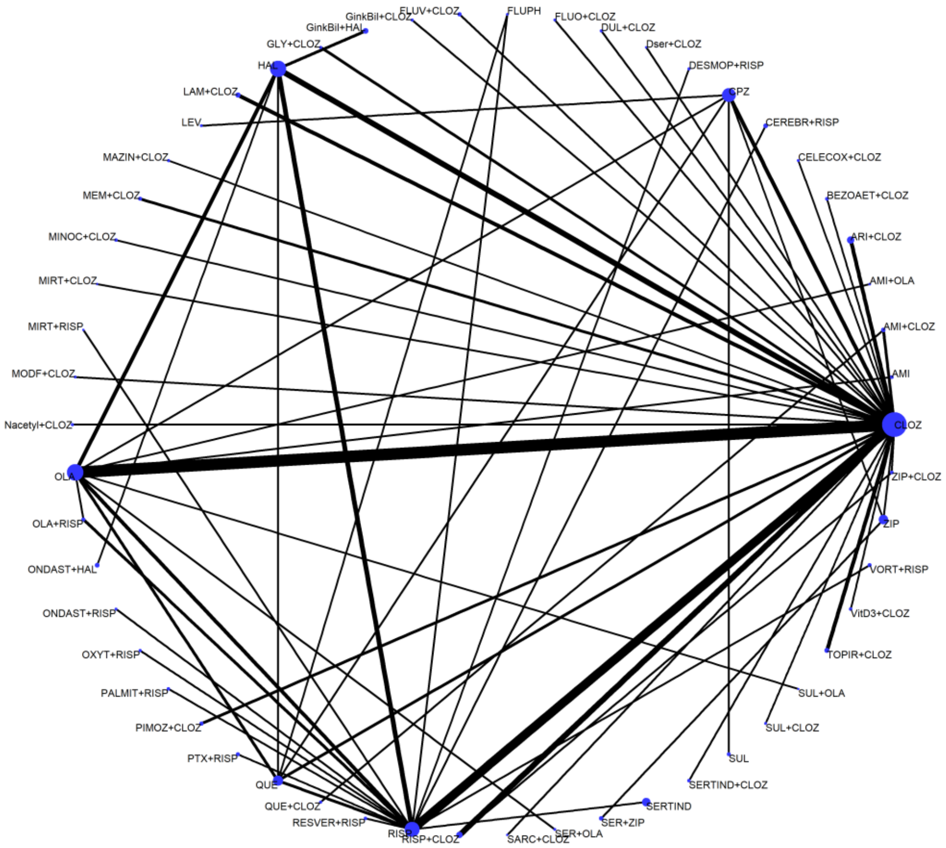

Figure S 10.2a. Network plot for the secondary outcome: Positive symptoms
AMI: amisulpride; ARI: aripiprazole; BEZOAET: benzoate sodium; CELECOX: celecoxib; CEREBR: cerebrolysin; CLOZ: clozapine; CPZ: chlorpromazine; Dcyclos: D-cycloserine; DESMOP: desmopressin; Dser: D-serine; DUL: duloxetine; FLUO: fluoxetine; FLUPH: fluphenazine; FLUPHLA: fluphenazine decanoate; FLUV: fluvoxamine; GinkBil: Ginkgo biloba; GLY: glycine; HAL: haloperidol; LAM: lamotrigine; LEV: levomepromazine; MAZIN: mazindol; MEM: memantine; MINOC: minocycline; MIRT: mirtazapine; MODF: modafinil; Nacetyl: N-acetylcysteine; OLA: olanzapine; ONDAST: ondansetron; OXYT: oxytocin; PALMIT: palmitoylethanolamide; PIMOZ: pimozide; PTX: pentoxifylline; QUE: quetiapine; RESVER: resveratrol; RISP: risperidone; SARC: sarcosine; SER: sertraline; SERTIND: sertindole; SUL: sulpiride; TOPIR: topiramate; VitD3: vitamin D3; VORT: vortioxetine; ZIP: ziprasidone.

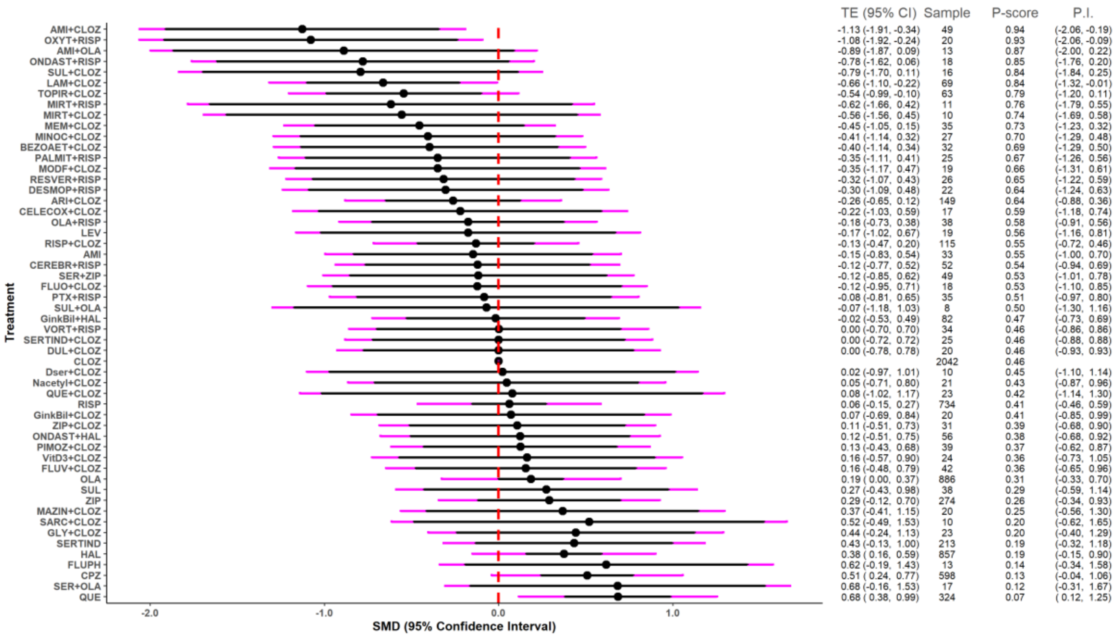

Figure S 10.2b. Forest plot with prediction intervals for the secondary outcome: Positive symptoms
Reference comparator: clozapine. AMI: amisulpride; ARI: aripiprazole; BEZOAET: benzoate sodium; CELECOX: celecoxib; CEREBR: cerebrolysin; CLOZ: clozapine; CPZ: chlorpromazine; Dcyclos: D-cycloserine; DESMOP: desmopressin; Dser: D-serine; DUL: duloxetine; FLUO: fluoxetine; FLUPH: fluphenazine; FLUPHLA: fluphenazine decanoate; FLUV: fluvoxamine; GinkBil: Ginkgo biloba; GLY: glycine; HAL: haloperidol; LAM: lamotrigine; LEV: levomepromazine; MAZIN: mazindol; MEM: memantine; MINOC: minocycline; MIRT: mirtazapine; MODF: modafinil; Nacetyl: N-acetylcysteine; OLA: olanzapine; ONDAST: ondansetron; OXYT: oxytocin; PALMIT: palmitoylethanolamide; PIMOZ: pimozide; PTX: pentoxifylline; QUE: quetiapine; RESVER: resveratrol; RISP: risperidone; SARC: sarcosine; SER: sertraline; SERTIND: sertindole; SUL: sulpiride; TOPIR: topiramate; VitD3: vitamin D3; VORT: vortioxetine; ZIP: ziprasidone.

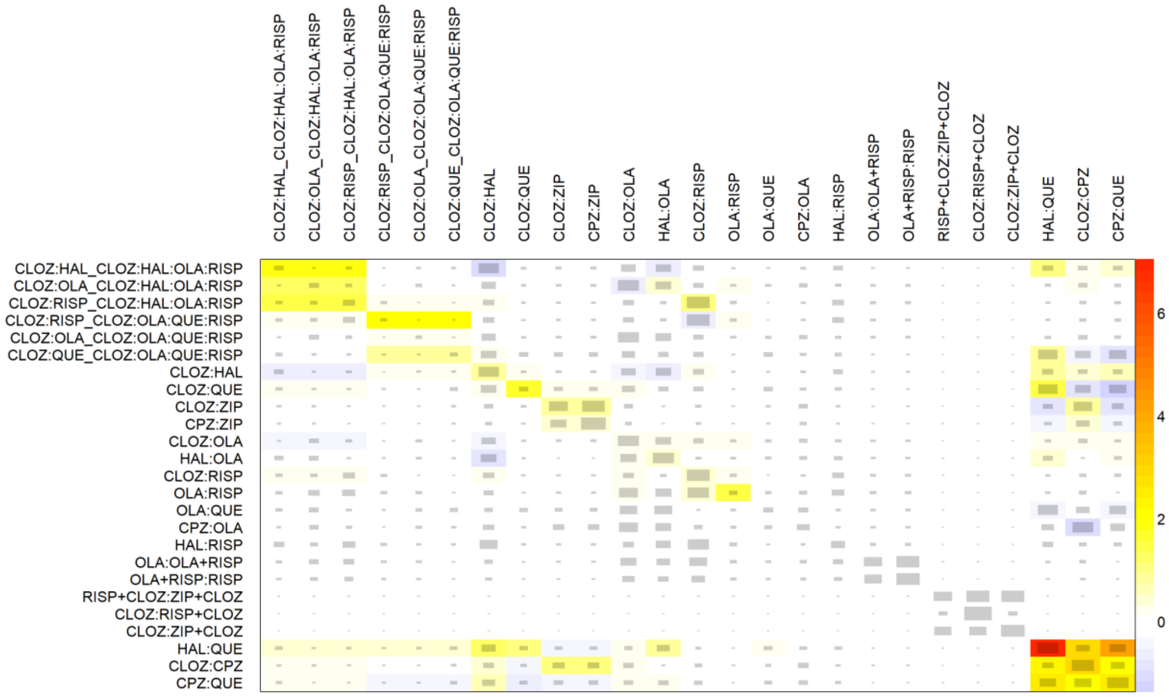

Figure S 10.2c. Net heat plot for the secondary outcome: Positive symptoms
CLOZ: clozapine; CPZ: chlorpromazine; HAL: haloperidol; OLA: olanzapine; QUE: quetiapine; RISP: risperidone; ZIP: ziprasidone.

10.3. Negative symptoms (Figure S 10.3a; Figure S 10.3b; Figure S 10.3c)

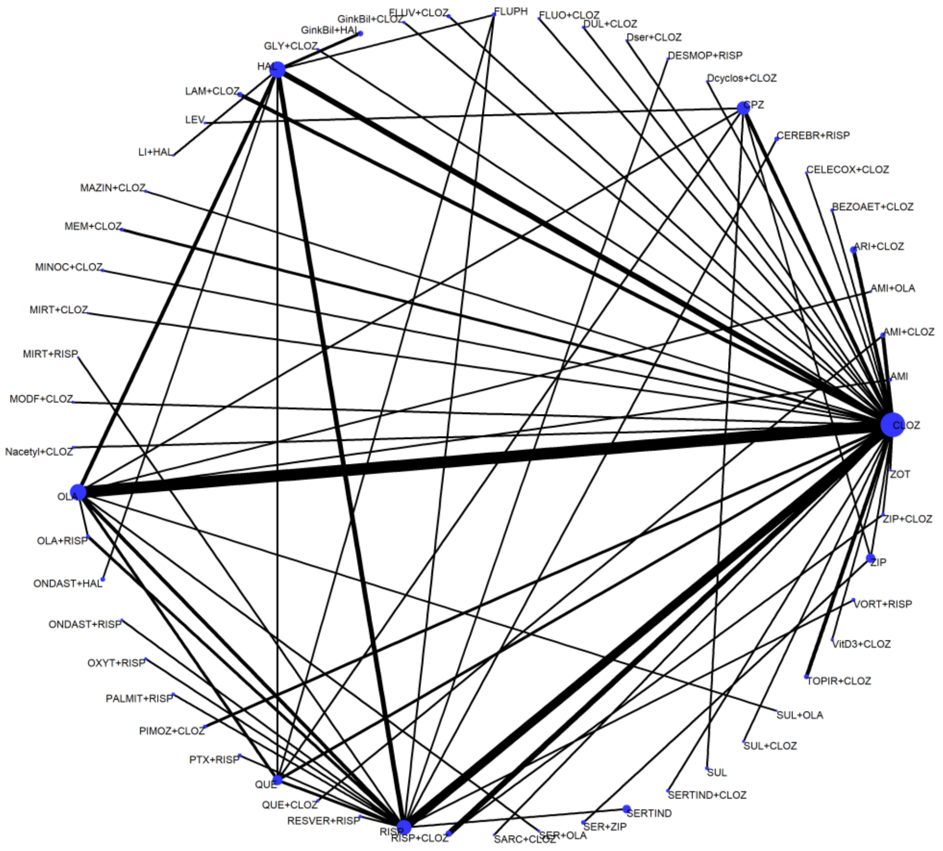

Figure S 10.3a. Network plot for the secondary outcome: Negative symptoms
AMI: amisulpride; ARI: aripiprazole; BEZOAET: benzoate sodium; CELECOX: celecoxib; CEREBR: cerebrolysin; CLOZ: clozapine; CPZ: chlorpromazine; Dcyclos: D-cycloserine; DESMOP: desmopressin; Dser: D-serine; DUL: duloxetine; FLUO: fluoxetine; FLUPH: fluphenazine; FLUPHLA: fluphenazine decanoate; FLUV: fluvoxamine; GinkBil: Ginkgo biloba; GLY: glycine; HAL: haloperidol; LAM: lamotrigine; LEV: levomepromazine; LI: lithium MAZIN: mazindol; MEM: memantine; MINOC: minocycline; MIRT: mirtazapine; MODF: modafinil; Nacetyl: N-acetylcysteine; OLA: olanzapine; ONDAST: ondansetron; OXYT: oxytocin; PALMIT: palmitoylethanolamide; PIMOZ: pimozide; PTX: pentoxifylline; QUE: quetiapine; RESVER: resveratrol; RISP: risperidone; SARC: sarcosine; SER: sertraline; SERTIND: sertindole; SUL: sulpiride; TOPIR: topiramate; VitD3: vitamin D3; VORT: vortioxetine; ZIP: ziprasidone; ZOT: zotepine.

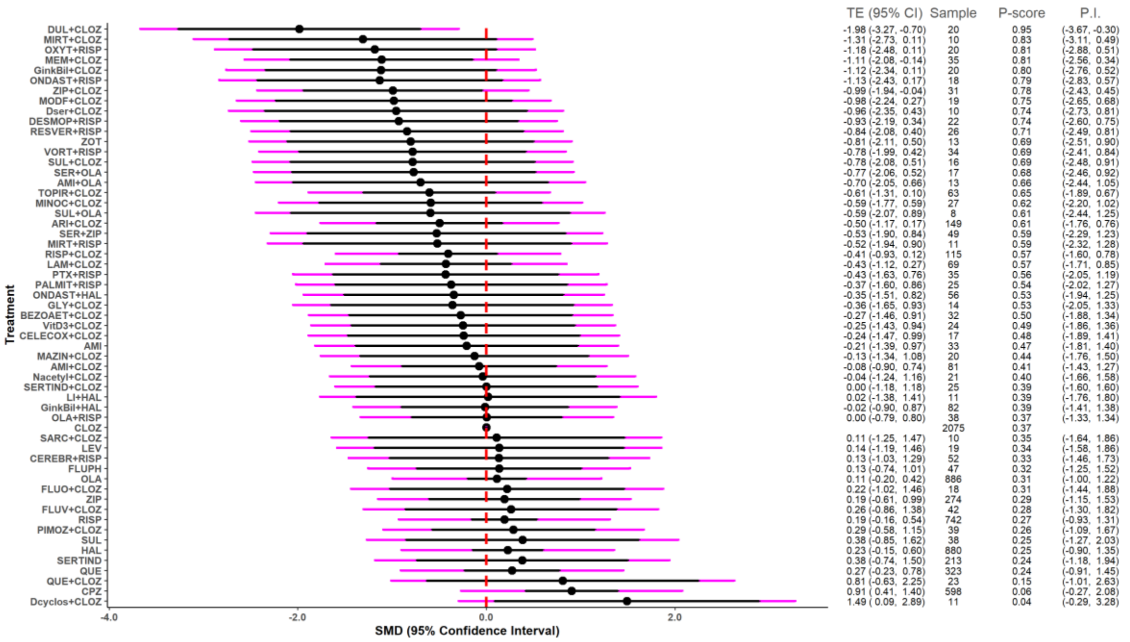

Treatment	TE (95% CI)	Sample	P-score	P.I.
DUL+CLOZ	-1.98 (-3.27, -0.70)	20	0.95	(-3.67, -0.30)
MIRT+CLOZ	-1.31 (-2.73, 0.11)	10	0.83	(-3.11, 0.49)
OXYT+RISP	-1.18 (-2.48, 0.11)	20	0.81	(-2.88, 0.51)
MEM+CLOZ	-1.11 (-2.08, -0.14)	35	0.81	(-2.56, 0.34)
GinkBil+CLOZ	-1.12 (-2.34, 0.11)	20	0.80	(-2.76, 0.52)
ONDAST+RISP	-1.13 (-2.43, 0.17)	18	0.79	(-2.83, 0.57)
ZIP+CLOZ	-0.99 (-1.94, -0.04)	31	0.78	(-2.43, 0.45)
MODF+CLOZ	-0.98 (-2.24, 0.27)	19	0.75	(-2.65, 0.68)
Dser+CLOZ	-0.96 (-2.35, 0.43)	10	0.74	(-2.73, 0.81)
DESMOP+RISP	-0.93 (-2.19, 0.34)	22	0.74	(-2.60, 0.75)
RESVER+RISP	-0.84 (-2.08, 0.40)	26	0.71	(-2.49, 0.81)
ZOT	-0.81 (-2.11, 0.50)	13	0.69	(-2.51, 0.90)
VORT+RISP	-0.78 (-1.99, 0.42)	34	0.69	(-2.41, 0.84)
SUL+CLOZ	-0.78 (-2.08, 0.51)	16	0.69	(-2.48, 0.91)
SER+OLA	-0.77 (-2.06, 0.52)	17	0.68	(-2.46, 0.92)
AMI+OLA	-0.70 (-2.05, 0.66)	13	0.66	(-2.44, 1.05)
TOPIR+CLOZ	-0.61 (-1.31, 0.10)	63	0.65	(-1.89, 0.67)
MINOC+CLOZ	-0.59 (-1.77, 0.59)	27	0.62	(-2.20, 1.02)
SUL+OLA	-0.59 (-2.07, 0.89)	8	0.61	(-2.44, 1.25)
ARI+CLOZ	-0.50 (-1.17, 0.17)	149	0.61	(-1.76, 0.76)
SER+ZIP	-0.53 (-1.90, 0.84)	49	0.59	(-2.29, 1.23)
MIRT+RISP	-0.52 (-1.94, 0.90)	11	0.59	(-2.32, 1.28)
RISP+CLOZ	-0.41 (-0.93, 0.12)	115	0.57	(-1.60, 0.78)
LAM+CLOZ	-0.43 (-1.12, 0.27)	69	0.57	(-1.71, 0.85)
PTX+RISP	-0.43 (-1.63, 0.76)	35	0.56	(-2.05, 1.19)
PALMIT+RISP	-0.37 (-1.60, 0.86)	25	0.54	(-2.02, 1.27)
ONDAST+HAL	-0.35 (-1.51, 0.82)	56	0.53	(-1.94, 1.25)
GLY+CLOZ	-0.36 (-1.65, 0.93)	14	0.53	(-2.05, 1.33)
BEZOAET+CLOZ	-0.27 (-1.46, 0.91)	32	0.50	(-1.88, 1.34)
VitD3+CLOZ	-0.25 (-1.43, 0.94)	24	0.49	(-1.86, 1.36)
CELECOX+CLOZ	-0.24 (-1.47, 0.99)	17	0.48	(-1.89, 1.41)
AMI	-0.21 (-1.39, 0.97)	33	0.47	(-1.81, 1.40)
MAZIN+CLOZ	-0.13 (-1.34, 1.08)	20	0.44	(-1.76, 1.50)
AMI+CLOZ	-0.08 (-0.90, 0.74)	81	0.41	(-1.43, 1.27)
Nacetyl+CLOZ	-0.04 (-1.24, 1.16)	21	0.40	(-1.66, 1.58)
SERTIND+CLOZ	0.00 (-1.18, 1.18)	25	0.39	(-1.60, 1.60)
LI+HAL	0.02 (-1.38, 1.41)	11	0.39	(-1.76, 1.80)
GinkBil+HAL	-0.02 (-0.90, 0.87)	82	0.39	(-1.41, 1.38)
OLA+RISP	0.00 (-0.79, 0.80)	38	0.37	(-1.33, 1.34)
CLOZ		2075	0.37	
SARC+CLOZ	0.11 (-1.25, 1.47)	10	0.35	(-1.64, 1.86)
LEV	0.14 (-1.19, 1.46)	19	0.34	(-1.58, 1.86)
CEREBR+RISP	0.13 (-1.03, 1.29)	52	0.33	(-1.46, 1.73)
FLUPH	0.13 (-0.74, 1.01)	47	0.32	(-1.25, 1.52)
OLA	0.11 (-0.20, 0.42)	886	0.31	(-1.00, 1.22)
FLUO+CLOZ	0.22 (-1.02, 1.46)	18	0.31	(-1.44, 1.88)
ZIP	0.19 (-0.61, 0.99)	274	0.29	(-1.15, 1.53)
FLUV+CLOZ	0.26 (-0.86, 1.38)	42	0.28	(-1.30, 1.82)
RISP	0.19 (-0.16, 0.54)	742	0.27	(-0.93, 1.31)
PIMOZ+CLOZ	0.29 (-0.58, 1.15)	39	0.26	(-1.09, 1.67)
SUL	0.38 (-0.85, 1.62)	38	0.25	(-1.27, 2.03)
HAL	0.23 (-0.15, 0.60)	880	0.25	(-0.90, 1.35)
SERTIND	0.38 (-0.74, 1.50)	213	0.24	(-1.18, 1.94)
QUE	0.27 (-0.23, 0.78)	323	0.24	(-0.91, 1.45)
QUE+CLOZ	0.81 (-0.63, 2.25)	23	0.15	(-1.01, 2.63)
CPZ	0.91 (0.41, 1.40)	598	0.06	(-0.27, 2.08)
Dcyclos+CLOZ	1.49 (0.09, 2.89)	11	0.04	(-0.29, 3.28)

Figure S 10.3b. Forest plot with prediction intervals for the secondary outcome: Negative symptoms
Reference comparator: clozapine. AMI: amisulpride; ARI: aripiprazole; BEZOAET: benzoate sodium; CELECOX: celecoxib; CEREBR: cerebrolysin; CLOZ: clozapine; CPZ: chlorpromazine; Dcyclos: D-cycloserine; DESMOP: desmopressin; Dser: D-serine; DUL: duloxetine; FLUO: fluoxetine; FLUPH: fluphenazine; FLUPHLA: fluphenazine decanoate; FLUV: fluvoxamine; GinkBil: Ginkgo biloba; GLY: glycine; HAL: haloperidol; LAM: lamotrigine; LEV: levomepromazine; LI: lithium MAZIN: mazindol; MEM: memantine; MINOC: minocycline; MIRT: mirtazapine; MODF: modafinil; Nacetyl: N-acetylcysteine; OLA: olanzapine; ONDAST: ondansetron; OXYT: oxytocin; PALMIT: palmitoylethanolamide; PIMOZ: pimozide; *PTX:* pentoxifylline*;* QUE: quetiapine; RESVER: resveratrol; RISP: risperidone; SARC: sarcosine; SER: sertraline; SERTIND: sertindole; SUL: sulpiride; TOPIR: topiramate; VitD3: vitamin D3; VORT: vortioxetine; ZIP: ziprasidone; ZOT: zotepine.

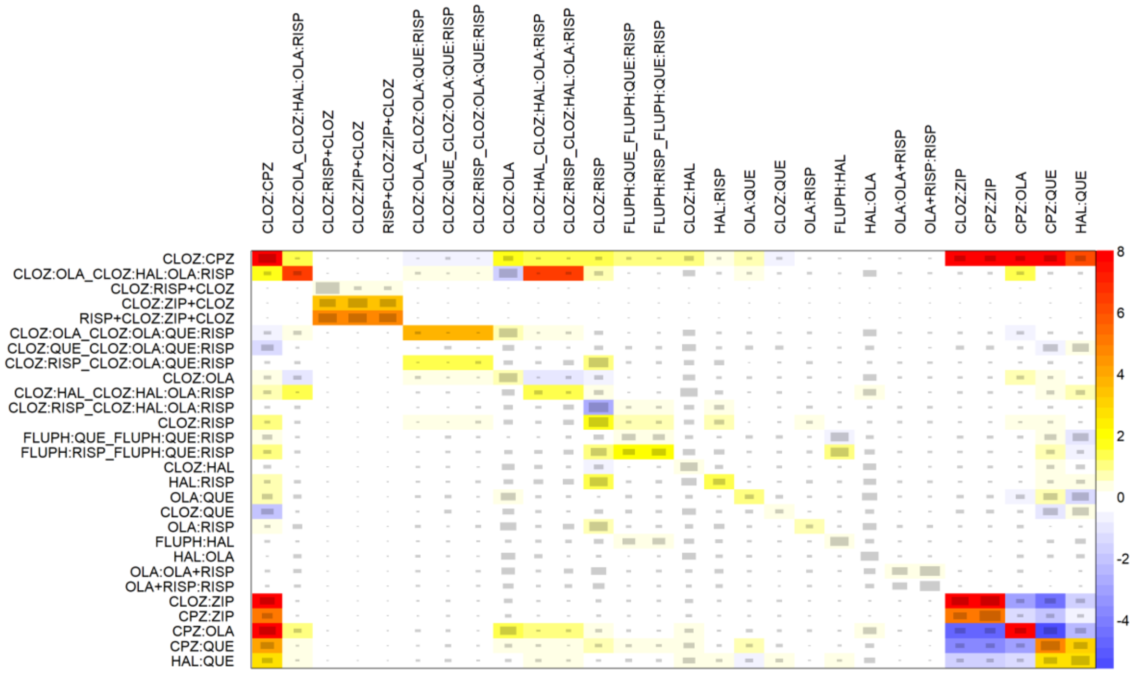

Figure S 10.3c. Net heat plot for the secondary outcome: Negative symptoms
CLOZ: clozapine; CPZ: chlorpromazine; FLUPH: fluphenazine; HAL: haloperidol; OLA: olanzapine; QUE: quetiapine; RISP: risperidone; ZIP: ziprasidone.

10.4. Depressive symptoms (Figure S 10.4a; Figure S 10.4b; Figure S 10.4c)

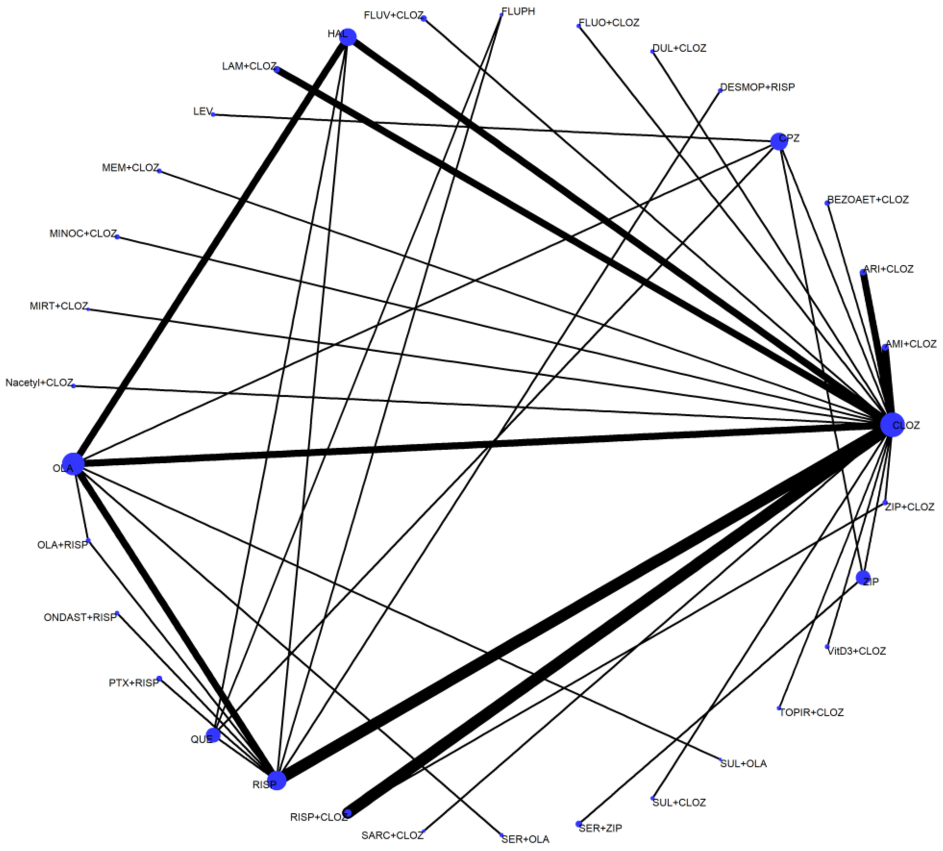

Figure S 10.4a. Network plot for the secondary outcome: Depressive symptoms
AMI: amisulpride; ARI: aripiprazole; BEZOAET: benzoate sodium; CLOZ: clozapine; CPZ: chlorpromazine; DESMOP: desmopressin; DUL: duloxetine; FLUO: fluoxetine; FLUPH: fluphenazine; FLUV: fluvoxamine; HAL: haloperidol; LAM: lamotrigine; LEV: levomepromazine; MEM: memantine; MINOC: minocycline; MIRT: mirtazapine; Nacetyl: N-acetylcysteine; OLA: olanzapine; ONDAST: ondansetron; PTX: pentoxifylline; QUE: quetiapine; RESVER: resveratrol; RISP: risperidone; SARC: sarcosine; SER: sertraline; SUL: sulpiride; TOPIR: topiramate; VitD3: vitamin D3; ZIP: ziprasidone.

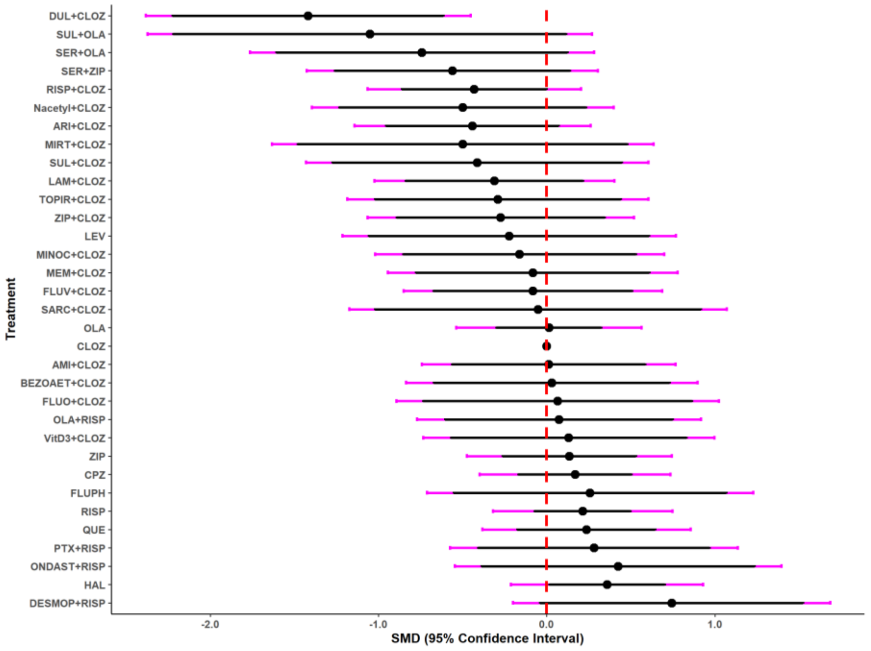

Treatment	TE (95% CI)	Sample	P-score	P.I.
DUL+CLOZ	-1.42 (-2.22, -0.61)	20	0.97	(-2.38, -0.45)
SUL+OLA	-1.05 (-2.22, 0.11)	8	0.89	(-2.37, 0.27)
SER+OLA	-0.74 (-1.61, 0.12)	17	0.83	(-1.76, 0.28)
SER+ZIP	-0.56 (-1.26, 0.14)	49	0.78	(-1.43, 0.30)
RISP+CLOZ	-0.43 (-0.86, 0.00)	63	0.74	(-1.07, 0.20)
Nacetyl+CLOZ	-0.50 (-1.23, 0.23)	21	0.74	(-1.40, 0.40)
ARI+CLOZ	-0.44 (-0.95, 0.07)	43	0.73	(-1.14, 0.26)
MIRT+CLOZ	-0.50 (-1.48, 0.48)	10	0.71	(-1.63, 0.63)
SUL+CLOZ	-0.41 (-1.27, 0.45)	16	0.68	(-1.43, 0.60)
LAM+CLOZ	-0.31 (-0.84, 0.22)	40	0.66	(-1.03, 0.40)
TOPIR+CLOZ	-0.29 (-1.02, 0.44)	19	0.63	(-1.19, 0.60)
ZIP+CLOZ	-0.27 (-0.89, 0.34)	31	0.62	(-1.07, 0.52)
LEV	-0.22 (-1.06, 0.61)	19	0.58	(-1.22, 0.77)
MINOC+CLOZ	-0.16 (-0.85, 0.53)	27	0.55	(-1.02, 0.70)
MEM+CLOZ	-0.08 (-0.78, 0.61)	25	0.49	(-0.94, 0.78)
FLUV+CLOZ	-0.08 (-0.67, 0.51)	42	0.49	(-0.85, 0.69)
SARC+CLOZ	-0.05 (-1.02, 0.92)	10	0.47	(-1.17, 1.07)
OLA	0.01 (-0.30, 0.32)	676	0.44	(-0.54, 0.56)
CLOZ		801	0.44	
AMI+CLOZ	0.01 (-0.56, 0.59)	48	0.43	(-0.74, 0.77)
BEZOAET+CLOZ	0.03 (-0.67, 0.73)	32	0.42	(-0.84, 0.90)
FLUO+CLOZ	0.07 (-0.73, 0.86)	18	0.40	(-0.89, 1.02)
OLA+RISP	0.07 (-0.60, 0.75)	25	0.39	(-0.77, 0.92)
VitD3+CLOZ	0.13 (-0.57, 0.83)	24	0.35	(-0.74, 1.00)
ZIP	0.13 (-0.26, 0.53)	274	0.33	(-0.48, 0.74)
CPZ	0.17 (-0.17, 0.50)	399	0.30	(-0.40, 0.73)
FLUPH	0.26 (-0.55, 1.07)	13	0.28	(-0.71, 1.23)
RISP	0.21 (-0.07, 0.50)	494	0.26	(-0.32, 0.75)
QUE	0.24 (-0.17, 0.64)	277	0.25	(-0.38, 0.85)
PTX+RISP	0.28 (-0.41, 0.97)	35	0.25	(-0.57, 1.14)
ONDAST+RISP	0.42 (-0.39, 1.23)	18	0.19	(-0.55, 1.39)
HAL	0.36 (0.02, 0.70)	397	0.16	(-0.21, 0.93)
DESMOP+RISP	0.74 (-0.04, 1.52)	22	0.07	(-0.20, 1.69)

Figure S 10.4b. Forest plot with prediction intervals for the secondary outcome: Depressive symptoms
Reference comparator: clozapine. AMI: amisulpride; ARI: aripiprazole; BEZOAET: benzoate sodium; CLOZ: clozapine; CPZ: chlorpromazine; DESMOP: desmopressin; DUL: duloxetine; FLUO: fluoxetine; FLUPH: fluphenazine; FLUV: fluvoxamine; HAL: haloperidol; LAM: lamotrigine; LEV: levomepromazine; MEM: memantine; MINOC: minocycline; MIRT: mirtazapine; Nacetyl: N-acetylcysteine; OLA: olanzapine; ONDAST: ondansetron; PTX: pentoxifylline; QUE: quetiapine; RESVER: resveratrol; RISP: risperidone; SARC: sarcosine; SER: sertraline; SUL: sulpiride; TOPIR: topiramate; VitD3: vitamin D3; ZIP: ziprasidone.

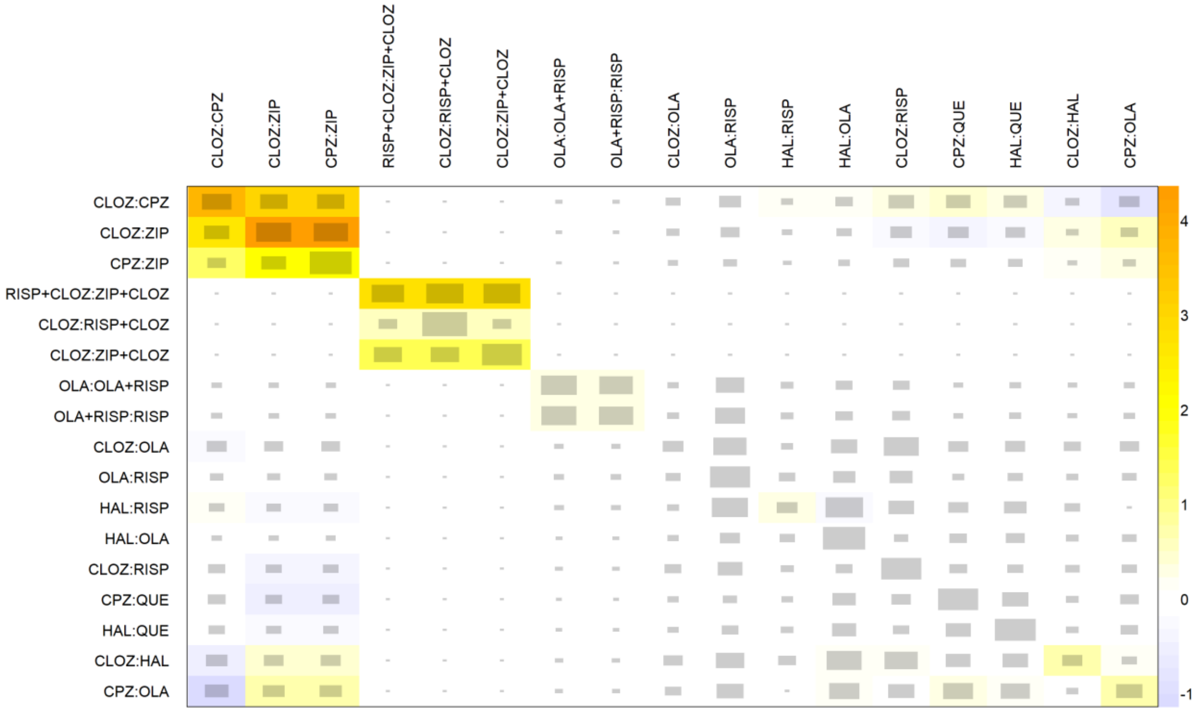

Figure S 10.4c. Net heat plot for the secondary outcome: Depressive symptoms
CLOZ: clozapine; CPZ: chlorpromazine; HAL: haloperidol; OLA: olanzapine; QUE: quetiapine; RISP: risperidone; ZIP: ziprasidone.

10.5. Response rates (Figure S 10.5a; Figure S 10.5b; Figure S 10.5c)

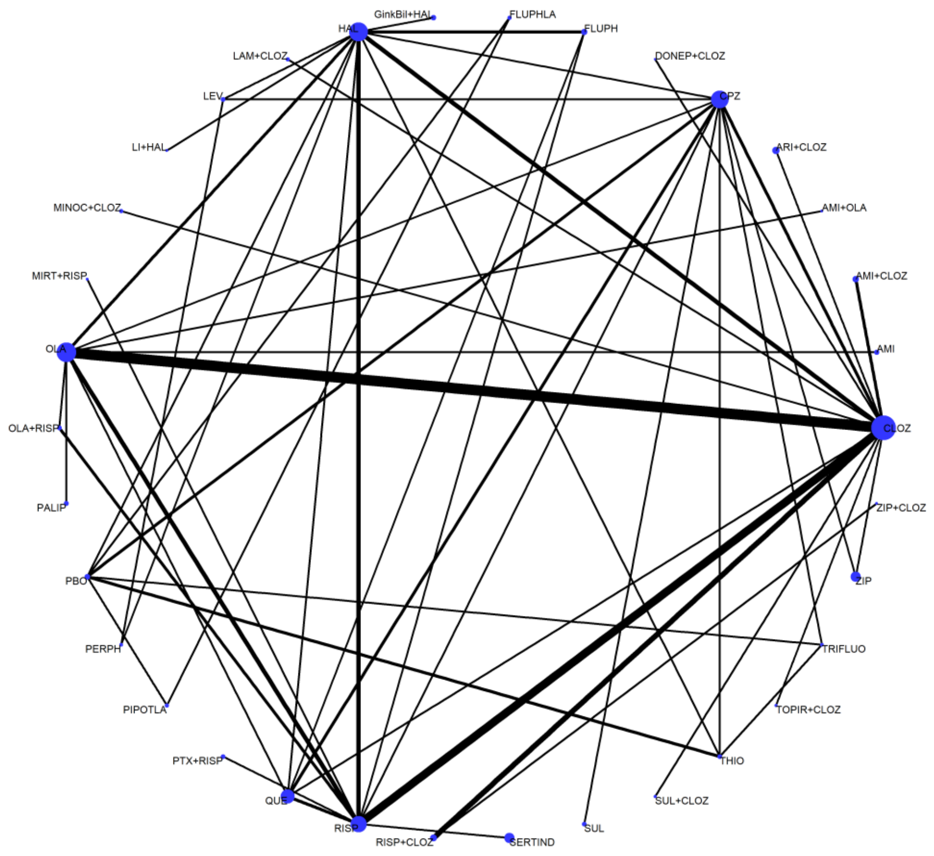

Figure S 10.5a. Network plot for the secondary outcome: Response rates
AMI: amisulpride; ARI: aripiprazole; CLOZ: clozapine; CPZ: chlorpromazine; DESMOP: desmopressin; DONEP: donepezil FLUPH: fluphenazine ; FLUPHLA: fluphenazine decanoate; GinkBil: Ginkgo biloba; HAL: haloperidol; LAM: lamotrigine; LEV: levomepromazine; LI: lithium; MINOC: minocycline; MIRT: mirtazapine; OLA: olanzapine; OXYT: Oxytocin; PALIP: paliperidone; PBO: placebo; PERPH: perphenazine; PIPOTLA: pipotiazine long-acting; PTX: pentoxifylline; RISP: risperidone; SERTIND: sertindole; SUL: sulpiride; THIO: thioridazine; TOPIR: topiramate; TRIFLUO: trifluoperazine; ZIP: ziprasidone.

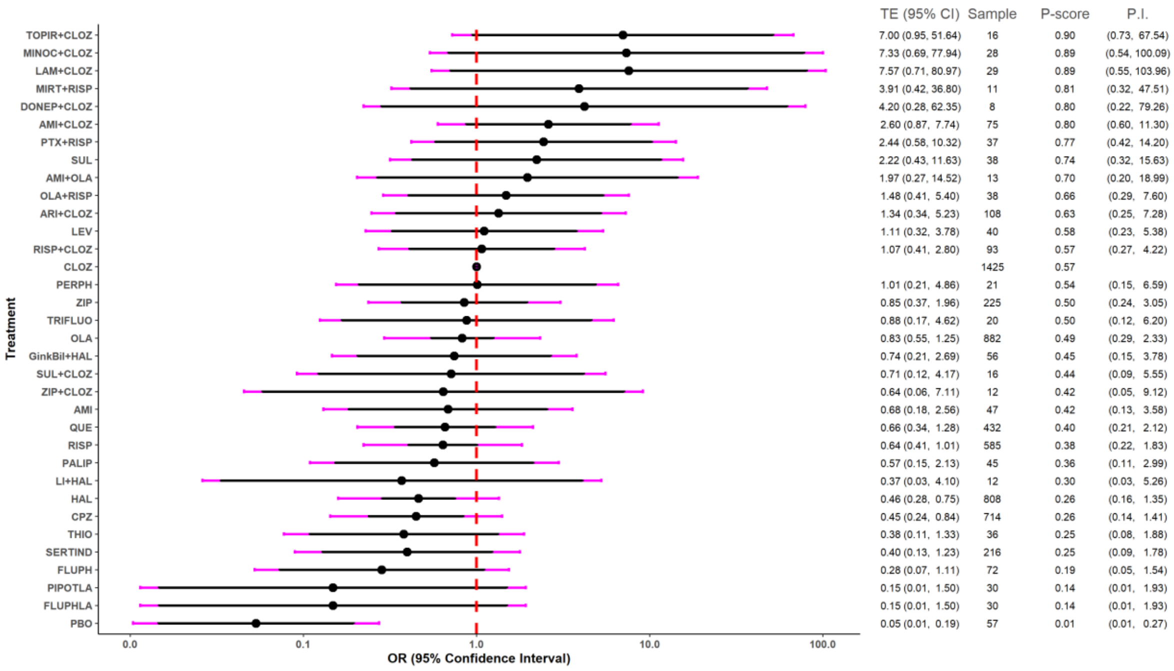

Figure S 10.5b. Forest plot with prediction intervals for the secondary outcome: Response rates
Reference comparator: clozapine. AMI: amisulpride; ARI: aripiprazole; CLOZ: clozapine; CPZ: chlorpromazine; DESMOP: desmopressin; DONEP: donepezil FLUPH: fluphenazine ; FLUPHLA: fluphenazine decanoate; GinkBil: Ginkgo biloba; HAL: haloperidol; LAM: lamotrigine; LEV: levomepromazine; LI: lithium; MINOC: minocycline; MIRT: mirtazapine; OLA: olanzapine; OXYT: Oxytocin; PALIP: paliperidone; PBO: placebo; PERPH: perphenazine; PIPOTLA: pipotiazine long-acting; *PTX:* pentoxifylline*;* RISP: risperidone; SERTIND: sertindole; SUL: sulpiride; THIO: thioridazine; TOPIR: topiramate; TRIFLUO: trifluoperazine; ZIP: ziprasidone.

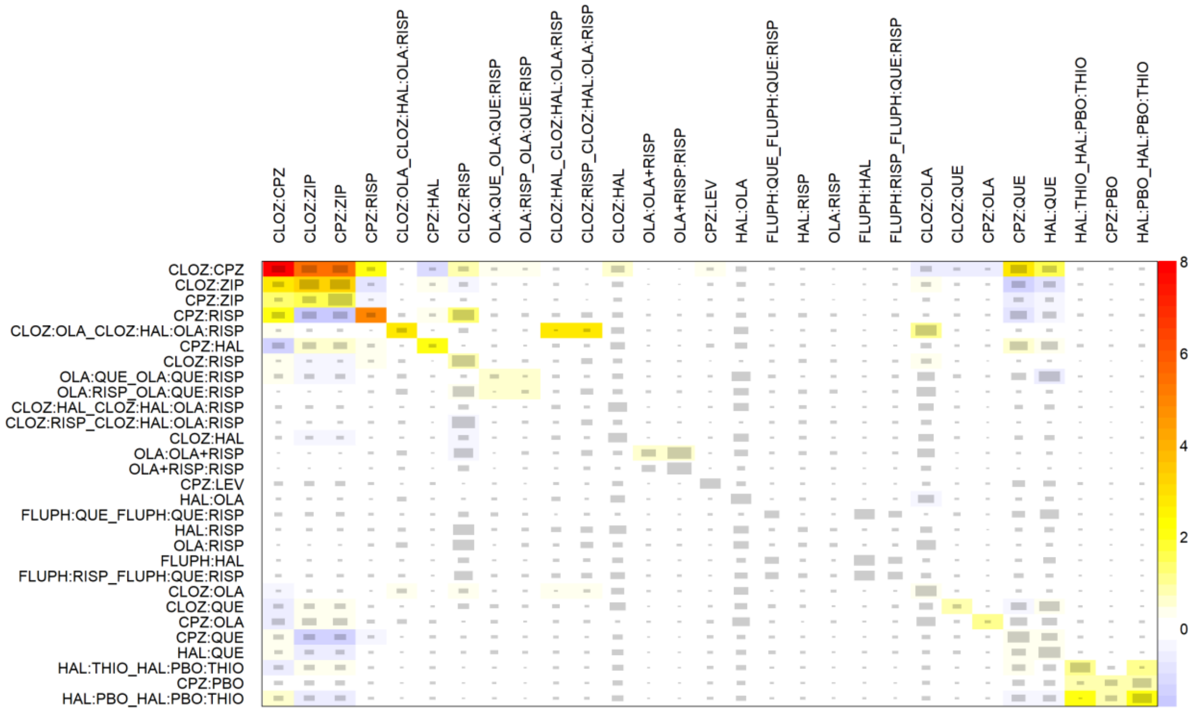

Figure S 10.5c. Net heat plot for the secondary outcome: Response rates
CLOZ: clozapine; CPZ: chlorpromazine; FLUPH: fluphenazine; HAL: haloperidol; LEV: levomepromazine; OLA: olanzapine; QUE: quetiapine; RISP: risperidone; THIO: thioridazine; ZIP: ziprasidone.

10.6. Drop-outs due to any reason (Figure S 10.6a; Figure S 10.6b; Figure S 10.6c)

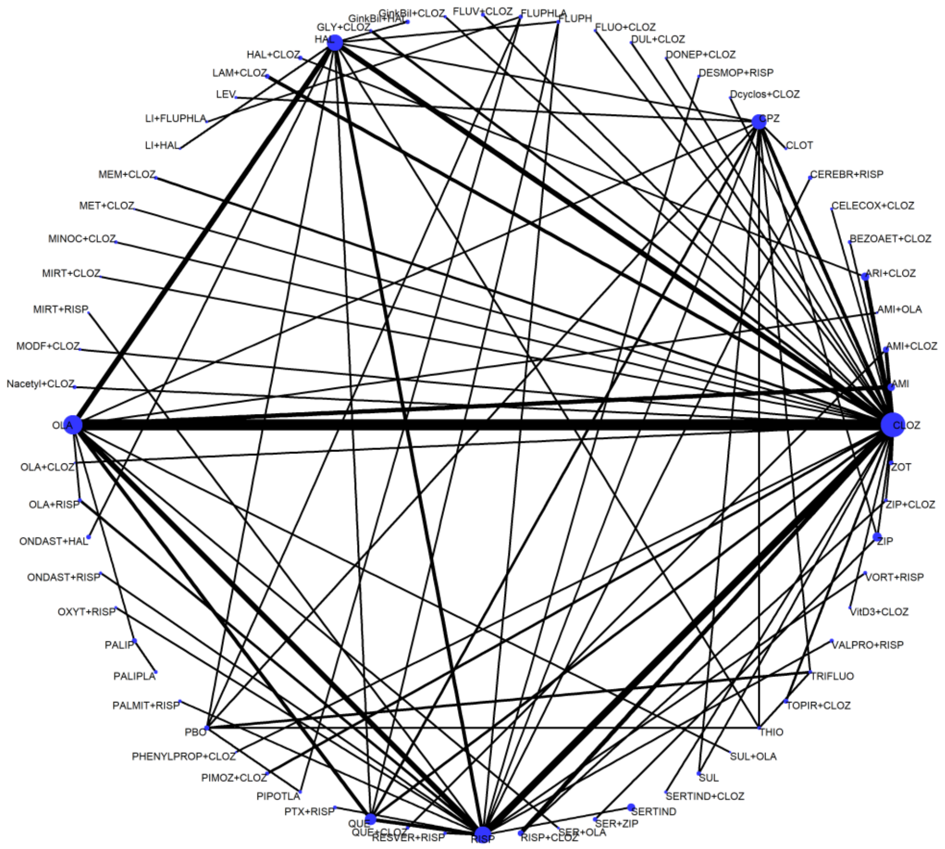

Figure S 10.6a. Network plot for the secondary outcome: Drop-outs due to any reason
AMI: amisulpride; ARI: aripiprazole; BEZOAET: benzoate sodium; CELECOX: celecoxib; CEREBR: cerebrolysin; CLOT: clotiapine; CLOZ: clozapine; CPZ: chlorpromazine; Dcyclos: D-cycloserine; DESMOP: desmopressin; DONEP: donepezil; DUL: duloxetine; FLUO: fluoxetine; FLUPH: fluphenazine; FLUPHLA: fluphenazine decanoate; FLUV: fluvoxamine; GinkBil: Ginkgo biloba; GLY: glycine; HAL: haloperidol; LAM: lamotrigine; LEV: levomepromazine; LI: lithium; MEM: memantine; MET: metformin; MINOC: minocycline; MIRT: mirtazapine; MODF: modafinil; Nacetyl: N-acetylcysteine; OLA: olanzapine; ONDAST: ondansetron; OXYT: oxytocin; PALIP: paliperidone; PALIPLA: paliperidone long-acting; PALMIT: palmitoylethanolamide; PBO: placebo; PHENYLPROP: phenylpropanolamine; PIMOZ: pimozide; PIPOTLA: pipotiazine long-acting; PTX: pentoxifylline; QUE: quetiapine; RESVER: resveratrol; RISP: risperidone; SER: sertraline; SERTIND: sertindole; SUL: sulpiride; THIO: thioridazine; TOPIR: topiramate; TRIFLUO: trifluoperazine; VALPRO: valproate acid VitD3: vitamin D3; VORT: vortioxetine; ZIP: ziprasidone; ZOT: zotepine.

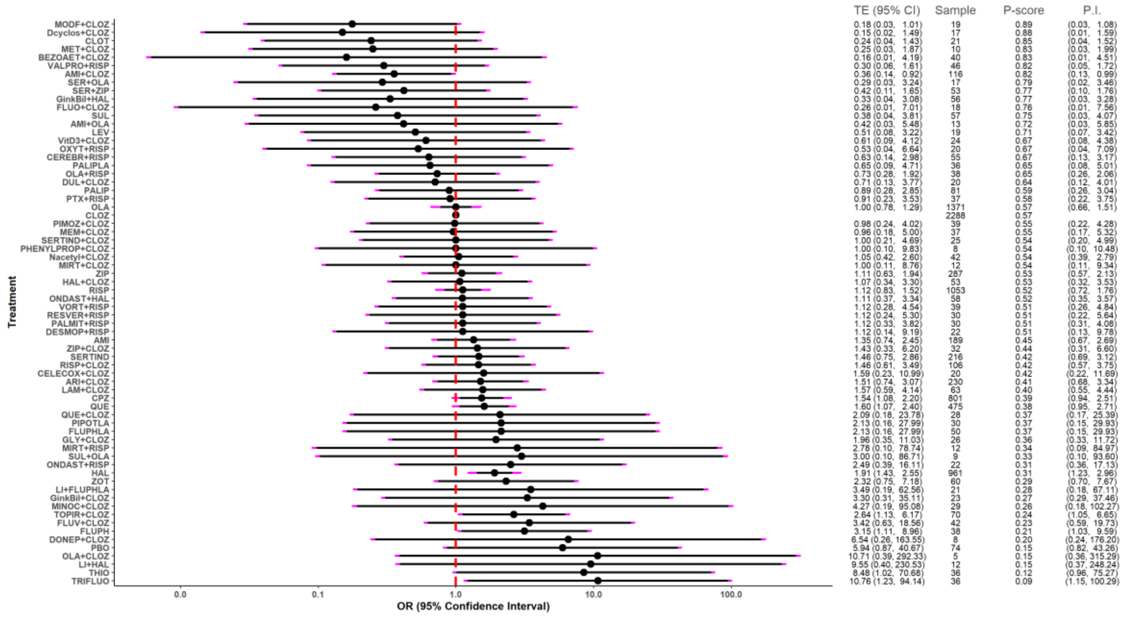

Figure S 10.6b. Forest plot with prediction intervals for the secondary outcome: Drop-outs due to any reason

Reference comparator: clozapine. AMI: amisulpride; ARI: aripiprazole; BEZOAET: benzoate sodium; CELECOX: celecoxib; CEREBR: cerebrolysin; CLOT: clotiapine; CLOZ: clozapine; CPZ: chlorpromazine; Dcyclos: D-cycloserine; DESMOP: desmopressin; DONEP: donepezil; DUL: duloxetine; FLUO: fluoxetine; FLUPH: fluphenazine; FLUPHLA: fluphenazine decanoate; FLUV: fluvoxamine; GinkBil: Ginkgo biloba; GLY: glycine; HAL: haloperidol; LAM: lamotrigine; LEV: levomepromazine; LI: lithium; MEM: memantine; MET: metformin; MINOC: minocycline; MIRT: mirtazapine; MODF: modafinil; Nacetyl: N-acetylcysteine; OLA: olanzapine; ONDAST: ondansetron; OXYT: oxytocin; PALIP: paliperidone; PALIPLA: paliperidone long-acting; PALMIT: palmitoylethanolamide; PBO: placebo; PHENYLPROP: phenylpropanolamine; PIMOZ: pimozide; PIPOTLA: pipotiazine long-acting; PTX: pentoxifylline; QUE: quetiapine; RESVER: resveratrol; RISP: risperidone; SER: sertraline; SERTIND: sertindole; SUL: sulpiride; THIO: thioridazine; TOPIR: topiramate; TRIFLUO: trifluoperazine; VALPRO: valproate acid VitD3: vitamin D3; VORT: vortioxetine; ZIP: ziprasidone; ZOT: zotepine.

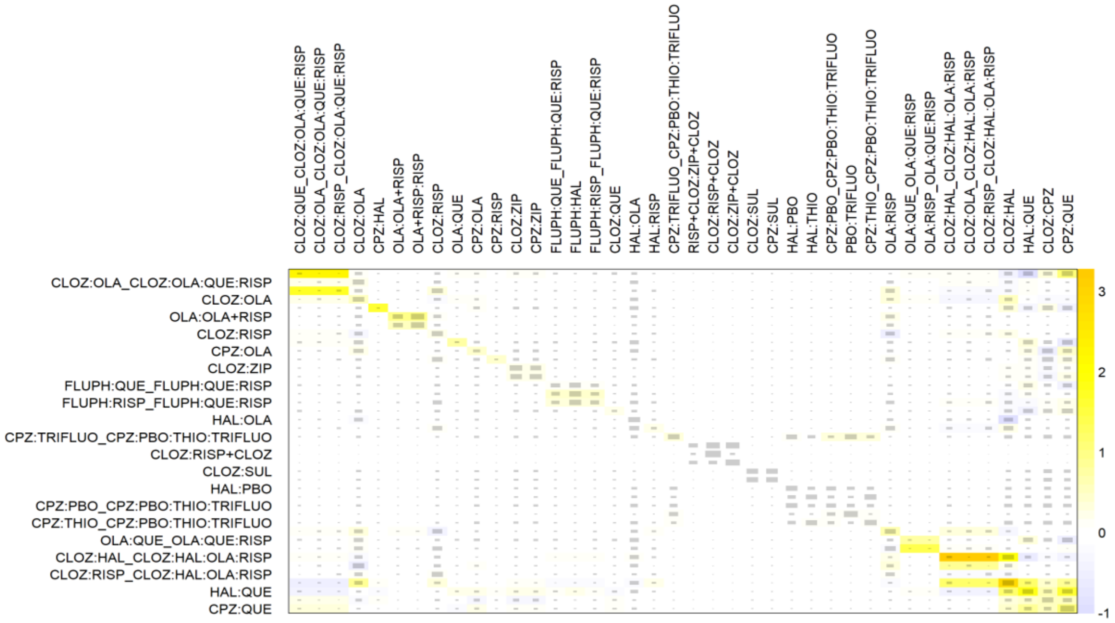

Figure S 10.6c. Net heat plot for the secondary outcome: Drop-outs due to any reason

CLOZ: clozapine; CPZ: chlorpromazine; FLUPH: fluphenazine; HAL: haloperidol; OLA: olanzapine; PBO: placebo; QUE: quetiapine; RISP: risperidone; SUL: sulpiride; THIO: thioridazine; TRIFLUO: trifluoperazine; ZIP: ziprasidone.

10.7. Drop-outs due to any adverse effects (Figure S 10.7a; Figure S 10.7b; Figure S 10.7c)

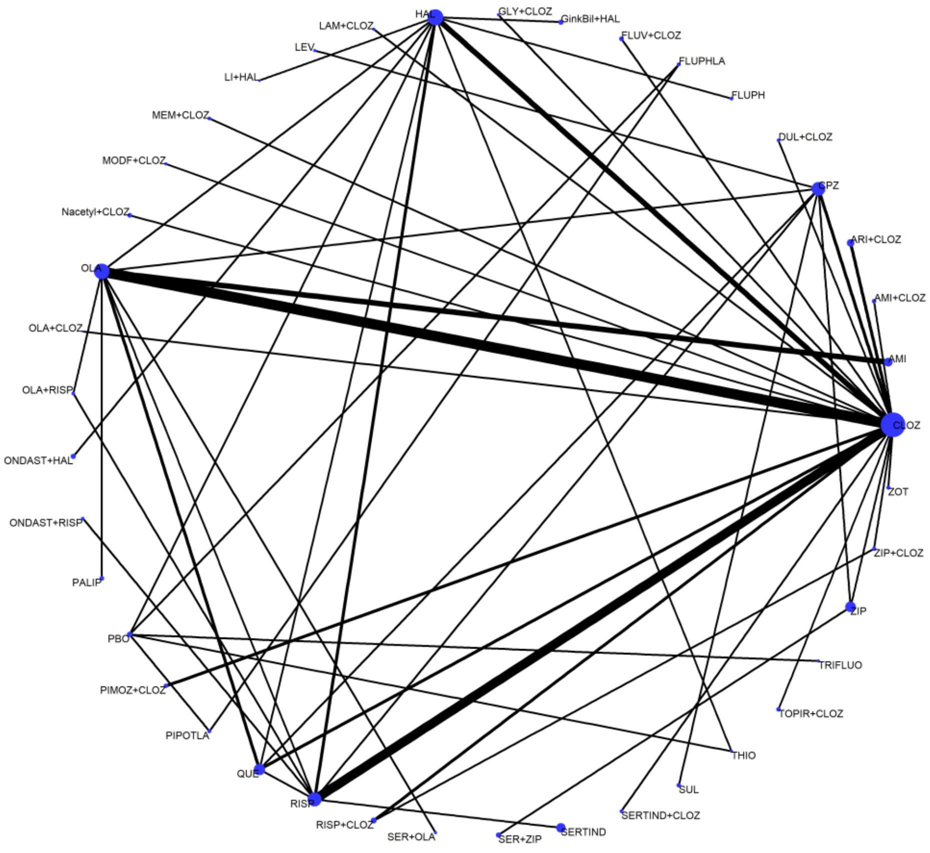

Figure S 10.7a. Network plot for the secondary outcome: Drop-outs due to any adverse effects
AMI: amisulpride; ARI: aripiprazole; CLOZ: clozapine; CPZ: chlorpromazine; DUL: duloxetine; FLUPH: fluphenazine; FLUPHLA: fluphenazine decanoate; FLUV: fluvoxamine; GinkBil: Ginkgo biloba; GLY: glycine; HAL: haloperidol; LAM: lamotrigine; LEV: levomepromazine; LI: lithium; MEM: memantine; MODF: modafinil; Nacetyl: N-acetylcysteine; OLA: olanzapine; ONDAST: ondansetron; PALIP: paliperidone; PBO: placebo; PIMOZ: pimozide; PIPOTLA: pipotiazine long-acting; QUE: quetiapine; RISP: risperidone; SER: sertraline; SERTIND: sertindole; SUL: sulpiride; THIO: thioridazine; TOPIR: topiramate; TRIFLUO: trifluoperazine; ZIP: ziprasidone; ZOT: zotepine.

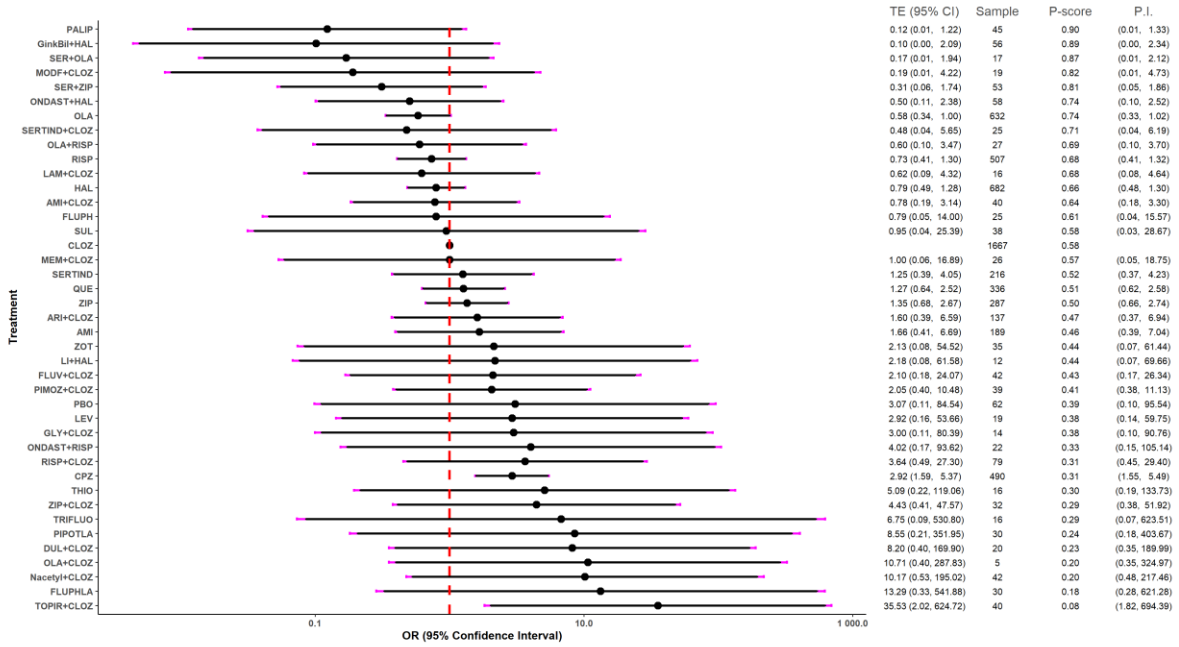

Figure S 10.7b. Forest plot with prediction intervals for the secondary outcome: Drop-outs due to any adverse effects
AMI: amisulpride; ARI: aripiprazole; CLOZ: clozapine; CPZ: chlorpromazine; DUL: duloxetine; FLUPH: fluphenazine; FLUPHLA: fluphenazine decanoate; FLUV: fluvoxamine; GinkBil: Ginkgo biloba; GLY: glycine; HAL: haloperidol; LAM: lamotrigine; LEV: levomepromazine; LI: lithium; MEM: memantine; MODF: modafinil; Nacetyl: N-acetylcysteine; OLA: olanzapine; ONDAST: ondansetron; PALIP: paliperidone; PBO: placebo; PIMOZ: pimozide; PIPOTLA: pipotiazine long-acting; QUE: quetiapine; RISP: risperidone; SER: sertraline; SERTIND: sertindole; SUL: sulpiride; THIO: thioridazine; TOPIR: topiramate; TRIFLUO: trifluoperazine; ZIP: ziprasidone; ZOT: zotepine.

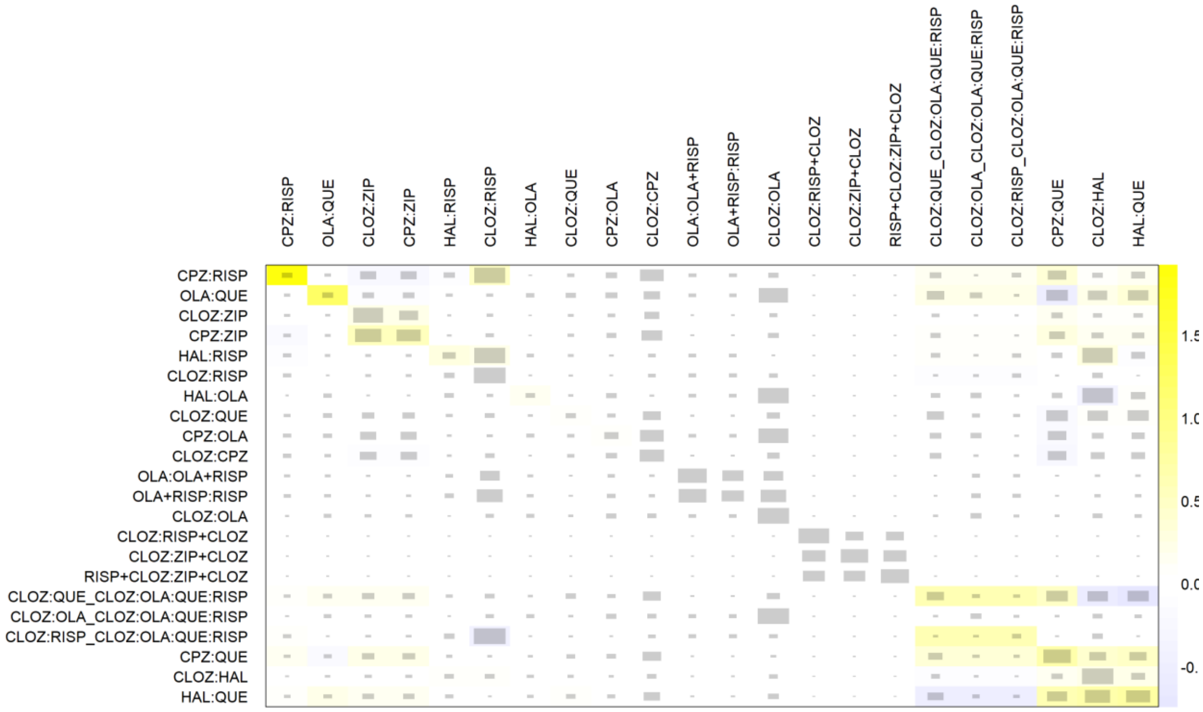

Figure S 10.7c. Net heat plot for the secondary outcome: Drop-outs due to any adverse effects
CLOZ: clozapine; CPZ: chlorpromazine; HAL: haloperidol; OLA: olanzapine; QUE: quetiapine; RISP: risperidone; ZIP: ziprasidone.

10.8.Drop-outs due to inefficacy (Figure S 10.8a; Figure S 10.8b; Figure S 10.8c)

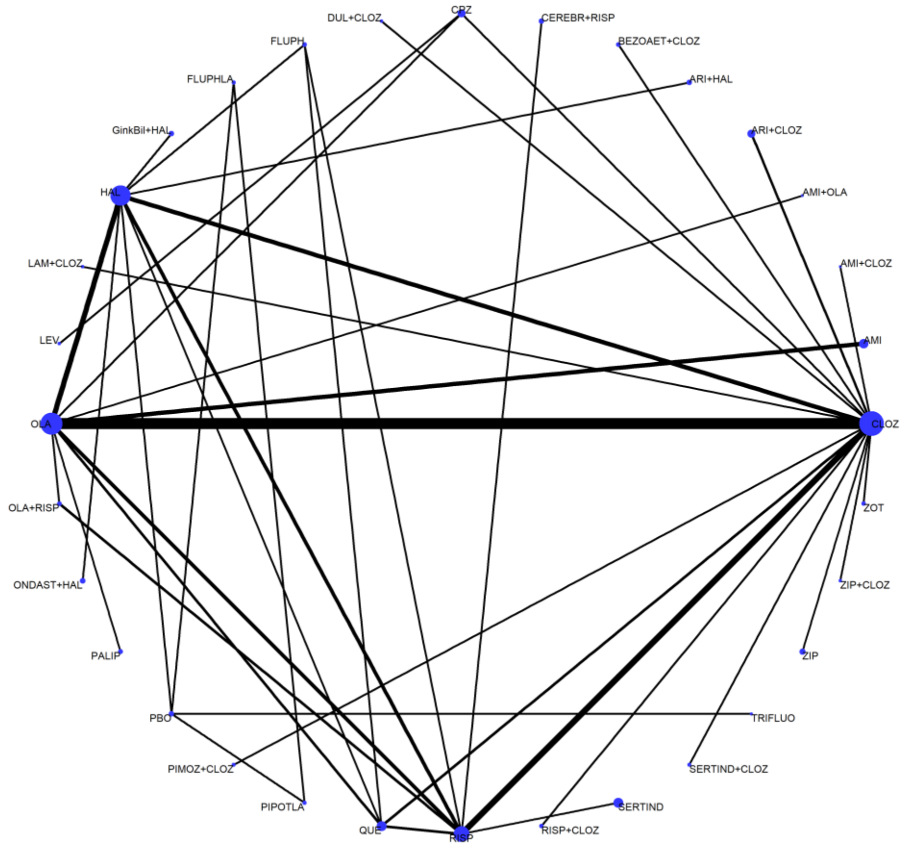

Figure S 10.8a. Network plot for the secondary outcome: Drop-outs due to inefficacy
AMI: amisulpride; ARI: aripiprazole; BEZOAET: benzoate sodium; CEREBR: cerebrolysin; CLOZ: clozapine; CPZ: chlorpromazine; DUL: duloxetine; FLUPH: fluphenazine; FLUPHLA: fluphenazine decanoate; GinkBil: Ginkgo biloba; haloperidol; HAL: haloperidol; LAM: lamotrigine; LEV: levomepromazine; LI: lithium; OLA: olanzapine; ONDAST: ondansetron; PALIP: paliperidone; PBO: placebo; PIMOZ: pimozide; PIPOTLA: pipotiazine long-acting; QUE: quetiapine; RISP: risperidone; SERTIND: sertindole; TRIFLUO: trifluoperazine; ZIP: ziprasidone; ZOT: zotepine.

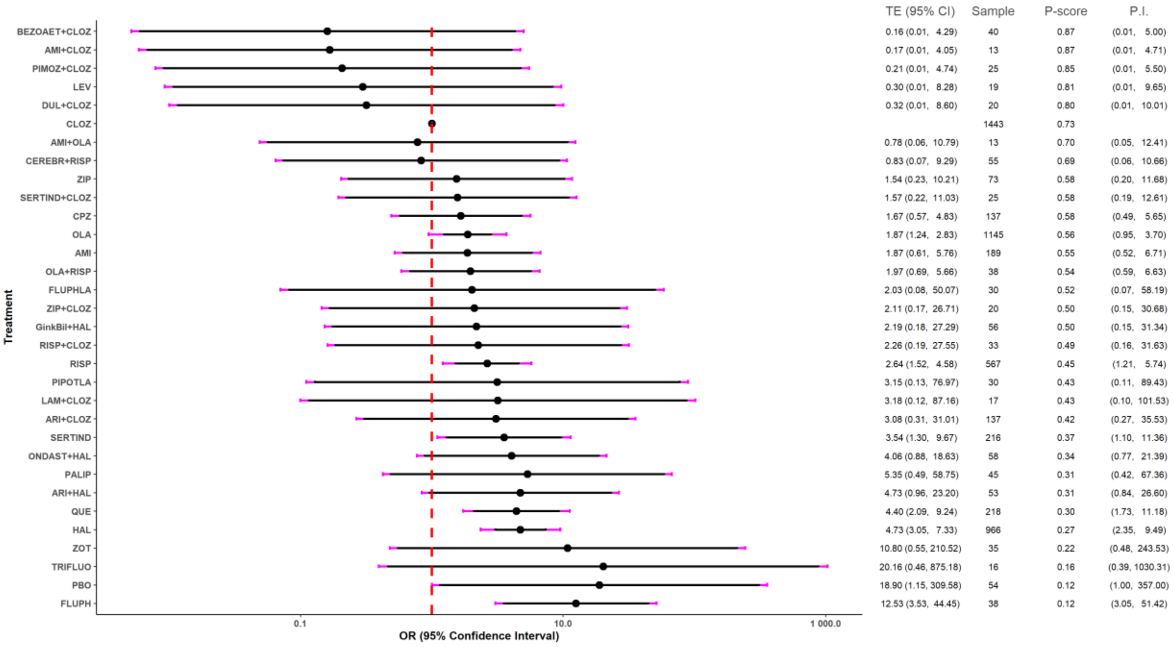

Figure S 10.8b. Forest plot with prediction intervals for the secondary outcome: Drop-outs due to inefficacy

Reference comparator: clozapine. AMI: amisulpride; ARI: aripiprazole; BEZOAET: benzoate sodium; CEREBR: cerebrolysin; CLOZ: clozapine; CPZ: chlorpromazine; DUL: duloxetine; FLUPH: fluphenazine; FLUPHLA: fluphenazine decanoate; GinkBil: Ginkgo biloba; haloperidol; HAL: haloperidol; LAM: lamotrigine; LEV: levomepromazine; LI: lithium; OLA: olanzapine; ONDAST: ondansetron; PALIP: paliperidone; PBO: placebo; PIMOZ: pimozide; PIPOTLA: pipotiazine long-acting; QUE: quetiapine; RISP: risperidone; SERTIND: sertindole; TRIFLUO: trifluoperazine; ZIP: ziprasidone; ZOT: zotepine.

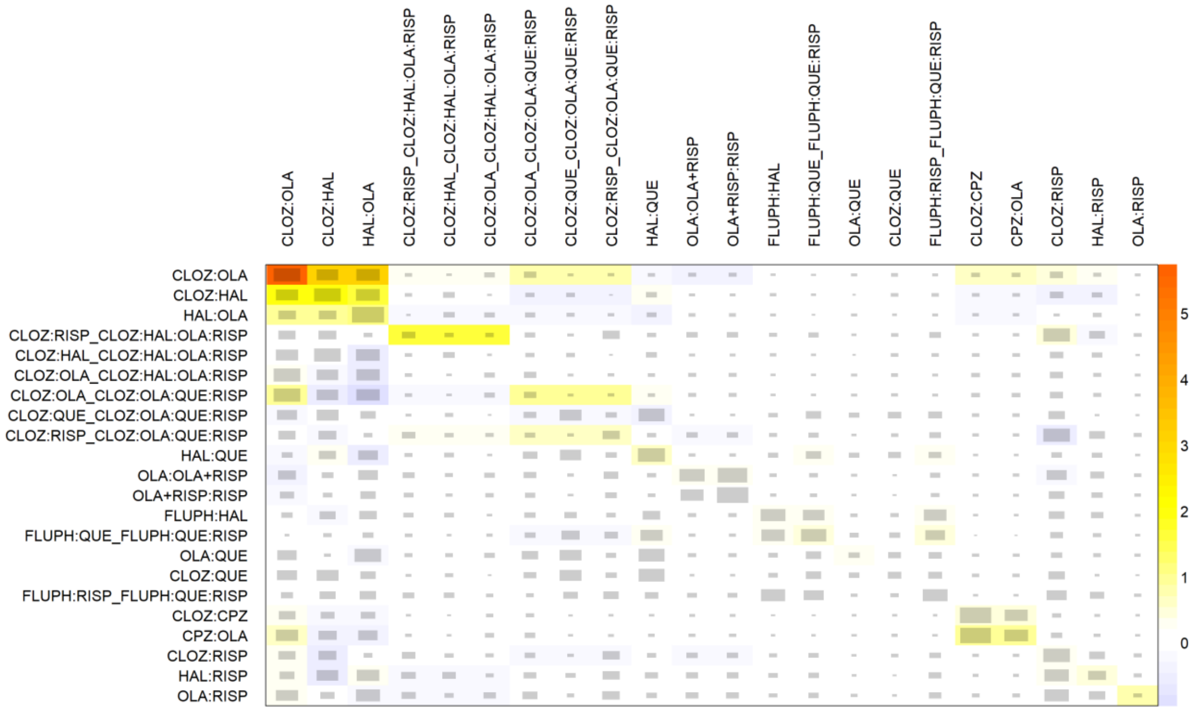

Figure S 10.8c. Net heat plot for the secondary outcome: Drop-outs due to inefficacy

CLOZ: clozapine; HAL: haloperidol; FLUPH: fluphenazine; OLA: olanzapine; QUE: quetiapine; RISP: risperidone.

10.9. Total number of participants with adverse effects (Figure S 10.9a; Figure S 10.9b; Figure S 10.9c)

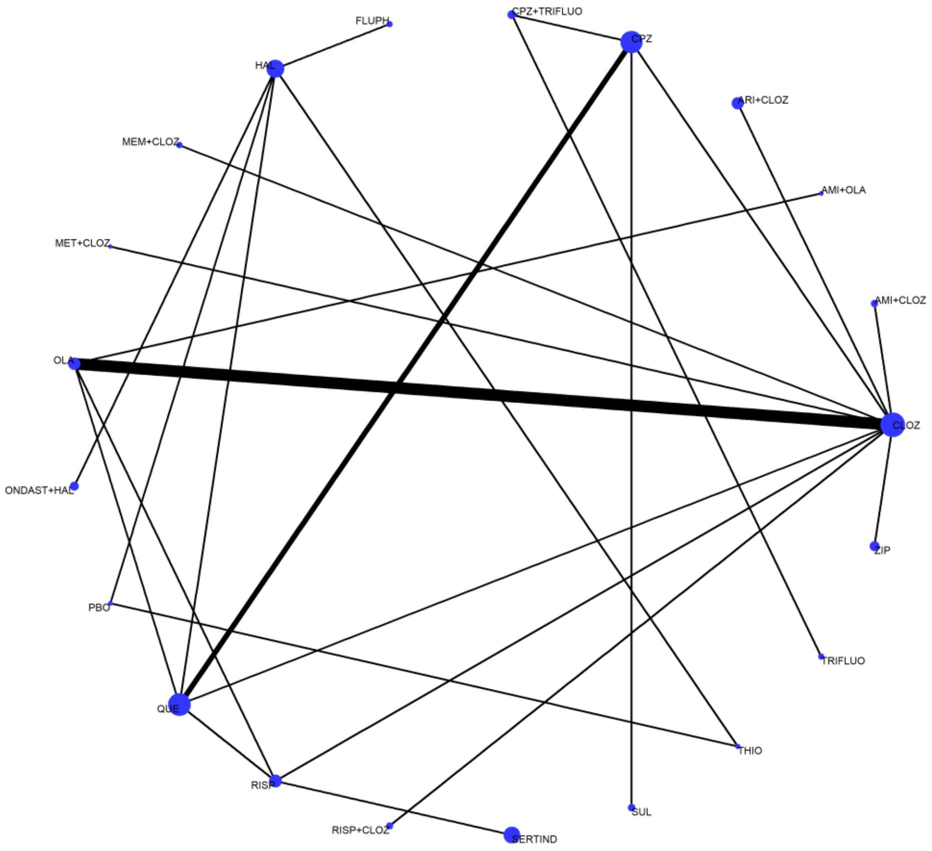

Figure S 10.9a. Network plot for the secondary outcome: Total number of participants experiencing adverse effects

AMI: amisulpride; ARI: aripiprazole; CLOZ: clozapine; CPZ: chlorpromazine; FLUPH: fluphenazine; HAL: haloperidol; MEM: memantine; MET: metformin; OLA: olanzapine; ONDAST: ondansetron; PBO: placebo; QUE: quetiapine; RISP: risperidone; SERTIND: sertindole; SUL: sulpiride; THIO: thioridazine; TRIFLUO: trifluoperazine; ZIP: ziprasidone.

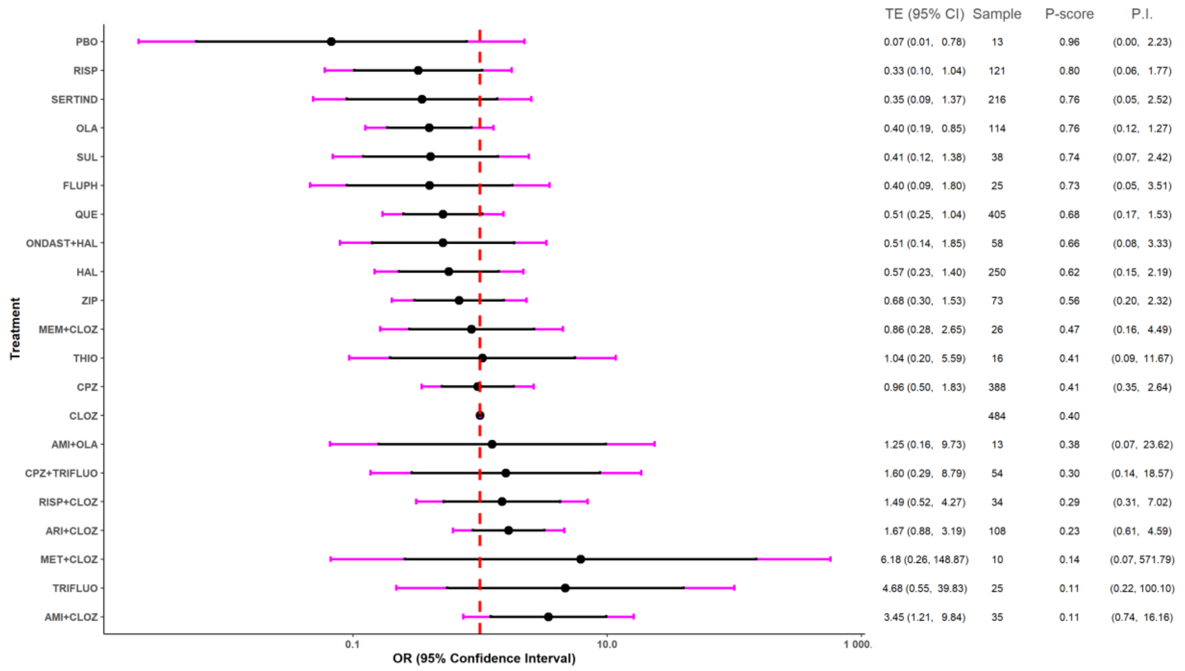

Figure S 10.9b. Forest plot with prediction intervals for the secondary outcome: Total number of participants experiencing adverse effects

Reference comparator: clozapine. AMI: amisulpride; ARI: aripiprazole; CI: confidence intervals; CLOZ: clozapine; CPZ: chlorpromazine; FLUPH: fluphenazine; HAL: haloperidol; MEM: memantine; MET: metformin; OLA: olanzapine; ONDAST: ondansetron; OR: odds ratio; PBO: placebo; P.I.: prediction interval; QUE: quetiapine; RISP: risperidone; SERTIND: sertindole; SUL: sulpiride; TE: treatment effect; THIO: thioridazine; TRIFLUO: trifluoperazine; ZIP: ziprasidone.

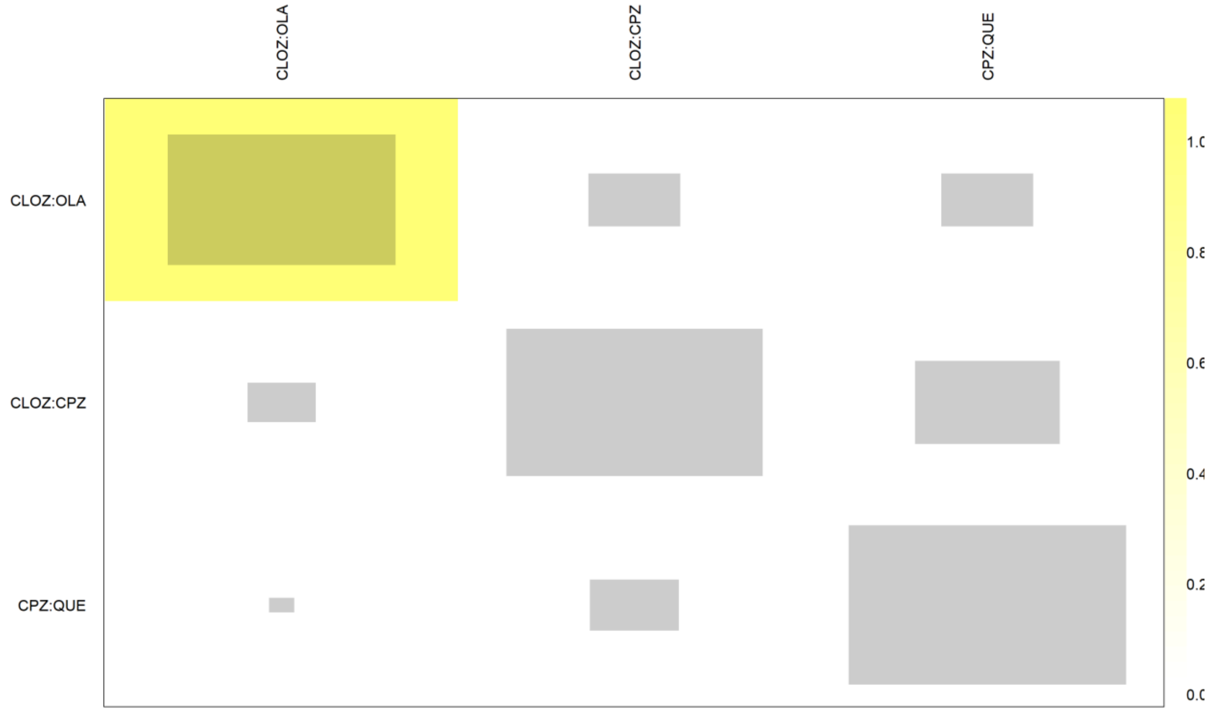

Figure S 10.9c. Net heat plot for the secondary outcome: Total number of participants experiencing adverse effects

CLOZ: clozapine; CPZ: chlorpromazine; OLA: olanzapine; QUE: quetiapine.

10.10. Antiparkinsonian medication use (Figure S 10.10a; Figure S 10.10b; Figure S 10.10c)

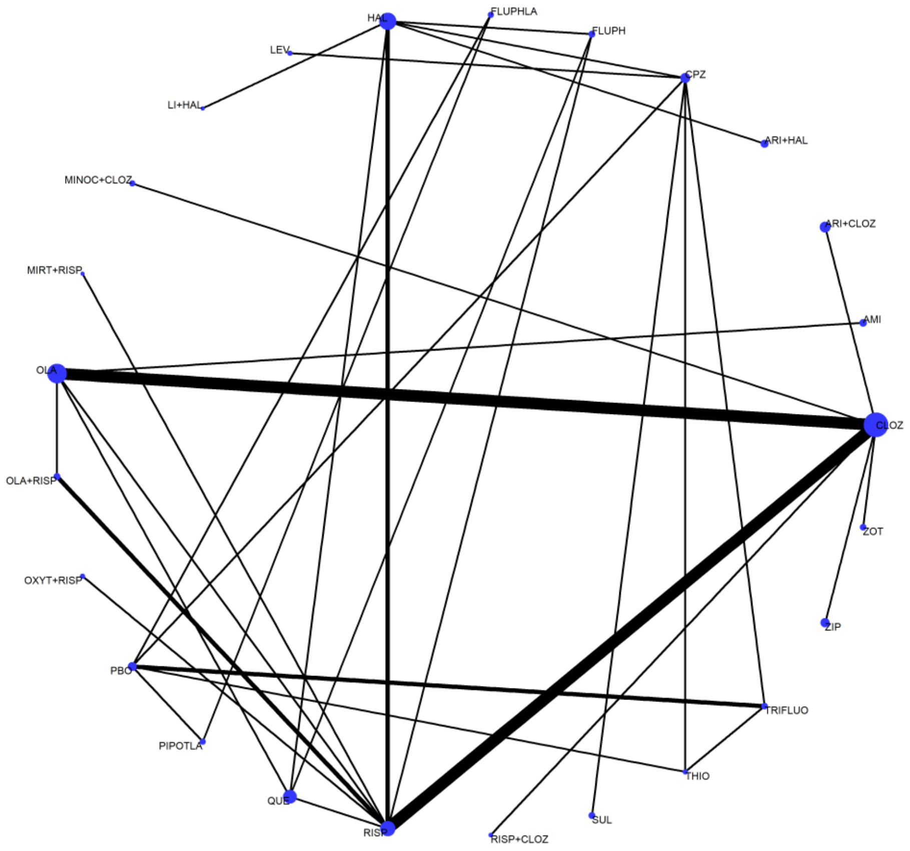

Figure S 10.10a. Network plot for the secondary outcome: Antiparkinsonian medication use
AMI: amisulpride; ARI: aripiprazole; CLOZ: clozapine; CPZ: chlorpromazine; FLUO: fluoxetine; FLUPHLA: fluphenazine decanoate; HAL: haloperidol; LEV: levomepromazine; LI: lithium; MINOC: minocycline; MIRT: mirtazapine; OLA: olanzapine; OXYT: oxytocin; PBO: placebo; PIPOTLA: pipotiazine long-acting; QUE: quetiapine; RISP: risperidone; THIO: thioridazine; TRIFLUO: trifluoperazine; ZIP: ziprasidone; ZOT: zotepine.

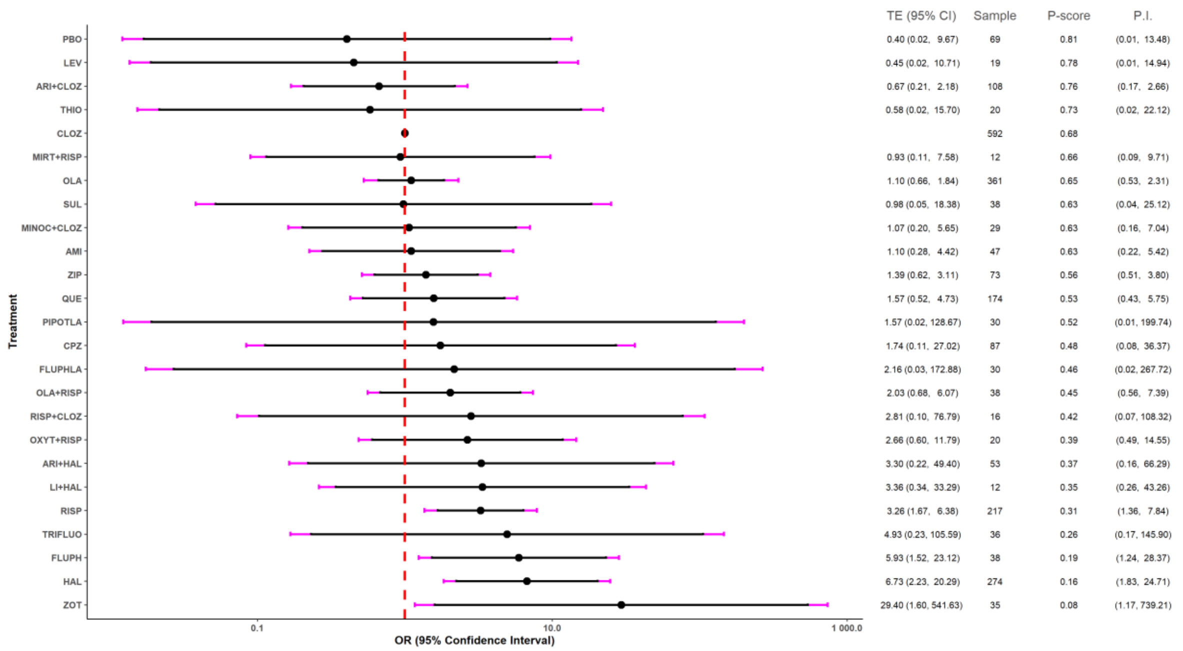

Figure S 10.10b. Forest plot with prediction intervals for the secondary outcome: Antiparkinsonian medication use

Reference comparator: Clozapine. AMI: amisulpride; ARI: aripiprazole; CI: confidence intervals; CLOZ: clozapine; CPZ: chlorpromazine; FLUO: fluoxetine; FLUPHLA: fluphenazine decanoate; HAL: haloperidol; LEV: levomepromazine; LI: lithium; MINOC: minocycline; MIRT: mirtazapine; OLA: olanzapine; OR: odds ratio; OXYT: oxytocin; PBO: placebo; P.I.: prediction interval; PIPOTLA: pipotiazine long-acting; QUE: quetiapine; RISP: risperidone; TE: treatment effect; THIO: thioridazine; TRIFLUO: trifluoperazine; ZIP: ziprasidone; ZOT: zotepine.

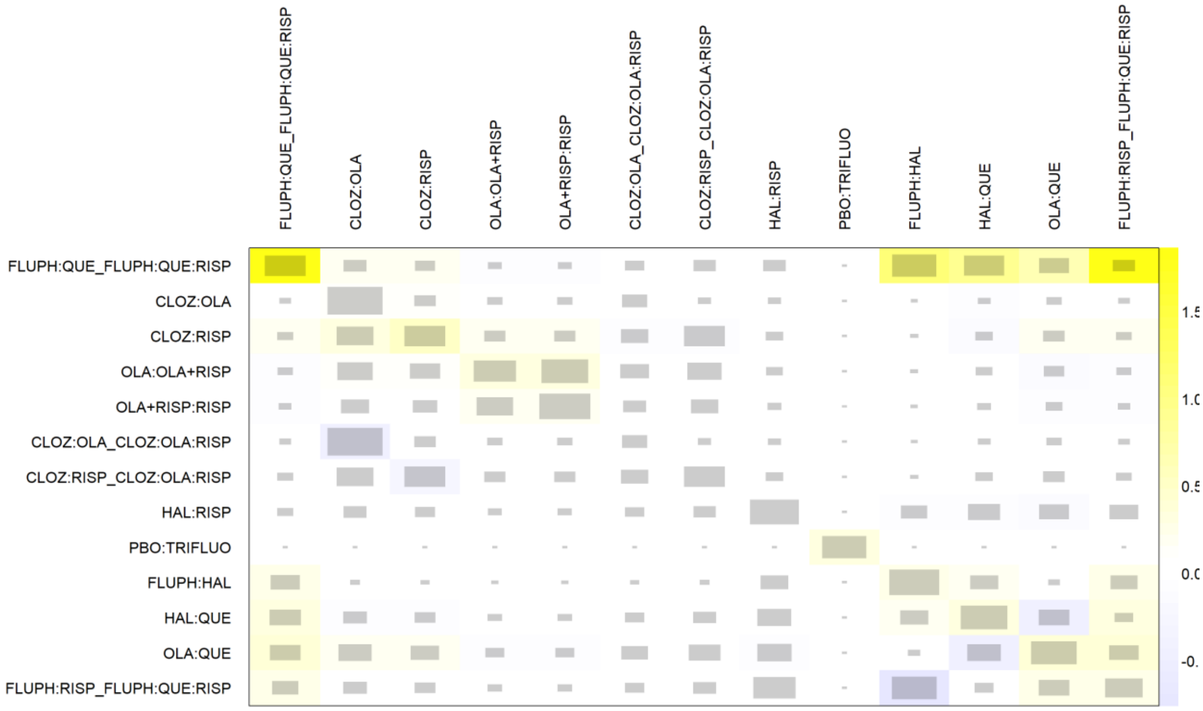

Figure S 10.10c. Net heat plot for the secondary outcome: Antiparkinsonian medication use

CLOZ: clozapine; FLUPH: fluphenazine; HAL: haloperidol; OLA: olanzapine; PBO: placebo; QUE: quetiapine; RISP: risperidone; TRIFLUO: trifluoperazine; ZIP: ziprasidone.

10.11. Sedation (Figure S 10.11a; Figure S 10.11b; Figure S 10.11c)

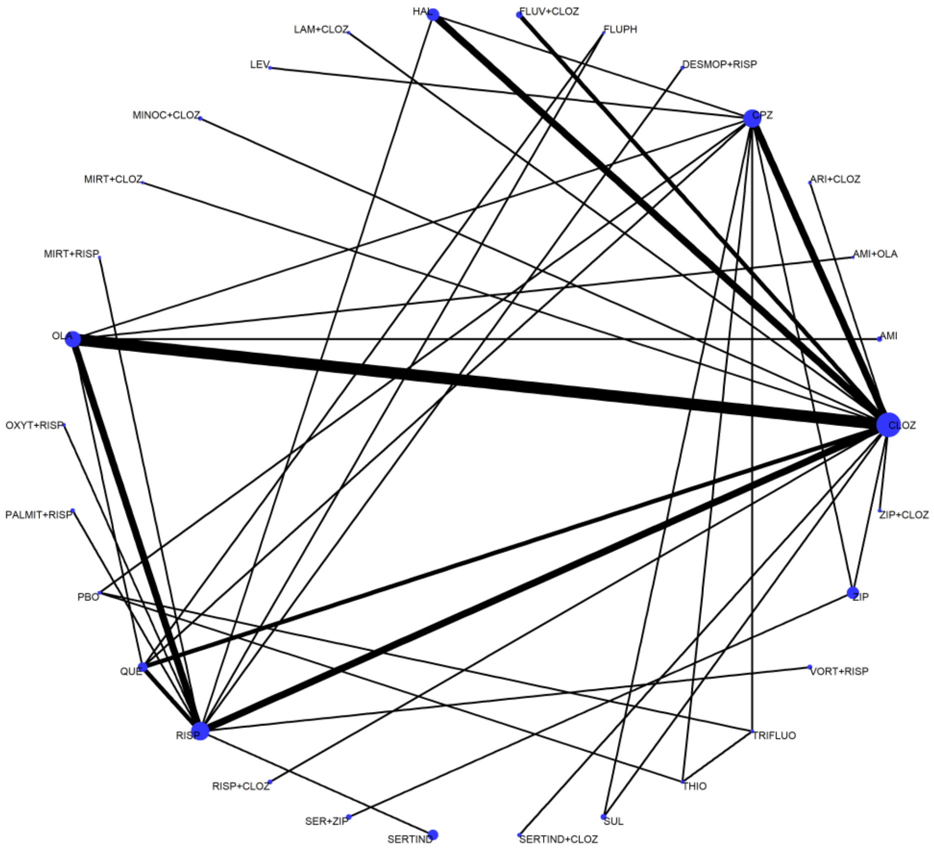

Figure S 10.11a. Network plot for the secondary outcome: Sedation
AMI: amisulpride; ARI: aripiprazole; CLOZ: clozapine; CPZ: chlorpromazine; DESMOP: desmopressin; FLUPH: fluphenazine; FLUV: fluvoxamine; HAL: haloperidol; LAM: lamotrigine; MINOC: minocycline; MIRT: mirtazapine; OLA: olanzapine; OXYT: Oxytocin; PALMIT: palmitoylethanolamide; PBO: placebo; PHENPROP: phenylpropanolamine; PIMOZ: pimozide; QUE: quetiapine; RISP: risperidone; SER: sertraline; SERTIND: sertindole; SUL: sulpiride; THIO: thioridazine; VORT: vortioxetine; ZIP: ziprasidone.

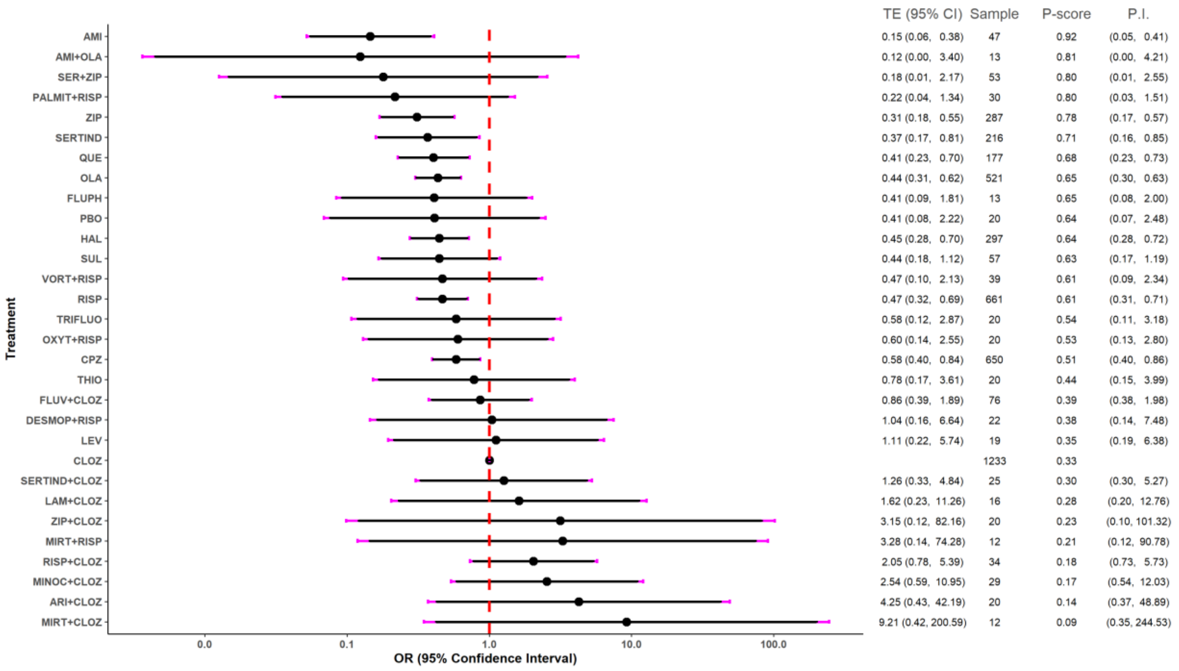

Figure S 10.11b. Forest plot with prediction intervals for the secondary outcome: Sedation
Reference comparator: clozapine. AMI: amisulpride; ARI: aripiprazole; CI: confidence intervals; CLOZ: clozapine; CPZ: chlorpromazine; DESMOP: desmopressin; FLUPH: fluphenazine; FLUV: fluvoxamine; HAL: haloperidol; LAM: lamotrigine; MINOC: minocycline; MIRT: mirtazapine; OLA: olanzapine; OR: odds ratio; OXYT: Oxytocin; PALMIT: palmitoylethanolamide; PBO: placebo; PHENPROP: phenylpropanolamine; P.I.: prediction interval; PIMOZ: pimozide; QUE: quetiapine; RISP: risperidone; SER: sertraline; SERTIND: sertindole; SUL: sulpiride; TE: treatment effect; THIO: thioridazine; VORT: vortioxetine; ZIP: ziprasidone.

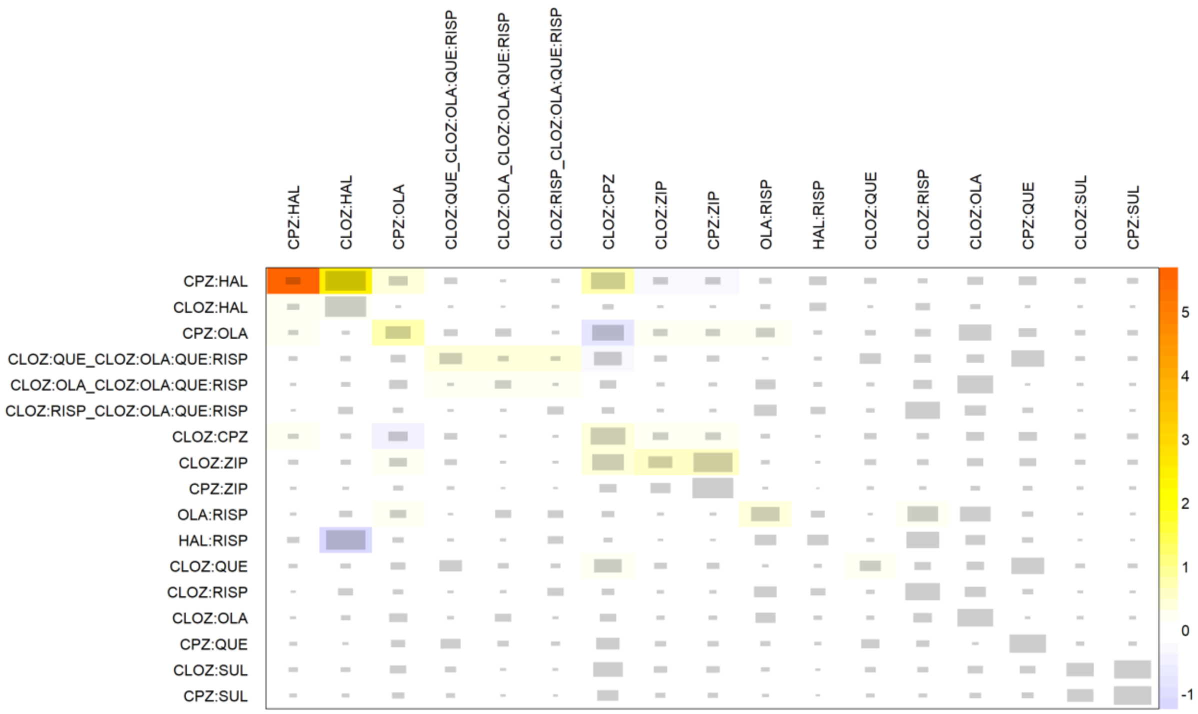

Figure S 10.11c. Net heat plot for the secondary outcome: Sedation
CLOZ: clozapine; CPZ: chlorpromazine; HAL: haloperidol; OLA: olanzapine; RISP: risperidone; QUE: quetiapine; ZIP: ziprasidone.

10.12. Weight gain (Figure S 10.12a; Figure S 10.12b; Figure S 10.12c)

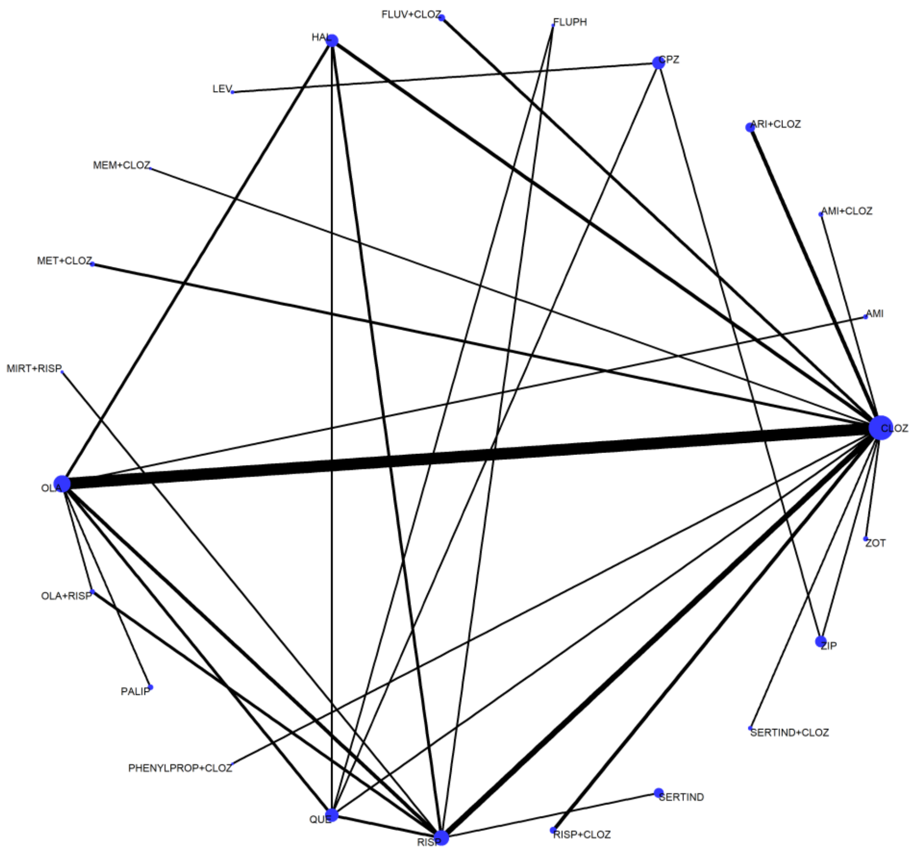

Figure S 10.12a. Network plot for the secondary outcome: Weight gain
AMI: amisulpride; ARI: aripiprazole; CLOZ: clozapine; CPZ: chlorpromazine; FLUPH: fluphenazine decanoate; FLUV: fluvoxamine; HAL: haloperidol; LEV: levomepromazine; MEM: memantine; MET: metformin; MIRT: mirtazapine; OLA: olanzapine; PALIP: paliperidone; PHENYLPROP: phenylpropanolamine; QUE: quetiapine; RISP: risperidone; SERTIND: Sertindole; ZIP: ziprasidone; ZOT: zotepine.

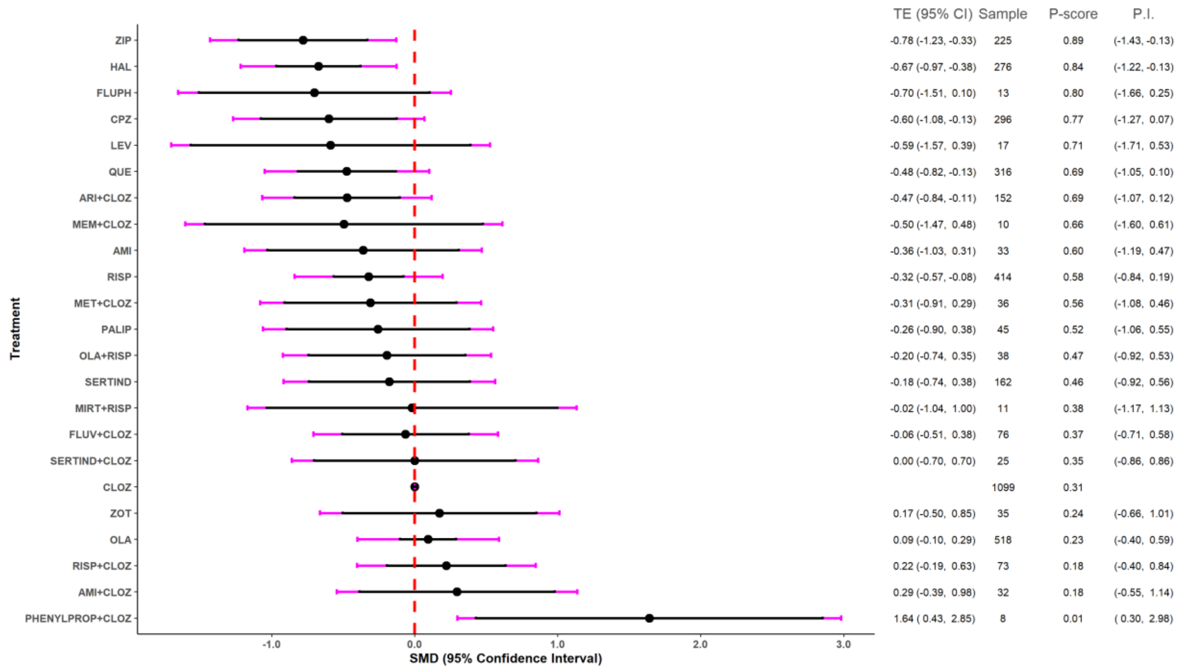

Figure S 10.12b. Forest plot with prediction intervals for the secondary outcome: Weight gain
Reference comparator: Clozapine. AMI: amisulpride; ARI: aripiprazole; CI: confidence intervals; CLOZ: clozapine; CPZ: chlorpromazine; FLUPH: fluphenazine decanoate; FLUV: fluvoxamine; HAL: haloperidol; LEV: levomepromazine; MEM: memantine; MET: metformin; MIRT: mirtazapine; OLA: olanzapine; PALIP: paliperidone; PHENYLPROP: phenylpropanolamine; P.I. prediction interval; QUE: quetiapine; RISP: risperidone; SERTIND: sertindole; SMD: standardized mean difference; TE: treatment effect; ZIP: ziprasidone; ZOT: zotepine.

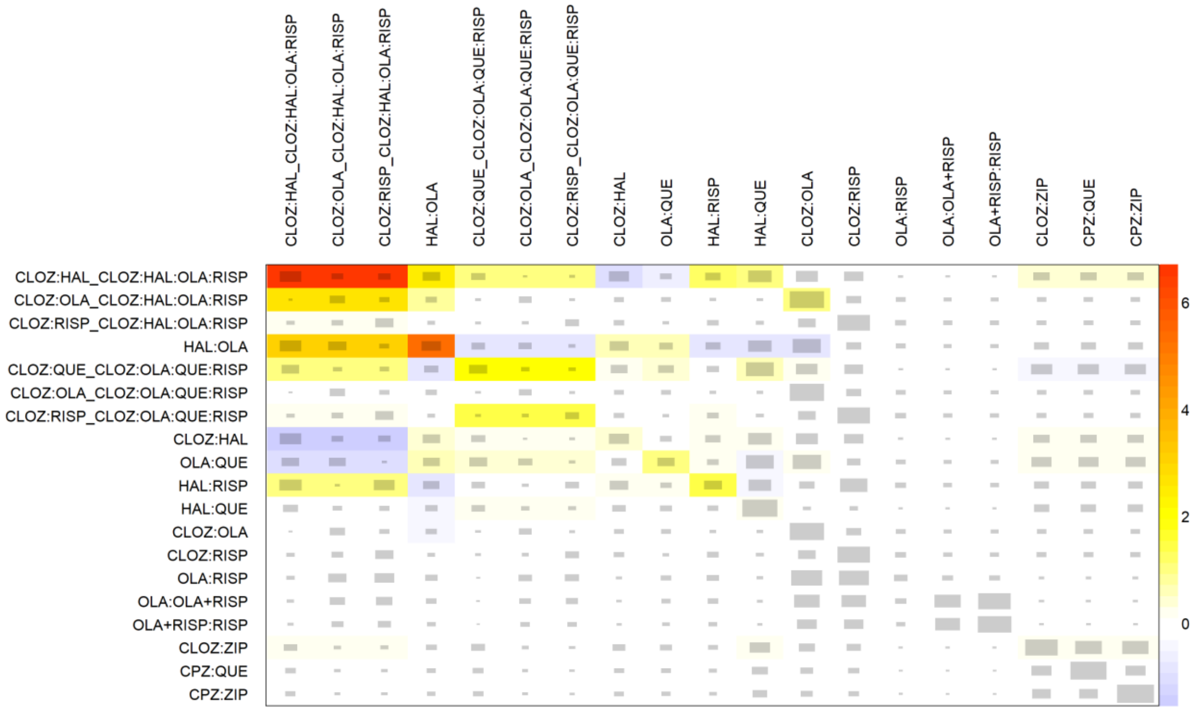

Figure S 10.12c. Net heat plot for the secondary outcome: Weight
CLOZ: clozapine; CPZ: chlorpromazine; HAL: haloperidol; OLA: olanzapine; RISP: risperidone; QUE: quetiapine; ZIP: ziprasidone.

10.13. Prolactin elevation (Figure S 10.13a; Figure S 10.13b; Figure S 10.13c)

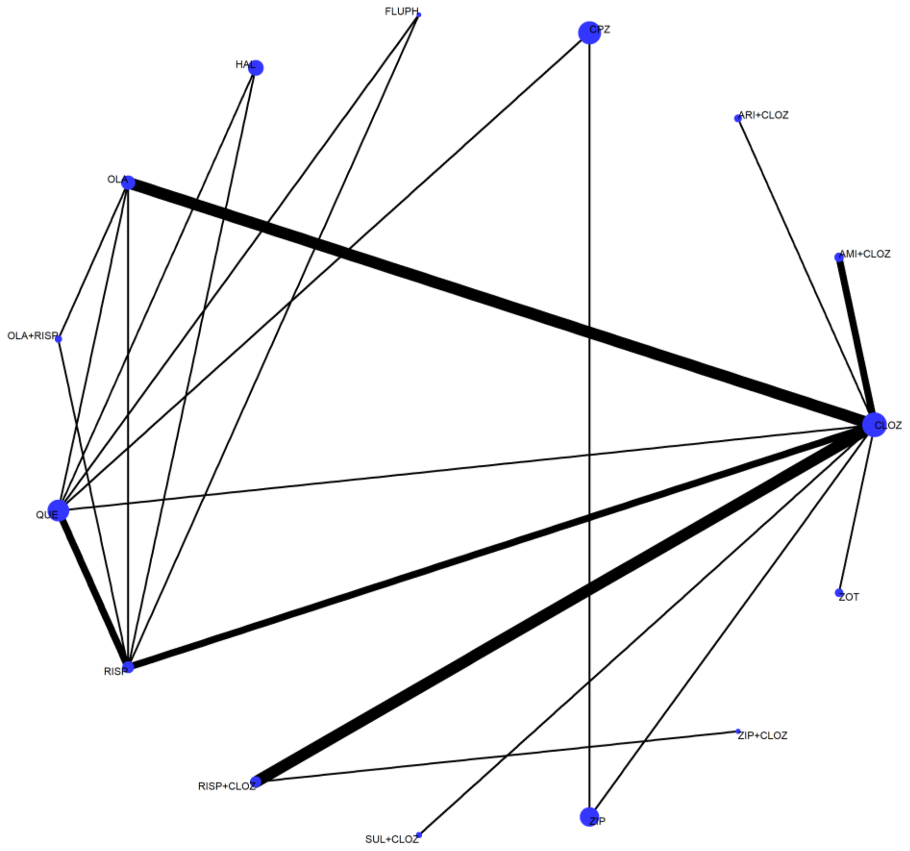

Figure S 10.13a. Network plot for the secondary outcome: Prolactin elevation
AMI: amisulpride; ARI: aripiprazole; CLOZ: clozapine; CPZ: chlorpromazine; FLUPH: fluphenazine decanoate; HAL: haloperidol; OLA: olanzapine; QUE: quetiapine; RISP: risperidone; SUL: sulpiride; ZIP: ziprasidone.

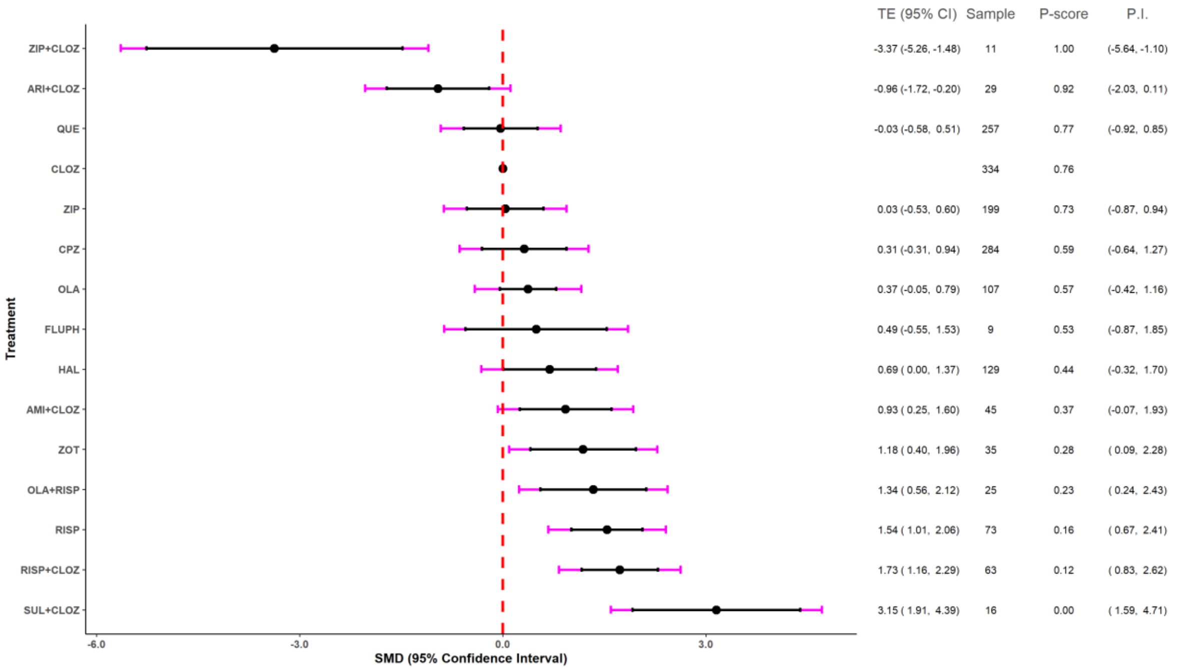

Figure S 10.13b. Forest plot with prediction intervals for the secondary outcome: Prolactin elevation
Reference comparator: Clozapine. AMI: amisulpride; ARI: aripiprazole; CI: confidence intervals; CLOZ: clozapine; CPZ: chlorpromazine; FLUPH: fluphenazine decanoate; HAL: haloperidol; OLA: olanzapine; QUE: quetiapine; P.I.: prediction interval; RISP: risperidone; SMD: standardized mean difference; SUL: sulpiride; TE: treatment effect; ZIP: ziprasidone.

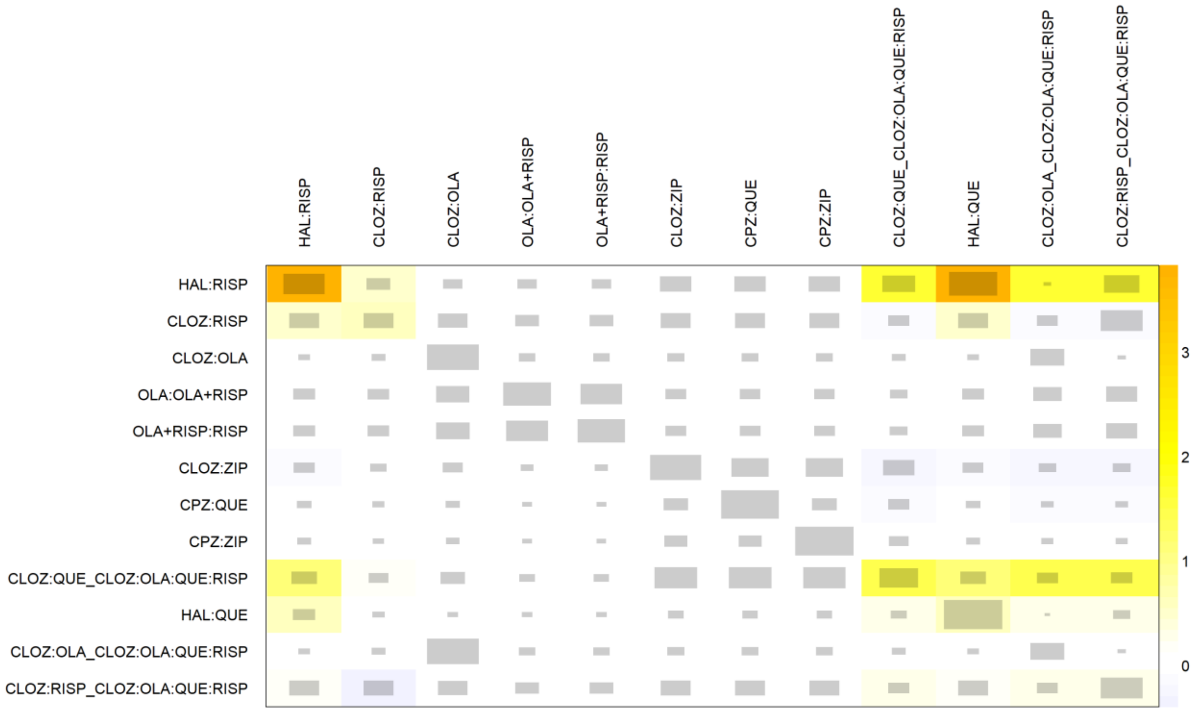

Figure S 10.13c. Net heat plot for the secondary outcome: Prolactin elevation
CLOZ: clozapine; CPZ: chlorpromazine; HAL: haloperidol; OLA: olanzapine; RISP: risperidone; QUE: quetiapine; ZIP: ziprasidone.

10.14. QTc prolongation (Figure S 10.14a; Figure S 10.14b; Figure S 10.14c)

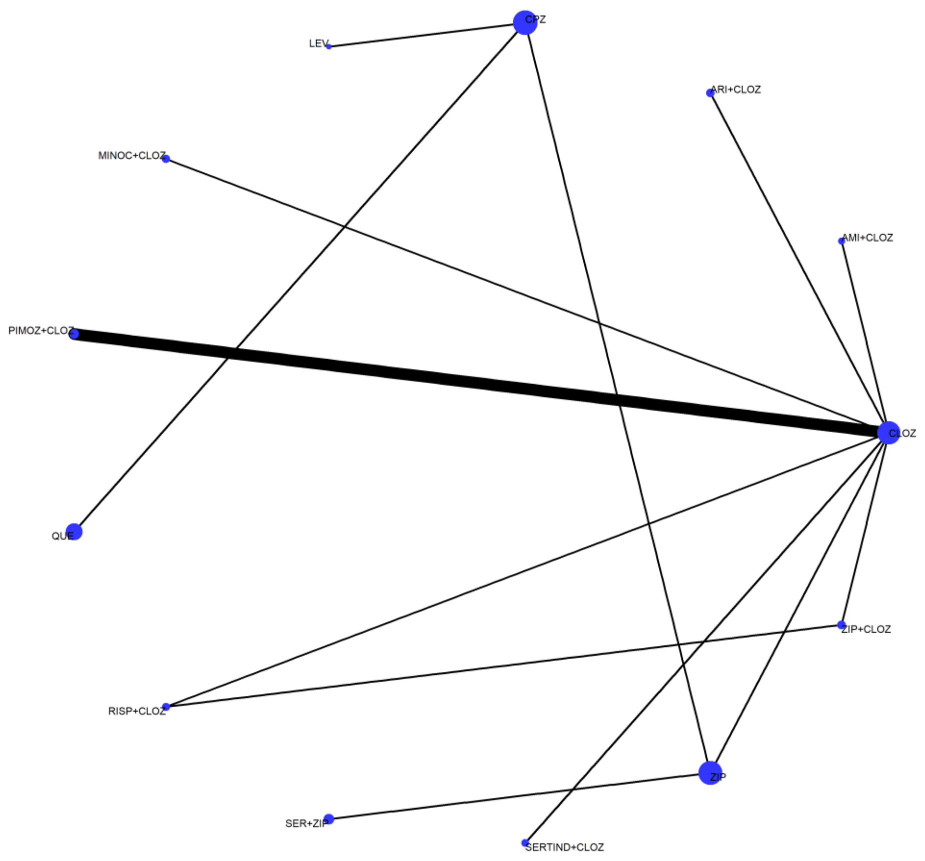

Figure S 10.14a. Network plot for the secondary outcome: QTc prolongation
AMI: amisulpride; ARI: aripiprazole; CLOZ: clozapine; CPZ: chlorpromazine; LEV: levomepromazine; MINOC: minocycline; PIMOZ: pimozide; QUE: quetiapine; RISP: risperidone; SER: sertraline; ZIP: ziprasidone.

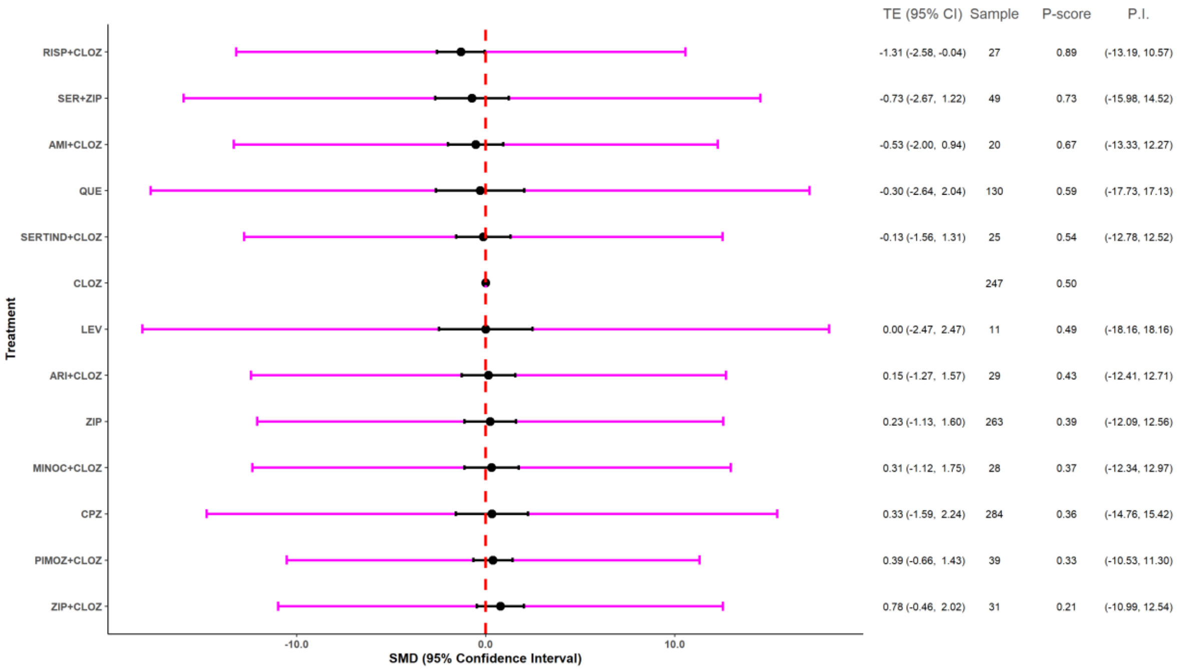

Figure S 10.14b. Forest plot with prediction intervals for the secondary outcome: QTc prolongation
Reference comparator: Clozapine. AMI: amisulpride; ARI: aripiprazole; CI: confidence intervals; CLOZ: clozapine; CPZ: chlorpromazine; LEV: levomepromazine; MINOC: minocycline; P.I.: prediction interval; PIMOZ: pimozide; QUE: quetiapine; RISP: risperidone; SER: sertraline; SMD: standardized mean difference; TE: treatment effect; ZIP: ziprasidone.

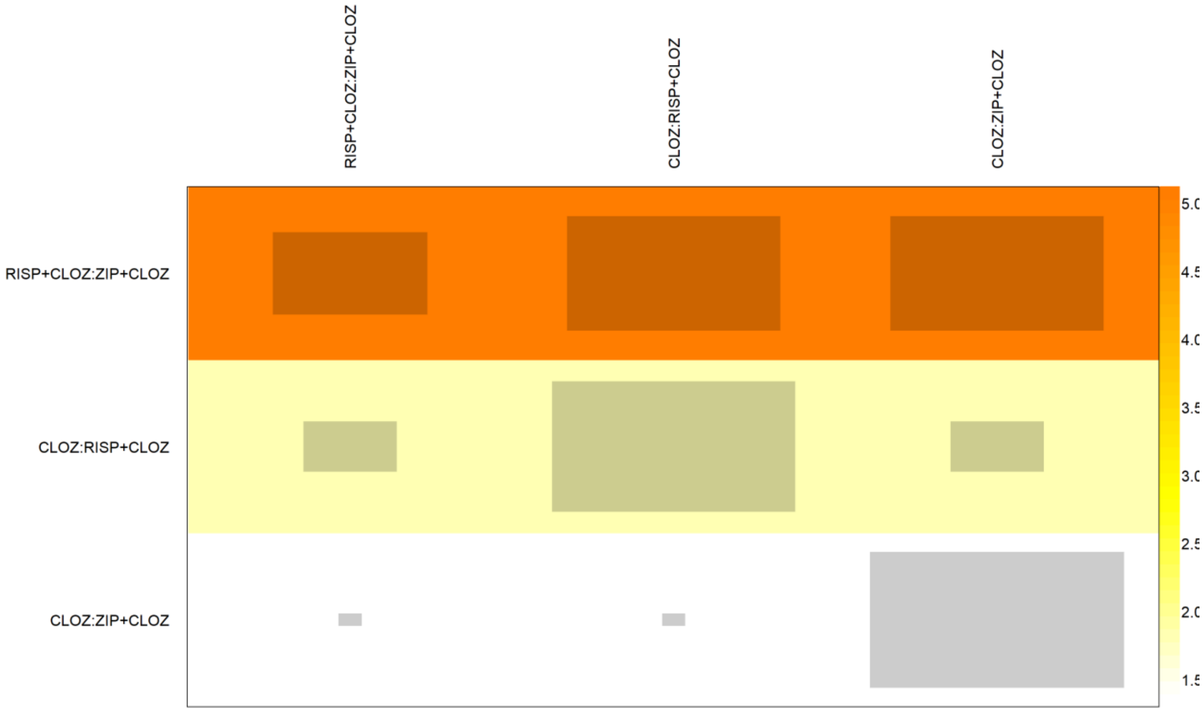

Figure S 10.14c. Net heat plot for the secondary outcome: QTc prolongation
CLOZ: clozapine; RISP: risperidone; ZIP: ziprasidone.

10.15. Quality of life (Figure S 10.15a; Figure S 10.15b; Figure S 10.15c)

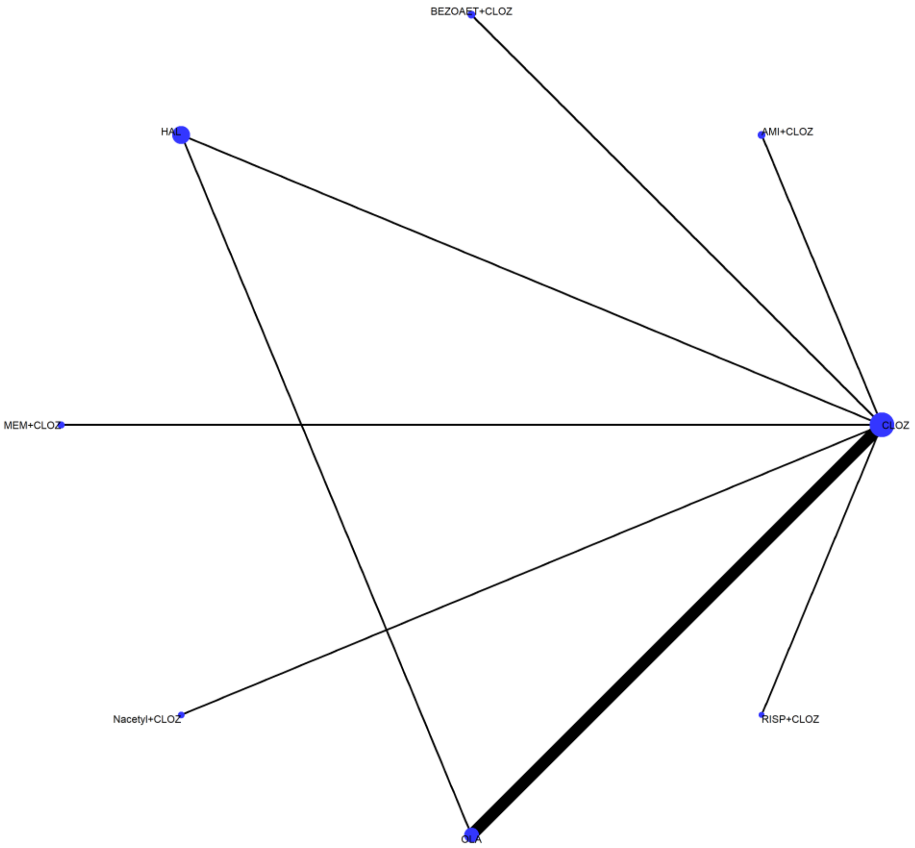

Figure S 10.15a. Network plot for the secondary outcome: Quality of life
AMI: amisulpride; BEZOAET: benzoate sodium; CLOZ: clozapine; HAL: haloperidol; MEM: memantine; Nacetyl: N-acetylcysteine; OLA: olanzapine; RISP: risperidone.

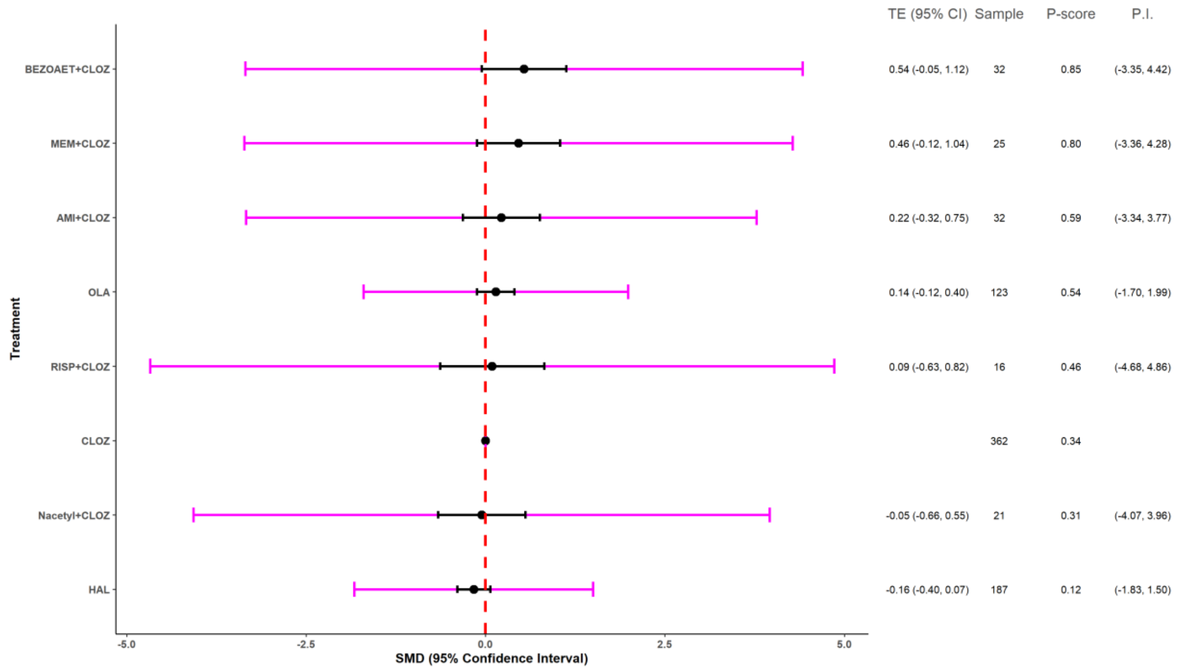

Figure S 10.15b. Forest plot with prediction intervals for the secondary outcome: Quality of life
Reference comparator: Clozapine. AMI: amisulpride; BEZOAET: benzoate sodium; CI: confidence intervals; CLOZ: clozapine; HAL: haloperidol; MEM: memantine; Nacetyl: N-acetylcysteine; OLA: olanzapine; P.I.: prediction interval; RISP: risperidone; SMD: standardized mean difference; TE: treatment effect.

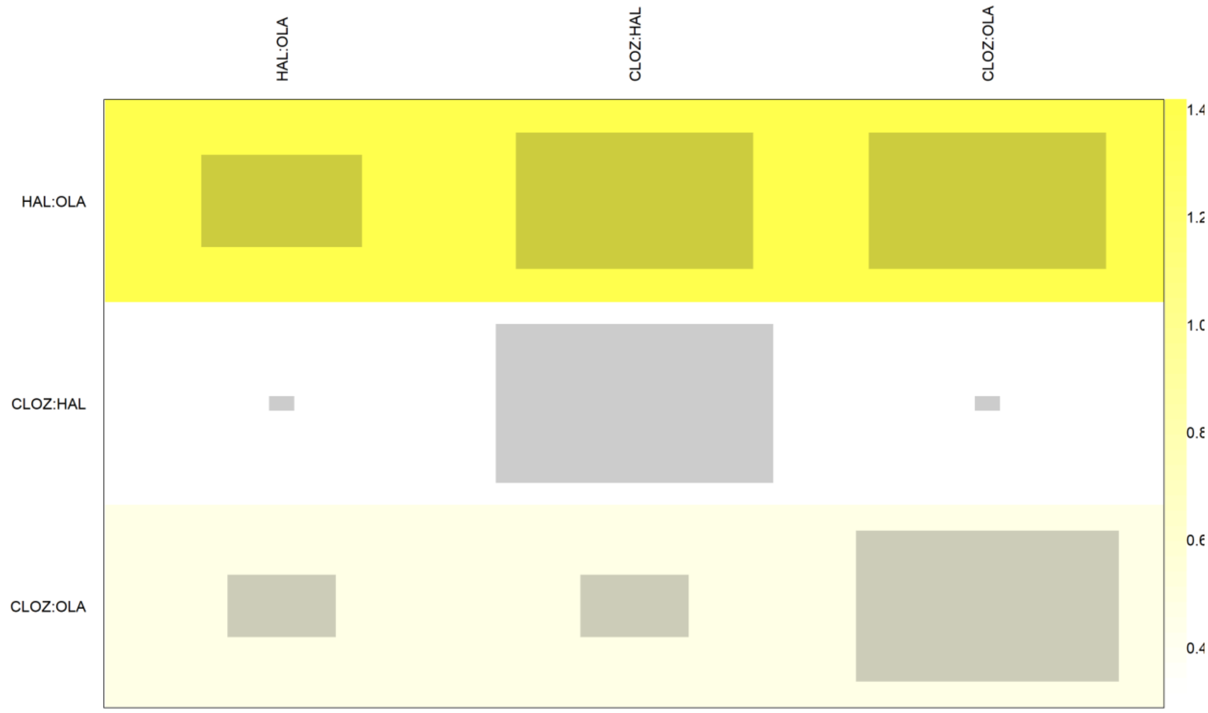

Figure S 10.15c. Net heat plot for the secondary outcome: Quality of life
CLOZ: clozapine; HAL: haloperidol; OLA: olanzapine.

10.16. Social functioning (Figure S 10.16a; Figure S 10.16b; Figure S 10.16c)

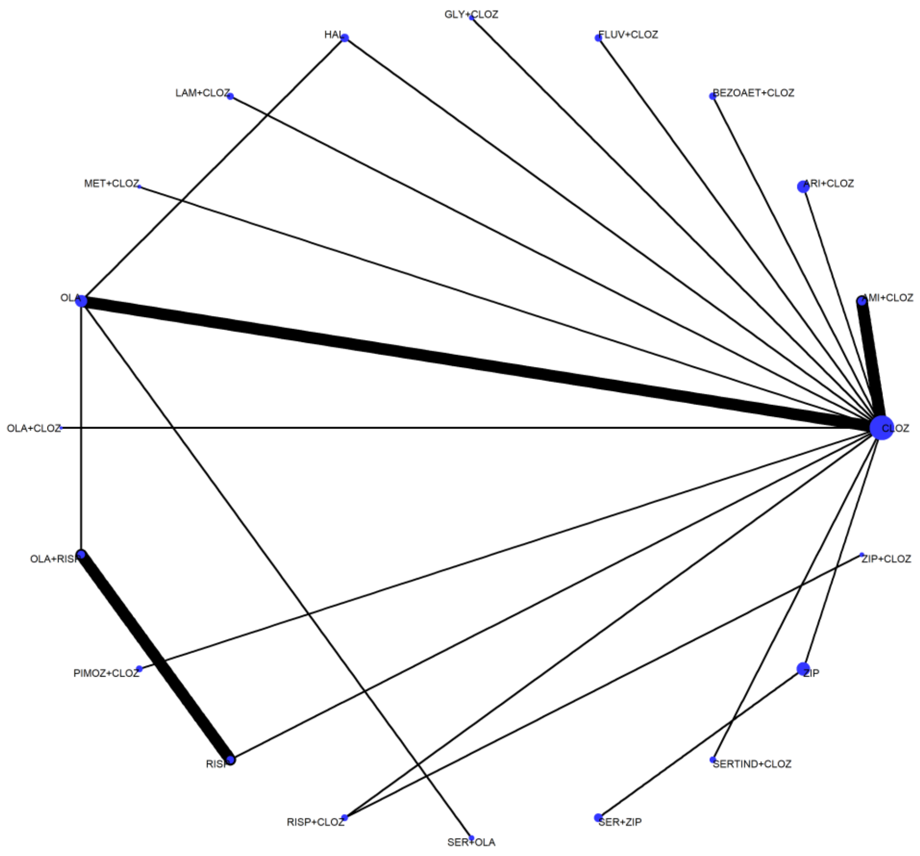

Figure S 10.16a. Network plot for the secondary outcome: Social functioning
AMI: amisulpride; ARI: aripiprazole; BEZOAET: benzoate sodium; CLOZ: clozapine; FLUV: fluvoxamine; GinkBil: Ginkgo biloba; GLY: glycine; HAL: haloperidol; LAM: lamotrigine; MET: metformin; OLA: olanzapine; PIMOZ: pimozide; RISP: risperidone; SER: sertraline; ZIP: ziprasidone.

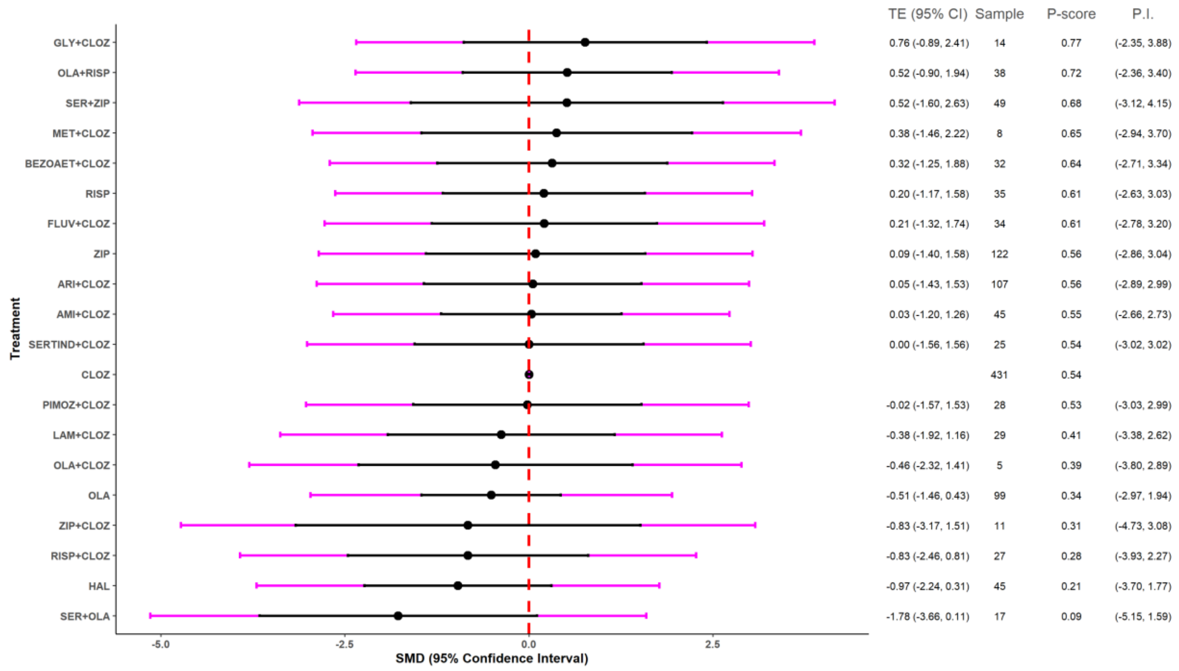

Figure S 10.16b. Forest plot with prediction intervals for the secondary outcome: Social functioning
Reference comparator: Clozapine. AMI: amisulpride; ARI: aripiprazole; BEZOAET: benzoate sodium; CI: confidence intervals; CLOZ: clozapine; FLUV: fluvoxamine; GinkBil: Ginkgo biloba; GLY: glycine; HAL: haloperidol; LAM: lamotrigine; MET: metformin; OLA: olanzapine; P.I.: prediction interval; PIMOZ: pimozide; RISP: risperidone; SER: sertraline; SMD: standardized mean difference; TE: treatment effect; ZIP: ziprasidone.

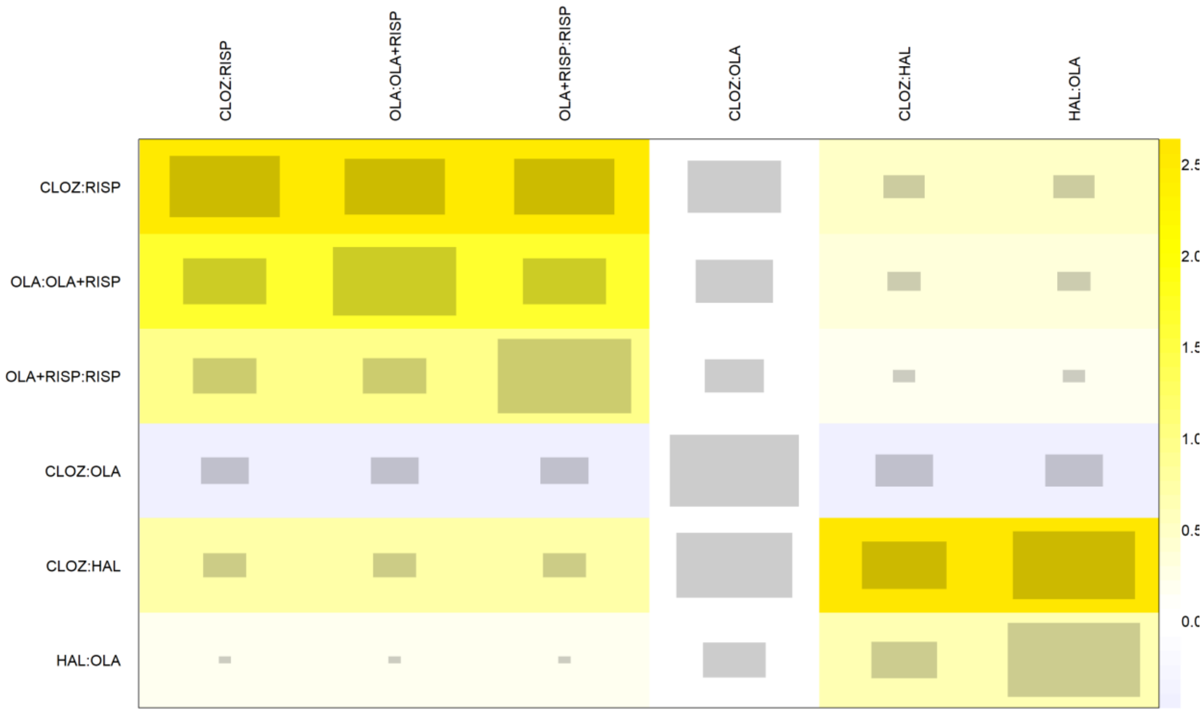

Figure S 10.16c. Net heat plot for the secondary outcome: Social functioning
CLOZ: clozapine; HAL: haloperidol; OLA: olanzapine; RISP: risperidone.

Appendix 11. League tables: Primary and secondary outcomes

The league tables are organized with treatment options listed alphabetically. To interpret the comparisons between treatments, read from left to right; the corresponding estimate is located in the intersecting cell between the column-defining and row-defining treatments. Continuous outcomes are expressed as SMDs, while dichotomous outcomes are presented as ORs. Additional details about the outcomes in this NMA, including whether they are continuous or dichotomous and their league table interpretation when expressed as SMDs or ORs, are provided in the table (Table S 4) below.

**Continuous outcomes presented as SMDs:**	**Dichotomous outcomes presented as ORs**
(11.1) Overall symptoms<br>(11.3) Positive symptoms<br>(11.4) Negative symptoms<br>(11.5) Depressive symptoms<br>(11.12) Weight gain<br>(11.13) Prolactin elevation<br>(11.14) QTc prolongation<br>(11.15) Quality of life<br>(11.6) Social functioning	(11.2) Response rates<br>(11.6) Drop-outs due to any reason<br>(11.7) Drop-outs due to any adverse effect<br>(11.8) Drop-outs due to inefficacy<br>(11.9) Total number of participants expressing adverse effects<br>(11.10) Antiparkinsonian medication use<br>(11.11) Sedation
**Interpretation:**<br>For the outcomes overall symptoms, positive, negative and depressive symptoms, weight, prolactin and QTc: In the left lower half, SMDs lower than 0 favor the column-defining treatment, in the upper right half SMDs lower than 0 favor the row defining treatment.<br>For the outcomes quality of life and functioning: In the left lower half, SMDs higher than 0 favor the column-defining treatment, in the upper right half SMDs higher than 0 favor the row defining treatment.	**Interpretation:**<br>For the outcome response rates: In the left lower half, ORs higher than 1 favor the column-defining treatment; in the upper right half, ORs lower than 1 favor the column-defining treatment.<br>For the remaining outcomes: In the left lower half, ORs lower than 1 favor the column-defining treatment; in the upper right half, ORs higher than 1 favor the column-defining treatment.

Table S 11. League table interpretation based on the type of outcome
OR: odd ratio; SMD: standardized mean difference

11.1. League table: Overall symptoms

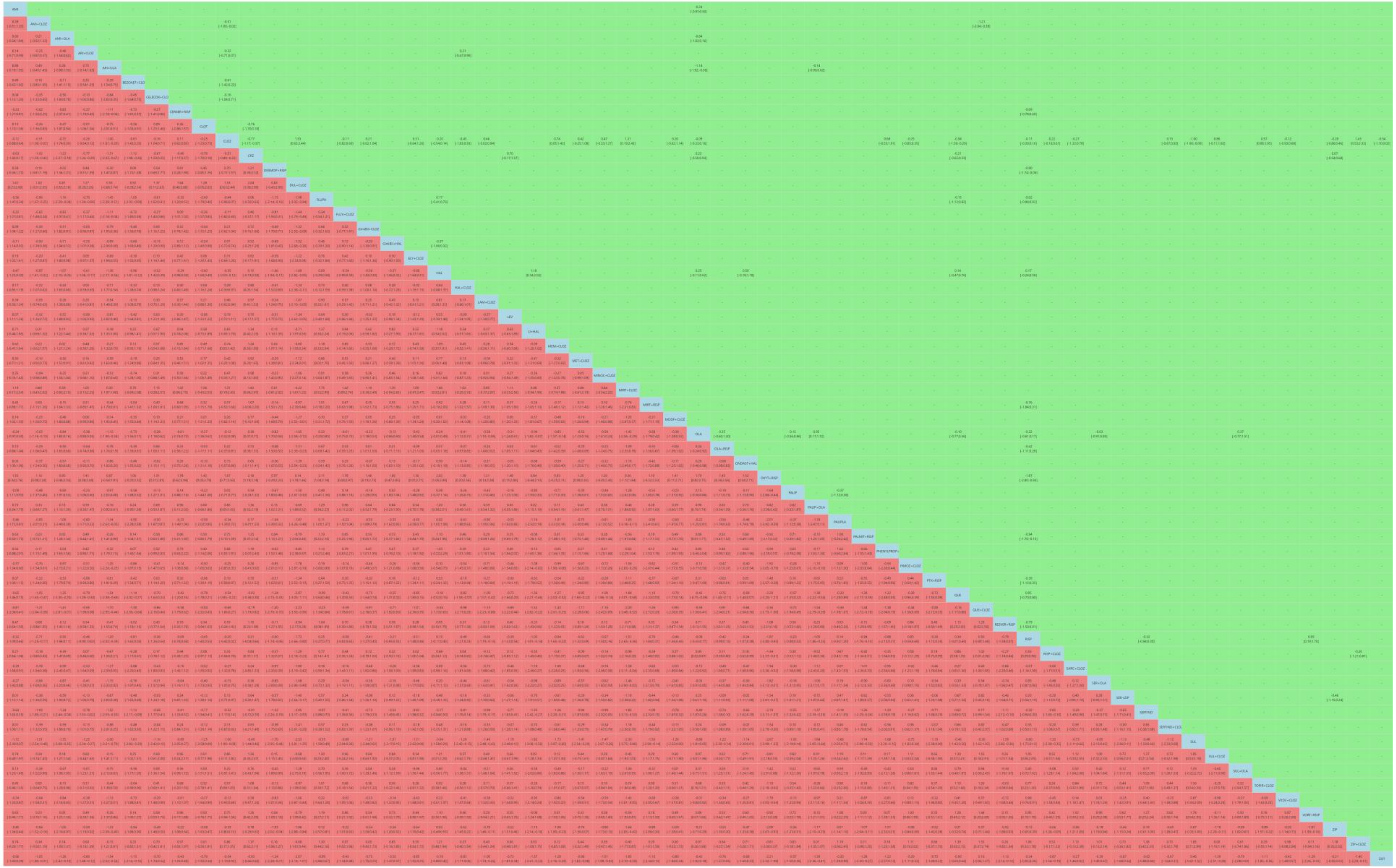

Table S 11.1. League table: Overall symptoms

AMI: amisulpride; ARI: aripiprazole; BEZOAET: benzoate sodium; CELECOX: celecoxib; CEREBR: cerebrolysin; CLOT: clotiapine; CLOZ: clozapine; CPZ: chlorpromazine; DESMOP: desmopressin; DUL: duloxetine; FLUO: fluoxetine; FLUPH: fluphenazine; FLUV: Fluvoxamine; GinkBil: Ginkgo biloba; GLY: glycine; HAL: haloperidol; LAM: lamotrigine; LEV: levomepromazine; LI: lithium; MEM: memantine; MET: metformin; MINOC: minocycline; MIRT: mirtazapine; MODF: modafinil; ONDAST: ondansetron; OLA: olanzapine; OXYT: Oxytocin; PALIP: paliperidone; PALIPLA: paliperidone long-acting; PALMIT: palmitoylethanolamide; PBO: placebo; PHENYLPROP: phenylpropanolamine; PIMOZ: pimozide; PTX: pentoxifylline; QUE: quetiapine; RESVER: resveratrol; RISP: risperidone; SARC: sarcosine; SER: sertraline; SERTIND: sertindole; SUL: sulpiride; TOPIR: topiramate; VitB6: vitamin B6; VitD3: vitamin D3; VORT: vortioxetine; ZIP: ziprasidone; ZOT: zotepine.

11.2. League table: Positive symptoms

AMI	-	-	-	-	-	-	-	-	-	[illegible]
0.98 [-0.06;2.02]	AMI+CLOZ	-	-	-	-	-	-1.13 [-1.91;-0.34]	-	-	[illegible]
0.74 [-0.42;1.91]	-0.24 [-1.49;1.02]	AMI+OLA	-	-	-	-	-	-	-	[illegible]
0.11 [-0.67;0.90]	-0.87 [-1.74;0.01]	-0.63 [-1.68;0.42]	ARI+CLOZ	-	-	-	-0.26 [-0.65;0.12]	-	-	[illegible]
0.25 [-0.76;1.26]	-0.73 [-1.81;0.35]	-0.49 [-1.72;0.73]	0.14 [-0.70;0.97]	BEZOAET+CLO	-	-	-0.40 [-1.14;0.34]	-	-	[illegible]
0.07 [-0.99;1.14]	-0.91 [-2.04;0.22]	-0.67 [-1.94;0.60]	-0.04 [-0.94;0.86]	-0.18 [-1.27;0.92]	CELECOX+CLO	-	-0.22 [-1.03;0.59]	-	-	[illegible]
-0.03 [-0.96;0.91]	-1.01 [-2.02;0.01]	-0.77 [-1.93;0.40]	-0.14 [-0.89;0.61]	-0.28 [-1.26;0.70]	-0.10 [-1.14;0.94]	CEREBR+RISP	-	-	-	[illegible]
-0.15 [-0.83;0.54]	-1.13 [-1.91;-0.34]	-0.89 [-1.87;0.08]	-0.26 [-0.65;0.12]	-0.40 [-1.14;0.34]	-0.22 [-1.03;0.59]	-0.12 [-0.77;0.52]	CLOZ	-0.62 [-0.97;-0.27]	-	[illegible]
-0.66 [-1.38;0.07]	-1.64 [-2.46;-0.81]	-1.40 [-2.40;-0.38]	-0.77 [-1.24;-0.30]	-0.91 [-1.68;-0.12]	-0.73 [-1.58;0.12]	-0.63 [-1.32;0.06]	-0.51 [-0.77;-0.24]	CPZ	-	[illegible]
0.16 [-0.88;1.19]	-0.82 [-1.93;0.29]	-0.59 [-1.83;0.66]	0.04 [-0.83;0.92]	-0.09 [-1.17;0.99]	0.08 [-1.05;1.21]	0.18 [-0.79;1.15]	0.30 [-0.48;1.09]	0.81 [-0.01;1.64]	DESMOP+RISP	[illegible]
-0.17 [-1.38;1.04]	-1.15 [-2.41;0.12]	-0.91 [-2.30;0.48]	-0.28 [-1.35;0.78]	-0.42 [-1.66;0.82]	-0.24 [-1.53;1.04]	-0.14 [-1.33;1.04]	-0.02 [-1.01;0.97]	0.49 [-0.54;1.51]	-0.33 [-1.59;0.94]	[illegible]

[illegible]

Table S 11.2. League table: Positive symptoms

AMI: amisulpride; ARI: aripiprazole; BEZOAET: benzoate sodium; CELECOX: celecoxib; CEREBR: cerebrolysin; CLOZ: clozapine; CPZ: chlorpromazine; Dcyclos: D-cycloserine; DESMOP: desmopressin; Dser: D-serine; DUL: duloxetine; FLUO: fluoxetine; FLUPH: fluphenazine; FLUPHLA: fluphenazine decanoate; FLUV: fluvoxamine; GinkBil: Ginkgo biloba; GLY: glycine; HAL: haloperidol; LAM: lamotrigine; LEV: levomepromazine; MAZIN: mazindol; MEM: memantine; MINOC: minocycline; MIRT: mirtazapine; MODF: modafinil; Nacetyl: N-acetylcysteine; OLA: olanzapine; ONDAST: ondansetron; OXYT: oxytocin; PALMIT: palmitoylethanolamide; PIMOZ: pimozide; PTX: pentoxifylline; QUE: quetiapine; RESVER: resveratrol; RISP: risperidone; SARC: sarcosine; SER: sertraline; SERTIND: sertindole; SUL: sulpiride; TOPIR: topiramate; VitD3: vitamin D3; VORT: vortioxetine; ZIP: ziprasidone

11.3. League table: Negative symptoms

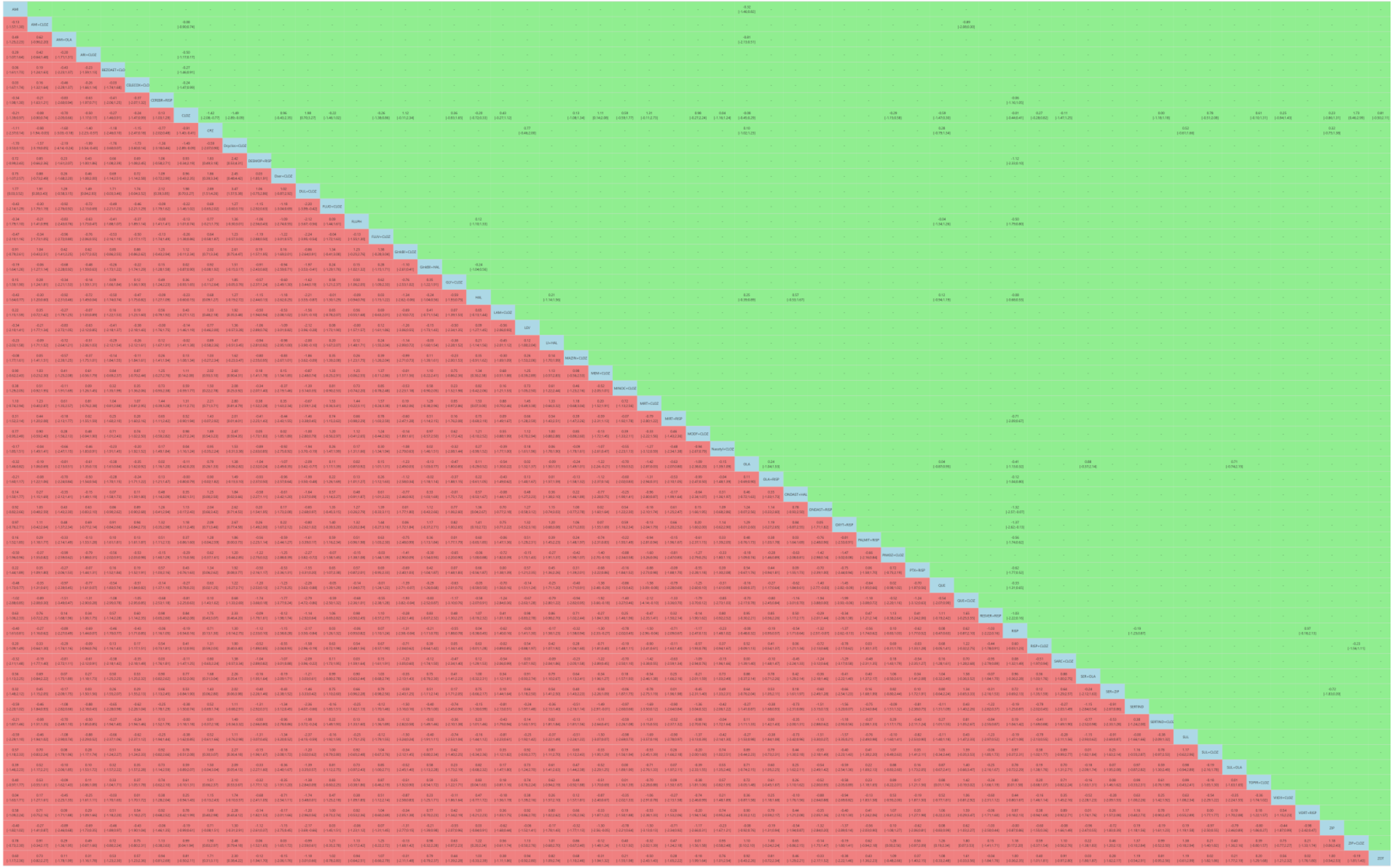

Table S 11.3. League table: Negative symptoms

AMI: amisulpride; ARI: aripiprazole; BEZOAET: benzoate sodium; CELECOX: celecoxib; CEREBR: cerebrolysin; CLOZ: clozapine; CPZ: chlorpromazine; Dcyclos: D-cycloserine; DESMOP: desmopressin; Dser: D-serine; DUL: duloxetine; FLUO: fluoxetine; FLUPH: fluphenazine; FLUPHLA: fluphenazine decanoate; FLUV: fluvoxamine; GinkBil: Ginkgo biloba; GLY: glycine; HAL: haloperidol; LAM: lamotrigine; LEV: levomepromazine; LI: lithium MAZIN: mazindol; MEM: memantine; MINOC: minocycline; MIRT: mirtazapine; MODF: modafinil; Nacetyl: N-acetylcysteine; OLA: olanzapine; ONDAST: ondansetron; OXYT: oxytocin; PALMIT: palmitoylethanolamide; PIMOZ: pimozide; PTX: pentoxifylline; QUE: quetiapine; RESVER: resveratrol; RISP: risperidone; SARC: sarcosine; SER: sertraline; SERTIND: sertindole; SUL: sulpiride; TOPIR: topiramate; VitD3: vitamin D3; VORT: vortioxetine; ZIP: ziprasidone; ZOT: zotepine

11.4. League table: Depressive symptoms

AMI+CLOZ	-	-	0.01 [-0.56;0.59]	-	-	-	-	-	-	-	-	-	-	-	-	-	-	-	-	-	-	-	-	-	-	-	-	-	-	-	-	-
0.45 [-0.32;1.22]	ARI+CLOZ	-	-0.44 [-0.95;0.07]	-	-	-	-	-	-	-	-	-	-	-	-	-	-	-	-	-	-	-	-	-	-	-	-	-	-	-	-	-
-0.02 [-0.92;0.89]	-0.47 [-1.34;0.40]	BEZOAET+CLO	0.03 [-0.67;0.73]	-	-	-	-	-	-	-	-	-	-	-	-	-	-	-	-	-	-	-	-	-	-	-	-	-	-	-	-	-
0.01 [-0.56;0.59]	-0.44 [-0.95;0.07]	0.03 [-0.67;0.73]	CLOZ	-0.45 [-1.00;0.09]	-	1.42 [0.61;2.22]	-0.07 [-0.86;0.73]	-	0.08 [-0.51;0.67]	-0.64 [-1.32;0.04]	0.31 [-0.22;0.84]	-	0.08 [-0.61;0.78]	0.16 [-0.53;0.85]	0.50 [-0.48;1.48]	0.50 [-0.23;1.23]	-0.02 [-0.62;0.59]	-	-	-	-	-0.16 [-0.52;0.19]	0.28 [-0.18;0.74]	0.05 [-0.92;1.02]	-	-	0.41 [-0.45;1.27]	-	0.29 [-0.44;1.02]	-0.13 [-0.83;0.57]	0.19 [-0.33;0.72]	0.69 [-0.07;1.45]
-0.16 [-0.82;0.51]	-0.61 [-1.22;0.00]	-0.14 [-0.92;0.64]	-0.17 [-0.50;0.17]	CPZ	-	-	-	-	-	-	-	0.39 [-0.37;1.15]	-	-	-	-	0.33 [-0.27;0.93]	-	-	-	-0.13 [-0.61;0.35]	-	-	-	-	-	-	-	-	-	-0.22 [-0.69;0.24]	-
-0.73 [-1.70;0.24]	-1.18 [-2.12;-0.25]	-0.71 [-1.76;0.34]	-0.74 [-1.52;0.04]	-0.57 [-1.40;0.25]	DESMOP+RISP	-	-	-	-	-	-	-	-	-	-	-	-	-	-	-	-	0.53 [-0.20;1.26]	-	-	-	-	-	-	-	-	-	-
1.43 [0.44;2.42]	0.98 [0.02;1.93]	1.45 [0.38;2.52]	1.42 [0.61;2.22]	1.59 [0.71;2.46]	2.16 [1.04;3.28]	DUL+CLOZ	-	-	-	-	-	-	-	-	-	-	-	-	-	-	-	-	-	-	-	-	-	-	-	-	-	-
-0.05 [-1.04;0.93]	-0.51 [-1.46;0.44]	-0.04 [-1.10;1.03]	-0.07 [-0.86;0.73]	0.10 [-0.76;0.97]	0.68 [-0.44;1.80]	-1.48 [-2.62;-0.35]	FLUO+CLOZ	-	-	-	-	-	-	-	-	-	-	-	-	-	-	-	-	-	-	-	-	-	-	-	-	-
-0.25 [-1.24;0.75]	-0.70 [-1.66;0.26]	-0.23 [-1.30;0.84]	-0.26 [-1.07;0.55]	-0.09 [-0.91;0.73]	0.49 [-0.58;1.56]	-1.68 [-2.82;-0.53]	-0.19 [-1.33;0.95]	FLUPH	-	-	-	-	-	-	-	-	-	-	-	-	0.06 [-0.83;0.94]	0.01 [-0.86;0.88]	-	-	-	-	-	-	-	-	-	-
0.09 [-0.73;0.92]	-0.36 [-1.14;0.42]	0.11 [-0.80;1.03]	0.08 [-0.51;0.67]	0.25 [-0.43;0.93]	0.82 [-0.16;1.80]	-1.34 [-2.34;-0.34]	0.15 [-0.85;1.14]	0.34 [-0.66;1.34]	FLUV+CLOZ	-	-	-	-	-	-	-	-	-	-	-	-	-	-	-	-	-	-	-	-	-	-	-
-0.35 [-1.02;0.32]	-0.80 [-1.42;-0.18]	-0.33 [-1.11;0.45]	-0.36 [-0.70;-0.02]	-0.19 [-0.58;0.20]	0.38 [-0.42;1.18]	-1.78 [-2.65;-0.90]	-0.29 [-1.16;0.58]	-0.10 [-0.91;0.71]	-0.44 [-1.12;0.24]	HAL	-	-	-	-	-	-	0.28 [-0.08;0.65]	-	-	-	0.16 [-0.31;0.64]	0.00 [-0.66;0.66]	-	-	-	-	-	-	-	-	-	-
0.32 [-0.46;1.10]	-0.13 [-0.87;0.61]	0.34 [-0.54;1.22]	0.31 [-0.22;0.84]	0.48 [-0.15;1.11]	1.05 [0.11;2.00]	-1.11 [-2.07;-0.14]	0.38 [-0.58;1.33]	0.57 [-0.40;1.54]	0.23 [-0.56;1.02]	0.67 [0.04;1.30]	LAM+CLOZ	-	-	-	-	-	-	-	-	-	-	-	-	-	-	-	-	-	-	-	-	-
0.24 [-0.78;1.25]	-0.22 [-1.20;0.76]	0.25 [-0.83;1.34]	0.22 [-0.61;1.06]	0.39 [-0.37;1.15]	0.97 [-0.15;2.09]	-1.19 [-2.35;-0.04]	0.29 [-0.86;1.44]	0.48 [-0.64;1.60]	0.14 [-0.88;1.16]	0.58 [-0.27;1.44]	-0.09 [-1.07;0.90]	LEV	-	-	-	-	-	-	-	-	-	-	-	-	-	-	-	-	-	-	-	-
0.09 [-0.81;1.00]	-0.36 [-1.22;0.51]	0.11 [-0.87;1.10]	0.08 [-0.61;0.78]	0.25 [-0.52;1.02]	0.83 [-0.22;1.87]	-1.34 [-2.40;-0.27]	0.15 [-0.91;1.21]	0.34 [-0.73;1.41]	0.00 [-0.91;0.91]	0.44 [-0.33;1.22]	-0.23 [-1.10;0.64]	-0.14 [-1.23;0.94]	MEM+CLOZ	-	-	-	-	-	-	-	-	-	-	-	-	-	-	-	-	-	-	-
0.17 [-0.73;1.07]	-0.28 [-1.14;0.58]	0.19 [-0.79;1.18]	0.16 [-0.53;0.85]	0.33 [-0.44;1.10]	0.90 [-0.14;1.95]	-1.26 [-2.32;-0.20]	0.23 [-0.83;1.28]	0.42 [-0.65;1.48]	0.08 [-0.83;0.99]	0.52 [-0.25;1.29]	-0.15 [-1.02;0.72]	-0.06 [-1.14;1.02]	0.08 [-0.90;1.06]	MINOC+CLOZ	-	-	-	-	-	-	-	-	-	-	-	-	-	-	-	-	-	-
0.51 [-0.62;1.65]	0.06 [-1.05;1.16]	0.53 [-0.68;1.73]	0.50 [-0.48;1.48]	0.67 [-0.37;1.70]	1.24 [-0.01;2.50]	-0.92 [-2.19;0.35]	0.57 [-0.70;1.83]	0.76 [-0.51;2.03]	0.42 [-0.73;1.56]	0.86 [-0.18;1.90]	0.19 [-0.92;1.30]	0.28 [-1.01;1.56]	0.42 [-0.78;1.62]	0.34 [-0.86;1.54]	MIRT+CLOZ	-	-	-	-	-	-	-	-	-	-	-	-	-	-	-	-	-
0.51 [-0.42;1.44]	0.06 [-0.84;0.95]	0.53 [-0.48;1.54]	0.50 [-0.23;1.23]	0.67 [-0.14;1.47]	1.24 [0.17;2.31]	-0.92 [-2.01;0.17]	0.56 [-0.52;1.65]	0.76 [-0.34;1.85]	0.42 [-0.52;1.36]	0.86 [0.05;1.67]	0.19 [-0.72;1.09]	0.28 [-0.83;1.38]	0.42 [-0.59;1.43]	0.34 [-0.67;1.35]	-0.00 [-1.22;1.22]	Nacetyl+CLOZ	-	-	-	-	-	-	-	-	-	-	-	-	-	-	-	-
-0.00 [-0.65;0.65]	-0.45 [-1.05;0.15]	0.02 [-0.75;0.78]	-0.01 [-0.32;0.30]	0.16 [-0.21;0.52]	0.73 [-0.05;1.51]	-1.43 [-2.30;-0.57]	0.05 [-0.80;0.91]	0.24 [-0.56;1.05]	-0.09 [-0.76;0.57]	0.35 [0.05;0.64]	-0.32 [-0.94;0.29]	-0.24 [-1.08;0.61]	-0.10 [-0.86;0.67]	-0.17 [-0.93;0.58]	-0.51 [-1.54;0.52]	-0.51 [-1.31;0.28]	OLA	0.13 [-0.72;0.99]	-	-	-	-0.24 [-0.64;0.16]	-	-	0.75 [-0.05;1.56]	-	-	1.06 [-0.06;2.19]	-	-	-	-
-0.06 [-0.95;0.83]	-0.51 [-1.36;0.33]	-0.04 [-1.02;0.93]	-0.07 [-0.75;0.60]	0.10 [-0.62;0.81]	0.67 [-0.30;1.64]	-1.49 [-2.54;-0.44]	-0.01 [-1.05;1.04]	0.18 [-0.82;1.19]	-0.16 [-1.05;0.74]	0.29 [-0.40;0.97]	-0.38 [-1.24;0.47]	-0.30 [-1.34;0.74]	-0.16 [-1.13;0.81]	-0.23 [-1.20;0.73]	-0.57 [-1.76;0.62]	-0.57 [-1.57;0.42]	-0.06 [-0.70;0.58]	OLA+RISP	-	-	-	0.07 [-0.83;0.98]	-	-	-	-	-	-	-	-	-	-
-0.41 [-1.41;0.58]	-0.87 [-1.82;0.09]	-0.39 [-1.47;0.68]	-0.42 [-1.23;0.39]	-0.26 [-1.10;0.59]	0.32 [-0.73;1.37]	-1.84 [-2.99;-0.70]	-0.36 [-1.50;0.78]	-0.17 [-1.26;0.92]	-0.51 [-1.51;0.50]	-0.07 [-0.89;0.76]	-0.74 [-1.70;0.23]	-0.65 [-1.79;0.49]	-0.51 [-1.57;0.56]	-0.59 [-1.65;0.48]	-0.92 [-2.20;0.35]	-0.92 [-2.02;0.17]	-0.41 [-1.22;0.40]	-0.35 [-1.34;0.64]	ONDAST+RISP	-	-	0.21 [-0.55;0.97]	-	-	-	-	-	-	-	-	-	-
-0.27 [-1.16;0.63]	-0.72 [-1.58;0.14]	-0.25 [-1.23;0.73]	-0.28 [-0.97;0.41]	-0.11 [-0.84;0.62]	0.46 [-0.50;1.42]	-1.70 [-2.76;-0.64]	-0.22 [-1.27;0.84]	-0.02 [-1.03;0.98]	-0.36 [-1.27;0.54]	0.08 [-0.63;0.79]	-0.59 [-1.46;0.27]	-0.50 [-1.56;0.55]	-0.36 [-1.34;0.61]	-0.44 [-1.42;0.53]	-0.78 [-1.98;0.42]	-0.78 [-1.78;0.22]	-0.27 [-0.96;0.42]	-0.21 [-1.10;0.69]	0.14 [-0.84;1.13]	PTX+RISP	-	0.07 [-0.56;0.69]	-	-	-	-	-	-	-	-	-	-
-0.22 [-0.93;0.48]	-0.68 [-1.33;-0.02]	-0.21 [-1.02;0.61]	-0.24 [-0.64;0.17]	-0.07 [-0.44;0.31]	0.51 [-0.33;1.34]	-1.65 [-2.56;-0.75]	-0.17 [-1.07;0.73]	0.02 [-0.77;0.81]	-0.32 [-1.04;0.40]	0.12 [-0.24;0.49]	-0.55 [-1.22;0.12]	-0.46 [-1.31;0.39]	-0.32 [-1.12;0.49]	-0.40 [-1.20;0.41]	-0.74 [-1.80;0.33]	-0.73 [-1.57;0.10]	-0.22 [-0.62;0.18]	-0.16 [-0.89;0.57]	0.19 [-0.67;1.05]	0.04 [-0.70;0.79]	QUE	-0.04 [-0.92;0.85]	-	-	-	-	-	-	-	-	-	-
-0.20 [-0.84;0.44]	-0.65 [-1.24;-0.07]	-0.18 [-0.94;0.57]	-0.21 [-0.50;0.07]	-0.04 [-0.43;0.34]	0.53 [-0.20;1.26]	-1.63 [-2.49;-0.78]	-0.15 [-1.00;0.70]	0.04 [-0.74;0.83]	-0.30 [-0.95;0.36]	0.15 [-0.19;0.48]	-0.52 [-1.12;0.08]	-0.44 [-1.29;0.41]	-0.30 [-1.05;0.45]	-0.37 [-1.12;0.37]	-0.71 [-1.73;0.31]	-0.71 [-1.50;0.07]	-0.20 [-0.49;0.08]	-0.14 [-0.78;0.50]	0.21 [-0.55;0.97]	0.07 [-0.56;0.69]	0.02 [-0.39;0.43]	RISP	-	-	-	-	-	-	-	-	-	-
0.44 [-0.27;1.16]	-0.01 [-0.68;0.66]	0.46 [-0.36;1.28]	0.43 [0.00;0.86]	0.60 [0.05;1.15]	1.17 [0.28;2.07]	-0.99 [-1.90;-0.07]	0.50 [-0.41;1.40]	0.69 [-0.23;1.61]	0.35 [-0.38;1.08]	0.79 [0.24;1.34]	0.12 [-0.56;0.80]	0.21 [-0.73;1.14]	0.35 [-0.47;1.16]	0.27 [-0.54;1.08]	-0.07 [-1.14;1.00]	-0.07 [-0.92;0.78]	0.44 [-0.09;0.97]	0.50 [-0.30;1.31]	0.85 [-0.06;1.77]	0.71 [-0.10;1.52]	0.67 [0.07;1.26]	0.64 [0.13;1.16]	RISP+CLOZ	-	-	-	-	-	-	-	-	-0.83 [-1.79;0.13]
0.06 [-1.06;1.19]	-0.39 [-1.49;0.71]	0.08 [-1.11;1.28]	0.05 [-0.92;1.02]	0.22 [-0.80;1.24]	0.79 [-0.45;2.04]	-1.37 [-2.63;-0.11]	0.12 [-1.14;1.37]	0.31 [-0.95;1.57]	-0.03 [-1.16;1.10]	0.41 [-0.62;1.44]	-0.26 [-1.36;0.84]	-0.17 [-1.45;1.10]	-0.03 [-1.22;1.16]	-0.11 [-1.30;1.08]	-0.45 [-1.83;0.93]	-0.45 [-1.66;0.77]	0.06 [-0.95;1.08]	0.13 [-1.06;1.31]	0.48 [-0.79;1.74]	0.33 [-0.85;1.52]	0.29 [-0.76;1.34]	0.27 [-0.74;1.27]	-0.38 [-1.44;0.68]	SARC+CLOZ	-	-	-	-	-	-	-	-
0.75 [-0.29;1.79]	0.30 [-0.71;1.31]	0.77 [-0.34;1.88]	0.74 [-0.12;1.61]	0.91 [0.02;1.80]	1.48 [0.36;2.61]	-0.68 [-1.86;0.51]	0.81 [-0.37;1.98]	1.00 [-0.14;2.14]	0.66 [-0.39;1.71]	1.10 [0.24;1.96]	0.43 [-0.58;1.44]	0.52 [-0.65;1.69]	0.66 [-0.45;1.77]	0.58 [-0.53;1.69]	0.24 [-1.07;1.55]	0.24 [-0.89;1.38]	0.75 [-0.05;1.56]	0.81 [-0.21;1.84]	1.17 [0.02;2.31]	1.02 [-0.04;2.08]	0.98 [0.08;1.88]	0.95 [0.10;1.81]	0.31 [-0.66;1.28]	0.69 [-0.61;1.99]	SER+OLA	-	-	-	-	-	-	-
0.57 [-0.33;1.48]	0.12 [-0.75;0.99]	0.59 [-0.40;1.58]	0.56 [-0.14;1.26]	0.73 [0.04;1.42]	1.30 [0.27;2.34]	-0.86 [-1.92;0.21]	0.63 [-0.44;1.69]	0.82 [-0.23;1.86]	0.48 [-0.44;1.39]	0.92 [0.17;1.67]	0.25 [-0.63;1.13]	0.34 [-0.69;1.36]	0.48 [-0.51;1.46]	0.40 [-0.58;1.38]	0.06 [-1.14;1.26]	0.06 [-0.95;1.07]	0.57 [-0.16;1.31]	0.63 [-0.32;1.59]	0.98 [-0.07;2.04]	0.84 [-0.12;1.81]	0.80 [0.04;1.56]	0.77 [0.04;1.51]	0.13 [-0.69;0.95]	0.51 [-0.68;1.70]	-0.18 [-1.27;0.91]	SER+ZIP	-	-	-	-	-0.69 [-1.27;-0.12]	-
0.42 [-0.61;1.46]	-0.03 [-1.03;0.97]	0.44 [-0.67;1.55]	0.41 [-0.45;1.27]	0.58 [-0.34;1.50]	1.16 [-0.01;2.32]	-1.01 [-2.18;0.17]	0.48 [-0.70;1.65]	0.67 [-0.51;1.85]	0.33 [-0.71;1.37]	0.77 [-0.16;1.70]	0.10 [-0.91;1.11]	0.19 [-1.01;1.39]	0.33 [-0.78;1.44]	0.25 [-0.85;1.35]	-0.09 [-1.39;1.22]	-0.09 [-1.22;1.04]	0.43 [-0.49;1.34]	0.49 [-0.61;1.58]	0.84 [-0.35;2.02]	0.69 [-0.41;1.79]	0.65 [-0.30;1.60]	0.63 [-0.28;1.53]	-0.02 [-0.98;0.94]	0.36 [-0.93;1.66]	-0.33 [-1.55;0.89]	-0.15 [-1.26;0.96]	SUL+CLOZ	-	-	-	-	-
1.06 [-0.24;2.36]	0.61 [-0.66;1.89]	1.08 [-0.28;2.44]	1.05 [-0.11;2.22]	1.22 [0.04;2.40]	1.79 [0.43;3.16]	-0.37 [-1.78;1.05]	1.12 [-0.30;2.53]	1.31 [-0.08;2.69]	0.97 [-0.34;2.28]	1.41 [0.25;2.57]	0.74 [-0.54;2.02]	0.83 [-0.58;2.23]	0.97 [-0.39;2.33]	0.89 [-0.47;2.25]	0.55 [-0.97;2.08]	0.55 [-0.83;1.93]	1.06 [-0.06;2.19]	1.13 [-0.17;2.42]	1.48 [0.09;2.86]	1.33 [0.01;2.65]	1.29 [0.09;2.48]	1.27 [0.11;2.43]	0.62 [-0.62;1.86]	1.00 [-0.52;2.52]	0.31 [-1.07;1.70]	0.49 [-0.85;1.84]	0.64 [-0.81;2.09]	SUL+OLA	-	-	-	-
0.30 [-0.63;1.23]	-0.15 [-1.04;0.74]	0.32 [-0.69;1.33]	0.29 [-0.44;1.02]	0.46 [-0.35;1.26]	1.03 [-0.04;2.10]	-1.13 [-2.22;-0.04]	0.36 [-0.73;1.44]	0.55 [-0.54;1.64]	0.21 [-0.73;1.15]	0.65 [-0.16;1.46]	-0.02 [-0.92;0.88]	0.07 [-1.04;1.17]	0.21 [-0.80;1.22]	0.13 [-0.88;1.14]	-0.21 [-1.43;1.01]	-0.21 [-1.24;0.83]	0.30 [-0.49;1.10]	0.36 [-0.63;1.36]	0.71 [-0.38;1.81]	0.57 [-0.43;1.57]	0.53 [-0.31;1.36]	0.50 [-0.28;1.29]	-0.14 [-0.99;0.71]	0.24 [-0.97;1.45]	-0.45 [-1.58;0.68]	-0.27 [-1.28;0.74]	-0.12 [-1.25;1.01]	-0.76 [-2.14;0.62]	TOPIR+CLOZ	-	-	-
-0.12 [-1.02;0.79]	-0.57 [-1.44;0.30]	-0.10 [-1.09;0.89]	-0.13 [-0.83;0.57]	0.04 [-0.74;0.81]	0.61 [-0.44;1.66]	-1.55 [-2.62;-0.48]	-0.07 [-1.13;1.00]	0.13 [-0.94;1.20]	-0.21 [-1.13;0.70]	0.23 [-0.55;1.01]	-0.44 [-1.32;0.43]	-0.35 [-1.44;0.73]	-0.21 [-1.20;0.77]	-0.29 [-1.28;0.69]	-0.63 [-1.83;0.57]	-0.63 [-1.64;0.38]	-0.12 [-0.88;0.65]	-0.06 [-1.03;0.91]	0.29 [-0.78;1.36]	0.15 [-0.83;1.13]	0.10 [-0.71;0.92]	0.08 [-0.67;0.84]	-0.56 [-1.38;0.26]	-0.18 [-1.38;1.01]	-0.87 [-1.99;0.24]	-0.69 [-1.68;0.30]	-0.54 [-1.65;0.57]	-1.18 [-2.54;0.18]	-0.42 [-1.43;0.59]	VitD3+CLOZ	-	-
-0.12 [-0.82;0.58]	-0.58 [-1.22;0.07]	-0.10 [-0.91;0.70]	-0.13 [-0.53;0.26]	0.03 [-0.35;0.41]	0.61 [-0.25;1.47]	-1.55 [-2.45;-0.65]	-0.07 [-0.96;0.82]	0.12 [-0.75;1.00]	-0.22 [-0.93;0.50]	0.22 [-0.25;0.70]	-0.45 [-1.11;0.22]	-0.36 [-1.21;0.49]	-0.22 [-1.02;0.58]	-0.30 [-1.09;0.50]	-0.63 [-1.69;0.42]	-0.63 [-1.47;0.20]	-0.12 [-0.58;0.34]	-0.06 [-0.82;0.70]	0.29 [-0.60;1.18]	0.15 [-0.63;0.92]	0.10 [-0.39;0.60]	0.08 [-0.38;0.54]	-0.56 [-1.15;0.02]	-0.19 [-1.23;0.86]	-0.88 [-1.80;0.05]	-0.69 [-1.27;-0.12]	-0.55 [-1.49;0.40]	-1.19 [-2.40;0.03]	-0.42 [-1.26;0.41]	-0.00 [-0.81;0.80]	ZIP	-
0.29 [-0.56;1.13]	-0.17 [-0.97;0.64]	0.30 [-0.63;1.24]	0.27 [-0.34;0.89]	0.44 [-0.26;1.15]	1.02 [0.02;2.01]	-1.14 [-2.16;-0.13]	0.34 [-0.67;1.35]	0.53 [-0.49;1.55]	0.19 [-0.66;1.05]	0.63 [-0.07;1.34]	-0.04 [-0.85;0.78]	0.05 [-0.99;1.09]	0.19 [-0.74;1.12]	0.11 [-0.82;1.04]	-0.23 [-1.38;0.93]	-0.23 [-1.18;0.73]	0.29 [-0.41;0.98]	0.35 [-0.57;1.26]	0.70 [-0.32;1.72]	0.55 [-0.37;1.48]	0.51 [-0.23;1.25]	0.49 [-0.19;1.17]	-0.16 [-0.81;0.50]	0.22 [-0.93;1.37]	-0.47 [-1.53;0.60]	-0.29 [-1.22;0.65]	-0.14 [-1.20;0.92]	-0.78 [-2.10;0.54]	-0.02 [-0.97;0.94]	0.40 [-0.53;1.34]	0.41 [-0.33;1.14]	ZIP+CLOZ

Table S 11.4. League table: Depressive symptoms

AMI: amisulpride; ARI: aripiprazole; BEZOAET: benzoate sodium; CLOZ: clozapine; CPZ: chlorpromazine; DESMOP: desmopressin; DUL: duloxetine; FLUO: fluoxetine; FLUPH: fluphenazine; FLUV: fluvoxamine; HAL: haloperidol; LAM: lamotrigine; LEV: levomepromazine; MEM: memantine; MINOC: minocycline; MIRT: mirtazapine; Nacetyl: N-acetylcysteine; OLA: olanzapine; ONDAST: ondansetron; PTX: pentoxifylline; QUE: quetiapine; RESVER: resveratrol; RISP: risperidone; SARC: sarcosine; SER: sertraline; SUL: sulpiride; TOPIR: topiramate; VitD3: vitamin D3; ZIP: ziprasidone

11.5. League table: Response rates

AMI	-	-	-	-	-	-	-	-	-	-	-	-	-	-	-	0.83 [0.24;2.89]	-	-	-	-	-	-	-	-	-	-	-	-	-	-	-	-	-
0.26 [0.05;1.46]	AMI+CLOZ	-	-	2.60 [0.87;7.74]	-	-	-	-	-	-	-	-	-	-	-	-	-	-	-	-	-	-	-	-	-	-	-	-	-	-	-	-	-
0.35 [0.03;3.54]	1.32 [0.14;12.86]	AMI+OLA	-	-	-	-	-	-	-	-	-	-	-	-	-	2.38 [0.34;16.81]	-	-	-	-	-	-	-	-	-	-	-	-	-	-	-	-	-
0.51 [0.08;3.40]	1.94 [0.34;11.09]	1.47 [0.13;16.47]	ARI+CLOZ	1.34 [0.34;5.23]	-	-	-	-	-	-	-	-	-	-	-	-	-	-	-	-	-	-	-	-	-	-	-	-	-	-	-	-	-
0.68 [0.18;2.56]	2.60 [0.87;7.74]	1.97 [0.27;14.52]	1.34 [0.34;5.23]	CLOZ	12.45 [3.66;42.37]	0.24 [0.02;3.53]	-	-	-	2.01 [1.00;4.03]	0.13 [0.01;1.41]	-	-	0.14 [0.01;1.45]	-	1.21 [0.75;1.94]	-	-	-	-	-	-	2.43 [0.40;14.77]	1.18 [0.68;2.05]	0.93 [0.36;2.44]	-	-	1.40 [0.24;8.18]	-	0.14 [0.02;1.05]	-	0.61 [0.20;1.90]	-
1.53 [0.36;6.39]	5.80 [1.65;20.38]	4.39 [0.55;34.94]	2.99 [0.67;13.37]	2.23 [1.19;4.16]	CPZ	-	-	-	-	0.25 [0.03;2.22]	-	0.53 [0.11;2.57]	-	-	-	0.13 [0.01;3.04]	-	-	19.85 [4.03;97.84]	-	-	-	0.86 [0.37;2.01]	5.60 [0.81;38.89]	-	-	0.20 [0.04;0.93]	-	0.62 [0.12;3.21]	-	0.46 [0.08;2.54]	0.90 [0.32;2.49]	-
0.16 [0.01;3.28]	0.62 [0.03;11.36]	0.47 [0.02;13.46]	0.32 [0.02;6.55]	0.24 [0.02;3.53]	0.11 [0.01;1.70]	DONEP+CLOZ	-	-	-	-	-	-	-	-	-	-	-	-	-	-	-	-	-	-	-	-	-	-	-	-	-	-	-
2.41 [0.37;15.56]	9.15 [1.60;52.34]	6.93 [0.63;75.97]	4.72 [0.69;32.33]	3.52 [0.90;13.72]	1.58 [0.38;6.52]	14.78 [0.72;303.24]	FLUPH	-	-	0.49 [0.08;2.84]	-	-	-	-	-	-	-	-	-	-	-	-	0.55 [0.06;4.90]	0.61 [0.07;5.39]	-	-	-	-	-	-	-	-	-
4.61 [0.33;64.77]	17.51 [1.36;225.62]	13.26 [0.63;277.09]	9.03 [0.62;132.03]	6.73 [0.67;67.94]	3.02 [0.31;29.50]	28.28 [0.81;987.10]	1.91 [0.14;26.55]	FLUPHLA	-	-	-	-	-	-	-	-	-	-	2.79 [0.41;18.89]	-	1.00 [0.24;4.20]	-	-	-	-	-	-	-	-	-	-	-	-
0.92 [0.15;5.61]	3.50 [0.65;18.85]	2.65 [0.25;27.74]	1.80 [0.28;11.71]	1.34 [0.37;4.85]	0.60 [0.16;2.34]	5.65 [0.28;112.01]	0.38 [0.06;2.26]	0.20 [0.02;2.65]	GinkBil+HAL	1.61 [0.49;5.27]	-	-	-	-	-	-	-	-	-	-	-	-	-	-	-	-	-	-	-	-	-	-	-
1.48 [0.38;5.77]	5.62 [1.70;18.54]	4.25 [0.56;32.28]	2.90 [0.68;12.29]	2.16 [1.33;3.51]	0.97 [0.50;1.86]	9.07 [0.59;140.65]	0.61 [0.16;2.30]	0.32 [0.03;3.18]	1.61 [0.49;5.27]	HAL	-	0.31 [0.06;1.62]	1.25 [0.12;13.17]	-	-	0.48 [0.22;1.06]	-	-	2.96 [0.47;18.78]	0.40 [0.08;2.00]	-	-	0.57 [0.20;1.59]	0.71 [0.28;1.85]	-	-	-	-	1.96 [0.36;10.62]	-	-	-	-
0.09 [0.01;1.36]	0.34 [0.03;4.67]	0.26 [0.01;5.78]	0.18 [0.01;2.73]	0.13 [0.01;1.41]	0.06 [0.01;0.69]	0.56 [0.02;20.14]	0.04 [0.00;0.58]	0.02 [0.00;0.54]	0.10 [0.01;1.46]	0.06 [0.01;0.69]	LAM+CLOZ	-	-	-	-	-	-	-	-	-	-	-	-	-	-	-	-	-	-	-	-	-	-
0.62 [0.10;3.64]	2.35 [0.45;12.12]	1.78 [0.17;18.14]	1.21 [0.19;7.56]	0.90 [0.26;3.08]	0.40 [0.12;1.32]	3.79 [0.20;73.39]	0.26 [0.04;1.48]	0.13 [0.01;1.69]	0.67 [0.12;3.60]	0.42 [0.13;1.37]	6.82 [0.47;98.50]	LEV	-	-	-	-	-	-	-	1.28 [0.24;6.71]	-	-	-	-	-	-	-	-	-	-	-	-	-
1.65 [0.12;28.05]	7.02 [0.50;98.41]	5.32 [0.24;118.84]	3.62 [0.23;57.37]	2.70 [0.24;29.89]	1.21 [0.11;13.94]	11.34 [0.31;420.69]	0.77 [0.05;11.41]	0.40 [0.01;10.74]	2.01 [0.14;28.08]	1.25 [0.12;13.17]	20.43 [0.70;597.77]	2.99 [0.21;41.84]	LI+HAL	-	-	-	-	-	-	-	-	-	-	-	-	-	-	-	-	-	-	-	-
0.09 [0.01;1.40]	0.35 [0.03;4.79]	0.27 [0.01;5.93]	0.18 [0.01;2.80]	0.14 [0.01;1.45]	0.06 [0.01;0.71]	0.57 [0.02;20.68]	0.04 [0.00;0.59]	0.02 [0.00;0.55]	0.10 [0.01;1.49]	0.06 [0.01;0.70]	1.03 [0.04;29.33]	0.15 [0.01;2.17]	0.05 [0.00;1.47]	MINOC+CLOZ	-	-	-	-	-	-	-	-	-	-	-	-	-	-	-	-	-	-	-
0.17 [0.01;2.32]	0.66 [0.05;8.04]	0.50 [0.03;9.99]	0.34 [0.02;4.72]	0.26 [0.03;2.40]	0.11 [0.01;1.15]	1.07 [0.03;35.81]	0.07 [0.01;0.96]	0.04 [0.00;0.93]	0.19 [0.01;2.46]	0.12 [0.01;1.14]	1.93 [0.07;50.50]	0.28 [0.02;3.57]	0.09 [0.00;2.49]	1.87 [0.07;48.70]	MIRT+RISP	-	-	-	-	-	-	-	-	6.13 [0.68;54.99]	-	-	-	-	-	-	-	-	-
0.83 [0.24;2.89]	3.14 [0.98;10.10]	2.38 [0.34;16.81]	1.62 [0.39;6.72]	1.21 [0.80;1.83]	0.54 [0.27;1.09]	5.08 [0.33;77.78]	0.34 [0.09;1.37]	0.18 [0.02;1.84]	0.90 [0.24;3.31]	0.56 [0.33;0.96]	9.14 [0.82;101.45]	1.34 [0.38;4.71]	0.45 [0.04;5.01]	8.86 [0.80;97.66]	4.73 [0.49;45.32]	OLA	0.21 [0.02;2.59]	1.45 [0.41;5.05]	-	-	-	-	0.67 [0.10;4.26]	1.93 [0.77;4.83]	-	-	-	-	-	-	-	-	-
0.46 [0.08;2.82]	1.76 [0.32;9.55]	1.33 [0.13;13.93]	0.91 [0.14;5.93]	0.68 [0.19;2.46]	0.30 [0.07;1.23]	2.84 [0.14;56.53]	0.19 [0.03;1.20]	0.10 [0.01;1.39]	0.50 [0.08;3.01]	0.31 [0.08;1.19]	5.11 [0.34;76.10]	0.75 [0.13;4.32]	0.25 [0.02;3.75]	4.95 [0.33;73.32]	2.64 [0.21;32.93]	0.56 [0.15;2.06]	OLA+RISP	-	-	-	-	-	-	1.69 [0.41;7.01]	-	-	-	-	-	-	-	-	-
1.20 [0.20;7.02]	4.55 [0.82;25.14]	3.45 [0.34;35.05]	2.35 [0.35;15.58]	1.75 [0.47;6.52]	0.78 [0.19;3.28]	7.35 [0.37;147.82]	0.50 [0.08;3.21]	0.26 [0.02;3.65]	1.30 [0.21;7.92]	0.81 [0.21;3.15]	13.24 [0.88;199.19]	1.94 [0.33;11.41]	0.65 [0.04;9.82]	12.83 [0.86;191.90]	6.85 [0.52;90.54]	1.45 [0.41;5.05]	2.59 [0.43;15.75]	PALIP	-	-	-	-	-	-	-	-	-	-	-	-	-	-	-
12.84 [2.07;79.43]	48.78 [8.97;265.38]	36.95 [3.49;391.62]	25.16 [3.84;164.72]	18.76 [5.14;68.53]	8.41 [2.44;28.98]	78.79 [3.95;1570.93]	5.33 [0.88;32.37]	2.79 [0.41;18.89]	13.96 [2.46;79.18]	8.68 [2.45;30.78]	141.92 [9.52;2114.88]	20.80 [3.96;109.35]	6.95 [0.48;100.62]	137.57 [9.29;2037.40]	73.40 [5.64;955.67]	15.52 [4.13;58.33]	27.78 [4.59;168.22]	10.72 [1.74;66.20]	PBO	-	0.36 [0.05;2.43]	-	-	-	-	-	-	-	0.17 [0.04;0.67]	-	0.03 [0.00;0.21]	-	-
0.68 [0.09;5.14]	2.58 [0.38;17.46]	1.95 [0.16;24.26]	1.33 [0.17;10.63]	0.99 [0.21;4.77]	0.44 [0.09;2.17]	4.16 [0.18;94.44]	0.28 [0.04;2.08]	0.15 [0.01;2.25]	0.74 [0.11;5.06]	0.46 [0.10;2.09]	7.49 [0.44;128.84]	1.10 [0.23;5.14]	0.37 [0.02;6.04]	7.26 [0.42;124.16]	3.88 [0.26;58.59]	0.82 [0.17;4.02]	1.47 [0.20;10.92]	0.57 [0.07;4.28]	0.05 [0.01;0.37]	PERPH	-	-	-	-	-	-	-	-	-	-	-	-	-
4.61 [0.33;64.77]	17.51 [1.36;225.62]	13.26 [0.63;277.09]	9.03 [0.62;132.03]	6.73 [0.67;67.94]	3.02 [0.31;29.50]	28.28 [0.81;987.10]	1.91 [0.14;26.55]	1.00 [0.24;4.20]	5.01 [0.38;66.39]	3.12 [0.31;30.93]	50.95 [1.86;1396.47]	7.47 [0.59;94.06]	2.49 [0.09;66.79]	49.38 [1.81;1346.85]	26.35 [1.07;647.52]	5.57 [0.54;57.12]	9.97 [0.72;138.11]	3.85 [0.27;54.02]	0.36 [0.05;2.43]	6.80 [0.44;104.10]	PIPOTLA	-	-	-	-	-	-	-	-	-	-	-	-
0.28 [0.04;1.93]	1.06 [0.17;6.48]	0.81 [0.07;9.29]	0.55 [0.08;3.98]	0.41 [0.10;1.73]	0.18 [0.04;0.85]	1.72 [0.08;36.57]	0.12 [0.02;0.80]	0.06 [0.00;0.91]	0.30 [0.05;2.03]	0.19 [0.04;0.83]	3.10 [0.19;49.58]	0.45 [0.07;2.92]	0.15 [0.01;2.44]	3.00 [0.19;47.77]	1.60 [0.12;21.24]	0.34 [0.08;1.47]	0.61 [0.10;3.84]	0.23 [0.03;1.61]	0.02 [0.00;0.15]	0.41 [0.05;3.39]	0.06 [0.00;0.91]	PTX+RISP	-	3.83 [0.98;15.00]	-	-	-	-	-	-	-	-	-
1.04 [0.25;4.39]	3.94 [1.10;14.16]	2.99 [0.37;23.95]	2.03 [0.45;9.26]	1.52 [0.78;2.95]	0.68 [0.36;1.30]	6.37 [0.40;102.55]	0.43 [0.11;1.73]	0.23 [0.02;2.32]	1.13 [0.29;4.38]	0.70 [0.37;1.35]	11.48 [0.98;134.63]	1.68 [0.47;6.00]	0.56 [0.05;6.47]	11.12 [0.95;129.62]	5.93 [0.59;59.75]	1.25 [0.61;2.57]	2.25 [0.55;9.22]	0.87 [0.21;3.66]	0.08 [0.02;0.31]	1.53 [0.30;7.74]	0.23 [0.02;2.32]	3.71 [0.79;17.36]	QUE	1.84 [0.44;7.76]	-	-	-	-	-	-	-	-	-
1.07 [0.27;4.19]	4.07 [1.25;13.28]	3.08 [0.41;23.42]	2.10 [0.50;8.82]	1.57 [0.99;2.47]	0.70 [0.35;1.41]	6.58 [0.43;101.40]	0.44 [0.11;1.74]	0.23 [0.02;2.39]	1.16 [0.31;4.34]	0.72 [0.41;1.28]	11.84 [1.06;132.38]	1.74 [0.49;6.15]	0.58 [0.05;6.53]	11.48 [1.03;127.43]	6.12 [0.68;54.99]	1.30 [0.76;2.22]	2.32 [0.67;8.04]	0.89 [0.23;3.49]	0.08 [0.02;0.32]	1.58 [0.32;7.83]	0.23 [0.02;2.39]	3.83 [0.98;15.00]	1.03 [0.50;2.12]	RISP	-	1.60 [0.57;4.49]	-	-	-	-	-	-	-
0.64 [0.12;3.26]	2.42 [0.57;10.38]	1.84 [0.20;16.86]	1.25 [0.24;6.62]	0.93 [0.36;2.44]	0.42 [0.13;1.32]	3.92 [0.22;68.65]	0.26 [0.05;1.40]	0.14 [0.01;1.69]	0.69 [0.14;3.45]	0.43 [0.15;1.27]	7.05 [0.55;91.08]	1.03 [0.22;4.92]	0.35 [0.03;4.60]	6.84 [0.53;87.72]	3.65 [0.32;41.81]	0.77 [0.27;2.20]	1.38 [0.28;6.93]	0.53 [0.10;2.72]	0.05 [0.01;0.25]	0.94 [0.15;5.95]	0.14 [0.01;1.69]	2.28 [0.40;12.88]	0.61 [0.19;1.98]	0.60 [0.21;1.73]	RISP+CLOZ	-	-	-	-	-	-	-	1.67 [0.18;15.08]
1.72 [0.31;9.48]	6.53 [1.36;31.29]	4.94 [0.51;48.02]	3.37 [0.58;19.68]	2.51 [0.81;7.73]	1.13 [0.32;3.90]	10.54 [0.57;196.03]	0.71 [0.13;3.94]	0.37 [0.03;4.76]	1.87 [0.35;9.93]	1.16 [0.36;3.76]	18.99 [1.38;261.88]	2.78 [0.55;14.21]	0.93 [0.07;12.91]	18.41 [1.34;252.24]	9.82 [0.87;110.88]	2.08 [0.65;6.63]	3.72 [0.74;18.68]	1.43 [0.26;7.90]	0.13 [0.02;0.72]	2.53 [0.38;16.98]	0.37 [0.03;4.76]	6.13 [1.11;33.93]	1.65 [0.47;5.80]	1.60 [0.57;4.49]	2.69 [0.61;11.82]	SERTIND	-	-	-	-	-	-	-
0.31 [0.04;2.50]	1.17 [0.16;8.48]	0.89 [0.07;11.67]	0.60 [0.07;5.14]	0.45 [0.09;2.35]	0.20 [0.04;0.93]	1.89 [0.08;44.71]	0.13 [0.02;1.03]	0.07 [0.00;1.04]	0.33 [0.04;2.59]	0.21 [0.04;1.10]	3.40 [0.19;61.25]	0.50 [0.07;3.45]	0.17 [0.01;2.98]	3.30 [0.18;59.03]	1.76 [0.11;27.96]	0.37 [0.07;2.00]	0.67 [0.08;5.31]	0.26 [0.03;2.09]	0.02 [0.00;0.17]	0.45 [0.05;4.12]	0.07 [0.00;1.04]	1.10 [0.13;9.61]	0.30 [0.06;1.57]	0.29 [0.05;1.55]	0.48 [0.07;3.27]	0.18 [0.02;1.29]	SUL	-	-	-	-	-	-
0.96 [0.11;8.68]	3.64 [0.46;29.00]	2.76 [0.19;39.65]	1.88 [0.20;17.44]	1.40 [0.24;8.18]	0.63 [0.10;4.08]	5.88 [0.23;147.73]	0.40 [0.04;3.69]	0.21 [0.01;3.81]	1.04 [0.12;9.24]	0.65 [0.10;4.04]	10.59 [0.55;203.48]	1.55 [0.18;13.33]	0.52 [0.03;10.23]	10.27 [0.54;196.13]	5.48 [0.32;94.98]	1.16 [0.19;7.10]	2.07 [0.23;18.50]	0.80 [0.09;7.23]	0.07 [0.01;0.67]	1.41 [0.13;15.03]	0.21 [0.01;3.81]	3.42 [0.35;33.39]	0.92 [0.14;6.09]	0.89 [0.14;5.54]	1.50 [0.20;11.20]	0.56 [0.07;4.52]	3.11 [0.28;34.99]	SUL+CLOZ	-	-	-	-	-
1.80 [0.30;10.77]	6.83 [1.30;35.90]	5.17 [0.50;53.49]	3.52 [0.56;22.36]	2.63 [0.75;9.17]	1.18 [0.36;3.90]	11.03 [0.56;215.77]	0.75 [0.13;4.38]	0.39 [0.04;4.00]	1.95 [0.36;10.69]	1.22 [0.36;4.10]	19.88 [1.36;289.86]	2.91 [0.57;14.81]	0.97 [0.07;13.76]	19.27 [1.33;279.23]	10.28 [0.81;130.82]	2.17 [0.60;7.81]	3.89 [0.66;22.80]	1.50 [0.25;8.97]	0.14 [0.04;0.53]	2.65 [0.39;17.98]	0.39 [0.04;4.00]	6.42 [0.98;41.92]	1.73 [0.48;6.30]	1.68 [0.46;6.07]	2.82 [0.58;13.64]	1.05 [0.20;5.43]	5.84 [0.84;40.83]	1.88 [0.22;16.32]	THIO	-	0.75 [0.13;4.32]	-	-
0.10 [0.01;1.07]	0.37 [0.04;3.62]	0.28 [0.02;4.75]	0.19 [0.02;2.15]	0.14 [0.02;1.05]	0.06 [0.01;0.52]	0.60 [0.02;17.23]	0.04 [0.00;0.46]	0.02 [0.00;0.45]	0.11 [0.01;1.14]	0.07 [0.01;0.52]	1.08 [0.05;24.00]	0.16 [0.02;1.65]	0.05 [0.00;1.21]	1.05 [0.05;23.14]	0.56 [0.03;11.26]	0.12 [0.02;0.91]	0.21 [0.02;2.29]	0.08 [0.01;0.89]	0.01 [0.00;0.08]	0.14 [0.01;1.83]	0.02 [0.00;0.45]	0.35 [0.03;4.10]	0.09 [0.01;0.77]	0.09 [0.01;0.71]	0.15 [0.02;1.41]	0.06 [0.01;0.56]	0.32 [0.02;4.25]	0.10 [0.01;1.47]	0.05 [0.01;0.57]	TOPIR+CLOZ	-	-	-
0.78 [0.10;6.34]	2.96 [0.41;21.54]	2.24 [0.17;29.55]	1.53 [0.18;13.04]	1.14 [0.22;5.98]	0.51 [0.10;2.49]	4.78 [0.20;113.39]	0.32 [0.04;2.60]	0.17 [0.01;2.21]	0.85 [0.11;6.46]	0.53 [0.10;2.74]	8.61 [0.48;155.36]	1.26 [0.18;8.86]	0.42 [0.02;7.46]	8.34 [0.46;149.73]	4.45 [0.28;70.89]	0.94 [0.17;5.06]	1.68 [0.21;13.46]	0.65 [0.08;5.29]	0.06 [0.01;0.34]	1.15 [0.13;10.42]	0.17 [0.01;2.21]	2.78 [0.32;24.37]	0.75 [0.14;4.03]	0.73 [0.13;3.93]	1.22 [0.18;8.30]	0.45 [0.06;3.27]	2.53 [0.28;22.91]	0.81 [0.07;9.16]	0.43 [0.09;2.19]	7.96 [0.59;106.91]	TRIFLUO	-	-
0.80 [0.17;3.77]	3.06 [0.77;12.06]	2.32 [0.27;19.96]	1.58 [0.32;7.77]	1.18 [0.51;2.70]	0.53 [0.23;1.18]	4.94 [0.29;83.09]	0.33 [0.07;1.58]	0.17 [0.02;1.92]	0.87 [0.20;3.89]	0.54 [0.22;1.34]	8.89 [0.72;109.69]	1.30 [0.33;5.22]	0.44 [0.03;5.42]	8.62 [0.70;105.63]	4.60 [0.43;49.61]	0.97 [0.39;2.41]	1.74 [0.38;7.96]	0.67 [0.14;3.14]	0.06 [0.01;0.26]	1.19 [0.21;6.66]	0.17 [0.02;1.92]	2.87 [0.55;14.89]	0.77 [0.30;2.01]	0.75 [0.30;1.88]	1.26 [0.35;4.50]	0.47 [0.12;1.86]	2.61 [0.46;14.77]	0.84 [0.12;5.91]	0.45 [0.11;1.82]	8.23 [0.94;71.70]	1.03 [0.18;6.00]	ZIP	-
1.06 [0.07;16.49]	4.04 [0.29;56.59]	3.06 [0.13;69.68]	2.08 [0.13;32.99]	1.55 [0.14;17.19]	0.70 [0.06;8.35]	6.53 [0.18;241.97]	0.44 [0.03;6.99]	0.23 [0.01;6.48]	1.16 [0.08;17.63]	0.72 [0.06;8.35]	11.76 [0.40;343.80]	1.72 [0.12;25.60]	0.58 [0.02;17.23]	11.40 [0.39;331.62]	6.08 [0.23;162.60]	1.29 [0.11;14.73]	2.30 [0.15;35.27]	0.89 [0.06;13.75]	0.08 [0.01;1.27]	1.57 [0.09;27.72]	0.23 [0.01;6.48]	3.80 [0.23;62.56]	1.02 [0.08;12.40]	0.99 [0.09;11.46]	1.67 [0.18;15.08]	0.62 [0.04;8.79]	3.46 [0.19;63.93]	1.11 [0.06;21.89]	0.59 [0.04;8.88]	10.88 [0.48;247.73]	1.37 [0.07;25.33]	1.32 [0.10;16.82]	ZIP+CLOZ

Table S 11.5. League table: Response rates

AMI: amisulpride; ARI: aripiprazole; CLOZ: clozapine; CPZ: chlorpromazine; DESMOP: desmopressin; DONEP: donepezil FLUPH: fluphenazine ; FLUPHLA: fluphenazine decanoate; GinkBil: Ginkgo biloba; HAL: haloperidol; LAM: lamotrigine; LEV: levomepromazine; LI: lithium; MINOC: minocycline; MIRT: mirtazapine; OLA: olanzapine; OXYT: Oxytocin; PALIP: paliperidone; PBO: placebo; PERPH: perphenazine; PIPOTLA: pipotiazine long-acting; PTX: pentoxifylline; RISP: risperidone; SERTIND: sertindole; SUL: sulpiride; THIO: thioridazine; TOPIR: topiramate; TRIFLUO: trifluoperazine; ZIP: ziprasidone.

11.6. League table: Drop-outs due to any reason

[illegible]

Table S 11.6. League table: Dropouts due to any reason

AMI: amisulpride; ARI: aripiprazole; BEZOAET: benzoate sodium; CELECOX: celecoxib; CEREBR: cerebrolysin; CLOT: clotiapine; CLOZ: clozapine; CPZ: chlorpromazine; Dcyclos: D-cycloserine; DESMOP: desmopressin; DONEP: donepezil; DUL: duloxetine; FLUO: fluoxetine; FLUPH: fluphenazine; FLUPHLA: fluphenazine decanoate; FLUV: fluvoxamine; GinkBil: Ginkgo biloba; GLY: glycine; HAL: haloperidol; LAM: lamotrigine; LEV: levomepromazine; LI: lithium; MEM: memantine; MET: metformin; MINOC: minocycline; MIRT: mirtazapine; MODF: modafinil; Nacetyl: N-acetylcysteine; OLA: olanzapine; ONDAST: ondansetron; OXYT: oxytocin; PALIP: paliperidone; PALIPLA: paliperidone long-acting; PALMIT: palmitoylethanolamide; PBO: placebo; PHENYLPROP: phenylpropanolamine; PIMOZ: pimozide; PIPOTLA: pipotiazine long-acting; PTX: pentoxifylline; QUE: quetiapine; RESVER: resveratrol; RISP: risperidone; SER: sertraline; SERTIND: sertindole; SUL: sulpiride; THIO: thioridazine; TOPIR: topiramate; TRIFLUO: trifluoperazine; VALPRO: valproate acid VitD3: vitamin D3; VORT: vortioxetine; ZIP: ziprasidone; ZOT: zotepine.

11.7. League table: Drop-outs due to any adverse effects

AMI	-	-	-	-	-	-	-	-	-	-	-	-	-	-	-	-	-	2.85 [0.79;10.30]	-	-	-	-	-	-	-	-	-	-	-	-	-	-	-	-	-	-	-	-	-	-
2.14 [0.30;15.34]	AMI+CLOZ	-	0.78 [0.19;3.14]	-	-	-	-	-	-	-	-	-	-	-	-	-	-	-	-	-	-	-	-	-	-	-	-	-	-	-	-	-	-	-	-	-	-	-	-	-
1.04 [0.14;7.53]	0.48 [0.07;3.53]	ARI+CLOZ	1.60 [0.39;6.59]	-	-	-	-	-	-	-	-	-	-	-	-	-	-	-	-	-	-	-	-	-	-	-	-	-	-	-	-	-	-	-	-	-	-	-	-	-
1.66 [0.41;6.69]	0.78 [0.19;3.14]	1.60 [0.39;6.59]	CLOZ	0.35 [0.14;0.85]	0.12 [0.01;2.53]	-	-	0.48 [0.04;5.46]	-	0.33 [0.01;8.93]	1.10 [0.65;1.87]	1.62 [0.23;11.26]	-	-	1.00 [0.06;16.89]	5.27 [0.24;117.26]	0.10 [0.01;1.89]	1.75 [0.98;3.13]	0.09 [0.00;2.51]	-	-	-	-	-	0.49 [0.10;2.48]	-	0.57 [0.15;2.26]	1.52 [0.83;2.78]	0.31 [0.03;3.05]	-	-	-	2.09 [0.18;24.61]	-	-	0.03 [0.00;0.49]	-	0.91 [0.41;2.00]	0.18 [0.01;4.01]	0.47 [0.02;12.01]
0.57 [0.13;2.55]	0.27 [0.06;1.22]	0.55 [0.12;2.56]	0.34 [0.19;0.63]	CPZ	-	-	-	-	-	-	-	-	1.00 [0.06;17.25]	-	-	-	-	6.83 [0.79;59.48]	-	-	-	-	-	-	-	-	3.39 [1.39;8.27]	0.39 [0.01;10.37]	-	-	-	-	-	3.08 [0.12;78.02]	-	-	-	1.40 [0.43;4.51]	-	-
0.20 [0.01;5.70]	0.09 [0.00;2.67]	0.20 [0.01;5.54]	0.12 [0.01;2.53]	0.36 [0.02;7.83]	DUL+CLOZ	-	-	-	-	-	-	-	-	-	-	-	-	-	-	-	-	-	-	-	-	-	-	-	-	-	-	-	-	-	-	-	-	-	-	-
2.09 [0.09;50.54]	0.98 [0.04;23.77]	2.02 [0.08;49.40]	1.26 [0.07;22.17]	3.67 [0.20;68.12]	10.32 [0.16;669.99]	FLUPH	-	-	-	-	1.00 [0.06;16.93]	-	-	-	-	-	-	-	-	-	-	-	-	-	-	-	-	-	-	-	-	-	-	-	-	-	-	-	-	-
0.13 [0.00;6.54]	0.06 [0.00;3.07]	0.12 [0.00;6.38]	0.08 [0.00;3.07]	0.22 [0.01;9.32]	0.62 [0.01;74.15]	0.06 [0.00;6.19]	FLUPHLA	-	-	-	-	-	-	-	-	-	-	-	-	-	-	-	-	4.33 [0.83;22.75]	-	1.56 [0.53;4.53]	-	-	-	-	-	-	-	-	-	-	-	-	-	-
0.79 [0.05;13.13]	0.37 [0.02;6.15]	0.76 [0.05;12.80]	0.48 [0.04;5.46]	1.39 [0.11;17.16]	3.90 [0.08;191.11]	0.38 [0.01;16.35]	6.33 [0.07;535.65]	FLUV+CLOZ	-	-	-	-	-	-	-	-	-	-	-	-	-	-	-	-	-	-	-	-	-	-	-	-	-	-	-	-	-	-	-	-
16.38 [0.59;456.03]	7.66 [0.27;214.38]	15.80 [0.56;445.45]	9.85 [0.48;202.97]	28.74 [1.33;622.12]	80.80 [1.12;5851.29]	7.83 [0.13;479.38]	130.97 [1.15;14952.19]	20.69 [0.42;1008.14]	GinkBil+HAL	-	0.13 [0.01;2.53]	-	-	-	-	-	-	-	-	-	-	-	-	-	-	-	-	-	-	-	-	-	-	-	-	-	-	-	-	-
0.55 [0.02;19.70]	0.26 [0.01;9.23]	0.53 [0.01;19.16]	0.33 [0.01;8.93]	0.97 [0.03;27.55]	2.73 [0.03;239.30]	0.26 [0.00;20.81]	4.43 [0.03;629.23]	0.70 [0.01;42.00]	0.03 [0.00;2.95]	GLY+CLOZ	-	-	-	-	-	-	-	-	-	-	-	-	-	-	-	-	-	-	-	-	-	-	-	-	-	-	-	-	-	-
2.09 [0.48;9.03]	0.98 [0.22;4.27]	2.02 [0.45;8.96]	1.26 [0.78;2.03]	3.67 [1.77;7.60]	10.32 [0.48;221.89]	1.00 [0.06;16.93]	16.72 [0.42;661.06]	2.64 [0.22;31.72]	0.13 [0.01;2.53]	3.77 [0.14;104.69]	HAL	-	-	0.37 [0.01;9.98]	-	-	-	2.79 [0.10;74.63]	-	-	1.58 [0.36;6.93]	-	-	0.24 [0.01;6.34]	-	-	0.39 [0.13;1.14]	0.57 [0.06;5.20]	-	-	-	-	-	-	0.17 [0.01;3.73]	-	-	-	-	-
2.69 [0.25;29.29]	1.26 [0.12;13.72]	2.59 [0.23;28.60]	1.62 [0.23;11.26]	4.71 [0.62;36.07]	13.25 [0.36;484.68]	1.28 [0.04;41.03]	21.47 [0.33;1411.44]	3.39 [0.15;76.66]	0.16 [0.00;5.97]	4.85 [0.11;220.76]	1.28 [0.17;9.48]	LAM+CLOZ	-	-	-	-	-	-	-	-	-	-	-	-	-	-	-	-	-	-	-	-	-	-	-	-	-	-	-	-
0.57 [0.02;14.24]	0.27 [0.01;6.73]	0.55 [0.02;14.00]	0.34 [0.02;6.31]	1.00 [0.06;17.25]	2.81 [0.04;188.14]	0.27 [0.00;16.11]	4.56 [0.04;504.88]	0.72 [0.02;32.15]	0.03 [0.00;2.30]	1.03 [0.01;83.16]	0.27 [0.01;5.15]	0.21 [0.01;7.03]	LEV	-	-	-	-	-	-	-	-	-	-	-	-	-	-	-	-	-	-	-	-	-	-	-	-	-	-	-
0.76 [0.02;28.43]	0.36 [0.01;13.36]	0.74 [0.02;27.75]	0.46 [0.02;12.99]	1.34 [0.05;39.64]	3.77 [0.04;343.16]	0.37 [0.00;28.36]	6.11 [0.04;858.61]	0.96 [0.02;60.44]	0.05 [0.00;4.02]	1.38 [0.01;149.81]	0.37 [0.01;9.98]	0.28 [0.01;13.57]	1.34 [0.02;111.92]	LI+HAL	-	-	-	-	-	-	-	-	-	-	-	-	-	-	-	-	-	-	-	-	-	-	-	-	-	-
1.66 [0.07;38.83]	0.78 [0.03;18.19]	1.60 [0.07;37.81]	1.00 [0.06;16.89]	2.92 [0.16;52.57]	8.20 [0.13;517.35]	0.79 [0.01;44.61]	13.29 [0.13;1407.60]	2.10 [0.05;87.84]	0.10 [0.00;6.38]	3.00 [0.04;229.27]	0.79 [0.05;13.97]	0.62 [0.02;19.10]	2.92 [0.05;168.84]	2.18 [0.03;173.37]	MEM+CLOZ	-	-	-	-	-	-	-	-	-	-	-	-	-	-	-	-	-	-	-	-	-	-	-	-	-
8.76 [0.29;262.64]	4.10 [0.14;122.99]	8.45 [0.28;255.52]	5.27 [0.24;117.26]	15.37 [0.65;362.90]	43.22 [0.56;3305.82]	4.19 [0.06;286.56]	70.05 [0.56;8811.08]	11.07 [0.21;572.72]	0.53 [0.01;40.75]	15.81 [0.17;1453.08]	4.19 [0.18;96.66]	3.26 [0.08;126.78]	15.37 [0.22;1083.07]	11.47 [0.12;1096.98]	5.27 [0.08;350.38]	MODF+CLOZ	-	-	-	-	-	-	-	-	-	-	-	-	-	-	-	-	-	-	-	-	-	-	-	-
0.16 [0.01;4.28]	0.08 [0.00;2.01]	0.16 [0.01;4.17]	0.10 [0.01;1.89]	0.29 [0.01;5.85]	0.81 [0.01;55.54]	0.08 [0.00;4.80]	1.31 [0.01;149.67]	0.21 [0.00;9.52]	0.01 [0.00;0.68]	0.30 [0.00;24.52]	0.08 [0.00;1.56]	0.06 [0.00;2.09]	0.29 [0.00;18.16]	0.21 [0.00;18.53]	0.10 [0.00;5.87]	0.02 [0.00;1.35]	Nacetyl+CLOZ	-	-	-	-	-	-	-	-	-	-	-	-	-	-	-	-	-	-	-	-	-	-	-
2.85 [0.79;10.30]	1.33 [0.30;5.95]	2.75 [0.61;12.48]	1.72 [1.00;2.94]	5.00 [2.31;10.84]	14.07 [0.65;305.74]	1.36 [0.07;25.16]	22.81 [0.54;963.50]	3.60 [0.30;43.81]	0.17 [0.01;3.75]	5.15 [0.18;144.14]	1.36 [0.68;2.75]	1.06 [0.14;7.97]	5.00 [0.26;95.69]	3.74 [0.13;109.92]	1.72 [0.10;30.50]	0.33 [0.01;7.59]	17.45 [0.87;351.37]	OLA	-	1.09 [0.13;9.12]	-	-	4.76 [0.51;44.44]	-	-	-	0.54 [0.08;3.68]	2.68 [0.10;70.31]	-	3.43 [0.32;36.83]	-	-	-	-	-	-	-	-	-	-
0.16 [0.00;5.53]	0.07 [0.00;2.59]	0.15 [0.00;5.38]	0.09 [0.00;2.51]	0.27 [0.01;7.73]	0.77 [0.01;67.12]	0.07 [0.00;5.84]	1.24 [0.01;176.47]	0.20 [0.00;11.78]	0.01 [0.00;0.83]	0.28 [0.00;29.35]	0.07 [0.00;2.06]	0.06 [0.00;2.64]	0.27 [0.00;22.05]	0.20 [0.00;22.13]	0.09 [0.00;7.15]	0.02 [0.00;1.63]	0.95 [0.01;79.02]	0.05 [0.00;1.53]	OLA+CLOZ	-	-	-	-	-	-	-	-	-	-	-	-	-	-	-	-	-	-	-	-	-
2.78 [0.32;24.03]	1.30 [0.14;12.29]	2.69 [0.28;25.64]	1.67 [0.29;9.72]	4.88 [0.77;30.97]	13.73 [0.41;456.76]	1.33 [0.05;38.35]	22.26 [0.37;1343.64]	3.52 [0.17;71.15]	0.17 [0.01;5.60]	5.02 [0.12;209.21]	1.33 [0.22;8.17]	1.04 [0.08;14.24]	4.88 [0.16;145.52]	3.65 [0.08;158.65]	1.67 [0.06;46.75]	0.32 [0.01;11.24]	17.03 [0.55;529.90]	0.98 [0.17;5.51]	17.94 [0.43;748.82]	OLA+RISP	-	-	-	-	-	-	-	1.00 [0.06;17.90]	-	-	-	-	-	-	-	-	-	-	-	-
3.31 [0.41;26.46]	1.55 [0.19;12.47]	3.19 [0.39;26.03]	1.99 [0.42;9.40]	5.80 [1.12;30.13]	16.31 [0.54;491.44]	1.58 [0.06;38.46]	26.43 [0.50;1390.66]	4.18 [0.23;75.27]	0.20 [0.01;5.66]	5.97 [0.16;226.48]	1.58 [0.36;6.93]	1.23 [0.10;14.80]	5.80 [0.22;155.70]	4.33 [0.12;162.18]	1.99 [0.08;50.03]	0.38 [0.01;12.12]	20.22 [0.72;569.02]	1.16 [0.23;5.95]	21.30 [0.56;810.66]	1.19 [0.11;12.33]	ONDAST+HAL	-	-	-	-	-	-	-	-	-	-	-	-	-	-	-	-	-	-	-
0.41 [0.01;12.85]	0.19 [0.01;6.04]	0.40 [0.01;12.55]	0.25 [0.01;5.78]	0.72 [0.03;17.78]	2.04 [0.03;160.94]	0.20 [0.00;13.91]	3.30 [0.03;426.02]	0.52 [0.01;27.97]	0.03 [0.00;1.98]	0.75 [0.01;70.65]	0.20 [0.01;4.74]	0.15 [0.00;6.21]	0.72 [0.01;52.56]	0.54 [0.01;53.12]	0.25 [0.00;17.08]	0.05 [0.00;3.91]	2.53 [0.03;189.23]	0.14 [0.01;3.51]	2.66 [0.03;252.77]	0.15 [0.00;5.28]	0.12 [0.00;4.16]	ONDAST+RISP	-	-	-	-	-	5.49 [0.25;121.18]	-	-	-	-	-	-	-	-	-	-	-	-
13.57 [1.03;178.52]	6.35 [0.43;93.40]	13.09 [0.88;194.41]	8.16 [0.82;81.30]	23.80 [2.24;253.24]	66.93 [1.49;3004.05]	6.49 [0.16;255.34]	108.49 [1.39;8487.18]	17.14 [0.60;489.34]	0.83 [0.02;36.88]	24.49 [0.44;1353.13]	6.49 [0.62;67.50]	5.05 [0.25;102.42]	23.80 [0.59;964.18]	17.77 [0.31;1023.37]	8.16 [0.21;311.95]	1.55 [0.03;73.59]	83.00 [1.97;3503.78]	4.76 [0.51;44.44]	87.45 [1.58;4842.40]	4.87 [0.29;82.33]	4.10 [0.26;65.48]	32.84 [0.67;1612.64]	PALIP	-	-	-	-	-	-	-	-	-	-	-	-	-	-	-	-	-
0.54 [0.01;19.70]	0.25 [0.01;9.26]	0.52 [0.01;19.23]	0.33 [0.01;8.98]	0.95 [0.03;27.42]	2.67 [0.03;238.92]	0.26 [0.00;19.73]	4.33 [0.83;22.75]	0.68 [0.01;42.01]	0.03 [0.00;2.80]	0.98 [0.01;104.38]	0.26 [0.01;6.90]	0.20 [0.00;9.42]	0.95 [0.01;77.89]	0.71 [0.01;74.97]	0.33 [0.00;25.45]	0.06 [0.00;5.80]	3.32 [0.04;281.33]	0.19 [0.01;5.45]	3.49 [0.03;373.43]	0.19 [0.00;8.28]	0.16 [0.00;6.00]	1.31 [0.01;126.40]	0.04 [0.00;2.25]	PBO	-	0.36 [0.07;1.93]	-	-	-	-	-	-	-	-	0.58 [0.05;7.26]	-	0.45 [0.03;7.77]	-	-	-
0.81 [0.09;6.91]	0.38 [0.04;3.24]	0.78 [0.09;6.75]	0.49 [0.10;2.48]	1.42 [0.25;8.09]	3.99 [0.13;124.71]	0.39 [0.01;10.49]	6.47 [0.11;371.62]	1.02 [0.05;19.22]	0.05 [0.00;1.54]	1.46 [0.04;57.35]	0.39 [0.07;2.11]	0.30 [0.02;3.80]	1.42 [0.05;39.97]	1.06 [0.03;43.69]	0.49 [0.02;12.72]	0.09 [0.00;3.07]	4.95 [0.17;144.51]	0.28 [0.05;1.58]	5.22 [0.13;205.26]	0.29 [0.03;3.20]	0.24 [0.03;2.33]	1.96 [0.06;67.81]	0.06 [0.00;1.00]	1.49 [0.04;60.13]	PIMOZ+CLOZ	-	-	-	-	-	-	-	-	-	-	-	-	-	-	-
0.19 [0.00;10.28]	0.09 [0.00;4.83]	0.19 [0.00;10.02]	0.12 [0.00;4.82]	0.34 [0.01;14.64]	0.96 [0.01;116.26]	0.09 [0.00;9.70]	1.56 [0.53;4.53]	0.25 [0.00;20.98]	0.01 [0.00;1.37]	0.35 [0.00;50.24]	0.09 [0.00;3.72]	0.07 [0.00;4.81]	0.34 [0.00;38.12]	0.25 [0.00;36.11]	0.12 [0.00;12.49]	0.02 [0.00;2.81]	1.19 [0.01;137.35]	0.07 [0.00;2.91]	1.25 [0.01;179.73]	0.07 [0.00;4.26]	0.06 [0.00;3.13]	0.47 [0.00;61.22]	0.01 [0.00;1.13]	0.36 [0.07;1.93]	0.24 [0.00;13.93]	PIPOTLA	-	-	-	-	-	-	-	-	-	-	-	-	-	-
1.31 [0.28;6.05]	0.61 [0.13;2.90]	1.26 [0.26;6.08]	0.79 [0.40;1.56]	2.30 [1.15;4.60]	6.47 [0.29;144.60]	0.63 [0.03;11.62]	10.48 [0.25;444.44]	1.66 [0.13;20.86]	0.08 [0.00;1.73]	2.37 [0.08;68.02]	0.63 [0.30;1.29]	0.49 [0.06;3.83]	2.30 [0.12;43.11]	1.72 [0.06;50.72]	0.79 [0.04;14.45]	0.15 [0.01;3.59]	8.02 [0.39;166.30]	0.46 [0.20;1.05]	8.45 [0.29;243.53]	0.47 [0.07;3.06]	0.40 [0.08;2.05]	3.17 [0.13;79.02]	0.10 [0.01;1.05]	2.42 [0.08;69.66]	1.62 [0.28;9.49]	6.74 [0.16;288.63]	QUE	9.24 [0.44;195.69]	-	-	-	-	-	-	-	-	-	-	-	-
2.27 [0.51;10.12]	1.06 [0.24;4.79]	2.19 [0.48;10.04]	1.36 [0.77;2.41]	3.98 [1.76;8.98]	11.18 [0.51;244.35]	1.08 [0.06;20.08]	18.13 [0.43;768.28]	2.86 [0.23;35.06]	0.14 [0.01;2.99]	4.09 [0.15;115.15]	1.08 [0.53;2.23]	0.84 [0.11;6.39]	3.98 [0.21;76.91]	2.97 [0.10;87.68]	1.36 [0.08;24.38]	0.26 [0.01;6.06]	13.87 [0.68;280.85]	0.79 [0.37;1.71]	14.61 [0.52;412.24]	0.81 [0.14;4.84]	0.69 [0.13;3.55]	5.49 [0.25;121.18]	0.17 [0.02;1.78]	4.18 [0.15;120.41]	2.80 [0.50;15.74]	11.65 [0.27;498.94]	1.73 [0.72;4.13]	RISP	-	-	-	0.59 [0.21;1.63]	-	-	-	-	-	-	-	-
0.46 [0.04;5.28]	0.21 [0.02;2.47]	0.44 [0.04;5.15]	0.27 [0.04;2.06]	0.80 [0.10;6.56]	2.25 [0.06;85.62]	0.22 [0.01;7.26]	3.65 [0.05;247.98]	0.58 [0.02;13.62]	0.03 [0.00;1.05]	0.82 [0.02;38.91]	0.22 [0.03;1.73]	0.17 [0.01;2.79]	0.80 [0.02;27.60]	0.60 [0.01;29.57]	0.27 [0.01;8.82]	0.05 [0.00;2.10]	2.79 [0.08;99.58]	0.16 [0.02;1.29]	2.94 [0.06;139.26]	0.16 [0.01;2.37]	0.14 [0.01;1.76]	1.10 [0.03;46.30]	0.03 [0.00;0.71]	0.84 [0.02;40.75]	0.56 [0.04;7.52]	2.34 [0.03;160.86]	0.35 [0.04;2.92]	0.20 [0.02;1.63]	RISP+CLOZ	-	-	-	-	-	-	-	-	-	1.00 [0.06;18.08]	-
9.78 [0.66;145.36]	4.58 [0.28;75.68]	9.44 [0.57;157.48]	5.88 [0.52;67.13]	17.16 [1.41;208.36]	48.24 [0.99;2354.18]	4.68 [0.11;200.69]	78.20 [0.93;6581.87]	12.35 [0.39;387.72]	0.60 [0.01;28.91]	17.65 [0.29;1055.91]	4.68 [0.39;55.60]	3.64 [0.16;82.00]	17.16 [0.39;757.32]	12.81 [0.21;797.95]	5.88 [0.14;245.35]	1.12 [0.02;57.60]	59.82 [1.30;2749.49]	3.43 [0.32;36.83]	63.03 [1.05;3778.60]	3.51 [0.19;66.35]	2.96 [0.17;52.89]	23.67 [0.44;1261.40]	0.72 [0.03;18.78]	18.05 [0.30;1100.91]	12.08 [0.65;226.20]	50.27 [0.59;4267.70]	7.46 [0.60;92.27]	4.31 [0.36;52.32]	21.44 [0.91;505.08]	SER+OLA	-	-	-	-	-	-	-	-	-	-
5.34 [0.59;48.64]	2.50 [0.27;22.90]	5.15 [0.56;47.75]	3.21 [0.57;17.96]	9.37 [1.61;54.50]	26.34 [0.81;859.79]	2.55 [0.09;72.22]	42.69 [0.72;2538.39]	6.74 [0.34;133.51]	0.33 [0.01;10.55]	9.64 [0.24;394.27]	2.55 [0.43;15.14]	1.99 [0.15;26.64]	9.37 [0.33;266.52]	6.99 [0.16;299.33]	3.21 [0.12;87.92]	0.61 [0.02;21.17]	32.66 [1.07;997.11]	1.87 [0.31;11.30]	34.41 [0.84;1411.19]	1.92 [0.16;22.39]	1.62 [0.16;16.34]	12.92 [0.36;466.19]	0.39 [0.02;6.93]	9.85 [0.24;412.10]	6.60 [0.62;70.59]	27.44 [0.46;1647.10]	4.07 [0.66;25.18]	2.36 [0.39;14.40]	11.70 [0.83;165.53]	0.55 [0.03;10.73]	SER+ZIP	-	-	-	-	-	-	0.23 [0.05;1.12]	-	-
1.33 [0.22;8.14]	0.62 [0.10;3.84]	1.28 [0.20;8.03]	0.80 [0.25;2.58]	2.33 [0.63;8.63]	6.54 [0.25;168.85]	0.63 [0.03;14.00]	10.61 [0.22;516.13]	1.68 [0.11;25.11]	0.08 [0.00;2.07]	2.39 [0.07;78.62]	0.63 [0.18;2.22]	0.49 [0.05;4.78]	2.33 [0.10;53.49]	1.74 [0.05;59.74]	0.80 [0.04;17.03]	0.15 [0.01;4.18]	8.12 [0.34;194.85]	0.47 [0.13;1.68]	8.55 [0.26;281.44]	0.48 [0.06;3.72]	0.40 [0.06;2.79]	3.21 [0.12;83.70]	0.10 [0.01;1.29]	2.45 [0.07;82.13]	1.64 [0.22;12.21]	6.82 [0.14;335.07]	1.01 [0.26;3.89]	0.59 [0.21;1.63]	2.91 [0.28;29.92]	0.14 [0.01;2.02]	0.25 [0.03;1.99]	SERTIND	-	-	-	-	-	-	-	-
3.47 [0.20;58.99]	1.62 [0.10;27.63]	3.35 [0.19;57.49]	2.09 [0.18;24.61]	6.09 [0.48;77.32]	17.11 [0.34;852.67]	1.66 [0.04;72.99]	27.74 [0.32;2384.69]	4.38 [0.14;140.80]	0.21 [0.00;10.51]	6.26 [0.10;382.05]	1.66 [0.13;20.48]	1.29 [0.06;29.85]	6.09 [0.13;276.80]	4.54 [0.07;289.55]	2.09 [0.05;88.94]	0.40 [0.01;20.86]	21.22 [0.45;996.17]	1.22 [0.10;15.21]	22.36 [0.37;1367.17]	1.25 [0.06;25.80]	1.05 [0.06;19.38]	8.40 [0.15;458.09]	0.26 [0.01;7.45]	6.40 [0.10;399.53]	4.29 [0.22;82.48]	17.83 [0.21;1546.17]	2.65 [0.20;34.27]	1.53 [0.12;19.26]	7.61 [0.31;183.81]	0.35 [0.01;11.36]	0.65 [0.03;13.17]	2.61 [0.17;40.19]	SERTIND+CLOZ	-	-	-	-	-	-	-
1.76 [0.05;61.89]	0.82 [0.02;29.25]	1.69 [0.05;60.74]	1.06 [0.04;28.32]	3.08 [0.12;78.02]	8.66 [0.10;758.53]	0.84 [0.01;65.46]	14.04 [0.10;1980.66]	2.22 [0.04;133.13]	0.11 [0.00;9.28]	3.17 [0.03;331.64]	0.84 [0.03;23.06]	0.65 [0.01;29.80]	3.08 [0.04;228.74]	2.30 [0.02;248.26]	1.06 [0.01;80.75]	0.20 [0.00;18.43]	10.74 [0.13;892.97]	0.62 [0.02;17.08]	11.31 [0.11;1186.49]	0.63 [0.02;26.09]	0.53 [0.01;19.99]	4.25 [0.04;401.51]	0.13 [0.00;7.10]	3.24 [0.03;343.37]	2.17 [0.06;85.19]	9.02 [0.06;1283.12]	1.34 [0.05;36.52]	0.77 [0.03;21.70]	3.85 [0.08;182.05]	0.18 [0.00;10.66]	0.33 [0.01;13.04]	1.32 [0.04;43.27]	0.51 [0.01;30.90]	SUL	-	-	-	-	-	-
0.33 [0.01;10.19]	0.15 [0.00;4.79]	0.31 [0.01;9.95]	0.20 [0.01;4.59]	0.57 [0.02;14.03]	1.61 [0.02;127.55]	0.16 [0.00;10.49]	2.61 [0.13;53.34]	0.41 [0.01;22.17]	0.02 [0.00;1.49]	0.59 [0.01;55.99]	0.16 [0.01;3.52]	0.12 [0.00;4.92]	0.57 [0.01;41.48]	0.43 [0.00;40.20]	0.20 [0.00;13.53]	0.04 [0.00;3.10]	2.00 [0.03;149.98]	0.11 [0.00;2.79]	2.10 [0.02;200.31]	0.12 [0.00;4.31]	0.10 [0.00;3.10]	0.79 [0.01;67.62]	0.02 [0.00;1.19]	0.60 [0.05;7.49]	0.40 [0.01;14.00]	1.68 [0.08;34.73]	0.25 [0.01;6.09]	0.14 [0.01;3.52]	0.72 [0.02;30.11]	0.03 [0.00;1.78]	0.06 [0.00;2.21]	0.25 [0.01;7.06]	0.09 [0.00;5.15]	0.19 [0.00;17.55]	THIO	-	-	-	-	-
0.05 [0.00;1.13]	0.02 [0.00;0.53]	0.05 [0.00;1.10]	0.03 [0.00;0.49]	0.08 [0.00;1.54]	0.23 [0.00;14.97]	0.02 [0.00;1.29]	0.37 [0.00;40.61]	0.06 [0.00;2.55]	0.00 [0.00;0.18]	0.08 [0.00;6.63]	0.02 [0.00;0.41]	0.02 [0.00;0.56]	0.08 [0.00;4.89]	0.06 [0.00;5.01]	0.03 [0.00;1.58]	0.01 [0.00;0.36]	0.29 [0.00;17.56]	0.02 [0.00;0.30]	0.30 [0.00;23.71]	0.02 [0.00;0.49]	0.01 [0.00;0.37]	0.11 [0.00;8.00]	0.00 [0.00;0.14]	0.09 [0.00;6.92]	0.06 [0.00;1.56]	0.24 [0.00;26.32]	0.04 [0.00;0.68]	0.02 [0.00;0.38]	0.10 [0.00;3.41]	0.00 [0.00;0.21]	0.01 [0.00;0.25]	0.04 [0.00;0.78]	0.01 [0.00;0.59]	0.03 [0.00;2.09]	0.14 [0.00;10.16]	TOPIR+CLOZ	-	-	-	-
0.25 [0.00;24.00]	0.12 [0.00;11.27]	0.24 [0.00;23.36]	0.15 [0.00;11.66]	0.43 [0.01;35.19]	1.22 [0.01;246.92]	0.12 [0.00;20.92]	1.97 [0.07;52.72]	0.31 [0.00;46.20]	0.02 [0.00;2.92]	0.44 [0.00;105.04]	0.12 [0.00;9.02]	0.09 [0.00;10.90]	0.43 [0.00;81.61]	0.32 [0.00;75.56]	0.15 [0.00;26.87]	0.03 [0.00;5.95]	1.51 [0.01;293.11]	0.09 [0.00;7.00]	1.59 [0.01;375.71]	0.09 [0.00;9.76]	0.07 [0.00;7.29]	0.60 [0.00;129.14]	0.02 [0.00;2.51]	0.45 [0.03;7.77]	0.30 [0.00;32.13]	1.27 [0.05;34.29]	0.19 [0.00;15.29]	0.11 [0.00;8.83]	0.54 [0.00;66.09]	0.03 [0.00;3.72]	0.05 [0.00;5.02]	0.19 [0.00;16.98]	0.07 [0.00;10.69]	0.14 [0.00;32.96]	0.75 [0.02;33.62]	5.26 [0.03;975.93]	TRIFLUO	-	-	-
1.23 [0.26;5.78]	0.58 [0.12;2.73]	1.19 [0.25;5.72]	0.74 [0.37;1.47]	2.16 [0.99;4.71]	6.08 [0.27;135.99]	0.59 [0.03;11.21]	9.86 [0.23;426.63]	1.56 [0.12;19.62]	0.08 [0.00;1.67]	2.23 [0.08;63.97]	0.59 [0.26;1.34]	0.46 [0.06;3.60]	2.16 [0.11;41.42]	1.61 [0.05;48.80]	0.74 [0.04;13.59]	0.14 [0.01;3.37]	7.54 [0.36;156.40]	0.43 [0.18;1.02]	7.95 [0.28;229.03]	0.44 [0.07;2.91]	0.37 [0.07;2.02]	2.98 [0.12;74.61]	0.09 [0.01;1.00]	2.28 [0.08;67.02]	1.52 [0.26;8.92]	6.34 [0.14;277.04]	0.94 [0.38;2.33]	0.54 [0.22;1.32]	2.70 [0.32;22.67]	0.13 [0.01;1.57]	0.23 [0.05;1.12]	0.93 [0.24;3.60]	0.36 [0.03;4.60]	0.70 [0.03;19.51]	3.78 [0.15;94.71]	26.35 [1.38;502.17]	5.01 [0.06;414.21]	ZIP	-	-
0.38 [0.02;5.88]	0.18 [0.01;2.76]	0.36 [0.02;5.73]	0.23 [0.02;2.42]	0.66 [0.06;7.64]	1.85 [0.04;87.00]	0.18 [0.00;7.43]	3.00 [0.04;245.07]	0.47 [0.02;14.26]	0.02 [0.00;1.07]	0.68 [0.01;39.09]	0.18 [0.02;2.02]	0.14 [0.01;3.00]	0.66 [0.02;28.20]	0.49 [0.01;29.65]	0.23 [0.01;9.05]	0.04 [0.00;2.13]	2.30 [0.05;101.54]	0.13 [0.01;1.50]	2.42 [0.04;139.90]	0.13 [0.01;2.59]	0.11 [0.01;1.94]	0.91 [0.02;46.80]	0.03 [0.00;0.75]	0.69 [0.01;40.89]	0.46 [0.03;8.25]	1.93 [0.02;158.92]	0.29 [0.02;3.39]	0.17 [0.01;1.90]	0.82 [0.08;8.33]	0.04 [0.00;1.15]	0.07 [0.00;1.32]	0.28 [0.02;4.00]	0.11 [0.00;3.32]	0.21 [0.00;12.35]	1.15 [0.02;59.46]	8.02 [0.19;331.70]	1.52 [0.01;219.17]	0.30 [0.03;3.60]	ZIP+CLOZ	-
0.78 [0.02;26.59]	0.37 [0.01;12.45]	0.75 [0.02;25.86]	0.47 [0.02;12.01]	1.37 [0.05;37.08]	3.85 [0.05;325.78]	0.37 [0.00;28.31]	6.24 [0.05;859.48]	0.99 [0.02;56.99]	0.05 [0.00;4.02]	1.41 [0.01;142.62]	0.37 [0.01;9.89]	0.29 [0.01;12.72]	1.37 [0.02;106.94]	1.02 [0.01;107.58]	0.47 [0.01;34.64]	0.09 [0.00;7.92]	4.77 [0.06;383.36]	0.27 [0.01;7.32]	5.03 [0.05;510.26]	0.28 [0.01;11.21]	0.24 [0.01;8.60]	1.89 [0.02;173.17]	0.06 [0.00;3.06]	1.44 [0.01;148.78]	0.96 [0.03;36.31]	4.01 [0.03;556.81]	0.60 [0.02;16.36]	0.34 [0.01;9.26]	1.71 [0.04;77.75]	0.08 [0.00;4.60]	0.15 [0.00;5.74]	0.59 [0.02;18.49]	0.22 [0.00;13.23]	0.44 [0.00;45.04]	2.39 [0.03;219.93]	16.68 [0.22;1263.93]	3.17 [0.01;728.10]	0.63 [0.02;17.39]	2.08 [0.04;115.62]	ZOT

Table S 11.7. League table: Dropouts due to any adverse effects

AMI: amisulpride; ARI: aripiprazole; CLOZ: clozapine; CPZ: chlorpromazine; DUL: duloxetine; FLUPH: fluphenazine; FLUPHLA: fluphenazine decanoate; FLUV: fluvoxamine; GinkBil: Ginkgo biloba; GLY: glycine; HAL: haloperidol; LAM: lamotrigine; LEV: levomepromazine; LI: lithium; MEM: memantine; MODF: modafinil; Nacetyl: N-acetylcysteine; OLA: olanzapine; ONDAST: ondansetron; PALIP: paliperidone; PBO: placebo; PIMOZ: pimozide; PIPOTLA: pipotiazine long-acting; QUE: quetiapine; RISP: risperidone; SER: sertraline; SERTIND: sertindole; SUL: sulpiride; THIO: thioridazine; TOPIR: topiramate; TRIFLUO: trifluoperazine; ZIP: ziprasidone; ZOT: zotepine.

11.8. League table: Drop-outs due to inefficacy

AMI	-	-	-	-	-	-	-	-	-	-	-	-	-	-	-	1.00 [0.35;2.84]	-	-	-	-	-	-	-	-	-	-	-	-	-	-	-
11.23 [0.38;331.02]	AMI+CLOZ	-	-	-	-	-	0.17 [0.01;4.05]	-	-	-	-	-	-	-	-	-	-	-	-	-	-	-	-	-	-	-	-	-	-	-	-
2.40 [0.15;39.32]	0.21 [0.00;13.33]	AMI+OLA	-	-	-	-	-	-	-	-	-	-	-	-	-	0.42 [0.03;5.58]	-	-	-	-	-	-	-	-	-	-	-	-	-	-	-
0.61 [0.05;7.93]	0.05 [0.00;2.78]	0.25 [0.01;8.37]	ARI+CLOZ	-	-	-	3.08 [0.31;31.01]	-	-	-	-	-	-	-	-	-	-	-	-	-	-	-	-	-	-	-	-	-	-	-	-
0.40 [0.06;2.65]	0.04 [0.00;1.25]	0.17 [0.01;3.46]	0.65 [0.04;10.77]	ARI+HAL	-	-	-	-	-	-	-	-	1.00 [0.22;4.62]	-	-	-	-	-	-	-	-	-	-	-	-	-	-	-	-	-	-
11.66 [0.36;375.85]	1.04 [0.01;101.35]	4.86 [0.07;326.32]	19.19 [0.35;1065.01]	29.44 [0.76;1133.96]	BEZOAET+CLO	-	0.16 [0.01;4.29]	-	-	-	-	-	-	-	-	-	-	-	-	-	-	-	-	-	-	-	-	-	-	-	-
2.25 [0.16;31.64]	0.20 [0.00;10.99]	0.94 [0.03;32.76]	3.71 [0.13;104.90]	5.69 [0.32;100.14]	0.19 [0.00;11.42]	CEREBR+RISP	-	-	-	-	-	-	-	-	-	-	-	-	-	-	-	-	-	0.31 [0.03;3.31]	-	-	-	-	-	-	-
1.87 [0.61;5.76]	0.17 [0.01;4.05]	0.78 [0.06;10.79]	3.08 [0.31;31.01]	4.73 [0.96;23.20]	0.16 [0.01;4.29]	0.83 [0.07;9.29]	CLOZ	0.37 [0.10;1.38]	3.15 [0.12;85.51]	-	-	-	0.17 [0.09;0.31]	0.31 [0.01;8.61]	-	0.73 [0.44;1.22]	-	-	-	-	4.81 [0.21;109.71]	-	0.18 [0.05;0.63]	0.31 [0.16;0.62]	0.44 [0.04;5.40]	-	0.64 [0.09;4.48]	-	0.65 [0.10;4.29]	0.47 [0.04;5.99]	0.09 [0.00;1.81]
1.12 [0.25;5.08]	0.10 [0.00;2.89]	0.47 [0.03;7.80]	1.85 [0.15;23.51]	2.84 [0.43;18.92]	0.10 [0.00;3.05]	0.50 [0.04;6.92]	0.60 [0.21;1.74]	CPZ	-	-	-	-	-	-	5.57 [0.24;129.51]	0.37 [0.06;2.18]	-	-	-	-	-	-	-	-	-	-	-	-	-	-	-
5.90 [0.18;192.79]	0.53 [0.01;51.82]	2.46 [0.04;167.00]	9.71 [0.17;545.32]	14.90 [0.38;581.29]	0.51 [0.00;53.31]	2.62 [0.04;156.32]	3.15 [0.12;85.51]	5.25 [0.16;168.45]	DUL+CLOZ	-	-	-	-	-	-	-	-	-	-	-	-	-	-	-	-	-	-	-	-	-	-
0.15 [0.03;0.77]	0.01 [0.00;0.41]	0.06 [0.00;1.12]	0.25 [0.02;3.42]	0.38 [0.05;2.70]	0.01 [0.00;0.43]	0.07 [0.00;0.96]	0.08 [0.02;0.28]	0.13 [0.03;0.68]	0.03 [0.00;0.87]	FLUPH	-	-	1.57 [0.22;11.03]	-	-	-	-	-	-	-	-	-	4.80 [0.80;28.86]	5.33 [0.90;31.69]	-	-	-	-	-	-	-
0.92 [0.03;26.94]	0.08 [0.00;7.60]	0.39 [0.01;23.81]	1.52 [0.03;79.20]	2.33 [0.07;79.35]	0.08 [0.00;7.82]	0.41 [0.01;22.32]	0.49 [0.02;12.21]	0.82 [0.03;23.93]	0.16 [0.00;15.61]	6.19 [0.20;187.41]	FLUPHLA	-	-	-	-	-	-	-	-	0.11 [0.02;0.52]	-	0.64 [0.20;2.10]	-	-	-	-	-	-	-	-	-
0.85 [0.06;13.08]	0.08 [0.00;4.45]	0.36 [0.01;13.25]	1.41 [0.05;42.97]	2.16 [0.12;39.87]	0.07 [0.00;4.61]	0.38 [0.01;12.19]	0.46 [0.04;5.69]	0.76 [0.05;11.62]	0.14 [0.00;9.21]	5.72 [0.36;91.81]	0.92 [0.02;52.17]	GinkBil+HAL	0.46 [0.04;5.56]	-	-	-	-	-	-	-	-	-	-	-	-	-	-	-	-	-	-
0.40 [0.13;1.23]	0.04 [0.00;0.88]	0.17 [0.01;2.29]	0.65 [0.06;6.84]	1.00 [0.22;4.62]	0.03 [0.00;0.94]	0.18 [0.02;1.99]	0.21 [0.14;0.33]	0.35 [0.11;1.08]	0.07 [0.00;1.87]	2.65 [0.77;9.15]	0.43 [0.02;10.28]	0.46 [0.04;5.56]	HAL	-	-	1.97 [1.14;3.38]	-	1.16 [0.27;5.01]	-	0.25 [0.02;3.96]	-	-	0.70 [0.24;2.05]	3.08 [1.08;8.82]	-	-	-	-	-	-	-
0.59 [0.02;19.40]	0.05 [0.00;5.20]	0.25 [0.00;16.78]	0.97 [0.02;54.80]	1.49 [0.04;58.46]	0.05 [0.00;5.35]	0.26 [0.00;15.71]	0.31 [0.01;8.61]	0.52 [0.02;16.95]	0.10 [0.00;10.68]	3.94 [0.11;136.33]	0.64 [0.01;63.93]	0.69 [0.01;44.19]	1.49 [0.05;41.88]	LAM+CLOZ	-	-	-	-	-	-	-	-	-	-	-	-	-	-	-	-	-
6.26 [0.19;205.01]	0.56 [0.01;55.79]	2.61 [0.04;177.49]	10.30 [0.18;588.41]	15.80 [0.40;622.89]	0.54 [0.01;57.39]	2.78 [0.05;167.76]	3.34 [0.12;92.63]	5.57 [0.24;129.51]	1.06 [0.01;114.51]	41.91 [1.21;1454.41]	6.77 [0.07;680.91]	7.33 [0.11;470.78]	15.80 [0.56;446.24]	10.64 [0.10;1157.45]	LEV	-	-	-	-	-	-	-	-	-	-	-	-	-	-	-	-
1.00 [0.35;2.84]	0.09 [0.00;2.22]	0.42 [0.03;5.58]	1.65 [0.16;17.19]	2.52 [0.52;12.36]	0.09 [0.00;2.35]	0.44 [0.04;5.02]	0.53 [0.35;0.81]	0.89 [0.30;2.64]	0.17 [0.01;4.71]	6.70 [1.88;23.90]	1.08 [0.04;26.71]	1.17 [0.09;14.55]	2.52 [1.64;3.88]	1.70 [0.06;47.78]	0.16 [0.01;4.46]	OLA	0.62 [0.12;3.14]	-	0.35 [0.03;3.71]	-	-	-	0.73 [0.19;2.78]	1.31 [0.44;3.90]	-	-	-	-	-	-	-
0.95 [0.22;4.15]	0.08 [0.00;2.44]	0.40 [0.02;6.47]	1.56 [0.12;19.79]	2.40 [0.37;15.51]	0.08 [0.00;2.57]	0.42 [0.03;5.40]	0.51 [0.18;1.46]	0.85 [0.19;3.69]	0.16 [0.01;5.14]	6.36 [1.32;30.52]	1.03 [0.04;29.37]	1.11 [0.07;16.62]	2.40 [0.82;7.00]	1.61 [0.05;52.09]	0.15 [0.00;4.90]	0.95 [0.34;2.69]	OLA+RISP	-	-	-	-	-	-	0.59 [0.18;1.98]	-	-	-	-	-	-	-
0.46 [0.07;2.92]	0.04 [0.00;1.41]	0.19 [0.01;3.88]	0.76 [0.05;12.07]	1.16 [0.14;9.63]	0.04 [0.00;1.48]	0.20 [0.01;3.46]	0.25 [0.05;1.13]	0.41 [0.07;2.59]	0.08 [0.00;2.96]	3.09 [0.46;20.92]	0.50 [0.02;16.46]	0.54 [0.03;9.62]	1.16 [0.27;5.01]	0.78 [0.02;29.97]	0.07 [0.00;2.82]	0.46 [0.10;2.11]	0.49 [0.08;2.97]	ONDAST+HAL	-	-	-	-	-	-	-	-	-	-	-	-	-
0.35 [0.03;4.62]	0.03 [0.00;1.69]	0.15 [0.00;4.86]	0.58 [0.02;16.06]	0.88 [0.05;15.20]	0.03 [0.00;1.75]	0.16 [0.01;4.58]	0.19 [0.02;2.05]	0.31 [0.02;4.19]	0.06 [0.00;3.50]	2.34 [0.16;34.23]	0.38 [0.01;20.29]	0.41 [0.01;12.94]	0.88 [0.08;9.73]	0.59 [0.01;35.42]	0.06 [0.00;3.31]	0.35 [0.03;3.71]	0.37 [0.03;4.86]	0.76 [0.05;12.58]	PALIP	-	-	-	-	-	-	-	-	-	-	-	-
0.10 [0.01;1.96]	0.01 [0.00;0.61]	0.04 [0.00;1.87]	0.16 [0.00;6.12]	0.25 [0.01;5.87]	0.01 [0.00;0.63]	0.04 [0.00;1.73]	0.05 [0.00;0.87]	0.09 [0.00;1.74]	0.02 [0.00;1.27]	0.66 [0.03;13.68]	0.11 [0.02;0.52]	0.12 [0.00;4.75]	0.25 [0.02;3.96]	0.17 [0.00;12.82]	0.02 [0.00;1.21]	0.10 [0.01;1.62]	0.10 [0.01;2.02]	0.21 [0.01;4.88]	0.28 [0.01;10.97]	PBO	-	6.00 [1.28;28.21]	-	-	-	-	-	0.94 [0.07;11.77]	-	-	-
9.00 [0.32;249.75]	0.80 [0.01;69.91]	3.75 [0.06;222.79]	14.82 [0.30;722.76]	22.73 [0.68;759.29]	0.77 [0.01;72.06]	3.99 [0.08;207.65]	4.81 [0.21;109.71]	8.02 [0.29;218.02]	1.53 [0.02;143.81]	60.30 [2.07;1759.49]	9.74 [0.11;859.41]	10.54 [0.19;585.59]	22.73 [0.97;534.54]	15.31 [0.16;1453.97]	1.44 [0.02;137.75]	9.01 [0.38;211.04]	9.49 [0.35;257.19]	19.53 [0.60;632.97]	25.73 [0.50;1322.55]	90.94 [1.37;6031.79]	PIMOZ+CLOZ	-	-	-	-	-	-	-	-	-	-
0.59 [0.02;17.12]	0.05 [0.00;4.84]	0.25 [0.00;15.17]	0.98 [0.02;50.42]	1.50 [0.04;50.46]	0.05 [0.00;4.99]	0.26 [0.00;14.21]	0.32 [0.01;7.76]	0.53 [0.02;15.21]	0.10 [0.00;9.95]	3.98 [0.13;119.14]	0.64 [0.20;2.10]	0.70 [0.01;38.88]	1.50 [0.06;35.56]	1.01 [0.01;100.62]	0.09 [0.00;9.47]	0.59 [0.02;14.50]	0.63 [0.02;17.70]	1.29 [0.04;42.07]	1.70 [0.03;90.13]	6.00 [1.28;28.21]	0.07 [0.00;5.77]	PIPOTLA	-	-	-	-	-	-	-	-	-
0.43 [0.12;1.54]	0.04 [0.00;1.00]	0.18 [0.01;2.64]	0.70 [0.06;7.92]	1.07 [0.20;5.82]	0.04 [0.00;1.06]	0.19 [0.02;2.27]	0.23 [0.11;0.48]	0.38 [0.11;1.36]	0.07 [0.00;2.12]	2.85 [0.78;10.34]	0.46 [0.02;11.96]	0.50 [0.04;6.61]	1.07 [0.52;2.20]	0.72 [0.02;21.51]	0.07 [0.00;2.03]	0.43 [0.20;0.90]	0.45 [0.13;1.51]	0.92 [0.18;4.69]	1.22 [0.10;14.47]	4.30 [0.25;74.49]	0.05 [0.00;1.17]	0.72 [0.03;18.39]	QUE	1.11 [0.34;3.67]	-	-	-	-	-	-	-
0.71 [0.21;2.36]	0.06 [0.00;1.61]	0.30 [0.02;4.23]	1.17 [0.11;12.55]	1.79 [0.35;9.24]	0.06 [0.00;1.70]	0.31 [0.03;3.31]	0.38 [0.22;0.66]	0.63 [0.19;2.06]	0.12 [0.00;3.41]	4.75 [1.34;16.82]	0.77 [0.03;19.47]	0.83 [0.06;10.68]	1.79 [0.99;3.24]	1.21 [0.04;34.60]	0.11 [0.00;3.27]	0.71 [0.39;1.29]	0.75 [0.28;2.02]	1.54 [0.32;7.44]	2.03 [0.18;23.14]	7.17 [0.43;120.78]	0.08 [0.00;1.89]	1.19 [0.05;29.92]	1.67 [0.75;3.73]	RISP	-	0.74 [0.32;1.72]	-	-	-	-	-
0.83 [0.05;12.87]	0.07 [0.00;4.26]	0.35 [0.01;12.99]	1.36 [0.05;41.06]	2.09 [0.11;40.58]	0.07 [0.00;4.42]	0.37 [0.01;11.90]	0.44 [0.04;5.40]	0.74 [0.05;11.19]	0.14 [0.00;8.83]	5.55 [0.34;91.61]	0.90 [0.02;52.41]	0.97 [0.03;33.86]	2.09 [0.17;26.53]	1.41 [0.02;89.32]	0.13 [0.00;8.47]	0.83 [0.07;10.47]	0.87 [0.06;13.19]	1.80 [0.10;33.64]	2.37 [0.07;75.68]	8.37 [0.20;356.54]	0.09 [0.00;5.05]	1.40 [0.02;80.76]	1.95 [0.14;26.48]	1.17 [0.09;15.13]	RISP+CLOZ	-	-	-	-	-	-
0.53 [0.12;2.29]	0.05 [0.00;1.34]	0.22 [0.01;3.59]	0.87 [0.07;10.79]	1.33 [0.21;8.42]	0.05 [0.00;1.41]	0.23 [0.02;2.85]	0.28 [0.10;0.77]	0.47 [0.11;2.00]	0.09 [0.00;2.82]	3.54 [0.78;16.12]	0.57 [0.02;16.13]	0.62 [0.04;9.09]	1.33 [0.48;3.73]	0.90 [0.03;28.56]	0.08 [0.00;2.70]	0.53 [0.19;1.48]	0.56 [0.15;2.04]	1.15 [0.19;6.83]	1.51 [0.11;19.82]	5.34 [0.28;101.56]	0.06 [0.00;1.57]	0.89 [0.03;24.80]	1.24 [0.39;3.97]	0.74 [0.32;1.72]	0.64 [0.04;9.44]	SERTIND	-	-	-	-	-
1.19 [0.13;11.34]	0.11 [0.00;4.48]	0.50 [0.02;13.11]	1.96 [0.10;40.37]	3.01 [0.24;37.35]	0.10 [0.00;4.67]	0.53 [0.02;11.81]	0.64 [0.09;4.48]	1.06 [0.12;9.80]	0.20 [0.00;9.35]	7.99 [0.78;81.77]	1.29 [0.03;55.15]	1.40 [0.06;33.88]	3.01 [0.41;22.25]	2.03 [0.04;94.62]	0.19 [0.00;8.98]	1.19 [0.16;8.77]	1.26 [0.14;11.55]	2.59 [0.22;30.76]	3.41 [0.16;74.95]	12.05 [0.40;364.48]	0.13 [0.00;5.28]	2.01 [0.05;84.92]	2.81 [0.35;22.62]	1.68 [0.22;12.76]	1.44 [0.06;34.36]	2.26 [0.25;20.26]	SERTIND+CLOZ	-	-	-	-
0.09 [0.00;4.64]	0.01 [0.00;1.16]	0.04 [0.00;3.76]	0.15 [0.00;12.72]	0.23 [0.00;13.39]	0.01 [0.00;1.18]	0.04 [0.00;3.57]	0.05 [0.00;2.15]	0.08 [0.00;4.12]	0.02 [0.00;2.36]	0.62 [0.01;32.11]	0.10 [0.01;1.98]	0.11 [0.00;9.72]	0.23 [0.01;9.92]	0.16 [0.00;23.84]	0.01 [0.00;2.24]	0.09 [0.00;4.03]	0.10 [0.00;4.81]	0.20 [0.00;11.21]	0.27 [0.00;22.66]	0.94 [0.07;11.77]	0.01 [0.00;1.38]	0.16 [0.01;3.03]	0.22 [0.00;9.89]	0.13 [0.00;5.80]	0.11 [0.00;10.34]	0.18 [0.00;8.54]	0.08 [0.00;5.43]	TRIFLUO	-	-	-
1.21 [0.13;10.94]	0.11 [0.00;4.41]	0.51 [0.02;12.86]	2.00 [0.10;39.46]	3.06 [0.26;36.23]	0.10 [0.00;4.61]	0.54 [0.03;11.55]	0.65 [0.10;4.29]	1.08 [0.12;9.45]	0.21 [0.00;9.21]	8.12 [0.84;79.00]	1.31 [0.03;54.33]	1.42 [0.06;33.20]	3.06 [0.44;21.32]	2.06 [0.05;93.28]	0.19 [0.00;8.85]	1.21 [0.18;8.40]	1.28 [0.15;11.13]	2.63 [0.23;29.82]	3.47 [0.16;73.34]	12.25 [0.42;357.94]	0.13 [0.00;5.20]	2.04 [0.05;83.65]	2.85 [0.37;21.72]	1.71 [0.24;12.24]	1.46 [0.06;33.65]	2.30 [0.27;19.51]	1.02 [0.07;15.37]	13.07 [0.19;887.03]	ZIP	-	-
0.89 [0.06;14.23]	0.08 [0.00;4.66]	0.37 [0.01;14.25]	1.46 [0.05;45.10]	2.24 [0.11;44.75]	0.08 [0.00;4.83]	0.39 [0.01;13.07]	0.47 [0.04;5.99]	0.79 [0.05;12.37]	0.15 [0.00;9.65]	5.94 [0.35;101.20]	0.96 [0.02;57.32]	1.04 [0.03;37.15]	2.24 [0.17;29.40]	1.51 [0.02;97.65]	0.14 [0.00;9.26]	0.89 [0.07;11.60]	0.93 [0.06;14.58]	1.92 [0.10;37.11]	2.53 [0.08;83.09]	8.95 [0.21;390.69]	0.10 [0.00;5.52]	1.49 [0.03;88.33]	2.08 [0.15;29.32]	1.25 [0.09;16.76]	1.07 [0.03;37.74]	1.68 [0.11;25.70]	0.74 [0.03;18.24]	9.55 [0.10;899.31]	0.73 [0.03;17.30]	ZIP+CLOZ	-
0.17 [0.01;4.15]	0.02 [0.00;1.21]	0.07 [0.00;3.81]	0.29 [0.01;12.28]	0.44 [0.02;12.72]	0.01 [0.00;1.25]	0.08 [0.00;3.54]	0.09 [0.00;1.81]	0.15 [0.01;3.62]	0.03 [0.00;2.49]	1.16 [0.05;29.31]	0.19 [0.00;14.85]	0.20 [0.00;9.99]	0.44 [0.02;8.81]	0.29 [0.00;25.17]	0.03 [0.00;2.39]	0.17 [0.01;3.48]	0.18 [0.01;4.27]	0.38 [0.01;10.59]	0.50 [0.01;22.51]	1.75 [0.03;103.47]	0.02 [0.00;1.44]	0.29 [0.00;22.90]	0.41 [0.02;8.71]	0.24 [0.01;5.01]	0.21 [0.00;10.16]	0.33 [0.01;7.55]	0.15 [0.00;5.07]	1.87 [0.02;226.93]	0.14 [0.00;4.83]	0.20 [0.00;9.73]	ZOT

Table S 11.8. League table: Dropouts due to inefficacy

AMI: amisulpride; ARI: aripiprazole; BEZOAET: benzoate sodium; CEREBR: cerebrolysin; CLOZ: clozapine; CPZ: chlorpromazine; DUL: duloxetine; FLUPH: fluphenazine; FLUPHLA: fluphenazine decanoate; GinkBil: Ginkgo biloba; haloperidol; HAL: haloperidol; LAM: lamotrigine; LEV: levomepromazine; LI: lithium; OLA: olanzapine; ONDAST: ondansetron; PALIP: paliperidone; PBO: placebo; PIMOZ: pimozide; PIPOTLA: pipotiazine long-acting; QUE: quetiapine; RISP: risperidone; SERTIND: sertindole; TRIFLUO: trifluoperazine; ZIP: ziprasidone; ZOT: zotepine.

11.9. League table: Total number of participants with adverse effects

AMI+CLOZ	-	-	3.45 [1.21;9.84]	-	-	-	-	-	-	-	-	-	-	-	-	-	-	-	-	-
2.77 [0.28;27.82]	AMI+OLA	-	-	-	-	-	-	-	-	3.12 [0.46;21.08]	-	-	-	-	-	-	-	-	-	-
2.06 [0.60;7.05]	0.74 [0.09;6.41]	ARI+CLOZ	1.67 [0.88;3.19]	-	-	-	-	-	-	-	-	-	-	-	-	-	-	-	-	-
3.45 [1.21;9.84]	1.25 [0.16;9.73]	1.67 [0.88;3.19]	CLOZ	1.04 [0.49;2.19]	-	-	-	1.17 [0.38;3.61]	0.16 [0.01;3.90]	2.51 [1.16;5.43]	-	-	1.54 [0.42;5.61]	2.40 [0.71;8.13]	0.67 [0.23;1.93]	-	-	-	-	1.46 [0.65;3.28]
3.60 [1.05;12.37]	1.30 [0.15;11.01]	1.75 [0.70;4.37]	1.04 [0.55;2.00]	CPZ	0.60 [0.12;2.91]	-	-	-	-	-	-	-	1.88 [1.20;2.93]	-	-	-	2.34 [0.84;6.57]	-	-	-
2.16 [0.29;16.01]	0.78 [0.05;11.10]	1.05 [0.17;6.50]	0.63 [0.11;3.45]	0.60 [0.12;2.91]	CPZ+TRIFLUO	-	-	-	-	-	-	-	-	-	-	-	-	-	0.34 [0.09;1.24]	-
8.60 [1.38;53.60]	3.10 [0.25;38.65]	4.17 [0.81;21.37]	2.49 [0.56;11.17]	2.39 [0.60;9.55]	3.98 [0.49;32.48]	FLUPH	0.71 [0.21;2.34]	-	-	-	-	-	-	-	-	-	-	-	-	-
6.07 [1.52;24.20]	2.19 [0.24;20.15]	2.95 [0.97;8.93]	1.76 [0.71;4.34]	1.68 [0.84;3.38]	2.81 [0.50;15.75]	0.71 [0.21;2.34]	HAL	-	-	-	1.11 [0.44;2.78]	8.40 [0.86;81.87]	1.12 [0.64;1.94]	-	-	-	-	0.54 [0.13;2.24]	-	-
4.02 [0.86;18.79]	1.45 [0.14;15.17]	1.95 [0.53;7.17]	1.17 [0.38;3.61]	1.12 [0.30;4.11]	1.86 [0.24;14.41]	0.47 [0.07;3.06]	0.66 [0.16;2.82]	MEM+CLOZ	-	-	-	-	-	-	-	-	-	-	-	-
0.56 [0.02;15.93]	0.20 [0.00;8.91]	0.27 [0.01;6.97]	0.16 [0.01;3.90]	0.16 [0.01;3.99]	0.26 [0.01;9.56]	0.06 [0.00;2.19]	0.09 [0.00;2.52]	0.14 [0.00;4.06]	MET+CLOZ	-	-	-	-	-	-	-	-	-	-	-
8.65 [2.37;31.63]	3.13 [0.46;21.08]	4.20 [1.55;11.41]	2.51 [1.17;5.38]	2.40 [0.92;6.26]	4.00 [0.63;25.36]	1.01 [0.19;5.23]	1.43 [0.46;4.42]	2.15 [0.55;8.40]	15.49 [0.59;408.66]	OLA	-	-	1.40 [0.31;6.35]	2.18 [0.51;9.30]	-	-	-	-	-	-
6.74 [1.28;35.47]	2.43 [0.22;26.87]	3.27 [0.78;13.82]	1.95 [0.54;7.08]	1.87 [0.59;5.93]	3.12 [0.44;22.01]	0.78 [0.17;3.55]	1.11 [0.44;2.78]	1.68 [0.30;9.29]	12.07 [0.39;373.80]	0.78 [0.18;3.35]	ONDAST+HAL	-	-	-	-	-	-	-	-	-
50.97 [3.55;731.73]	18.41 [0.77;442.29]	24.74 [1.97;311.50]	14.77 [1.28;171.09]	14.15 [1.31;153.07]	23.58 [1.36;410.25]	5.93 [0.45;77.71]	8.40 [0.86;81.87]	12.66 [0.85;187.90]	91.26 [1.65;5061.56]	5.89 [0.46;74.87]	7.56 [0.65;88.06]	PBO	-	-	-	-	-	0.06 [0.01;0.64]	-	-
6.77 [1.91;24.06]	2.44 [0.29;20.97]	3.29 [1.26;8.60]	1.96 [0.96;4.01]	1.88 [1.23;2.87]	3.13 [0.61;16.04]	0.79 [0.21;2.95]	1.12 [0.64;1.94]	1.68 [0.44;6.40]	12.12 [0.46;316.18]	0.78 [0.29;2.10]	1.00 [0.34;2.93]	0.13 [0.01;1.38]	QUE	1.56 [0.35;6.90]	-	-	-	-	-	-
10.59 [2.22;50.46]	3.82 [0.39;37.92]	5.14 [1.37;19.34]	3.07 [0.96;9.76]	2.94 [0.84;10.33]	4.90 [0.65;36.82]	1.23 [0.20;7.67]	1.74 [0.44;6.94]	2.63 [0.52;13.25]	18.95 [0.64;560.23]	1.22 [0.34;4.37]	1.57 [0.30;8.25]	0.21 [0.01;2.98]	1.56 [0.44;5.55]	RISP	-	0.93 [0.45;1.91]	-	-	-	-
2.32 [0.52;10.27]	0.84 [0.08;8.45]	1.13 [0.33;3.88]	0.67 [0.23;1.93]	0.64 [0.19;2.23]	1.07 [0.14;7.98]	0.27 [0.04;1.69]	0.38 [0.10;1.53]	0.58 [0.12;2.71]	4.16 [0.15;118.80]	0.27 [0.07;0.99]	0.34 [0.07;1.82]	0.05 [0.00;0.66]	0.34 [0.10;1.23]	0.22 [0.05;1.05]	RISP+CLOZ	-	-	-	-	-
9.87 [1.77;55.08]	3.56 [0.32;39.46]	4.79 [1.06;21.64]	2.86 [0.73;11.18]	2.74 [0.64;11.66]	4.57 [0.54;38.87]	1.15 [0.16;8.19]	1.63 [0.34;7.72]	2.45 [0.42;14.40]	17.67 [0.55;563.21]	1.14 [0.26;4.92]	1.46 [0.24;8.93]	0.19 [0.01;3.05]	1.46 [0.34;6.25]	0.93 [0.45;1.91]	4.25 [0.76;23.82]	SERTIND	-	-	-	-
8.44 [1.69;42.14]	3.05 [0.28;32.67]	4.10 [1.03;16.28]	2.45 [0.72;8.28]	2.34 [0.84;6.57]	3.91 [0.59;25.72]	0.98 [0.17;5.53]	1.39 [0.40;4.83]	2.10 [0.40;11.05]	15.11 [0.50;456.46]	0.98 [0.24;3.99]	1.25 [0.27;5.88]	0.17 [0.01;2.22]	1.25 [0.41;3.80]	0.80 [0.16;4.05]	3.64 [0.73;18.23]	0.86 [0.14;5.06]	SUL	-	-	-
3.30 [0.46;23.87]	1.19 [0.09;16.57]	1.60 [0.27;9.67]	0.96 [0.18;5.12]	0.92 [0.19;4.44]	1.53 [0.16;14.21]	0.38 [0.06;2.45]	0.54 [0.13;2.24]	0.82 [0.11;6.20]	5.91 [0.16;215.88]	0.38 [0.06;2.33]	0.49 [0.09;2.64]	0.06 [0.01;0.64]	0.49 [0.11;2.23]	0.31 [0.04;2.25]	1.42 [0.20;10.32]	0.33 [0.04;2.74]	0.39 [0.06;2.57]	THIO	-	-
0.74 [0.07;7.99]	0.27 [0.01;5.10]	0.36 [0.04;3.35]	0.21 [0.03;1.82]	0.20 [0.03;1.57]	0.34 [0.09;1.24]	0.09 [0.01;1.01]	0.12 [0.01;1.05]	0.18 [0.02;2.06]	1.32 [0.03;61.10]	0.09 [0.01;0.81]	0.11 [0.01;1.14]	0.01 [0.00;0.33]	0.11 [0.01;0.87]	0.07 [0.01;0.76]	0.32 [0.03;3.45]	0.07 [0.01;0.91]	0.09 [0.01;0.86]	0.22 [0.02;2.94]	TRIFLUO	-
5.05 [1.34;18.97]	1.82 [0.20;16.60]	2.45 [0.87;6.90]	1.46 [0.65;3.28]	1.40 [0.50;3.96]	2.34 [0.35;15.43]	0.59 [0.11;3.23]	0.83 [0.25;2.80]	1.25 [0.31;5.03]	9.04 [0.34;241.10]	0.58 [0.19;1.77]	0.75 [0.16;3.43]	0.10 [0.01;1.31]	0.75 [0.25;2.19]	0.48 [0.12;1.96]	2.17 [0.58;8.21]	0.51 [0.10;2.50]	0.60 [0.14;2.58]	1.53 [0.24;9.84]	6.85 [0.70;67.58]	ZIP

Table S 11.9. League table: Total number of participants with adverse effects

AMI: amisulpride; ARI: aripiprazole; CLOZ: clozapine; CPZ: chlorpromazine; FLUPH: fluphenazine; HAL: haloperidol; MEM: memantine; MET: metformin; OLA: olanzapine; ONDAST: ondansetron; PBO: placebo; QUE: quetiapine; RISP: risperidone; SERTIND: sertindole; SUL: sulpiride; THIO: thioridazine; TRIFLUO: trifluoperazine; ZIP: ziprasidone

11.10. League table: Antiparkinsonian medication use

AMI	-	-	-	-	-	-	-	-	-	-	-	1.00 [0.28;3.63]	-	-	-	-	-	-	-	-	-	-	-	-
1.65 [0.27;10.19]	ARI+CLOZ	-	0.67 [0.21;2.18]	-	-	-	-	-	-	-	-	-	-	-	-	-	-	-	-	-	-	-	-	-
0.33 [0.02;6.68]	0.20 [0.01;3.89]	ARI+HAL	-	-	-	-	0.49 [0.04;5.80]	-	-	-	-	-	-	-	-	-	-	-	-	-	-	-	-	-
1.10 [0.28;4.42]	0.67 [0.21;2.18]	3.30 [0.22;49.40]	CLOZ	-	-	-	-	-	-	0.94 [0.18;4.97]	-	1.01 [0.59;1.73]	-	-	-	-	-	0.25 [0.11;0.54]	0.36 [0.01;9.75]	-	-	-	0.72 [0.32;1.61]	0.03 [0.00;0.63]
0.63 [0.03;13.04]	0.38 [0.02;7.59]	1.89 [0.06;63.96]	0.57 [0.04;8.89]	CPZ	-	-	0.26 [0.02;3.18]	3.88 [0.79;19.12]	-	-	-	-	-	-	3.00 [0.48;18.72]	-	-	-	-	1.79 [0.63;5.10]	3.00 [0.48;18.72]	0.41 [0.10;1.67]	-	-
0.19 [0.03;1.20]	0.11 [0.02;0.68]	0.56 [0.04;8.24]	0.17 [0.04;0.66]	0.29 [0.02;4.51]	FLUPH	-	1.23 [0.32;4.71]	-	-	-	-	-	-	-	-	-	1.33 [0.22;8.18]	2.44 [0.34;17.40]	-	-	-	-	-	-
0.51 [0.01;49.03]	0.31 [0.00;28.94]	1.53 [0.01;206.46]	0.46 [0.01;36.99]	0.81 [0.03;24.63]	2.75 [0.03;218.23]	FLUPHLA	-	-	-	-	-	-	-	-	5.26 [0.26;107.92]	1.38 [0.27;7.21]	-	-	-	-	-	-	-	-
0.16 [0.03;0.89]	0.10 [0.02;0.50]	0.49 [0.04;5.80]	0.15 [0.05;0.45]	0.26 [0.02;3.18]	0.88 [0.30;2.60]	0.32 [0.00;22.31]	HAL	-	2.00 [0.27;14.91]	-	-	-	-	-	-	-	6.20 [1.65;23.34]	1.99 [0.59;6.71]	-	-	-	-	-	-
2.45 [0.08;75.03]	1.49 [0.05;43.88]	7.34 [0.15;350.11]	2.22 [0.09;53.01]	3.88 [0.79;19.12]	13.20 [0.56;312.00]	4.81 [0.11;209.12]	14.96 [0.77;292.34]	LEV	-	-	-	-	-	-	-	-	-	-	-	-	-	-	-	-
0.33 [0.02;4.54]	0.20 [0.02;2.62]	0.98 [0.04;23.69]	0.30 [0.03;2.94]	0.52 [0.02;12.89]	1.76 [0.18;17.27]	0.64 [0.01;70.11]	2.00 [0.27;14.91]	0.13 [0.00;4.83]	LI+HAL	-	-	-	-	-	-	-	-	-	-	-	-	-	-	-
1.03 [0.12;9.05]	0.63 [0.08;4.84]	3.09 [0.13;74.28]	0.94 [0.18;4.97]	1.63 [0.07;40.43]	5.56 [0.65;47.85]	2.03 [0.02;220.22]	6.31 [0.85;46.60]	0.42 [0.01;15.16]	3.15 [0.19;53.68]	MINOC+CLOZ	-	-	-	-	-	-	-	-	-	-	-	-	-	-
1.18 [0.10;14.00]	0.72 [0.06;7.96]	3.54 [0.13;97.11]	1.07 [0.13;8.73]	1.87 [0.07;52.80]	6.37 [0.61;66.54]	2.32 [0.02;276.10]	7.22 [0.80;65.48]	0.48 [0.01;19.53]	3.61 [0.18;71.27]	1.14 [0.08;16.66]	MIRT+RISP	-	-	-	-	-	-	0.29 [0.04;2.08]	-	-	-	-	-	-
1.00 [0.28;3.63]	0.61 [0.17;2.20]	2.99 [0.20;44.64]	0.91 [0.54;1.51]	1.58 [0.10;24.42]	5.38 [1.39;20.79]	1.96 [0.02;156.43]	6.10 [2.04;18.26]	0.41 [0.02;9.69]	3.05 [0.31;30.07]	0.97 [0.17;5.54]	0.85 [0.10;6.96]	OLA	0.83 [0.17;4.13]	-	-	-	1.12 [0.26;4.83]	0.32 [0.09;1.08]	-	-	-	-	-	-
0.54 [0.10;2.89]	0.33 [0.07;1.65]	1.62 [0.10;27.16]	0.49 [0.16;1.47]	0.86 [0.05;14.84]	2.92 [0.61;14.06]	1.06 [0.01;91.15]	3.31 [0.85;12.83]	0.22 [0.01;5.80]	1.65 [0.15;18.66]	0.52 [0.07;3.85]	0.46 [0.05;4.23]	0.54 [0.19;1.57]	OLA+RISP	-	-	-	-	0.80 [0.24;2.67]	-	-	-	-	-	-
0.41 [0.06;3.01]	0.25 [0.04;1.68]	1.24 [0.06;24.09]	0.38 [0.08;1.67]	0.66 [0.03;13.14]	2.23 [0.36;13.86]	0.81 [0.01;76.69]	2.53 [0.49;13.05]	0.17 [0.01;5.04]	1.27 [0.09;16.92]	0.40 [0.04;3.75]	0.35 [0.03;3.83]	0.41 [0.09;1.87]	0.77 [0.15;4.04]	OXYT+RISP	-	-	-	0.81 [0.22;3.08]	-	-	-	-	-	-
2.73 [0.09;83.68]	1.65 [0.06;48.93]	8.16 [0.17;390.31]	2.47 [0.10;59.13]	4.31 [0.87;21.39]	14.68 [0.62;348.03]	5.35 [0.26;109.62]	16.64 [0.85;326.17]	1.11 [0.12;10.66]	8.32 [0.23;301.52]	2.64 [0.07;95.17]	2.31 [0.06;93.62]	2.73 [0.11;65.04]	5.03 [0.19;132.37]	6.57 [0.22;196.60]	PBO	0.25 [0.01;5.40]	-	-	-	-	1.00 [0.12;8.27]	0.08 [0.02;0.29]	-	-
0.70 [0.01;69.48]	0.43 [0.00;41.02]	2.11 [0.02;292.02]	0.64 [0.01;52.48]	1.11 [0.04;35.20]	3.79 [0.05;309.58]	1.38 [0.26;7.19]	4.30 [0.06;306.67]	0.29 [0.01;12.89]	2.15 [0.02;240.26]	0.68 [0.01;75.92]	0.60 [0.00;72.64]	0.70 [0.01;57.76]	1.30 [0.01;114.42]	1.70 [0.02;164.31]	0.26 [0.01;5.51]	PIPOTLA	-	-	-	-	-	-	-	-
0.70 [0.13;3.74]	0.43 [0.08;2.15]	2.11 [0.15;30.14]	0.64 [0.21;1.93]	1.11 [0.08;16.50]	3.79 [1.10;13.08]	1.38 [0.02;107.34]	4.29 [1.60;11.54]	0.29 [0.01;6.58]	2.15 [0.23;20.14]	0.68 [0.09;5.03]	0.60 [0.06;5.60]	0.70 [0.24;2.03]	1.30 [0.33;5.18]	1.70 [0.31;9.20]	0.26 [0.01;5.94]	1.00 [0.01;79.91]	QUE	1.83 [0.24;14.13]	-	-	-	-	-	-
0.34 [0.08;1.47]	0.21 [0.05;0.80]	1.01 [0.07;14.32]	0.31 [0.16;0.60]	0.53 [0.04;7.84]	1.82 [0.52;6.35]	0.66 [0.01;51.21]	2.06 [0.79;5.38]	0.14 [0.01;3.13]	1.03 [0.11;9.55]	0.33 [0.05;1.97]	0.29 [0.04;2.08]	0.34 [0.17;0.69]	0.62 [0.23;1.69]	0.81 [0.22;3.08]	0.12 [0.01;2.83]	0.48 [0.01;38.12]	0.48 [0.17;1.36]	RISP	-	-	-	-	-	-
0.39 [0.01;14.21]	0.24 [0.01;8.00]	1.18 [0.02;84.48]	0.36 [0.01;9.75]	0.62 [0.01;45.64]	2.11 [0.06;75.69]	0.77 [0.00;186.77]	2.40 [0.07;78.46]	0.16 [0.00;15.67]	1.20 [0.02;67.12]	0.38 [0.01;15.46]	0.33 [0.01;16.69]	0.39 [0.01;11.19]	0.72 [0.02;23.65]	0.95 [0.03;35.68]	0.14 [0.00;14.12]	0.56 [0.00;138.25]	0.56 [0.02;18.27]	1.16 [0.04;34.02]	RISP+CLOZ	-	-	-	-	-
1.13 [0.05;27.77]	0.68 [0.03;16.20]	3.38 [0.09;133.05]	1.02 [0.05;19.26]	1.79 [0.63;5.10]	6.08 [0.33;113.28]	2.21 [0.06;79.08]	6.89 [0.45;104.40]	0.46 [0.07;3.10]	3.44 [0.12;101.16]	1.09 [0.04;31.92]	0.95 [0.03;31.62]	1.13 [0.06;21.18]	2.08 [0.10;43.44]	2.72 [0.11;65.13]	0.41 [0.06;2.81]	1.60 [0.04;59.21]	1.60 [0.09;28.94]	3.34 [0.19;59.67]	2.87 [0.03;239.44]	SUL	-	-	-	-
1.90 [0.06;65.19]	1.15 [0.03;38.17]	5.67 [0.11;300.23]	1.72 [0.06;46.46]	3.00 [0.48;18.72]	10.21 [0.38;273.54]	3.72 [0.10;133.07]	11.57 [0.52;258.28]	0.77 [0.07;8.77]	5.79 [0.14;233.69]	1.84 [0.05;73.77]	1.60 [0.04;72.33]	1.90 [0.07;51.11]	3.50 [0.12;103.65]	4.57 [0.14;153.28]	0.70 [0.10;4.73]	2.69 [0.07;99.64]	2.69 [0.10;70.13]	5.61 [0.22;144.78]	4.83 [0.05;515.48]	1.68 [0.20;13.86]	THIO	0.14 [0.02;0.79]	-	-
0.22 [0.01;6.19]	0.14 [0.01;3.62]	0.67 [0.02;29.23]	0.20 [0.01;4.34]	0.35 [0.09;1.39]	1.20 [0.06;25.53]	0.44 [0.02;11.54]	1.36 [0.08;23.75]	0.09 [0.01;0.75]	0.68 [0.02;22.42]	0.22 [0.01;7.07]	0.19 [0.01;6.98]	0.22 [0.01;4.77]	0.41 [0.02;9.74]	0.54 [0.02;14.53]	0.08 [0.02;0.29]	0.32 [0.01;8.67]	0.32 [0.02;6.53]	0.66 [0.03;13.47]	0.57 [0.01;51.70]	0.20 [0.04;1.11]	0.12 [0.02;0.66]	TRIFLUO	-	-
0.79 [0.16;3.94]	0.48 [0.12;2.01]	2.37 [0.14;39.90]	0.72 [0.32;1.61]	1.25 [0.07;21.79]	4.26 [0.88;20.71]	1.55 [0.02;133.64]	4.83 [1.23;18.95]	0.32 [0.01;8.51]	2.42 [0.21;27.43]	0.77 [0.12;4.88]	0.67 [0.07;6.32]	0.79 [0.31;2.06]	1.46 [0.38;5.68]	1.91 [0.35;10.38]	0.29 [0.01;7.68]	1.12 [0.01;99.42]	1.13 [0.29;4.41]	2.34 [0.82;6.68]	2.02 [0.07;60.75]	0.70 [0.03;14.71]	0.42 [0.01;12.42]	3.54 [0.15;84.16]	ZIP	-
0.04 [0.00;0.95]	0.02 [0.00;0.53]	0.11 [0.00;5.98]	0.03 [0.00;0.63]	0.06 [0.00;3.24]	0.20 [0.01;5.03]	0.07 [0.00;14.18]	0.23 [0.01;5.16]	0.02 [0.00;1.13]	0.11 [0.00;4.66]	0.04 [0.00;1.04]	0.03 [0.00;1.15]	0.04 [0.00;0.72]	0.07 [0.00;1.55]	0.09 [0.00;2.38]	0.01 [0.00;1.02]	0.05 [0.00;10.51]	0.05 [0.00;1.20]	0.11 [0.01;2.21]	0.10 [0.00;7.85]	0.03 [0.00;2.08]	0.02 [0.00;1.61]	0.17 [0.00;11.51]	0.05 [0.00;0.97]	ZOT

Table S 11.10. League table: Antiparkinsonian medication use

AMI: amisulpride; ARI: aripiprazole; CLOZ: clozapine; CPZ: chlorpromazine; FLUPH: fluphenazine; FLUPHLA: fluphenazine decanoate; HAL: haloperidol; LEV: levomepromazine; LI: lithium; MINOC: minocycline; MIRT: mirtazapine; OLA: olanzapine; OXYT: oxytocin; PBO: placebo; PIPOTLA: pipotiazine long-acting; QUE: quetiapine; RISP: risperidone; SUL: sulpiride; THIO: thioridazine; TRIFLUO: trifluoperazine; ZIP: ziprasidone; ZOT: zotepine

11.11. League table: Sedation

AMI	-	-	-	-	-	-	-	-	-	-	-	-	-	0.33 [0.14;0.83]	-	-	-	-	-	-	-	-	-	-	-	-	-	-	-
1.18 [0.04;35.94]	AMI+OLA	-	-	-	-	-	-	-	-	-	-	-	-	0.28 [0.01;7.67]	-	-	-	-	-	-	-	-	-	-	-	-	-	-	-
0.03 [0.00;0.41]	0.03 [0.00;1.64]	ARI+CLOZ	4.25 [0.43;42.19]	-	-	-	-	-	-	-	-	-	-	-	-	-	-	-	-	-	-	-	-	-	-	-	-	-	-
0.15 [0.06;0.38]	0.12 [0.00;3.40]	4.25 [0.43;42.19]	CLOZ	1.48 [0.92;2.40]	-	-	1.16 [0.53;2.53]	2.52 [1.55;4.11]	0.62 [0.09;4.32]	-	0.39 [0.09;1.70]	0.11 [0.00;2.36]	-	2.26 [1.50;3.41]	-	-	-	2.31 [0.95;5.60]	2.01 [1.25;3.22]	0.49 [0.19;1.29]	-	-	0.79 [0.21;3.03]	2.91 [0.60;14.18]	-	-	-	5.14 [1.64;16.16]	0.32 [0.01;8.26]
0.25 [0.09;0.69]	0.21 [0.01;5.90]	7.28 [0.71;74.43]	1.71 [1.19;2.47]	CPZ	-	-	-	0.11 [0.01;0.84]	-	0.52 [0.11;2.60]	-	-	-	1.98 [0.83;4.75]	-	-	1.42 [0.27;7.34]	1.39 [0.72;2.67]	-	-	-	-	-	1.16 [0.40;3.43]	0.75 [0.17;3.33]	1.00 [0.21;4.71]	-	1.70 [0.99;2.90]	-
0.14 [0.02;1.11]	0.12 [0.00;5.25]	4.08 [0.21;78.05]	0.96 [0.15;6.13]	0.56 [0.09;3.69]	DESMOP+RISP	-	-	-	-	-	-	-	-	-	-	-	-	-	2.22 [0.36;13.62]	-	-	-	-	-	-	-	-	-	-
0.36 [0.06;2.08]	0.30 [0.01;11.37]	10.40 [0.67;160.66]	2.45 [0.55;10.88]	1.43 [0.32;6.46]	2.55 [0.25;26.39]	FLUPH	-	-	-	-	-	-	-	-	-	-	-	1.33 [0.23;7.74]	0.71 [0.14;3.61]	-	-	-	-	-	-	-	-	-	-
0.17 [0.05;0.59]	0.14 [0.00;4.31]	4.93 [0.44;55.66]	1.16 [0.53;2.53]	0.68 [0.29;1.60]	1.21 [0.16;9.02]	0.47 [0.09;2.55]	FLUV+CLOZ	-	-	-	-	-	-	-	-	-	-	-	-	-	-	-	-	-	-	-	-	-	-
0.33 [0.11;0.95]	0.28 [0.01;7.85]	9.53 [0.92;98.85]	2.24 [1.43;3.52]	1.31 [0.74;2.31]	2.33 [0.35;15.57]	0.92 [0.19;4.31]	1.94 [0.79;4.77]	HAL	-	-	-	-	-	-	-	-	-	-	0.80 [0.22;2.97]	-	-	-	-	-	-	-	-	-	-
0.09 [0.01;0.79]	0.08 [0.00;3.56]	2.63 [0.13;53.19]	0.62 [0.09;4.32]	0.36 [0.05;2.61]	0.64 [0.04;9.44]	0.25 [0.02;2.93]	0.53 [0.07;4.33]	0.28 [0.04;2.03]	LAM+CLOZ	-	-	-	-	-	-	-	-	-	-	-	-	-	-	-	-	-	-	-	-
0.13 [0.02;0.87]	0.11 [0.00;4.46]	3.82 [0.23;64.30]	0.90 [0.17;4.65]	0.53 [0.11;2.60]	0.94 [0.08;11.09]	0.37 [0.04;3.32]	0.78 [0.13;4.79]	0.40 [0.07;2.19]	1.45 [0.11;18.49]	LEV	-	-	-	-	-	-	-	-	-	-	-	-	-	-	-	-	-	-	-
0.06 [0.01;0.33]	0.05 [0.00;1.82]	1.67 [0.11;25.43]	0.39 [0.09;1.70]	0.23 [0.05;1.04]	0.41 [0.04;4.34]	0.16 [0.02;1.30]	0.34 [0.06;1.78]	0.18 [0.04;0.81]	0.64 [0.06;7.23]	0.44 [0.05;3.94]	MINOC+CLOZ	-	-	-	-	-	-	-	-	-	-	-	-	-	-	-	-	-	-
0.02 [0.00;0.40]	0.01 [0.00;1.24]	0.46 [0.01;21.51]	0.11 [0.00;2.36]	0.06 [0.00;1.41]	0.11 [0.00;4.12]	0.04 [0.00;1.36]	0.09 [0.00;2.25]	0.05 [0.00;1.09]	0.18 [0.00;6.69]	0.12 [0.00;3.96]	0.28 [0.01;8.34]	MIRT+CLOZ	-	-	-	-	-	-	-	-	-	-	-	-	-	-	-	-	-
0.04 [0.00;1.15]	0.04 [0.00;3.55]	1.30 [0.03;62.40]	0.31 [0.01;6.91]	0.18 [0.01;4.11]	0.32 [0.01;11.48]	0.12 [0.00;3.85]	0.26 [0.01;6.57]	0.14 [0.01;3.16]	0.49 [0.01;19.45]	0.34 [0.01;11.49]	0.77 [0.02;24.31]	2.81 [0.04;225.53]	MIRT+RISP	-	-	-	-	-	7.00 [0.32;154.87]	-	-	-	-	-	-	-	-	-	-
0.33 [0.14;0.83]	0.28 [0.01;7.67]	9.76 [0.96;99.47]	2.30 [1.62;3.25]	1.34 [0.85;2.11]	2.39 [0.37;15.47]	0.94 [0.21;4.26]	1.98 [0.84;4.67]	1.02 [0.59;1.79]	3.71 [0.52;26.68]	2.55 [0.48;13.49]	5.83 [1.30;26.20]	21.16 [0.95;469.83]	7.53 [0.33;172.01]	OLA	-	-	-	0.92 [0.22;3.92]	1.22 [0.63;2.34]	-	-	-	-	-	-	-	-	-	-
0.24 [0.04;1.35]	0.21 [0.01;7.56]	7.06 [0.47;106.33]	1.66 [0.39;7.05]	0.97 [0.22;4.27]	1.73 [0.18;17.00]	0.68 [0.09;5.16]	1.43 [0.28;7.41]	0.74 [0.16;3.32]	2.68 [0.24;30.19]	1.85 [0.21;16.36]	4.22 [0.54;32.94]	15.30 [0.51;459.79]	5.44 [0.18;162.38]	0.72 [0.17;3.12]	OXYT+RISP	-	-	-	1.29 [0.32;5.17]	-	-	-	-	-	-	-	-	-	-
0.67 [0.09;5.18]	0.57 [0.01;24.72]	19.55 [1.04;365.99]	4.60 [0.74;28.41]	2.68 [0.42;17.08]	4.79 [0.38;60.70]	1.88 [0.19;18.97]	3.97 [0.55;28.80]	2.05 [0.32;13.25]	7.43 [0.52;106.44]	5.11 [0.44;59.07]	11.68 [1.13;120.63]	42.37 [1.18;1517.89]	15.08 [0.42;536.26]	2.00 [0.32;12.54]	2.77 [0.29;26.52]	PALMIT+RISP	-	-	0.46 [0.08;2.75]	-	-	-	-	-	-	-	-	-	-
0.35 [0.05;2.44]	0.30 [0.01;12.28]	10.32 [0.60;177.96]	2.43 [0.45;13.10]	1.42 [0.27;7.34]	2.53 [0.21;30.80]	0.99 [0.11;9.25]	2.10 [0.33;13.43]	1.08 [0.19;6.17]	3.92 [0.30;51.32]	2.70 [0.27;26.80]	6.17 [0.66;57.39]	22.36 [0.67;749.38]	7.96 [0.23;275.23]	1.06 [0.19;5.83]	1.46 [0.16;13.39]	0.53 [0.04;6.28]	PBO	-	-	-	-	-	-	-	0.53 [0.11;2.60]	0.71 [0.14;3.66]	-	-	-
0.36 [0.12;1.07]	0.31 [0.01;8.72]	10.49 [0.99;111.04]	2.47 [1.43;4.27]	1.44 [0.85;2.43]	2.57 [0.38;17.51]	1.01 [0.23;4.51]	2.13 [0.82;5.53]	1.10 [0.55;2.21]	3.99 [0.53;29.98]	2.74 [0.51;14.78]	6.27 [1.32;29.85]	22.73 [0.99;519.54]	8.09 [0.34;190.74]	1.07 [0.58;1.98]	1.49 [0.32;6.85]	0.54 [0.08;3.54]	1.02 [0.18;5.71]	QUE	0.94 [0.30;2.99]	-	-	-	-	-	-	-	-	-	-
0.31 [0.11;0.85]	0.26 [0.01;7.35]	9.08 [0.89;93.05]	2.14 [1.45;3.14]	1.25 [0.75;2.07]	2.22 [0.36;13.62]	0.87 [0.20;3.82]	1.84 [0.77;4.41]	0.95 [0.54;1.67]	3.45 [0.48;24.99]	2.37 [0.44;12.73]	5.42 [1.20;24.59]	19.67 [0.88;438.84]	7.00 [0.32;154.87]	0.93 [0.59;1.45]	1.29 [0.32;5.17]	0.46 [0.08;2.75]	0.88 [0.16;4.92]	0.87 [0.46;1.63]	RISP	-	-	1.27 [0.64;2.53]	-	-	-	-	1.00 [0.23;4.32]	-	-
0.07 [0.02;0.28]	0.06 [0.00;1.91]	2.08 [0.17;25.07]	0.49 [0.19;1.29]	0.29 [0.10;0.80]	0.51 [0.06;4.12]	0.20 [0.03;1.18]	0.42 [0.12;1.46]	0.22 [0.07;0.63]	0.79 [0.09;6.91]	0.54 [0.08;3.65]	1.24 [0.22;7.16]	4.50 [0.18;113.72]	1.60 [0.06;42.03]	0.21 [0.08;0.59]	0.29 [0.05;1.68]	0.11 [0.01;0.84]	0.20 [0.03;1.41]	0.20 [0.07;0.60]	0.23 [0.08;0.65]	RISP+CLOZ	-	-	-	-	-	-	-	-	-
0.81 [0.06;11.74]	0.69 [0.01;43.50]	23.73 [0.80;703.95]	5.58 [0.46;67.67]	3.26 [0.27;38.81]	5.81 [0.26;129.60]	2.28 [0.13;41.25]	4.82 [0.35;65.83]	2.49 [0.20;31.33]	9.02 [0.38;212.92]	6.20 [0.32;118.54]	14.18 [0.79;255.49]	51.42 [0.98;2709.47]	18.30 [0.34;992.09]	2.43 [0.20;29.95]	3.36 [0.19;59.87]	1.21 [0.06;26.56]	2.30 [0.12;45.01]	2.26 [0.18;28.35]	2.61 [0.21;32.52]	11.42 [0.79;165.96]	SER+ZIP	-	-	-	-	-	-	0.58 [0.05;6.55]	-
0.40 [0.12;1.34]	0.34 [0.01;10.04]	11.56 [1.02;130.86]	2.72 [1.24;5.98]	1.59 [0.67;3.73]	2.83 [0.41;19.67]	1.11 [0.22;5.66]	2.35 [0.77;7.13]	1.21 [0.50;2.95]	4.39 [0.54;35.73]	3.02 [0.49;18.56]	6.91 [1.31;36.34]	25.05 [1.04;602.42]	8.91 [0.37;212.63]	1.18 [0.52;2.69]	1.64 [0.35;7.74]	0.59 [0.09;3.98]	1.12 [0.18;7.15]	1.10 [0.43;2.80]	1.27 [0.64;2.53]	5.56 [1.60;19.39]	0.49 [0.04;6.64]	SERTIND	-	-	-	-	-	-	-
0.12 [0.02;0.60]	0.10 [0.00;3.50]	3.36 [0.24;48.06]	0.79 [0.21;3.03]	0.46 [0.11;1.86]	0.82 [0.08;8.12]	0.32 [0.04;2.41]	0.68 [0.14;3.23]	0.35 [0.09;1.45]	1.28 [0.12;13.56]	0.88 [0.11;7.34]	2.01 [0.28;14.63]	7.29 [0.25;210.09]	2.59 [0.09;77.54]	0.34 [0.09;1.38]	0.48 [0.07;3.43]	0.17 [0.02;1.65]	0.33 [0.04;2.81]	0.32 [0.08;1.37]	0.37 [0.09;1.50]	1.62 [0.31;8.48]	0.14 [0.01;2.41]	0.29 [0.06;1.38]	SERTIND+CLOZ	-	-	-	-	-	-
0.33 [0.09;1.24]	0.28 [0.01;8.64]	9.56 [0.80;113.58]	2.25 [0.89;5.68]	1.31 [0.53;3.23]	2.34 [0.30;18.51]	0.92 [0.16;5.23]	1.94 [0.58;6.53]	1.00 [0.36;2.79]	3.63 [0.42;31.24]	2.50 [0.40;15.68]	5.71 [1.01;32.23]	20.71 [0.83;516.96]	7.37 [0.28;190.63]	0.98 [0.37;2.59]	1.35 [0.24;7.50]	0.49 [0.06;3.76]	0.93 [0.14;6.04]	0.91 [0.33;2.54]	1.05 [0.39;2.85]	4.60 [1.20;17.57]	0.40 [0.03;5.61]	0.83 [0.25;2.77]	2.84 [0.56;14.52]	SUL	-	-	-	-	-
0.19 [0.03;1.14]	0.16 [0.00;6.09]	5.46 [0.35;86.46]	1.29 [0.28;5.97]	0.75 [0.17;3.33]	1.34 [0.12;14.78]	0.53 [0.06;4.38]	1.11 [0.20;6.22]	0.57 [0.12;2.83]	2.08 [0.17;24.69]	1.43 [0.16;12.74]	3.26 [0.39;27.20]	11.84 [0.38;370.16]	4.21 [0.13;136.04]	0.56 [0.12;2.66]	0.77 [0.09;6.34]	0.28 [0.03;3.01]	0.53 [0.11;2.60]	0.52 [0.11;2.53]	0.60 [0.12;2.91]	2.63 [0.43;16.16]	0.23 [0.01;4.15]	0.47 [0.08;2.64]	1.62 [0.21;12.49]	0.57 [0.10;3.26]	THIO	1.33 [0.30;5.93]	-	-	-
0.25 [0.04;1.59]	0.21 [0.01;8.32]	7.28 [0.45;118.99]	1.71 [0.35;8.42]	1.00 [0.21;4.71]	1.78 [0.16;20.43]	0.70 [0.08;6.09]	1.48 [0.25;8.72]	0.76 [0.15;3.98]	2.77 [0.22;34.11]	1.90 [0.21;17.68]	4.35 [0.50;37.79]	15.79 [0.49;506.29]	5.62 [0.17;186.02]	0.75 [0.15;3.75]	1.03 [0.12;8.81]	0.37 [0.03;4.16]	0.71 [0.14;3.66]	0.69 [0.14;3.56]	0.80 [0.16;4.10]	3.51 [0.54;22.60]	0.31 [0.02;5.71]	0.63 [0.11;3.70]	2.16 [0.27;17.38]	0.76 [0.13;4.57]	1.33 [0.30;5.93]	TRIFLUO	-	-	-
0.31 [0.05;1.84]	0.26 [0.01;9.99]	9.08 [0.58;141.84]	2.14 [0.47;9.70]	1.25 [0.26;5.86]	2.22 [0.22;22.83]	0.87 [0.11;6.97]	1.84 [0.34;10.12]	0.95 [0.20;4.56]	3.45 [0.29;40.45]	2.37 [0.26;22.02]	5.42 [0.66;44.45]	19.67 [0.64;608.81]	7.00 [0.23;215.02]	0.93 [0.20;4.29]	1.29 [0.17;9.69]	0.46 [0.05;4.65]	0.88 [0.09;8.43]	0.87 [0.18;4.26]	1.00 [0.23;4.32]	4.37 [0.73;26.33]	0.38 [0.02;7.05]	0.79 [0.16;3.95]	2.70 [0.36;20.39]	0.95 [0.16;5.58]	1.66 [0.19;14.27]	1.25 [0.14;11.14]	VORT+RISP	-	-
0.47 [0.16;1.42]	0.40 [0.01;11.43]	13.69 [1.29;145.69]	3.22 [1.82;5.70]	1.88 [1.15;3.06]	3.35 [0.48;23.19]	1.32 [0.27;6.36]	2.78 [1.06;7.32]	1.44 [0.70;2.94]	5.20 [0.69;39.37]	3.58 [0.67;19.09]	8.18 [1.70;39.26]	29.67 [1.29;680.75]	10.56 [0.44;251.13]	1.40 [0.74;2.65]	1.94 [0.41;9.11]	0.70 [0.10;4.69]	1.33 [0.24;7.38]	1.31 [0.65;2.63]	1.51 [0.77;2.96]	6.59 [2.14;20.26]	0.58 [0.05;6.55]	1.18 [0.45;3.10]	4.07 [0.95;17.49]	1.43 [0.52;3.96]	2.51 [0.52;12.04]	1.88 [0.37;9.54]	1.51 [0.30;7.55]	ZIP	-
0.05 [0.00;1.39]	0.04 [0.00;4.09]	1.35 [0.03;72.62]	0.32 [0.01;8.26]	0.19 [0.01;4.92]	0.33 [0.01;14.03]	0.13 [0.00;4.67]	0.27 [0.01;7.82]	0.14 [0.01;3.80]	0.51 [0.01;22.77]	0.35 [0.01;13.56]	0.81 [0.02;28.68]	2.92 [0.03;259.12]	1.04 [0.01;94.77]	0.14 [0.01;3.66]	0.19 [0.01;6.75]	0.07 [0.00;2.88]	0.13 [0.00;5.13]	0.13 [0.00;3.50]	0.15 [0.01;3.96]	0.65 [0.02;19.45]	0.06 [0.00;3.45]	0.12 [0.00;3.34]	0.40 [0.01;13.61]	0.14 [0.00;4.18]	0.25 [0.01;9.06]	0.19 [0.00;6.96]	0.15 [0.00;5.40]	0.10 [0.00;2.69]	ZIP+CLOZ

Table S 11.11. League table: Sedation

AMI: amisulpride; ARI: aripiprazole; CLOZ: clozapine; CPZ: chlorpromazine; DESMOP: desmopressin; FLUPH: fluphenazine; FLUV: fluvoxamine; HAL: haloperidol; LAM: lamotrigine; LEV: levomepromazine; MINOC: minocycline; MIRT: mirtazapine; OLA: olanzapine; ONDAST: ondansetron; OXYT: oxytocin; PALMIT: palmitoylethanolamide; PBO: placebo; QUE: quetiapine; RISP: risperidone; SER: sertraline; SERTIND: sertindole; SUL: sulpiride; THIO: thioridazine; TRIFLUO: trifluoperazine; VORT: vortioxetine; ZIP: ziprasidone

11.12. League table: Weight gain

AMI	-	-	-	-	-	-	-	-	-	-	-	-0.45 [-1.09;0.18]	-	-	-	-	-	-	-	-	-	-
-0.66 [-1.61;0.30]	AMI+CLOZ	-	0.29 [-0.39;0.98]	-	-	-	-	-	-	-	-	-	-	-	-	-	-	-	-	-	-	-
0.11 [-0.65;0.88]	0.77 [-0.01;1.54]	ARI+CLOZ	-0.47 [-0.84;-0.11]	-	-	-	-	-	-	-	-	-	-	-	-	-	-	-	-	-	-	-
-0.36 [-1.03;0.31]	0.29 [-0.39;0.98]	-0.47 [-0.84;-0.11]	CLOZ	-	-	0.06 [-0.38;0.51]	0.94 [0.52;1.37]	-	0.50 [-0.48;1.47]	0.31 [-0.29;0.91]	-	-0.11 [-0.32;0.10]	-	-	-1.64 [-2.85;-0.43]	0.06 [-0.67;0.78]	0.31 [0.03;0.59]	-0.22 [-0.63;0.19]	-	0.00 [-0.70;0.70]	0.74 [0.20;1.29]	-0.17 [-0.85;0.50]
0.24 [-0.57;1.05]	0.90 [0.07;1.73]	0.13 [-0.47;0.73]	0.60 [0.13;1.08]	CPZ	-	-	-	-0.01 [-0.87;0.84]	-	-	-	-	-	-	-	-0.16 [-0.66;0.34]	-	-	-	-	0.21 [-0.28;0.70]	-
0.34 [-0.69;1.37]	1.00 [-0.06;2.05]	0.23 [-0.66;1.11]	0.70 [-0.10;1.51]	0.10 [-0.78;0.98]	FLUPH	-	-	-	-	-	-	-	-	-	-	-0.16 [-1.06;0.74]	-0.45 [-1.33;0.44]	-	-	-	-	-
-0.30 [-1.10;0.50]	0.36 [-0.45;1.17]	-0.41 [-0.98;0.17]	0.06 [-0.38;0.51]	-0.54 [-1.19;0.11]	-0.64 [-1.56;0.28]	FLUV+CLOZ	-	-	-	-	-	-	-	-	-	-	-	-	-	-	-	-
0.31 [-0.40;1.02]	0.97 [0.23;1.71]	0.20 [-0.27;0.67]	0.67 [0.38;0.97]	0.07 [-0.43;0.58]	-0.03 [-0.85;0.79]	0.61 [0.08;1.14]	HAL	-	-	-	-	-0.85 [-1.32;-0.38]	-	-	-	-0.12 [-0.61;0.37]	-0.57 [-1.05;-0.09]	-	-	-	-	-
0.23 [-0.95;1.40]	0.88 [-0.31;2.08]	0.11 [-0.93;1.16]	0.59 [-0.39;1.57]	-0.01 [-0.87;0.84]	-0.11 [-1.34;1.12]	0.52 [-0.55;1.60]	-0.08 [-1.08;0.91]	LEV	-	-	-	-	-	-	-	-	-	-	-	-	-	-
0.13 [-1.04;1.31]	0.79 [-0.40;1.98]	0.02 [-1.02;1.06]	0.50 [-0.48;1.47]	-0.11 [-1.19;0.97]	-0.21 [-1.47;1.06]	0.43 [-0.64;1.50]	-0.18 [-1.19;0.84]	-0.09 [-1.47;1.29]	MEM+CLOZ	-	-	-	-	-	-	-	-	-	-	-	-	-
-0.05 [-0.95;0.85]	0.61 [-0.30;1.51]	-0.16 [-0.87;0.54]	0.31 [-0.29;0.91]	-0.29 [-1.06;0.47]	-0.39 [-1.40;0.61]	0.25 [-0.50;0.99]	-0.36 [-1.03;0.31]	-0.28 [-1.43;0.87]	-0.19 [-1.33;0.96]	MET+CLOZ	-	-	-	-	-	-	-	-	-	-	-	-
-0.34 [-1.55;0.86]	0.31 [-0.91;1.54]	-0.45 [-1.54;0.63]	0.02 [-1.00;1.04]	-0.58 [-1.69;0.53]	-0.68 [-1.95;0.58]	-0.05 [-1.15;1.06]	-0.65 [-1.69;0.38]	-0.57 [-1.97;0.83]	-0.48 [-1.88;0.93]	-0.29 [-1.47;0.89]	MIRT+RISP	-	-	-	-	-	0.30 [-0.68;1.29]	-	-	-	-	-
-0.45 [-1.09;0.18]	0.20 [-0.51;0.91]	-0.57 [-0.98;-0.15]	-0.09 [-0.29;0.10]	-0.70 [-1.19;-0.20]	-0.80 [-1.61;0.02]	-0.16 [-0.64;0.33]	-0.77 [-1.07;-0.46]	-0.68 [-1.67;0.30]	-0.59 [-1.58;0.40]	-0.40 [-1.04;0.23]	-0.11 [-1.13;0.91]	OLA	0.23 [-0.65;1.10]	0.35 [-0.26;0.96]	-	0.55 [-0.01;1.11]	0.51 [0.08;0.93]	-	-	-	-	-
-0.17 [-1.01;0.67]	0.49 [-0.38;1.36]	-0.28 [-0.94;0.38]	0.20 [-0.35;0.74]	-0.41 [-1.11;0.30]	-0.51 [-1.45;0.43]	0.13 [-0.57;0.83]	-0.48 [-1.07;0.11]	-0.39 [-1.50;0.72]	-0.30 [-1.42;0.81]	-0.11 [-0.93;0.70]	0.18 [-0.94;1.29]	0.29 [-0.26;0.83]	OLA+RISP	-	-	-	0.09 [-0.54;0.73]	-	-	-	-	-
-0.10 [-0.99;0.78]	0.55 [-0.38;1.49]	-0.22 [-0.95;0.52]	0.26 [-0.38;0.90]	-0.35 [-1.13;0.44]	-0.45 [-1.46;0.57]	0.19 [-0.58;0.97]	-0.42 [-1.10;0.26]	-0.33 [-1.49;0.83]	-0.24 [-1.40;0.92]	-0.05 [-0.93;0.82]	0.24 [-0.95;1.43]	0.35 [-0.26;0.96]	0.06 [-0.75;0.88]	PALIP	-	-	-	-	-	-	-	-
-2.00 [-3.39;-0.62]	-1.35 [-2.74;0.05]	-2.11 [-3.38;-0.85]	-1.64 [-2.85;-0.43]	-2.24 [-3.54;-0.94]	-2.34 [-3.80;-0.89]	-1.70 [-3.00;-0.41]	-2.31 [-3.56;-1.07]	-2.23 [-3.79;-0.67]	-2.14 [-3.69;-0.58]	-1.95 [-3.30;-0.60]	-1.66 [-3.24;-0.08]	-1.55 [-2.78;-0.32]	-1.84 [-3.17;-0.50]	-1.90 [-3.27;-0.53]	PHENYLPROP+	-	-	-	-	-	-	-
0.11 [-0.62;0.84]	0.77 [0.01;1.53]	0.00 [-0.50;0.50]	0.48 [0.13;0.82]	-0.13 [-0.55;0.30]	-0.23 [-1.02;0.57]	0.41 [-0.15;0.97]	-0.20 [-0.55;0.15]	-0.11 [-1.07;0.84]	-0.02 [-1.05;1.01]	0.17 [-0.53;0.86]	0.46 [-0.60;1.51]	0.57 [0.22;0.92]	0.28 [-0.34;0.90]	0.22 [-0.48;0.92]	2.12 [0.86;3.38]	QUE	-0.14 [-0.74;0.47]	-	-	-	-	-
-0.04 [-0.73;0.66]	0.62 [-0.11;1.34]	-0.15 [-0.59;0.29]	0.32 [0.08;0.57]	-0.28 [-0.78;0.23]	-0.38 [-1.17;0.41]	0.26 [-0.25;0.76]	-0.35 [-0.67;-0.03]	-0.27 [-1.26;0.73]	-0.17 [-1.17;0.83]	0.01 [-0.64;0.66]	0.30 [-0.68;1.29]	0.42 [0.15;0.69]	0.13 [-0.39;0.65]	0.07 [-0.60;0.73]	1.96 [0.73;3.20]	-0.15 [-0.52;0.22]	RISP	-	-0.15 [-0.65;0.36]	-	-	-
-0.58 [-1.37;0.20]	0.07 [-0.72;0.87]	-0.69 [-1.25;-0.14]	-0.22 [-0.63;0.19]	-0.82 [-1.45;-0.19]	-0.92 [-1.83;-0.02]	-0.29 [-0.89;0.32]	-0.89 [-1.40;-0.39]	-0.81 [-1.87;0.25]	-0.72 [-1.77;0.34]	-0.53 [-1.26;0.20]	-0.24 [-1.34;0.86]	-0.13 [-0.59;0.33]	-0.42 [-1.10;0.27]	-0.48 [-1.24;0.28]	1.42 [0.14;2.70]	-0.70 [-1.23;-0.16]	-0.54 [-1.02;-0.06]	RISP+CLOZ	-	-	-	-
-0.18 [-1.04;0.67]	0.47 [-0.41;1.36]	-0.30 [-0.97;0.38]	0.18 [-0.38;0.74]	-0.42 [-1.14;0.29]	-0.52 [-1.47;0.42]	0.11 [-0.60;0.83]	-0.50 [-1.10;0.10]	-0.41 [-1.53;0.70]	-0.32 [-1.44;0.80]	-0.13 [-0.96;0.69]	0.16 [-0.95;1.27]	0.27 [-0.30;0.84]	-0.02 [-0.75;0.71]	-0.08 [-0.92;0.76]	1.82 [0.48;3.15]	-0.30 [-0.92;0.33]	-0.15 [-0.65;0.36]	0.40 [-0.30;1.10]	SERTIND	-	-	-
-0.36 [-1.33;0.61]	0.29 [-0.68;1.27]	-0.47 [-1.27;0.32]	0.00 [-0.70;0.70]	-0.60 [-1.45;0.25]	-0.70 [-1.77;0.37]	-0.06 [-0.90;0.77]	-0.67 [-1.44;0.09]	-0.59 [-1.80;0.62]	-0.50 [-1.70;0.70]	-0.31 [-1.24;0.62]	-0.02 [-1.26;1.22]	0.09 [-0.64;0.82]	-0.20 [-1.09;0.70]	-0.26 [-1.21;0.69]	1.64 [0.24;3.04]	-0.48 [-1.26;0.31]	-0.32 [-1.07;0.42]	0.22 [-0.60;1.04]	-0.18 [-1.08;0.72]	SERTIND+CLO	-	-
0.42 [-0.38;1.22]	1.08 [0.26;1.89]	0.31 [-0.27;0.89]	0.78 [0.33;1.23]	0.18 [-0.24;0.60]	0.08 [-0.82;0.97]	0.72 [0.09;1.35]	0.11 [-0.40;0.62]	0.19 [-0.76;1.15]	0.29 [-0.78;1.36]	0.47 [-0.28;1.22]	0.76 [-0.34;1.87]	0.88 [0.40;1.35]	0.59 [-0.11;1.29]	0.53 [-0.25;1.30]	2.42 [1.13;3.72]	0.31 [-0.17;0.79]	0.46 [-0.04;0.96]	1.00 [0.39;1.61]	0.60 [-0.10;1.31]	0.78 [-0.05;1.62]	ZIP	-
-0.54 [-1.49;0.42]	0.12 [-0.84;1.08]	-0.65 [-1.42;0.12]	-0.17 [-0.85;0.50]	-0.78 [-1.60;0.05]	-0.88 [-1.93;0.18]	-0.24 [-1.05;0.57]	-0.85 [-1.59;-0.11]	-0.76 [-1.95;0.43]	-0.67 [-1.85;0.52]	-0.48 [-1.39;0.42]	-0.19 [-1.41;1.03]	-0.08 [-0.79;0.62]	-0.37 [-1.24;0.50]	-0.43 [-1.36;0.50]	1.47 [0.08;2.86]	-0.65 [-1.41;0.11]	-0.50 [-1.22;0.22]	0.05 [-0.75;0.84]	-0.35 [-1.23;0.53]	-0.17 [-1.15;0.80]	-0.96 [-1.77;-0.14]	ZOT

Table S 11.12. League table: Weight gain

AMI: amisulpride; ARI: aripiprazole; CLOZ: clozapine; CPZ: chlorpromazine; FLUPH: fluphenazine; FLUV: fluvoxamine; HAL: haloperidol; LEV: levomepromazine; MEM: memantine; MET: metformin; MIRT: mirtazapine; OLA: olanzapine; PALMIT: palmitoylethanolamide; PHENYLPROP: phenylpropanolamine; QUE: quetiapine; RISP: risperidone; SERTIND: sertindole; ZIP: ziprasidone; ZOT: zotepine

11.13. League table: Prolactin elevation

AMI+CLOZ	-	0.93 [0.25;1.60]	-	-	-	-	-	-	-	-	-	-	-	-
1.89 [0.87;2.90]	ARI+CLOZ	-0.96 [-1.72;-0.20]	-	-	-	-	-	-	-	-	-	-	-	-
0.93 [0.25;1.60]	-0.96 [-1.72;-0.20]	CLOZ	-	-	-	-0.36 [-0.80;0.07]	-	0.39 [-0.40;1.18]	-1.69 [-2.33;-1.05]	-1.73 [-2.29;-1.16]	-3.15 [-4.39;-1.91]	-0.03 [-0.70;0.65]	-	-1.18 [-1.96;-0.40]
0.61 [-0.31;1.53]	-1.27 [-2.25;-0.29]	-0.31 [-0.94;0.31]	CPZ	-	-	-	-	0.35 [-0.24;0.95]	-	-	-	0.28 [-0.31;0.86]	-	-
0.43 [-0.81;1.68]	-1.45 [-2.74;-0.16]	-0.49 [-1.53;0.55]	-0.18 [-1.27;0.92]	FLUPH	-	-	-	0.74 [-0.44;1.92]	-1.20 [-2.27;-0.13]	-	-	-	-	-
0.24 [-0.73;1.20]	-1.65 [-2.67;-0.63]	-0.69 [-1.37;0.00]	-0.37 [-1.09;0.34]	-0.20 [-1.27;0.87]	HAL	-	-	0.45 [-0.15;1.06]	-0.29 [-1.16;0.57]	-	-	-	-	-
0.55 [-0.24;1.35]	-1.33 [-2.20;-0.47]	-0.37 [-0.79;0.05]	-0.06 [-0.76;0.64]	0.12 [-0.95;1.19]	0.32 [-0.41;1.05]	OLA	-0.98 [-1.94;-0.01]	0.66 [-0.21;1.53]	-1.41 [-2.29;-0.53]	-	-	-	-	-
-0.41 [-1.44;0.62]	-2.30 [-3.38;-1.21]	-1.34 [-2.12;-0.56]	-1.02 [-1.95;-0.09]	-0.85 [-2.04;0.35]	-0.65 [-1.57;0.27]	-0.97 [-1.71;-0.22]	OLA+RISP	-	-0.21 [-1.18;0.76]	-	-	-	-	-
0.96 [0.09;1.83]	-0.93 [-1.86;0.01]	0.03 [-0.51;0.58]	0.35 [-0.17;0.87]	0.52 [-0.47;1.52]	0.72 [0.20;1.25]	0.40 [-0.20;1.01]	1.37 [0.52;2.22]	QUE	-1.72 [-2.45;-0.98]	-	-	-	-	-
-0.61 [-1.47;0.25]	-2.50 [-3.42;-1.58]	-1.54 [-2.06;-1.01]	-1.22 [-1.91;-0.53]	-1.05 [-2.01;-0.08]	-0.85 [-1.47;-0.23]	-1.17 [-1.74;-0.59]	-0.20 [-0.94;0.54]	-1.57 [-2.12;-1.02]	RISP	-	-	-	-	-
-0.80 [-1.68;0.08]	-2.69 [-3.63;-1.74]	-1.73 [-2.29;-1.16]	-1.41 [-2.25;-0.57]	-1.23 [-2.42;-0.05]	-1.04 [-1.92;-0.15]	-1.35 [-2.05;-0.66]	-0.39 [-1.35;0.57]	-1.76 [-2.54;-0.98]	-0.19 [-0.96;0.58]	RISP+CLOZ	-	-	5.10 [3.29;6.90]	-
-2.23 [-3.64;-0.81]	-4.11 [-5.56;-2.66]	-3.15 [-4.39;-1.91]	-2.84 [-4.22;-1.45]	-2.66 [-4.28;-1.04]	-2.46 [-3.88;-1.05]	-2.78 [-4.09;-1.47]	-1.82 [-3.28;-0.35]	-3.19 [-4.54;-1.83]	-1.62 [-2.96;-0.27]	-1.43 [-2.79;-0.07]	SUL+CLOZ	-	-	-
0.89 [0.01;1.78]	-0.99 [-1.94;-0.05]	-0.03 [-0.60;0.53]	0.28 [-0.24;0.80]	0.46 [-0.66;1.58]	0.66 [-0.12;1.43]	0.34 [-0.34;1.01]	1.30 [0.38;2.23]	-0.07 [-0.69;0.55]	1.50 [0.80;2.20]	1.69 [0.89;2.49]	3.12 [1.76;4.48]	ZIP	-	-
4.30 [2.29;6.31]	2.41 [0.38;4.45]	3.37 [1.48;5.26]	3.69 [1.70;5.68]	3.86 [1.70;6.02]	4.06 [2.05;6.07]	3.74 [1.81;5.68]	4.71 [2.66;6.76]	3.34 [1.37;5.31]	4.91 [2.95;6.87]	5.10 [3.29;6.90]	6.53 [4.27;8.79]	3.41 [1.43;5.38]	ZIP+CLOZ	-
-0.26 [-1.29;0.77]	-2.14 [-3.23;-1.06]	-1.18 [-1.96;-0.40]	-0.87 [-1.87;0.13]	-0.69 [-1.99;0.61]	-0.49 [-1.53;0.54]	-0.81 [-1.70;0.07]	0.15 [-0.95;1.26]	-1.22 [-2.17;-0.27]	0.35 [-0.59;1.29]	0.54 [-0.42;1.50]	1.97 [0.51;3.43]	-1.15 [-2.11;-0.19]	-4.56 [-6.60;-2.51]	ZOT

Table S 11.13. League table: Prolactin elevation

AMI: amisulpride; ARI: aripiprazole; CLOZ: clozapine; CPZ: chlorpromazine; FLUPH: fluphenazine; FLUV: fluvoxamine; HAL: haloperidol; LEV: levomepromazine; OLA: olanzapine; QUE: quetiapine; RISP: risperidone; SUL: sulpiride; ZIP: ziprasidone; ZOT: zotepine

11.14. League table: QTc prolongation

AMI+CLOZ	-	-0.53<br>[-2.00;0.94]	-	-	-	-	-	-	-	-	-
-0.68<br>[-2.72;1.36]	ARI+CLOZ	0.15<br>[-1.27;1.57]	-	-	-	-	-	-	-	-	-
-0.53<br>[-2.00;0.94]	0.15<br>[-1.27;1.57]	CLOZ	-	-	-0.31<br>[-1.75;1.12]	-0.39<br>[-1.43;0.66]	-	0.63<br>[-0.88;2.14]	-	0.13<br>[-1.31;1.56]	-0.23<br>[-1.60;1.13]
-0.86<br>[-3.27;1.55]	-0.18<br>[-2.56;2.20]	-0.33<br>[-2.24;1.59]	CPZ	0.33<br>[-1.23;1.89]	-	-	0.63<br>[-0.72;1.97]	-	-	-	0.10<br>[-1.25;1.44]
-0.53<br>[-3.40;2.34]	0.15<br>[-2.70;2.99]	-0.00<br>[-2.47;2.47]	0.33<br>[-1.23;1.89]	LEV	-	-	-	-	-	-	-
-0.84<br>[-2.90;1.21]	-0.16<br>[-2.18;1.85]	-0.31<br>[-1.75;1.12]	0.01<br>[-2.38;2.41]	-0.31<br>[-3.17;2.54]	MINOC+CLOZ	-	-	-	-	-	-
-0.92<br>[-2.71;0.88]	-0.24<br>[-1.99;1.52]	-0.39<br>[-1.43;0.66]	-0.06<br>[-2.24;2.12]	-0.38<br>[-3.06;2.29]	-0.07<br>[-1.84;1.70]	PIMOZ+CLOZ	-	-	-	-	-
-0.23<br>[-2.99;2.53]	0.45<br>[-2.29;3.18]	0.30<br>[-2.04;2.64]	0.63<br>[-0.72;1.97]	0.30<br>[-1.76;2.36]	0.61<br>[-2.13;3.36]	0.68<br>[-1.88;3.25]	QUE	-	-	-	-
0.78<br>[-1.15;2.72]	1.46<br>[-0.44;3.36]	1.31<br>[0.04;2.58]	1.64<br>[-0.66;3.94]	1.31<br>[-1.46;4.09]	1.63<br>[-0.29;3.54]	1.70<br>[0.06;3.34]	1.02<br>[-1.65;3.68]	RISP+CLOZ	-	-	-
0.20<br>[-2.24;2.63]	0.88<br>[-1.53;3.28]	0.73<br>[-1.22;2.67]	1.06<br>[-0.87;2.99]	0.73<br>[-1.75;3.21]	1.04<br>[-1.37;3.46]	1.11<br>[-1.09;3.32]	0.43<br>[-1.92;2.78]	-0.58<br>[-2.91;1.74]	SER+ZIP	-	-0.96<br>[-2.35;0.42]
-0.40<br>[-2.45;1.65]	0.28<br>[-1.74;2.29]	0.13<br>[-1.31;1.56]	0.46<br>[-1.94;2.85]	0.13<br>[-2.73;2.98]	0.44<br>[-1.59;2.47]	0.51<br>[-1.26;2.29]	-0.17<br>[-2.91;2.58]	-1.18<br>[-3.10;0.73]	-0.60<br>[-3.02;1.82]	SERTIND+CLOZ	-
-0.76<br>[-2.77;1.24]	-0.08<br>[-2.05;1.88]	-0.23<br>[-1.60;1.13]	0.10<br>[-1.25;1.44]	-0.23<br>[-2.29;1.83]	0.08<br>[-1.90;2.06]	0.15<br>[-1.56;1.87]	-0.53<br>[-2.43;1.37]	-1.55<br>[-3.41;0.32]	-0.96<br>[-2.35;0.42]	-0.36<br>[-2.34;1.62]	ZIP
-1.31<br>[-3.23;0.61]	-0.63<br>[-2.51;1.25]	-0.78<br>[-2.02;0.46]	-0.45<br>[-2.73;1.83]	-0.78<br>[-3.54;1.99]	-0.46<br>[-2.36;1.43]	-0.39<br>[-2.01;1.23]	-1.08<br>[-3.73;1.57]	-2.09<br>[-3.46;-0.72]	-1.51<br>[-3.81;0.80]	-0.91<br>[-2.80;0.99]	-0.55<br>[-2.39;1.30]

Table S 11.14. League table: QTc prolongation

AMI: amisulpride; ARI: aripiprazole; CLOZ: clozapine; CPZ: chlorpromazine; LEV: levomepromazine; MINOC: minocycline; PIMOZ: pimozide; QUE: quetiapine; RISP: risperidone; SER: sertraline; ZIP: ziprasidone

11.15. League table: Quality of life

AMI+CLOZ	-	0.22 [-0.32;0.75]	-	-	-	-	-
-0.32 [-1.11;0.48]	BEZOAET+CLO	0.54 [-0.05;1.12]	-	-	-	-	-
0.22 [-0.32;0.75]	0.54 [-0.05;1.12]	CLOZ	0.24 [-0.01;0.49]	-0.46 [-1.04;0.12]	0.05 [-0.55;0.66]	-0.24 [-0.53;0.05]	-0.09 [-0.82;0.63
0.38 [-0.20;0.97]	0.70 [0.07;1.33]	0.16 [-0.07;0.40]	HAL	-	-	-0.02 [-0.52;0.49]	-
-0.24 [-1.03;0.55]	0.08 [-0.75;0.90]	-0.46 [-1.04;0.12]	-0.62 [-1.25;0.00]	MEM+CLOZ	-	-	-
0.27 [-0.54;1.08]	0.59 [-0.26;1.44]	0.05 [-0.55;0.66]	-0.11 [-0.76;0.54]	0.51 [-0.33;1.35]	Nacetyl+CLOZ	-	-
0.08 [-0.52;0.67]	0.39 [-0.25;1.04]	-0.14 [-0.40;0.12]	-0.31 [-0.61;0.00]	0.32 [-0.32;0.95]	-0.20 [-0.86;0.47]	OLA	-
0.13 [-0.78;1.03]	0.44 [-0.49;1.38]	-0.09 [-0.82;0.63]	-0.26 [-1.02;0.51]	0.37 [-0.56;1.29]	-0.15 [-1.09;0.80]	0.05 [-0.72;0.82]	RISP+CLO

Table S 11.15. League table: Quality of life

AMI: amisulpride; BEZOAET: benzoate sodium; CLOZ: clozapine; HAL: haloperidol; MEM: memantine; Nacetyl: N-acetylcysteine; OLA: olanzapine; RISP: risperidone

11.16. League table: Social functioning

AMI+CLOZ	-	-	0.03 [-1.20;1.26]	-	-	-	-	-	-	-	-	-	-	-	-	-	-	-	-
-0.02 [-1.94;1.90]	ARI+CLOZ	-	0.05 [-1.43;1.53]	-	-	-	-	-	-	-	-	-	-	-	-	-	-	-	-
-0.28 [-2.27;1.70]	-0.26 [-2.41;1.89]	BEZOAET+CLO	0.32 [-1.25;1.88]	-	-	-	-	-	-	-	-	-	-	-	-	-	-	-	-
0.03 [-1.20;1.26]	0.05 [-1.43;1.53]	0.32 [-1.25;1.88]	CLOZ	-0.21 [-1.74;1.32]	-0.76 [-2.41;0.89]	1.55 [-0.20;3.30]	0.38 [-1.16;1.92]	-0.38 [-2.22;1.46]	0.55 [-0.58;1.67]	0.46 [-1.41;2.32]	-	0.02 [-1.53;1.57]	-0.85 [-2.58;0.88]	0.83 [-0.81;2.46]	-	-	0.00 [-1.56;1.56]	-0.09 [-1.58;1.40]	-
-0.18 [-2.14;1.78]	-0.16 [-2.28;1.97]	0.11 [-2.08;2.29]	-0.21 [-1.74;1.32]	FLUV+CLOZ	-	-	-	-	-	-	-	-	-	-	-	-	-	-	-
-0.73 [-2.79;1.32]	-0.71 [-2.93;1.50]	-0.45 [-2.72;1.82]	-0.76 [-2.41;0.89]	-0.56 [-2.81;1.69]	GLY+CLOZ	-	-	-	-	-	-	-	-	-	-	-	-	-	-
1.00 [-0.77;2.76]	1.02 [-0.93;2.97]	1.28 [-0.73;3.29]	0.97 [-0.31;2.24]	1.17 [-0.82;3.16]	1.73 [-0.35;3.81]	HAL	-	-	0.00 [-1.54;1.54]	-	-	-	-	-	-	-	-	-	-
0.41 [-1.56;2.38]	0.43 [-1.71;2.57]	0.69 [-1.50;2.89]	0.38 [-1.16;1.92]	0.59 [-1.58;2.76]	1.14 [-1.12;3.40]	-0.59 [-2.59;1.41]	LAM+CLOZ	-	-	-	-	-	-	-	-	-	-	-	-
-0.35 [-2.56;1.87]	-0.33 [-2.69;2.03]	-0.06 [-2.47;2.35]	-0.38 [-2.22;1.46]	-0.17 [-2.56;2.22]	0.39 [-2.08;2.86]	-1.34 [-3.58;0.89]	-0.76 [-3.16;1.64]	MET+CLOZ	-	-	-	-	-	-	-	-	-	-	-
0.55 [-1.00;2.09]	0.57 [-1.19;2.32]	0.83 [-1.00;2.65]	0.51 [-0.43;1.46]	0.72 [-1.08;2.52]	1.28 [-0.62;3.18]	-0.45 [-1.68;0.77]	0.14 [-1.67;1.94]	0.89 [-1.18;2.96]	OLA	-	-0.45 [-2.09;1.20]	-	-	-	1.26 [-0.37;2.89]	-	-	-	-
0.49 [-1.74;2.72]	0.51 [-1.87;2.88]	0.77 [-1.66;3.20]	0.46 [-1.41;2.32]	0.66 [-1.74;3.07]	1.22 [-1.27;3.71]	-0.51 [-2.76;1.74]	0.08 [-2.34;2.49]	0.83 [-1.78;3.45]	-0.06 [-2.14;2.03]	OLA+CLOZ	-	-	-	-	-	-	-	-	-
-0.49 [-2.36;1.39]	-0.47 [-2.52;1.58]	-0.20 [-2.31;1.91]	-0.52 [-1.94;0.90]	-0.31 [-2.40;1.78]	0.25 [-1.93;2.42]	-1.48 [-3.22;0.25]	-0.90 [-2.99;1.20]	-0.14 [-2.46;2.18]	-1.03 [-2.37;0.31]	-0.98 [-3.31;1.36]	OLA+RISP	-	0.62 [-0.56;1.79]	-	-	-	-	-	-
0.05 [-1.92;2.03]	0.07 [-2.07;2.22]	0.34 [-1.86;2.54]	0.02 [-1.53;1.57]	0.23 [-1.95;2.41]	0.79 [-1.48;3.05]	-0.94 [-2.95;1.06]	-0.36 [-2.54;1.83]	0.40 [-2.01;2.80]	-0.49 [-2.31;1.32]	-0.43 [-2.86;1.99]	0.54 [-1.56;2.64]	PIMOZ+CLOZ	-	-	-	-	-	-	-
-0.17 [-2.01;1.67]	-0.15 [-2.17;1.87]	0.11 [-1.97;2.19]	-0.20 [-1.58;1.17]	0.01 [-2.05;2.06]	0.56 [-1.58;2.71]	-1.17 [-2.92;0.59]	-0.58 [-2.64;1.49]	0.18 [-2.12;2.47]	-0.71 [-2.14;0.71]	-0.66 [-2.97;1.66]	0.32 [-0.75;1.39]	-0.22 [-2.29;1.85]	RISP	-	-	-	-	-	-
0.86 [-1.18;2.90]	0.88 [-1.32;3.08]	1.14 [-1.12;3.40]	0.83 [-0.81;2.46]	1.04 [-1.20;3.28]	1.59 [-0.73;3.92]	-0.14 [-2.21;1.93]	0.45 [-1.80;2.70]	1.20 [-1.26;3.66]	0.31 [-1.57;2.20]	0.37 [-2.10;2.85]	1.35 [-0.82;3.51]	0.81 [-1.45;3.06]	1.03 [-1.11;3.16]	RISP+CLOZ	-	-	-	-	0.00 [-1.68;1.68]
1.81 [-0.44;4.06]	1.83 [-0.57;4.22]	2.09 [-0.35;4.54]	1.78 [-0.11;3.66]	1.99 [-0.44;4.41]	2.54 [0.04;5.05]	0.81 [-1.23;2.85]	1.40 [-1.04;3.83]	2.15 [-0.48;4.79]	1.26 [-0.37;2.89]	1.32 [-1.33;3.97]	2.30 [0.19;4.41]	1.75 [-0.68;4.19]	1.98 [-0.18;4.14]	0.95 [-1.54;3.44]	SER+OLA	-	-	-	-
-0.48 [-2.93;1.96]	-0.46 [-3.05;2.12]	-0.20 [-2.83;2.43]	-0.52 [-2.63;1.60]	-0.31 [-2.92;2.31]	0.25 [-2.44;2.93]	-1.48 [-3.95;0.99]	-0.89 [-3.51;1.73]	-0.14 [-2.94;2.67]	-1.03 [-3.35;1.29]	-0.97 [-3.79;1.85]	0.00 [-2.54;2.55]	-0.54 [-3.16;2.09]	-0.31 [-2.84;2.21]	-1.34 [-4.02;1.33]	-2.29 [-5.13;0.54]	SER+ZIP	-	0.43 [-1.08;1.93]	-
0.03 [-1.95;2.01]	0.05 [-2.09;2.20]	0.32 [-1.89;2.52]	0.00 [-1.56;1.56]	0.21 [-1.97;2.39]	0.76 [-1.50;3.03]	-0.97 [-2.97;1.04]	-0.38 [-2.57;1.81]	0.38 [-2.03;2.79]	-0.51 [-2.33;1.31]	-0.46 [-2.88;1.97]	0.52 [-1.59;2.62]	-0.02 [-2.22;2.17]	0.20 [-1.87;2.28]	-0.83 [-3.08;1.43]	-1.78 [-4.22;0.67]	0.52 [-2.11;3.14]	SERTIND+CLO	-	-
-0.06 [-1.99;1.87]	-0.04 [-2.14;2.06]	0.23 [-1.93;2.38]	-0.09 [-1.58;1.40]	0.12 [-2.01;2.26]	0.68 [-1.55;2.90]	-1.05 [-3.01;0.90]	-0.47 [-2.61;1.68]	0.29 [-2.08;2.65]	-0.60 [-2.37;1.16]	-0.54 [-2.93;1.84]	0.43 [-1.63;2.49]	-0.11 [-2.26;2.04]	0.11 [-1.91;2.14]	-0.92 [-3.13;1.29]	-1.87 [-4.27;0.54]	0.43 [-1.08;1.93]	-0.09 [-2.24;2.07]	ZIP	-
0.86 [-1.78;3.50]	0.88 [-1.89;3.65]	1.14 [-1.67;3.96]	0.83 [-1.51;3.17]	1.04 [-1.76;3.83]	1.59 [-1.27;4.46]	-0.14 [-2.80;2.53]	0.45 [-2.35;3.25]	1.20 [-1.77;4.18]	0.31 [-2.21;2.84]	0.37 [-2.62;3.36]	1.35 [-1.39;4.08]	0.81 [-2.00;3.61]	1.03 [-1.68;3.74]	0.00 [-1.68;1.68]	-0.95 [-3.95;2.06]	1.34 [-1.81;4.50]	0.83 [-1.98;3.64]	0.92 [-1.86;3.69]	ZIP+CLOZ

Table S 11.16. League table: Social functioning

AMI: amisulpride; ARI: aripiprazole; BEZOAET: benzoate sodium; CLOZ: clozapine; FLUV: fluvoxamine; GLY: glycine; HAL: haloperidol; LAM: lamotrigine; MET: metformin; OLA: olanzapine; PIMOZ: pimozide; RISP: risperidone; SER: sertraline; ZIP: ziprasidone

Appendix 12. Other models for dichotomous outcomes

A key issue in rare event (network) meta-analysis is that random-effects models often fail to estimate the heterogeneity variance due to increased uncertainty around the trials' effect sizes and it is common not to find heterogeneity at all. In such a case random. In such cases random-effects models reduce to their common-effect counterparts. The various common-effect models for dichotomous (i.e., binary) outcomes that we used are Mantel-Haenszel (MH), non-central hypergeometric distribution with Breslow approximation, and logistic regression with Firth's correction [174,175].

12.1. Other models for dichotomous outcomes: Response rates

Treatment	Firth results	Common MH With Correction	Common NCH With Correction	Common MH Without Correction	Common NCH Without Correction
**AMI**	0.76 (0.24, 2.37)	0.77 (0.31, 1.87)	0.85 (0.41, 1.74)	0.76 (0.31, 1.86)	0.85 (0.41, 1.74)
**AMI+CLOZ**	2.46 (0.88, 6.86)	2.54 (1.12, 5.73)	1.96 (0.98, 3.92)	2.54 (1.12, 5.73)	1.96 (0.98, 3.92)
**AMI+OLA**	2.01 (0.22, 18.13)	2.21 (0.39, 12.67)	1.26 (0.47, 3.37)	2.19 (0.38, 12.56)	1.25 (0.47, 3.35)
**ARI+CLOZ**	1.31 (0.37, 4.63)	1.34 (0.49, 3.67)	1.31 (0.5, 3.44)	1.34 (0.49, 3.67)	1.31 (0.5, 3.44)
**CPZ**	0.47 (0.29, 0.75)	0.6 (0.39, 0.92)	0.6 (0.45, 0.81)	0.62 (0.41, 0.95)	0.61 (0.46, 0.83)
**DONEP+CLOZ**	3.18 (0.16, 63.65)	4.2 (0.33, 53.12)	3 (0.31, 28.82)	4.2 (0.33, 53.12)	2.99 (0.31, 28.65)
**FLUPH**	0.32 (0.08, 1.33)	0.32 (0.1, 1.02)	0.42 (0.14, 1.22)	0.32 (0.1, 1.03)	0.42 (0.14, 1.23)
**FLUPHLA**	0.12 (0.01, 1.47)	0.2 (0.03, 1.5)	0.36 (0.06, 2.07)	0.2 (0.03, 1.52)	0.36 (0.06, 2.09)
**GinkBil+HAL**	0.85 (0.3, 2.36)	0.89 (0.4, 1.98)	0.85 (0.48, 1.48)	0.89 (0.4, 1.98)	0.85 (0.48, 1.48)
**HAL**	0.53 (0.38, 0.74)	0.55 (0.42, 0.72)	0.68 (0.55, 0.83)	0.55 (0.42, 0.72)	0.68 (0.55, 0.83)
**LAM+CLOZ**	5.4 (0.52, 56.19)	7.57 (0.85, 67.37)	6.21 (0.75, 51.57)	7.57 (0.85, 67.37)	6.16 (0.75, 50.91)
**LEV**	1.17 (0.34, 4.02)	1.4 (0.53, 3.73)	0.94 (0.49, 1.77)	1.42 (0.53, 3.79)	0.94 (0.5, 1.79)
**LI+HAL**	0.43 (0.03, 6.14)	0.44 (0.05, 3.93)	0.56 (0.08, 4.05)	0.44 (0.05, 3.93)	0.57 (0.08, 4.07)
**MINOC+CLOZ**	5.23 (0.47, 58.68)	7.33 (0.83, 64.8)	5.76 (0.71, 46.86)	7.33 (0.83, 64.8)	5.72 (0.71, 46.26)
**MIRT+RISP**	3.68 (0.3, 45.36)	4.62 (0.62, 34.72)	2.49 (0.51, 12.1)	4.62 (0.62, 34.73)	2.49 (0.51, 12.14)
**OLA**	0.91 (0.66, 1.27)	0.93 (0.72, 1.2)	0.96 (0.8, 1.14)	0.92 (0.71, 1.19)	0.95 (0.8, 1.14)
**OLA+RISP**	1.65 (0.41, 6.61)	1.69 (0.55, 5.2)	1.61 (0.63, 4.15)	1.68 (0.55, 5.19)	1.61 (0.63, 4.15)
**PALIP**	0.64 (0.2, 1.98)	0.64 (0.26, 1.56)	0.79 (0.42, 1.49)	0.64 (0.26, 1.55)	0.79 (0.42, 1.48)
**PBO**	0.05 (0.01, 0.21)	0.07 (0.02, 0.22)	0.16 (0.07, 0.38)	0.07 (0.02, 0.22)	0.16 (0.07, 0.38)
**PERPH**	1.08 (0.22, 5.42)	1.24 (0.35, 4.4)	0.91 (0.42, 1.95)	1.25 (0.35, 4.44)	0.91 (0.43, 1.97)
**PIPOTLA**	0.12 (0.01, 1.47)	0.2 (0.03, 1.5)	0.36 (0.06, 2.07)	0.2 (0.03, 1.52)	0.36 (0.06, 2.09)
**PTX+RISP**	2.69 (0.7, 10.29)	2.89 (1.01, 8.3)	2.07 (0.89, 4.85)	2.89 (1.01, 8.3)	2.07 (0.89, 4.85)
**QUE**	0.75 (0.45, 1.23)	0.84 (0.56, 1.26)	0.84 (0.61, 1.16)	0.85 (0.56, 1.28)	0.85 (0.62, 1.16)
**RISP**	0.74 (0.51, 1.07)	0.75 (0.56, 1.01)	0.87 (0.72, 1.06)	0.76 (0.56, 1.01)	0.87 (0.72, 1.06)
**RISP+CLOZ**	1.06 (0.4, 2.8)	1.06 (0.5, 2.26)	1.05 (0.53, 2.09)	1.06 (0.5, 2.26)	1.05 (0.53, 2.08)
**SERTIND**	0.46 (0.23, 0.93)	0.47 (0.27, 0.82)	0.69 (0.47, 1.01)	0.47 (0.27, 0.82)	0.69 (0.47, 1.01)
**SUL**	2.12 (0.43, 10.51)	2.98 (0.81, 10.93)	0.85 (0.47, 1.55)	3.07 (0.84, 11.27)	0.87 (0.48, 1.59)
**SUL+CLOZ**	0.73 (0.11, 5.05)	0.71 (0.16, 3.23)	0.86 (0.31, 2.36)	0.71 (0.16, 3.23)	0.86 (0.31, 2.36)
**THIO**	0.44 (0.13, 1.5)	0.5 (0.18, 1.37)	0.61 (0.32, 1.16)	0.51 (0.18, 1.4)	0.62 (0.32, 1.17)
**TOPIR+CLOZ**	5.8 (0.66, 50.81)	7 (1.18, 41.36)	3.97 (0.85, 18.65)	7 (1.18, 41.36)	3.99 (0.85, 18.77)
**TRIFLUO**	0.86 (0.15, 4.8)	1.14 (0.29, 4.56)	0.73 (0.35, 1.51)	1.17 (0.29, 4.66)	0.74 (0.36, 1.53)
**ZIP**	0.75 (0.42, 1.34)	0.89 (0.55, 1.43)	0.79 (0.58, 1.08)	0.91 (0.56, 1.46)	0.8 (0.58, 1.09)

**ZIP+CLOZ**	0.69 (0.05, 9.66)	0.64 (0.08, 5.42)	0.95 (0.31, 2.94)	0.64 (0.08, 5.42)	0.95 (0.31, 2.93)

Table S 12.1. Other models for dichotomous outcomes: Response rates
AMI: amisulpride; ARI: aripiprazole; CLOZ: clozapine; CPZ: chlorpromazine; DESMOP: desmopressin; DONEP: donepezil; FLUPH: fluphenazine; FLUPHLA: fluphenazine decanoate; GinkBil: Ginkgo biloba; HAL: haloperidol; IV: inverse variance; LAM: lamotrigine; LEV: levomepromazine; LI: lithium; MH: Mantel-Haenszel; MINOC: minocycline; MIRT: mirtazapine; OLA: olanzapine; PALIP: paliperidone; PBO: placebo; PERPH: perphenazine; PIPOTLA: pipotiazine long-acting; PTX: pentoxifylline; QUE: quetiapine; RISP: risperidone; SERTIND: sertindole; SUL: sulpiride; THIO: thioridazine; TOPIR: topiramate; TRIFLUO: trifluoperazine; VORT: vortioxetine; ZIP: ziprasidone.

12.2.Other models for dichotomous outcomes: Drop-outs due to any reason

Treatment	Firth results	Common MH With Correction	Common NCH With Correction	Common MH Without Correction	Common NCH Without Correction
**AMI**	1.37 (0.79, 2.38)	1.36 (0.79, 2.37)	1.25 (0.77, 2.03)	1.36 (0.78, 2.37)	1.26 (0.78, 2.06)
**AMI+CLOZ**	0.35 (0.14, 0.84)	0.34 (0.14, 0.83)	0.4 (0.18, 0.92)	0.34 (0.14, 0.83)	0.4 (0.18, 0.92)
**AMI+OLA**	0.51 (0.05, 5.02)	0.42 (0.03, 5.41)	0.46 (0.04, 5.07)	0.42 (0.03, 5.41)	0.45 (0.04, 5.05)
**ARI+CLOZ**	1.5 (0.77, 2.93)	1.52 (0.77, 3.01)	1.44 (0.76, 2.71)	1.52 (0.77, 3.01)	1.44 (0.76, 2.71)
**ARI+OLA**	1.02 (0.02, 56.9)				
**BEZOAET+CLOZ**	0.16 (0.01, 4.34)	0.16 (0.01, 4.12)	0.17 (0.01, 4.2)		
**CELECOX+CLOZ**	1.48 (0.25, 8.89)	1.59 (0.24, 10.7)	1.51 (0.25, 9.03)	1.59 (0.24, 10.7)	1.5 (0.25, 9)
**CEREBR+RISP**	0.66 (0.16, 2.78)	0.62 (0.14, 2.82)	0.62 (0.15, 2.62)	0.63 (0.14, 2.85)	0.63 (0.15, 2.66)
**CLOT**	0.3 (0.06, 1.57)	0.25 (0.04, 1.42)	0.32 (0.07, 1.54)	0.24 (0.04, 1.38)	0.31 (0.06, 1.51)
**CPZ**	1.6 (1.15, 2.21)	1.57 (1.14, 2.18)	1.34 (1.03, 1.75)	1.54 (1.11, 2.13)	1.31 (1, 1.72)
**CPZ+TRIFLUO**	15.4 (0.14, 1643.15)				
**Dcyclos+CLOZ**	0.21 (0.03, 1.54)	0.15 (0.02, 1.46)	0.2 (0.02, 1.71)	0.15 (0.02, 1.46)	0.2 (0.02, 1.71)
**DESMOP+RISP**	1.1 (0.16, 7.51)	1.1 (0.14, 8.77)	1.05 (0.15, 7.54)	1.12 (0.14, 8.88)	1.06 (0.15, 7.61)
**DONEP+CLOZ**	6.54 (0.22, 195.51)	6.54 (0.27, 160.97)	5.03 (0.24, 105.41)		
**Dser+CLOZ**	1 (0.01, 67.23)				
**DUL+CLOZ**	0.73 (0.15, 3.59)	0.71 (0.14, 3.66)	0.75 (0.17, 3.35)	0.71 (0.14, 3.66)	0.75 (0.17, 3.35)
**FLUO+CLOZ**	0.26 (0.01, 7.58)	0.26 (0.01, 6.9)	0.28 (0.01, 6.9)		
**FLUPH**	3.22 (1.19, 8.72)	3.24 (1.17, 8.96)	1.94 (0.91, 4.16)	3.26 (1.18, 9)	1.94 (0.91, 4.16)
**FLUPHLA**	3.52 (0.24, 50.78)	2.33 (0.19, 29.12)	2.18 (0.5, 9.59)	2.94 (0.12, 72.26)	1.88 (0.36, 9.87)
**FLUV+CLOZ**	2.95 (0.66, 13.19)	3.42 (0.65, 18)	3.08 (0.62, 15.26)	3.42 (0.65, 18)	3.08 (0.62, 15.29)
**GinkBil+CLOZ**	2.56 (0.33, 19.84)	3.3 (0.32, 34.35)	3 (0.31, 28.8)	3.3 (0.32, 34.35)	3.01 (0.31, 29.06)
**GinkBil+HAL**	0.49 (0.07, 3.2)	0.35 (0.04, 3.18)	0.28 (0.03, 2.38)	0.36 (0.04, 3.21)	0.28 (0.03, 2.39)
**GLY+CLOZ**	2.01 (0.37, 11.1)	2.31 (0.35, 15.23)	1.99 (0.36, 10.86)	2.31 (0.35, 15.23)	1.98 (0.36, 10.84)
**HAL**	2.05 (1.62, 2.61)	2.03 (1.6, 2.58)	1.46 (1.23, 1.73)	2.05 (1.61, 2.61)	1.46 (1.23, 1.74)
**HAL+CLOZ**	1.07 (0.37, 3.08)	1.08 (0.37, 3.12)	1.13 (0.45, 2.87)	1.08 (0.37, 3.12)	1.14 (0.45, 2.87)
**IMI+FLUPHLA**	5.87 (0.04, 815.39)				
**LAM+CLOZ**	1.58 (0.61, 4.05)	1.62 (0.61, 4.26)	1.44 (0.62, 3.38)	1.62 (0.61, 4.26)	1.44 (0.62, 3.38)
**LEV**	0.6 (0.11, 3.37)	0.52 (0.08, 3.19)	0.54 (0.1, 2.83)	0.51 (0.08, 3.11)	0.52 (0.1, 2.77)
**LI+FLUPHLA**	5.63 (0.29, 107.61)	3.82 (0.23, 64.26)	2.65 (0.49, 14.17)	4.81 (0.15, 150.42)	2.28 (0.36, 14.29)
**LI+HAL**	10.27 (0.37, 281.88)	10.14 (0.43, 239.86)	6.19 (0.29, 130.34)		
**MAZIN+CLOZ**	1 (0.02, 58.29)				
**MEM+CLOZ**	1 (0.21, 4.66)	1 (0.19, 5.29)	1 (0.2, 4.96)	1 (0.19, 5.29)	1 (0.2, 4.96)
**MET+CLOZ**	0.29 (0.04, 2.03)	0.25 (0.03, 1.82)	0.4 (0.08, 2.06)	0.25 (0.03, 1.82)	0.4 (0.08, 2.06)
**MINOC+CLOZ**	4.27 (0.18, 99.7)	4.27 (0.2, 93.52)	4.02 (0.19, 84.35)		
**MIRT+CLOZ**	1 (0.13, 7.62)	1 (0.12, 8.56)	1 (0.14, 7.12)	1 (0.12, 8.56)	1 (0.14, 7.1)
**MIRT+RISP**	2.74 (0.08, 89.92)	2.74 (0.1, 76.03)	2.43 (0.1, 60.56)		
**MODF+CLOZ**	0.21 (0.04, 1.07)	0.18 (0.03, 0.98)	0.26 (0.06, 1.24)	0.18 (0.03, 0.98)	0.26 (0.06, 1.24)
**Nacetyl+CLOZ**	1.05 (0.45, 2.45)	1.05 (0.45, 2.45)	1.02 (0.56, 1.87)	1.05 (0.45, 2.45)	1.02 (0.56, 1.87)
**OLA**	1.02 (0.82, 1.27)	1.01 (0.81, 1.26)	0.99 (0.84, 1.17)	1.01 (0.81, 1.26)	0.99 (0.84, 1.17)
**OLA+CLOZ**	10.71 (0.31, 369.06)	10.71 (0.4, 287.83)	6.7 (0.32, 140.46)		
**OLA+RISP**	0.74 (0.29, 1.88)	0.73 (0.28, 1.87)	0.85 (0.45, 1.62)	0.73 (0.29, 1.88)	0.86 (0.45, 1.64)
**ONDAST+HAL**	1.23 (0.44, 3.42)	1.18 (0.42, 3.34)	0.92 (0.36, 2.37)	1.2 (0.42, 3.37)	0.93 (0.36, 2.39)

**ONDAST+RISP**	2.2 (0.39, 12.39)	2.45 (0.39, 15.33)	2.11 (0.38, 11.66)	2.48 (0.4, 15.51)	2.13 (0.38, 11.77)
**OXYT+RISP**	0.63 (0.07, 5.62)	0.52 (0.04, 6.37)	0.52 (0.05, 5.83)	0.53 (0.04, 6.44)	0.53 (0.05, 5.88)
**PALIP**	0.91 (0.3, 2.72)	0.9 (0.3, 2.74)	0.9 (0.33, 2.43)	0.9 (0.3, 2.73)	0.9 (0.33, 2.44)
**PALIP+OLA**	1.02 (0.02, 56.9)				
**PALIPLA**	0.69 (0.11, 4.39)	0.66 (0.1, 4.5)	0.68 (0.11, 4.08)	0.66 (0.1, 4.5)	0.68 (0.11, 4.08)
**PALMIT+RISP**	1.1 (0.34, 3.54)	1.1 (0.34, 3.57)	1.05 (0.38, 2.85)	1.12 (0.35, 3.62)	1.06 (0.39, 2.89)
**PBO**	8.4 (0.99, 71.36)	6.5 (0.99, 42.73)	2.7 (0.73, 9.99)	8.2 (0.54, 124.93)	2.33 (0.52, 10.5)
**PHENYLPROP+CLOZ**	1 (0.11, 8.92)	1 (0.1, 9.61)	1 (0.14, 7.12)	1 (0.1, 9.61)	1 (0.14, 7.1)
**PIMOZ+CLOZ**	1.11 (0.31, 3.96)	1.11 (0.29, 4.18)	1.1 (0.32, 3.8)	1.11 (0.29, 4.18)	1.1 (0.32, 3.78)
**PIPOTLA**	3.52 (0.24, 50.78)	2.33 (0.19, 29.12)	2.18 (0.5, 9.59)	2.94 (0.12, 72.26)	1.88 (0.36, 9.86)
**PTX+RISP**	0.91 (0.25, 3.26)	0.89 (0.24, 3.31)	0.88 (0.26, 2.92)	0.9 (0.24, 3.35)	0.88 (0.26, 2.95)
**QUE**	1.68 (1.18, 2.4)	1.64 (1.14, 2.35)	1.34 (1.01, 1.78)	1.61 (1.12, 2.32)	1.32 (0.99, 1.75)
**QUE+CLOZ**	1.49 (0.18, 12.34)	2 (0.18, 21.85)	2.02 (0.2, 20.14)	2 (0.18, 21.85)	2.03 (0.2, 20.25)
**RESVER+RISP**	1.1 (0.26, 4.76)	1.1 (0.24, 5.01)	1.05 (0.26, 4.26)	1.12 (0.25, 5.07)	1.06 (0.26, 4.3)
**RISP**	1.1 (0.85, 1.44)	1.1 (0.85, 1.44)	1.05 (0.85, 1.29)	1.12 (0.86, 1.46)	1.06 (0.86, 1.31)
**RISP+CLOZ**	1.47 (0.65, 3.31)	1.5 (0.64, 3.52)	1.38 (0.64, 2.97)	1.5 (0.64, 3.52)	1.38 (0.64, 2.97)
**SARC+CLOZ**	1 (0.01, 67.23)				
**SER+OLA**	0.38 (0.05, 3.16)	0.3 (0.03, 3.2)	0.33 (0.03, 3.19)	0.29 (0.03, 3.2)	0.33 (0.03, 3.18)
**SER+ZIP**	0.46 (0.13, 1.64)	0.42 (0.11, 1.57)	0.44 (0.13, 1.49)	0.42 (0.11, 1.55)	0.44 (0.13, 1.48)
**SERTIND**	1.43 (0.81, 2.53)	1.44 (0.81, 2.55)	1.26 (0.78, 2.02)	1.46 (0.82, 2.58)	1.27 (0.79, 2.04)
**SERTIND+CLOZ**	1 (0.23, 4.33)	1 (0.22, 4.54)	1 (0.25, 4)	1 (0.22, 4.54)	1 (0.25, 4)
**SUL**	0.22 (0.01, 4.06)	0.38 (0.04, 3.81)	0.36 (0.04, 3.53)		
**SUL+CLOZ**	0.76 (0.01, 47.16)				
**SUL+OLA**	3.05 (0.09, 105.53)	3.04 (0.11, 86.2)	2.69 (0.11, 66.58)		
**THIO**	15.89 (1.47, 172.24)	8.91 (1.17, 67.71)	4.72 (0.85, 26.17)	11.26 (0.27, 476.72)	3.2 (0.16, 62.63)
**TOPIR+CLOZ**	2.56 (1.14, 5.74)	2.64 (1.17, 5.99)	2.09 (1.02, 4.29)	2.64 (1.17, 5.99)	2.09 (1.02, 4.29)
**TRIFLUO**	16.61 (1.57, 175.14)	11.09 (1.36, 90.42)	4.8 (0.92, 25.18)	15.47 (0.7, 342.69)	4.1 (0.52, 32.14)
**VALPRO+RISP**	0.34 (0.07, 1.59)	0.29 (0.06, 1.53)	0.31 (0.06, 1.52)	0.3 (0.06, 1.55)	0.32 (0.06, 1.54)
**VitD3+CLOZ**	0.65 (0.11, 3.81)	0.61 (0.09, 4.01)	0.64 (0.11, 3.82)	0.61 (0.09, 4.01)	0.64 (0.11, 3.82)
**VORT+RISP**	1.1 (0.3, 4.12)	1.1 (0.29, 4.28)	1.05 (0.3, 3.7)	1.12 (0.29, 4.33)	1.06 (0.3, 3.73)
**ZIP**	1.13 (0.68, 1.87)	1.12 (0.67, 1.85)	1.04 (0.68, 1.58)	1.1 (0.66, 1.83)	1.03 (0.68, 1.57)
**ZIP+CLOZ**	1.4 (0.35, 5.55)	1.44 (0.34, 6.09)	1.35 (0.36, 5.09)	1.44 (0.34, 6.09)	1.34 (0.36, 5.07)
**ZOT**	2.79 (1.01, 7.68)	3.07 (1.05, 8.96)	2.19 (0.91, 5.3)	3.07 (1.05, 8.96)	2.19 (0.91, 5.29)

Table S 12.2. Other models for dichotomous outcomes: Drop-outs due to any reason
AMI: amisulpride; ARI: aripiprazole; BEZOAET: benzoate sodium; CELECOX: celecoxib; CEREBR: cerebrolysin; CLOZ: clozapine; CLOT: clotapine; CPZ: chlorpromazine; Dcyclos: D-cycloserine; DESMOP: desmopressin; DONEP: donepezil; Dser: D-serine; DUL: duloxetine; FLUO: fluoxetine; FLUPH: fluphenazine; FLUPHLA: fluphenazine decanoate; FLUV: fluvoxamine; GinkBil: Ginkgo biloba; GLY: glycine; HAL: haloperidol; IMI: imipramine; LAM: lamotrigine; LEV: levomepromazine; IV: inverse variance; LI: lithium; MH: Mantel-Haenszel; MAZIN: mazindol; MEM: memantine; MET: metformin; MINOC: minocycline; MIRT: mirtazapine; MODF: modafinil; Nacetyl: N-acetylcysteine; OLA: olanzapine; ONDAST: ondansetron; OXYT: oxytocin; PALMIT: palmitoylethanolamide; PALIP: paliperidone; PALIPLA: paliperidone long-acting; PBO: placebo; PHENPROP: phenylpropanolamine; PIMOZ: pimozide; PTX: pentoxifylline; QUE: quetiapine; RESVER: resveratrol; RISP: risperidone; SARC: sarcosine; SER: sertraline; SERTIND: sertindole; SUL: sulpiride; THIO: thioridazine; TOPIR: topiramate; TRIFLUO: trifluoperazine; VALPRO: valproate; VitD3: vitamin D3; VORT: vortioxetine; ZIP: ziprasidone; ZOT: zotepine.

12.3. Other models for dichotomous outcomes: Drop-outs due to any adverse effect

Treatment	Firth results	Common MH With Correction	Common NCH With Correction	Common MH Without Correction	Common NCH Without Correction
**AMI**	1.6 (0.44, 5.74)	1.94 (0.48, 7.88)	1.98 (0.5, 7.9)	1.65 (0.39, 6.96)	1.69 (0.41, 6.98)
**AMI+CLOZ**	0.79 (0.21, 2.97)	0.78 (0.19, 3.14)	0.8 (0.21, 2.98)	0.78 (0.19, 3.14)	0.8 (0.21, 2.98)
**AMI+OLA**	0.47 (0.01, 30.79)				
**ARI+CLOZ**	1.71 (0.53, 5.48)	1.82 (0.51, 6.51)	1.75 (0.51, 5.97)	1.82 (0.51, 6.51)	1.74 (0.51, 5.97)
**ARI+OLA**	0.51 (0.01, 29.09)				
**BEZOAET+CLOZ**	0.51 (0.01, 28.46)				
**CELECOX+CLOZ**	1 (0.02, 58.29)				
**CPZ**	2.93 (1.63, 5.28)	2.91 (1.59, 5.32)	2.58 (1.46, 4.54)	3.1 (1.65, 5.84)	2.79 (1.53, 5.09)
**CPZ+TRIFLUO**	5.42 (0.03, 1032.48)				
**Dser+CLOZ**	1 (0.01, 67.23)				
**DUL+CLOZ**	8.2 (0.37, 183.11)	8.2 (0.4, 169.9)	6.99 (0.36, 134.92)		
**FLUO+CLOZ**	0.84 (0.01, 50.43)				
**FLUPH**	0.44 (0.06, 3.18)	0.76 (0.04, 13.38)	0.77 (0.05, 12.72)	0.83 (0.05, 14.67)	0.84 (0.05, 13.95)
**FLUPHLA**	10.1 (0.4, 252.51)	13.84 (0.34, 563.76)	9.04 (0.25, 321.78)		
**FLUV+CLOZ**	1.74 (0.24, 12.45)	2.1 (0.18, 24.07)	2.05 (0.19, 22.6)	2.1 (0.18, 24.07)	2.05 (0.19, 22.57)
**GinkBil+HAL**	0.09 (0, 1.85)	0.1 (0, 2)	0.1 (0.01, 2.08)		
**GLY+CLOZ**	3 (0.1, 90.82)	3 (0.11, 80.39)	2.8 (0.11, 68.63)		
**HAL**	0.69 (0.44, 1.07)	0.76 (0.47, 1.22)	0.77 (0.49, 1.2)	0.83 (0.5, 1.37)	0.84 (0.53, 1.34)
**IMI+FLUPHLA**	16.84 (0.09, 3214.72)				
**LAM+CLOZ**	0.67 (0.13, 3.56)	0.62 (0.09, 4.32)	0.67 (0.11, 3.99)	0.62 (0.09, 4.32)	0.67 (0.11, 3.99)
**LEV**	2.93 (0.24, 35.16)	2.91 (0.16, 53.46)	2.57 (0.15, 43.54)	3.1 (0.17, 57.4)	2.8 (0.16, 47.71)
**LI+HAL**	1.88 (0.06, 61.86)	2.08 (0.07, 58.87)	1.95 (0.08, 49.35)		
**MAZIN+CLOZ**	1 (0.02, 58.29)				
**MEM+CLOZ**	1 (0.09, 10.73)	1 (0.06, 16.89)	1 (0.06, 15.96)	1 (0.06, 16.89)	1 (0.06, 15.96)
**MINOC+CLOZ**	0.8 (0.01, 44.98)				
**MIRT+CLOZ**	1 (0.02, 64.13)				
**MODF+CLOZ**	0.19 (0.01, 4.57)	0.19 (0.01, 4.22)	0.21 (0.01, 4.37)		
**Nacetyl+CLOZ**	10.17 (0.51, 201.82)	10.17 (0.53, 195.02)	9.19 (0.5, 170.42)		
**OLA**	0.51 (0.31, 0.83)	0.56 (0.33, 0.94)	0.59 (0.36, 0.97)	0.53 (0.3, 0.94)	0.56 (0.33, 0.97)
**OLA+CLOZ**	10.71 (0.31, 369.06)	10.71 (0.4, 287.83)	6.65 (0.32, 138.17)		
**OLA+RISP**	0.54 (0.11, 2.6)	0.58 (0.1, 3.34)	0.61 (0.12, 3.15)	0.52 (0.09, 3.02)	0.55 (0.11, 2.88)
**ONDAST+HAL**	0.46 (0.11, 2.01)	0.48 (0.1, 2.27)	0.5 (0.11, 2.24)	0.52 (0.11, 2.5)	0.55 (0.12, 2.47)
**ONDAST+RISP**	3.69 (0.15, 90.82)	3.94 (0.17, 91.42)	3.67 (0.17, 79.58)		
**OXYT+RISP**	0.67 (0.01, 40.41)				
**PALIP**	0.14 (0.02, 1.04)	0.12 (0.01, 1.16)	0.14 (0.01, 1.28)	0.11 (0.01, 1.12)	0.13 (0.01, 1.23)
**PALIP+OLA**	0.51 (0.01, 29.09)				
**PBO**	2.77 (0.17, 45.9)	3.19 (0.12, 87.95)	3.01 (0.12, 77.04)		
**PIMOZ+CLOZ**	2.1 (0.43, 10.34)	2.37 (0.4, 14.07)	2.2 (0.4, 11.99)	1.77 (0.27, 11.58)	1.67 (0.28, 10.02)
**PIPOTLA**	6.61 (0.26, 166.94)	8.9 (0.22, 366.16)	6.78 (0.19, 244.99)		
**PTX+RISP**	0.67 (0.01, 37.83)				
**QUE**	1.31 (0.68, 2.5)	1.26 (0.64, 2.47)	1.21 (0.64, 2.28)	1.26 (0.57, 2.79)	1.25 (0.6, 2.6)
**RESVER+RISP**	0.67 (0.01, 38.52)				

**RISP**	0.67 (0.41, 1.1)	0.72 (0.42, 1.24)	0.74 (0.44, 1.22)	0.58 (0.32, 1.06)	0.61 (0.34, 1.09)
**RISP+CLOZ**	7.9 (0.6, 104.56)	3.64 (0.49, 27.3)	3.51 (0.47, 26.24)		
**SARC+CLOZ**	1 (0.01, 67.23)				
**SER+OLA**	0.19 (0.02, 1.65)	0.16 (0.01, 1.85)	0.2 (0.02, 2.01)	0.15 (0.01, 1.78)	0.19 (0.02, 1.93)
**SER+ZIP**	0.37 (0.07, 1.85)	0.31 (0.06, 1.74)	0.32 (0.06, 1.7)	0.32 (0.06, 1.78)	0.33 (0.06, 1.74)
**SERTIND**	1.08 (0.36, 3.26)	1.23 (0.38, 3.93)	1.22 (0.4, 3.72)	0.99 (0.3, 3.26)	1.01 (0.32, 3.19)
**SERTIND+CLOZ**	0.58 (0.07, 4.91)	0.48 (0.04, 5.65)	0.5 (0.05, 5.52)	0.48 (0.04, 5.65)	0.5 (0.05, 5.51)
**SUL**	0.75 (0.04, 14.31)	0.94 (0.04, 25.3)	0.86 (0.03, 22.14)		
**SUL+CLOZ**	0.76 (0.01, 47.16)				
**THIO**	4.44 (0.26, 74.83)	4.59 (0.2, 107.14)	4.12 (0.19, 89.8)		
**TOPIR+CLOZ**	35.24 (2.11, 588.21)	35.53 (2.02, 624.72)	24.98 (1.48, 421.6)		
**TRIFLUO**	5.84 (0.2, 168.51)	7.03 (0.09, 552.29)	6.41 (0.09, 456.93)		
**ZIP**	1.35 (0.68, 2.67)	1.35 (0.68, 2.67)	1.25 (0.67, 2.34)	1.38 (0.69, 2.73)	1.28 (0.68, 2.4)
**ZIP+CLOZ**	8.25 (0.52, 129.98)	4.43 (0.41, 47.57)	4.14 (0.44, 39.12)		
**ZOT**	2.13 (0.08, 57.98)	2.13 (0.08, 54.52)	2.08 (0.08, 51.05)		

Table S 12.3. Other models for dichotomous outcomes: Drop-outs due to any adverse effect
AMI: amisulpride; ARI: aripiprazole; BEZOAET: benzoate sodium; CELECOX: celecoxib; CEREBR: cerebrolysin; CLOZ: clozapine; CLOT: clotapine; CPZ: chlorpromazine; Dcyclos: D-cycloserine; DESMOP: desmopressin; DONEP: donepezil; Dser: D-serine; DUL: duloxetine; FLUO: fluoxetine; FLUPH: fluphenazine; FLUPHLA: fluphenazine decanoate; FLUV: Fluvoxamine; GinkBil: Ginkgo biloba; GLY: glycine; HAL: haloperidol; IMI: imipramine; LAM: lamotrigine; LEV: levomepromazine; IV: inverse variance; LI: lithium; MH: Mantel-Haenszel; MAZIN: mazindol; MEM: memantine; MET: metformin; MINOC: minocycline; MIRT: mirtazapine; MODF: modafinil; Nacetyl: N-acetylcysteine; OLA: olanzapine; ONDAST: ondansetron; OXYT: Oxytocin; PALIP: paliperidone; PBO: placebo; PIMOZ: pimozide; PIPOTLA: pipotiazine long-acting; QUE: quetiapine; RESVER: resveratrol; RISP: risperidone; SARC: sarcosine; SER: sertraline; SERTIND: sertindole; SUL: sulpiride; THIO: thioridazine; TOPIR: topiramate; TRIFLUO: trifluoperazine; ZIP: ziprasidone; ZOT: zotepine

12.4.Other models for dichotomous outcomes: Drop-outs due to inefficacy

Treatment	Firth results	Common MH With Correction	Common NCH With Correction	Common MH Without Correction	Common NCH Without Correction
**AMI**	2.1 (0.8, 5.5)	2.04 (0.74, 5.59)	1.93 (0.73, 5.14)	2.01 (0.73, 5.53)	1.92 (0.72, 5.11)
**AMI+CLOZ**	0.2 (0.01, 3.72)	0.17 (0.01, 3.89)	0.23 (0.01, 3.71)	0.17 (0.01, 3.89)	0.23 (0.01, 3.71)
**AMI+OLA**	1.06 (0.11, 10.51)	0.85 (0.07, 11.06)	0.89 (0.08, 10.07)	0.84 (0.06, 10.94)	0.88 (0.08, 9.93)
**ARI+CLOZ**	5.06 (0.36, 72.13)	3.08 (0.31, 30.14)	3.03 (0.31, 29.18)		
**ARI+HAL**	5 (1.21, 20.65)	4.85 (1.1, 21.39)	3.64 (0.88, 15.08)	4.83 (1.09, 21.31)	3.63 (0.88, 15.04)
**ARI+OLA**	2.1 (0.04, 118.42)				
**BEZOAET+CLOZ**	0.16 (0.01, 4.34)	0.16 (0.01, 4.12)	0.17 (0.01, 4.2)		
**CELECOX+CLOZ**	1 (0.02, 58.29)				
**CEREBR+RISP**	1.13 (0.15, 8.56)	0.89 (0.09, 9.38)	0.77 (0.08, 7.67)	0.92 (0.09, 9.67)	0.78 (0.08, 7.82)
**CPZ**	1.71 (0.73, 4.02)	1.76 (0.65, 4.73)	1.66 (0.69, 4)	1.75 (0.65, 4.71)	1.65 (0.68, 3.99)
**CPZ+TRIFLUO**	9.26 (0.07, 1288.28)				
**Dser+CLOZ**	1 (0.01, 67.23)				
**DUL+CLOZ**	0.32 (0.01, 8.95)	0.32 (0.01, 8.26)	0.33 (0.01, 8.19)		
**FLUO+CLOZ**	0.92 (0.03, 24.52)				
**FLUPH**	12.8 (3.9, 42.07)	13.3 (3.99, 44.28)	6.9 (2.58, 18.41)	13.41 (4.03, 44.67)	6.94 (2.6, 18.53)
**FLUPHLA**	1.78 (0.12, 26.43)	2.08 (0.09, 46.78)	2.19 (0.38, 12.67)	2.07 (0.09, 46.6)	2.17 (0.37, 12.56)
**GinkBil+HAL**	2.78 (0.34, 22.85)	2.25 (0.19, 26.21)	1.73 (0.15, 19.41)	2.24 (0.19, 26.11)	1.71 (0.15, 19.28)
**GLY+CLOZ**	0.93 (0.01, 58.17)				
**HAL**	5 (3.55, 7.02)	4.85 (3.4, 6.91)	3.64 (2.66, 4.98)	4.83 (3.39, 6.89)	3.63 (2.65, 4.96)
**IMI+FLUPHLA**	2.96 (0.02, 418.4)				
**LAM+CLOZ**	3.18 (0.11, 92.1)	3.18 (0.12, 83.76)	2.98 (0.12, 72.74)		
**LEV**	0.31 (0.01, 8.3)	0.32 (0.01, 8.2)	0.33 (0.01, 7.83)		
**LI+HAL**	4.2 (0.06, 279.38)				
**MAZIN+CLOZ**	1 (0.02, 58.29)				
**MINOC+CLOZ**	0.8 (0.01, 44.98)				
**MIRT+CLOZ**	1 (0.02, 64.13)				
**Nacetyl+CLOZ**	1.02 (0.02, 55.26)				
**OLA**	2.1 (1.48, 2.97)	2.04 (1.44, 2.89)	1.94 (1.4, 2.67)	2.02 (1.42, 2.86)	1.92 (1.39, 2.65)
**OLA+RISP**	2.16 (0.81, 5.73)	2.14 (0.79, 5.77)	1.98 (0.92, 4.26)	2.17 (0.8, 5.86)	2 (0.93, 4.31)
**ONDAST+HAL**	4.39 (1.13, 16.99)	4.17 (1.02, 17.08)	3.16 (0.82, 12.22)	4.15 (1.01, 17.02)	3.15 (0.82, 12.18)
**ONDAST+RISP**	2.8 (0.05, 165.28)				
**OXYT+RISP**	2.8 (0.05, 167.45)				
**PALIP**	4.67 (0.62, 34.98)	5.83 (0.57, 59.9)	5.3 (0.54, 52.11)	5.76 (0.56, 59.24)	5.26 (0.53, 51.89)
**PALIP+OLA**	2.1 (0.04, 118.42)				
**PALIPLA**	4.67 (0.05, 411.75)				
**PBO**	14.36 (1.47, 140.07)	19.39 (1.26, 299.27)	5.83 (1.26, 26.89)	19.32 (1.25, 298.12)	5.78 (1.25, 26.68)
**PIMOZ+CLOZ**	0.21 (0.01, 4.82)	0.21 (0.01, 4.54)	0.22 (0.01, 4.65)		
**PIPOTLA**	2.72 (0.19, 39.89)	3.23 (0.15, 71.89)	2.91 (0.52, 16.38)	3.22 (0.14, 71.61)	2.89 (0.51, 16.24)
**PTX+RISP**	2.8 (0.05, 156.75)				
**QUE**	4.78 (2.48, 9.21)	4.72 (2.4, 9.3)	3.54 (1.97, 6.37)	4.73 (2.4, 9.32)	3.55 (1.97, 6.39)
**RESVER+RISP**	2.8 (0.05, 159.63)				
**RISP**	2.8 (1.75, 4.47)	2.84 (1.72, 4.69)	2.33 (1.52, 3.59)	2.93 (1.77, 4.85)	2.39 (1.55, 3.69)

**RISP+CLOZ**	1.88 (0.29, 12.37)	2.26 (0.2, 26.13)	2.19 (0.2, 24.16)	2.26 (0.2, 26.13)	2.18 (0.2, 24.02)
**SARC+CLOZ**	1 (0.01, 67.23)				
**SER+OLA**	2.1 (0.03, 127.42)				
**SER+ZIP**	1.7 (0.02, 122.62)				
**SERTIND**	3.69 (1.64, 8.27)	3.82 (1.66, 8.78)	3 (1.42, 6.35)	3.93 (1.71, 9.06)	3.07 (1.45, 6.52)
**SERTIND+CLOZ**	1.46 (0.25, 8.47)	1.57 (0.24, 10.3)	1.5 (0.25, 8.96)	1.57 (0.24, 10.3)	1.5 (0.25, 8.95)
**SUL**	1.25 (0.04, 35.06)				
**SUL+CLOZ**	0.76 (0.01, 47.16)				
**THIO**	20.52 (1.11, 378.14)				
**TOPIR+CLOZ**	1 (0.04, 25.86)				
**TRIFLUO**	9.98 (0.58, 171.96)	20.68 (0.52, 829.5)	6.21 (0.36, 106.79)	20.6 (0.51, 826.31)	6.14 (0.36, 105.71)
**ZIP**	1.45 (0.29, 7.18)	1.54 (0.25, 9.51)	1.52 (0.25, 9.08)	1.54 (0.25, 9.51)	1.52 (0.25, 9.07)
**ZIP+CLOZ**	1.79 (0.23, 13.78)	2.11 (0.18, 25.35)	2.01 (0.18, 22.14)	2.11 (0.18, 25.35)	2 (0.18, 22.01)
**ZOT**	10.8 (0.55, 213.57)	10.8 (0.58, 201.37)	9 (0.51, 159.23)		

Table S 12.4. Other models for dichotomous outcomes: Drop-outs due to inefficacy

AMI: amisulpride; ARI: aripiprazole; BEZOAET: benzoate sodium; CELECOX: celecoxib; CEREBR: cerebrolysin; CLOZ: clozapine; CLOT: clotapine; CPZ: chlorpromazine; Dcyclos: D-cycloserine; DESMOP: desmopressin; DONEP: donepezil; Dser: D-serine; DUL: duloxetine; FLUO: fluoxetine; FLUPH: fluphenazine; FLUPHLA: fluphenazine decanoate; FLUV: Fluvoxamine; GinkBil: Ginkgo biloba; GLY: glycine; HAL: haloperidol; IMI: imipramine; LAM: lamotrigine; LEV: levomepromazine; IV: inverse variance; LI: lithium; MH: Mantel-Haenszel; MAZIN: mazindol; MEM: memantine; MET: metformin; MINOC: minocycline; MIRT: mirtazapine; MODF: modafinil; Nacetyl: N-acetylcysteine; OLA: olanzapine; ONDAST: ondansetron; OXYT: oxytocin; PALIP: paliperidone; PBO: placebo; PIMOZ: pimozide; QUE: quetiapine; RESVER: resveratrol; RISP: risperidone; SARC: sarcosine; SER: sertraline; SERTIND: sertindole; SUL: sulpiride; THIO: thioridazine; TOPIR: topiramate; TRIFLUO: trifluoperazine; ZIP: ziprasidone; ZOT: zotepine.

12.5.Other models for dichotomous outcomes: Total number of participants with adverse effects

Treatment	Firth results	Common MH With Correction	Common NCH With Correction	Common MH Without Correction	Common NCH Without Correction
**AMI+CLOZ**	3.32 (0.93, 11.86)	3.45 (1.26, 9.42)	1.96 (0.93, 4.16)	3.45 (1.26, 9.42)	1.97 (0.93, 4.18)
**AMI+OLA**	1.04 (0.09, 12.35)	1.2 (0.16, 9.09)	1.94 (0.37, 10.26)	1.2 (0.16, 9.09)	1.94 (0.37, 10.28)
**ARI+CLOZ**	1.67 (0.81, 3.44)	1.67 (0.95, 2.97)	1.21 (0.85, 1.7)	1.67 (0.95, 2.97)	1.21 (0.85, 1.7)
**CELECOX+CLOZ**	1 (0.01, 176.51)				
**CPZ**	0.95 (0.44, 2.05)	0.96 (0.53, 1.75)	1 (0.71, 1.41)	0.96 (0.53, 1.75)	1 (0.71, 1.41)
**CPZ+TRIFLUO**	1.5 (0.19, 11.56)	1.6 (0.3, 8.42)	1.53 (0.35, 6.67)	1.6 (0.3, 8.42)	1.54 (0.35, 6.73)
**FLUPH**	0.4 (0.07, 2.41)	0.4 (0.1, 1.64)	0.69 (0.24, 1.97)	0.4 (0.1, 1.64)	0.69 (0.24, 1.97)
**HAL**	0.56 (0.2, 1.56)	0.56 (0.25, 1.27)	0.86 (0.53, 1.4)	0.56 (0.25, 1.27)	0.86 (0.53, 1.4)
**MEM+CLOZ**	0.86 (0.22, 3.45)	0.86 (0.29, 2.55)	0.92 (0.42, 2.02)	0.86 (0.29, 2.55)	0.93 (0.42, 2.03)
**MET+CLOZ**	6.18 (0.09, 423.75)	6.18 (0.26, 146.78)	1.23 (0.5, 3.05)		
**OLA**	0.38 (0.16, 0.92)	0.38 (0.18, 0.81)	0.84 (0.62, 1.12)	0.38 (0.18, 0.81)	0.84 (0.62, 1.12)
**ONDAST+HAL**	0.5 (0.11, 2.26)	0.51 (0.16, 1.66)	0.84 (0.45, 1.59)	0.51 (0.16, 1.66)	0.84 (0.45, 1.58)
**PBO**	0.09 (0.01, 1.47)	0.07 (0.01, 0.74)	0.16 (0.02, 1.38)	0.07 (0.01, 0.74)	0.16 (0.02, 1.38)
**QUE**	0.5 (0.22, 1.16)	0.51 (0.26, 0.98)	0.82 (0.56, 1.19)	0.51 (0.26, 0.98)	0.82 (0.56, 1.19)
**RISP**	0.32 (0.08, 1.34)	0.32 (0.11, 1)	0.71 (0.35, 1.44)	0.32 (0.11, 1)	0.71 (0.35, 1.44)
**RISP+CLOZ**	1.47 (0.41, 5.28)	1.49 (0.54, 4.08)	1.14 (0.64, 2.05)	1.49 (0.54, 4.08)	1.14 (0.64, 2.05)
**SERTIND**	0.34 (0.07, 1.82)	0.35 (0.09, 1.28)	0.72 (0.34, 1.52)	0.35 (0.09, 1.28)	0.72 (0.34, 1.52)
**SUL**	0.42 (0.1, 1.81)	0.41 (0.13, 1.3)	0.76 (0.39, 1.45)	0.41 (0.13, 1.3)	0.76 (0.39, 1.45)
**THIO**	0.99 (0.13, 7.58)	1.04 (0.21, 5.14)	1.18 (0.39, 3.54)	1.04 (0.21, 5.14)	1.17 (0.39, 3.53)
**TRIFLUO**	4.15 (0.31, 54.92)	4.69 (0.58, 37.7)	3.31 (0.54, 20.43)	4.69 (0.58, 37.7)	3.34 (0.54, 20.62)
**ZIP**	0.69 (0.27, 1.78)	0.68 (0.32, 1.45)	0.91 (0.62, 1.32)	0.68 (0.32, 1.45)	0.91 (0.62, 1.32)

Table S 12.5. Other models for dichotomous outcomes: Total number of participants with adverse effects
AMI: amisulpride; ARI: aripiprazole; CELECOX: celecoxib; CLOZ: clozapine; CPZ: chlorpromazine; FLUPH: fluphenazine; HAL: haloperidol; MH: Mantel-Haenszel; MEM: memantine; MET: metformin; OLA: olanzapine; ONDAST: ondansetron; PBO: placebo; QUE: quetiapine; RISP: risperidone; SERTIND: sertindole; SUL: sulpiride; THIO: thioridazine; TRIFLUO: trifluoperazine; ZIP: ziprasidone.

12.6. Other models for dichotomous outcomes: Antiparkinsonian medication use

Treatment	Firth results	Common MH With Correction	Common NCH With Correction	Common MH Without Correction	Common NCH Without Correction
**AMI**	1.12 (0.31, 4.1)	1.11 (0.31, 4.04)	1.07 (0.33, 3.53)	1.07 (0.29, 3.9)	1.05 (0.32, 3.45)
**ARI+CLOZ**	0.68 (0.23, 2)	0.67 (0.22, 2)	0.69 (0.24, 1.98)	0.67 (0.22, 2)	0.69 (0.24, 1.98)
**ARI+HAL**	4.17 (0.39, 44.7)	3.47 (0.25, 48.91)	2.06 (0.16, 25.83)	3 (0.21, 42.32)	1.8 (0.14, 22.68)
**CPZ**	2.4 (0.19, 29.91)	1.84 (0.13, 26.78)	1.36 (0.12, 15.07)	1.58 (0.11, 23.17)	1.19 (0.11, 13.22)
**FLUPH**	6.61 (1.83, 23.86)	6.29 (1.73, 22.87)	4.02 (1.6, 10.09)	5.43 (1.48, 19.9)	3.53 (1.4, 8.94)
**FLUPHLA**	2.99 (0.04, 231.32)	2.23 (0.03, 164.26)	1.78 (0.03, 102.16)		
**HAL**	7.09 (2.51, 20)	7.08 (2.5, 20.06)	4.11 (1.84, 9.14)	6.11 (2.14, 17.44)	3.61 (1.61, 8.11)
**LEV**	0.69 (0.04, 13.17)	0.47 (0.02, 10.38)	0.51 (0.03, 7.95)	0.41 (0.02, 8.97)	0.44 (0.03, 6.97)
**LI+HAL**	3.94 (0.43, 35.73)	3.54 (0.38, 32.59)	3.42 (0.97, 12.13)	3.05 (0.33, 28.21)	3.01 (0.84, 10.71)
**MINOC+CLOZ**	1.03 (0.21, 5.03)	1.07 (0.21, 5.33)	1.06 (0.24, 4.72)	1.07 (0.21, 5.33)	1.06 (0.24, 4.73)
**MIRT+RISP**	1.21 (0.16, 9.27)	1 (0.13, 7.62)	1.69 (0.5, 5.64)	0.84 (0.11, 6.41)	1.46 (0.43, 4.91)
**OLA**	1.12 (0.71, 1.77)	1.11 (0.71, 1.74)	1.07 (0.73, 1.55)	1.07 (0.69, 1.67)	1.04 (0.71, 1.51)
**OLA+RISP**	2.2 (0.76, 6.42)	2.14 (0.76, 6)	1.77 (0.76, 4.14)	1.88 (0.67, 5.31)	1.6 (0.69, 3.73)
**OXYT+RISP**	3 (0.72, 12.52)	2.85 (0.71, 11.53)	2.41 (0.91, 6.34)	2.38 (0.59, 9.71)	2.08 (0.78, 5.53)
**PBO**	0.57 (0.03, 11.28)	0.42 (0.02, 9.33)	0.38 (0.02, 6.24)	0.36 (0.02, 8.07)	0.33 (0.02, 5.46)
**PIPOTLA**	2.24 (0.03, 178.6)	1.67 (0.02, 126.56)	1.39 (0.02, 81.9)		
**QUE**	1.6 (0.55, 4.6)	1.61 (0.57, 4.55)	1.15 (0.47, 2.81)	1.43 (0.5, 4.08)	1.05 (0.43, 2.57)
**RISP**	3.64 (1.95, 6.8)	3.5 (1.9, 6.45)	2.63 (1.57, 4.41)	2.93 (1.56, 5.49)	2.27 (1.33, 3.87)
**RISP+CLOZ**	2.81 (0.09, 90.86)	2.81 (0.11, 74.56)	2.65 (0.11, 65)		
**SUL**	1.37 (0.09, 20.44)	1.03 (0.06, 17.68)	1.11 (0.09, 13.1)	0.89 (0.05, 15.29)	0.97 (0.08, 11.49)
**THIO**	0.91 (0.04, 19.4)	0.61 (0.02, 15.25)	0.54 (0.03, 9.99)	0.53 (0.02, 13.19)	0.47 (0.03, 8.75)
**TRIFLUO**	6.35 (0.37, 109.69)	5.16 (0.26, 101.52)	2.62 (0.19, 36.28)	4.45 (0.23, 87.81)	2.27 (0.16, 31.76)
**ZIP**	1.38 (0.69, 2.76)	1.39 (0.71, 2.73)	1.13 (0.75, 1.7)	1.39 (0.71, 2.73)	1.13 (0.75, 1.7)
**ZIP+CLOZ**	1 (0.02, 64.64)				
**ZOT**	29.4 (1.44, 598.37)	29.4 (1.65, 523.79)	18.68 (1.12, 312.86)		

Table S 12.6. Other models for dichotomous outcomes: Antiparkinson medication use
AMI: amisulpride; ARI: aripiprazole; CPZ: chlorpromazine; FLUPH: fluphenazine; FLUPHLA: fluphenazine decanoate; HAL: haloperidol; LEV: levomepromazine; LI: lithium; IV: inverse variance; MH: Mantel-Haenszel; MINOC: minocycline; MIRT: mirtazapine; OLA: olanzapine; OXYT: oxytocin; PBO: placebo; QUE: quetiapine; RISP: risperidone; SUL: sulpiride; THIO: thioridazine; TRIFLUO: trifluoperazine; ZIP: ziprasidone; ZOT: zotepine

12.7. Other models for dichotomous outcomes: Sedation

Treatment	Firth results	Common MH With Correction	Common NCH With Correction	Common MH Without Correction	Common NCH Without Correction
**AMI**	0.15 (0.06, 0.39)	0.14 (0.05, 0.38)	0.31 (0.14, 0.68)	0.14 (0.05, 0.38)	0.3 (0.14, 0.67)
**AMI+OLA**	0.12 (0, 3.84)	0.12 (0, 3.36)	0.2 (0.01, 4.94)		
**ARI+CLOZ**	3.18 (0.42, 23.96)	4.25 (0.43, 42.19)	3.6 (0.4, 32.19)	4.25 (0.43, 42.19)	3.6 (0.4, 32.17)
**BEZOAET+CLOZ**	0.51 (0.01, 28.46)				
**CELECOX+CLOZ**	1 (0.02, 58.29)				
**CPZ**	0.57 (0.4, 0.82)	0.58 (0.4, 0.84)	0.69 (0.51, 0.94)	0.58 (0.4, 0.84)	0.69 (0.51, 0.94)
**DESMOP+RISP**	0.94 (0.16, 5.38)	1.04 (0.16, 6.61)	1.28 (0.23, 7.2)	1.04 (0.16, 6.61)	1.29 (0.23, 7.22)
**FLUPH**	0.42 (0.1, 1.85)	0.41 (0.09, 1.81)	0.57 (0.17, 1.97)	0.41 (0.09, 1.81)	0.58 (0.17, 1.97)
**FLUV+CLOZ**	0.87 (0.4, 1.88)	0.86 (0.39, 1.88)	0.9 (0.46, 1.76)	0.86 (0.39, 1.88)	0.9 (0.46, 1.76)
**HAL**	0.45 (0.29, 0.69)	0.43 (0.28, 0.68)	0.84 (0.7, 1.01)	0.43 (0.28, 0.68)	0.84 (0.7, 1.01)
**LAM+CLOZ**	1.5 (0.24, 9.46)	1.62 (0.23, 11.26)	1.5 (0.25, 8.97)	1.62 (0.23, 11.26)	1.5 (0.25, 9)
**LEV**	1.02 (0.21, 5.05)	1.11 (0.21, 5.73)	1.15 (0.26, 4.95)	1.11 (0.21, 5.73)	1.15 (0.27, 4.96)
**MINOC+CLOZ**	2.32 (0.56, 9.5)	2.54 (0.59, 10.95)	2.12 (0.56, 8)	2.54 (0.59, 10.95)	2.12 (0.56, 7.98)
**MIRT+CLOZ**	9.21 (0.37, 227.5)	9.21 (0.42, 200.59)	6.94 (0.36, 133.15)		
**MIRT+RISP**	3.28 (0.12, 87.3)	3.26 (0.14, 73.91)	3.44 (0.18, 67.31)		
**OLA**	0.43 (0.31, 0.61)	0.43 (0.3, 0.61)	0.64 (0.5, 0.82)	0.43 (0.3, 0.61)	0.64 (0.5, 0.82)
**OXYT+RISP**	0.59 (0.14, 2.47)	0.6 (0.14, 2.54)	0.77 (0.23, 2.62)	0.6 (0.14, 2.54)	0.77 (0.23, 2.63)
**PALMIT+RISP**	0.24 (0.04, 1.33)	0.22 (0.04, 1.34)	0.32 (0.06, 1.8)	0.22 (0.04, 1.34)	0.32 (0.06, 1.81)
**PBO**	0.42 (0.08, 2.14)	0.41 (0.08, 2.22)	0.52 (0.11, 2.38)	0.41 (0.08, 2.22)	0.52 (0.11, 2.39)
**QUE**	0.4 (0.23, 0.7)	0.4 (0.23, 0.7)	0.55 (0.35, 0.87)	0.4 (0.23, 0.7)	0.55 (0.35, 0.87)
**RESVER+RISP**	0.47 (0.01, 26.54)				
**RISP**	0.47 (0.32, 0.69)	0.47 (0.32, 0.69)	0.64 (0.47, 0.87)	0.47 (0.32, 0.69)	0.64 (0.47, 0.87)
**RISP+CLOZ**	2 (0.76, 5.27)	2.05 (0.78, 5.39)	1.46 (0.72, 2.96)	2.05 (0.78, 5.39)	1.46 (0.72, 2.96)
**SER+ZIP**	0.21 (0.02, 1.81)	0.18 (0.01, 2.17)	0.24 (0.02, 2.75)	0.18 (0.01, 2.17)	0.24 (0.02, 2.76)
**SERTIND**	0.36 (0.17, 0.8)	0.37 (0.17, 0.81)	0.52 (0.26, 1.06)	0.37 (0.17, 0.81)	0.52 (0.26, 1.06)
**SERTIND+CLOZ**	1.24 (0.33, 4.65)	1.26 (0.33, 4.84)	1.2 (0.37, 3.93)	1.26 (0.33, 4.84)	1.2 (0.37, 3.93)
**SUL**	0.45 (0.18, 1.11)	0.44 (0.18, 1.12)	0.55 (0.25, 1.23)	0.44 (0.18, 1.12)	0.55 (0.25, 1.24)
**THIO**	0.74 (0.17, 3.35)	0.78 (0.17, 3.6)	0.86 (0.22, 3.31)	0.78 (0.17, 3.6)	0.86 (0.22, 3.33)
**TOPIR+CLOZ**	1 (0.02, 54.22)				
**TRIFLUO**	0.57 (0.12, 2.71)	0.58 (0.12, 2.86)	0.69 (0.17, 2.84)	0.58 (0.12, 2.86)	0.69 (0.17, 2.85)
**VORT+RISP**	0.47 (0.11, 2.01)	0.47 (0.1, 2.12)	0.64 (0.16, 2.65)	0.47 (0.1, 2.12)	0.64 (0.16, 2.66)
**ZIP**	0.31 (0.18, 0.53)	0.31 (0.18, 0.55)	0.41 (0.25, 0.67)	0.31 (0.18, 0.55)	0.41 (0.25, 0.67)
**ZIP+CLOZ**	3.15 (0.11, 89.06)	3.15 (0.12, 82.16)	2.97 (0.12, 72.52)		

Table S 12.7. Other models for dichotomous outcomes: Sedation
AMI: amisulpride; ARI: aripiprazole; BEZOAET: benzoate sodium; CELECOX: celecoxib; CLOZ: clozapine; CPZ: chlorpromazine; DESMOP: desmopressin; FLUPH: fluphenazine; FLUV: fluvoxamine; LAM: lamotrigine; LEV: levomepromazine; IV: inverse variance; MH: Mantel-Haenszel; MINOC: minocycline; MIRT: mirtazapine; OLA: olanzapine; OXYT: oxytocin; PALMIT: palmitoylethanolamide; QUE: quetiapine; RESVER: resveratrol; RISP: risperidone; SER: sertraline; SERTIND: sertindole; SUL: sulpiride; THIO: thioridazine; TOPIR: topiramate; TRIFLUO: trifluoperazine; VORT: vortioxetine; ZIP: ziprasidone

Appendix 13. Summary of statistical evaluation of heterogeneity and consistency for the network meta-analysis

We assessed the level of heterogeneity by comparing the estimated τ^2 to empirical distributions of heterogeneity commonly observed in meta-analyses[185]. Based on the predictive τ^2 distribution for mental health outcomes described by Rhodes et al.[185], the median is 0.049, with an interquartile range (IQR) of 0.01 to 0.242. We also calculated the variation percentage across studies due to heterogeneity rather than chance. To detect local inconsistency, we used a 10% threshold for significance[186,187]. Additionally, we evaluated the global consistency by applying a full design-by-treatment interaction model via the decompose.design function from the netmeta R package.

Outcome	Between study variance (τ^2)	Heterogeneity (I^2)	Design by treatment - Global Inconsistency			Percentage of loops exhibiting inconsistency using SIDE splitting
			Q	d. f.	p-value	
**Overall symptoms**	0.0841	58.60%	16.594	19	0.617	15.38%
**Positive symptoms**	0.0566	48.30%	8.666	17	0.950	4.55%
**Negative symptoms**	0.2799	81.80%	12.788	18	0.804	17.39%
**Depressive symptoms**	0.0438	42.60%	8.527	9	0.482	25%
**Response rates**	0.2182	43.90%	32.863	21	0.0478	17.65%
**Drop-outs due to any reason**	0.0267	7.80%	18.315	26	0.864	0%
**Drop-outs due to any adverse effects**	0	0%	7.710	13	0.862	0%
**Drop-outs due to inefficacy**	0.0682	10.30%	12.117	15	0.670	5.88%
**Total number of participants with adverse effects**	0.0234	8.90%	2.823	2	0.244	0%
**Antiparkinsonian medication use**	0.0505	8.30%	3.840	7	0.798	0%
**Sedation**	0	0%	9.853	11	0.544	5.56%
**Weight gain**	0.0489	42.60%	14.010	13	0.373	5.88%
**Prolactin elevation**	0.0759	43.00%	2.026	6	0.917	13.33%
**QTc prolongation**	0.4556	75.70%	8.242	1	0.004	0%
**Quality of life**	0.0034	7.70%	2.167	1	0.141	0%
**Social functioning**	0.5498	77.50%	1.995	2	0.369	0%

Table S 13. Heterogeneity and inconsistency for primary and secondary outcomes
I^2: percentage of variation across studies due to heterogeneity; **τ^2:** between-study variance; **SIDE:** Separate Indirect and Direct Evidence approach via netsplit command in R; **Q:** statistic assessing inconsistency under the assumption of a full design-by-treatment interaction random effects model using the decomp.design command in R; **df:** degrees of freedom for Q; **p:** p-value for Q

Appendix 14. Confidence evaluation in network meta-analysis: CINeMA

14.1. Reasons for downgrading

We assessed the confidence in the evidence of NMA estimates for the primary outcome using the Confidence in Network Meta-Analysis (CINeMA) framework[183] and the official online tool available at https://cinema.ispm.unibe.ch/ .The CINeMA framework evaluates six key domains, influencing the confidence level in NMA results, listed below:

1. Within-study bias
2. Reporting bias
3. Indirectness
4. Imprecision
5. Heterogeneity
6. Incoherence

Each comparison is assessed across these domains, leading to an overall confidence rating for a specific comparison. The tool utilizes original data estimating the contribution of each study to the effect size of a given comparison and consequently generates a contribution matrix. This process requires specific settings and judgments, which are described in detail in the following sections.

Domain 1: Within-study bias
We assigned an overall risk of bias rating for each study based on the assessment of individual risk of bias items according to Furukawa et al.[188]. The Table S below (29) outlines how the global rating was determined based on the individual components of the risk of bias tool.

Global rating	Items with high risk	Items with unclear risk
**low risk**	0	=< 3
**moderate**	0	>3
**moderate**	1	any
**high risk**	>1	any

Table S 14.1. Global risk of bias rating based on the individual risk of bias items

Domain 2: Reporting bias
The evaluation of reporting bias was carried out using the Risk of Bias due to Missing Evidence in Network Meta-Analysis (ROB-MEN) tool[189]. ROB-MEN, integrated within the CINeMA framework, constitutes a web application (https://cinema.ispm.unibe.ch/rob-men/) facilitating the evaluation of risk of bias as a result of missing evidence in the NMA estimates. The assessment of reporting bias was conducted based on the criteria as outlined in the ROB-MEN web application[189].

Domain 3: Indirectness
To assess indirectness, we evaluated each study based the listed factors: (a) age, (b) whether patients predominantly had negative symptoms, (c) the level of treatment resistance type of schizophrenia, schizoaffective disorder, or schizophreniform disorder in the baseline population, and (d) whether the primary study outcome focused on drug efficacy. Our assessment was classified as “high=3” if at least two of these criteria were met, if the population had predominantly negative symptoms, including adolescents or children, or if the primary outcome differed from efficacy. It was classified as “moderate=2” if it involved only a lower level of treatment resistance, and “low=1” if none of the above-mentioned criteria were present.

Domain 4: Imprecision
For this domain, thresholds for clinically meaningful differences between interventions need to be established. We defined SMDs outside the range of -0.1 to +0.1 as clinically significant.

Domain 5: Heterogeneity
Heterogeneity is important if a prediction interval includes values that lead to a different conclusion than an assessment based on the confidence interval. We defined SMDs outside the range of -0.1 to +0.1 as clinically significant. Heterogeneity was assessed by examining the 95% CIs and PIs of the SMDs. If the SMDs fell outside the range of -0.1 to +0.1, they were considered clinically significant. In other words, to explain and quantify heterogeneity, we used prediction intervals, which reveals us the plausible range of values for an effect size in a

future trial. This is the question one is really interested in when it comes to heterogeneity [190]. In our analysis, we see that prediction intervals are rather large rendering our results inconclusive and prone to change in the future. Based on the overlap of prediction and confidence intervals with the clinically meaningful thresholds, the heterogeneity was categorized as “no concerns”, “some concerns”, or “major concerns”, following the CINeMA guidelines [183]

Domain 6: Incoherence

We defined SMDs outside the interval of -0.1 to +0.1 as clinically significant. Incoherence was assessed utilizing a design-by-treatment test for comparisons with only direct or indirect evidence, and the SIDE approach when both types of evidence were present, following the CINeMA documentation guidelines[183]. Consequently, comparisons were categorized based on incoherence evaluation as presenting “no concerns”, “some concerns”, or “major concerns”.

Overall confidence

The final step of CINeMA involved the evaluation for each of the six different domains to be summarized into an overall confidence rating for the NMA estimate for each comparison and a specific outcome. The summary of the judgements across the domains was based on four levels of confidence as outlined in the Grading of Recommendations Assessment, Development and Evaluation (GRADE) approach: (1)“very low quality”, (2) “low quality”, (3) “moderate quality”, or (4) “high quality” [191] . The interpretation of these four levels is provided below:

- “High quality”: We are confident that the true effect is very close to that of the effect estimate
- “Moderate quality”: We are moderately confident in the effect estimate: the true effect is expected to be close to the estimate, however it is possible to be significantly
- “Low quality”: Our confidence in the effect estimate is limited: the true effect might significantly differ from the effect estimate
- “Very low quality”: Our confidence in the effect estimate is very little: the true effect is likely to be significantly different from the effect estimate.

According to CINeMA guidelines[183], each comparison initially begins at the high confidence level. Then, the rating is downgraded by one level if there are “some concerns” and by two levels if there are “major concerns”. When multiple domains present concerns, it is recommended to evaluate them collectively as opposed to in isolation, since they might be interconnected. For instance, indirectness might involve intransitivity considerations, displaying intransitivity within the data as statistical incoherence. Additionally, heterogeneity is considered to increase imprecision in the treatment effects and might be associated with biases within studies or reporting bias. Considering the above mentioned guidelines [183], we adopted an approach followed in previous NMA studies[11,192] to determine an overall confidence level for each comparison, as described below:

- A single “some concerns” judgment results in a one-level downgrade
- A “major concerns” judgment results in a two-level downgrade
- Two “some concerns” judgments, if related, do not justify more than a one-level downgrade
- A “major concerns” judgment along with up to two “some concerns” or an additional “major concerns” judgment does not justify more than a two-level downgrade
- Two “major concerns” judgments, along with any further concerns, lead to a three-level downgrade.

14.2. CINeMA rating for all comparisons: Overall symptoms primary outcome

Comparison	n of studies	Within-study bias	Reporting bias	Indirectness	Imprecision	Heterogeneity	Incoherence	Confidence rating	Reason(s) for downgrading
Cloz:Ola	11	Some concerns	Low risk	Some concerns	Some concerns	Some concerns	No concerns	Low	[Within-study bias, Indirectness, Imprecision, Heterogeneity]
Cloz:Risp	8	Some concerns	Low risk	No concerns	No concerns	Major concerns	No concerns	Low	[Within-study bias, Heterogeneity]
Cloz:Hal	5	Some concerns	Low risk	No concerns	No concerns	Major concerns	No concerns	Low	[Within-study bias, Heterogeneity]
Cloz:RispCloz	5	No concerns	Low risk	No concerns	Some concerns	Some concerns	Major concerns	Low	[Imprecision, Heterogeneity]
AmiCloz:Cloz	4	Some concerns	Low risk	No concerns	Some concerns	Some concerns	No concerns	Low	[Within-study bias, Imprecision, Heterogeneity]
AriCloz:Cloz	4	Some concerns	Low risk	No concerns	Major concerns	No concerns	No concerns	Low	[Within-study bias, Imprecision]
Hal:Ola	4	Some concerns	Low risk	No concerns	No concerns	Major concerns	No concerns	Low	[Within-study bias, Heterogeneity]
Hal:Risp	4	Some concerns	Low risk	No concerns	Major concerns	No concerns	No concerns	Low	[Within-study bias, Imprecision]
Ola:Risp	4	Some concerns	Low risk	Some concerns	Major concerns	No concerns	No concerns	Low	[Within-study bias, Indirectness, Imprecision]
Cloz:Cpz	3	Some concerns	Low risk	No concerns	No concerns	Some concerns	Major concerns	Low	[Within-study bias, Heterogeneity, Incoherence]
Cloz:LamCloz	3	No concerns	Low risk	No concerns	No concerns	Major concerns	No concerns	Low	[Heterogeneity]
Cloz:TopirCloz	3	Some concerns	Low risk	No concerns	No concerns	Major concerns	No concerns	Low	[Within-study bias, Heterogeneity]
Cloz:MemCloz	2	No concerns	Low risk	No concerns	Some concerns	Some concerns	No concerns	Moderate	[Imprecision, Heterogeneity]
Cloz:MetCloz	2	No concerns	Low risk	Major concerns	Major concerns	No concerns	No concerns	Low	[Indirectness, Imprecision]

Cloz:PimozCloz	2	No concerns	Low risk	No concerns	Major concerns	No concerns	No concerns	Low	[Imprecision]
Cloz:Que	2	Some concerns	Low risk	No concerns	No concerns	Some concerns	No concerns	Moderate	[Within-study bias, Heterogeneity]
Cpz:Que	2	Some concerns	Low risk	No concerns	Major concerns	No concerns	No concerns	Low	[Within-study bias, Imprecision]
Fluph:Hal	2	Some concerns	Low risk	No concerns	Major concerns	No concerns	No concerns	Low	[Within-study bias, Imprecision]
Hal:LiHal	2	No concerns	Low risk	Some concerns	No concerns	No concerns	No concerns	Moderate	[Indirectness]
Ola:Que	2	Some concerns	Low risk	No concerns	No concerns	Major concerns	No concerns	Low	[Within-study bias, Heterogeneity]
OlaRisp:Risp	2	Some concerns	Low risk	Some concerns	Major concerns	No concerns	No concerns	Low	[Within-study bias, Indirectness, Imprecision]
Que:Risp	2	Some concerns	Low risk	No concerns	No concerns	Major concerns	No concerns	Low	[Within-study bias, Heterogeneity]
Ami:Ola	1	No concerns	Low risk	Some concerns	Major concerns	No concerns	No concerns	Low	[Indirectness, Imprecision]
AmiCloz:QueCloz	1	Major concerns	Some concerns	No concerns	No concerns	No concerns	No concerns	Low	[Within-study bias, Reporting bias]
AmiOla:Ola	1	Major concerns	Low risk	Some concerns	Major concerns	No concerns	No concerns	Very Low	[Within-study bias, Indirectness, Imprecision]
AriCloz:HalCloz	1	Major concerns	Low risk	No concerns	Major concerns	No concerns	No concerns	Low	[Within-study bias, Imprecision]
AriOla:Ola	1	No concerns	Some concerns	Major concerns	No concerns	No concerns	No concerns	Low	[Reporting bias, Indirectness]
AriOla:PalipOla	1	No concerns	Some concerns	Major concerns	Major concerns	No concerns	No concerns	Very Low	[Reporting bias, Indirectness, Imprecision]
BezoaetCloz:Cloz	1	No concerns	Low risk	No concerns	Major concerns	No concerns	No concerns	Low	[Imprecision]
CelecoxCloz:Cloz	1	Some concerns	Some concerns	Major concerns	Major concerns	No concerns	No concerns	Very Low	[Within-study bias, Reporting bias, Indirectness, Imprecision]
CerebrRisp:Risp	1	No concerns	Low risk	Major concerns	Major concerns	No concerns	No concerns	Low	[Indirectness, Imprecision]

Clot:Cpz	1	Major concerns	Low risk	No concerns	Major concerns	No concerns	No concerns	Low	[Within-study bias, Imprecision]
Cloz:DulCloz	1	No concerns	Some concerns	No concerns	No concerns	No concerns	No concerns	Moderate	[Reporting bias]
Cloz:FluvCloz	1	No concerns	Low risk	No concerns	Major concerns	No concerns	No concerns	Low	[Imprecision]
Cloz:GinkBilCloz	1	Some concerns	Some concerns	No concerns	Major concerns	No concerns	No concerns	Low	[Within-study bias, Reporting bias, Imprecision]
Cloz:GlyCloz	1	Some concerns	Some concerns	Some concerns	Major concerns	No concerns	No concerns	Very Low	[Within-study bias, Reporting bias, Indirectness, Imprecision]
Cloz:HalCloz	1	Major concerns	Low risk	No concerns	Major concerns	No concerns	No concerns	Low	[Within-study bias, Imprecision]
Cloz:MinocCloz	1	No concerns	Low risk	No concerns	Major concerns	No concerns	No concerns	Low	[Imprecision]
Cloz:MirtCloz	1	Some concerns	Some concerns	Some concerns	No concerns	No concerns	No concerns	Low	[Within-study bias, Reporting bas, Indirectness]
Cloz:ModfCloz	1	No concerns	Low risk	Some concerns	Major concerns	No concerns	No concerns	Low	[Indirectness, Imprecision]
Cloz:PhenylpropClo z	1	Some concerns	Some concerns	Major concerns	Major concerns	No concerns	No concerns	Very Low	[Within-study bias, Reporting bias, Indirectness, Imprecision]
Cloz:SarcCloz	1	No concerns	Low risk	No concerns	Major concerns	No concerns	No concerns	Low	[Imprecision]
Cloz:SertindCloz	1	Some concerns	Low risk	No concerns	Major concerns	No concerns	No concerns	Low	[Within-study bias, Imprecision]
Cloz:Sul	1	Some concerns	Low risk	No concerns	No concerns	Some concerns	No concerns	Moderate	[Within-study bias, Heterogeneity]
Cloz:SulCloz	1	Major concerns	Low risk	No concerns	Major concerns	No concerns	No concerns	Low	[Within-study bias, Imprecision]
Cloz:VitD3Cloz	1	No concerns	Low risk	Some concerns	Major concerns	No concerns	No concerns	Low	[Indirectness, Imprecision]

Cloz:Zip	1	Some concerns	Some concerns	Some concerns	Major concerns	No concerns	No concerns	Very Low	[Within-study bias, Reporting bias, Indirectness, Imprecision]
Cloz:ZipCloz	1	No concerns	Low risk	No concerns	No concerns	Some concerns	Major concerns	Low	[Heterogeneity]
Cloz:Zot	1	Major concerns	Low risk	Some concerns	Major concerns	No concerns	No concerns	Very Low	[Within-study bias, Indirectness, Imprecision]
Cpz:Lev	1	No concerns	Low risk	No concerns	Major concerns	No concerns	No concerns	Low	[Imprecision]
Cpz:Ola	1	Some concerns	Low risk	No concerns	No concerns	Major concerns	No concerns	Low	[Within-study bias, Heterogeneity]
Cpz:Zip	1	No concerns	Low risk	No concerns	Major concerns	No concerns	No concerns	Low	[Imprecision]
DesmopRisp:Risp	1	Major concerns	Low risk	Some concerns	No concerns	Some concerns	No concerns	Low	[Within-study bias, Indirectness, Heterogeneity]
Fluph:Que	1	Some concerns	Low risk	No concerns	Major concerns	No concerns	No concerns	Low	[Within-study bias, Imprecision]
Fluph:Risp	1	Some concerns	Low risk	No concerns	Major concerns	No concerns	No concerns	Low	[Within-study bias, Imprecision]
GinkBilHal:Hal	1	Some concerns	Some concerns	No concerns	Major concerns	No concerns	No concerns	Low	[Within-study bias, Reporting bias, Imprecision]
Hal:OndastHal	1	No concerns	Low risk	No concerns	Major concerns	No concerns	No concerns	Low	[Imprecision]
Hal:Que	1	Some concerns	Low risk	No concerns	Major concerns	No concerns	No concerns	Low	[Within-study bias, Imprecision]
MirtRisp:Risp	1	Some concerns	Low risk	Major concerns	Major concerns	No concerns	No concerns	Very Low	[Within-study bias, Indirectness, Imprecision]
Ola:OlaRisp	1	Some concerns	Some concerns	Some concerns	Major concerns	No concerns	No concerns	Very Low	[Within-study bias, Reporting bias, Indirectness, Imprecision]
Ola:Palip	1	Some concerns	Low risk	No concerns	Major concerns	No concerns	No concerns	Low	[Within-study bias, Imprecision]

Ola:PalipOla	1	No concerns	Some concerns	Major concerns	No concerns	No concerns	No concerns	Low	[Reporting bias, Indirectness]
Ola:SerOla	1	Some concerns	Some concerns	No concerns	Major concerns	No concerns	No concerns	Low	[Within-study bias, Reporting bias, Imprecision]
Ola:SulOla	1	Major concerns	Low risk	No concerns	Major concerns	No concerns	No concerns	Low	[Within-study bias, Imprecision]
OxytRisp:Risp	1	Major concerns	Low risk	Major concerns	No concerns	No concerns	No concerns	Low	[Within-study bias, Indirectness]
Palip:Palipla	1	Major concerns	Low risk	Some concerns	Major concerns	No concerns	No concerns	Very Low	[Within-study bias, Indirectness, Imprecision]
PalmitRisp:Risp	1	Major concerns	Some concerns	Major concerns	No concerns	Some concerns	No concerns	Very Low	[Within-study bias, Reporting bias, Indirectness, Heterogeneity]
PtxRisp:Risp	1	Major concerns	Low risk	Major concerns	Major concerns	No concerns	No concerns	Very Low	[Within-study bias, Indirectness, Imprecision]
ResverRisp:Risp	1	Major concerns	Some concerns	Major concerns	No concerns	Major concerns	No concerns	Very Low	[Within-study bias, Reporting bias, Indirectness, Heterogeneity]
Risp:Sertind	1	Some concerns	Some concerns	No concerns	Major concerns	No concerns	No concerns	Low	[Within-study bias, Reporting bias, Imprecision]
Risp:VortRisp	1	No concerns	Some concerns	Major concerns	No concerns	No concerns	No concerns	Low	[Reporting bias, Indirectness]
RispCloz:ZipCloz	1	Major concerns	Some concerns	No concerns	Major concerns	No concerns	Major concerns	Very Low	[Within-study bias, Reporting bias, Imprecision, Incoherence]
SerZip:Zip	1	No concerns	Low risk	No concerns	Major concerns	No concerns	No concerns	Low	[Imprecision]
Ami:AmiCloz	0	No concerns	Low risk	Some concerns	Major concerns	No concerns	No concerns	Low	[Indirectness, Imprecision]
Ami:AmiOla	0	No concerns	Low risk	Some concerns	Major concerns	No concerns	No concerns	Low	[Indirectness, Imprecision]

Ami:AriCloz	0	No concerns	Low risk	Some concerns	Major concerns	No concerns	No concerns	Low	[Indirectness, Imprecision]
Ami:AriOla	0	No concerns	Low risk	Major concerns	Some concerns	Some concerns	No concerns	Low	[Indirectiness. Imprecision, Heterogeneity]
Ami:BezoaetCloz	0	No concerns	Low risk	Some concerns	Major concerns	No concerns	No concerns	Low	[Indirectness, Imprecision]
Ami:CelecoxCloz	0	Some concerns	Low risk	Some concerns	Major concerns	No concerns	No concerns	Low	[Within-study bias, Indirectness, Imprecision]
Ami:CerebrRisp	0	No concerns	Low risk	Some concerns	Major concerns	No concerns	No concerns	Low	[Indirectness, Imprecision]
Ami:Clot	0	No concerns	Low risk	No concerns	Major concerns	No concerns	No concerns	Low	[Imprecision]
Ami:Cloz	0	No concerns	Low risk	Some concerns	Major concerns	No concerns	No concerns	Low	[Indirectness, Imprecision]
Ami:Cpz	0	No concerns	Low risk	Some concerns	Major concerns	No concerns	No concerns	Low	[Indirectness, Imprecision]
Ami:DesmopRisp	0	Major concerns	Low risk	Some concerns	Major concerns	No concerns	No concerns	Very Low	[Within-study bias, Indirectness, Imprecision]
Ami:DulCloz	0	No concerns	Low risk	Some concerns	No concerns	No concerns	No concerns	Moderate	[Indirectness]
Ami:Fluph	0	Some concerns	Low risk	Some concerns	Major concerns	No concerns	No concerns	Low	[Within-study bias, Indirectness, Imprecision]
Ami:FluvCloz	0	No concerns	Low risk	Some concerns	Major concerns	No concerns	No concerns	Low	[Indirectness, Imprecision]
Ami:GinkBilCloz	0	Some concerns	Low risk	Some concerns	Major concerns	No concerns	No concerns	Low	[Within-study bias, Indirectness, Imprecision]
Ami:GinkBilHal	0	Some concerns	Low risk	No concerns	Major concerns	No concerns	No concerns	Low	[Within-study bias, Imprecision]
Ami:GlyCloz	0	Some concerns	Low risk	Some concerns	Major concerns	No concerns	No concerns	Low	[Within-study bias, Indirectness, Imprecision]
Ami:Hal	0	No concerns	Low risk	Some concerns	Major concerns	No concerns	No concerns	Low	[Indirectness, Imprecision]
Ami:HalCloz	0	No concerns	Low risk	No concerns	Major concerns	No concerns	No concerns	Low	[Imprecision]

Ami:LamCloz	0	No concerns	Low risk	Some concerns	Major concerns	No concerns	No concerns	Low	[Indirectness, Imprecision]
Ami:Lev	0	No concerns	Low risk	No concerns	Major concerns	No concerns	No concerns	Low	[Imprecision]
Ami:LiHal	0	No concerns	Low risk	Some concerns	Major concerns	No concerns	No concerns	Low	[Indirectness, Imprecision]
Ami:MemCloz	0	No concerns	Low risk	Some concerns	Major concerns	No concerns	No concerns	Low	[Indirectness, Imprecision]
Ami:MetCloz	0	No concerns	Low risk	Some concerns	Major concerns	No concerns	No concerns	Low	[Indirectness, Imprecision]
Ami:MinocCloz	0	No concerns	Low risk	Some concerns	Major concerns	No concerns	No concerns	Low	[Indirectness, Imprecision]
Ami:MirtCloz	0	Some concerns	Low risk	Some concerns	Major concerns	No concerns	No concerns	Low	[Within-study bias, Indirectness, Imprecision]
Ami:MirtRisp	0	Some concerns	Low risk	Some concerns	Major concerns	No concerns	No concerns	Low	[Within-study bias, Indirectness, Imprecision]
Ami:ModfCloz	0	No concerns	Low risk	Some concerns	Major concerns	No concerns	No concerns	Low	[Indirectness, Imprecision]
Ami:OlaRisp	0	Some concerns	Low risk	Some concerns	Major concerns	No concerns	No concerns	Low	[Within-study bias, Indirectness, Imprecision]
Ami:OndastHal	0	No concerns	Low risk	No concerns	Major concerns	No concerns	No concerns	Low	[Imprecision]
Ami:OxytRisp	0	Major concerns	Low risk	Some concerns	No concerns	No concerns	No concerns	Low	[Within-study bias, Indirectness]
Ami:Palip	0	No concerns	Low risk	No concerns	Major concerns	No concerns	No concerns	Low	[Imprecision]
Ami:Palipla	0	No concerns	Low risk	Some concerns	Major concerns	No concerns	No concerns	Low	[Indirectness, Imprecision]
Ami:PalipOla	0	No concerns	Low risk	Major concerns	Major concerns	No concerns	No concerns	Low	[Indirectness, Imprecision]
Ami:PalmitRisp	0	Major concerns	Low risk	Some concerns	Major concerns	No concerns	No concerns	Very Low	[Within-study bias, Indirectness, Imprecision]
Ami:PhenylpropCloz	0	Some concerns	Low risk	Some concerns	Major concerns	No concerns	No concerns	Low	[Within-study bias, Indirectness, Imprecision]

Ami:PimozCloz	0	No concerns	Low risk	Some concerns	Major concerns	No concerns	No concerns	Low	[Indirectness, Imprecision]
Ami:PtxRisp	0	Major concerns	Low risk	Some concerns	Major concerns	No concerns	No concerns	Very Low	[Within-study bias, Indirectness, Imprecision]
Ami:Que	0	No concerns	Low risk	Some concerns	Some concerns	Some concerns	No concerns	Low	[Indirectness, Imprecision, Heterogeneity]
Ami:QueCloz	0	Major concerns	Low risk	No concerns	Major concerns	No concerns	No concerns	Low	[Within-study bias, Imprecision]
Ami:ResverRisp	0	Major concerns	Low risk	Some concerns	Major concerns	No concerns	No concerns	Very Low	[Within-study bias, Indirectness, Imprecision]
Ami:Risp	0	No concerns	Low risk	Some concerns	Major concerns	No concerns	No concerns	Low	[Indirectness, Imprecision]
Ami:RispCloz	0	No concerns	Low risk	No concerns	Major concerns	No concerns	No concerns	Low	[Imprecision]
Ami:SarcCloz	0	No concerns	Low risk	Some concerns	Major concerns	No concerns	No concerns	Low	[Indirectness, Imprecision]
Ami:SerOla	0	No concerns	Low risk	No concerns	Major concerns	No concerns	No concerns	Low	[Imprecision]
Ami:Sertind	0	Some concerns	Low risk	Some concerns	Major concerns	No concerns	No concerns	Low	[Within-study bias, Indirectness, Imprecision]
Ami:SertindCloz	0	Some concerns	Low risk	Some concerns	Major concerns	No concerns	No concerns	Low	[Within-study bias, Indirectness, Imprecision]
Ami:SerZip	0	No concerns	Low risk	No concerns	Major concerns	No concerns	No concerns	Low	[Imprecision]
Ami:Sul	0	Some concerns	Low risk	Some concerns	Some concerns	Some concerns	No concerns	Low	[Within-study bias, Indirectness, Imprecision, Heterogeneity]
Ami:SulCloz	0	No concerns	Low risk	Some concerns	Major concerns	No concerns	No concerns	Low	[Indirectness, Imprecision]
Ami:SulOla	0	No concerns	Low risk	No concerns	Major concerns	No concerns	No concerns	Low	[Imprecision]
Ami:TopirCloz	0	No concerns	Low risk	Some concerns	Major concerns	No concerns	No concerns	Low	[Indirectness, Imprecision]
Ami:VitD3Cloz	0	No concerns	Low risk	Some concerns	Major concerns	No concerns	No concerns	Low	[Indirectness, Imprecision]

Ami:VortRisp	0	No concerns	Low risk	Some concerns	Major concerns	No concerns	No concerns	Low	[Indirectness, Imprecision]
Ami:Zip	0	No concerns	Low risk	Some concerns	Major concerns	No concerns	No concerns	Low	[Indirectness, Imprecision]
Ami:ZipCloz	0	No concerns	Low risk	No concerns	Major concerns	No concerns	No concerns	Low	[Imprecision]
Ami:Zot	0	No concerns	Low risk	Some concerns	Major concerns	No concerns	No concerns	Low	[Indirectness, Imprecision]
AmiCloz:AmiOla	0	Major concerns	Low risk	Some concerns	Major concerns	No concerns	No concerns	Very Low	[Within-study bias, Indirectness, Imprecision]
AmiCloz:AriCloz	0	Some concerns	Low risk	No concerns	Major concerns	No concerns	No concerns	Low	[Within-study bias, Imprecision]
AmiCloz:AriOla	0	No concerns	Low risk	No concerns	Major concerns	No concerns	No concerns	Low	[Imprecision]
AmiCloz:BezoaetCloz	0	No concerns	Low risk	No concerns	Major concerns	No concerns	No concerns	Low	[Imprecision]
AmiCloz:CelecoxCloz	0	Some concerns	Low risk	No concerns	Major concerns	No concerns	No concerns	Low	[Within-study bias, Imprecision]
AmiCloz:CerebrRisp	0	Some concerns	Low risk	No concerns	Major concerns	No concerns	No concerns	Low	[Within-study bias, Imprecision]
AmiCloz:Clot	0	Some concerns	Low risk	No concerns	Major concerns	No concerns	No concerns	Low	[Within-study bias, Imprecision]
AmiCloz:Cpz	0	Some concerns	Low risk	No concerns	No concerns	No concerns	No concerns	Moderate	[Within-study bias]
AmiCloz:DesmopRisp	0	Major concerns	Low risk	No concerns	Major concerns	No concerns	No concerns	Low	[Within-study bias, Imprecision]
AmiCloz:DulCloz	0	No concerns	Low risk	No concerns	No concerns	Some concerns	No concerns	Moderate	[Heterogeneity]
AmiCloz:Fluph	0	Some concerns	Low risk	No concerns	No concerns	Some concerns	No concerns	Moderate	[Within-study bias, Heterogeneity]
AmiCloz:FluvCloz	0	No concerns	Low risk	No concerns	Major concerns	No concerns	No concerns	Low	[Imprecision]
AmiCloz:GinkBilCloz	0	Some concerns	Low risk	No concerns	Major concerns	No concerns	No concerns	Low	[Within-study bias, Imprecision]
AmiCloz:GinkBilHal	0	Some concerns	Low risk	No concerns	Major concerns	No concerns	No concerns	Low	[Within-study bias, Imprecision]

AmiCloz:GlyCloz	0	Some concerns	Low risk	No concerns	Major concerns	No concerns	No concerns	Low	[Within-study bias, Imprecision]
AmiCloz:Hal	0	Some concerns	Low risk	No concerns	No concerns	No concerns	No concerns	Moderate	[Within-study bias]
AmiCloz:HalCloz	0	Some concerns	Low risk	No concerns	Major concerns	No concerns	No concerns	Low	[Within-study bias, Imprecision]
AmiCloz:LamCloz	0	Some concerns	Low risk	No concerns	Major concerns	No concerns	No concerns	Low	[Within-study bias, Imprecision]
AmiCloz:Lev	0	Some concerns	Low risk	No concerns	Major concerns	No concerns	No concerns	Low	[Within-study bias, Imprecision]
AmiCloz:LiHal	0	Some concerns	Low risk	No concerns	Major concerns	No concerns	No concerns	Low	[Within-study bias, Imprecision]
AmiCloz:MemCloz	0	No concerns	Low risk	No concerns	Major concerns	No concerns	No concerns	Low	[Imprecision]
AmiCloz:MetCloz	0	Some concerns	Low risk	No concerns	Major concerns	No concerns	No concerns	Low	[Within-study bias, Imprecision]
AmiCloz:MinocCloz	0	No concerns	Low risk	No concerns	Major concerns	No concerns	No concerns	Low	[Imprecision]
AmiCloz:MirtCloz	0	Some concerns	Low risk	No concerns	Major concerns	No concerns	No concerns	Low	[Within-study bias, Imprecision]
AmiCloz:MirtRisp	0	Some concerns	Low risk	No concerns	Major concerns	No concerns	No concerns	Low	[Within-study bias, Imprecision]
AmiCloz:ModfCloz	0	No concerns	Low risk	No concerns	Major concerns	No concerns	No concerns	Low	[Imprecision]
AmiCloz:Ola	0	Some concerns	Low risk	No concerns	No concerns	Major concerns	No concerns	Low	[Within-study bias, Heterogeneity]
AmiCloz:OlaRisp	0	Some concerns	Low risk	No concerns	Major concerns	No concerns	No concerns	Low	[Within-study bias, Imprecision]
AmiCloz:OndastHal	0	Some concerns	Low risk	No concerns	Major concerns	No concerns	No concerns	Low	[Within-study bias, Imprecision]
AmiCloz:OxytRisp	0	Major concerns	Low risk	No concerns	No concerns	No concerns	No concerns	Low	[Within-study bias]
AmiCloz:Palip	0	Some concerns	Low risk	No concerns	Major concerns	No concerns	No concerns	Low	[Within-study bias, Imprecision]

AmiCloz:Palipla	0	Some concerns	Low risk	No concerns	Major concerns	No concerns	No concerns	Low	[Within-study bias, Imprecision]
AmiCloz:PalipOla	0	No concerns	Low risk	No concerns	Major concerns	No concerns	No concerns	Low	[Imprecision]
AmiCloz:PalmitRisp	0	Major concerns	Low risk	No concerns	Major concerns	No concerns	No concerns	Low	[Within-study bias, Imprecision]
AmiCloz:PhenylpropCloz	0	Some concerns	Low risk	No concerns	Major concerns	No concerns	No concerns	Low	[Within-study bias, Imprecision]
AmiCloz:PimozCloz	0	No concerns	Low risk	No concerns	Some concerns	Some concerns	No concerns	Moderate	[Imprecision, Heterogeneity]
AmiCloz:PtxRisp	0	Major concerns	Low risk	No concerns	Major concerns	No concerns	No concerns	Low	[Within-study bias, Imprecision]
AmiCloz:Que	0	Some concerns	Low risk	No concerns	No concerns	No concerns	No concerns	Moderate	[Within-study bias]
AmiCloz:ResverRisp	0	Major concerns	Low risk	No concerns	Major concerns	No concerns	No concerns	Low	[Within-study bias, Imprecision]
AmiCloz:Risp	0	Some concerns	Low risk	No concerns	No concerns	Major concerns	No concerns	Low	[Within-study bias, Heterogeneity]
AmiCloz:RispCloz	0	Some concerns	Low risk	No concerns	Major concerns	No concerns	No concerns	Low	[Within-study bias, Imprecision]
AmiCloz:SarcCloz	0	No concerns	Low risk	No concerns	Major concerns	No concerns	No concerns	Low	[Imprecision]
AmiCloz:SerOla	0	Some concerns	Low risk	No concerns	Major concerns	No concerns	No concerns	Low	[Within-study bias, Imprecision]
AmiCloz:Sertind	0	Some concerns	Low risk	No concerns	No concerns	No concerns	No concerns	Moderate	[Within-study bias]
AmiCloz:SertindCloz	0	Some concerns	Low risk	No concerns	Major concerns	No concerns	No concerns	Low	[Within-study bias, Imprecision]
AmiCloz:SerZip	0	Some concerns	Low risk	No concerns	Major concerns	No concerns	No concerns	Low	[Within-study bias, Imprecision]
AmiCloz:Sul	0	Some concerns	Low risk	No concerns	No concerns	No concerns	No concerns	Moderate	[Within-study bias]
AmiCloz:SulCloz	0	Major concerns	Low risk	No concerns	Major concerns	No concerns	No concerns	Low	[Within-study bias, Imprecision]

AmiCloz:SulOla	0	Major concerns	Low risk	No concerns	Major concerns	No concerns	No concerns	Low	[Within-study bias, Imprecision]
AmiCloz:TopirCloz	0	Some concerns	Low risk	No concerns	Major concerns	No concerns	No concerns	Low	[Within-study bias, Imprecision]
AmiCloz:VitD3Cloz	0	No concerns	Low risk	No concerns	Major concerns	No concerns	No concerns	Low	[Imprecision]
AmiCloz:VortRisp	0	Some concerns	Low risk	No concerns	Major concerns	No concerns	No concerns	Low	[Within-study bias, Imprecision]
AmiCloz:Zip	0	Some concerns	Low risk	No concerns	No concerns	Some concerns	No concerns	Moderate	[Within-study bias, Heterogeneity]
AmiCloz:ZipCloz	0	No concerns	Low risk	No concerns	Major concerns	No concerns	No concerns	Low	[Imprecision]
AmiCloz:Zot	0	Major concerns	Low risk	No concerns	Major concerns	No concerns	No concerns	Low	[Within-study bias, Imprecision]
AmiOla:AriCloz	0	Some concerns	Low risk	Some concerns	Major concerns	No concerns	No concerns	Low	[Within-study bias, Indirectness, Imprecision]
AmiOla:AriOla	0	No concerns	Low risk	Major concerns	Major concerns	No concerns	No concerns	Low	[Indirectness, Imprecision]
AmiOla:BezoaetCloz	0	No concerns	Low risk	Some concerns	Major concerns	No concerns	No concerns	Low	[Indirectness, Imprecision]
AmiOla:CelecoxCloz	0	Some concerns	Low risk	Some concerns	Major concerns	No concerns	No concerns	Low	[Within-study bias, Indirectness, Imprecision]
AmiOla:CerebrRisp	0	No concerns	Low risk	Some concerns	Major concerns	No concerns	No concerns	Low	[Indirectness, Imprecision]
AmiOla:Clot	0	Major concerns	Low risk	No concerns	Major concerns	No concerns	No concerns	Low	[Within-study bias, Imprecision]
AmiOla:Cloz	0	Major concerns	Low risk	Some concerns	Major concerns	No concerns	No concerns	Very Low	[Within-study bias, Indirectness, Imprecision]
AmiOla:Cpz	0	Some concerns	Low risk	Some concerns	No concerns	No concerns	No concerns	Moderate	[Within-study bias, Indirectness]
AmiOla:DesmopRisp	0	Major concerns	Low risk	Some concerns	Major concerns	No concerns	No concerns	Vey Low	[Within-study bias, Indirectness, Imprecision]
AmiOla:DulCloz	0	No concerns	Low risk	Some concerns	Major concerns	No concerns	No concerns	Low	[Indirectness, Imprecision]

AmiOla:Fluph	0	Some concerns	Low risk	Some concerns	No concerns	Some concerns	No concerns	Low	[Within-study bias, Indirectness, Heterogeneity]
AmiOla:FluvCloz	0	No concerns	Low risk	Some concerns	Major concerns	No concerns	No concerns	Low	[Indirectness, Imprecision]
AmiOla:GinkBilCloz	0	Some concerns	Low risk	Some concerns	Major concerns	No concerns	No concerns	Low	[Within-study bias, Indirectness, Imprecision]
AmiOla:GinkBilHal	0	Some concerns	Low risk	No concerns	Major concerns	No concerns	No concerns	Low	[Within-study bias, Imprecision]
AmiOla:GlyCloz	0	Some concerns	Low risk	Some concerns	Major concerns	No concerns	No concerns	Low	[Within-study bias, Indirectness, Imprecision]
AmiOla:Hal	0	Major concerns	Low risk	Some concerns	No concerns	Some concerns	No concerns	Low	[Within-study bias, Indirectness, Heterogeneity]
AmiOla:HalCloz	0	Major concerns	Low risk	No concerns	Major concerns	No concerns	No concerns	Low	[Within-study bias, Imprecision]
AmiOla:LamCloz	0	No concerns	Low risk	Some concerns	Major concerns	No concerns	No concerns	Low	[Indirectness, Imprecision]
AmiOla:Lev	0	No concerns	Low risk	No concerns	Major concerns	No concerns	No concerns	Low	[Imprecision]
AmiOla:LiHal	0	Some concerns	Low risk	Some concerns	Major concerns	No concerns	No concerns	Low	[Within-study bias, Indirectness, Imprecision]
AmiOla:MemCloz	0	No concerns	Low risk	Some concerns	Major concerns	No concerns	No concerns	Low	[Indirectness, Imprecision]
AmiOla:MetCloz	0	No concerns	Low risk	Some concerns	Major concerns	No concerns	No concerns	Low	[Indirectness, Imprecision]
AmiOla:MinocCloz	0	No concerns	Low risk	Some concerns	Major concerns	No concerns	No concerns	Low	[Indirectness, Imprecision]
AmiOla:MirtCloz	0	Some concerns	Low risk	Some concerns	Major concerns	No concerns	No concerns	Low	[Within-study bias, Indirectness, Imprecision]
AmiOla:MirtRisp	0	Some concerns	Low risk	Some concerns	Major concerns	No concerns	No concerns	Low	[Within-study bias, Indirectness, Imprecision]
AmiOla:ModfCloz	0	No concerns	Low risk	Some concerns	Major concerns	No concerns	No concerns	Low	[Indirectness, Imprecision]

AmiOla:OlaRisp	0	Some concerns	Low risk	Some concerns	Major concerns	No concerns	No concerns	Low	[Within-study bias, Indirectness, Imprecision]
AmiOla:OndastHal	0	No concerns	Low risk	No concerns	Major concerns	No concerns	No concerns	Low	[Imprecision]
AmiOla:OxytRisp	0	Major concerns	Low risk	Some concerns	Major concerns	No concerns	No concerns	Very Low	[Within-study bias, Indirectness, Imprecision]
AmiOla:Palip	0	Some concerns	Low risk	No concerns	Major concerns	No concerns	No concerns	Low	[Within-study bias, Imprecision]
AmiOla:Palipla	0	Major concerns	Low risk	Some concerns	Major concerns	No concerns	No concerns	Very Low	[Within-study bias, Indirectness, Imprecision]
AmiOla:PalipOla	0	No concerns	Low risk	Major concerns	Major concerns	No concerns	No concerns	Low	[Indirectness, Imprecision]
AmiOla:PalmitRisp	0	Major concerns	Low risk	Some concerns	Major concerns	No concerns	No concerns	Very Low	[Within-study bias, Indirectness, Imprecision]
AmiOla:Phenylprop Cloz	0	Some concerns	Low risk	Some concerns	Major concerns	No concerns	No concerns	Low	[Within-study bias, Indirectness, Imprecision]
AmiOla:PimozCloz	0	No concerns	Low risk	Some concerns	Major concerns	No concerns	No concerns	Low	[Indirectness, Imprecision]
AmiOla:PtxRisp	0	Major concerns	Low risk	Some concerns	Major concerns	No concerns	No concerns	Very Low	[Within-study bias, Indirectness, Imprecision]
AmiOla:Que	0	Some concerns	Low risk	Some concerns	No concerns	No concerns	No concerns	Moderate	[Within-study bias, Indirectness]
AmiOla:QueCloz	0	Major concerns	Low risk	No concerns	No concerns	No concerns	No concerns	Low	[Within-study bias]
AmiOla:ResverRisp	0	Major concerns	Low risk	Some concerns	Major concerns	No concerns	No concerns	Very Low	[Within-study bias, Indirectness, Imprecision]
AmiOla:Risp	0	Major concerns	Low risk	Some concerns	Some concerns	Some concerns	No concerns	Very Low	[Within-study bias, Indirectness, Imprecision, Heterogeneity]
AmiOla:RispCloz	0	Major concerns	Low risk	No concerns	Major concerns	No concerns	No concerns	Low	[Within-study bias, Imprecision]
AmiOla:SarcCloz	0	No concerns	Low risk	Some concerns	Major concerns	No concerns	No concerns	Low	[Indirectness, Imprecision]
AmiOla:SerOla	0	Some concerns	Low risk	No concerns	Major concerns	No concerns	No concerns	Low	[Within-study bias, Imprecision]

AmiOla:Sertind	0	Some concerns	Low risk	Some concerns	No concerns	Some concerns	No concerns	Low	[Within-study bias, Indirectness, Heterogeneity]
AmiOla:SertindCloz	0	Some concerns	Low risk	Some concerns	Major concerns	No concerns	No concerns	Low	[Within-study bias, Indirectness, Imprecision]
AmiOla:SerZip	0	No concerns	Low risk	No concerns	Major concerns	No concerns	No concerns	Low	[Imprecision]
AmiOla:Sul	0	Some concerns	Low risk	Some concerns	No concerns	No concerns	No concerns	Moderate	[Within-study bias, Indirectness]
AmiOla:SulCloz	0	Major concerns	Low risk	Some concerns	Major concerns	No concerns	No concerns	Very Low	[Within-study bias, Indirectness, Imprecision]
AmiOla:SulOla	0	Major concerns	Low risk	No concerns	Major concerns	No concerns	No concerns	Low	[Within-study bias, Imprecision]
AmiOla:TopirCloz	0	Major concerns	Low risk	Some concerns	Major concerns	No concerns	No concerns	Very Low	[Within-study bias, Indirectness, Imprecision]
AmiOla:VitD3Cloz	0	No concerns	Low risk	Some concerns	Major concerns	No concerns	No concerns	Low	[Indirectness, Imprecision]
AmiOla:VortRisp	0	No concerns	Low risk	Some concerns	Major concerns	No concerns	No concerns	Low	[Indirectness, Imprecision]
AmiOla:Zip	0	Some concerns	Low risk	Some concerns	Some concerns	Some concerns	No concerns	Low	[Within-study bias, Indirectness, Imprecision, Heterogeneity]
AmiOla:ZipCloz	0	Major concerns	Low risk	No concerns	Major concerns	No concerns	No concerns	Low	[Within-study bias, Imprecision]
AmiOla:Zot	0	Major concerns	Low risk	Some concerns	Major concerns	No concerns	No concerns	Very Low	[Within-study bias, Indirectness, Imprecision]
AriCloz:AriOla	0	No concerns	Low risk	Major concerns	No concerns	Major concerns	No concerns	Low	[Indirectness, Heterogeneity]
AriCloz:BezoaetCloz	0	No concerns	Low risk	No concerns	Major concerns	No concerns	No concerns	Low	[Imprecision]
AriCloz:CelecoxCloz	0	Some concerns	Low risk	Major concerns	Major concerns	No concerns	No concerns	Very Low	[Within-study bias, Indirectness, Imprecision]
AriCloz:CerebrRisp	0	No concerns	Low risk	No concerns	Major concerns	No concerns	No concerns	Low	[Imprecision]

AriCloz:Clot	0	Some concerns	Low risk	No concerns	Major concerns	No concerns	No concerns	Low	[Within-study bias, Imprecision]
AriCloz:Cpz	0	Some concerns	Low risk	No concerns	No concerns	No concerns	No concerns	Moderate	[Within-study bias]
AriCloz:DesmopRisp	0	Major concerns	Low risk	Some concerns	Major concerns	No concerns	No concerns	Very Low	[Within-study bias, Indirectness, Imprecision]
AriCloz:DulCloz	0	No concerns	Low risk	No concerns	No concerns	No concerns	No concerns	High	
AriCloz:Fluph	0	Some concerns	Low risk	No concerns	No concerns	Major concerns	No concerns	Low	[Within-study bias, Heterogeneity]
AriCloz:FluvCloz	0	No concerns	Low risk	No concerns	Major concerns	No concerns	No concerns	Low	[Imprecision]
AriCloz:GinkBilCloz	0	Some concerns	Low risk	No concerns	Major concerns	No concerns	No concerns	Low	[Within-study bias, Imprecision]
AriCloz:GinkBilHal	0	Some concerns	Low risk	No concerns	Major concerns	No concerns	No concerns	Low	[Within-study bias, Imprecision]
AriCloz:GlyCloz	0	Some concerns	Low risk	Some concerns	Major concerns	No concerns	No concerns	Low	[Within-study bias, Indirectness, Imprecision]
AriCloz:Hal	0	Some concerns	Low risk	No concerns	No concerns	Some concerns	No concerns	Moderate	[Within-study bias, Heterogeneity]
AriCloz:LamCloz	0	No concerns	Low risk	No concerns	Major concerns	No concerns	No concerns	Low	[Imprecision]
AriCloz:Lev	0	Some concerns	Low risk	No concerns	Major concerns	No concerns	No concerns	Low	[Within-study bias, Imprecision]
AriCloz:LiHal	0	Some concerns	Low risk	No concerns	Major concerns	No concerns	No concerns	Low	[Within-study bias, Imprecision]
AriCloz:MemCloz	0	No concerns	Low risk	No concerns	Major concerns	No concerns	No concerns	Low	[Imprecision]
AriCloz:MetCloz	0	No concerns	Low risk	Major concerns	Major concerns	No concerns	No concerns	Low	[Indirectness, Imprecision]
AriCloz:MinocCloz	0	No concerns	Low risk	No concerns	Major concerns	No concerns	No concerns	Low	[Imprecision]
AriCloz:MirtCloz	0	Some concerns	Low risk	Some concerns	Major concerns	No concerns	No concerns	Low	[Within-study bias, Indirectness, Imprecision]
AriCloz:MirtRisp	0	Some concerns	Low risk	No concerns	Major concerns	No concerns	No concerns	Low	[Within-study bias, Imprecision]

AriCloz:ModfCloz	0	No concerns	Low risk	Some concerns	Major concerns	No concerns	No concerns	Low	[Indirectness, Imprecision]
AriCloz:Ola	0	Some concerns	Low risk	No concerns	Some concerns	Some concerns	No concerns	Low	[Within-study bias, Imprecision, Heterogeneity]
AriCloz:OlaRisp	0	Some concerns	Low risk	No concerns	Major concerns	No concerns	No concerns	Low	[Within-study bias, Imprecision]
AriCloz:OndastHal	0	Some concerns	Low risk	No concerns	Major concerns	No concerns	No concerns	Low	[Within-study bias, Imprecision]
AriCloz:OxytRisp	0	Major concerns	Low risk	Some concerns	No concerns	No concerns	No concerns	Low	[Within-study bias, Indirectness]
AriCloz:Palip	0	Some concerns	Low risk	No concerns	Major concerns	No concerns	No concerns	Low	[Within-study bias, Imprecision]
AriCloz:Palipla	0	Some concerns	Low risk	No concerns	Major concerns	No concerns	No concerns	Low	[Within-study bias, Imprecision]
AriCloz:PalipOla	0	No concerns	Low risk	Major concerns	Major concerns	No concerns	No concerns	Low	[Indirectness, Imprecision]
AriCloz:PalmitRisp	0	Major concerns	Low risk	No concerns	Major concerns	No concerns	No concerns	Low	[Within-study bias, Imprecision]
AriCloz:Phenylprop Cloz	0	Some concerns	Low risk	Major concerns	Major concerns	No concerns	No concerns	Very Low	[Within-study bias, Indirectness, Imprecision]
AriCloz:PimozCloz	0	No concerns	Low risk	No concerns	Major concerns	No concerns	No concerns	Low	[Imprecision]
AriCloz:PtxRisp	0	Major concerns	Low risk	No concerns	Major concerns	No concerns	No concerns	Low	[Within-study bias, Imprecision]
AriCloz:Que	0	Some concerns	Low risk	No concerns	No concerns	No concerns	No concerns	Moderate	[Within-study bias]
AriCloz:QueCloz	0	Major concerns	Low risk	No concerns	No concerns	Major concerns	No concerns	Low	[Within-study bias, Heterogeneity]
AriCloz:ResverRisp	0	Major concerns	Low risk	No concerns	Major concerns	No concerns	No concerns	Low	[Within-study bias, Imprecision]
AriCloz:Risp	0	Some concerns	Low risk	No concerns	No concerns	Major concerns	No concerns	Low	[Within-study bias, Heterogeneity]
AriCloz:RispCloz	0	No concerns	Low risk	No concerns	Major concerns	No concerns	No concerns	Low	[Imprecision]

AriCloz:SarcCloz	0	No concerns	Low risk	No concerns	Major concerns	No concerns	No concerns	Low	[Imprecision]
AriCloz:SerOla	0	Some concerns	Low risk	No concerns	Major concerns	No concerns	No concerns	Low	[Within-study bias, Imprecision]
AriCloz:Sertind	0	Some concerns	Low risk	No concerns	No concerns	Major concerns	No concerns	Low	[Within-study bias, Heterogeneity]
AriCloz:SertindCloz	0	Some concerns	Low risk	No concerns	Major concerns	No concerns	No concerns	Low	[Within-study bias, Imprecision]
AriCloz:SerZip	0	No concerns	Low risk	No concerns	Major concerns	No concerns	No concerns	Low	[Imprecision]
AriCloz:Sul	0	Some concerns	Low risk	No concerns	No concerns	No concerns	No concerns	Moderate	[Within-study bias]
AriCloz:SulCloz	0	Major concerns	Low risk	No concerns	Major concerns	No concerns	No concerns	Low	[Within-study bias, Imprecision]
AriCloz:SulOla	0	Some concerns	Low risk	No concerns	Major concerns	No concerns	No concerns	Low	[Within-study bias, Imprecision]
AriCloz:TopirCloz	0	Some concerns	Low risk	No concerns	Major concerns	No concerns	No concerns	Low	[Within-study bias, Imprecision]
AriCloz:VitD3Cloz	0	No concerns	Low risk	Some concerns	Major concerns	No concerns	No concerns	Low	[Indirectness, Imprecision]
AriCloz:VortRisp	0	No concerns	Low risk	No concerns	Major concerns	No concerns	No concerns	Low	[Imprecision]
AriCloz:Zip	0	Some concerns	Low risk	No concerns	No concerns	Major concerns	No concerns	Low	[Within-study bias, Heterogeneity]
AriCloz:ZipCloz	0	No concerns	Low risk	No concerns	Major concerns	No concerns	No concerns	Low	[Imprecision]
AriCloz:Zot	0	Major concerns	Low risk	Some concerns	Major concerns	No concerns	No concerns	Very Low	[Within-study bias, Indirectness, Imprecision]
AriOla:BezoaetCloz	0	No concerns	Low risk	No concerns	Major concerns	No concerns	No concerns	Low	[Imprecision]
AriOla:CelecoxCloz	0	Some concerns	Low risk	Major concerns	Major concerns	No concerns	No concerns	Very Low	[Within-study bias, Indirectness, Imprecision]
AriOla:CerebrRisp	0	No concerns	Low risk	Major concerns	No concerns	No concerns	No concerns	Low	[Indirectness]

AriOla:Clot	0	No concerns	Low risk	No concerns	Major concerns	No concerns	No concerns	Low	[Imprecision]
AriOla:Cloz	0	No concerns	Low risk	Major concerns	No concerns	No concerns	No concerns	Low	[Indirectness]
AriOla:Cpz	0	No concerns	Low risk	Major concerns	No concerns	No concerns	No concerns	Low	[Indirectness]
AriOla:DesmopRisp	0	Major concerns	Low risk	Some concerns	Major concerns	No concerns	No concerns	Very Low	[Within-study bias, Indirectness, Imprecision]
AriOla:DulCloz	0	No concerns	Low risk	No concerns	Major concerns	No concerns	No concerns	Low	[Imprecision]
AriOla:Fluph	0	Some concerns	Low risk	No concerns	No concerns	No concerns	No concerns	Moderate	[Within-study bias]
AriOla:FluvCloz	0	No concerns	Low risk	No concerns	No concerns	No concerns	No concerns	High	
AriOla:GinkBilCloz	0	Some concerns	Low risk	No concerns	Major concerns	No concerns	No concerns	Low	[Within-study bias, Imprecision]
AriOla:GinkBilHal	0	Some concerns	Low risk	No concerns	No concerns	Some concerns	No concerns	Moderate	[Within-study bias, Heterogeneity]
AriOla:GlyCloz	0	Some concerns	Low risk	Some concerns	Major concerns	No concerns	No concerns	Low	[Within-study bias, Indirectness, Imprecision]
AriOla:Hal	0	No concerns	Low risk	Major concerns	No concerns	No concerns	No concerns	Low	[Indirectness]
AriOla:HalCloz	0	No concerns	Low risk	No concerns	Major concerns	No concerns	No concerns	Low	[Imprecision]
AriOla:LamCloz	0	No concerns	Low risk	No concerns	Major concerns	No concerns	No concerns	Low	[Imprecision]
AriOla:Lev	0	No concerns	Low risk	No concerns	Major concerns	No concerns	No concerns	Low	[Imprecision]
AriOla:LiHal	0	No concerns	Low risk	Major concerns	Major concerns	No concerns	No concerns	Low	[Indirectness, Imprecision]
AriOla:MemCloz	0	No concerns	Low risk	Major concerns	Major concerns	No concerns	No concerns	Low	[Indirectness, Imprecision]
AriOla:MetCloz	0	No concerns	Low risk	Major concerns	Major concerns	No concerns	No concerns	Low	[Indirectness, Imprecision]
AriOla:MinocCloz	0	No concerns	Low risk	No concerns	Major concerns	No concerns	No concerns	Low	[Imprecision]

AriOla:MirtCloz	0	Some concerns	Low risk	Some concerns	Major concerns	No concerns	No concerns	Low	[Within-study bias, Indirectness, Imprecision]
AriOla:MirtRisp	0	Some concerns	Low risk	Major concerns	Major concerns	No concerns	No concerns	Very Low	[Within-study bias, Indirectness, Imprecision]
AriOla:ModfCloz	0	No concerns	Low risk	Some concerns	Major concerns	No concerns	No concerns	Low	[Indirectness, Imprecision]
AriOla:OlaRisp	0	No concerns	Low risk	Some concerns	Some concerns	Some concerns	No concerns	Low	[Indirectness, Imprecision, Heterogeneity]
AriOla:OndastHal	0	No concerns	Low risk	No concerns	Some concerns	Some concerns	No concerns	Moderate	[Imprecision, Heterogeneity]
AriOla:OxytRisp	0	Major concerns	Low risk	Some concerns	Major concerns	No concerns	No concerns	Very Low	[Within-study bias, Indirectness, Imprecision]
AriOla:Palip	0	No concerns	Low risk	Major concerns	No concerns	Some concerns	No concerns	Low	[Indirectness, Heterogeneity]
AriOla:Palipla	0	No concerns	Low risk	Major concerns	No concerns	No concerns	No concerns	Low	[Indirectness]
AriOla:PalmitRisp	0	Major concerns	Low risk	Major concerns	Major concerns	No concerns	No concerns	Very Low	[Within-study bias, Indirectness, Imprecision]
AriOla:PhenylpropCloz	0	Some concerns	Low risk	Major concerns	Major concerns	No concerns	No concerns	Very Low	[Within-study bias, Indirectness, Imprecision]
AriOla:PimozCloz	0	No concerns	Low risk	No concerns	No concerns	No concerns	No concerns	High	
AriOla:PtxRisp	0	Major concerns	Low risk	Major concerns	Major concerns	No concerns	No concerns	Very Low	[Within-study bias, Indirectness, Imprecision]
AriOla:Que	0	No concerns	Low risk	Major concerns	No concerns	No concerns	No concerns	Low	[Indirectness]
AriOla:QueCloz	0	No concerns	Low risk	No concerns	No concerns	No concerns	No concerns	High	
AriOla:ResverRisp	0	Major concerns	Low risk	Major concerns	Major concerns	No concerns	No concerns	Very Low	[Within-study bias, Indirectness, Imprecision]
AriOla:Risp	0	No concerns	Low risk	Major concerns	No concerns	No concerns	No concerns	Low	[Indirectness]
AriOla:RispCloz	0	No concerns	Low risk	No concerns	Some concerns	Some concerns	No concerns	Moderate	[Imprecision, Heterogeneity]
AriOla:SarcCloz	0	No concerns	Low risk	No concerns	No concerns	Some concerns	No concerns	Moderate	[Heterogeneity]
AriOla:SerOla	0	No concerns	Low risk	Major concerns	No concerns	Some concerns	No concerns	Low	[Indirectness, Heterogeneity]

AriOla:Sertind	0	Some concerns	Low risk	No concerns	No concerns	No concerns	No concerns	Moderate	[Within-study bias]
AriOla:SertindCloz	0	Some concerns	Low risk	No concerns	Some concerns	Some concerns	No concerns	Low	[Within-study bias, Imprecision, Heterogeneity]
AriOla:SerZip	0	No concerns	Low risk	No concerns	Major concerns	No concerns	No concerns	Low	[Imprecision]
AriOla:Sul	0	Some concerns	Low risk	No concerns	No concerns	No concerns	No concerns	Moderate	[Within-study bias]
AriOla:SulCloz	0	No concerns	Low risk	No concerns	Major concerns	No concerns	No concerns	Low	[Imprecision]
AriOla:SulOla	0	No concerns	Low risk	Major concerns	Major concerns	No concerns	No concerns	Low	[Indirectness, Imprecision]
AriOla:TopirCloz	0	No concerns	Low risk	No concerns	Major concerns	No concerns	No concerns	Low	[Imprecision]
AriOla:VitD3Cloz	0	No concerns	Low risk	Some concerns	No concerns	No concerns	No concerns	Moderate	[Indirectness]
AriOla:VortRisp	0	No concerns	Low risk	Major concerns	Major concerns	No concerns	No concerns	Low	[Indirectness, Imprecision]
AriOla:Zip	0	No concerns	Low risk	Major concerns	No concerns	No concerns	No concerns	Low	[Indirectness]
AriOla:ZipCloz	0	No concerns	Low risk	No concerns	Major concerns	No concerns	No concerns	Low	[Imprecision]
AriOla:Zot	0	No concerns	Low risk	Some concerns	No concerns	No concerns	No concerns	Moderate	[Indirectness]
BezoaetCloz:CelecoxCloz	0	No concerns	Low risk	No concerns	Major concerns	No concerns	No concerns	Low	[Imprecision]
BezoaetCloz:CerebrRisp	0	No concerns	Low risk	No concerns	Major concerns	No concerns	No concerns	Low	[Imprecision]
BezoaetCloz:Clot	0	No concerns	Low risk	No concerns	Major concerns	No concerns	No concerns	Low	[Imprecision]
BezoaetCloz:Cpz	0	No concerns	Low risk	No concerns	No concerns	No concerns	No concerns	High	
BezoaetCloz:DesmopRisp	0	Major concerns	Low risk	No concerns	Major concerns	No concerns	No concerns	Low	[Within-study bias, Imprecision]

BezoaetCloz:DulCloz	0	No concerns	Low risk	No concerns	Major concerns	No concerns	No concerns	Low	[Imprecision]
BezoaetCloz:Fluph	0	Some concerns	Low risk	No concerns	No concerns	Some concerns	No concerns	Moderate	[Within-study bias, Heterogeneity]
BezoaetCloz:FluvCloz	0	No concerns	Low risk	No concerns	Major concerns	No concerns	No concerns	Low	[Imprecision]
BezoaetCloz:GinkBilCloz	0	No concerns	Low risk	No concerns	Major concerns	No concerns	No concerns	Low	[Imprecision]
BezoaetCloz:GinkBilHal	0	Some concerns	Low risk	No concerns	Major concerns	No concerns	No concerns	Low	[Within-study bias, Imprecision]
BezoaetCloz:GlyCloz	0	No concerns	Low risk	No concerns	Major concerns	No concerns	No concerns	Low	[Imprecision]
BezoaetCloz:Hal	0	No concerns	Low risk	No concerns	No concerns	Some concerns	No concerns	Moderate	[Heterogeneity]
BezoaetCloz:HalCloz	0	No concerns	Low risk	No concerns	Major concerns	No concerns	No concerns	Low	[Imprecision]
BezoaetCloz:LamCloz	0	No concerns	Low risk	No concerns	Major concerns	No concerns	No concerns	Low	[Imprecision]
BezoaetCloz:Lev	0	No concerns	Low risk	No concerns	Major concerns	No concerns	No concerns	Low	[Imprecision]
BezoaetCloz:LiHal	0	No concerns	Low risk	No concerns	Major concerns	No concerns	No concerns	Low	[Imprecision]
BezoaetCloz:MemCloz	0	No concerns	Low risk	No concerns	Major concerns	No concerns	No concerns	Low	[Imprecision]
BezoaetCloz:MetCloz	0	No concerns	Low risk	No concerns	Major concerns	No concerns	No concerns	Low	[Imprecision]
BezoaetCloz:MinocCloz	0	No concerns	Low risk	No concerns	Major concerns	No concerns	No concerns	Low	[Imprecision]
BezoaetCloz:MirtCloz	0	No concerns	Low risk	No concerns	Major concerns	No concerns	No concerns	Low	[Imprecision]
BezoaetCloz:MirtRisp	0	Some concerns	Low risk	No concerns	Major concerns	No concerns	No concerns	Low	[Within-study bias, Imprecision]
BezoaetCloz:ModfCloz	0	No concerns	Low risk	No concerns	Major concerns	No concerns	No concerns	Low	[Imprecision]
BezoaetCloz:Ola	0	No concerns	Low risk	No concerns	Major concerns	No concerns	No concerns	Low	[Imprecision]

BezoaetCloz:OlaRisp	0	Some concerns	Low risk	No concerns	Major concerns	No concerns	No concerns	Low	[Within-study bias, Imprecision]
BezoaetCloz:OndastHal	0	No concerns	Low risk	No concerns	Major concerns	No concerns	No concerns	Low	[Imprecision]
BezoaetCloz:OxytRisp	0	Major concerns	Low risk	No concerns	Major concerns	No concerns	No concerns	Low	[Within-study bias, Imprecision]
BezoaetCloz:Palip	0	Some concerns	Low risk	No concerns	Major concerns	No concerns	No concerns	Low	[Within-study bias, Imprecision]
BezoaetCloz:Palipla	0	Some concerns	Low risk	No concerns	Major concerns	No concerns	No concerns	Low	[Within-study bias, Imprecision]
BezoaetCloz:PalipOla	0	No concerns	Low risk	No concerns	Major concerns	No concerns	No concerns	Low	[Imprecision]
BezoaetCloz:PalmitRisp	0	Major concerns	Low risk	No concerns	Major concerns	No concerns	No concerns	Low	[Within-study bias, Imprecision]
BezoaetCloz:PhenylpropCloz	0	No concerns	Low risk	No concerns	Major concerns	No concerns	No concerns	Low	[Imprecision]
BezoaetCloz:PimozCloz	0	No concerns	Low risk	No concerns	Major concerns	No concerns	No concerns	Low	[Imprecision]
BezoaetCloz:PtxRisp	0	Major concerns	Low risk	No concerns	Major concerns	No concerns	No concerns	Low	[Within-study bias, Imprecision]
BezoaetCloz:Que	0	No concerns	Low risk	No concerns	No concerns	No concerns	No concerns	High	
BezoaetCloz:QueCloz	0	Major concerns	Low risk	No concerns	No concerns	No concerns	No concerns	Low	[Within-study bias]
BezoaetCloz:ResverRisp	0	Major concerns	Low risk	No concerns	Major concerns	No concerns	No concerns	Low	[Within-study bias, Imprecision]
BezoaetCloz:Risp	0	No concerns	Low risk	No concerns	No concerns	Major concerns	No concerns	Low	[Heterogeneity]
BezoaetCloz:RispCloz	0	No concerns	Low risk	No concerns	Major concerns	No concerns	No concerns	Low	[Imprecision]
BezoaetCloz:SarcCloz	0	No concerns	Low risk	No concerns	Major concerns	No concerns	No concerns	Low	[Imprecision]
BezoaetCloz:SerOla	0	Some concerns	Low risk	No concerns	Major concerns	No concerns	No concerns	Low	[Within-study bias, Imprecision]
BezoaetCloz:Sertind	0	Some concerns	Low risk	No concerns	No concerns	Some concerns	No concerns	Moderate	[Within-study bias, Heterogeneity]

BezoaetCloz:SertindCloz	0	No concerns	Low risk	No concerns	Major concerns	No concerns	No concerns	Low	[Imprecision]
BezoaetCloz:SerZip	0	No concerns	Low risk	No concerns	Major concerns	No concerns	No concerns	Low	[Imprecision]
BezoaetCloz:Sul	0	No concerns	Low risk	No concerns	No concerns	No concerns	No concerns	High	
BezoaetCloz:SulCloz	0	No concerns	Low risk	No concerns	Major concerns	No concerns	No concerns	Low	[Imprecision]
BezoaetCloz:SulOla	0	No concerns	Low risk	No concerns	Major concerns	No concerns	No concerns	Low	[Imprecision]
BezoaetCloz:TopirCloz	0	No concerns	Low risk	No concerns	Major concerns	No concerns	No concerns	Low	[Imprecision]
BezoaetCloz:VitD3Cloz	0	No concerns	Low risk	No concerns	Major concerns	No concerns	No concerns	Low	[Imprecision]
BezoaetCloz:VortRisp	0	No concerns	Low risk	No concerns	Major concerns	No concerns	No concerns	Low	[Imprecision]
BezoaetCloz:Zip	0	No concerns	Low risk	No concerns	No concerns	Major concerns	No concerns	Low	[Heterogeneity]
BezoaetCloz:ZipCloz	0	No concerns	Low risk	No concerns	Major concerns	No concerns	No concerns	Low	[Imprecision]
BezoaetCloz:Zot	0	No concerns	Low risk	No concerns	Major concerns	No concerns	No concerns	Low	[Imprecision]
CelecoxCloz:CerebrRisp	0	Some concerns	Low risk	Major concerns	Major concerns	No concerns	No concerns	Very Low	[Within-study bias, Indirectness, Imprecision]
CelecoxCloz:Clot	0	Some concerns	Low risk	No concerns	Major concerns	No concerns	No concerns	Low	[Within-study bias, Imprecision]
CelecoxCloz:Cpz	0	Some concerns	Low risk	No concerns	Major concerns	No concerns	No concerns	Low	[Within-study bias, Imprecision]
CelecoxCloz:DesmopRisp	0	Major concerns	Low risk	Some concerns	Major concerns	No concerns	No concerns	Very Low	[Within-study bias, Indirectness, Imprecision]
CelecoxCloz:DulCloz	0	No concerns	Low risk	No concerns	No concerns	No concerns	No concerns	High	
CelecoxCloz:Fluph	0	Some concerns	Low risk	No concerns	Major concerns	No concerns	No concerns	Low	[Within-study bias, Imprecision]
CelecoxCloz:FluvCloz	0	No concerns	Low risk	No concerns	Major concerns	No concerns	No concerns	Low	[Imprecision]

CelecoxCloz:GinkBilCloz	0	Some concerns	Low risk	No concerns	Major concerns	No concerns	No concerns	Low	[Within-study bias, Imprecision]
CelecoxCloz:GinkBilHal	0	Some concerns	Low risk	No concerns	Major concerns	No concerns	No concerns	Low	[Within-study bias, Imprecision]
CelecoxCloz:GlyCloz	0	Some concerns	Low risk	Some concerns	Major concerns	No concerns	No concerns	Low	[Within-study bias, Indirectness, Imprecision]
CelecoxCloz:Hal	0	Some concerns	Low risk	Major concerns	Major concerns	No concerns	No concerns	Very Low	[Within-study bias, Indirectness, Imprecision]
CelecoxCloz:HalCloz	0	Some concerns	Low risk	No concerns	Major concerns	No concerns	No concerns	Low	[Within-study bias, Imprecision]
CelecoxCloz:LamCloz	0	Some concerns	Low risk	Major concerns	Major concerns	No concerns	No concerns	Very Low	[Within-study bias, Indirectness, Imprecision]
CelecoxCloz:Lev	0	Some concerns	Low risk	No concerns	Major concerns	No concerns	No concerns	Low	[Within-study bias, Imprecision]
CelecoxCloz:LiHal	0	Some concerns	Low risk	Major concerns	Major concerns	No concerns	No concerns	Very Low	[Within-study bias, Indirectness, Imprecision]
CelecoxCloz:MemCloz	0	No concerns	Low risk	Major concerns	Major concerns	No concerns	No concerns	Low	[Indirectness, Imprecision]
CelecoxCloz:MetCloz	0	Some concerns	Low risk	Major concerns	Major concerns	No concerns	No concerns	Very Low	[Within-study bias, Indirectness, Imprecision]
CelecoxCloz:MinocCloz	0	No concerns	Low risk	No concerns	Major concerns	No concerns	No concerns	Low	[Imprecision]
CelecoxCloz:MirtCloz	0	Some concerns	Low risk	Some concerns	Major concerns	No concerns	No concerns	Low	[Within-study bias, Indirectness, Imprecision]
CelecoxCloz:MirtRisp	0	Some concerns	Low risk	Major concerns	Major concerns	No concerns	No concerns	Very Low	[Within-study bias, Indirectness, Imprecision]
CelecoxCloz:ModfCloz	0	No concerns	Low risk	Some concerns	Major concerns	No concerns	No concerns	Low	[Indirectness, Imprecision]
CelecoxCloz:Ola	0	Some concerns	Low risk	Major concerns	Major concerns	No concerns	No concerns	Very Low	[Within-study bias, Indirectness, Imprecision]
CelecoxCloz:OlaRisp	0	Some concerns	Low risk	Some concerns	Major concerns	No concerns	No concerns	Low	[Within-study bias, Indirectness, Imprecision]
CelecoxCloz:OndastHal	0	Some concerns	Low risk	No concerns	Major concerns	No concerns	No concerns	Low	[Within-study bias, Imprecision]

CelecoxCloz:OxytRisp	0	Major concerns	Low risk	Some concerns	No concerns	No concerns	No concerns	Low	[Within-study bias, Indirectness]
CelecoxCloz:Palip	0	Some concerns	Low risk	No concerns	Major concerns	No concerns	No concerns	Low	[Within-study bias, Imprecision]
CelecoxCloz:Palipla	0	Some concerns	Low risk	Some concerns	Major concerns	No concerns	No concerns	Low	[Within-study bias, Indirectness, Imprecision]
CelecoxCloz:PalipOla	0	Some concerns	Low risk	Major concerns	Major concerns	No concerns	No concerns	Very Low	[Within-study bias, Indirectness, Imprecision]
CelecoxCloz:PalmitRisp	0	Major concerns	Low risk	Major concerns	Major concerns	No concerns	No concerns	Very Low	[Within-study bias, Indirectness, Imprecision]
CelecoxCloz:PhenylpropCloz	0	Some concerns	Low risk	Major concerns	Major concerns	No concerns	No concerns	Very Low	[Within-study bias, Indirectness, Imprecision]
CelecoxCloz:PimozCloz	0	No concerns	Low risk	No concerns	Major concerns	No concerns	No concerns	Low	[Imprecision]
CelecoxCloz:PtxRisp	0	Major concerns	Low risk	Major concerns	Major concerns	No concerns	No concerns	Very Low	[Within-study bias, Indirectness, Imprecision]
CelecoxCloz:Que	0	Some concerns	Low risk	No concerns	Major concerns	No concerns	No concerns	Low	[Within-study bias, Imprecision]
CelecoxCloz:QueCloz	0	Some concerns	Low risk	No concerns	Major concerns	No concerns	No concerns	Low	[Within-study bias, Imprecision]
CelecoxCloz:ResverRisp	0	Major concerns	Low risk	Major concerns	Major concerns	No concerns	No concerns	Very Low	[Within-study bias, Indirectness, Imprecision]
CelecoxCloz:Risp	0	Some concerns	Low risk	Major concerns	Major concerns	No concerns	No concerns	Very Low	[Within-study bias, Indirectness, Imprecision]
CelecoxCloz:RispCloz	0	Some concerns	Low risk	No concerns	Major concerns	No concerns	No concerns	Low	[Within-study bias, Imprecision]
CelecoxCloz:SarcCloz	0	No concerns	Low risk	No concerns	Major concerns	No concerns	No concerns	Low	[Imprecision]
CelecoxCloz:SerOla	0	Some concerns	Low risk	No concerns	Major concerns	No concerns	No concerns	Low	[Within-study bias, Imprecision]
CelecoxCloz:Sertind	0	Some concerns	Low risk	No concerns	Major concerns	No concerns	No concerns	Low	[Within-study bias, Imprecision]
CelecoxCloz:SertindCloz	0	Some concerns	Low risk	No concerns	Major concerns	No concerns	No concerns	Low	[Within-study bias, Imprecision]

CelecoxCloz:SerZip	0	Some concerns	Low risk	No concerns	Major concerns	No concerns	No concerns	Low	[Within-study bias, Imprecision]
CelecoxCloz:Sul	0	Some concerns	Low risk	No concerns	Some concerns	Some concerns	No concerns	Low	[Within-study bias, Imprecision, Heterogeneity]
CelecoxCloz:SulCloz	0	Some concerns	Low risk	No concerns	Major concerns	No concerns	No concerns	Low	[Within-study bias, Imprecision]
CelecoxCloz:SulOla	0	Some concerns	Low risk	No concerns	Major concerns	No concerns	No concerns	Low	[Within-study bias, Imprecision]
CelecoxCloz:TopirCloz	0	Some concerns	Low risk	No concerns	Major concerns	No concerns	No concerns	Low	[Within-study bias, Imprecision]
CelecoxCloz:VitD3Cloz	0	No concerns	Low risk	Some concerns	Major concerns	No concerns	No concerns	Low	[Indirectness, Imprecision]
CelecoxCloz:VortRisp	0	Some concerns	Low risk	Major concerns	Major concerns	No concerns	No concerns	Very Low	[Within-study bias, Indirectness, Imprecision]
CelecoxCloz:Zip	0	Some concerns	Low risk	Major concerns	Major concerns	No concerns	No concerns	Very Low	[Within-study bias, Indirectness, Imprecision]
CelecoxCloz:ZipCloz	0	Some concerns	Low risk	No concerns	Major concerns	No concerns	No concerns	Low	[Within-study bias, Imprecision]
CelecoxCloz:Zot	0	Some concerns	Low risk	Some concerns	Major concerns	No concerns	No concerns	Low	[Within-study bias, Indirectness, Imprecision]
CerebrRisp:Clot	0	Some concerns	Low risk	No concerns	Major concerns	No concerns	No concerns	Low	[Within-study bias, Imprecision]
CerebrRisp:Cloz	0	No concerns	Low risk	Major concerns	Major concerns	No concerns	No concerns	Low	[Indirectness, Imprecision]
CerebrRisp:Cpz	0	Some concerns	Low risk	No concerns	Major concerns	No concerns	No concerns	Low	[Within-study bias, Imprecision]
CerebrRisp:DesmopRisp	0	Major concerns	Low risk	Some concerns	Major concerns	No concerns	No concerns	Very Low	[Within-study bias, Indirectness, Imprecision]
CerebrRisp:DulCloz	0	No concerns	Low risk	No concerns	No concerns	No concerns	No concerns	High	
CerebrRisp:Fluph	0	Some concerns	Low risk	No concerns	Major concerns	No concerns	No concerns	Low	[Within-study bias, Imprecision]
CerebrRisp:FluvCloz	0	No concerns	Low risk	No concerns	Major concerns	No concerns	No concerns	Low	[Imprecision]

CerebrRisp:GinkBil Cloz	0	Some concerns	Low risk	No concerns	Major concerns	No concerns	No concerns	Low	[Within-study bias, Imprecision]
CerebrRisp:GinkBil Hal	0	Some concerns	Low risk	No concerns	Major concerns	No concerns	No concerns	Low	[Within-study bias, Imprecision]
CerebrRisp:GlyCloz	0	Some concerns	Low risk	Some concerns	Major concerns	No concerns	No concerns	Low	[Within-study bias, Indirectness, Imprecision]
CerebrRisp:Hal	0	No concerns	Low risk	Major concerns	Major concerns	No concerns	No concerns	Low	[Indirectness, Imprecision]
CerebrRisp:HalCloz	0	Some concerns	Low risk	No concerns	Major concerns	No concerns	No concerns	Low	[Within-study bias, Imprecision]
CerebrRisp:LamClo z	0	No concerns	Low risk	No concerns	Major concerns	No concerns	No concerns	Low	[Imprecision]
CerebrRisp:Lev	0	No concerns	Low risk	No concerns	Major concerns	No concerns	No concerns	Low	[Imprecision]
CerebrRisp:LiHal	0	No concerns	Low risk	Major concerns	Major concerns	No concerns	No concerns	Low	[Indirectness, Imprecision]
CerebrRisp:MemClo z	0	No concerns	Low risk	No concerns	Major concerns	No concerns	No concerns	Low	[Imprecision]
CerebrRisp:MetCloz	0	No concerns	Low risk	Major concerns	Major concerns	No concerns	No concerns	Low	[Indirectness, Imprecision]
CerebrRisp:MinocCl oz	0	No concerns	Low risk	No concerns	Major concerns	No concerns	No concerns	Low	[Imprecision]
CerebrRisp:MirtCloz	0	Some concerns	Low risk	Some concerns	No concerns	Some concerns	No concerns	Low	[Within-study bias, Indirectness, Heterogeneity]
CerebrRisp:MirtRisp	0	No concerns	Low risk	Major concerns	Major concerns	No concerns	No concerns	Low	[Indirectness, Imprecision]
CerebrRisp:ModfClo z	0	No concerns	Low risk	Some concerns	Major concerns	No concerns	No concerns	Low	[Indirectness, Imprecision]
CerebrRisp:Ola	0	No concerns	Low risk	Major concerns	Major concerns	No concerns	No concerns	Low	[Indirectness, Imprecision]
CerebrRisp:OlaRisp	0	Some concerns	Low risk	Some concerns	Major concerns	No concerns	No concerns	Low	[Within-study bias, Indirectness, Imprecision]
CerebrRisp:OndastH al	0	No concerns	Low risk	No concerns	Major concerns	No concerns	No concerns	Low	[Imprecision]

CerebrRisp:OxytRisp	0	Major concerns	Low risk	Some concerns	No concerns	No concerns	No concerns	Low	[Within-study bias, Indirectness]
CerebrRisp:Palip	0	Some concerns	Low risk	No concerns	Major concerns	No concerns	No concerns	Low	[Within-study bias, Imprecision]
CerebrRisp:Palipla	0	Some concerns	Low risk	Some concerns	Major concerns	No concerns	No concerns	Low	[Within-study bias, Indirectness, Imprecision]
CerebrRisp:PalipOla	0	No concerns	Low risk	Major concerns	No concerns	Some concerns	No concerns	Low	[Indirectness, Heterogeneity]
CerebrRisp:PalmitRisp	0	Major concerns	Low risk	Major concerns	Major concerns	No concerns	No concerns	Very Low	[Within-study bias, Indirectness, Imprecision]
CerebrRisp:PhenylpropCloz	0	Some concerns	Low risk	Major concerns	Major concerns	No concerns	No concerns	Very Low	[Within-study bias, Indirectness, Imprecision]
CerebrRisp:PimozCloz	0	No concerns	Low risk	No concerns	Major concerns	No concerns	No concerns	Low	[Imprecision]
CerebrRisp:PtxRisp	0	Major concerns	Low risk	Major concerns	Major concerns	No concerns	No concerns	Very Low	[Within-study bias, Indirectness, Imprecision]
CerebrRisp:Que	0	No concerns	Low risk	No concerns	Major concerns	No concerns	No concerns	Low	[Imprecision]
CerebrRisp:QueCloz	0	Major concerns	Low risk	No concerns	Major concerns	No concerns	No concerns	Low	[Within-study bias, Imprecision]
CerebrRisp:ResverRisp	0	Major concerns	Low risk	Major concerns	Major concerns	No concerns	No concerns	Very Low	[Within-study bias, Indirectness, Imprecision]
CerebrRisp:RispCloz	0	No concerns	Low risk	No concerns	Major concerns	No concerns	No concerns	Low	[Imprecision]
CerebrRisp:SarcCloz	0	No concerns	Low risk	No concerns	Major concerns	No concerns	No concerns	Low	[Imprecision]
CerebrRisp:SerOla	0	Some concerns	Low risk	No concerns	Major concerns	No concerns	No concerns	Low	[Within-study bias, Imprecision]
CerebrRisp:Sertind	0	No concerns	Low risk	No concerns	Major concerns	No concerns	No concerns	Low	[Imprecision]
CerebrRisp:SertindCloz	0	Some concerns	Low risk	No concerns	Major concerns	No concerns	No concerns	Low	[Within-study bias, Imprecision]
CerebrRisp:SerZip	0	No concerns	Low risk	No concerns	Major concerns	No concerns	No concerns	Low	[Imprecision]

CerebrRisp:Sul	0	Some concerns	Low risk	No concerns	Major concerns	No concerns	No concerns	Low	[Within-study bias, Imprecision]
CerebrRisp:SulCloz	0	No concerns	Low risk	No concerns	Major concerns	No concerns	No concerns	Low	[Imprecision]
CerebrRisp:SulOla	0	No concerns	Low risk	No concerns	Major concerns	No concerns	No concerns	Low	[Imprecision]
CerebrRisp:TopirCloz	0	No concerns	Low risk	No concerns	Major concerns	No concerns	No concerns	Low	[Imprecision]
CerebrRisp:VitD3Cloz	0	No concerns	Low risk	Some concerns	Major concerns	No concerns	No concerns	Low	[Indirectness, Imprecision]
CerebrRisp:VortRisp	0	No concerns	Low risk	Major concerns	Major concerns	No concerns	No concerns	Low	[Indirectness, Imprecision]
CerebrRisp:Zip	0	Some concerns	Low risk	No concerns	Major concerns	No concerns	No concerns	Low	[Within-study bias, Imprecision]
CerebrRisp:ZipCloz	0	No concerns	Low risk	No concerns	No concerns	Major concerns	No concerns	Low	[Heterogeneity]
CerebrRisp:Zot	0	No concerns	Low risk	Some concerns	Major concerns	No concerns	No concerns	Low	[Indirectness, Imprecision]
Clot:Cloz	0	Some concerns	Low risk	No concerns	Major concerns	No concerns	No concerns	Low	[Within-study bias, Imprecision]
Clot:DesmopRisp	0	Major concerns	Low risk	No concerns	Major concerns	No concerns	No concerns	Low	[Within-study bias, Imprecision]
Clot:DulCloz	0	No concerns	Low risk	No concerns	Some concerns	Some concerns	No concerns	Moderate	[Imprecision, Heterogeneity]
Clot:Fluph	0	Some concerns	Low risk	No concerns	Major concerns	No concerns	No concerns	Low	[Within-study bias, Imprecision]
Clot:FluvCloz	0	No concerns	Low risk	No concerns	Major concerns	No concerns	No concerns	Low	[Imprecision]
Clot:GinkBilCloz	0	Some concerns	Low risk	No concerns	Major concerns	No concerns	No concerns	Low	[Within-study bias, Imprecision]
Clot:GinkBilHal	0	Some concerns	Low risk	No concerns	Major concerns	No concerns	No concerns	Low	[Within-study bias, Imprecision]
Clot:GlyCloz	0	Some concerns	Low risk	No concerns	Major concerns	No concerns	No concerns	Low	[Within-study bias, Imprecision]
Clot:Hal	0	Some concerns	Low risk	No concerns	Major concerns	No concerns	No concerns	Low	[Within-study bias, Imprecision]

Clot:HalCloz	0	Major concerns	Low risk	No concerns	Major concerns	No concerns	No concerns	Low	[Within-study bias, Imprecision]
Clot:LamCloz	0	Some concerns	Low risk	No concerns	Major concerns	No concerns	No concerns	Low	[Within-study bias, Imprecision]
Clot:Lev	0	No concerns	Low risk	No concerns	Major concerns	No concerns	No concerns	Low	[Imprecision]
Clot:LiHal	0	Some concerns	Low risk	No concerns	Major concerns	No concerns	No concerns	Low	[Within-study bias, Imprecision]
Clot:MemCloz	0	No concerns	Low risk	No concerns	Major concerns	No concerns	No concerns	Low	[Imprecision]
Clot:MetCloz	0	Some concerns	Low risk	No concerns	Major concerns	No concerns	No concerns	Low	[Within-study bias, Imprecision]
Clot:MinocCloz	0	No concerns	Low risk	No concerns	Major concerns	No concerns	No concerns	Low	[Imprecision]
Clot:MirtCloz	0	Some concerns	Low risk	No concerns	Major concerns	No concerns	No concerns	Low	[Within-study bias, Imprecision]
Clot:MirtRisp	0	Some concerns	Low risk	No concerns	Major concerns	No concerns	No concerns	Low	[Within-study bias, Imprecision]
Clot:ModfCloz	0	No concerns	Low risk	No concerns	Major concerns	No concerns	No concerns	Low	[Imprecision]
Clot:Ola	0	Some concerns	Low risk	No concerns	Major concerns	No concerns	No concerns	Low	[Within-study bias, Imprecision]
Clot:OlaRisp	0	Some concerns	Low risk	No concerns	Major concerns	No concerns	No concerns	Low	[Within-study bias, Imprecision]
Clot:OndastHal	0	Some concerns	Low risk	No concerns	Major concerns	No concerns	No concerns	Low	[Within-study bias, Imprecision]
Clot:OxytRisp	0	Major concerns	Low risk	No concerns	No concerns	Some concerns	No concerns	Low	[Within-study bias, Heterogeneity]
Clot:Palip	0	Some concerns	Low risk	No concerns	Major concerns	No concerns	No concerns	Low	[Within-study bias, Imprecision]
Clot:Palipla	0	Some concerns	Low risk	No concerns	Major concerns	No concerns	No concerns	Low	[Within-study bias, Imprecision]
Clot:PalipOla	0	No concerns	Low risk	No concerns	Major concerns	No concerns	No concerns	Low	[Imprecision]

Clot:PalmitRisp	0	Major concerns	Low risk	No concerns	Major concerns	No concerns	No concerns	Low	[Within-study bias, Imprecision]
Clot:PhenylpropCloz	0	Some concerns	Low risk	No concerns	Major concerns	No concerns	No concerns	Low	[Within-study bias, Imprecision]
Clot:PimozCloz	0	No concerns	Low risk	No concerns	Major concerns	No concerns	No concerns	Low	[Imprecision]
Clot:PtxRisp	0	Major concerns	Low risk	No concerns	Major concerns	No concerns	No concerns	Low	[Within-study bias, Imprecision]
Clot:Que	0	Some concerns	Low risk	No concerns	Major concerns	No concerns	No concerns	Low	[Within-study bias, Imprecision]
Clot:QueCloz	0	Major concerns	Low risk	No concerns	Major concerns	No concerns	No concerns	Low	[Within-study bias, Imprecision]
Clot:ResverRisp	0	Major concerns	Low risk	No concerns	Major concerns	No concerns	No concerns	Low	[Within-study bias, Imprecision]
Clot:Risp	0	Some concerns	Low risk	No concerns	Major concerns	No concerns	No concerns	Low	[Within-study bias, Imprecision]
Clot:RispCloz	0	Some concerns	Low risk	No concerns	Major concerns	No concerns	No concerns	Low	[Within-study bias, Imprecision]
Clot:SarcCloz	0	No concerns	Low risk	No concerns	Major concerns	No concerns	No concerns	Low	[Imprecision]
Clot:SerOla	0	Some concerns	Low risk	No concerns	Major concerns	No concerns	No concerns	Low	[Within-study bias, Imprecision]
Clot:Sertind	0	Some concerns	Low risk	No concerns	Major concerns	No concerns	No concerns	Low	[Within-study bias, Imprecision]
Clot:SertindCloz	0	Some concerns	Low risk	No concerns	Major concerns	No concerns	No concerns	Low	[Within-study bias, Imprecision]
Clot:SerZip	0	No concerns	Low risk	No concerns	Major concerns	No concerns	No concerns	Low	[Imprecision]
Clot:Sul	0	Some concerns	Low risk	No concerns	Some concerns	Some concerns	No concerns	Low	[Within-study bias, Imprecision, Heterogeneity]
Clot:SulCloz	0	Major concerns	Low risk	No concerns	Major concerns	No concerns	No concerns	Low	[Within-study bias, Imprecision]
Clot:SulOla	0	Major concerns	Low risk	No concerns	Major concerns	No concerns	No concerns	Low	[Within-study bias, Imprecision]

Clot:TopirCloz	0	Some concerns	Low risk	No concerns	Major concerns	No concerns	No concerns	Low	[Within-study bias, Imprecision]
Clot:VitD3Cloz	0	No concerns	Low risk	No concerns	Major concerns	No concerns	No concerns	Low	[Imprecision]
Clot:VortRisp	0	Some concerns	Low risk	No concerns	Major concerns	No concerns	No concerns	Low	[Within-study bias, Imprecision]
Clot:Zip	0	Major concerns	Low risk	No concerns	Major concerns	No concerns	No concerns	Low	[Within-study bias, Imprecision]
Clot:ZipCloz	0	Major concerns	Low risk	No concerns	Major concerns	No concerns	No concerns	Low	[Within-study bias, Imprecision]
Clot:Zot	0	Major concerns	Low risk	No concerns	Major concerns	No concerns	No concerns	Low	[Within-study bias, Imprecision]
Cloz:DesmopRisp	0	Major concerns	Low risk	Some concerns	Major concerns	No concerns	No concerns	Very Low	[Within-study bias, Indirectness, Imprecision]
Cloz:Fluph	0	Some concerns	Low risk	No concerns	Some concerns	Some concerns	No concerns	Low	[Within-study bias, Imprecision, Heterogeneity]
Cloz:GinkBilHal	0	Some concerns	Low risk	No concerns	Major concerns	No concerns	No concerns	Low	[Within-study bias, Imprecision]
Cloz:Lev	0	No concerns	Low risk	No concerns	Major concerns	No concerns	No concerns	Low	[Imprecision]
Cloz:LiHal	0	Some concerns	Low risk	Some concerns	Some concerns	Some concerns	No concerns	Low	[Within-study bias, Indirectness, Imprecision, Heterogeneity]
Cloz:MirtRisp	0	Some concerns	Low risk	Major concerns	Major concerns	No concerns	No concerns	Very Low	[Within-study bias, Indirectness, Imprecision]
Cloz:OlaRisp	0	Some concerns	Low risk	Some concerns	Major concerns	No concerns	No concerns	Low	[Within-study bias, Indirectness, Imprecision]
Cloz:OndastHal	0	No concerns	Low risk	No concerns	Major concerns	No concerns	No concerns	Low	[Imprecision]
Cloz:OxytRisp	0	Major concerns	Low risk	Major concerns	No concerns	No concerns	No concerns	Very Low	[Within-study bias, Indirectness]
Cloz:Palip	0	Some concerns	Low risk	No concerns	Major concerns	No concerns	No concerns	Low	[Within-study bias, Imprecision]

Cloz:Palipla	0	Some concerns	Low risk	Some concerns	Major concerns	No concerns	No concerns	Low	[Within-study bias, Indirectness, Imprecision]
Cloz:PalipOla	0	No concerns	Low risk	Major concerns	No concerns	No concerns	No concerns	Low	[Indirectness]
Cloz:PalmitRisp	0	Major concerns	Low risk	Major concerns	Some concerns	Some concerns	No concerns	Very Low	[Within-study bias, Indirectness, Imprecision, Heterogeneity]
Cloz:PtxRisp	0	Major concerns	Low risk	Major concerns	Major concerns	No concerns	No concerns	Very Low	[Within-study bias, Indirectness, Imprecision]
Cloz:QueCloz	0	Major concerns	Low risk	No concerns	Major concerns	No concerns	No concerns	Low	[Within-study bias, Imprecision]
Cloz:ResverRisp	0	Major concerns	Low risk	Major concerns	Major concerns	No concerns	No concerns	Very Low	[Within-study bias, Indirectness, Imprecision]
Cloz:SerOla	0	Some concerns	Low risk	No concerns	Major concerns	No concerns	No concerns	Low	[Within-study bias, Imprecision]
Cloz:Sertind	0	Some concerns	Low risk	No concerns	Some concerns	Some concerns	No concerns	Low	[Within-study bias, Imprecision, Heterogeneity]
Cloz:SerZip	0	No concerns	Low risk	No concerns	Major concerns	No concerns	No concerns	Low	[Imprecision]
Cloz:SulOla	0	Major concerns	Low risk	No concerns	Major concerns	No concerns	No concerns	Low	[Within-study bias, Imprecision]
Cloz:VortRisp	0	No concerns	Low risk	Major concerns	No concerns	Major concerns	No concerns	Low	[Indirectness, Heterogeneity]
Cpz:DesmopRisp	0	Major concerns	Low risk	Some concerns	No concerns	No concerns	No concerns	Low	[Within-study bias, Indirectness]
Cpz:DulCloz	0	No concerns	Low risk	No concerns	No concerns	No concerns	No concerns	High	
Cpz:Fluph	0	Some concerns	Low risk	No concerns	Major concerns	No concerns	No concerns	Low	[Within-study bias, Imprecision]
Cpz:FluvCloz	0	No concerns	Low risk	No concerns	Major concerns	No concerns	No concerns	Low	[Imprecision]
Cpz:GinkBilCloz	0	Some concerns	Low risk	No concerns	Major concerns	No concerns	No concerns	Low	[Within-study bias, Imprecision]
Cpz:GinkBilHal	0	Some concerns	Low risk	No concerns	Major concerns	No concerns	No concerns	Low	[Within-study bias, Imprecision]

Cpz:GlyCloz	0	Some concerns	Low risk	Some concerns	Major concerns	No concerns	No concerns	Low	[Within-study bias, Indirectness, Imprecision]
Cpz:Hal	0	Some concerns	Low risk	No concerns	Major concerns	No concerns	No concerns	Low	[Within-study bias, Imprecision]
Cpz:HalCloz	0	Some concerns	Low risk	No concerns	No concerns	Some concerns	No concerns	Moderate	[Within-study bias, Heterogeneity]
Cpz:LamCloz	0	Some concerns	Low risk	No concerns	No concerns	No concerns	No concerns	Moderate	[Within-study bias]
Cpz:LiHal	0	Some concerns	Low risk	No concerns	No concerns	No concerns	No concerns	Moderate	[Within-study bias]
Cpz:MemCloz	0	No concerns	Low risk	No concerns	No concerns	No concerns	No concerns	High	
Cpz:MetCloz	0	Some concerns	Low risk	No concerns	No concerns	No concerns	No concerns	Moderate	[Within-study bias]
Cpz:MinocCloz	0	No concerns	Low risk	No concerns	No concerns	Some concerns	No concerns	Moderate	[Heterogeneity]
Cpz:MirtCloz	0	Some concerns	Low risk	Some concerns	No concerns	No concerns	No concerns	Moderate	[Within-study bias, Indirectness]
Cpz:MirtRisp	0	Some concerns	Low risk	No concerns	Some concerns	Some concerns	No concerns	Low	[Within-study bias, Imprecision, Heterogeneity]
Cpz:ModfCloz	0	No concerns	Low risk	Some concerns	Major concerns	No concerns	No concerns	Low	[Indirectness, Imprecision]
Cpz:OlaRisp	0	Some concerns	Low risk	No concerns	No concerns	Some concerns	No concerns	Moderate	[Within-study bias, Heterogeneity]
Cpz:OndastHal	0	Some concerns	Low risk	No concerns	Some concerns	Some concerns	No concerns	Low	[Within-study bias, Imprecision, Heterogeneity]
Cpz:OxytRisp	0	Major concerns	Low risk	Some concerns	No concerns	No concerns	No concerns	Low	[Within-study bias, Indirectness]
Cpz:Palip	0	Some concerns	Low risk	No concerns	Major concerns	No concerns	No concerns	Low	[Within-study bias, Imprecision]
Cpz:Palipla	0	Some concerns	Low risk	No concerns	Major concerns	No concerns	No concerns	Low	[Within-study bias, Imprecision]
Cpz:PalipOla	0	No concerns	Low risk	Major concerns	No concerns	No concerns	No concerns	Low	[Indirectness]

Cpz:PalmitRisp	0	Major concerns	Low risk	No concerns	No concerns	No concerns	No concerns	Low	[Within-study bias]
Cpz:PhenylpropCloz	0	Some concerns	Low risk	No concerns	Major concerns	No concerns	No concerns	Low	[Within-study bias, Imprecision]
Cpz:PimozCloz	0	No concerns	Low risk	No concerns	Major concerns	No concerns	No concerns	Low	[Imprecision]
Cpz:PtxRisp	0	Major concerns	Low risk	No concerns	Some concerns	Some concerns	No concerns	Low	[Within-study bias, Imprecision, Heterogeneity]
Cpz:QueCloz	0	Some concerns	Low risk	No concerns	Major concerns	No concerns	No concerns	Low	[Within-study bias, Imprecision]
Cpz:ResverRisp	0	Major concerns	Low risk	No concerns	No concerns	No concerns	No concerns	Low	[Within-study bias]
Cpz:Risp	0	Some concerns	Low risk	No concerns	Some concerns	Some concerns	No concerns	Low	[Within-study bias, Imprecision, Heterogeneity]
Cpz:RispCloz	0	Some concerns	Low risk	No concerns	No concerns	No concerns	No concerns	Moderate	[Within-study bias]
Cpz:SarcCloz	0	No concerns	Low risk	No concerns	Major concerns	No concerns	No concerns	Low	[Imprecision]
Cpz:SerOla	0	Some concerns	Low risk	No concerns	Major concerns	No concerns	No concerns	Low	[Within-study bias, Imprecision]
Cpz:Sertind	0	Some concerns	Low risk	No concerns	Major concerns	No concerns	No concerns	Low	[Within-study bias, Imprecision]
Cpz:SertindCloz	0	Some concerns	Low risk	No concerns	Major concerns	No concerns	No concerns	Low	[Within-study bias, Imprecision]
Cpz:SerZip	0	No concerns	Low risk	No concerns	Major concerns	No concerns	No concerns	Low	[Imprecision]
Cpz:Sul	0	Some concerns	Low risk	No concerns	Major concerns	No concerns	No concerns	Low	[Within-study bias, Imprecision]
Cpz:SulCloz	0	Some concerns	Low risk	No concerns	No concerns	No concerns	No concerns	Moderate	[Within-study bias]
Cpz:SulOla	0	Some concerns	Low risk	No concerns	Major concerns	No concerns	No concerns	Low	[Within-study bias, Imprecision]

Cpz:TopirCloz	0	Some concerns	Low risk	No concerns	No concerns	No concerns	No concerns	Moderate	[Within-study bias]
Cpz:VitD3Cloz	0	No concerns	Low risk	Some concerns	Major concerns	No concerns	No concerns	Low	[Indirectness, Imprecision]
Cpz:VortRisp	0	Some concerns	Low risk	No concerns	No concerns	No concerns	No concerns	Moderate	[Within-study bias]
Cpz:ZipCloz	0	No concerns	Low risk	No concerns	No concerns	No concerns	No concerns	High	
Cpz:Zot	0	Some concerns	Low risk	Some concerns	Major concerns	No concerns	No concerns	Low	[Within-study bias, Indirectness, Imprecision]
DesmopRisp:DulCloz	0	Major concerns	Low risk	No concerns	Major concerns	No concerns	No concerns	Low	[Within-study bias, Imprecision]
DesmopRisp:Fluph	0	Major concerns	Low risk	Some concerns	No concerns	No concerns	No concerns	Low	[Within-study bias, Indirectness]
DesmopRisp:FluvCloz	0	Major concerns	Low risk	No concerns	Major concerns	No concerns	No concerns	Low	[Within-study bias, Imprecision]
DesmopRisp:GinkBilCloz	0	Major concerns	Low risk	No concerns	Major concerns	No concerns	No concerns	Low	[Within-study bias, Imprecision]
DesmopRisp:GinkBilHal	0	Major concerns	Low risk	No concerns	Major concerns	No concerns	No concerns	Low	[Within-study bias, Imprecision]
DesmopRisp:GlyCloz	0	Major concerns	Low risk	Some concerns	Major concerns	No concerns	No concerns	Very Low	[Within-study bias, Indirectness, Imprecision]
DesmopRisp:Hal	0	Major concerns	Low risk	Some concerns	No concerns	No concerns	No concerns	Low	[Within-study bias, Indirectness]
DesmopRisp:HalCloz	0	Major concerns	Low risk	No concerns	Major concerns	No concerns	No concerns	Low	[Within-study bias, Imprecision]
DesmopRisp:LamCloz	0	Major concerns	Low risk	No concerns	Major concerns	No concerns	No concerns	Low	[Within-study bias, Imprecision]
DesmopRisp:Lev	0	Major concerns	Low risk	No concerns	Major concerns	No concerns	No concerns	Low	[Within-study bias, Imprecision]
DesmopRisp:LiHal	0	Major concerns	Low risk	Some concerns	Major concerns	No concerns	No concerns	Very Low	[Within-study bias, Indirectness, Imprecision]
DesmopRisp:MemCloz	0	Major concerns	Low risk	Some concerns	Major concerns	No concerns	No concerns	Very Low	[Within-study bias, Indirectness, Imprecision]
DesmopRisp:MetCloz	0	Major concerns	Low risk	Some concerns	Major concerns	No concerns	No concerns	Very Low	[Within-study bias, Indirectness, Imprecision]

DesmopRisp:MinocCloz	0	Major concerns	Low risk	No concerns	Major concerns	No concerns	No concerns	Low	[Within-study bias, Imprecision]
DesmopRisp:MirtCloz	0	Major concerns	Low risk	Some concerns	Major concerns	No concerns	No concerns	Vey Low	[Within-study bias, Indirectness, Imprecision]
DesmopRisp:MirtRisp	0	Major concerns	Low risk	Some concerns	Major concerns	No concerns	No concerns	Vey Low	[Within-study bias, Indirectness, Imprecision]
DesmopRisp:ModfCloz	0	Major concerns	Low risk	Some concerns	Major concerns	No concerns	No concerns	Vey Low	[Within-study bias, Indirectness, Imprecision]
DesmopRisp:Ola	0	Major concerns	Low risk	Some concerns	Some concerns	Some concerns	No concerns	Vey Low	[Within-study bias, Indirectness, Imprecision, Heterogeneity]
DesmopRisp:OlaRisp	0	Major concerns	Low risk	Some concerns	Major concerns	No concerns	No concerns	Vey Low	[Within-study bias, Indirectness, Imprecision]
DesmopRisp:OndastHal	0	Major concerns	Low risk	No concerns	Major concerns	No concerns	No concerns	Low	[Within-study bias, Imprecision]
DesmopRisp:OxytRisp	0	Major concerns	Low risk	Some concerns	Major concerns	No concerns	Some concerns	Vey Low	[Within-study bias, Indirectness, Imprecision, Incoherence]
DesmopRisp:Palip	0	Major concerns	Low risk	Some concerns	Major concerns	No concerns	No concerns	Vey Low	[Within-study bias, Indirectness, Imprecision]
DesmopRisp:Palipla	0	Major concerns	Low risk	Some concerns	Major concerns	No concerns	No concerns	Vey Low	[Within-study bias, Indirectness, Imprecision]
DesmopRisp:PalipOla	0	Major concerns	Low risk	Some concerns	Major concerns	No concerns	No concerns	Vey Low	[Within-study bias, Indirectness, Imprecision]
DesmopRisp:PalmitRisp	0	Major concerns	Low risk	Some concerns	Major concerns	No concerns	No concerns	Vey Low	[Within-study bias, Indirectness, Imprecision]
DesmopRisp:PhenylpropCloz	0	Major concerns	Low risk	Some concerns	Major concerns	No concerns	No concerns	Vey Low	[Within-study bias, Indirectness, Imprecision]
DesmopRisp:PimozCloz	0	Major concerns	Low risk	No concerns	Some concerns	Some concerns	No concerns	Low	[Within-study bias, Imprecision, Heterogeneity]
DesmopRisp:PtxRisp	0	Major concerns	Low risk	Some concerns	Major concerns	No concerns	No concerns	Vey Low	[Within-study bias, Indirectness, Imprecision]
DesmopRisp:Que	0	Major concerns	Low risk	Some concerns	No concerns	No concerns	No concerns	Low	[Within-study bias, Indirectness]

DesmopRisp:QueCloz	0	Major concerns	Low risk	No concerns	No concerns	No concerns	No concerns	Low	[Within-study bias]
DesmopRisp:ResverRisp	0	Major concerns	Low risk	Some concerns	Major concerns	No concerns	No concerns	Vey Low	[Within-study bias, Indirectness, Imprecision]
DesmopRisp:RispCloz	0	Major concerns	Low risk	No concerns	Major concerns	No concerns	No concerns	Low	[Within-study bias, Imprecision]
DesmopRisp:SarcCloz	0	Major concerns	Low risk	No concerns	Major concerns	No concerns	No concerns	Low	[Within-study bias, Imprecision]
DesmopRisp:SerOla	0	Major concerns	Low risk	Some concerns	Major concerns	No concerns	No concerns	Vey Low	[Within-study bias, Indirectness, Imprecision]
DesmopRisp:Sertind	0	Major concerns	Low risk	No concerns	No concerns	No concerns	No concerns	Low	[Within-study bias]
DesmopRisp:SertindCloz	0	Major concerns	Low risk	No concerns	Major concerns	No concerns	No concerns	Low	[Within-study bias, Imprecision]
DesmopRisp:SerZip	0	Major concerns	Low risk	No concerns	Major concerns	No concerns	No concerns	Low	[Within-study bias, Imprecision]
DesmopRisp:Sul	0	Major concerns	Low risk	No concerns	No concerns	No concerns	No concerns	Low	[Within-study bias]
DesmopRisp:SulCloz	0	Major concerns	Low risk	No concerns	Major concerns	No concerns	No concerns	Low	[Within-study bias, Imprecision]
DesmopRisp:SulOla	0	Major concerns	Low risk	Some concerns	Major concerns	No concerns	No concerns	Vey Low	[Within-study bias, Indirectness, Imprecision]
DesmopRisp:TopirCloz	0	Major concerns	Low risk	No concerns	Major concerns	No concerns	No concerns	Low	[Within-study bias, Imprecision]
DesmopRisp:VitD3Cloz	0	Major concerns	Low risk	Some concerns	Major concerns	No concerns	No concerns	Vey Low	[Within-study bias, Indirectness, Imprecision]
DesmopRisp:VortRisp	0	Major concerns	Low risk	Some concerns	Major concerns	No concerns	No concerns	Vey Low	[Within-study bias, Indirectness, Imprecision]
DesmopRisp:Zip	0	Major concerns	Low risk	Some concerns	No concerns	Some concerns	No concerns	Low	[Within-study bias, Indirectness, Heterogeneity]
DesmopRisp:ZipCloz	0	Major concerns	Low risk	No concerns	Major concerns	No concerns	No concerns	Low	[Within-study bias, Imprecision]
DesmopRisp:Zot	0	Major concerns	Low risk	Some concerns	Major concerns	No concerns	No concerns	Very Low	[Within-study bias, Indirectness, Imprecision]

DulCloz:Fluph	0	Some concerns	Low risk	No concerns	No concerns	No concerns	No concerns	Moderate	[Within-study bias]
DulCloz:FluvCloz	0	No concerns	Low risk	No concerns	No concerns	No concerns	No concerns	High	
DulCloz:GinkBilCloz	0	No concerns	Low risk	No concerns	No concerns	Some concerns	No concerns	Moderate	[Heterogeneity]
DulCloz:GinkBilHal	0	Some concerns	Low risk	No concerns	No concerns	No concerns	No concerns	Moderate	[Within-study bias]
DulCloz:GlyCloz	0	No concerns	Low risk	No concerns	Some concerns	Some concerns	No concerns	Moderate	[Imprecision, Heterogeneity]
DulCloz:Hal	0	No concerns	Low risk	No concerns	No concerns	No concerns	No concerns	High	
DulCloz:HalCloz	0	No concerns	Low risk	No concerns	No concerns	Some concerns	No concerns	Moderate	[Heterogeneity]
DulCloz:LamCloz	0	No concerns	Low risk	No concerns	No concerns	Some concerns	No concerns	Moderate	[Heterogeneity]
DulCloz:Lev	0	No concerns	Low risk	No concerns	No concerns	Some concerns	No concerns	Moderate	[Heterogeneity]
DulCloz:LiHal	0	No concerns	Low risk	No concerns	Major concerns	No concerns	No concerns	Low	[Imprecision]
DulCloz:MemCloz	0	No concerns	Low risk	No concerns	Major concerns	No concerns	No concerns	Low	[Imprecision]
DulCloz:MetCloz	0	No concerns	Low risk	No concerns	No concerns	Major concerns	No concerns	Low	[Heterogeneity]
DulCloz:MinocCloz	0	No concerns	Low risk	No concerns	Major concerns	No concerns	No concerns	Low	[Imprecision]
DulCloz:MirtCloz	0	No concerns	Low risk	No concerns	Major concerns	No concerns	No concerns	Low	[Imprecision]
DulCloz:MirtRisp	0	Some concerns	Low risk	No concerns	Major concerns	No concerns	No concerns	Low	[Within-study bias, Imprecision]
DulCloz:ModfCloz	0	No concerns	Low risk	No concerns	No concerns	Some concerns	No concerns	Moderate	[Heterogeneity]
DulCloz:Ola	0	No concerns	Low risk	No concerns	No concerns	No concerns	No concerns	High	
DulCloz:OlaRisp	0	Some concerns	Low risk	No concerns	No concerns	No concerns	No concerns	Moderate	[Within-study bias]
DulCloz:OndastHal	0	No concerns	Low risk	No concerns	No concerns	No concerns	No concerns	High	
DulCloz:OxytRisp	0	Major concerns	Low risk	No concerns	Major concerns	No concerns	No concerns	Low	[Within-study bias, Imprecision]
DulCloz:Palip	0	Some concerns	Low risk	No concerns	No concerns	No concerns	No concerns	Moderate	[Within-study bias]
DulCloz:Palipla	0	Some concerns	Low risk	No concerns	No concerns	No concerns	No concerns	Moderate	[Within-study bias]

DulCloz:PalipOla	0	No concerns	Low risk	No concerns	Major concerns	No concerns	No concerns	Low	[Imprecision]
DulCloz:PalmitRisp	0	Major concerns	Low risk	No concerns	Major concerns	No concerns	No concerns	Low	[Within-study bias, Imprecision]
DulCloz:Phenylprop Cloz	0	No concerns	Low risk	No concerns	Major concerns	No concerns	No concerns	Low	[Imprecision]
DulCloz:PimozCloz	0	No concerns	Low risk	No concerns	No concerns	No concerns	No concerns	High	
DulCloz:PtxRisp	0	Major concerns	Low risk	No concerns	No concerns	No concerns	No concerns	Low	[Within-study bias]
DulCloz:Que	0	No concerns	Low risk	No concerns	No concerns	No concerns	No concerns	High	
DulCloz:QueCloz	0	Major concerns	Low risk	No concerns	No concerns	No concerns	No concerns	Low	[Within-study bias]
DulCloz:ResverRisp	0	Major concerns	Low risk	No concerns	Major concerns	No concerns	No concerns	Low	[Within-study bias, Imprecision]
DulCloz:Risp	0	No concerns	Low risk	No concerns	No concerns	No concerns	No concerns	High	
DulCloz:RispCloz	0	No concerns	Low risk	No concerns	No concerns	No concerns	No concerns	High	
DulCloz:SarcCloz	0	No concerns	Low risk	No concerns	No concerns	No concerns	No concerns	High	
DulCloz:SerOla	0	Some concerns	Low risk	No concerns	No concerns	No concerns	No concerns	Moderate	[Within-study bias]
DulCloz:Sertind	0	Some concerns	Low risk	No concerns	No concerns	No concerns	No concerns	Moderate	[Within-study bias]
DulCloz:SertindCloz	0	No concerns	Low risk	No concerns	No concerns	No concerns	No concerns	High	
DulCloz:SerZip	0	No concerns	Low risk	No concerns	No concerns	No concerns	No concerns	High	
DulCloz:Sul	0	No concerns	Low risk	No concerns	No concerns	No concerns	No concerns	High	
DulCloz:SulCloz	0	No concerns	Low risk	No concerns	Major concerns	No concerns	No concerns	Low	[Imprecision]
DulCloz:SulOla	0	No concerns	Low risk	No concerns	Major concerns	No concerns	No concerns	Low	[Imprecision]
DulCloz:TopirCloz	0	No concerns	Low risk	No concerns	Some concerns	Some concerns	No concerns	Moderate	[Imprecision, Heterogeneity]
DulCloz:VitD3Cloz	0	No concerns	Low risk	No concerns	No concerns	No concerns	No concerns	High	
DulCloz:VortRisp	0	No concerns	Low risk	No concerns	Major concerns	No concerns	No concerns	Low	[Imprecision]
DulCloz:Zip	0	No concerns	Low risk	No concerns	No concerns	No concerns	No concerns	High	

DulCloz:ZipCloz	0	No concerns	Low risk	No concerns	Major concerns	No concerns	No concerns	Low	[Imprecision]
DulCloz:Zot	0	No concerns	Low risk	No concerns	No concerns	No concerns	No concerns	High	
Fluph:FluvCloz	0	Some concerns	Low risk	No concerns	Major concerns	No concerns	No concerns	Low	[Within-study bias, Imprecision]
Fluph:GinkBilCloz	0	Some concerns	Low risk	No concerns	Major concerns	No concerns	No concerns	Low	[Within-study bias, Imprecision]
Fluph:GinkBilHal	0	Some concerns	Low risk	No concerns	Major concerns	No concerns	No concerns	Low	[Within-study bias, Imprecision]
Fluph:GlyCloz	0	Some concerns	Low risk	Some concerns	Major concerns	No concerns	No concerns	Low	[Within-study bias, Indirectness, Imprecision]
Fluph:HalCloz	0	Some concerns	Low risk	No concerns	Some concerns	Some concerns	No concerns	Low	[Within-study bias, Imprecision, Heterogeneity]
Fluph:LamCloz	0	Some concerns	Low risk	No concerns	No concerns	No concerns	No concerns	Moderate	[Within-study bias]
Fluph:Lev	0	Some concerns	Low risk	No concerns	Major concerns	No concerns	No concerns	Low	[Within-study bias, Imprecision]
Fluph:LiHal	0	Some concerns	Low risk	Some concerns	No concerns	No concerns	No concerns	Moderate	[Within-study bias, Indirectness]
Fluph:MemCloz	0	Some concerns	Low risk	No concerns	No concerns	No concerns	No concerns	Moderate	[Within-study bias]
Fluph:MetCloz	0	Some concerns	Low risk	No concerns	No concerns	Major concerns	No concerns	Low	[Within-study bias, Heterogeneity]
Fluph:MinocCloz	0	Some concerns	Low risk	No concerns	Some concerns	Some concerns	No concerns	Low	[Within-study bias, Imprecision, Heterogeneity]
Fluph:MirtCloz	0	Some concerns	Low risk	Some concerns	No concerns	No concerns	No concerns	Moderate	[Within-study bias, Indirectness]
Fluph:MirtRisp	0	Some concerns	Low risk	No concerns	Major concerns	No concerns	No concerns	Low	[Within-study bias, Imprecision]
Fluph:ModfCloz	0	Some concerns	Low risk	Some concerns	Major concerns	No concerns	No concerns	Low	[Within-study bias, Indirectness, Imprecision]
Fluph:Ola	0	Some concerns	Low risk	No concerns	Major concerns	No concerns	No concerns	Low	[Within-study bias, Imprecision]

Fluph:OlaRisp	0	Some concerns	Low risk	Some concerns	Some concerns	Some concerns	No concerns	Low	[Within-study bias, Indirectness, Imprecision, Heterogeneity]
Fluph:OndastHal	0	Some concerns	Low risk	No concerns	Major concerns	No concerns	No concerns	Low	[Within-study bias, Imprecision]
Fluph:OxytRisp	0	Major concerns	Low risk	Some concerns	No concerns	No concerns	No concerns	Low	[Within-study bias, Indirectness]
Fluph:Palip	0	Some concerns	Low risk	No concerns	Major concerns	No concerns	No concerns	Low	[Within-study bias, Imprecision]
Fluph:Palipla	0	Some concerns	Low risk	No concerns	Major concerns	No concerns	No concerns	Low	[Within-study bias, Imprecision]
Fluph:PalipOla	0	Some concerns	Low risk	No concerns	No concerns	No concerns	No concerns	Moderate	[Within-study bias]
Fluph:PalmitRisp	0	Major concerns	Low risk	No concerns	No concerns	No concerns	No concerns	Low	[Within-study bias]
Fluph:PhenylpropCl oz	0	Some concerns	Low risk	No concerns	Major concerns	No concerns	No concerns	Low	[Within-study bias, Imprecision]
Fluph:PimozCloz	0	Some concerns	Low risk	No concerns	Major concerns	No concerns	No concerns	Low	[Within-study bias, Imprecision]
Fluph:PtxRisp	0	Major concerns	Low risk	No concerns	Major concerns	No concerns	No concerns	Low	[Within-study bias, Imprecision]
Fluph:QueCloz	0	Some concerns	Low risk	No concerns	Major concerns	No concerns	No concerns	Low	[Within-study bias, Imprecision]
Fluph:ResverRisp	0	Major concerns	Low risk	No concerns	No concerns	Some concerns	No concerns	Low	[Within-study bias, Heterogeneity]
Fluph:RispCloz	0	Some concerns	Low risk	No concerns	No concerns	Some concerns	No concerns	Moderate	[Within-study bias, Heterogeneity]
Fluph:SarcCloz	0	Some concerns	Low risk	No concerns	Major concerns	No concerns	No concerns	Low	[Within-study bias, Imprecision]
Fluph:SerOla	0	Some concerns	Low risk	No concerns	Major concerns	No concerns	No concerns	Low	[Within-study bias, Imprecision]
Fluph:Sertind	0	Some concerns	Low risk	No concerns	Major concerns	No concerns	No concerns	Low	[Within-study bias, Imprecision]
Fluph:SertindCloz	0	Some concerns	Low risk	No concerns	Major concerns	No concerns	No concerns	Low	[Within-study bias, Imprecision]

Fluph:SerZip	0	Some concerns	Low risk	No concerns	Major concerns	No concerns	No concerns	Low	[Within-study bias, Imprecision]
Fluph:Sul	0	Some concerns	Low risk	No concerns	Major concerns	No concerns	No concerns	Low	[Within-study bias, Imprecision]
Fluph:SulCloz	0	Some concerns	Low risk	No concerns	No concerns	No concerns	No concerns	Moderate	[Within-study bias]
Fluph:SulOla	0	Some concerns	Low risk	No concerns	Major concerns	No concerns	No concerns	Low	[Within-study bias, Imprecision]
Fluph:TopirCloz	0	Some concerns	Low risk	No concerns	No concerns	No concerns	No concerns	Moderate	[Within-study bias]
Fluph:VitD3Cloz	0	Some concerns	Low risk	Some concerns	Major concerns	No concerns	No concerns	Low	[Within-study bias, Indirectness, Imprecision]
Fluph:VortRisp	0	Some concerns	Low risk	No concerns	No concerns	No concerns	No concerns	Moderate	[Within-study bias]
Fluph:Zip	0	Some concerns	Low risk	No concerns	Major concerns	No concerns	No concerns	Low	[Within-study bias, Imprecision]
Fluph:ZipCloz	0	Some concerns	Low risk	No concerns	No concerns	No concerns	No concerns	Moderate	[Within-study bias]
Fluph:Zot	0	Some concerns	Low risk	Some concerns	Major concerns	No concerns	No concerns	Low	[Within-study bias, Indirectness, Imprecision]
FluvCloz:GinkBilCloz	0	No concerns	Low risk	No concerns	Major concerns	No concerns	No concerns	Low	[Imprecision]
FluvCloz:GinkBilHal	0	Some concerns	Low risk	No concerns	Major concerns	No concerns	No concerns	Low	[Within-study bias, Imprecision]
FluvCloz:GlyCloz	0	No concerns	Low risk	No concerns	Major concerns	No concerns	No concerns	Low	[Imprecision]
FluvCloz:Hal	0	No concerns	Low risk	No concerns	Major concerns	No concerns	No concerns	Low	[Imprecision]
FluvCloz:HalCloz	0	No concerns	Low risk	No concerns	Major concerns	No concerns	No concerns	Low	[Imprecision]
FluvCloz:LamCloz	0	No concerns	Low risk	No concerns	Major concerns	No concerns	No concerns	Low	[Imprecision]
FluvCloz:Lev	0	No concerns	Low risk	No concerns	Major concerns	No concerns	No concerns	Low	[Imprecision]

FluvCloz:LiHal	0	No concerns	Low risk	No concerns	Major concerns	No concerns	No concerns	Low	[Imprecision]
FluvCloz:MemCloz	0	No concerns	Low risk	No concerns	Major concerns	No concerns	No concerns	Low	[Imprecision]
FluvCloz:MetCloz	0	No concerns	Low risk	No concerns	Major concerns	No concerns	No concerns	Low	[Imprecision]
FluvCloz:MinocCloz	0	No concerns	Low risk	No concerns	Major concerns	No concerns	No concerns	Low	[Imprecision]
FluvCloz:MirtCloz	0	No concerns	Low risk	No concerns	No concerns	Some concerns	No concerns	Moderate	[Heterogeneity]
FluvCloz:MirtRisp	0	Some concerns	Low risk	No concerns	Major concerns	No concerns	No concerns	Low	[Within-study bias, Imprecision]
FluvCloz:ModfCloz	0	No concerns	Low risk	No concerns	Major concerns	No concerns	No concerns	Low	[Imprecision]
FluvCloz:Ola	0	No concerns	Low risk	No concerns	Major concerns	No concerns	No concerns	Low	[Imprecision]
FluvCloz:OlaRisp	0	Some concerns	Low risk	No concerns	Major concerns	No concerns	No concerns	Low	[Within-study bias, Imprecision]
FluvCloz:OndastHal	0	No concerns	Low risk	No concerns	Major concerns	No concerns	No concerns	Low	[Imprecision]
FluvCloz:OxytRisp	0	Major concerns	Low risk	No concerns	No concerns	No concerns	No concerns	Low	[Within-study bias]
FluvCloz:Palip	0	Some concerns	Low risk	No concerns	Major concerns	No concerns	No concerns	Low	[Within-study bias, Imprecision]
FluvCloz:Palipla	0	Some concerns	Low risk	No concerns	Major concerns	No concerns	No concerns	Low	[Within-study bias, Imprecision]
FluvCloz:PalipOla	0	No concerns	Low risk	No concerns	No concerns	Some concerns	No concerns	Moderate	[Heterogeneity]
FluvCloz:PalmitRisp	0	Major concerns	Low risk	No concerns	Major concerns	No concerns	No concerns	Low	[Within-study bias, Imprecision]
FluvCloz:PhenylpropCloz	0	No concerns	Low risk	No concerns	Major concerns	No concerns	No concerns	Low	[Imprecision]
FluvCloz:PimozCloz	0	No concerns	Low risk	No concerns	Major concerns	No concerns	No concerns	Low	[Imprecision]
FluvCloz:PtxRisp	0	Major concerns	Low risk	No concerns	Major concerns	No concerns	No concerns	Low	[Within-study bias, Imprecision]

FluvCloz:Que	0	No concerns	Low risk	No concerns	Major concerns	No concerns	No concerns	Low	[Imprecision]
FluvCloz:QueCloz	0	Major concerns	Low risk	No concerns	Major concerns	No concerns	No concerns	Low	[Within-study bias, Imprecision]
FluvCloz:ResverRisp	0	Major concerns	Low risk	No concerns	Major concerns	No concerns	No concerns	Low	[Within-study bias, Imprecision]
FluvCloz:Risp	0	No concerns	Low risk	No concerns	Major concerns	No concerns	No concerns	Low	[Imprecision]
FluvCloz:RispCloz	0	No concerns	Low risk	No concerns	Major concerns	No concerns	No concerns	Low	[Imprecision]
FluvCloz:SarcCloz	0	No concerns	Low risk	No concerns	Major concerns	No concerns	No concerns	Low	[Imprecision]
FluvCloz:SerOla	0	Some concerns	Low risk	No concerns	Major concerns	No concerns	No concerns	Low	[Within-study bias, Imprecision]
FluvCloz:Sertind	0	Some concerns	Low risk	No concerns	Major concerns	No concerns	No concerns	Low	[Within-study bias, Imprecision]
FluvCloz:SertindCloz	0	No concerns	Low risk	No concerns	Major concerns	No concerns	No concerns	Low	[Imprecision]
FluvCloz:SerZip	0	No concerns	Low risk	No concerns	Major concerns	No concerns	No concerns	Low	[Imprecision]
FluvCloz:Sul	0	No concerns	Low risk	No concerns	Major concerns	No concerns	No concerns	Low	[Imprecision]
FluvCloz:SulCloz	0	No concerns	Low risk	No concerns	Major concerns	No concerns	No concerns	Low	[Imprecision]
FluvCloz:SulOla	0	No concerns	Low risk	No concerns	Major concerns	No concerns	No concerns	Low	[Imprecision]
FluvCloz:TopirCloz	0	No concerns	Low risk	No concerns	Major concerns	No concerns	No concerns	Low	[Imprecision]
FluvCloz:VitD3Cloz	0	No concerns	Low risk	No concerns	Major concerns	No concerns	No concerns	Low	[Imprecision]
FluvCloz:VortRisp	0	No concerns	Low risk	No concerns	Major concerns	No concerns	No concerns	Low	[Imprecision]
FluvCloz:Zip	0	No concerns	Low risk	No concerns	Major concerns	No concerns	No concerns	Low	[Imprecision]
FluvCloz:ZipCloz	0	No concerns	Low risk	No concerns	No concerns	Major concerns	No concerns	Low	[Heterogeneity]

FluvCloz:Zot	0	No concerns	Low risk	No concerns	Major concerns	No concerns	No concerns	Low	[Imprecision]
GinkBilCloz:GinkBilHal	0	Some concerns	Low risk	No concerns	Major concerns	No concerns	No concerns	Low	[Within-study bias, Imprecision]
GinkBilCloz:GlyCloz	0	Some concerns	Low risk	No concerns	Major concerns	No concerns	No concerns	Low	[Within-study bias, Imprecision]
GinkBilCloz:Hal	0	Some concerns	Low risk	No concerns	Major concerns	No concerns	No concerns	Low	[Within-study bias, Imprecision]
GinkBilCloz:HalCloz	0	Some concerns	Low risk	No concerns	Major concerns	No concerns	No concerns	Low	[Within-study bias, Imprecision]
GinkBilCloz:LamCloz	0	Some concerns	Low risk	No concerns	Major concerns	No concerns	No concerns	Low	[Within-study bias, Imprecision]
GinkBilCloz:Lev	0	Some concerns	Low risk	No concerns	Major concerns	No concerns	No concerns	Low	[Within-study bias, Imprecision]
GinkBilCloz:LiHal	0	Some concerns	Low risk	No concerns	Major concerns	No concerns	No concerns	Low	[Within-study bias, Imprecision]
GinkBilCloz:MemCloz	0	No concerns	Low risk	No concerns	Major concerns	No concerns	No concerns	Low	[Imprecision]
GinkBilCloz:MetCloz	0	Some concerns	Low risk	No concerns	Major concerns	No concerns	No concerns	Low	[Within-study bias, Imprecision]
GinkBilCloz:MinocCloz	0	No concerns	Low risk	No concerns	Major concerns	No concerns	No concerns	Low	[Imprecision]
GinkBilCloz:MirtCloz	0	Some concerns	Low risk	No concerns	Major concerns	No concerns	No concerns	Low	[Within-study bias, Imprecision]
GinkBilCloz:MirtRisp	0	Some concerns	Low risk	No concerns	Major concerns	No concerns	No concerns	Low	[Within-study bias, Imprecision]
GinkBilCloz:ModfCloz	0	No concerns	Low risk	No concerns	Major concerns	No concerns	No concerns	Low	[Imprecision]
GinkBilCloz:Ola	0	Some concerns	Low risk	No concerns	Major concerns	No concerns	No concerns	Low	[Within-study bias, Imprecision]
GinkBilCloz:OlaRisp	0	Some concerns	Low risk	No concerns	Major concerns	No concerns	No concerns	Low	[Within-study bias, Imprecision]
GinkBilCloz:OndastHal	0	Some concerns	Low risk	No concerns	Major concerns	No concerns	No concerns	Low	[Within-study bias, Imprecision]

GinkBilCloz:OxytRisp	0	Major concerns	Low risk	No concerns	No concerns	No concerns	No concerns	Low	[Within-study bias]
GinkBilCloz:Palip	0	Some concerns	Low risk	No concerns	Major concerns	No concerns	No concerns	Low	[Within-study bias, Imprecision]
GinkBilCloz:Palipla	0	Some concerns	Low risk	No concerns	Major concerns	No concerns	No concerns	Low	[Within-study bias, Imprecision]
GinkBilCloz:PalipOla	0	Some concerns	Low risk	No concerns	Major concerns	No concerns	No concerns	Low	[Within-study bias, Imprecision]
GinkBilCloz:PalmitRisp	0	Major concerns	Low risk	No concerns	Major concerns	No concerns	No concerns	Low	[Within-study bias, Imprecision]
GinkBilCloz:PhenylpropCloz	0	Some concerns	Low risk	No concerns	Major concerns	No concerns	No concerns	Low	[Within-study bias, Imprecision]
GinkBilCloz:PimozCloz	0	No concerns	Low risk	No concerns	Major concerns	No concerns	No concerns	Low	[Imprecision]
GinkBilCloz:PtxRisp	0	Major concerns	Low risk	No concerns	Major concerns	No concerns	No concerns	Low	[Within-study bias, Imprecision]
GinkBilCloz:Que	0	Some concerns	Low risk	No concerns	Major concerns	No concerns	No concerns	Low	[Within-study bias, Imprecision]
GinkBilCloz:QueCloz	0	Some concerns	Low risk	No concerns	Major concerns	No concerns	No concerns	Low	[Within-study bias, Imprecision]
GinkBilCloz:ResverRisp	0	Major concerns	Low risk	No concerns	Major concerns	No concerns	No concerns	Low	[Within-study bias, Imprecision]
GinkBilCloz:Risp	0	Some concerns	Low risk	No concerns	Major concerns	No concerns	No concerns	Low	[Within-study bias, Imprecision]
GinkBilCloz:RispCloz	0	Some concerns	Low risk	No concerns	Major concerns	No concerns	No concerns	Low	[Within-study bias, Imprecision]
GinkBilCloz:SarcCloz	0	No concerns	Low risk	No concerns	Major concerns	No concerns	No concerns	Low	[Imprecision]
GinkBilCloz:SerOla	0	Some concerns	Low risk	No concerns	Major concerns	No concerns	No concerns	Low	[Within-study bias, Imprecision]
GinkBilCloz:Sertind	0	Some concerns	Low risk	No concerns	Major concerns	No concerns	No concerns	Low	[Within-study bias, Imprecision]
GinkBilCloz:SertindCloz	0	Some concerns	Low risk	No concerns	Major concerns	No concerns	No concerns	Low	[Within-study bias, Imprecision]

GinkBilCloz:SerZip	0	Some concerns	Low risk	No concerns	Major concerns	No concerns	No concerns	Low	[Within-study bias, Imprecision]
GinkBilCloz:Sul	0	Some concerns	Low risk	No concerns	No concerns	Major concerns	No concerns	Low	[Within-study bias, Heterogeneity]
GinkBilCloz:SulCloz	0	Some concerns	Low risk	No concerns	Major concerns	No concerns	No concerns	Low	[Within-study bias, Imprecision]
GinkBilCloz:SulOla	0	Some concerns	Low risk	No concerns	Major concerns	No concerns	No concerns	Low	[Within-study bias, Imprecision]
GinkBilCloz:TopirCloz	0	Some concerns	Low risk	No concerns	Major concerns	No concerns	No concerns	Low	[Within-study bias, Imprecision]
GinkBilCloz:VitD3Cloz	0	No concerns	Low risk	No concerns	Major concerns	No concerns	No concerns	Low	[Imprecision]
GinkBilCloz:VortRisp	0	Some concerns	Low risk	No concerns	Major concerns	No concerns	No concerns	Low	[Within-study bias, Imprecision]
GinkBilCloz:Zip	0	Some concerns	Low risk	No concerns	Major concerns	No concerns	No concerns	Low	[Within-study bias, Imprecision]
GinkBilCloz:ZipCloz	0	Some concerns	Low risk	No concerns	Major concerns	No concerns	No concerns	Low	[Within-study bias, Imprecision]
GinkBilCloz:Zot	0	Some concerns	Low risk	No concerns	Major concerns	No concerns	No concerns	Low	[Within-study bias, Imprecision]
GinkBilHal:GlyCloz	0	Some concerns	Low risk	No concerns	Major concerns	No concerns	No concerns	Low	[Within-study bias, Imprecision]
GinkBilHal:HalCloz	0	Some concerns	Low risk	No concerns	Major concerns	No concerns	No concerns	Low	[Within-study bias, Imprecision]
GinkBilHal:LamCloz	0	Some concerns	Low risk	No concerns	Major concerns	No concerns	No concerns	Low	[Within-study bias, Imprecision]
GinkBilHal:Lev	0	Some concerns	Low risk	No concerns	Major concerns	No concerns	No concerns	Low	[Within-study bias, Imprecision]
GinkBilHal:LiHal	0	Some concerns	Low risk	No concerns	Major concerns	No concerns	No concerns	Low	[Within-study bias, Imprecision]
GinkBilHal:MemCloz	0	Some concerns	Low risk	No concerns	Major concerns	No concerns	No concerns	Low	[Within-study bias, Imprecision]
GinkBilHal:MetCloz	0	Some concerns	Low risk	No concerns	Major concerns	No concerns	No concerns	Low	[Within-study bias, Imprecision]

GinkBilHal:MinocCloz	0	Some concerns	Low risk	No concerns	Major concerns	No concerns	No concerns	Low	[Within-study bias, Imprecision]
GinkBilHal:MirtCloz	0	Some concerns	Low risk	No concerns	Some concerns	Some concerns	No concerns	Low	[Within-study bias, Imprecision, Heterogeneity]
GinkBilHal:MirtRisp	0	Some concerns	Low risk	No concerns	Major concerns	No concerns	No concerns	Low	[Within-study bias, Imprecision]
GinkBilHal:ModfCloz	0	Some concerns	Low risk	No concerns	Major concerns	No concerns	No concerns	Low	[Within-study bias, Imprecision]
GinkBilHal:Ola	0	Some concerns	Low risk	No concerns	Major concerns	No concerns	No concerns	Low	[Within-study bias, Imprecision]
GinkBilHal:OlaRisp	0	Some concerns	Low risk	No concerns	Major concerns	No concerns	No concerns	Low	[Within-study bias, Imprecision]
GinkBilHal:OndastHal	0	No concerns	Low risk	No concerns	Major concerns	No concerns	No concerns	Low	[Imprecision]
GinkBilHal:OxytRisp	0	Major concerns	Low risk	No concerns	No concerns	No concerns	No concerns	Low	[Within-study bias]
GinkBilHal:Palip	0	Some concerns	Low risk	No concerns	Major concerns	No concerns	No concerns	Low	[Within-study bias, Imprecision]
GinkBilHal:Palipla	0	Some concerns	Low risk	No concerns	Major concerns	No concerns	No concerns	Low	[Within-study bias, Imprecision]
GinkBilHal:PalipOla	0	Some concerns	Low risk	No concerns	Some concerns	Some concerns	No concerns	Low	[Within-study bias, Imprecision, Heterogeneity]
GinkBilHal:PalmitRisp	0	Major concerns	Low risk	No concerns	Major concerns	No concerns	No concerns	Low	[Within-study bias, Imprecision]
GinkBilHal:PhenylpropCloz	0	Some concerns	Low risk	No concerns	Major concerns	No concerns	No concerns	Low	[Within-study bias, Imprecision]
GinkBilHal:PimozCloz	0	Some concerns	Low risk	No concerns	Major concerns	No concerns	No concerns	Low	[Within-study bias, Imprecision]
GinkBilHal:PtxRisp	0	Major concerns	Low risk	No concerns	Major concerns	No concerns	No concerns	Low	[Within-study bias, Imprecision]
GinkBilHal:Que	0	Some concerns	Low risk	No concerns	Major concerns	No concerns	No concerns	Low	[Within-study bias, Imprecision]

GinkBilHal:QueCloz	0	Some concerns	Low risk	No concerns	Major concerns	No concerns	No concerns	Low	[Within-study bias, Imprecision]
GinkBilHal:ResverRisp	0	Major concerns	Low risk	No concerns	Major concerns	No concerns	No concerns	Low	[Within-study bias, Imprecision]
GinkBilHal:Risp	0	Some concerns	Low risk	No concerns	Major concerns	No concerns	No concerns	Low	[Within-study bias, Imprecision]
GinkBilHal:RispCloz	0	Some concerns	Low risk	No concerns	Major concerns	No concerns	No concerns	Low	[Within-study bias, Imprecision]
GinkBilHal:SarcCloz	0	Some concerns	Low risk	No concerns	Major concerns	No concerns	No concerns	Low	[Within-study bias, Imprecision]
GinkBilHal:SerOla	0	Some concerns	Low risk	No concerns	Major concerns	No concerns	No concerns	Low	[Within-study bias, Imprecision]
GinkBilHal:Sertind	0	Some concerns	Low risk	No concerns	Major concerns	No concerns	No concerns	Low	[Within-study bias, Imprecision]
GinkBilHal:SertindCloz	0	Some concerns	Low risk	No concerns	Major concerns	No concerns	No concerns	Low	[Within-study bias, Imprecision]
GinkBilHal:SerZip	0	Some concerns	Low risk	No concerns	Major concerns	No concerns	No concerns	Low	[Within-study bias, Imprecision]
GinkBilHal:Sul	0	Some concerns	Low risk	No concerns	Some concerns	Some concerns	No concerns	Low	[Within-study bias, Imprecision, Heterogeneity]
GinkBilHal:SulCloz	0	Some concerns	Low risk	No concerns	Major concerns	No concerns	No concerns	Low	[Within-study bias, Imprecision]
GinkBilHal:SulOla	0	Some concerns	Low risk	No concerns	Major concerns	No concerns	No concerns	Low	[Within-study bias, Imprecision]
GinkBilHal:TopirCloz	0	Some concerns	Low risk	No concerns	Major concerns	No concerns	No concerns	Low	[Within-study bias, Imprecision]
GinkBilHal:VitD3Cloz	0	Some concerns	Low risk	No concerns	Major concerns	No concerns	No concerns	Low	[Within-study bias, Imprecision]
GinkBilHal:VortRisp	0	Some concerns	Low risk	No concerns	Major concerns	No concerns	No concerns	Low	[Within-study bias, Imprecision]
GinkBilHal:Zip	0	Some concerns	Low risk	No concerns	Major concerns	No concerns	No concerns	Low	[Within-study bias, Imprecision]
GinkBilHal:ZipCloz	0	Some concerns	Low risk	No concerns	Major concerns	No concerns	No concerns	Low	[Within-study bias, Imprecision]

GinkBilHal:Zot	0	Some concerns	Low risk	No concerns	Major concerns	No concerns	No concerns	Low	[Within-study bias, Imprecision]
GlyCloz:Hal	0	Some concerns	Low risk	Some concerns	Major concerns	No concerns	No concerns	Low	[Within-study bias, Indirectness, Imprecision]
GlyCloz:HalCloz	0	Some concerns	Low risk	No concerns	Major concerns	No concerns	No concerns	Low	[Within-study bias, Imprecision]
GlyCloz:LamCloz	0	Some concerns	Low risk	Some concerns	Major concerns	No concerns	No concerns	Low	[Within-study bias, Indirectness, Imprecision]
GlyCloz:Lev	0	Some concerns	Low risk	No concerns	Major concerns	No concerns	No concerns	Low	[Within-study bias, Imprecision]
GlyCloz:LiHal	0	Some concerns	Low risk	Some concerns	Major concerns	No concerns	No concerns	Low	[Within-study bias, Indirectness, Imprecision]
GlyCloz:MemCloz	0	No concerns	Low risk	Some concerns	Major concerns	No concerns	No concerns	Low	[Indirectness, Imprecision]
GlyCloz:MetCloz	0	Some concerns	Low risk	Some concerns	Major concerns	No concerns	No concerns	Low	[Within-study bias, Indirectness, Imprecision]
GlyCloz:MinocCloz	0	No concerns	Low risk	No concerns	Major concerns	No concerns	No concerns	Low	[Imprecision]
GlyCloz:MirtCloz	0	Some concerns	Low risk	Some concerns	Major concerns	No concerns	No concerns	Low	[Within-study bias, Indirectness, Imprecision]
GlyCloz:MirtRisp	0	Some concerns	Low risk	Some concerns	Major concerns	No concerns	No concerns	Low	[Within-study bias, Indirectness, Imprecision]
GlyCloz:ModfCloz	0	No concerns	Low risk	Some concerns	Major concerns	No concerns	No concerns	Low	[Indirectness, Imprecision]
GlyCloz:Ola	0	Some concerns	Low risk	Some concerns	Major concerns	No concerns	No concerns	Low	[Within-study bias, Indirectness, Imprecision]
GlyCloz:OlaRisp	0	Some concerns	Low risk	Some concerns	Major concerns	No concerns	No concerns	Low	[Within-study bias, Indirectness, Imprecision]
GlyCloz:OndastHal	0	Some concerns	Low risk	No concerns	Major concerns	No concerns	No concerns	Low	[Within-study bias, Imprecision]
GlyCloz:OxytRisp	0	Major concerns	Low risk	Some concerns	No concerns	Some concerns	No concerns	Low	[Within-study bias, Indirectness, Heterogeneity]
GlyCloz:Palip	0	Some concerns	Low risk	Some concerns	Major concerns	No concerns	No concerns	Low	[Within-study bias, Indirectness, Imprecision]

GlyCloz:Palipla	0	Some concerns	Low risk	Some concerns	Major concerns	No concerns	No concerns	Low	[Within-study bias, Indirectness, Imprecision]
GlyCloz:PalipOla	0	Some concerns	Low risk	Some concerns	Major concerns	No concerns	No concerns	Low	[Within-study bias, Indirectness, Imprecision]
GlyCloz:PalmitRisp	0	Major concerns	Low risk	Some concerns	Major concerns	No concerns	No concerns	Very Low	[Within-study bias, Indirectness, Imprecision]
GlyCloz:Phenylprop Cloz	0	Some concerns	Low risk	Some concerns	Major concerns	No concerns	No concerns	Low	[Within-study bias, Indirectness, Imprecision]
GlyCloz:PimozCloz	0	No concerns	Low risk	No concerns	Major concerns	No concerns	No concerns	Low	[Imprecision]
GlyCloz:PtxRisp	0	Major concerns	Low risk	Some concerns	Major concerns	No concerns	No concerns	Very Low	[Within-study bias, Indirectness, Imprecision]
GlyCloz:Que	0	Some concerns	Low risk	Some concerns	Major concerns	No concerns	No concerns	Low	[Within-study bias, Indirectness, Imprecision]
GlyCloz:QueCloz	0	Some concerns	Low risk	No concerns	Major concerns	No concerns	No concerns	Low	[Within-study bias, Imprecision]
GlyCloz:ResverRisp	0	Major concerns	Low risk	Some concerns	Major concerns	No concerns	No concerns	Very Low	[Within-study bias, Indirectness, Imprecision]
GlyCloz:Risp	0	Some concerns	Low risk	Some concerns	Major concerns	No concerns	No concerns	Low	[Within-study bias, Indirectness, Imprecision]
GlyCloz:RispCloz	0	Some concerns	Low risk	No concerns	Major concerns	No concerns	No concerns	Low	[Within-study bias, Imprecision]
GlyCloz:SarcCloz	0	No concerns	Low risk	No concerns	Major concerns	No concerns	No concerns	Low	[Imprecision]
GlyCloz:SerOla	0	Some concerns	Low risk	Some concerns	Major concerns	No concerns	No concerns	Low	[Within-study bias, Indirectness, Imprecision]
GlyCloz:Sertind	0	Some concerns	Low risk	No concerns	Major concerns	No concerns	No concerns	Low	[Within-study bias, Imprecision]
GlyCloz:SertindCloz	0	Some concerns	Low risk	No concerns	Major concerns	No concerns	No concerns	Low	[Within-study bias, Imprecision]
GlyCloz:SerZip	0	Some concerns	Low risk	No concerns	Major concerns	No concerns	No concerns	Low	[Within-study bias, Imprecision]
GlyCloz:Sul	0	Some concerns	Low risk	No concerns	No concerns	Major concerns	No concerns	Low	[Within-study bias, Heterogeneity]

GlyCloz:SulCloz	0	Some concerns	Low risk	No concerns	Major concerns	No concerns	No concerns	Low	[Within-study bias, Imprecision]
GlyCloz:SulOla	0	Some concerns	Low risk	Some concerns	Major concerns	No concerns	No concerns	Low	[Within-study bias, Indirectness, Imprecision]
GlyCloz:TopirCloz	0	Some concerns	Low risk	No concerns	Major concerns	No concerns	No concerns	Low	[Within-study bias, Imprecision]
GlyCloz:VitD3Cloz	0	No concerns	Low risk	Some concerns	Major concerns	No concerns	No concerns	Low	[Indirectness, Imprecision]
GlyCloz:VortRisp	0	Some concerns	Low risk	Some concerns	Major concerns	No concerns	No concerns	Low	[Within-study bias, Indirectness, Imprecision]
GlyCloz:Zip	0	Some concerns	Low risk	Some concerns	Major concerns	No concerns	No concerns	Low	[Within-study bias, Indirectness, Imprecision]
GlyCloz:ZipCloz	0	Some concerns	Low risk	No concerns	Major concerns	No concerns	No concerns	Low	[Within-study bias, Imprecision]
GlyCloz:Zot	0	Some concerns	Low risk	Some concerns	Major concerns	No concerns	No concerns	Low	[Within-study bias, Indirectness, Imprecision]
Hal:HalCloz	0	Some concerns	Low risk	No concerns	Some concerns	Some concerns	No concerns	Low	[Within-study bias, Imprecision, Heterogeneity]
Hal:LamCloz	0	Some concerns	Low risk	No concerns	No concerns	No concerns	No concerns	Moderate	[Within-study bias]
Hal:Lev	0	Some concerns	Low risk	No concerns	Major concerns	No concerns	No concerns	Low	[Within-study bias, Imprecision]
Hal:MemCloz	0	No concerns	Low risk	No concerns	No concerns	No concerns	No concerns	High	
Hal:MetCloz	0	Some concerns	Low risk	Major concerns	No concerns	Major concerns	No concerns	Very Low	[Within-study bias, Indirectness, Heterogeneity]
Hal:MinocCloz	0	No concerns	Low risk	No concerns	Some concerns	Some concerns	No concerns	Moderate	[Imprecision, Heterogeneity]
Hal:MirtCloz	0	Some concerns	Low risk	Some concerns	No concerns	No concerns	No concerns	Moderate	[Within-study bias, Indirectness]
Hal:MirtRisp	0	Some concerns	Low risk	Major concerns	Major concerns	No concerns	No concerns	Very Low	[Within-study bias, Indirectness, Imprecision]
Hal:ModfCloz	0	No concerns	Low risk	Some concerns	Major concerns	No concerns	No concerns	Low	[Indirectness, Imprecision]

Hal:OlaRisp	0	Some concerns	Low risk	Some concerns	Some concerns	Some concerns	No concerns	Low	[Within-study bias, Indirectness, Imprecision, Heterogeneity]
Hal:OxytRisp	0	Major concerns	Low risk	Some concerns	No concerns	No concerns	No concerns	Low	[Within-study bias, Indirectness]
Hal:Palip	0	Some concerns	Low risk	No concerns	Major concerns	No concerns	No concerns	Low	[Within-study bias, Imprecision]
Hal:Palipla	0	Some concerns	Low risk	No concerns	Major concerns	No concerns	No concerns	Low	[Within-study bias, Imprecision]
Hal:PalipOla	0	No concerns	Low risk	Major concerns	No concerns	No concerns	No concerns	Low	[Indirectness]
Hal:PalmitRisp	0	Major concerns	Low risk	Major concerns	No concerns	No concerns	No concerns	Low	[Within-study bias, Indirectness]
Hal:PhenylpropCloz	0	Some concerns	Low risk	Major concerns	Major concerns	No concerns	No concerns	Very Low	[Within-study bias, Indirectness, Imprecision]
Hal:PimozCloz	0	No concerns	Low risk	No concerns	Major concerns	No concerns	No concerns	Low	[Imprecision]
Hal:PtxRisp	0	Major concerns	Low risk	Major concerns	Major concerns	No concerns	No concerns	Very Low	[Within-study bias, Indirectness, Imprecision]
Hal:QueCloz	0	Some concerns	Low risk	No concerns	Major concerns	No concerns	No concerns	Low	[Within-study bias, Imprecision]
Hal:ResverRisp	0	Major concerns	Low risk	Major concerns	No concerns	Some concerns	No concerns	Very Low	[Within-study bias, Indirectness, Heterogeneity]
Hal:RispCloz	0	Some concerns	Low risk	No concerns	No concerns	Some concerns	No concerns	Moderate	[Within-study bias, Heterogeneity]
Hal:SarcCloz	0	No concerns	Low risk	No concerns	Major concerns	No concerns	No concerns	Low	[Imprecision]
Hal:SerOla	0	Some concerns	Low risk	No concerns	Major concerns	No concerns	No concerns	Low	[Within-study bias, Imprecision]
Hal:Sertind	0	Some concerns	Low risk	No concerns	Major concerns	No concerns	No concerns	Low	[Within-study bias, Imprecision]
Hal:SertindCloz	0	Some concerns	Low risk	No concerns	Major concerns	No concerns	No concerns	Low	[Within-study bias, Imprecision]

Hal:SerZip	0	Some concerns	Low risk	No concerns	Major concerns	No concerns	No concerns	Low	[Within-study bias, Imprecision]
Hal:Sul	0	Some concerns	Low risk	No concerns	Major concerns	No concerns	No concerns	Low	[Within-study bias, Imprecision]
Hal:SulCloz	0	Some concerns	Low risk	No concerns	No concerns	No concerns	No concerns	Moderate	[Within-study bias]
Hal:SulOla	0	Major concerns	Low risk	No concerns	Major concerns	No concerns	No concerns	Low	[Within-study bias, Imprecision]
Hal:TopirCloz	0	Some concerns	Low risk	No concerns	No concerns	No concerns	No concerns	Moderate	[Within-study bias]
Hal:VitD3Cloz	0	No concerns	Low risk	Some concerns	Major concerns	No concerns	No concerns	Low	[Indirectness, Imprecision]
Hal:VortRisp	0	No concerns	Low risk	Major concerns	No concerns	No concerns	No concerns	Low	[Indirectness]
Hal:Zip	0	Some concerns	Low risk	No concerns	Major concerns	No concerns	No concerns	Low	[Within-study bias, Imprecision]
Hal:ZipCloz	0	Some concerns	Low risk	No concerns	No concerns	No concerns	No concerns	Moderate	[Within-study bias]
Hal:Zot	0	Some concerns	Low risk	Some concerns	Major concerns	No concerns	No concerns	Low	[Within-study bias, Indirectness, Imprecision]
HalCloz:LamCloz	0	Some concerns	Low risk	No concerns	Major concerns	No concerns	No concerns	Low	[Within-study bias, Imprecision]
HalCloz:Lev	0	Some concerns	Low risk	No concerns	Major concerns	No concerns	No concerns	Low	[Within-study bias, Imprecision]
HalCloz:LiHal	0	Some concerns	Low risk	No concerns	Major concerns	No concerns	No concerns	Low	[Within-study bias, Imprecision]
HalCloz:MemCloz	0	No concerns	Low risk	No concerns	Major concerns	No concerns	No concerns	Low	[Imprecision]
HalCloz:MetCloz	0	Some concerns	Low risk	No concerns	Major concerns	No concerns	No concerns	Low	[Within-study bias, Imprecision]
HalCloz:MinocCloz	0	No concerns	Low risk	No concerns	Major concerns	No concerns	No concerns	Low	[Imprecision]
HalCloz:MirtCloz	0	Some concerns	Low risk	No concerns	Major concerns	No concerns	No concerns	Low	[Within-study bias, Imprecision]

HalCloz:MirtRisp	0	Some concerns	Low risk	No concerns	Major concerns	No concerns	No concerns	Low	[Within-study bias, Imprecision]
HalCloz:ModfCloz	0	No concerns	Low risk	No concerns	Major concerns	No concerns	No concerns	Low	[Imprecision]
HalCloz:Ola	0	Some concerns	Low risk	No concerns	Major concerns	No concerns	No concerns	Low	[Within-study bias, Imprecision]
HalCloz:OlaRisp	0	Some concerns	Low risk	No concerns	Major concerns	No concerns	No concerns	Low	[Within-study bias, Imprecision]
HalCloz:OndastHal	0	Some concerns	Low risk	No concerns	Major concerns	No concerns	No concerns	Low	[Within-study bias, Imprecision]
HalCloz:OxytRisp	0	Major concerns	Low risk	No concerns	No concerns	No concerns	No concerns	Low	[Within-study bias]
HalCloz:Palip	0	Some concerns	Low risk	No concerns	Major concerns	No concerns	No concerns	Low	[Within-study bias, Imprecision]
HalCloz:Palipla	0	Some concerns	Low risk	No concerns	Major concerns	No concerns	No concerns	Low	[Within-study bias, Imprecision]
HalCloz:PalipOla	0	No concerns	Low risk	No concerns	Major concerns	No concerns	No concerns	Low	[Imprecision]
HalCloz:PalmitRisp	0	Major concerns	Low risk	No concerns	Major concerns	No concerns	No concerns	Low	[Within-study bias, Imprecision]
HalCloz:Phenylprop Cloz	0	Some concerns	Low risk	No concerns	Major concerns	No concerns	No concerns	Low	[Within-study bias, Imprecision]
HalCloz:PimozCloz	0	No concerns	Low risk	No concerns	Major concerns	No concerns	No concerns	Low	[Imprecision]
HalCloz:PtxRisp	0	Major concerns	Low risk	No concerns	Major concerns	No concerns	No concerns	Low	[Within-study bias, Imprecision]
HalCloz:Que	0	Some concerns	Low risk	No concerns	No concerns	Some concerns	No concerns	Moderate	[Within-study bias, Heterogeneity]
HalCloz:QueCloz	0	Major concerns	Low risk	No concerns	Some concerns	Some concerns	No concerns	Low	[Within-study bias, Imprecision, Heterogeneity]
HalCloz:ResverRisp	0	Major concerns	Low risk	No concerns	Major concerns	No concerns	No concerns	Low	[Within-study bias, Imprecision]
HalCloz:Risp	0	Some concerns	Low risk	No concerns	Major concerns	No concerns	No concerns	Low	[Within-study bias, Imprecision]

HalCloz:RispCloz	0	Some concerns	Low risk	No concerns	Major concerns	No concerns	No concerns	Low	[Within-study bias, Imprecision]
HalCloz:SarcCloz	0	No concerns	Low risk	No concerns	Major concerns	No concerns	No concerns	Low	[Imprecision]
HalCloz:SerOla	0	Some concerns	Low risk	No concerns	Major concerns	No concerns	No concerns	Low	[Within-study bias, Imprecision]
HalCloz:Sertind	0	Some concerns	Low risk	No concerns	Some concerns	Some concerns	No concerns	Low	[Within-study bias, Imprecision, Heterogeneity]
HalCloz:SertindCloz	0	Some concerns	Low risk	No concerns	Major concerns	No concerns	No concerns	Low	[Within-study bias, Imprecision]
HalCloz:SerZip	0	Some concerns	Low risk	No concerns	Major concerns	No concerns	No concerns	Low	[Within-study bias, Imprecision]
HalCloz:Sul	0	Some concerns	Low risk	No concerns	No concerns	No concerns	No concerns	Moderate	[Within-study bias]
HalCloz:SulCloz	0	Major concerns	Low risk	No concerns	Major concerns	No concerns	No concerns	Low	[Within-study bias, Imprecision]
HalCloz:SulOla	0	Major concerns	Low risk	No concerns	Major concerns	No concerns	No concerns	Low	[Within-study bias, Imprecision]
HalCloz:TopirCloz	0	Major concerns	Low risk	No concerns	Major concerns	No concerns	No concerns	Low	[Within-study bias, Imprecision]
HalCloz:VitD3Cloz	0	No concerns	Low risk	No concerns	Major concerns	No concerns	No concerns	Low	[Imprecision]
HalCloz:VortRisp	0	Some concerns	Low risk	No concerns	Major concerns	No concerns	No concerns	Low	[Within-study bias, Imprecision]
HalCloz:Zip	0	Some concerns	Low risk	No concerns	Major concerns	No concerns	No concerns	Low	[Within-study bias, Imprecision]
HalCloz:ZipCloz	0	No concerns	Low risk	No concerns	Major concerns	No concerns	No concerns	Low	[Imprecision]
HalCloz:Zot	0	Major concerns	Low risk	No concerns	Major concerns	No concerns	No concerns	Low	[Within-study bias, Imprecision]
LamCloz:Lev	0	No concerns	Low risk	No concerns	Major concerns	No concerns	No concerns	Low	[Imprecision]
LamCloz:LiHal	0	Some concerns	Low risk	No concerns	Major concerns	No concerns	No concerns	Low	[Within-study bias, Imprecision]

LamCloz:MemCloz	0	No concerns	Low risk	No concerns	Major concerns	No concerns	No concerns	Low	[Imprecision]
LamCloz:MetCloz	0	No concerns	Low risk	Major concerns	Major concerns	No concerns	No concerns	Low	[Indirectness, Imprecision]
LamCloz:MinocCloz	0	No concerns	Low risk	No concerns	Major concerns	No concerns	No concerns	Low	[Imprecision]
LamCloz:MirtCloz	0	Some concerns	Low risk	Some concerns	Major concerns	No concerns	No concerns	Low	[Within-study bias, Indirectness, Imprecision]
LamCloz:MirtRisp	0	Some concerns	Low risk	No concerns	Major concerns	No concerns	No concerns	Low	[Within-study bias, Imprecision]
LamCloz:ModfCloz	0	No concerns	Low risk	Some concerns	Major concerns	No concerns	No concerns	Low	[Indirectness, Imprecision]
LamCloz:Ola	0	No concerns	Low risk	No concerns	No concerns	Major concerns	No concerns	Low	[Heterogeneity]
LamCloz:OlaRisp	0	Some concerns	Low risk	No concerns	Major concerns	No concerns	No concerns	Low	[Within-study bias, Imprecision]
LamCloz:OndastHal	0	No concerns	Low risk	No concerns	Major concerns	No concerns	No concerns	Low	[Imprecision]
LamCloz:OxytRisp	0	Major concerns	Low risk	No concerns	No concerns	No concerns	No concerns	Low	[Within-study bias]
LamCloz:Palip	0	Some concerns	Low risk	No concerns	Major concerns	No concerns	No concerns	Low	[Within-study bias, Imprecision]
LamCloz:Palipla	0	Some concerns	Low risk	No concerns	Major concerns	No concerns	No concerns	Low	[Within-study bias, Imprecision]
LamCloz:PalipOla	0	No concerns	Low risk	No concerns	Major concerns	No concerns	No concerns	Low	[Imprecision]
LamCloz:PalmitRisp	0	Major concerns	Low risk	No concerns	Major concerns	No concerns	No concerns	Low	[Within-study bias, Imprecision]
LamCloz:PhenylpropCloz	0	Some concerns	Low risk	Major concerns	Major concerns	No concerns	No concerns	Very Low	[Within-study bias, Indirectness, Imprecision]
LamCloz:PimozCloz	0	No concerns	Low risk	No concerns	Some concerns	Some concerns	No concerns	Moderate	[Imprecision, Heterogeneity]
LamCloz:PtxRisp	0	Major concerns	Low risk	No concerns	Major concerns	No concerns	No concerns	Low	[Within-study bias, Imprecision]
LamCloz:Que	0	Some concerns	Low risk	No concerns	No concerns	No concerns	No concerns	Moderate	[Within-study bias]

LamCloz:QueCloz	0	Major concerns	Low risk	No concerns	No concerns	No concerns	No concerns	Low	[Within-study bias]
LamCloz:ResverRisp	0	Major concerns	Low risk	No concerns	Major concerns	No concerns	No concerns	Low	[Within-study bias, Imprecision]
LamCloz:Risp	0	Some concerns	Low risk	No concerns	No concerns	Some concerns	No concerns	Moderate	[Within-study bias, Heterogeneity]
LamCloz:RispCloz	0	No concerns	Low risk	No concerns	Major concerns	No concerns	No concerns	Low	[Imprecision]
LamCloz:SarcCloz	0	No concerns	Low risk	No concerns	Major concerns	No concerns	No concerns	Low	[Imprecision]
LamCloz:SerOla	0	Some concerns	Low risk	No concerns	Major concerns	No concerns	No concerns	Low	[Within-study bias, Imprecision]
LamCloz:Sertind	0	Some concerns	Low risk	No concerns	No concerns	No concerns	No concerns	Moderate	[Within-study bias]
LamCloz:SertindCloz	0	Some concerns	Low risk	No concerns	Major concerns	No concerns	No concerns	Low	[Within-study bias, Imprecision]
LamCloz:SerZip	0	No concerns	Low risk	No concerns	Major concerns	No concerns	No concerns	Low	[Imprecision]
LamCloz:Sul	0	Some concerns	Low risk	No concerns	No concerns	No concerns	No concerns	Moderate	[Within-study bias]
LamCloz:SulCloz	0	Major concerns	Low risk	No concerns	Major concerns	No concerns	No concerns	Low	[Within-study bias, Imprecision]
LamCloz:SulOla	0	No concerns	Low risk	No concerns	Major concerns	No concerns	No concerns	Low	[Imprecision]
LamCloz:TopirCloz	0	No concerns	Low risk	No concerns	Major concerns	No concerns	No concerns	Low	[Imprecision]
LamCloz:VitD3Cloz	0	No concerns	Low risk	Some concerns	Major concerns	No concerns	No concerns	Low	[Indirectness, Imprecision]
LamCloz:VortRisp	0	No concerns	Low risk	No concerns	Major concerns	No concerns	No concerns	Low	[Imprecision]
LamCloz:Zip	0	Some concerns	Low risk	No concerns	No concerns	Some concerns	No concerns	Moderate	[Within-study bias, Heterogeneity]
LamCloz:ZipCloz	0	No concerns	Low risk	No concerns	Major concerns	No concerns	No concerns	Low	[Imprecision]

LamCloz:Zot	0	Major concerns	Low risk	Some concerns	Major concerns	No concerns	No concerns	Very Low	[Within-study bias, Indirectness, Imprecision]
Lev:LiHal	0	Some concerns	Low risk	No concerns	Major concerns	No concerns	No concerns	Low	[Within-study bias, Imprecision]
Lev:MemCloz	0	No concerns	Low risk	No concerns	Major concerns	No concerns	No concerns	Low	[Imprecision]
Lev:MetCloz	0	No concerns	Low risk	No concerns	Major concerns	No concerns	No concerns	Low	[Imprecision]
Lev:MinocCloz	0	No concerns	Low risk	No concerns	Major concerns	No concerns	No concerns	Low	[Imprecision]
Lev:MirtCloz	0	Some concerns	Low risk	No concerns	Major concerns	No concerns	No concerns	Low	[Within-study bias, Imprecision]
Lev:MirtRisp	0	Some concerns	Low risk	No concerns	Major concerns	No concerns	No concerns	Low	[Within-study bias, Imprecision]
Lev:ModfCloz	0	No concerns	Low risk	No concerns	Major concerns	No concerns	No concerns	Low	[Imprecision]
Lev:Ola	0	No concerns	Low risk	No concerns	Major concerns	No concerns	No concerns	Low	[Imprecision]
Lev:OlaRisp	0	Some concerns	Low risk	No concerns	Major concerns	No concerns	No concerns	Low	[Within-study bias, Imprecision]
Lev:OndastHal	0	No concerns	Low risk	No concerns	Major concerns	No concerns	No concerns	Low	[Imprecision]
Lev:OxytRisp	0	Major concerns	Low risk	No concerns	No concerns	No concerns	No concerns	Low	[Within-study bias]
Lev:Palip	0	Some concerns	Low risk	No concerns	Major concerns	No concerns	No concerns	Low	[Within-study bias, Imprecision]
Lev:Palipla	0	Some concerns	Low risk	No concerns	Major concerns	No concerns	No concerns	Low	[Within-study bias, Imprecision]
Lev:PalipOla	0	No concerns	Low risk	No concerns	Major concerns	No concerns	No concerns	Low	[Imprecision]
Lev:PalmitRisp	0	Major concerns	Low risk	No concerns	Major concerns	No concerns	No concerns	Low	[Within-study bias, Imprecision]
Lev:PhenylpropCloz	0	Some concerns	Low risk	No concerns	Major concerns	No concerns	No concerns	Low	[Within-study bias, Imprecision]

Lev:PimozCloz	0	No concerns	Low risk	No concerns	Major concerns	No concerns	No concerns	Low	[Imprecision]
Lev:PtxRisp	0	Major concerns	Low risk	No concerns	Major concerns	No concerns	No concerns	Low	[Within-study bias, Imprecision]
Lev:Que	0	Some concerns	Low risk	No concerns	Major concerns	No concerns	No concerns	Low	[Within-study bias, Imprecision]
Lev:QueCloz	0	Some concerns	Low risk	No concerns	Major concerns	No concerns	No concerns	Low	[Within-study bias, Imprecision]
Lev:ResverRisp	0	Major concerns	Low risk	No concerns	Major concerns	No concerns	No concerns	Low	[Within-study bias, Imprecision]
Lev:Risp	0	Some concerns	Low risk	No concerns	Major concerns	No concerns	No concerns	Low	[Within-study bias, Imprecision]
Lev:RispCloz	0	No concerns	Low risk	No concerns	Major concerns	No concerns	No concerns	Low	[Imprecision]
Lev:SarcCloz	0	No concerns	Low risk	No concerns	Major concerns	No concerns	No concerns	Low	[Imprecision]
Lev:SerOla	0	Some concerns	Low risk	No concerns	Major concerns	No concerns	No concerns	Low	[Within-study bias, Imprecision]
Lev:Sertind	0	Some concerns	Low risk	No concerns	Major concerns	No concerns	No concerns	Low	[Within-study bias, Imprecision]
Lev:SertindCloz	0	Some concerns	Low risk	No concerns	Major concerns	No concerns	No concerns	Low	[Within-study bias, Imprecision]
Lev:SerZip	0	No concerns	Low risk	No concerns	Major concerns	No concerns	No concerns	Low	[Imprecision]
Lev:Sul	0	Some concerns	Low risk	No concerns	Some concerns	Some concerns	No concerns	Low	[Within-study bias, Imprecision, Heterogeneity]
Lev:SulCloz	0	No concerns	Low risk	No concerns	Major concerns	No concerns	No concerns	Low	[Imprecision]
Lev:SulOla	0	No concerns	Low risk	No concerns	Major concerns	No concerns	No concerns	Low	[Imprecision]
Lev:TopirCloz	0	No concerns	Low risk	No concerns	Major concerns	No concerns	No concerns	Low	[Imprecision]
Lev:VitD3Cloz	0	No concerns	Low risk	No concerns	Major concerns	No concerns	No concerns	Low	[Imprecision]

Lev:VortRisp	0	No concerns	Low risk	No concerns	Major concerns	No concerns	No concerns	Low	[Imprecision]
Lev:Zip	0	No concerns	Low risk	No concerns	Major concerns	No concerns	No concerns	Low	[Imprecision]
Lev:ZipCloz	0	No concerns	Low risk	No concerns	Major concerns	No concerns	No concerns	Low	[Imprecision]
Lev:Zot	0	No concerns	Low risk	No concerns	Major concerns	No concerns	No concerns	Low	[Imprecision]
LiHal:MemCloz	0	No concerns	Low risk	No concerns	Major concerns	No concerns	No concerns	Low	[Imprecision]
LiHal:MetCloz	0	Some concerns	Low risk	Major concerns	Major concerns	No concerns	No concerns	Very Low	[Within-study bias, Indirectness, Imprecision]
LiHal:MinocCloz	0	No concerns	Low risk	No concerns	Major concerns	No concerns	No concerns	Low	[Imprecision]
LiHal:MirtCloz	0	Some concerns	Low risk	Some concerns	Major concerns	No concerns	No concerns	Low	[Within-study bias, Indirectness, Imprecision]
LiHal:MirtRisp	0	Some concerns	Low risk	Major concerns	Major concerns	No concerns	No concerns	Very Low	[Within-study bias, Indirectness, Imprecision]
LiHal:ModfCloz	0	No concerns	Low risk	Some concerns	Major concerns	No concerns	No concerns	Low	[Indirectness, Imprecision]
LiHal:Ola	0	Some concerns	Low risk	Some concerns	No concerns	Major concerns	No concerns	Low	[Within-study bias, Indirectness, Heterogeneity]
LiHal:OlaRisp	0	Some concerns	Low risk	Some concerns	Major concerns	No concerns	No concerns	Low	[Within-study bias, Indirectness, Imprecision]
LiHal:OndastHal	0	No concerns	Low risk	No concerns	Major concerns	No concerns	No concerns	Low	[Imprecision]
LiHal:OxytRisp	0	Major concerns	Low risk	Some concerns	Major concerns	No concerns	No concerns	Very Low	[Within-study bias, Indirectness, Imprecision]
LiHal:Palip	0	Some concerns	Low risk	No concerns	Major concerns	No concerns	No concerns	Low	[Within-study bias, Imprecision]
LiHal:Palipla	0	Some concerns	Low risk	Some concerns	Major concerns	No concerns	No concerns	Low	[Within-study bias, Indirectness, Imprecision]
LiHal:PalipOla	0	No concerns	Low risk	Major concerns	Major concerns	No concerns	No concerns	Low	[Indirectness, Imprecision]

LiHal:PalmitRisp	0	Major concerns	Low risk	Major concerns	Major concerns	No concerns	No concerns	Very Low	[Within-study bias, Indirectness, Imprecision]
LiHal:PhenylpropCloz	0	Some concerns	Low risk	Major concerns	Major concerns	No concerns	No concerns	Very Low	[Within-study bias, Indirectness, Imprecision]
LiHal:PimozCloz	0	No concerns	Low risk	No concerns	No concerns	Major concerns	No concerns	Low	[Heterogeneity]
LiHal:PtxRisp	0	Major concerns	Low risk	Major concerns	Major concerns	No concerns	No concerns	Very Low	[Within-study bias, Indirectness, Imprecision]
LiHal:Que	0	Some concerns	Low risk	No concerns	No concerns	No concerns	No concerns	Moderate	[Within-study bias]
LiHal:QueCloz	0	Some concerns	Low risk	No concerns	No concerns	No concerns	No concerns	Moderate	[Within-study bias]
LiHal:ResverRisp	0	Major concerns	Low risk	Major concerns	Major concerns	No concerns	No concerns	Very Low	[Within-study bias, Indirectness, Imprecision]
LiHal:Risp	0	Some concerns	Low risk	Some concerns	No concerns	Some concerns	No concerns	Low	[Within-study bias, Indirectness, Heterogeneity]
LiHal:RispCloz	0	Some concerns	Low risk	No concerns	Major concerns	No concerns	No concerns	Low	[Within-study bias, Imprecision]
LiHal:SarcCloz	0	No concerns	Low risk	No concerns	Major concerns	No concerns	No concerns	Low	[Imprecision]
LiHal:SerOla	0	Some concerns	Low risk	No concerns	Major concerns	No concerns	No concerns	Low	[Within-study bias, Imprecision]
LiHal:Sertind	0	Some concerns	Low risk	No concerns	No concerns	No concerns	No concerns	Moderate	[Within-study bias]
LiHal:SertindCloz	0	Some concerns	Low risk	No concerns	Major concerns	No concerns	No concerns	Low	[Within-study bias, Imprecision]
LiHal:SerZip	0	Some concerns	Low risk	No concerns	Major concerns	No concerns	No concerns	Low	[Within-study bias, Imprecision]
LiHal:Sul	0	Some concerns	Low risk	No concerns	No concerns	No concerns	No concerns	Moderate	[Within-study bias]
LiHal:SulCloz	0	Some concerns	Low risk	No concerns	Major concerns	No concerns	No concerns	Low	[Within-study bias, Imprecision]
LiHal:SulOla	0	Some concerns	Low risk	No concerns	Major concerns	No concerns	No concerns	Low	[Within-study bias, Imprecision]

LiHal:TopirCloz	0	Some concerns	Low risk	No concerns	Major concerns	No concerns	No concerns	Low	[Within-study bias, Imprecision]
LiHal:VitD3Cloz	0	No concerns	Low risk	Some concerns	Major concerns	No concerns	No concerns	Low	[Indirectness, Imprecision]
LiHal:VortRisp	0	No concerns	Low risk	Major concerns	Major concerns	No concerns	No concerns	Low	[Indirectness, Imprecision]
LiHal:Zip	0	Some concerns	Low risk	No concerns	No concerns	No concerns	No concerns	Moderate	[Within-study bias]
LiHal:ZipCloz	0	No concerns	Low risk	No concerns	Major concerns	No concerns	No concerns	Low	[Imprecision]
LiHal:Zot	0	Some concerns	Low risk	Some concerns	Some concerns	Some concerns	No concerns	Low	[Within-study bias, Indirectness, Imprecision, Heterogeneity]
MemCloz:MetCloz	0	No concerns	Low risk	Major concerns	Major concerns	No concerns	No concerns	Low	[Indirectness, Imprecision]
MemCloz:MinocCloz	0	No concerns	Low risk	No concerns	Major concerns	No concerns	No concerns	Low	[Imprecision]
MemCloz:MirtCloz	0	No concerns	Low risk	Some concerns	Major concerns	No concerns	No concerns	Low	[Indirectness, Imprecision]
MemCloz:MirtRisp	0	Some concerns	Low risk	No concerns	Major concerns	No concerns	No concerns	Low	[Within-study bias, Imprecision]
MemCloz:ModfCloz	0	No concerns	Low risk	Some concerns	Major concerns	No concerns	No concerns	Low	[Indirectness, Imprecision]
MemCloz:Ola	0	No concerns	Low risk	No concerns	No concerns	Major concerns	No concerns	Low	[Heterogeneity]
MemCloz:OlaRisp	0	Some concerns	Low risk	Some concerns	Major concerns	No concerns	No concerns	Low	[Within-study bias, Indirectness, Imprecision]
MemCloz:OndastHal	0	No concerns	Low risk	No concerns	Major concerns	No concerns	No concerns	Low	[Imprecision]
MemCloz:OxytRisp	0	Major concerns	Low risk	Some concerns	Major concerns	No concerns	No concerns	Very Low	[Within-study bias, Indirectness, Imprecision]
MemCloz:Palip	0	Some concerns	Low risk	No concerns	Major concerns	No concerns	No concerns	Low	[Within-study bias, Imprecision]
MemCloz:Palipla	0	Some concerns	Low risk	No concerns	Major concerns	No concerns	No concerns	Low	[Within-study bias, Imprecision]

MemCloz:PalipOla	0	No concerns	Low risk	Major concerns	Major concerns	No concerns	No concerns	Low	[Indirectness, Imprecision]
MemCloz:PalmitRisp	0	Major concerns	Low risk	No concerns	Major concerns	No concerns	No concerns	Low	[Within-study bias, Imprecision]
MemCloz:PhenylpropCloz	0	No concerns	Low risk	Major concerns	Major concerns	No concerns	No concerns	Low	[Indirectness, Imprecision]
MemCloz:PimozCloz	0	No concerns	Low risk	No concerns	No concerns	Major concerns	No concerns	Low	[Heterogeneity]
MemCloz:PtxRisp	0	Major concerns	Low risk	No concerns	Major concerns	No concerns	No concerns	Low	[Within-study bias, Imprecision]
MemCloz:Que	0	No concerns	Low risk	No concerns	No concerns	No concerns	No concerns	High	
MemCloz:QueCloz	0	Major concerns	Low risk	No concerns	No concerns	No concerns	No concerns	Low	[Within-study bias]
MemCloz:ResverRisp	0	Major concerns	Low risk	No concerns	Major concerns	No concerns	No concerns	Low	[Within-study bias, Imprecision]
MemCloz:Risp	0	No concerns	Low risk	No concerns	No concerns	Some concerns	No concerns	Moderate	[Heterogeneity]
MemCloz:RispCloz	0	No concerns	Low risk	No concerns	Major concerns	No concerns	No concerns	Low	[Imprecision]
MemCloz:SarcCloz	0	No concerns	Low risk	No concerns	Major concerns	No concerns	No concerns	Low	[Imprecision]
MemCloz:SerOla	0	Some concerns	Low risk	No concerns	Major concerns	No concerns	No concerns	Low	[Within-study bias, Imprecision]
MemCloz:Sertind	0	Some concerns	Low risk	No concerns	No concerns	No concerns	No concerns	Moderate	[Within-study bias]
MemCloz:SertindCloz	0	No concerns	Low risk	No concerns	Major concerns	No concerns	No concerns	Low	[Imprecision]
MemCloz:SerZip	0	No concerns	Low risk	No concerns	Major concerns	No concerns	No concerns	Low	[Imprecision]
MemCloz:Sul	0	No concerns	Low risk	No concerns	No concerns	No concerns	No concerns	High	
MemCloz:SulCloz	0	No concerns	Low risk	No concerns	Major concerns	No concerns	No concerns	Low	[Imprecision]
MemCloz:SulOla	0	No concerns	Low risk	No concerns	Major concerns	No concerns	No concerns	Low	[Imprecision]
MemCloz:TopirCloz	0	No concerns	Low risk	No concerns	Major concerns	No concerns	No concerns	Low	[Imprecision]

MemCloz:VitD3Cloz	0	No concerns	Low risk	Some concerns	Major concerns	No concerns	No concerns	Low	[Indirectness, Imprecision]
MemCloz:VortRisp	0	No concerns	Low risk	No concerns	Major concerns	No concerns	No concerns	Low	[Imprecision]
MemCloz:Zip	0	No concerns	Low risk	No concerns	No concerns	No concerns	No concerns	High	
MemCloz:ZipCloz	0	No concerns	Low risk	No concerns	Major concerns	No concerns	No concerns	Low	[Imprecision]
MemCloz:Zot	0	No concerns	Low risk	Some concerns	Some concerns	Some concerns	No concerns	Low	[Indirectness, Imprecision, Heterogeneity]
MetCloz:MinocCloz	0	No concerns	Low risk	No concerns	Major concerns	No concerns	No concerns	Low	[Imprecision]
MetCloz:MirtCloz	0	Some concerns	Low risk	Some concerns	Major concerns	No concerns	No concerns	Low	[Within-study bias, Indirectness, Imprecision]
MetCloz:MirtRisp	0	Some concerns	Low risk	Major concerns	Major concerns	No concerns	No concerns	Very Low	[Within-study bias, Indirectness, Imprecision]
MetCloz:ModfCloz	0	No concerns	Low risk	Some concerns	Major concerns	No concerns	No concerns	Low	[Indirectness, Imprecision]
MetCloz:Ola	0	No concerns	Low risk	Major concerns	Major concerns	No concerns	No concerns	Low	[Indirectness, Imprecision]
MetCloz:OlaRisp	0	Some concerns	Low risk	Some concerns	Major concerns	No concerns	No concerns	Low	[Within-study bias, Indirectness, Imprecision]
MetCloz:OndastHal	0	No concerns	Low risk	No concerns	Major concerns	No concerns	No concerns	Low	[Imprecision]
MetCloz:OxytRisp	0	Major concerns	Low risk	Some concerns	No concerns	Some concerns	No concerns	Low	[Within-study bias, Indirectness, Heterogeneity]
MetCloz:Palip	0	Some concerns	Low risk	No concerns	Major concerns	No concerns	No concerns	Low	[Within-study bias, Imprecision]
MetCloz:Palipla	0	Some concerns	Low risk	Some concerns	Major concerns	No concerns	No concerns	Low	[Within-study bias, Indirectness, Imprecision]
MetCloz:PalipOla	0	No concerns	Low risk	Major concerns	Major concerns	No concerns	No concerns	Low	[Indirectness, Imprecision]
MetCloz:PalmitRisp	0	Major concerns	Low risk	Major concerns	Major concerns	No concerns	No concerns	Very Low	[Within-study bias, Indirectness, Imprecision]

MetCloz:PhenylpropCloz	0	Some concerns	Low risk	Major concerns	Major concerns	No concerns	No concerns	Very Low	[Within-study bias, Indirectness, Imprecision]
MetCloz:PimozCloz	0	No concerns	Low risk	No concerns	Major concerns	No concerns	No concerns	Low	[Imprecision]
MetCloz:PtxRisp	0	Major concerns	Low risk	Major concerns	Major concerns	No concerns	No concerns	Very Low	[Within-study bias, Indirectness, Imprecision]
MetCloz:Que	0	Some concerns	Low risk	No concerns	No concerns	No concerns	No concerns	Moderate	[Within-study bias]
MetCloz:QueCloz	0	Major concerns	Low risk	No concerns	No concerns	Some concerns	No concerns	Low	[Within-study bias, Heterogeneity]
MetCloz:ResverRisp	0	Major concerns	Low risk	Major concerns	Major concerns	No concerns	No concerns	Very Low	[Within-study bias, Indirectness, Imprecision]
MetCloz:Risp	0	Some concerns	Low risk	Major concerns	Some concerns	Some concerns	No concerns	Very Low	[Within-study bias, Indirectness, Imprecision, Heterogeneity]
MetCloz:RispCloz	0	No concerns	Low risk	No concerns	Major concerns	No concerns	No concerns	Low	[Imprecision]
MetCloz:SarcCloz	0	No concerns	Low risk	No concerns	Major concerns	No concerns	No concerns	Low	[Imprecision]
MetCloz:SerOla	0	Some concerns	Low risk	No concerns	Major concerns	No concerns	No concerns	Low	[Within-study bias, Imprecision]
MetCloz:Sertind	0	Some concerns	Low risk	No concerns	No concerns	Major concerns	No concerns	Low	[Within-study bias, Heterogeneity]
MetCloz:SertindCloz	0	Some concerns	Low risk	No concerns	Major concerns	No concerns	No concerns	Low	[Within-study bias, Imprecision]
MetCloz:SerZip	0	No concerns	Low risk	No concerns	Major concerns	No concerns	No concerns	Low	[Imprecision]
MetCloz:Sul	0	Some concerns	Low risk	No concerns	No concerns	No concerns	No concerns	Moderate	[Within-study bias]
MetCloz:SulCloz	0	Major concerns	Low risk	No concerns	Major concerns	No concerns	No concerns	Low	[Within-study bias, Imprecision]
MetCloz:SulOla	0	No concerns	Low risk	No concerns	Major concerns	No concerns	No concerns	Low	[Imprecision]
MetCloz:TopirCloz	0	No concerns	Low risk	No concerns	Major concerns	No concerns	No concerns	Low	[Imprecision]

MetCloz:VitD3Cloz	0	No concerns	Low risk	Some concerns	Major concerns	No concerns	No concerns	Low	[Indirectness, Imprecision]
MetCloz:VortRisp	0	No concerns	Low risk	Major concerns	Major concerns	No concerns	No concerns	Low	[Indirectness, Imprecision]
MetCloz:Zip	0	Some concerns	Low risk	Major concerns	Some concerns	Some concerns	No concerns	Very Low	[Within-study bias, Indirectness, Imprecision, Heterogeneity]
MetCloz:ZipCloz	0	No concerns	Low risk	No concerns	Major concerns	No concerns	No concerns	Low	[Imprecision]
MetCloz:Zot	0	Major concerns	Low risk	Some concerns	Major concerns	No concerns	No concerns	Very Low	[Within-study bias, Indirectness, Imprecision]
MinocCloz:MirtCloz	0	No concerns	Low risk	No concerns	Major concerns	No concerns	No concerns	Low	[Imprecision]
MinocCloz:MirtRisp	0	Some concerns	Low risk	No concerns	Major concerns	No concerns	No concerns	Low	[Within-study bias, Imprecision]
MinocCloz:ModfCloz	0	No concerns	Low risk	No concerns	Major concerns	No concerns	No concerns	Low	[Imprecision]
MinocCloz:Ola	0	No concerns	Low risk	No concerns	Major concerns	No concerns	No concerns	Low	[Imprecision]
MinocCloz:OlaRisp	0	Some concerns	Low risk	No concerns	Major concerns	No concerns	No concerns	Low	[Within-study bias, Imprecision]
MinocCloz:OndastHal	0	No concerns	Low risk	No concerns	Major concerns	No concerns	No concerns	Low	[Imprecision]
MinocCloz:OxytRisp	0	Major concerns	Low risk	No concerns	Some concerns	Some concerns	No concerns	Low	[Within-study bias, Imprecision, Heterogeneity]
MinocCloz:Palip	0	Some concerns	Low risk	No concerns	Major concerns	No concerns	No concerns	Low	[Within-study bias, Imprecision]
MinocCloz:Palipla	0	Some concerns	Low risk	No concerns	Major concerns	No concerns	No concerns	Low	[Within-study bias, Imprecision]
MinocCloz:PalipOla	0	No concerns	Low risk	No concerns	Major concerns	No concerns	No concerns	Low	[Imprecision]
MinocCloz:PalmitRisp	0	Major concerns	Low risk	No concerns	Major concerns	No concerns	No concerns	Low	[Within-study bias, Imprecision]

MinocCloz:PhenylpropCloz	0	No concerns	Low risk	No concerns	Major concerns	No concerns	No concerns	Low	[Imprecision]
MinocCloz:PimozCloz	0	No concerns	Low risk	No concerns	Major concerns	No concerns	No concerns	Low	[Imprecision]
MinocCloz:PtxRisp	0	Major concerns	Low risk	No concerns	Major concerns	No concerns	No concerns	Low	[Within-study bias, Imprecision]
MinocCloz:Que	0	No concerns	Low risk	No concerns	No concerns	Some concerns	No concerns	Moderate	[Heterogeneity]
MinocCloz:QueCloz	0	Major concerns	Low risk	No concerns	No concerns	Some concerns	No concerns	Low	[Within-study bias, Heterogeneity]
MinocCloz:ResverRisp	0	Major concerns	Low risk	No concerns	Major concerns	No concerns	No concerns	Low	[Within-study bias, Imprecision]
MinocCloz:Risp	0	No concerns	Low risk	No concerns	Major concerns	No concerns	No concerns	Low	[Imprecision]
MinocCloz:RispCloz	0	No concerns	Low risk	No concerns	Major concerns	No concerns	No concerns	Low	[Imprecision]
MinocCloz:SarcCloz	0	No concerns	Low risk	No concerns	Major concerns	No concerns	No concerns	Low	[Imprecision]
MinocCloz:SerOla	0	Some concerns	Low risk	No concerns	Major concerns	No concerns	No concerns	Low	[Within-study bias, Imprecision]
MinocCloz:Sertind	0	Some concerns	Low risk	No concerns	No concerns	Major concerns	No concerns	Low	[Within-study bias, Heterogeneity]
MinocCloz:SertindCloz	0	No concerns	Low risk	No concerns	Major concerns	No concerns	No concerns	Low	[Imprecision]
MinocCloz:SerZip	0	No concerns	Low risk	No concerns	Major concerns	No concerns	No concerns	Low	[Imprecision]
MinocCloz:Sul	0	No concerns	Low risk	No concerns	No concerns	No concerns	No concerns	High	
MinocCloz:SulCloz	0	No concerns	Low risk	No concerns	Major concerns	No concerns	No concerns	Low	[Imprecision]
MinocCloz:SulOla	0	No concerns	Low risk	No concerns	Major concerns	No concerns	No concerns	Low	[Imprecision]
MinocCloz:TopirCloz	0	No concerns	Low risk	No concerns	Major concerns	No concerns	No concerns	Low	[Imprecision]
MinocCloz:VitD3Cloz	0	No concerns	Low risk	No concerns	Major concerns	No concerns	No concerns	Low	[Imprecision]

MinocCloz:VortRisp	0	No concerns	Low risk	No concerns	Major concerns	No concerns	No concerns	Low	[Imprecision]
MinocCloz:Zip	0	No concerns	Low risk	No concerns	Some concerns	Some concerns	No concerns	Moderate	[Imprecision, Heterogeneity]
MinocCloz:ZipCloz	0	No concerns	Low risk	No concerns	Major concerns	No concerns	No concerns	Low	[Imprecision]
MinocCloz:Zot	0	No concerns	Low risk	No concerns	Major concerns	No concerns	No concerns	Low	[Imprecision]
MirtCloz:MirtRisp	0	Some concerns	Low risk	Some concerns	Major concerns	No concerns	No concerns	Low	[Within-study bias, Indirectness, Imprecision]
MirtCloz:ModfCloz	0	No concerns	Low risk	Some concerns	Major concerns	No concerns	No concerns	Low	[Indirectness, Imprecision]
MirtCloz:Ola	0	Some concerns	Low risk	Some concerns	No concerns	No concerns	No concerns	Moderate	[Within-study bias, Indirectness]
MirtCloz:OlaRisp	0	Some concerns	Low risk	Some concerns	Major concerns	No concerns	No concerns	Low	[Within-study bias, Indirectness, Imprecision]
MirtCloz:OndastHal	0	Some concerns	Low risk	No concerns	Major concerns	No concerns	No concerns	Low	[Within-study bias, Imprecision]
MirtCloz:OxytRisp	0	Major concerns	Low risk	Some concerns	Major concerns	No concerns	No concerns	Very Low	[Within-study bias, Indirectness, Imprecision]
MirtCloz:Palip	0	Some concerns	Low risk	Some concerns	Major concerns	No concerns	No concerns	Low	[Within-study bias, Indirectness, Imprecision]
MirtCloz:Palipla	0	Some concerns	Low risk	Some concerns	No concerns	Some concerns	No concerns	Low	[Within-study bias, Indirectness, Heterogeneity]
MirtCloz:PalipOla	0	Some concerns	Low risk	Some concerns	Major concerns	No concerns	No concerns	Low	[Within-study bias, Indirectness, Imprecision]
MirtCloz:PalmitRisp	0	Major concerns	Low risk	Some concerns	Major concerns	No concerns	No concerns	Very Low	[Within-study bias, Indirectness, Imprecision]
MirtCloz:PhenylpropCloz	0	Some concerns	Low risk	Some concerns	Major concerns	No concerns	No concerns	Low	[Within-study bias, Indirectness, Imprecision]
MirtCloz:PimozCloz	0	No concerns	Low risk	No concerns	No concerns	No concerns	No concerns	High	
MirtCloz:PtxRisp	0	Major concerns	Low risk	Some concerns	Major concerns	No concerns	No concerns	Very Low	[Within-study bias, Indirectness, Imprecision]

MirtCloz:Que	0	Some concerns	Low risk	Some concerns	No concerns	No concerns	No concerns	Moderate	[Within-study bias, Indirectness]
MirtCloz:QueCloz	0	Some concerns	Low risk	No concerns	No concerns	No concerns	No concerns	Moderate	[Within-study bias]
MirtCloz:ResverRisp	0	Major concerns	Low risk	Some concerns	Major concerns	No concerns	No concerns	Very Low	[Within-study bias, Indirectness, Imprecision]
MirtCloz:Risp	0	Some concerns	Low risk	Some concerns	No concerns	No concerns	No concerns	Moderate	[Within-study bias, Indirectness]
MirtCloz:RispCloz	0	Some concerns	Low risk	No concerns	Major concerns	No concerns	No concerns	Low	[Within-study bias, Imprecision]
MirtCloz:SarcCloz	0	No concerns	Low risk	No concerns	No concerns	Major concerns	No concerns	Low	[Heterogeneity]
MirtCloz:SerOla	0	Some concerns	Low risk	Some concerns	Some concerns	Some concerns	No concerns	Low	[Within-study bias, Indirectness, Imprecision, Heterogeneity]
MirtCloz:Sertind	0	Some concerns	Low risk	No concerns	No concerns	No concerns	No concerns	Moderate	[Within-study bias]
MirtCloz:SertindCloz	0	Some concerns	Low risk	No concerns	Major concerns	No concerns	No concerns	Low	[Within-study bias, Imprecision]
MirtCloz:SerZip	0	Some concerns	Low risk	No concerns	Major concerns	No concerns	No concerns	Low	[Within-study bias, Imprecision]
MirtCloz:Sul	0	Some concerns	Low risk	No concerns	No concerns	No concerns	No concerns	Moderate	[Within-study bias]
MirtCloz:SulCloz	0	Some concerns	Low risk	No concerns	Major concerns	No concerns	No concerns	Low	[Within-study bias, Imprecision]
MirtCloz:SulOla	0	Some concerns	Low risk	Some concerns	Major concerns	No concerns	No concerns	Low	[Within-study bias, Indirectness, Imprecision]
MirtCloz:TopirCloz	0	Some concerns	Low risk	No concerns	Major concerns	No concerns	No concerns	Low	[Within-study bias, Imprecision]
MirtCloz:VitD3Cloz	0	No concerns	Low risk	Some concerns	No concerns	Some concerns	No concerns	Moderate	[Indirectness, Heterogeneity]
MirtCloz:VortRisp	0	Some concerns	Low risk	Some concerns	Major concerns	No concerns	No concerns	Low	[Within-study bias, Indirectness, Imprecision]
MirtCloz:Zip	0	Some concerns	Low risk	Some concerns	No concerns	No concerns	No concerns	Moderate	[Within-study bias, Indirectness]

MirtCloz:ZipCloz	0	Some concerns	Low risk	No concerns	Major concerns	No concerns	No concerns	Low	[Within-study bias, Imprecision]
MirtCloz:Zot	0	Some concerns	Low risk	Some concerns	No concerns	No concerns	No concerns	Moderate	[Within-study bias, Indirectness]
MirtRisp:ModfCloz	0	Some concerns	Low risk	Some concerns	Major concerns	No concerns	No concerns	Low	[Within-study bias, Indirectness, Imprecision]
MirtRisp:Ola	0	Some concerns	Low risk	Major concerns	Major concerns	No concerns	No concerns	Very Low	[Within-study bias, Indirectness, Imprecision]
MirtRisp:OlaRisp	0	Some concerns	Low risk	Some concerns	Major concerns	No concerns	No concerns	Low	[Within-study bias, Indirectness, Imprecision]
MirtRisp:OndastHal	0	Some concerns	Low risk	No concerns	Major concerns	No concerns	No concerns	Low	[Within-study bias, Imprecision]
MirtRisp:OxytRisp	0	Major concerns	Low risk	Some concerns	Major concerns	No concerns	No concerns	Very Low	[Within-study bias, Indirectness, Imprecision]
MirtRisp:Palip	0	Some concerns	Low risk	No concerns	Major concerns	No concerns	No concerns	Low	[Within-study bias, Imprecision]
MirtRisp:Palipla	0	Some concerns	Low risk	Some concerns	Major concerns	No concerns	No concerns	Low	[Within-study bias, Indirectness, Imprecision]
MirtRisp:PalipOla	0	Some concerns	Low risk	Major concerns	Major concerns	No concerns	No concerns	Very Low	[Within-study bias, Indirectness, Imprecision]
MirtRisp:PalmitRisp	0	Major concerns	Low risk	Major concerns	Major concerns	No concerns	No concerns	Very Low	[Within-study bias, Indirectness, Imprecision]
MirtRisp:Phenylprop Cloz	0	Some concerns	Low risk	Major concerns	Major concerns	No concerns	No concerns	Very Low	[Within-study bias, Indirectness, Imprecision]
MirtRisp:PimozCloz	0	Some concerns	Low risk	No concerns	Major concerns	No concerns	No concerns	Low	[Within-study bias, Imprecision]
MirtRisp:PtxRisp	0	Major concerns	Low risk	Major concerns	Major concerns	No concerns	No concerns	Very Low	[Within-study bias, Indirectness, Imprecision]
MirtRisp:Que	0	Some concerns	Low risk	No concerns	Some concerns	Some concerns	No concerns	Low	[Within-study bias, Imprecision, Heterogeneity]
MirtRisp:QueCloz	0	Some concerns	Low risk	No concerns	Major concerns	No concerns	No concerns	Low	[Within-study bias, Imprecision]
MirtRisp:ResverRisp	0	Major concerns	Low risk	Major concerns	Major concerns	No concerns	No concerns	Very Low	[Within-study bias, Indirectness, Imprecision]

MirtRisp:RispCloz	0	Some concerns	Low risk	No concerns	Major concerns	No concerns	No concerns	Low	[Within-study bias, Imprecision]
MirtRisp:SarcCloz	0	Some concerns	Low risk	No concerns	Major concerns	No concerns	No concerns	Low	[Within-study bias, Imprecision]
MirtRisp:SerOla	0	Some concerns	Low risk	No concerns	Major concerns	No concerns	No concerns	Low	[Within-study bias, Imprecision]
MirtRisp:Sertind	0	Some concerns	Low risk	No concerns	Major concerns	No concerns	No concerns	Low	[Within-study bias, Imprecision]
MirtRisp:SertindCloz	0	Some concerns	Low risk	No concerns	Major concerns	No concerns	No concerns	Low	[Within-study bias, Imprecision]
MirtRisp:SerZip	0	Some concerns	Low risk	No concerns	Major concerns	No concerns	No concerns	Low	[Within-study bias, Imprecision]
MirtRisp:Sul	0	Some concerns	Low risk	No concerns	No concerns	No concerns	No concerns	Moderate	[Within-study bias]
MirtRisp:SulCloz	0	Some concerns	Low risk	No concerns	Major concerns	No concerns	No concerns	Low	[Within-study bias, Imprecision]
MirtRisp:SulOla	0	Some concerns	Low risk	No concerns	Major concerns	No concerns	No concerns	Low	[Within-study bias, Imprecision]
MirtRisp:TopirCloz	0	Some concerns	Low risk	No concerns	Major concerns	No concerns	No concerns	Low	[Within-study bias, Imprecision]
MirtRisp:VitD3Cloz	0	Some concerns	Low risk	Some concerns	Major concerns	No concerns	No concerns	Low	[Within-study bias, Indirectness, Imprecision]
MirtRisp:VortRisp	0	No concerns	Low risk	Major concerns	Major concerns	No concerns	No concerns	Low	[Indirectness, Imprecision]
MirtRisp:Zip	0	Some concerns	Low risk	No concerns	Major concerns	No concerns	No concerns	Low	[Within-study bias, Imprecision]
MirtRisp:ZipCloz	0	Some concerns	Low risk	No concerns	Major concerns	No concerns	No concerns	Low	[Within-study bias, Imprecision]
MirtRisp:Zot	0	Some concerns	Low risk	Some concerns	Major concerns	No concerns	No concerns	Low	[Within-study bias, Indirectness, Imprecision]
ModfCloz:Ola	0	No concerns	Low risk	Some concerns	Major concerns	No concerns	No concerns	Low	[Indirectness, Imprecision]
ModfCloz:OlaRisp	0	Some concerns	Low risk	Some concerns	Major concerns	No concerns	No concerns	Low	[Within-study bias, Indirectness, Imprecision]

ModfCloz:OndastHal	0	No concerns	Low risk	No concerns	Major concerns	No concerns	No concerns	Low	[Imprecision]
ModfCloz:OxytRisp	0	Major concerns	Low risk	Some concerns	No concerns	No concerns	No concerns	Low	[Within-study bias, Indirectness]
ModfCloz:Palip	0	Some concerns	Low risk	Some concerns	Major concerns	No concerns	No concerns	Low	[Within-study bias, Indirectness, Imprecision]
ModfCloz:Palipla	0	Some concerns	Low risk	Some concerns	Major concerns	No concerns	No concerns	Low	[Within-study bias, Indirectness, Imprecision]
ModfCloz:PalipOla	0	No concerns	Low risk	Some concerns	Major concerns	No concerns	No concerns	Low	[Indirectness, Imprecision]
ModfCloz:PalmitRisp	0	Major concerns	Low risk	Some concerns	Major concerns	No concerns	No concerns	Very Low	[Within-study bias, Indirectness, Imprecision]
ModfCloz:PhenylpropCloz	0	No concerns	Low risk	Some concerns	Major concerns	No concerns	No concerns	Low	[Indirectness, Imprecision]
ModfCloz:PimozCloz	0	No concerns	Low risk	No concerns	Major concerns	No concerns	No concerns	Low	[Imprecision]
ModfCloz:PtxRisp	0	Major concerns	Low risk	Some concerns	Major concerns	No concerns	No concerns	Very Low	[Within-study bias, Indirectness, Imprecision]
ModfCloz:Que	0	No concerns	Low risk	Some concerns	Major concerns	No concerns	No concerns	Low	[Indirectness, Imprecision]
ModfCloz:QueCloz	0	Major concerns	Low risk	No concerns	Major concerns	No concerns	No concerns	Low	[Within-study bias, Imprecision]
ModfCloz:ResverRisp	0	Major concerns	Low risk	Some concerns	Major concerns	No concerns	No concerns	Very Low	[Within-study bias, Indirectness, Imprecision]
ModfCloz:Risp	0	No concerns	Low risk	Some concerns	Major concerns	No concerns	No concerns	Low	[Indirectness, Imprecision]
ModfCloz:RispCloz	0	No concerns	Low risk	No concerns	Major concerns	No concerns	No concerns	Low	[Imprecision]
ModfCloz:SarcCloz	0	No concerns	Low risk	No concerns	Major concerns	No concerns	No concerns	Low	[Imprecision]
ModfCloz:SerOla	0	Some concerns	Low risk	Some concerns	Major concerns	No concerns	No concerns	Low	[Within-study bias, Indirectness, Imprecision]
ModfCloz:Sertind	0	Some concerns	Low risk	No concerns	Major concerns	No concerns	No concerns	Low	[Within-study bias, Imprecision]

ModfCloz:SertindCloz	0	No concerns	Low risk	No concerns	Major concerns	No concerns	No concerns	Low	[Imprecision]
ModfCloz:SerZip	0	No concerns	Low risk	No concerns	Major concerns	No concerns	No concerns	Low	[Imprecision]
ModfCloz:Sul	0	No concerns	Low risk	No concerns	No concerns	Major concerns	No concerns	Low	[Heterogeneity]
ModfCloz:SulCloz	0	No concerns	Low risk	No concerns	Major concerns	No concerns	No concerns	Low	[Imprecision]
ModfCloz:SulOla	0	No concerns	Low risk	Some concerns	Major concerns	No concerns	No concerns	Low	[Indirectness, Imprecision]
ModfCloz:TopirCloz	0	No concerns	Low risk	No concerns	Major concerns	No concerns	No concerns	Low	[Imprecision]
ModfCloz:VitD3Cloz	0	No concerns	Low risk	Some concerns	Major concerns	No concerns	No concerns	Low	[Indirectness, Imprecision]
ModfCloz:VortRisp	0	No concerns	Low risk	Some concerns	Major concerns	No concerns	No concerns	Low	[Indirectness, Imprecision]
ModfCloz:Zip	0	No concerns	Low risk	Some concerns	Major concerns	No concerns	No concerns	Low	[Indirectness, Imprecision]
ModfCloz:ZipCloz	0	No concerns	Low risk	No concerns	Major concerns	No concerns	No concerns	Low	[Imprecision]
ModfCloz:Zot	0	No concerns	Low risk	Some concerns	Major concerns	No concerns	No concerns	Low	[Indirectness, Imprecision]
Ola:OndastHal	0	No concerns	Low risk	No concerns	Major concerns	No concerns	No concerns	Low	[Imprecision]
Ola:OxytRisp	0	Major concerns	Low risk	Some concerns	No concerns	No concerns	No concerns	Low	[Within-study bias, Indirectness]
Ola:Palipla	0	Some concerns	Low risk	No concerns	Major concerns	No concerns	No concerns	Low	[Within-study bias, Imprecision]
Ola:PalmitRisp	0	Major concerns	Low risk	Major concerns	No concerns	Major concerns	No concerns	Very Low	[Within-study bias, Indirectness, Heterogeneity]
Ola:PhenylpropCloz	0	Some concerns	Low risk	Major concerns	Major concerns	No concerns	No concerns	Very Low	[Within-study bias, Indirectness, Imprecision]
Ola:PimozCloz	0	No concerns	Low risk	No concerns	Major concerns	No concerns	No concerns	Low	[Imprecision]

Ola:PtxRisp	0	Major concerns	Low risk	Major concerns	Major concerns	No concerns	No concerns	Very Low	[Within-study bias, Indirectness, Imprecision]
Ola:QueCloz	0	Major concerns	Low risk	No concerns	Major concerns	No concerns	No concerns	Low	[Within-study bias, Imprecision]
Ola:ResverRisp	0	Major concerns	Low risk	Major concerns	Major concerns	No concerns	No concerns	Very Low	[Within-study bias, Indirectness, Imprecision]
Ola:RispCloz	0	No concerns	Low risk	No concerns	No concerns	Major concerns	No concerns	Low	[Heterogeneity]
Ola:SarcCloz	0	No concerns	Low risk	No concerns	Major concerns	No concerns	No concerns	Low	[Imprecision]
Ola:Sertind	0	Some concerns	Low risk	No concerns	Major concerns	No concerns	No concerns	Low	[Within-study bias, Imprecision]
Ola:SertindCloz	0	Some concerns	Low risk	No concerns	Major concerns	No concerns	No concerns	Low	[Within-study bias, Imprecision]
Ola:SerZip	0	No concerns	Low risk	No concerns	Major concerns	No concerns	No concerns	Low	[Imprecision]
Ola:Sul	0	Some concerns	Low risk	No concerns	Some concerns	Some concerns	No concerns	Low	[Within-study bias, Imprecision, Heterogeneity]
Ola:SulCloz	0	Major concerns	Low risk	No concerns	Some concerns	Some concerns	No concerns	Low	[Within-study bias, Imprecision, Heterogeneity]
Ola:TopirCloz	0	Some concerns	Low risk	No concerns	No concerns	Some concerns	No concerns	Moderate	[Within-study bias, Heterogeneity]
Ola:VitD3Cloz	0	No concerns	Low risk	Some concerns	Major concerns	No concerns	No concerns	Low	[Indirectness, Imprecision]
Ola:VortRisp	0	No concerns	Low risk	Major concerns	No concerns	Some concerns	No concerns	Low	[Indirectness, Heterogeneity]
Ola:Zip	0	Some concerns	Low risk	No concerns	Major concerns	No concerns	No concerns	Low	[Within-study bias, Imprecision]
Ola:ZipCloz	0	No concerns	Low risk	No concerns	No concerns	No concerns	No concerns	High	
Ola:Zot	0	Major concerns	Low risk	Some concerns	Major concerns	No concerns	No concerns	Very Low	[Within-study bias, Indirectness, Imprecision]
OlaRisp:OndastHal	0	Some concerns	Low risk	No concerns	Major concerns	No concerns	No concerns	Low	[Within-study bias, Imprecision]

OlaRisp:OxytRisp	0	Major concerns	Low risk	Some concerns	No concerns	No concerns	No concerns	Low	[Within-study bias, Indirectness]
OlaRisp:Palip	0	Some concerns	Low risk	Some concerns	Major concerns	No concerns	No concerns	Low	[Within-study bias, Indirectness, Imprecision]
OlaRisp:Palipla	0	Some concerns	Low risk	Some concerns	Major concerns	No concerns	No concerns	Low	[Within-study bias, Indirectness, Imprecision]
OlaRisp:PalipOla	0	No concerns	Low risk	Some concerns	Major concerns	No concerns	No concerns	Low	[Indirectness, Imprecision]
OlaRisp:PalmitRisp	0	Major concerns	Low risk	Some concerns	Major concerns	No concerns	No concerns	Very Low	[Within-study bias, Indirectness, Imprecision]
OlaRisp:Phenylprop Cloz	0	Some concerns	Low risk	Some concerns	Major concerns	No concerns	No concerns	Low	[Within-study bias, Indirectness, Imprecision]
OlaRisp:PimozCloz	0	Some concerns	Low risk	No concerns	Major concerns	No concerns	No concerns	Low	[Within-study bias, Imprecision]
OlaRisp:PtxRisp	0	Major concerns	Low risk	Some concerns	Major concerns	No concerns	No concerns	Very Low	[Within-study bias, Indirectness, Imprecision]
OlaRisp:Que	0	Some concerns	Low risk	Some concerns	No concerns	Some concerns	No concerns	Low	[Within-study bias, Indirectness, Heterogeneity]
OlaRisp:QueCloz	0	Some concerns	Low risk	No concerns	Some concerns	Some concerns	No concerns	Low	[Within-study bias, Imprecision, Heterogeneity]
OlaRisp:ResverRisp	0	Major concerns	Low risk	Some concerns	Major concerns	No concerns	No concerns	Very Low	[Within-study bias, Indirectness, Imprecision]
OlaRisp:RispCloz	0	Some concerns	Low risk	No concerns	Major concerns	No concerns	No concerns	Low	[Within-study bias, Imprecision]
OlaRisp:SarcCloz	0	Some concerns	Low risk	No concerns	Major concerns	No concerns	No concerns	Low	[Within-study bias, Imprecision]
OlaRisp:SerOla	0	Some concerns	Low risk	Some concerns	Major concerns	No concerns	No concerns	Low	[Within-study bias, Indirectness, Imprecision]
OlaRisp:Sertind	0	Some concerns	Low risk	Some concerns	Some concerns	Some concerns	No concerns	Low	[Within-study bias, Indirectness, Imprecision, Heterogeneity]
OlaRisp:SertindCloz	0	Some concerns	Low risk	No concerns	Major concerns	No concerns	No concerns	Low	[Within-study bias, Imprecision]

OlaRisp:SerZip	0	Some concerns	Low risk	No concerns	Major concerns	No concerns	No concerns	Low	[Within-study bias, Imprecision]
OlaRisp:Sul	0	Some concerns	Low risk	No concerns	No concerns	No concerns	No concerns	Moderate	[Within-study bias]
OlaRisp:SulCloz	0	Some concerns	Low risk	No concerns	Major concerns	No concerns	No concerns	Low	[Within-study bias, Imprecision]
OlaRisp:SulOla	0	Some concerns	Low risk	Some concerns	Major concerns	No concerns	No concerns	Low	[Within-study bias, Indirectness, Imprecision]
OlaRisp:TopirCloz	0	Some concerns	Low risk	No concerns	Major concerns	No concerns	No concerns	Low	[Within-study bias, Imprecision]
OlaRisp:VitD3Cloz	0	Some concerns	Low risk	Some concerns	Major concerns	No concerns	No concerns	Low	[Within-study bias, Indirectness, Imprecision]
OlaRisp:VortRisp	0	Some concerns	Low risk	Some concerns	Major concerns	No concerns	No concerns	Low	[Within-study bias, Indirectness, Imprecision]
OlaRisp:Zip	0	Some concerns	Low risk	Some concerns	Major concerns	No concerns	No concerns	Low	[Within-study bias, Indirectness, Imprecision]
OlaRisp:ZipCloz	0	Some concerns	Low risk	No concerns	Major concerns	No concerns	No concerns	Low	[Within-study bias, Imprecision]
OlaRisp:Zot	0	Some concerns	Low risk	Some concerns	Major concerns	No concerns	No concerns	Low	[Within-study bias, Indirectness, Imprecision]
OndastHal:OxytRisp	0	Major concerns	Low risk	No concerns	No concerns	No concerns	No concerns	Low	[Within-study bias]
OndastHal:Palip	0	Some concerns	Low risk	No concerns	Major concerns	No concerns	No concerns	Low	[Within-study bias, Imprecision]
OndastHal:Palipla	0	Some concerns	Low risk	No concerns	Major concerns	No concerns	No concerns	Low	[Within-study bias, Imprecision]
OndastHal:PalipOla	0	No concerns	Low risk	No concerns	Major concerns	No concerns	No concerns	Low	[Imprecision]
OndastHal:PalmitRisp	0	Major concerns	Low risk	No concerns	Major concerns	No concerns	No concerns	Low	[Within-study bias, Imprecision]
OndastHal:PhenylpropCloz	0	Some concerns	Low risk	No concerns	Major concerns	No concerns	No concerns	Low	[Within-study bias, Imprecision]
OndastHal:PimozCloz	0	No concerns	Low risk	No concerns	Major concerns	No concerns	No concerns	Low	[Imprecision]

OndastHal:PtxRisp	0	Major concerns	Low risk	No concerns	Major concerns	No concerns	No concerns	Low	[Within-study bias, Imprecision]
OndastHal:Que	0	Some concerns	Low risk	No concerns	Some concerns	Some concerns	No concerns	Low	[Within-study bias, Imprecision, Heterogeneity]
OndastHal:QueCloz	0	Some concerns	Low risk	No concerns	Major concerns	No concerns	No concerns	Low	[Within-study bias, Imprecision]
OndastHal:ResverRisp	0	Major concerns	Low risk	No concerns	Major concerns	No concerns	No concerns	Low	[Within-study bias, Imprecision]
OndastHal:Risp	0	No concerns	Low risk	No concerns	Major concerns	No concerns	No concerns	Low	[Imprecision]
OndastHal:RispCloz	0	No concerns	Low risk	No concerns	Major concerns	No concerns	No concerns	Low	[Imprecision]
OndastHal:SarcCloz	0	No concerns	Low risk	No concerns	Major concerns	No concerns	No concerns	Low	[Imprecision]
OndastHal:SerOla	0	Some concerns	Low risk	No concerns	Major concerns	No concerns	No concerns	Low	[Within-study bias, Imprecision]
OndastHal:Sertind	0	Some concerns	Low risk	No concerns	Major concerns	No concerns	No concerns	Low	[Within-study bias, Imprecision]
OndastHal:SertindCloz	0	Some concerns	Low risk	No concerns	Major concerns	No concerns	No concerns	Low	[Within-study bias, Imprecision]
OndastHal:SerZip	0	No concerns	Low risk	No concerns	Major concerns	No concerns	No concerns	Low	[Imprecision]
OndastHal:Sul	0	Some concerns	Low risk	No concerns	No concerns	Some concerns	No concerns	Moderate	[Within-study bias, Heterogeneity]
OndastHal:SulCloz	0	No concerns	Low risk	No concerns	Major concerns	No concerns	No concerns	Low	[Imprecision]
OndastHal:SulOla	0	No concerns	Low risk	No concerns	Major concerns	No concerns	No concerns	Low	[Imprecision]
OndastHal:TopirCloz	0	Some concerns	Low risk	No concerns	Major concerns	No concerns	No concerns	Low	[Within-study bias, Imprecision]
OndastHal:VitD3Cloz	0	No concerns	Low risk	No concerns	Major concerns	No concerns	No concerns	Low	[Imprecision]
OndastHal:VortRisp	0	No concerns	Low risk	No concerns	Major concerns	No concerns	No concerns	Low	[Imprecision]

OndastHal:Zip	0	Some concerns	Low risk	No concerns	Major concerns	No concerns	No concerns	Low	[Within-study bias, Imprecision]
OndastHal:ZipCloz	0	No concerns	Low risk	No concerns	Major concerns	No concerns	No concerns	Low	[Imprecision]
OndastHal:Zot	0	No concerns	Low risk	No concerns	Major concerns	No concerns	No concerns	Low	[Imprecision]
OxytRisp:Palip	0	Major concerns	Low risk	Some concerns	No concerns	No concerns	No concerns	Low	[Within-study bias, Indirectness]
OxytRisp:Palipla	0	Major concerns	Low risk	Some concerns	No concerns	No concerns	No concerns	Low	[Within-study bias, Indirectness]
OxytRisp:PalipOla	0	Major concerns	Low risk	Some concerns	Major concerns	No concerns	No concerns	Very Low	[Within-study bias, Indirectness, Imprecision]
OxytRisp:PalmitRisp	0	Major concerns	Low risk	Some concerns	Major concerns	No concerns	No concerns	Very Low	[Within-study bias, Indirectness, Imprecision]
OxytRisp:PhenylpropCloz	0	Major concerns	Low risk	Some concerns	Major concerns	No concerns	No concerns	Very Low	[Within-study bias, Indirectness, Imprecision]
OxytRisp:PimozCloz	0	Major concerns	Low risk	No concerns	No concerns	No concerns	No concerns	Low	[Within-study bias]
OxytRisp:PtxRisp	0	Major concerns	Low risk	Some concerns	No concerns	No concerns	No concerns	Low	[Within-study bias, Indirectness]
OxytRisp:Que	0	Major concerns	Low risk	Some concerns	No concerns	No concerns	No concerns	Low	[Within-study bias, Indirectness]
OxytRisp:QueCloz	0	Major concerns	Low risk	No concerns	No concerns	No concerns	No concerns	Low	[Within-study bias]
OxytRisp:ResverRisp	0	Major concerns	Low risk	Some concerns	Major concerns	No concerns	No concerns	Very Low	[Within-study bias, Indirectness, Imprecision]
OxytRisp:RispCloz	0	Major concerns	Low risk	No concerns	No concerns	No concerns	No concerns	Low	[Within-study bias]
OxytRisp:SarcCloz	0	Major concerns	Low risk	No concerns	No concerns	No concerns	No concerns	Low	[Within-study bias]
OxytRisp:SerOla	0	Major concerns	Low risk	Some concerns	No concerns	No concerns	No concerns	Low	[Within-study bias, Indirectness]
OxytRisp:Sertind	0	Major concerns	Low risk	No concerns	No concerns	No concerns	No concerns	Low	[Within-study bias]

OxytRisp:SertindCloz	0	Major concerns	Low risk	No concerns	No concerns	No concerns	No concerns	Low	[Within-study bias]
OxytRisp:SerZip	0	Major concerns	Low risk	No concerns	No concerns	No concerns	No concerns	Low	[Within-study bias]
OxytRisp:Sul	0	Major concerns	Low risk	No concerns	No concerns	No concerns	No concerns	Low	[Within-study bias]
OxytRisp:SulCloz	0	Major concerns	Low risk	No concerns	Major concerns	No concerns	No concerns	Low	[Within-study bias, Imprecision]
OxytRisp:SulOla	0	Major concerns	Low risk	Some concerns	Some concerns	Some concerns	No concerns	Very Low	[Within -study bias, Indirectness, Imprecision, Heterogeneity]
OxytRisp:TopirCloz	0	Major concerns	Low risk	No concerns	No concerns	Some concerns	No concerns	Low	[Within-study bias, Heterogeneity]
OxytRisp:VitD3Cloz	0	Major concerns	Low risk	Some concerns	No concerns	No concerns	No concerns	Low	[Within-study bias, Indirectness]
OxytRisp:VortRisp	0	Major concerns	Low risk	Some concerns	Major concerns	No concerns	No concerns	Very Low	[Within-study bias, Indirectness, Imprecision]
OxytRisp:Zip	0	Major concerns	Low risk	Some concerns	No concerns	No concerns	No concerns	Low	[Within-study bias, Indirectness]
OxytRisp:ZipCloz	0	Major concerns	Low risk	No concerns	Major concerns	No concerns	No concerns	Low	[Within-study bias, Imprecision]
OxytRisp:Zot	0	Major concerns	Low risk	Some concerns	No concerns	No concerns	No concerns	Low	[Within-study bias, Indirectness]
Palip:PalipOla	0	No concerns	Low risk	Major concerns	Some concerns	Some concerns	No concerns	Low	[Indirectness, Imprecision, Heterogeneity]
Palip:PalmitRisp	0	Major concerns	Low risk	No concerns	Major concerns	No concerns	No concerns	Low	[Within-study bias, Imprecision]
Palip:PhenylpropCloz	0	Some concerns	Low risk	No concerns	Major concerns	No concerns	No concerns	Low	[Within-study bias, Imprecision]
Palip:PimozCloz	0	Some concerns	Low risk	No concerns	Major concerns	No concerns	No concerns	Low	[Within-study bias, Imprecision]
Palip:PtxRisp	0	Major concerns	Low risk	No concerns	Major concerns	No concerns	No concerns	Low	[Within-study bias, Imprecision]
Palip:Que	0	Some concerns	Low risk	No concerns	Major concerns	No concerns	No concerns	Low	[Within-study bias, Imprecision]

Palip:QueCloz	0	Some concerns	Low risk	No concerns	Major concerns	No concerns	No concerns	Low	[Within-study bias, Imprecision]
Palip:ResverRisp	0	Major concerns	Low risk	No concerns	Major concerns	No concerns	No concerns	Low	[Within-study bias, Imprecision]
Palip:Risp	0	Some concerns	Low risk	No concerns	Major concerns	No concerns	No concerns	Low	[Within-study bias, Imprecision]
Palip:RispCloz	0	Some concerns	Low risk	No concerns	Major concerns	No concerns	No concerns	Low	[Within-study bias, Imprecision]
Palip:SarcCloz	0	Some concerns	Low risk	No concerns	Major concerns	No concerns	No concerns	Low	[Within-study bias, Imprecision]
Palip:SerOla	0	Some concerns	Low risk	No concerns	Major concerns	No concerns	No concerns	Low	[Within-study bias, Imprecision]
Palip:Sertind	0	Some concerns	Low risk	No concerns	Major concerns	No concerns	No concerns	Low	[Within-study bias, Imprecision]
Palip:SertindCloz	0	Some concerns	Low risk	No concerns	Major concerns	No concerns	No concerns	Low	[Within-study bias, Imprecision]
Palip:SerZip	0	Some concerns	Low risk	No concerns	Major concerns	No concerns	No concerns	Low	[Within-study bias, Imprecision]
Palip:Sul	0	Some concerns	Low risk	No concerns	Some concerns	Some concerns	No concerns	Low	[Within-study bias, Imprecision, Heterogeneity]
Palip:SulCloz	0	Some concerns	Low risk	No concerns	Major concerns	No concerns	No concerns	Low	[Within-study bias, Imprecision]
Palip:SulOla	0	Some concerns	Low risk	No concerns	Major concerns	No concerns	No concerns	Low	[Within-study bias, Imprecision]
Palip:TopirCloz	0	Some concerns	Low risk	No concerns	Major concerns	No concerns	No concerns	Low	[Within-study bias, Imprecision]
Palip:VitD3Cloz	0	Some concerns	Low risk	Some concerns	Major concerns	No concerns	No concerns	Low	[Within-study bias, Indirectness, Imprecision]
Palip:VortRisp	0	Some concerns	Low risk	No concerns	Major concerns	No concerns	No concerns	Low	[Within-study bias, Imprecision]
Palip:Zip	0	Some concerns	Low risk	No concerns	Major concerns	No concerns	No concerns	Low	[Within-study bias, Imprecision]
Palip:ZipCloz	0	Some concerns	Low risk	No concerns	Major concerns	No concerns	No concerns	Low	[Within-study bias, Imprecision]

Palip:Zot	0	Some concerns	Low risk	Some concerns	Major concerns	No concerns	No concerns	Low	[Within-study bias, Indirectness, Imprecision]
Palipla:PalipOla	0	No concerns	Low risk	Major concerns	No concerns	Some concerns	No concerns	Low	[Indirectness, Heterogeneity]
Palipla:PalmitRisp	0	Major concerns	Low risk	Some concerns	Major concerns	No concerns	No concerns	Very Low	[Within-study bias, Indirectness, Imprecision]
Palipla:PhenylpropCloz	0	Some concerns	Low risk	Some concerns	Major concerns	No concerns	No concerns	Low	[Within-study bias, Indirectness, Imprecision]
Palipla:PimozCloz	0	Some concerns	Low risk	No concerns	Major concerns	No concerns	No concerns	Low	[Within-study bias, Imprecision]
Palipla:PtxRisp	0	Major concerns	Low risk	Some concerns	Major concerns	No concerns	No concerns	Very Low	[Within-study bias, Indirectness, Imprecision]
Palipla:Que	0	Some concerns	Low risk	No concerns	Major concerns	No concerns	No concerns	Low	[Within-study bias, Imprecision]
Palipla:QueCloz	0	Major concerns	Low risk	No concerns	Major concerns	No concerns	No concerns	Low	[Within-study bias, Imprecision]
Palipla:ResverRisp	0	Major concerns	Low risk	Some concerns	Major concerns	No concerns	No concerns	Very Low	[Within-study bias, Indirectness, Imprecision]
Palipla:Risp	0	Some concerns	Low risk	Some concerns	Major concerns	No concerns	No concerns	Low	[Within-study bias, Indirectness, Imprecision]
Palipla:RispCloz	0	Some concerns	Low risk	No concerns	Major concerns	No concerns	No concerns	Low	[Within-study bias, Imprecision]
Palipla:SarcCloz	0	Some concerns	Low risk	No concerns	Major concerns	No concerns	No concerns	Low	[Within-study bias, Imprecision]
Palipla:SerOla	0	Some concerns	Low risk	No concerns	Major concerns	No concerns	No concerns	Low	[Within-study bias, Imprecision]
Palipla:Sertind	0	Some concerns	Low risk	No concerns	Major concerns	No concerns	No concerns	Low	[Within-study bias, Imprecision]
Palipla:SertindCloz	0	Some concerns	Low risk	No concerns	Major concerns	No concerns	No concerns	Low	[Within-study bias, Imprecision]
Palipla:SerZip	0	Some concerns	Low risk	No concerns	Major concerns	No concerns	No concerns	Low	[Within-study bias, Imprecision]
Palipla:Sul	0	Some concerns	Low risk	No concerns	Major concerns	No concerns	No concerns	Low	[Within-study bias, Imprecision]

Palipla:SulCloz	0	Major concerns	Low risk	No concerns	Major concerns	No concerns	No concerns	Low	[Within-study bias, Imprecision]
Palipla:SulOla	0	Major concerns	Low risk	No concerns	Major concerns	No concerns	No concerns	Low	[Within-study bias, Imprecision]
Palipla:TopirCloz	0	Some concerns	Low risk	No concerns	Major concerns	No concerns	No concerns	Low	[Within-study bias, Imprecision]
Palipla:VitD3Cloz	0	Some concerns	Low risk	Some concerns	Major concerns	No concerns	No concerns	Low	[Within-study bias, Indirectness, Imprecision]
Palipla:VortRisp	0	Some concerns	Low risk	Some concerns	Major concerns	No concerns	No concerns	Low	[Within-study bias, Indirectness, Imprecision]
Palipla:Zip	0	Some concerns	Low risk	No concerns	Major concerns	No concerns	No concerns	Low	[Within-study bias, Imprecision]
Palipla:ZipCloz	0	Some concerns	Low risk	No concerns	Some concerns	Some concerns	No concerns	Low	[Within-study bias, Imprecision, Heterogeneity]
Palipla:Zot	0	Major concerns	Low risk	Some concerns	Major concerns	No concerns	No concerns	Very Low	[Within-study bias, Indirectness, Imprecision]
PalipOla:PalmitRisp	0	Major concerns	Low risk	Major concerns	Major concerns	No concerns	No concerns	Very Low	[Within-study bias, Indirectness, Imprecision]
PalipOla:Phenylprop Cloz	0	Some concerns	Low risk	Major concerns	Major concerns	No concerns	No concerns	Very Low	[Within-study bias, Indirectness, Imprecision]
PalipOla:PimozCloz	0	No concerns	Low risk	No concerns	No concerns	No concerns	No concerns	High	
PalipOla:PtxRisp	0	Major concerns	Low risk	Major concerns	Major concerns	No concerns	No concerns	Very Low	[Within-study bias, Indirectness, Imprecision]
PalipOla:Que	0	No concerns	Low risk	Major concerns	No concerns	No concerns	No concerns	Low	[Indirectness]
PalipOla:QueCloz	0	No concerns	Low risk	No concerns	No concerns	No concerns	No concerns	High	
PalipOla:ResverRisp	0	Major concerns	Low risk	Major concerns	Major concerns	No concerns	No concerns	Very Low	[Within-study bias, Indirectness, Imprecision]
PalipOla:Risp	0	No concerns	Low risk	Major concerns	No concerns	No concerns	No concerns	Low	[Indirectness]
PalipOla:RispCloz	0	No concerns	Low risk	No concerns	Major concerns	No concerns	No concerns	Low	[Imprecision]
PalipOla:SarcCloz	0	No concerns	Low risk	No concerns	Some concerns	Some concerns	No concerns	Moderate	[Imprecision, Heterogeneity]

PalipOla:SerOla	0	No concerns	Low risk	Major concerns	Some concerns	Some concerns	No concerns	Low	[Indirectness, Imprecision, Heterogeneity]
PalipOla:Sertind	0	Some concerns	Low risk	No concerns	No concerns	No concerns	No concerns	Moderate	[Within-study bias]
PalipOla:SertindCloz	0	Some concerns	Low risk	No concerns	Major concerns	No concerns	No concerns	Low	[Within-study bias, Imprecision]
PalipOla:SerZip	0	No concerns	Low risk	No concerns	Major concerns	No concerns	No concerns	Low	[Imprecision]
PalipOla:Sul	0	Some concerns	Low risk	No concerns	No concerns	No concerns	No concerns	Moderate	[Within-study bias]
PalipOla:SulCloz	0	No concerns	Low risk	No concerns	Major concerns	No concerns	No concerns	Low	[Imprecision]
PalipOla:SulOla	0	No concerns	Low risk	Major concerns	Major concerns	No concerns	No concerns	Low	[Indirectness, Imprecision]
PalipOla:TopirCloz	0	No concerns	Low risk	No concerns	Major concerns	No concerns	No concerns	Low	[Imprecision]
PalipOla:VitD3Cloz	0	No concerns	Low risk	Some concerns	Some concerns	Some concerns	No concerns	Low	[Indirectness, Imprecision, Heterogeneity]
PalipOla:VortRisp	0	No concerns	Low risk	Major concerns	Major concerns	No concerns	No concerns	Low	[Indirectness, Imprecision]
PalipOla:Zip	0	No concerns	Low risk	Major concerns	No concerns	No concerns	No concerns	Low	[Indirectness]
PalipOla:ZipCloz	0	No concerns	Low risk	No concerns	Major concerns	No concerns	No concerns	Low	[Imprecision]
PalipOla:Zot	0	No concerns	Low risk	Some concerns	No concerns	No concerns	No concerns	Moderate	[Indirectness]
PalmitRisp:PhenylpropCloz	0	Major concerns	Low risk	Major concerns	Major concerns	No concerns	No concerns	Very Low	[Within-study bias, Indirectness, Imprecision]
PalmitRisp:PimozCloz	0	Major concerns	Low risk	No concerns	Some concerns	Some concerns	No concerns	Low	[Within-study bias, Imprecision, Heterogeneity]
PalmitRisp:PtxRisp	0	Major concerns	Low risk	Major concerns	Major concerns	No concerns	No concerns	Very Low	[Within-study bias, Indirectness, Imprecision]
PalmitRisp:Que	0	Major concerns	Low risk	No concerns	No concerns	No concerns	No concerns	Low	[Within-study bias]

PalmitRisp:QueCloz	0	Major concerns	Low risk	No concerns	No concerns	No concerns	No concerns	Low	[Within-study bias]
PalmitRisp:ResverRisp	0	Major concerns	Low risk	Major concerns	Major concerns	No concerns	No concerns	Very Low	[Within-study bias, Indirectness, Imprecision]
PalmitRisp:RispCloz	0	Major concerns	Low risk	No concerns	Major concerns	No concerns	No concerns	Low	[Within-study bias, Imprecision]
PalmitRisp:SarcCloz	0	Major concerns	Low risk	No concerns	Major concerns	No concerns	No concerns	Low	[Within-study bias, Imprecision]
PalmitRisp:SerOla	0	Major concerns	Low risk	No concerns	Major concerns	No concerns	No concerns	Low	[Within-study bias, Imprecision]
PalmitRisp:Sertind	0	Major concerns	Low risk	No concerns	No concerns	No concerns	No concerns	Low	[Within-study bias]
PalmitRisp:SertindCloz	0	Major concerns	Low risk	No concerns	Major concerns	No concerns	No concerns	Low	[Within-study bias, Imprecision]
PalmitRisp:SerZip	0	Major concerns	Low risk	No concerns	Major concerns	No concerns	No concerns	Low	[Within-study bias, Imprecision]
PalmitRisp:Sul	0	Major concerns	Low risk	No concerns	No concerns	No concerns	No concerns	Low	[Within-study bias]
PalmitRisp:SulCloz	0	Major concerns	Low risk	No concerns	Major concerns	No concerns	No concerns	Low	[Within-study bias, Imprecision]
PalmitRisp:SulOla	0	Major concerns	Low risk	No concerns	Major concerns	No concerns	No concerns	Low	[Within-study bias, Imprecision]
PalmitRisp:TopirCloz	0	Major concerns	Low risk	No concerns	Major concerns	No concerns	No concerns	Low	[Within-study bias, Imprecision]
PalmitRisp:VitD3Cloz	0	Major concerns	Low risk	Some concerns	Major concerns	No concerns	No concerns	Very Low	[Within-study bias, Indirectness, Imprecision]
PalmitRisp:VortRisp	0	Major concerns	Low risk	Major concerns	Major concerns	No concerns	No concerns	Very Low	[Within-study bias, Indirectness, Imprecision]
PalmitRisp:Zip	0	Major concerns	Low risk	No concerns	No concerns	Some concerns	No concerns	Low	[Within-study bias, Heterogeneity]
PalmitRisp:ZipCloz	0	Major concerns	Low risk	No concerns	Major concerns	No concerns	No concerns	Low	[Within-study bias, Imprecision]
PalmitRisp:Zot	0	Major concerns	Low risk	Some concerns	Some concerns	Some concerns	No concerns	Very Low	[Within-study bias, Indirectness, Imprecision, Heterogeneity]

PhenylpropCloz:PimozCloz	0	No concerns	Low risk	No concerns	Major concerns	No concerns	No concerns	Low	[Imprecision]
PhenylpropCloz:PtxRisp	0	Major concerns	Low risk	Major concerns	Major concerns	No concerns	No concerns	Very Low	[Within-study bias, Indirectness, Imprecision]
PhenylpropCloz:Que	0	Some concerns	Low risk	No concerns	Some concerns	Some concerns	No concerns	Low	[Within-study bias, Imprecision, Heterogeneity]
PhenylpropCloz:QueCloz	0	Some concerns	Low risk	No concerns	Major concerns	No concerns	No concerns	Low	[Within-study bias, Imprecision]
PhenylpropCloz:ResverRisp	0	Major concerns	Low risk	Major concerns	Major concerns	No concerns	No concerns	Very Low	[Within-study bias, Indirectness, Imprecision]
PhenylpropCloz:Risp	0	Some concerns	Low risk	Major concerns	Major concerns	No concerns	No concerns	Very Low	[Within-study bias, Indirectness, Imprecision]
PhenylpropCloz:RispCloz	0	Some concerns	Low risk	No concerns	Major concerns	No concerns	No concerns	Low	[Within-study bias, Imprecision]
PhenylpropCloz:SarcCloz	0	No concerns	Low risk	No concerns	Major concerns	No concerns	No concerns	Low	[Imprecision]
PhenylpropCloz:SerOla	0	Some concerns	Low risk	No concerns	Major concerns	No concerns	No concerns	Low	[Within-study bias, Imprecision]
PhenylpropCloz:Sertind	0	Some concerns	Low risk	No concerns	Major concerns	No concerns	No concerns	Low	[Within-study bias, Imprecision]
PhenylpropCloz:SertindCloz	0	Some concerns	Low risk	No concerns	Major concerns	No concerns	No concerns	Low	[Within-study bias, Imprecision]
PhenylpropCloz:SerZip	0	Some concerns	Low risk	No concerns	Major concerns	No concerns	No concerns	Low	[Within-study bias, Imprecision]
PhenylpropCloz:Sul	0	Some concerns	Low risk	No concerns	No concerns	No concerns	No concerns	Moderate	[Within-study bias]
PhenylpropCloz:SulCloz	0	Some concerns	Low risk	No concerns	Major concerns	No concerns	No concerns	Low	[Within-study bias, Imprecision]
PhenylpropCloz:SulOla	0	Some concerns	Low risk	No concerns	Major concerns	No concerns	No concerns	Low	[Within-study bias, Imprecision]
PhenylpropCloz:TopirCloz	0	Some concerns	Low risk	No concerns	Major concerns	No concerns	No concerns	Low	[Within-study bias, Imprecision]
PhenylpropCloz:VitD3Cloz	0	No concerns	Low risk	Some concerns	Major concerns	No concerns	No concerns	Low	[Indirectness, Imprecision]

PhenylpropCloz:VortRisp	0	Some concerns	Low risk	Major concerns	Major concerns	No concerns	No concerns	Very Low	[Within-study bias, Indirectness, Imprecision]
PhenylpropCloz:Zip	0	Some concerns	Low risk	Major concerns	Major concerns	No concerns	No concerns	Very Low	[Within-study bias, Indirectness, Imprecision]
PhenylpropCloz:ZipCloz	0	Some concerns	Low risk	No concerns	Major concerns	No concerns	No concerns	Low	[Within-study bias, Imprecision]
PhenylpropCloz:Zot	0	Some concerns	Low risk	Some concerns	Major concerns	No concerns	No concerns	Low	[Within-study bias, Indirectness, Imprecision]
PimozCloz:PtxRisp	0	Major concerns	Low risk	No concerns	Major concerns	No concerns	No concerns	Low	[Within-study bias, Imprecision]
PimozCloz:Que	0	No concerns	Low risk	No concerns	Major concerns	No concerns	No concerns	Low	[Imprecision]
PimozCloz:QueCloz	0	Major concerns	Low risk	No concerns	Major concerns	No concerns	No concerns	Low	[Within-study bias, Imprecision]
PimozCloz:ResverRisp	0	Major concerns	Low risk	No concerns	Major concerns	No concerns	No concerns	Low	[Within-study bias, Imprecision]
PimozCloz:Risp	0	No concerns	Low risk	No concerns	Major concerns	No concerns	No concerns	Low	[Imprecision]
PimozCloz:RispCloz	0	No concerns	Low risk	No concerns	Major concerns	No concerns	No concerns	Low	[Imprecision]
PimozCloz:SarcCloz	0	No concerns	Low risk	No concerns	Major concerns	No concerns	No concerns	Low	[Imprecision]
PimozCloz:SerOla	0	Some concerns	Low risk	No concerns	Major concerns	No concerns	No concerns	Low	[Within-study bias, Imprecision]
PimozCloz:Sertind	0	Some concerns	Low risk	No concerns	Major concerns	No concerns	No concerns	Low	[Within-study bias, Imprecision]
PimozCloz:SertindCloz	0	No concerns	Low risk	No concerns	Major concerns	No concerns	No concerns	Low	[Imprecision]
PimozCloz:SerZip	0	No concerns	Low risk	No concerns	Major concerns	No concerns	No concerns	Low	[Imprecision]
PimozCloz:Sul	0	No concerns	Low risk	No concerns	Major concerns	No concerns	No concerns	Low	[Imprecision]
PimozCloz:SulCloz	0	No concerns	Low risk	No concerns	Some concerns	Some concerns	No concerns	Moderate	[Imprecision, Heterogeneity]

PimozCloz:SulOla	0	No concerns	Low risk	No concerns	Major concerns	No concerns	No concerns	Low	[Imprecision]
PimozCloz:TopirCloz	0	No concerns	Low risk	No concerns	No concerns	Major concerns	No concerns	Low	[Heterogeneity]
PimozCloz:VitD3Cloz	0	No concerns	Low risk	No concerns	Major concerns	No concerns	No concerns	Low	[Imprecision]
PimozCloz:VortRisp	0	No concerns	Low risk	No concerns	No concerns	Some concerns	No concerns	Moderate	[Heterogeneity]
PimozCloz:Zip	0	No concerns	Low risk	No concerns	Major concerns	No concerns	No concerns	Low	[Imprecision]
PimozCloz:ZipCloz	0	No concerns	Low risk	No concerns	No concerns	No concerns	No concerns	High	
PimozCloz:Zot	0	No concerns	Low risk	No concerns	Major concerns	No concerns	No concerns	Low	[Imprecision]
PtxRisp:Que	0	Major concerns	Low risk	No concerns	Some concerns	Some concerns	No concerns	Low	[Within-study bias, Imprecision, Heterogeneity]
PtxRisp:QueCloz	0	Major concerns	Low risk	No concerns	Major concerns	No concerns	No concerns	Low	[Within-study bias, Imprecision]
PtxRisp:ResverRisp	0	Major concerns	Low risk	Major concerns	Major concerns	No concerns	No concerns	Very Low	[Within-study bias, Indirectness, Imprecision]
PtxRisp:RispCloz	0	Major concerns	Low risk	No concerns	Major concerns	No concerns	No concerns	Low	[Within-study bias, Imprecision]
PtxRisp:SarcCloz	0	Major concerns	Low risk	No concerns	Major concerns	No concerns	No concerns	Low	[Within-study bias, Imprecision]
PtxRisp:SerOla	0	Major concerns	Low risk	No concerns	Major concerns	No concerns	No concerns	Low	[Within-study bias, Imprecision]
PtxRisp:Sertind	0	Major concerns	Low risk	No concerns	Major concerns	No concerns	No concerns	Low	[Within-study bias, Imprecision]
PtxRisp:SertindCloz	0	Major concerns	Low risk	No concerns	Major concerns	No concerns	No concerns	Low	[Within-study bias, Imprecision]
PtxRisp:SerZip	0	Major concerns	Low risk	No concerns	Major concerns	No concerns	No concerns	Low	[Within-study bias, Imprecision]
PtxRisp:Sul	0	Major concerns	Low risk	No concerns	No concerns	Some concerns	No concerns	Low	[Within-study bias, Heterogeneity]
PtxRisp:SulCloz	0	Major concerns	Low risk	No concerns	Major concerns	No concerns	No concerns	Low	[Within-study bias, Imprecision]

PtxRisp:SulOla	0	Major concerns	Low risk	No concerns	Major concerns	No concerns	No concerns	Low	[Within-study bias, Imprecision]
PtxRisp:TopirCloz	0	Major concerns	Low risk	No concerns	Major concerns	No concerns	No concerns	Low	[Within-study bias, Imprecision]
PtxRisp:VitD3Cloz	0	Major concerns	Low risk	Some concerns	Major concerns	No concerns	No concerns	Very Low	[Within-study bias, Indirectness, Imprecision]
PtxRisp:VortRisp	0	Major concerns	Low risk	Major concerns	Major concerns	No concerns	No concerns	Very Low	[Within-study bias, Indirectness, Imprecision]
PtxRisp:Zip	0	Major concerns	Low risk	No concerns	Major concerns	No concerns	No concerns	Low	[Within-study bias, Imprecision]
PtxRisp:ZipCloz	0	Major concerns	Low risk	No concerns	Major concerns	No concerns	No concerns	Low	[Within-study bias, Imprecision]
PtxRisp:Zot	0	Major concerns	Low risk	Some concerns	Major concerns	No concerns	No concerns	Very Low	[Within-study bias, Indirectness, Imprecision]
Que:QueCloz	0	Some concerns	Low risk	No concerns	Major concerns	No concerns	No concerns	Low	[Within-study bias, Imprecision]
Que:ResverRisp	0	Major concerns	Low risk	No concerns	No concerns	No concerns	No concerns	Low	[Within-study bias]
Que:RispCloz	0	Some concerns	Low risk	No concerns	No concerns	No concerns	No concerns	Moderate	[Within-study bias]
Que:SarcCloz	0	No concerns	Low risk	No concerns	Major concerns	No concerns	No concerns	Low	[Imprecision]
Que:SerOla	0	Some concerns	Low risk	No concerns	Major concerns	No concerns	No concerns	Low	[Within-study bias, Imprecision]
Que:Sertind	0	Some concerns	Low risk	No concerns	Major concerns	No concerns	No concerns	Low	[Within-study bias, Imprecision]
Que:SertindCloz	0	Some concerns	Low risk	No concerns	Major concerns	No concerns	No concerns	Low	[Within-study bias, Imprecision]
Que:SerZip	0	No concerns	Low risk	No concerns	Major concerns	No concerns	No concerns	Low	[Imprecision]
Que:Sul	0	Some concerns	Low risk	No concerns	Major concerns	No concerns	No concerns	Low	[Within-study bias, Imprecision]
Que:SulCloz	0	Some concerns	Low risk	No concerns	No concerns	No concerns	No concerns	Moderate	[Within-study bias]

Que:SulOla	0	Some concerns	Low risk	No concerns	Major concerns	No concerns	No concerns	Low	[Within-study bias, Imprecision]
Que:TopirCloz	0	Some concerns	Low risk	No concerns	No concerns	No concerns	No concerns	Moderate	[Within-study bias]
Que:VitD3Cloz	0	No concerns	Low risk	Some concerns	Major concerns	No concerns	No concerns	Low	[Indirectness, Imprecision]
Que:VortRisp	0	No concerns	Low risk	No concerns	No concerns	No concerns	No concerns	High	
Que:Zip	0	Some concerns	Low risk	No concerns	Major concerns	No concerns	No concerns	Low	[Within-study bias, Imprecision]
Que:ZipCloz	0	No concerns	Low risk	No concerns	No concerns	No concerns	No concerns	High	
Que:Zot	0	Some concerns	Low risk	Some concerns	Major concerns	No concerns	No concerns	Low	[Within-study bias, Indirectness, Imprecision]
QueCloz:ResverRisp	0	Major concerns	Low risk	No concerns	No concerns	No concerns	No concerns	Low	[Within-study bias]
QueCloz:Risp	0	Major concerns	Low risk	No concerns	Major concerns	No concerns	No concerns	Low	[Within-study bias, Imprecision]
QueCloz:RispCloz	0	Major concerns	Low risk	No concerns	No concerns	Some concerns	No concerns	Low	[Within-study bias, Heterogeneity]
QueCloz:SarcCloz	0	Major concerns	Low risk	No concerns	Major concerns	No concerns	No concerns	Low	[Within-study bias, Imprecision]
QueCloz:SerOla	0	Some concerns	Low risk	No concerns	Major concerns	No concerns	No concerns	Low	[Within-study bias, Imprecision]
QueCloz:Sertind	0	Some concerns	Low risk	No concerns	Major concerns	No concerns	No concerns	Low	[Within-study bias, Imprecision]
QueCloz:SertindClo z	0	Some concerns	Low risk	No concerns	Major concerns	No concerns	No concerns	Low	[Within-study bias, Imprecision]
QueCloz:SerZip	0	Some concerns	Low risk	No concerns	Major concerns	No concerns	No concerns	Low	[Within-study bias, Imprecision]
QueCloz:Sul	0	Some concerns	Low risk	No concerns	Major concerns	No concerns	No concerns	Low	[Within-study bias, Imprecision]
QueCloz:SulCloz	0	Major concerns	Low risk	No concerns	No concerns	No concerns	No concerns	Low	[Within-study bias]
QueCloz:SulOla	0	Major concerns	Low risk	No concerns	Major concerns	No concerns	No concerns	Low	[Within-study bias, Imprecision]

QueCloz:TopirCloz	0	Major concerns	Low risk	No concerns	No concerns	No concerns	No concerns	Low	[Within-study bias]
QueCloz:VitD3Cloz	0	Major concerns	Low risk	No concerns	Major concerns	No concerns	No concerns	Low	[Within-study bias, Imprecision]
QueCloz:VortRisp	0	Major concerns	Low risk	No concerns	No concerns	No concerns	No concerns	Low	[Within-study bias]
QueCloz:Zip	0	Some concerns	Low risk	No concerns	Major concerns	No concerns	No concerns	Low	[Within-study bias, Imprecision]
QueCloz:ZipCloz	0	Major concerns	Low risk	No concerns	No concerns	No concerns	No concerns	Low	[Within-study bias]
QueCloz:Zot	0	Major concerns	Low risk	No concerns	Major concerns	No concerns	No concerns	Low	[Within-study bias, Imprecision]
ResverRisp:RispCloz	0	Major concerns	Low risk	No concerns	Major concerns	No concerns	No concerns	Low	[Within-study bias, Imprecision]
ResverRisp:SarcCloz	0	Major concerns	Low risk	No concerns	Major concerns	No concerns	No concerns	Low	[Within-study bias, Imprecision]
ResverRisp:SerOla	0	Major concerns	Low risk	No concerns	Major concerns	No concerns	No concerns	Low	[Within-study bias, Imprecision]
ResverRisp:Sertind	0	Major concerns	Low risk	No concerns	No concerns	No concerns	No concerns	Low	[Within-study bias]
ResverRisp:SertindCloz	0	Major concerns	Low risk	No concerns	Major concerns	No concerns	No concerns	Low	[Within-study bias, Imprecision]
ResverRisp:SerZip	0	Major concerns	Low risk	No concerns	Major concerns	No concerns	No concerns	Low	[Within-study bias, Imprecision]
ResverRisp:Sul	0	Major concerns	Low risk	No concerns	No concerns	No concerns	No concerns	Low	[Within-study bias]
ResverRisp:SulCloz	0	Major concerns	Low risk	No concerns	Major concerns	No concerns	No concerns	Low	[Within-study bias, Imprecision]
ResverRisp:SulOla	0	Major concerns	Low risk	No concerns	Major concerns	No concerns	No concerns	Low	[Within-study bias, Imprecision]
ResverRisp:TopirCloz	0	Major concerns	Low risk	No concerns	Major concerns	No concerns	No concerns	Low	[Within-study bias, Imprecision]
ResverRisp:VitD3Cloz	0	Major concerns	Low risk	Some concerns	Major concerns	No concerns	No concerns	Very Low	[Within-study bias, Indirectness, Imprecision]

ResverRisp:VortRisp	0	Major concerns	Low risk	Major concerns	Major concerns	No concerns	No concerns	Very Low	[Within-study bias, Indirectness, Imprecision]
ResverRisp:Zip	0	Major concerns	Low risk	No concerns	No concerns	Major concerns	No concerns	Low	[Within-study bias, Heterogeneity]
ResverRisp:ZipCloz	0	Major concerns	Low risk	No concerns	Major concerns	No concerns	No concerns	Low	[Within-study bias, Imprecision]
ResverRisp:Zot	0	Major concerns	Low risk	Some concerns	Major concerns	No concerns	No concerns	Very Low	[Within-study bias, Indirectness, Imprecision]
Risp:RispCloz	0	Some concerns	Low risk	No concerns	No concerns	Major concerns	No concerns	Low	[Within-study bias, Heterogeneity]
Risp:SarcCloz	0	No concerns	Low risk	No concerns	Major concerns	No concerns	No concerns	Low	[Imprecision]
Risp:SerOla	0	Some concerns	Low risk	No concerns	Major concerns	No concerns	No concerns	Low	[Within-study bias, Imprecision]
Risp:SertindCloz	0	Some concerns	Low risk	No concerns	Major concerns	No concerns	No concerns	Low	[Within-study bias, Imprecision]
Risp:SerZip	0	Some concerns	Low risk	No concerns	Major concerns	No concerns	No concerns	Low	[Within-study bias, Imprecision]
Risp:Sul	0	Some concerns	Low risk	No concerns	Major concerns	No concerns	No concerns	Low	[Within-study bias, Imprecision]
Risp:SulCloz	0	Major concerns	Low risk	No concerns	No concerns	Some concerns	No concerns	Low	[Within-study bias, Heterogeneity]
Risp:SulOla	0	Major concerns	Low risk	No concerns	Major concerns	No concerns	No concerns	Low	[Within-study bias, Imprecision]
Risp:TopirCloz	0	Some concerns	Low risk	No concerns	No concerns	No concerns	No concerns	Moderate	[Within-study bias]
Risp:VitD3Cloz	0	No concerns	Low risk	Some concerns	Major concerns	No concerns	No concerns	Low	[Indirectness, Imprecision]
Risp:Zip	0	Some concerns	Low risk	No concerns	Major concerns	No concerns	No concerns	Low	[Within-study bias, Imprecision]
Risp:ZipCloz	0	No concerns	Low risk	No concerns	No concerns	No concerns	No concerns	High	
Risp:Zot	0	Major concerns	Low risk	Some concerns	Major concerns	No concerns	No concerns	Very Low	[Within-study bias, Indirectness, Imprecision]
RispCloz:SarcCloz	0	No concerns	Low risk	No concerns	Major concerns	No concerns	No concerns	Low	[Imprecision]

RispCloz:SerOla	0	Some concerns	Low risk	No concerns	Major concerns	No concerns	No concerns	Low	[Within-study bias, Imprecision]
RispCloz:Sertind	0	Some concerns	Low risk	No concerns	No concerns	Some concerns	No concerns	Moderate	[Within-study bias, Heterogeneity]
RispCloz:SertindCloz	0	Some concerns	Low risk	No concerns	Major concerns	No concerns	No concerns	Low	[Within-study bias, Imprecision]
RispCloz:SerZip	0	No concerns	Low risk	No concerns	Major concerns	No concerns	No concerns	Low	[Imprecision]
RispCloz:Sul	0	Some concerns	Low risk	No concerns	No concerns	No concerns	No concerns	Moderate	[Within-study bias]
RispCloz:SulCloz	0	Major concerns	Low risk	No concerns	Major concerns	No concerns	No concerns	Low	[Within-study bias, Imprecision]
RispCloz:SulOla	0	Major concerns	Low risk	No concerns	Major concerns	No concerns	No concerns	Low	[Within-study bias, Imprecision]
RispCloz:TopirCloz	0	No concerns	Low risk	No concerns	Major concerns	No concerns	No concerns	Low	[Imprecision]
RispCloz:VitD3Cloz	0	No concerns	Low risk	No concerns	Major concerns	No concerns	No concerns	Low	[Imprecision]
RispCloz:VortRisp	0	No concerns	Low risk	No concerns	Major concerns	No concerns	No concerns	Low	[Imprecision]
RispCloz:Zip	0	Some concerns	Low risk	No concerns	No concerns	Major concerns	No concerns	Low	[Within-study bias, Heterogeneity]
RispCloz:Zot	0	Major concerns	Low risk	No concerns	Major concerns	No concerns	No concerns	Low	[Within-study bias, Imprecision]
SarcCloz:SerOla	0	Some concerns	Low risk	No concerns	Major concerns	No concerns	No concerns	Low	[Within-study bias, Imprecision]
SarcCloz:Sertind	0	Some concerns	Low risk	No concerns	Major concerns	No concerns	No concerns	Low	[Within-study bias, Imprecision]
SarcCloz:SertindCloz	0	No concerns	Low risk	No concerns	Major concerns	No concerns	No concerns	Low	[Imprecision]
SarcCloz:SerZip	0	No concerns	Low risk	No concerns	Major concerns	No concerns	No concerns	Low	[Imprecision]
SarcCloz:Sul	0	No concerns	Low risk	No concerns	Major concerns	No concerns	No concerns	Low	[Imprecision]

SarcCloz:SulCloz	0	No concerns	Low risk	No concerns	Major concerns	No concerns	No concerns	Low	[Imprecision]
SarcCloz:SulOla	0	No concerns	Low risk	No concerns	Major concerns	No concerns	No concerns	Low	[Imprecision]
SarcCloz:TopirCloz	0	No concerns	Low risk	No concerns	Major concerns	No concerns	No concerns	Low	[Imprecision]
SarcCloz:VitD3Cloz	0	No concerns	Low risk	No concerns	Major concerns	No concerns	No concerns	Low	[Imprecision]
SarcCloz:VortRisp	0	No concerns	Low risk	No concerns	Major concerns	No concerns	No concerns	Low	[Imprecision]
SarcCloz:Zip	0	No concerns	Low risk	No concerns	Major concerns	No concerns	No concerns	Low	[Imprecision]
SarcCloz:ZipCloz	0	No concerns	Low risk	No concerns	Major concerns	No concerns	No concerns	Low	[Imprecision]
SarcCloz:Zot	0	No concerns	Low risk	No concerns	Major concerns	No concerns	No concerns	Low	[Imprecision]
SerOla:Sertind	0	Some concerns	Low risk	No concerns	Major concerns	No concerns	No concerns	Low	[Within-study bias, Imprecision]
SerOla:SertindCloz	0	Some concerns	Low risk	No concerns	Major concerns	No concerns	No concerns	Low	[Within-study bias, Imprecision]
SerOla:SerZip	0	Some concerns	Low risk	No concerns	Major concerns	No concerns	No concerns	Low	[Within-study bias, Imprecision]
SerOla:Sul	0	Some concerns	Low risk	No concerns	Major concerns	No concerns	No concerns	Low	[Within-study bias, Imprecision]
SerOla:SulCloz	0	Some concerns	Low risk	No concerns	Major concerns	No concerns	No concerns	Low	[Within-study bias, Imprecision]
SerOla:SulOla	0	Some concerns	Low risk	No concerns	Major concerns	No concerns	No concerns	Low	[Within-study bias, Imprecision]
SerOla:TopirCloz	0	Some concerns	Low risk	No concerns	Major concerns	No concerns	No concerns	Low	[Within-study bias, Imprecision]
SerOla:VitD3Cloz	0	Some concerns	Low risk	Some concerns	Major concerns	No concerns	No concerns	Low	[Within-study bias, Indirectness, Imprecision]
SerOla:VortRisp	0	Some concerns	Low risk	No concerns	Major concerns	No concerns	No concerns	Low	[Within-study bias, Imprecision]

SerOla:Zip	0	Some concerns	Low risk	No concerns	Major concerns	No concerns	No concerns	Low	[Within-study bias, Imprecision]
SerOla:ZipCloz	0	Some concerns	Low risk	No concerns	Major concerns	No concerns	No concerns	Low	[Within-study bias, Imprecision]
SerOla:Zot	0	Some concerns	Low risk	Some concerns	Major concerns	No concerns	No concerns	Low	[Within-study bias, Indirectness, Imprecision]
Sertind:SertindCloz	0	Some concerns	Low risk	No concerns	Major concerns	No concerns	No concerns	Low	[Within-study bias, Imprecision]
Sertind:SerZip	0	Some concerns	Low risk	No concerns	Major concerns	No concerns	No concerns	Low	[Within-study bias, Imprecision]
Sertind:Sul	0	Some concerns	Low risk	No concerns	Major concerns	No concerns	No concerns	Low	[Within-study bias, Imprecision]
Sertind:SulCloz	0	Some concerns	Low risk	No concerns	No concerns	No concerns	No concerns	Moderate	[Within-study bias]
Sertind:SulOla	0	Some concerns	Low risk	No concerns	Major concerns	No concerns	No concerns	Low	[Within-study bias, Imprecision]
Sertind:TopirCloz	0	Some concerns	Low risk	No concerns	No concerns	No concerns	No concerns	Moderate	[Within-study bias]
Sertind:VitD3Cloz	0	Some concerns	Low risk	No concerns	Major concerns	No concerns	No concerns	Low	[Within-study bias, Imprecision]
Sertind:VortRisp	0	No concerns	Low risk	No concerns	No concerns	No concerns	No concerns	High	
Sertind:Zip	0	Some concerns	Low risk	No concerns	Major concerns	No concerns	No concerns	Low	[Within-study bias, Imprecision]
Sertind:ZipCloz	0	Some concerns	Low risk	No concerns	No concerns	No concerns	No concerns	Moderate	[Within-study bias]
Sertind:Zot	0	Some concerns	Low risk	No concerns	Major concerns	No concerns	No concerns	Low	[Within-study bias, Imprecision]
SertindCloz:SerZip	0	Some concerns	Low risk	No concerns	Major concerns	No concerns	No concerns	Low	[Within-study bias, Imprecision]
SertindCloz:Sul	0	Some concerns	Low risk	No concerns	Some concerns	Some concerns	No concerns	Low	[Within-study bias, Imprecision, Heterogeneity]
SertindCloz:SulCloz	0	Some concerns	Low risk	No concerns	Major concerns	No concerns	No concerns	Low	[Within-study bias, Imprecision]

SertindCloz:SulOla	0	Some concerns	Low risk	No concerns	Major concerns	No concerns	No concerns	Low	[Within-study bias, Imprecision]
SertindCloz:TopirCloz	0	Some concerns	Low risk	No concerns	Major concerns	No concerns	No concerns	Low	[Within-study bias, Imprecision]
SertindCloz:VitD3Cloz	0	No concerns	Low risk	No concerns	Major concerns	No concerns	No concerns	Low	[Imprecision]
SertindCloz:VortRisp	0	Some concerns	Low risk	No concerns	Major concerns	No concerns	No concerns	Low	[Within-study bias, Imprecision]
SertindCloz:Zip	0	Some concerns	Low risk	No concerns	Major concerns	No concerns	No concerns	Low	[Within-study bias, Imprecision]
SertindCloz:ZipCloz	0	Some concerns	Low risk	No concerns	Major concerns	No concerns	No concerns	Low	[Within-study bias, Imprecision]
SertindCloz:Zot	0	Some concerns	Low risk	No concerns	Major concerns	No concerns	No concerns	Low	[Within-study bias, Imprecision]
SerZip:Sul	0	Some concerns	Low risk	No concerns	Some concerns	Some concerns	No concerns	Low	[Within-study bias, Imprecision, Heterogeneity]
SerZip:SulCloz	0	No concerns	Low risk	No concerns	Major concerns	No concerns	No concerns	Low	[Imprecision]
SerZip:SulOla	0	No concerns	Low risk	No concerns	Major concerns	No concerns	No concerns	Low	[Imprecision]
SerZip:TopirCloz	0	No concerns	Low risk	No concerns	Major concerns	No concerns	No concerns	Low	[Imprecision]
SerZip:VitD3Cloz	0	No concerns	Low risk	No concerns	Major concerns	No concerns	No concerns	Low	[Imprecision]
SerZip:VortRisp	0	No concerns	Low risk	No concerns	Major concerns	No concerns	No concerns	Low	[Imprecision]
SerZip:ZipCloz	0	No concerns	Low risk	No concerns	Major concerns	No concerns	No concerns	Low	[Imprecision]
SerZip:Zot	0	No concerns	Low risk	No concerns	Major concerns	No concerns	No concerns	Low	[Imprecision]
Sul:SulCloz	0	Some concerns	Low risk	No concerns	No concerns	No concerns	No concerns	Moderate	[Within-study bias]
Sul:SulOla	0	Some concerns	Low risk	No concerns	Major concerns	No concerns	No concerns	Low	[Within-study bias, Imprecision]

Sul:TopirCloz	0	Some concerns	Low risk	No concerns	No concerns	No concerns	No concerns	Moderate	[Within-study bias]
Sul:VitD3Cloz	0	No concerns	Low risk	No concerns	Major concerns	No concerns	No concerns	Low	[Imprecision]
Sul:VortRisp	0	Some concerns	Low risk	No concerns	No concerns	No concerns	No concerns	Moderate	[Within-study bias]
Sul:Zip	0	Some concerns	Low risk	No concerns	Major concerns	No concerns	No concerns	Low	[Within-study bias, Imprecision]
Sul:ZipCloz	0	Some concerns	Low risk	No concerns	No concerns	No concerns	No concerns	Moderate	[Within-study bias]
Sul:Zot	0	Some concerns	Low risk	No concerns	Major concerns	No concerns	No concerns	Low	[Within-study bias, Imprecision]
SulCloz:SulOla	0	Major concerns	Low risk	No concerns	Major concerns	No concerns	No concerns	Low	[Within-study bias, Imprecision]
SulCloz:TopirCloz	0	Major concerns	Low risk	No concerns	Major concerns	No concerns	No concerns	Low	[Within-study bias, Imprecision]
SulCloz:VitD3Cloz	0	No concerns	Low risk	No concerns	Major concerns	No concerns	No concerns	Low	[Imprecision]
SulCloz:VortRisp	0	No concerns	Low risk	No concerns	Major concerns	No concerns	No concerns	Low	[Imprecision]
SulCloz:Zip	0	Some concerns	Low risk	No concerns	No concerns	Some concerns	No concerns	Moderate	[Within-study bias, Heterogeneity]
SulCloz:ZipCloz	0	Major concerns	Low risk	No concerns	Major concerns	No concerns	No concerns	Low	[Within-study bias, Imprecision]
SulCloz:Zot	0	Major concerns	Low risk	No concerns	Some concerns	Some concerns	No concerns	Low	[Within-study bias, Imprecision, Heterogeneity]
SulOla:TopirCloz	0	Major concerns	Low risk	No concerns	Major concerns	No concerns	No concerns	Low	[Within-study bias, Imprecision]
SulOla:VitD3Cloz	0	No concerns	Low risk	Some concerns	Major concerns	No concerns	No concerns	Low	[Indirectness, Imprecision]
SulOla:VortRisp	0	No concerns	Low risk	No concerns	Major concerns	No concerns	No concerns	Low	[Imprecision]
SulOla:Zip	0	Some concerns	Low risk	No concerns	Major concerns	No concerns	No concerns	Low	[Within-study bias, Imprecision]

SulOla:ZipCloz	0	Major concerns	Low risk	No concerns	Major concerns	No concerns	No concerns	Low	[Within-study bias, Imprecision]
SulOla:Zot	0	Major concerns	Low risk	Some concerns	Major concerns	No concerns	No concerns	Very Low	[Within-study bias, Indirectness, Imprecision]
TopirCloz:VitD3Cloz	0	No concerns	Low risk	No concerns	Major concerns	No concerns	No concerns	Low	[Imprecision]
TopirCloz:VortRisp	0	No concerns	Low risk	No concerns	Major concerns	No concerns	No concerns	Low	[Imprecision]
TopirCloz:Zip	0	Some concerns	Low risk	No concerns	No concerns	No concerns	No concerns	Moderate	[Within-study bias]
TopirCloz:ZipCloz	0	No concerns	Low risk	No concerns	Major concerns	No concerns	No concerns	Low	[Imprecision]
TopirCloz:Zot	0	Major concerns	Low risk	No concerns	Some concerns	Some concerns	No concerns	Low	[Within-study bias, Imprecision, Heterogeneity]
VitD3Cloz:VortRisp	0	No concerns	Low risk	Some concerns	Major concerns	No concerns	No concerns	Low	[Indirectness, Imprecision]
VitD3Cloz:Zip	0	No concerns	Low risk	Some concerns	Major concerns	No concerns	No concerns	Low	[Indirectness, Imprecision]
VitD3Cloz:ZipCloz	0	No concerns	Low risk	No concerns	Some concerns	Some concerns	No concerns	Moderate	[Imprecision, Heterogeneity]
VitD3Cloz:Zot	0	No concerns	Low risk	Some concerns	Major concerns	No concerns	No concerns	Low	[Indirectness, Imprecision]
VortRisp:Zip	0	Some concerns	Low risk	No concerns	No concerns	No concerns	No concerns	Moderate	[Within-study bias]
VortRisp:ZipCloz	0	No concerns	Low risk	No concerns	Major concerns	No concerns	No concerns	Low	[Imprecision]
VortRisp:Zot	0	No concerns	Low risk	Some concerns	Some concerns	Some concerns	No concerns	Low	[Indirectness, Imprecision, Heterogeneity]
Zip:ZipCloz	0	No concerns	Low risk	No concerns	No concerns	No concerns	No concerns	High	
Zip:Zot	0	Some concerns	Low risk	Some concerns	Major concerns	No concerns	No concerns	Low	[Within-study bias, Indirectness, Imprecision]
ZipCloz:Zot	0	Major concerns	Low risk	No concerns	No concerns	Some concerns	No concerns	Low	[Within-study bias, Heterogeneity]

Table S 14.2. CINeMA rating for all comparisons: Overall symptoms primary outcome

AMI: amisulpride; ARI: aripiprazole; BEZOAET: benzoate sodium; CELECOX: celecoxib; CEREBR: cerebrolysin; CLOPENTHLA: clopenthixol; CLOT: clotiapine; CLOZ: clozapine; CPZ: chlorpromazine; Dcyclos: D-cycloserine; DESMOP: desmopressin; DONEP: donepezil; Dser: D-serine; DUL: duloxetine; FLUO: fluoxetine; FLUPH: fluphenazine; FLUPHLA: fluphenazine decanoate; FLUV: fluvoxamine; GinkBil: Ginkgo biloba; GLY: glycine; HAL: haloperidol; IM: intravascular; IMI: imipramine; LAM: lamotrigine; LI: lithium; MAZIN: mazindol; MEM: memantine; MET: metformin; MINOC: minocycline; MIRT: mirtazapine; MODF: modafinil; Nacetyl: N-acetylcysteine; NS: normal saline; ONDAST: ondansetron; OLA: olanzapine; OXYT: oxytocin; PALIP: paliperidone; PALMIT: palmitoylethanolamide; PBO: placebo; PERP: perphenazine; PHENYLPROP: phenylpropanolamine; PIMOZ: pimozide; PIPOTLA: pipotiazine long-acting; PTX: pentoxifylline; QUE: quetiapine; RESVER: resveratrol; RISP: risperidone; SARC: sarcosine; SER: sertraline; SERTIND: sertindole; SUL: sulpiride; THIO: thioridazine; TOPIR: topiramate; TRIFLUO: trifluoperazine; VALPRO: valproate; VitD3: vitamin D3; VORT: vortioxetine; ZIP: ziprasidone; ZOT: zotepine.

Appendix 15. Sub-group and meta-regression analysis

15.1. Sub-group: Criteria of treatment-resistant definitions

Criteria for treatment resistance were based on the following four categories: (1) ‘one previously failed antipsychotic trial, based on historical information prior to entering the study’; (2) ‘at least 2 previously failed antipsychotic trials, based on historical information prior to entering the study’; (3) ‘a combination of retrospective (historical information prior to entering the study) and prospective (failed trials as part of the study design) criteria for treatment resistance’; (4) ‘no or partial response to clozapine (ultra treatment-resistant)’.

15.1.1. Sub-group: Treatment-resistant criteria – Group 1 versus Groups 2 to 4

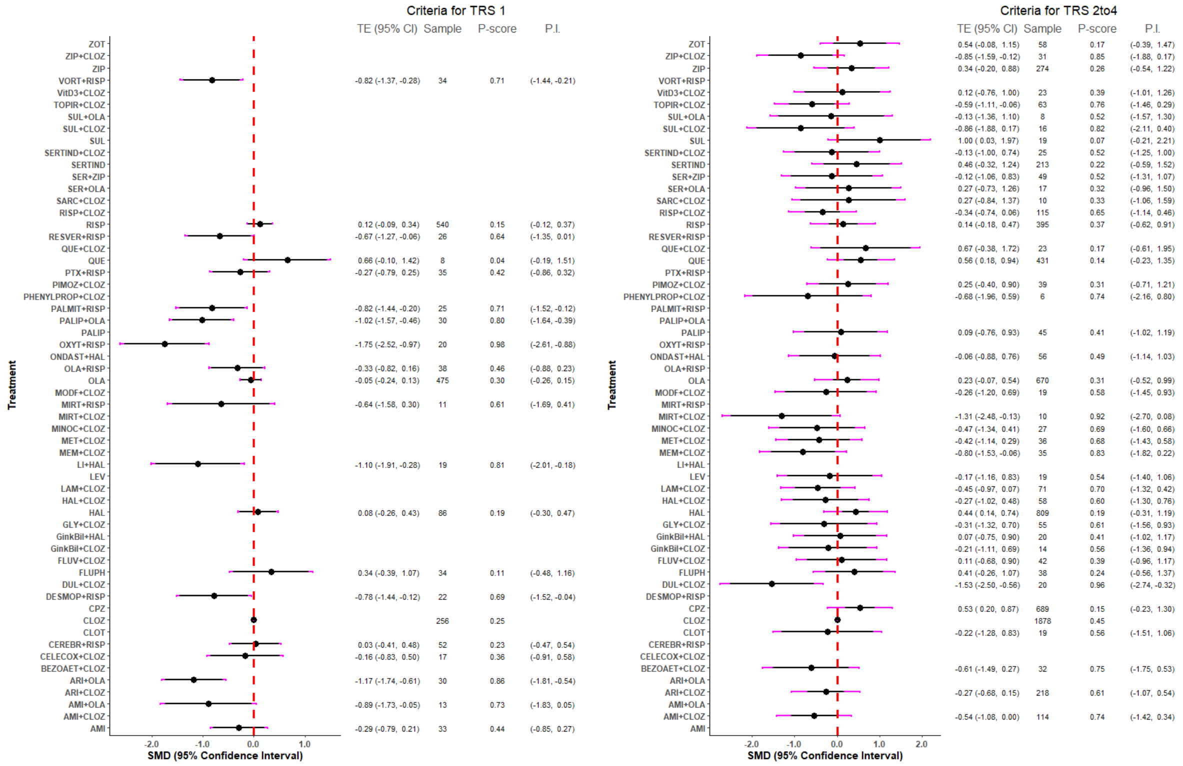

Figure S 15.1.1. Forest plot with prediction intervals for treatment-resistant criteria: Group 1 versus Groups 2 to 4

AMI: amisulpride; ARI: aripiprazole; BEZOAET: benzoate sodium; CELECOX: celecoxib; CEREBR: cerebrolysin; CI: confidence intervals; CLOT: clotiapine; CLOZ: clozapine; CPZ: chlorpromazine; DESMOP: desmopressin; DUL: duloxetine; FLUPH: fluphenazine; FLUV: fluvoxamine; GinkBil: Ginkgo biloba; GLY: glycine; HAL: haloperidol; LAM: lamotrigine; LEV: levomepromazine; LI: lithium; MEM: memantine; MET: metformin; MINOC: minocycline; MIRT: mirtazapine; MODF: modafinil; OLA: olanzapine; ONDAST: ondansetron; OXYT: oxytocin; PALIP: paliperidone; PALMIT: palmitoylethanolamide; PHENYLPROP: phenylpropanolamine; PIMOZ: pimozide; PTX: pentoxifylline; QUE: quetiapine; RESVER: resveratrol; RISP: risperidone; SARC: sarcosine; SER: sertraline; SERTIND: sertindole; SMD: standardized mean difference; SUL: sulpiride; TOPIR: topiramate; TRS: treatment-resistant; VitD3: vitamin D3; VORT: vortioxetine; ZIP: ziprasidone; ZOT: zotepine.

15.1.2. Sub-group: Treatment-resistant criteria – Groups 1 to 2 versus Group 3 to 4

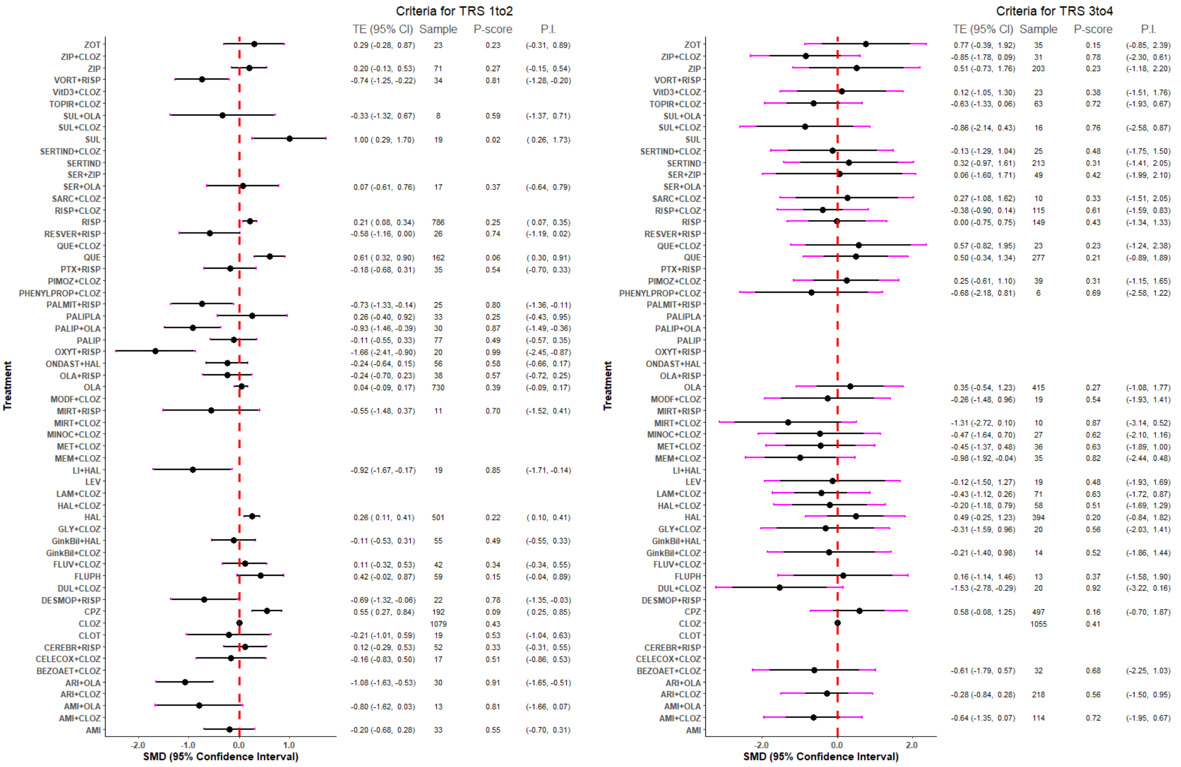

Figure S 15.1.2. Forest plot with prediction intervals for treatment-resistant criteria: Group 1 to 3 vs Group 4

AMI: amisulpride; ARI: aripiprazole; BEZOAET: benzoate sodium; CELECOX: celecoxib; CEREBR: cerebrolysin; CI: confidence intervals; CLOT: clotiapine; CLOZ: clozapine; CPZ: chlorpromazine; DESMOP: desmopressin; DUL: duloxetine; FLUPH: fluphenazine; FLUV: fluvoxamine; GinkBil: Ginkgo biloba; GLY: glycine; HAL: haloperidol; LAM: lamotrigine; LEV: levomepromazine; LI: lithium; MEM: memantine; MET: metformin; MINOC: minocycline; MIRT: mirtazapine; MODF: modafinil; OLA: olanzapine; ONDAST: ondansetron; OXYT: oxytocin; PALIP: paliperidone; PALMIT: palmitoylethanolamide; PHENYLPROP: phenylpropanolamine; PIMOZ: pimozide; PTX: pentoxifylline; QUE: quetiapine; RESVER: resveratrol; RISP: risperidone; SARC: sarcosine; SER: sertraline; SERTIND: sertindole; SMD: standardized mean difference; SUL: sulpiride; TOPIR: topiramate; TRS: treatment-resistant; VitD3: vitamin D3; VORT: vortioxetine; ZIP: ziprasidone; ZOT: zotepine.

15.1.3. Sub-group: Treatment-resistant criteria – Groups 1 to 3 versus Groups 4 (ultra resistant)

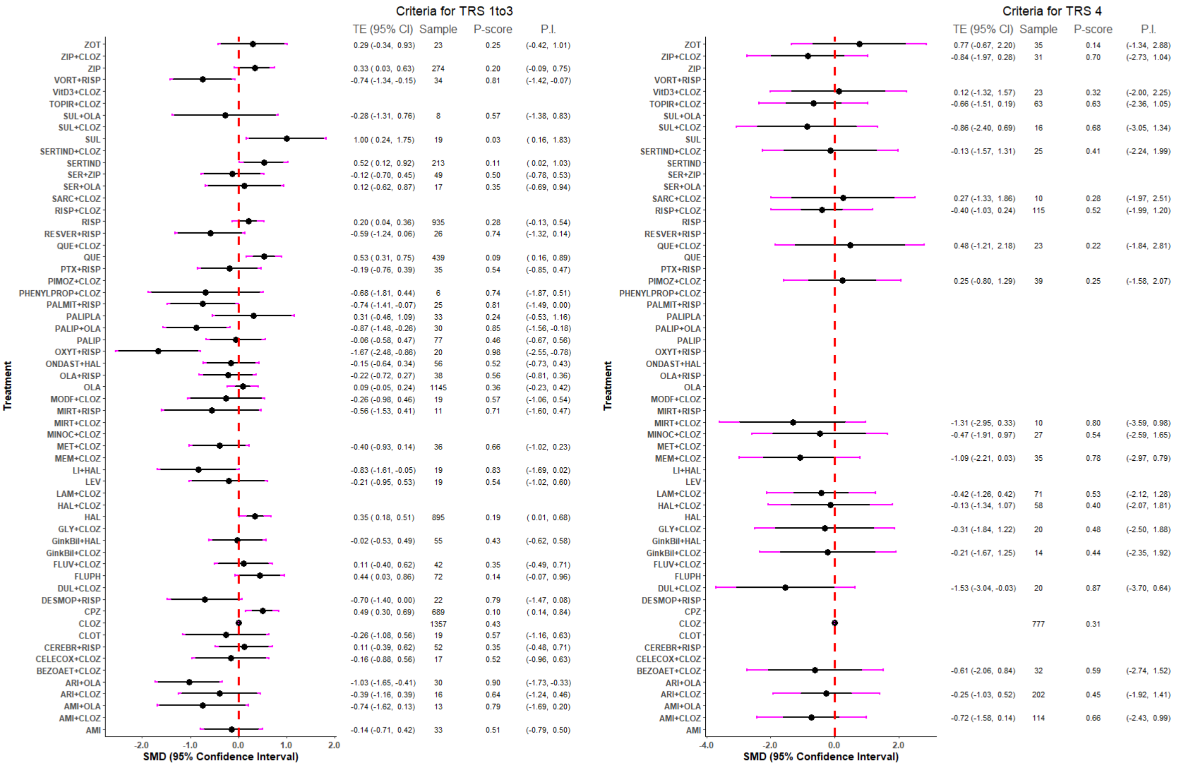

Figure S 15.1.3. Forest plot with prediction intervals for treatment-resistant criteria: Groups 1 to 3 versus Groups 4 (ultra resistant)

AMI: amisulpride; ARI: aripiprazole; BEZOAET: benzoate sodium; CELECOX: celecoxib; CEREBR: cerebrolysin; CI: confidence intervals; CLOT: clotiapine; CLOZ: clozapine; CPZ: chlorpromazine; DESMOP: desmopressin; DUL: duloxetine; FLUPH: fluphenazine; FLUV: fluvoxamine; GinkBil: Ginkgo biloba; GLY: glycine; HAL: haloperidol; LAM: lamotrigine; LEV: levomepromazine; LI: lithium; MEM: memantine; MET: metformin; MINOC: minocycline; MIRT: mirtazapine; MODF: modafinil; OLA: olanzapine; ONDAST: ondansetron; OXYT: oxytocin; PALIP: paliperidone; PALMIT: palmitoylethanolamide; PHENYLPROP: phenylpropanolamine; PIMOZ: pimozide; PTX: pentoxifylline; QUE: quetiapine; RESVER: resveratrol; RISP: risperidone; SARC: sarcosine; SER: sertraline; SERTIND: sertindole; SMD: standardized mean difference; SUL: sulpiride; TOPIR: topiramate; TRS: treatment-resistant; VitD3: vitamin D3; VORT: vortioxetine; ZIP: ziprasidone; ZOT: zotepine.

15.2. Sub-group: Residual symptoms

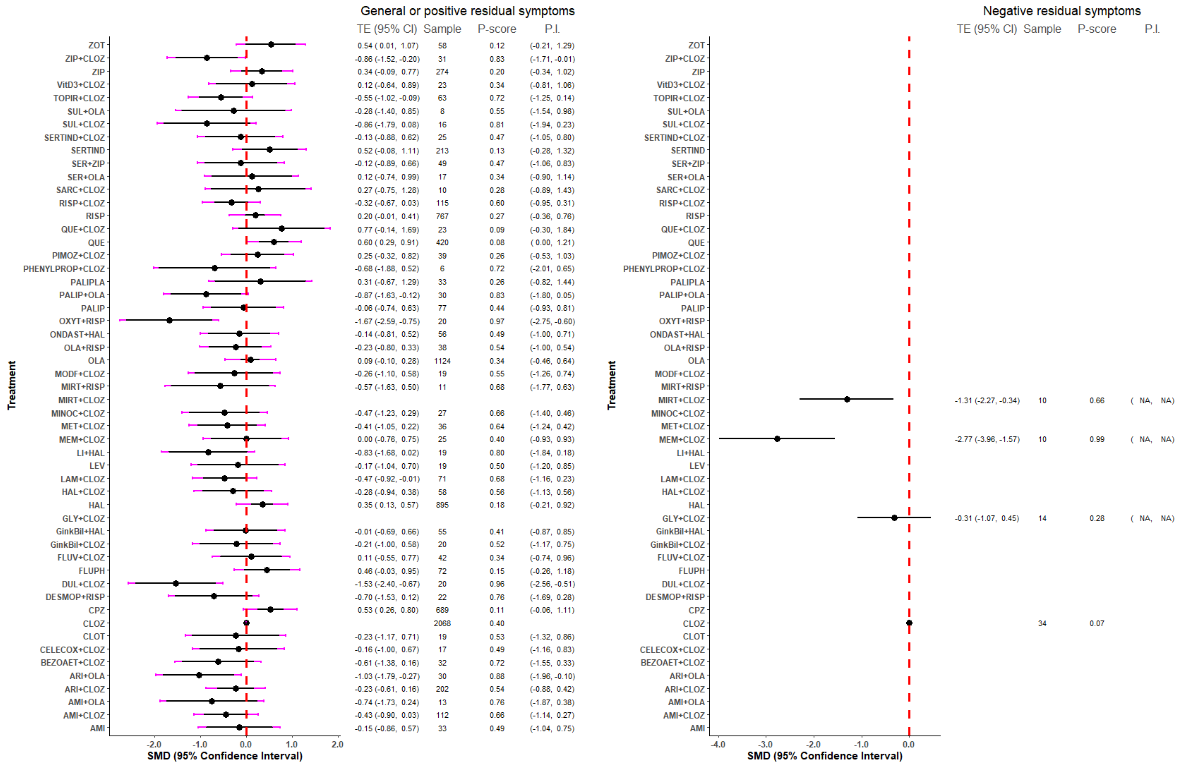

Figure S 15.2.3. Forest plot with prediction intervals for Residual symptoms: (1) General or positive and (2) negative residual symptoms

AMI: amisulpride; ARI: aripiprazole; BEZOAET: benzoate sodium; CELECOX: celecoxib; CEREBR: cerebrolysin; CI: confidence intervals; CLOT: clotiapine; CLOZ: clozapine; CPZ: chlorpromazine; DESMOP: desmopressin; DUL: duloxetine; FLUPH: fluphenazine; FLUV: fluvoxamine; GinkBil: Ginkgo biloba; GLY: glycine; HAL: haloperidol; LAM: lamotrigine; LEV: levomepromazine; LI: lithium; MEM: memantine; MET: metformin; MINOC: minocycline; MIRT: mirtazapine; MODF: modafinil; OLA: olanzapine; ONDAST: ondansetron; OXYT: oxytocin; PALIP: paliperidone; PALMIT: palmitoylethanolamide; PHENYLPROP: phenylpropanolamine; PIMOZ: pimozide; QUE: quetiapine; RESVER: resveratrol; RISP: risperidone; SARC: sarcosine; SER: sertraline; SERTIND: sertindole; SMD: standardized mean difference; SUL: sulpiride; TOPIR: topiramate; VitD3: vitamin D3; VORT: vortioxetine; ZIP: ziprasidone; ZOT: zotepine.

15.3. Sub-group: Treatment groups based on their main therapeutic concept (Figure 15.3.1.; Figure 15.3.2)

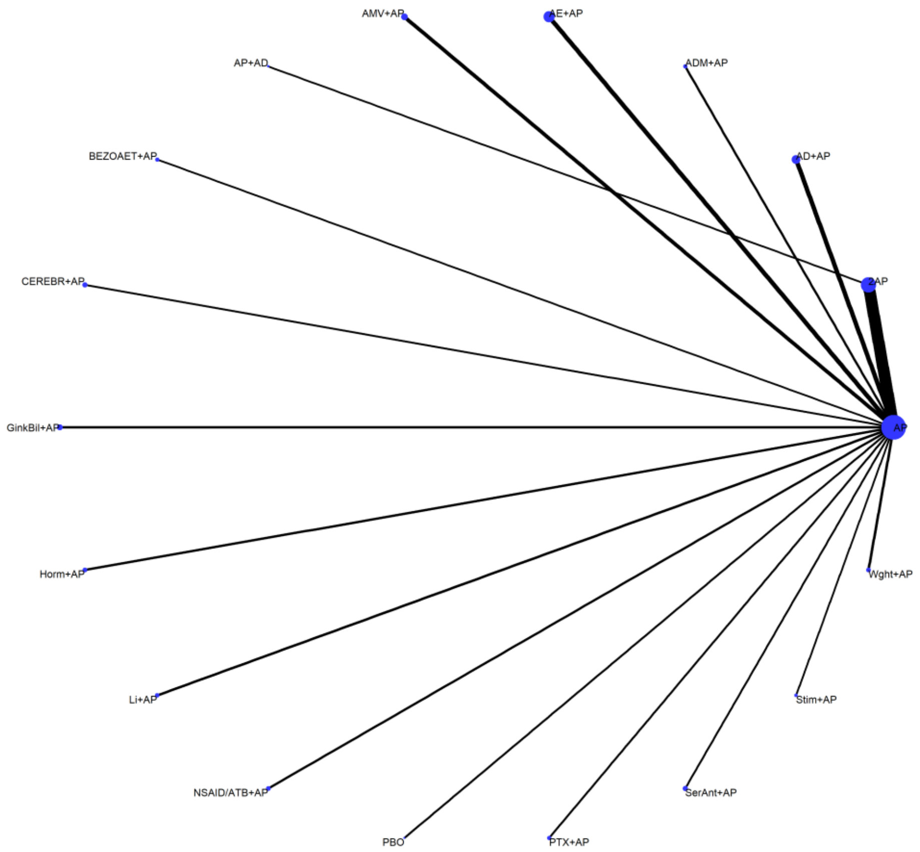

Figure S15.3.1. Network plot for treatment groups based on their main therapeutic concept
ADM: anti-dementia medicine; AD: antidepressants; AE: antiepileptic; AMV: amino acid variant; AP: antipsychotics; BEZOAET: benzoate sodium; CEREBR: cerebrolysin; Horm: hormones; LI: lithium; NSAID/ATB: non-steroidal anti-inflammatory drug/antibiotic; PBO: placebo; PTX: pentoxifylline; SerAnt: serotonin antagonist; Stim: stimulant; Wght: weight management.

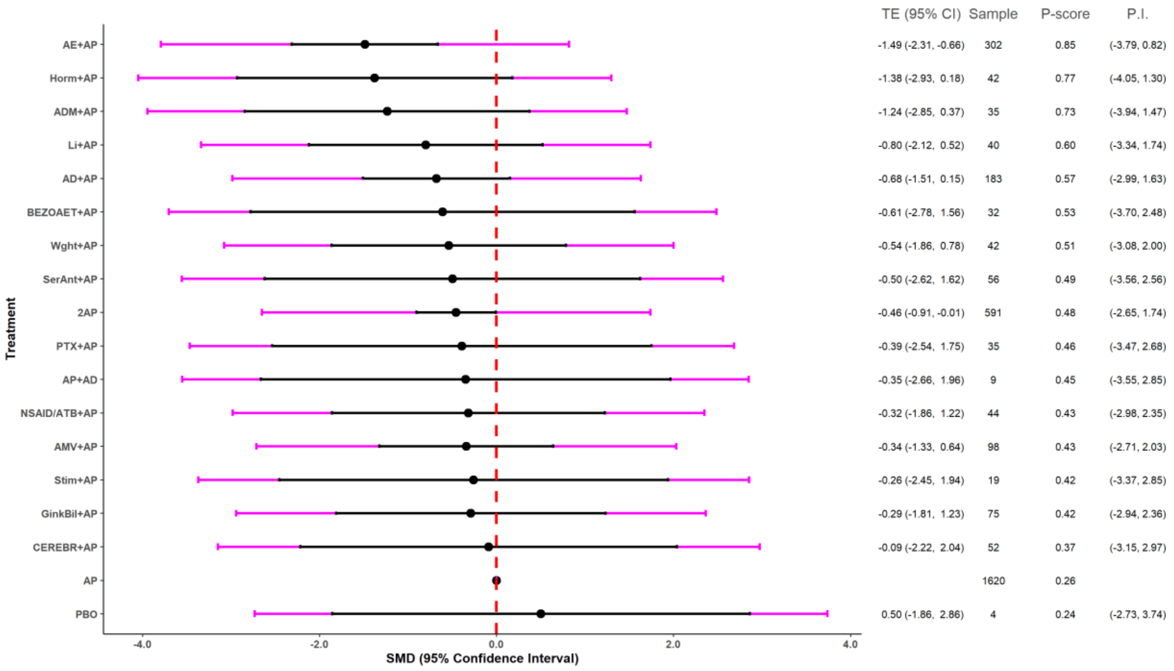

Figure S 15.3.2. Forest plot with prediction intervals for treatment groups based on their main therapeutic concept

Reference comparator: Antipsychotic monotherapy. ADM: anti-dementia medicine; AE: antiepileptic; AMV: amino acid variant; AP: antipsychotics; BEZOAET: benzoate sodium; CEREBR: cerebrolysin; CI: confidence intervals; Horm: hormones; LI: lithium; NSAID/ATB: non-steroidal anti-inflammatory drug/antibiotic; PBO: placebo; PTX: pentoxifylline; SerAnt: serotonin antagonist; SMD: standardized mean difference; Stim: stimulant; Wght: weight management.

15.4. Sub-group: Sponsorship: Sponsored vs non-sponsored

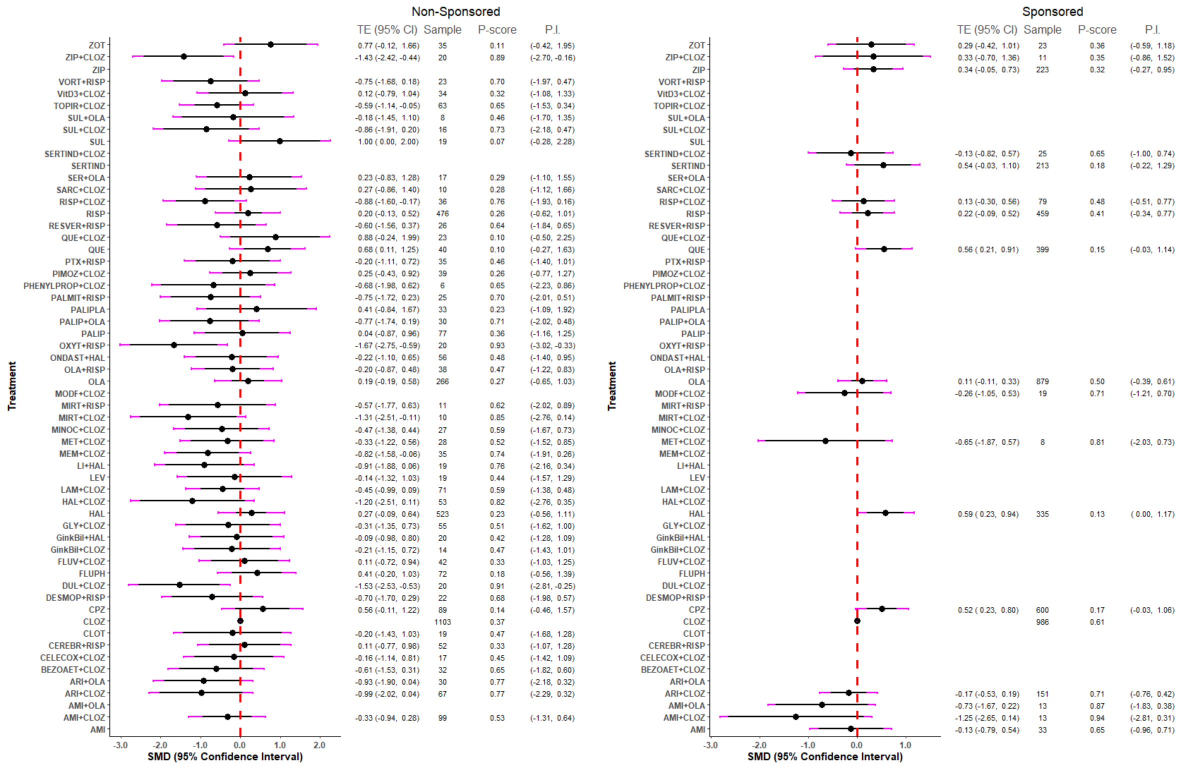

Figure S 15.7. Forest plot with prediction intervals for sponsorship: Sponsored vs non-sponsored RCTs
Reference comparator: Clozapine. AMI: amisulpride; ARI: aripiprazole; BEZOAET: benzoate sodium; CELECOX: celecoxib; CEREBR: cerebrolysin; CI: confidence intervals; CLOZ: clozapine; CPZ: chlorpromazine; DESMOP: desmopressin; DUL: duloxetine; FLUPH: fluphenazine; FLUV: fluvoxamine; GinkBil: Ginkgo biloba; GLY: glycine; HAL: haloperidol; LAM: lamotrigine; LEV: levomepromazine; LI: lithium; MEM: memantine; MET: metformin; MINOC: minocycline; MIRT: mirtazapine; MODF: modafinil; OLA: olanzapine; ONDAST: ondansetron; OXYT: oxytocin; PALIP: paliperidone; PALMIT: palmitoylethanolamide; PHENYLPROP: phenylpropanolamine; PIMOZ: pimozide; PTX: pentoxifylline; QUE: quetiapine; RESVER: resveratrol; RISP: risperidone; SARC: sarcosine; SER: sertraline; SERTIND: sertindole; SMD: standardized mean difference; SUL: sulpiride; TOPIR: topiramate; VitD3: vitamin D3; VORT: vortioxetine; ZIP: ziprasidone; ZOT: zotepine.

15.5. Meta-regression: Baseline severity

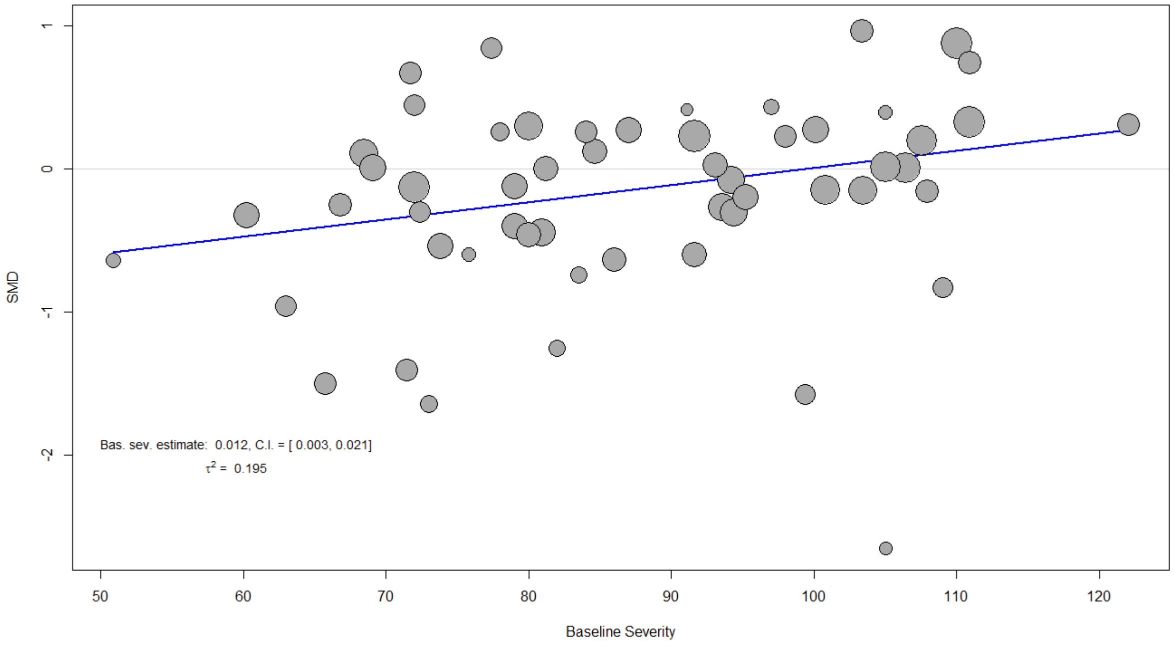

Figure S 15.5. Meta-Regression: Baseline severity
CI: confidence intervals; SMD: standardized mean difference.

15.6. Meta-regression: Publication date

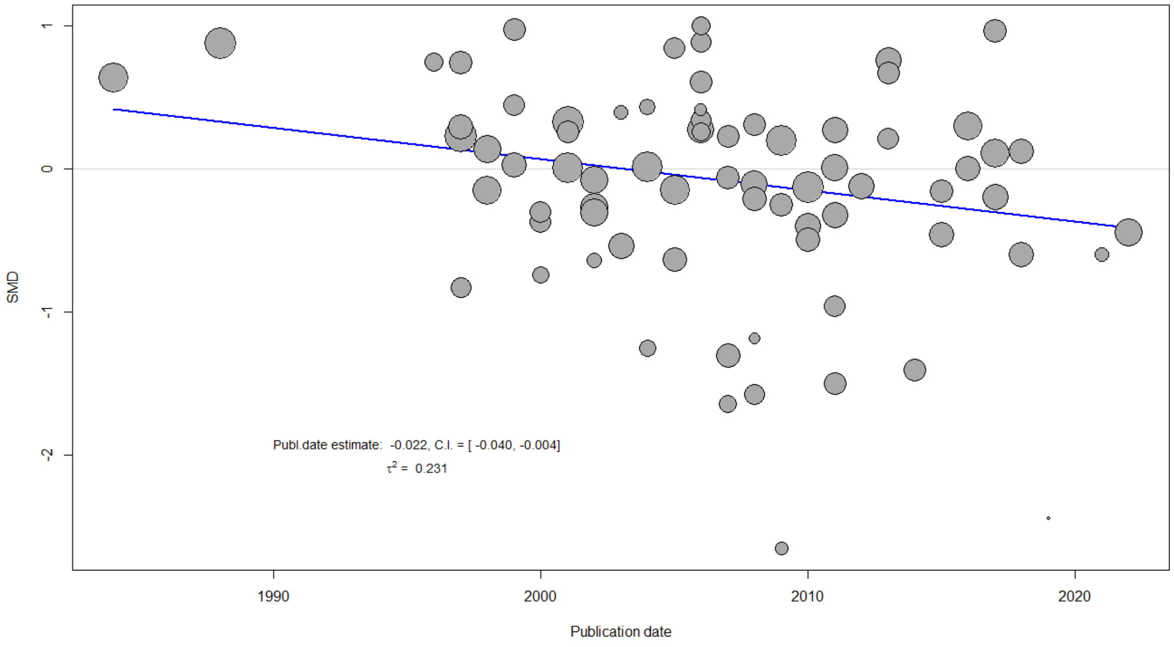

Figure S 15.6. Meta-Regression: Publication date
CI: confidence intervals; SMD: standardized mean difference.

15.7. Meta-Regression: Age

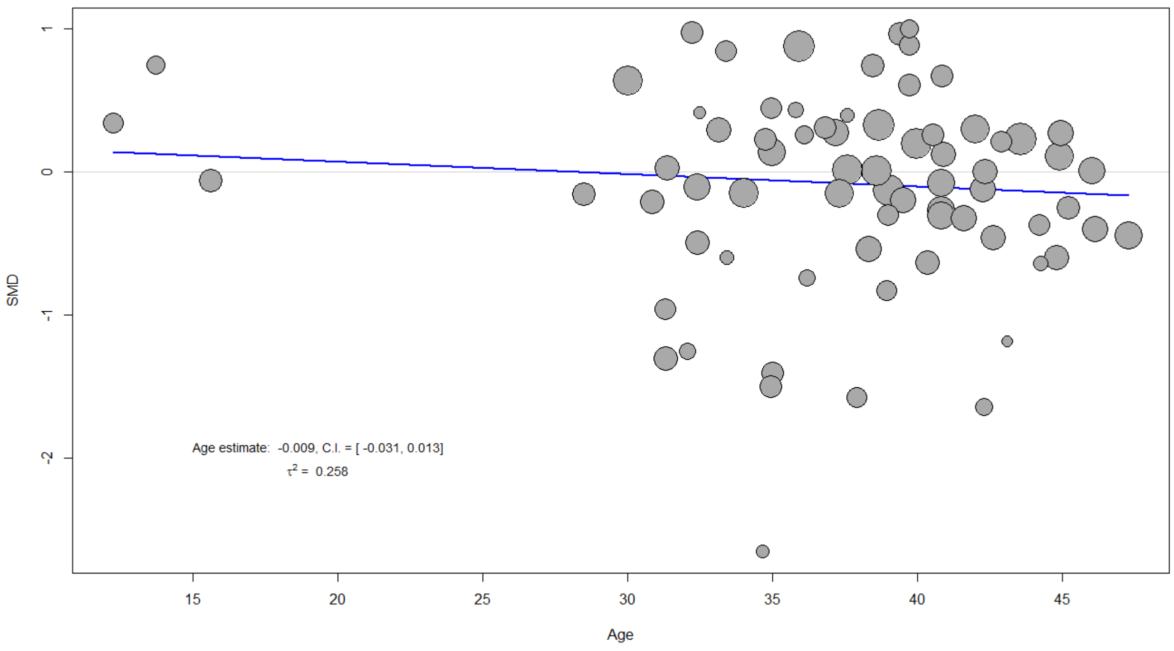

Figure S 15.7. Meta-Regression: Age
CI: confidence intervals; SMD: standardized mean difference.

15.8. Meta-regression: Percentage of males

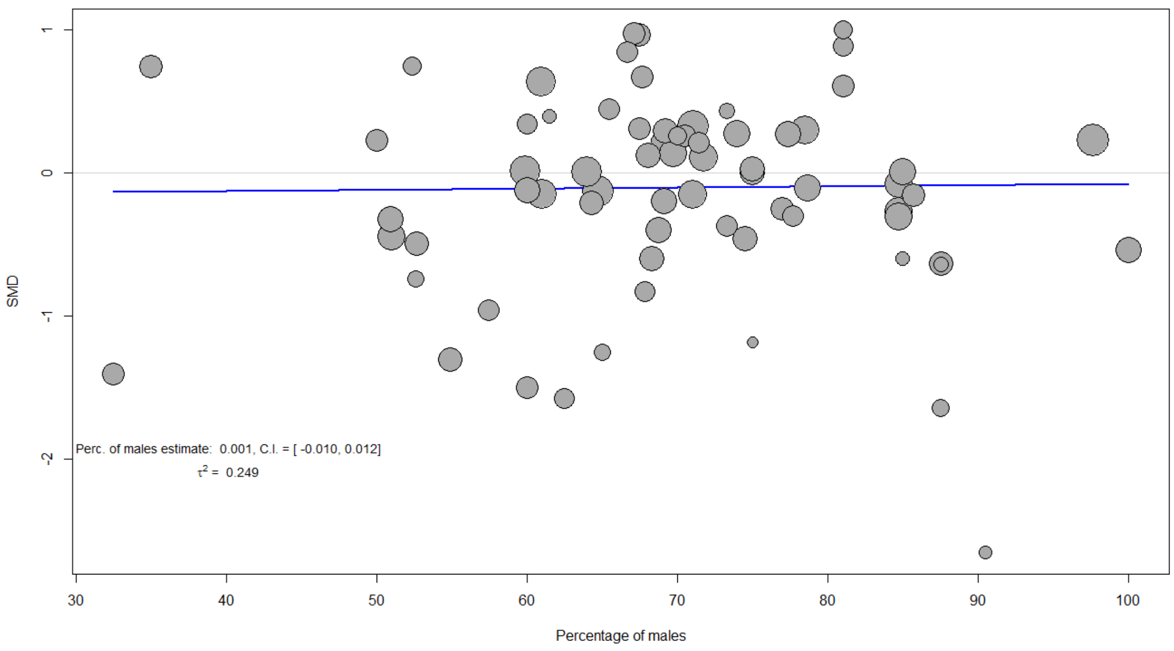

Figure S 15.8. Meta-Regression: Percentage of males
CI: confidence intervals; SMD: standardized mean difference.

15.9. Meta-regression: Dose ratio

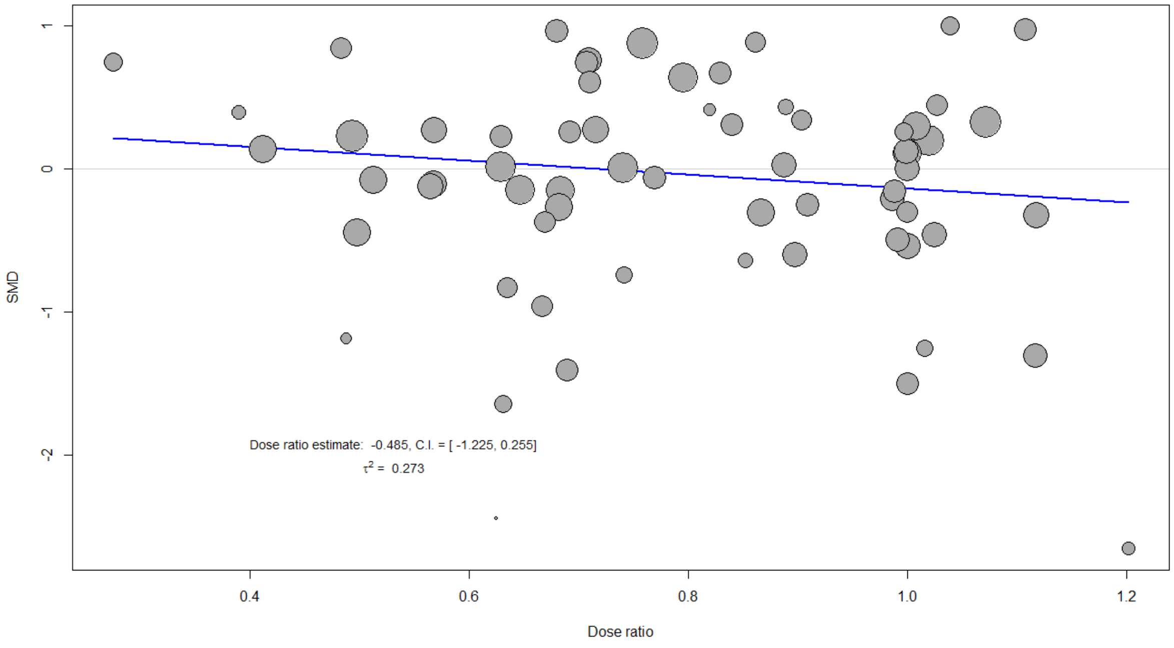

Figure S 15.9. Meta-Regression: Dose ratio
CI: confidence intervals; SMD: standardized mean difference.

15.10. Meta-regression: Duration of illness

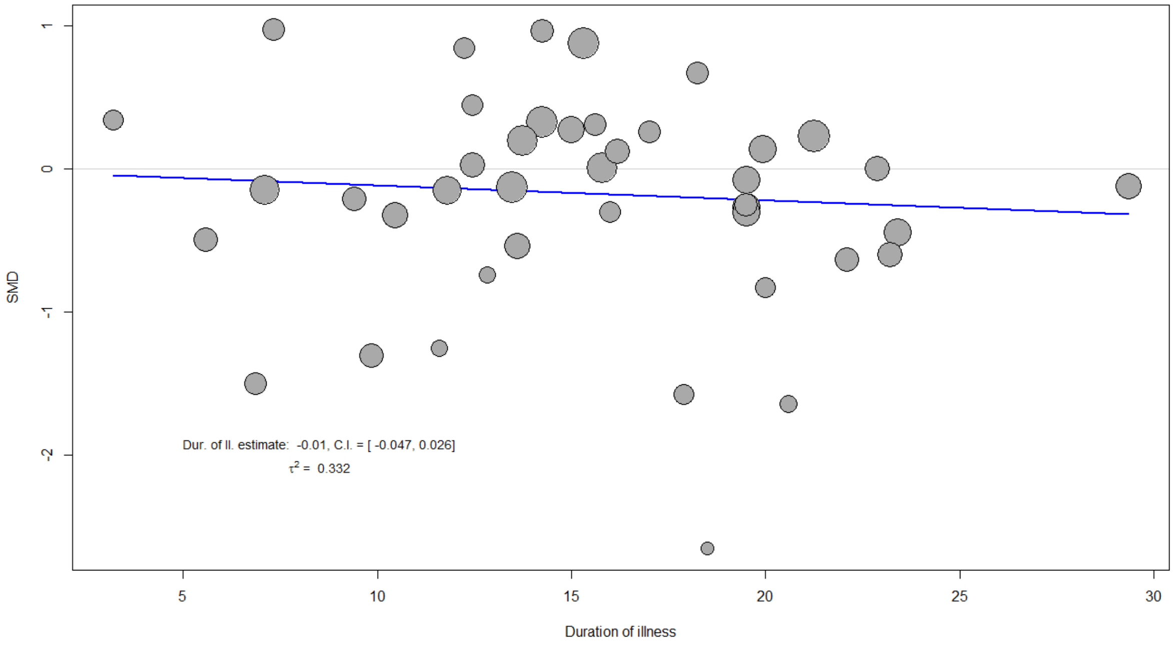

Figure S 15.10. Meta-Regression: Duration of illness
CI: confidence intervals; SMD: standardized mean difference.

15.11. Meta-regression: Duration of trial

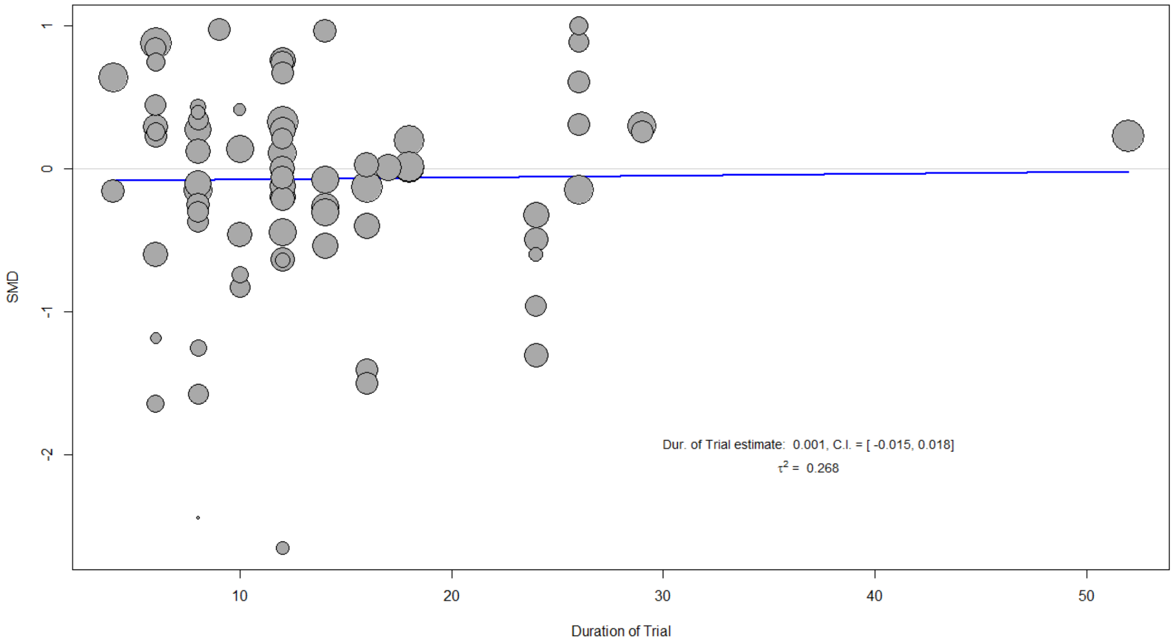

Figure S 15.11. Meta-Regression: Duration of trial
CI: confidence intervals; SMD: standardized mean difference.

15.12. Multivariable meta-regression analyses

Our univariable meta-regression analyses identified baseline severity and publication date as potential effect modifiers. However, when these moderators were analysed concurrently, the apparent effect of publication year disappeared. This suggests that the observed temporal trend was likely confounded by baseline severity differences, with earlier studies tending to include patients with more severe symptoms. The multivariable meta-regression analyses with all the potential moderators included found no effect, but its statistical power was extremely low since trials with at least one missing value was excluded from the analysis.

Covariate	Estimate	Lower bound	Upper bound
Multivariable with baseline severity and publication date analysed concurrently			
Baseline Severity	0.0104	0.0003	0.0205*
Publication date	-0.0087	-0.0301	0.0127
Multivariable with all possible moderators included			
Age	0.036	-0.069	0.141
Perc. Of males	-0.006	-0.029	0.017
Baseline Severity	0.008	-0.008	0.024
Illness duration	-0.031	-0.108	0.047
Publication date	0	-0.045	0.045
Trial duration	0.006	-0.021	0.033
Dose ratio	-0.729	-2.039	0.580

Appendix 16. Sensitivity analyses

Sensitivity analysis involves repeating the meta-analysis after excluding studies with specific characteristics or assumptions and comparing the results of these modified analyses to the primary (original) analysis. This allows the assessment of the robustness of the main findings and determine whether certain studies disproportionately influence the results. In a series of predetermined sensitivity analyses, studies with specific characteristics were excluded.

16.1. Exclusion of the first quartile of trials with the smallest sample sizes (Figure S 16.1a; Figure S 16.1b)

The first quartile of trials with the smallest sample sizes was excluded since small studies are more prone to bias and may disproportionately influence the results.

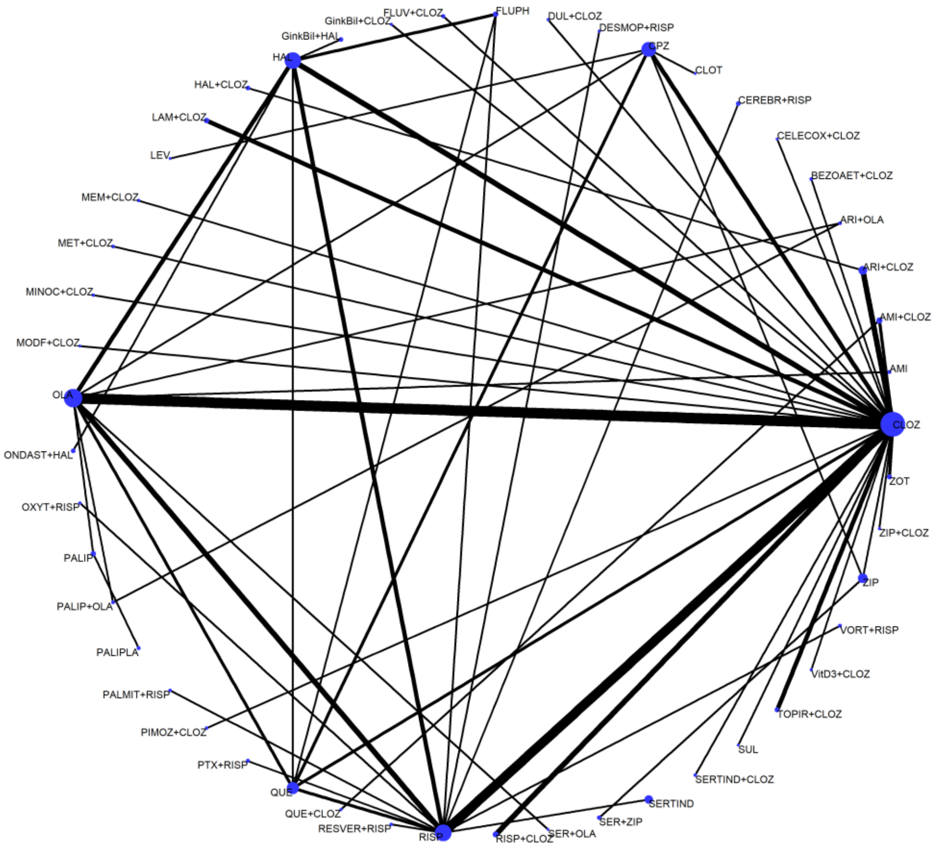

Figure S 16.1a. Network plot after excluding all studies falling at or below the 25th percentile (first quartile, Q1) in terms of sample size.
AMI: amisulpride; ARI: aripiprazole; BEZOAET: benzoate sodium; CELECOX: celecoxib; CEREBR: cerebrolysin; CLOT: clotiapine; CLOZ: clozapine; CPZ: chlorpromazine; DESMOP: desmopressin; DUL: duloxetine; FLUPH: fluphenazine; FLUV: fluvoxamine; GinkBil: Ginkgo biloba; GLY: glycine; HAL: haloperidol; LAM: lamotrigine; LEV: levomepromazine; LI: lithium; MEM: memantine; MET: metformin; MINOC: minocycline; MIRT: mirtazapine; MODF: modafinil; OLA: olanzapine; ONDAST: ondansetron; OXYT: oxytocin; PALIP: paliperidone; PALMIT: palmitoylethanolamide; PHENYLPROP: phenylpropanolamine; PIMOZ: pimozide; PTX: pentoxifylline; QUE: quetiapine; RESVER: resveratrol; RISP: risperidone; SER: sertraline; SERTIND: sertindole; SUL: sulpiride; TOPIR: topiramate; VitD3: vitamin D3; VORT: vortioxetine; ZIP: ziprasidone; ZOT: zotepine.

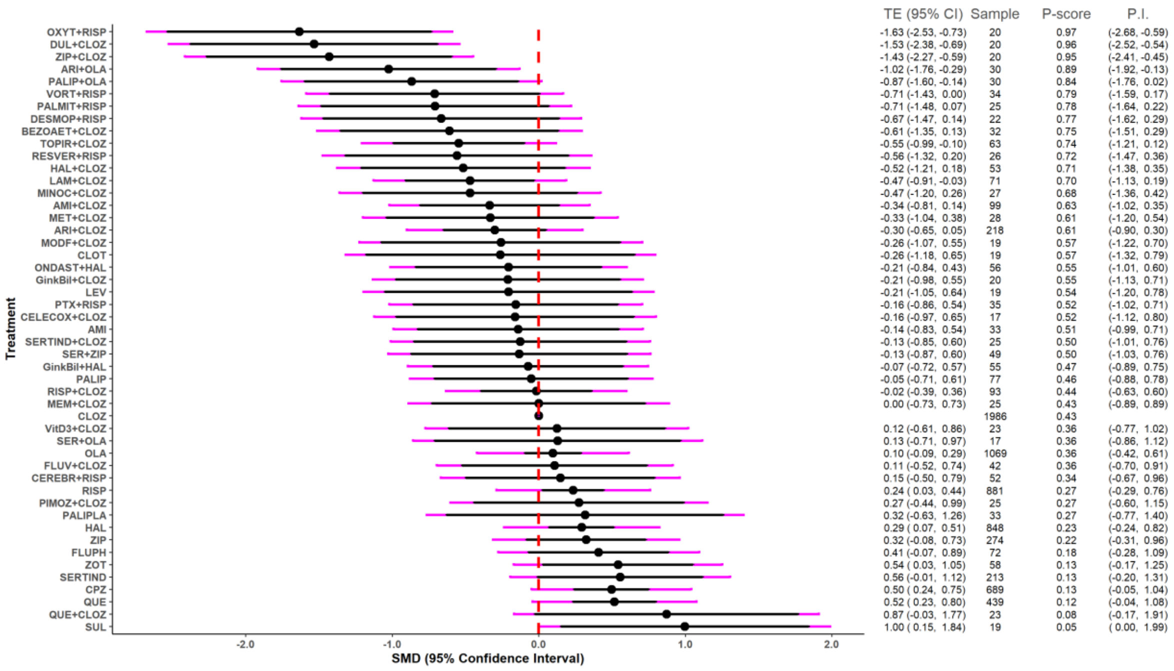

Figure S 16.1b. Forest plot with prediction intervals after excluding all studies falling at or below the 25th percentile (first quartile, Q1) in terms of sample size.

Reference comparator: Clozapine. AMI: amisulpride; ARI: aripiprazole; BEZOAET: benzoate sodium; CELECOX: celecoxib; CEREBR: cerebrolysin; CI: confidence intervals; CLOT: clotiapine; CLOZ: clozapine; CPZ: chlorpromazine; DESMOP: desmopressin; DUL: duloxetine; FLUPH: fluphenazine; FLUV: fluvoxamine; GinkBil: Ginkgo biloba; GLY: glycine; HAL: haloperidol; LAM: lamotrigine; LEV: levomepromazine; LI: lithium; MEM: memantine; MET: metformin; MINOC: minocycline; MIRT: mirtazapine; MODF: modafinil; OLA: olanzapine; ONDAST: ondansetron; OXYT: oxytocin; PALIP: paliperidone; PALMIT: palmitoylethanolamide; PHENYLPROP: phenylpropanolamine; PIMOZ: pimozide; PTX: pentoxifylline; QUE: quetiapine; RESVER: resveratrol; RISP: risperidone; SER: sertraline; SERTIND: sertindole; SMD: standardized mean difference; SUL: sulpiride; TOPIR: topiramate; VitD3: vitamin D3; VORT: vortioxetine; ZIP: ziprasidone; ZOT: zotepine.

16.2. Double blind randomised control trials only (Figure S 16.2a; Figure S 16.2b)

Double-blind RCTs are considered the gold standard for minimizing bias. Non-double-blind studies may introduce bias due to lack of blinding.

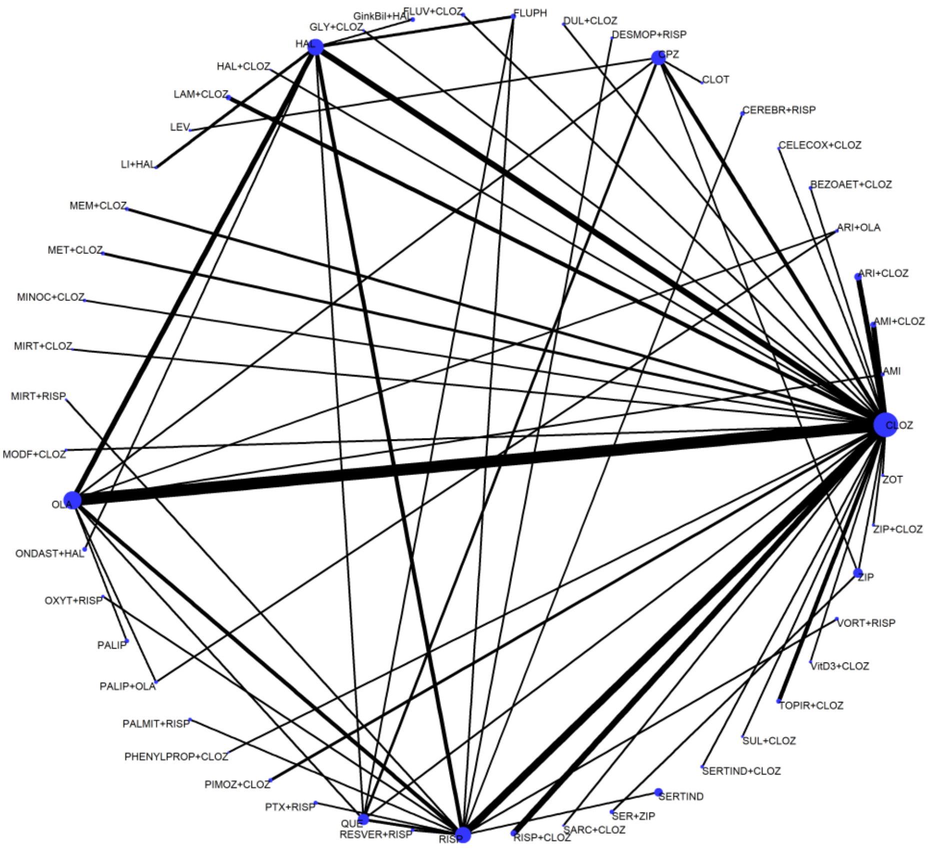

Figure S 16.2a. Network plot for double blind RCTs only
AMI: amisulpride; ARI: aripiprazole; BEZOAET: benzoate sodium; CELECOX: celecoxib; CEREBR: cerebrolysin; CLOT: clotiapine; CLOZ: clozapine; CPZ: chlorpromazine; DESMOP: desmopressin; DUL: duloxetine; FLUPH: fluphenazine; FLUV: fluvoxamine; GinkBil: Ginkgo biloba; GLY: glycine; HAL: haloperidol; LAM: lamotrigine; LEV: levomepromazine; LI: lithium; MEM: memantine; MET: metformin; MINOC: minocycline; MIRT: mirtazapine; MODF: modafinil; OLA: olanzapine; ONDAST: ondansetron; OXYT: oxytocin; PALIP: paliperidone; PALMIT: palmitoylethanolamide; PHENYLPROP: phenylpropanolamine; PIMOZ: pimozide; PTX: pentoxifylline; QUE: quetiapine; RESVER: resveratrol; RISP: risperidone; SARC: sarcosine; SER: sertraline; SERTIND: sertindole; SUL: sulpiride; TOPIR: topiramate; VitD3: vitamin D3; VORT: vortioxetine; ZIP: ziprasidone; ZOT: zotepine.

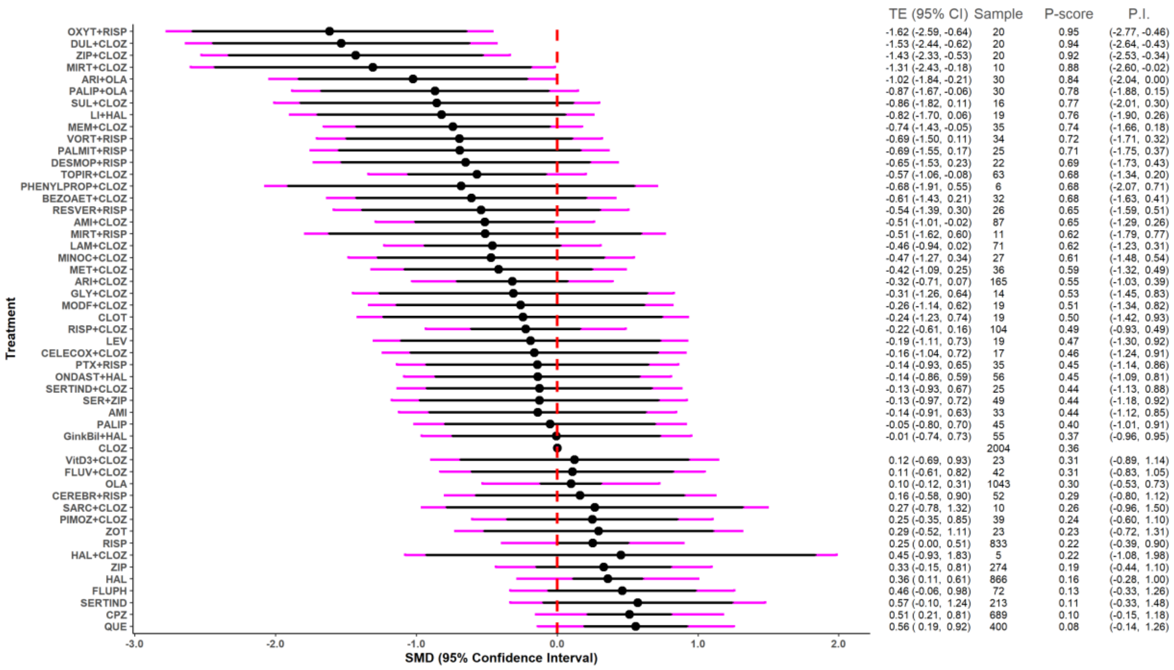

Figure S 16.2b. Forest plot with prediction intervals for double blind RCTs only

Reference comparator: Clozapine. AMI: amisulpride; ARI: aripiprazole; BEZOAET: benzoate sodium; CELECOX: celecoxib; CEREBR: cerebrolysin; CI: confidence intervals; CLOT: clotiapine; CLOZ: clozapine; CPZ: chlorpromazine; DESMOP: desmopressin; DUL: duloxetine; FLUPH: fluphenazine; FLUV: fluvoxamine; GinkBil: Ginkgo biloba; GLY: glycine; HAL: haloperidol; LAM: lamotrigine; LEV: levomepromazine; LI: lithium; MEM: memantine; MET: metformin; MINOC: minocycline; MIRT: mirtazapine; MODF: modafinil; OLA: olanzapine; ONDAST: ondansetron; OXYT: oxytocin; PALIP: paliperidone; PALMIT: palmitoylethanolamide; PHENYLPROP: phenylpropanolamine; PIMOZ: pimozide; PTX: pentoxifylline; QUE: quetiapine; RESVER: resveratrol; RISP: risperidone; SARC: sarcosine; SER: sertraline; SERTIND: sertindole; SMD: standardized mean difference; SUL: sulpiride; TOPIR: topiramate; VitD3: vitamin D3; VORT: vortioxetine; ZIP: ziprasidone; ZOT: zotepine.

16.3. Exclusion of randomised control trials that did not employ operationalized criteria for schizophrenia diagnosis (Figure S 16.3a; Figure S 16.3b)

Excluding studies that did not use standardized diagnostic criteria (e.g., DSM or ICD) ensures that the patient populations across studies are comparable and diagnoses are reliable.

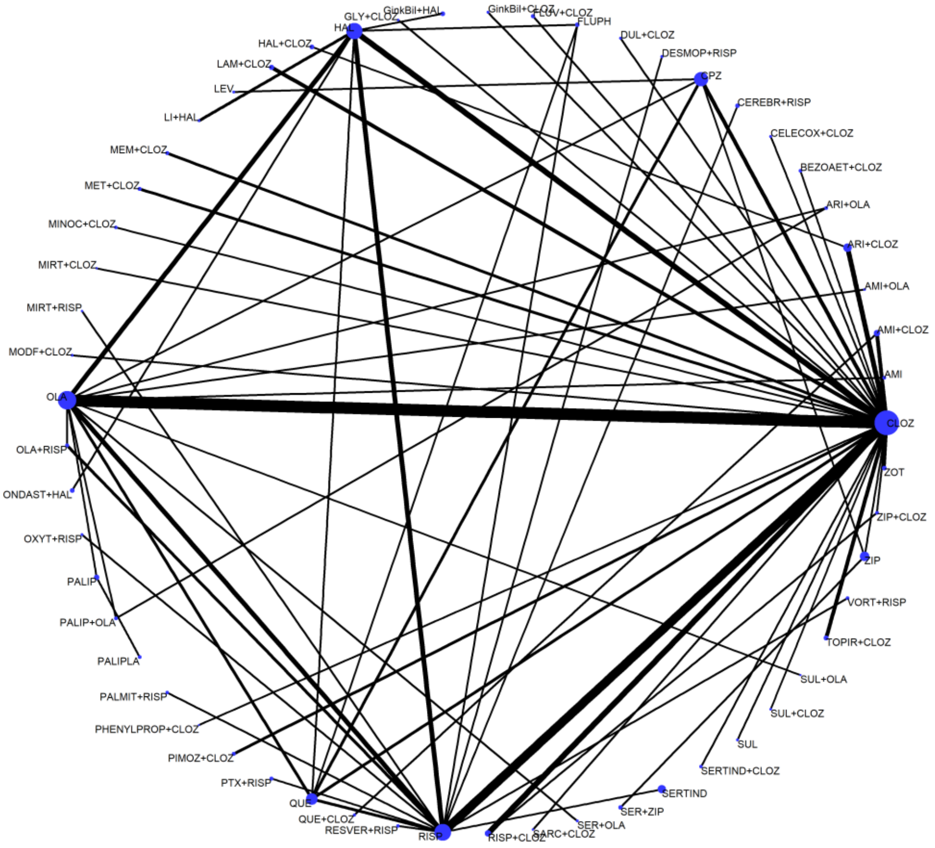

Figure S 16.3a. Network plot for the exclusion of RCTs that did not employ operationalized criteria for schizophrenia diagnosis

AMI: amisulpride; ARI: aripiprazole; BEZOAET: benzoate sodium; CELECOX: celecoxib; CEREBR: cerebrolysin; CLOT: clotiapine; CLOZ: clozapine; CPZ: chlorpromazine; DESMOP: desmopressin; DUL: duloxetine; FLUPH: fluphenazine; FLUV: fluvoxamine; GinkBil: Ginkgo biloba; GLY: glycine; HAL: haloperidol; LAM: lamotrigine; LEV: levomepromazine; LI: lithium; MEM: memantine; MET: metformin; MINOC: minocycline; MIRT: mirtazapine; MODF: modafinil; OLA: olanzapine; ONDAST: ondansetron; OXYT: oxytocin; PALIP: paliperidone; PALMIT: palmitoylethanolamide; PHENYLPROP: phenylpropanolamine; PIMOZ: pimozide; PTX: pentoxifylline; QUE: quetiapine; RISP: risperidone; SARC: sarcosine; SER: sertraline; SERTIND: sertindole; SUL: sulpiride; TOPIR: topiramate; VORT: vortioxetine; ZIP: ziprasidone; ZOT: zotepine.

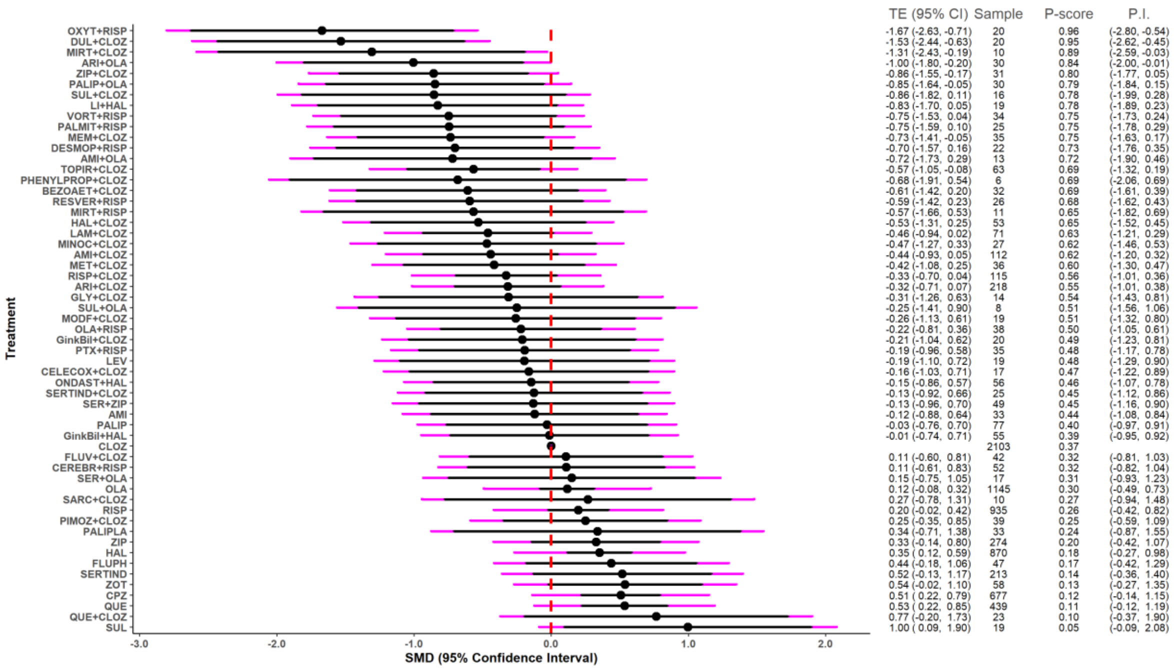

Figure S 16.3b. Forest plot with prediction intervals for the exclusion of RCTs that did not employ operationalized criteria for schizophrenia diagnosis

Reference comparator: Clozapine. AMI: amisulpride; ARI: aripiprazole; BEZOAET: benzoate sodium; CELECOX: celecoxib; CEREBR: cerebrolysin; CI: confidence intervals; CLOT: clotiapine; CLOZ: clozapine; CPZ: chlorpromazine; DESMOP: desmopressin; DUL: duloxetine; FLUPH: fluphenazine; FLUV: fluvoxamine; GinkBil: Ginkgo biloba; GLY: glycine; HAL: haloperidol; LAM: lamotrigine; LEV: levomepromazine; LI: lithium; MEM: memantine; MET: metformin; MINOC: minocycline; MIRT: mirtazapine; MODF: modafinil; OLA: olanzapine; ONDAST: ondansetron; OXYT: oxytocin; PALIP: paliperidone; PALMIT: palmitoylethanolamide; PHENYLPROP: phenylpropanolamine; PIMOZ: pimozide; PTX: pentoxifylline; QUE: quetiapine; RISP: risperidone; SARC: sarcosine; SER: sertraline; SERTIND: sertindole; SMD: standardized mean difference; SUL: sulpiride; TOPIR: topiramate; VORT: vortioxetine; ZIP: ziprasidone; ZOT: zotepine.

16.4. Exclusion of randomised control trials including intolerant patients (Figure S 16.4a; Figure S 16.4b)

Intolerance and non-response are distinct issues and mixing them may confound the results. Excluding studies where patients were classified as non-responders due to intolerance rather than lack of efficacy ensures similarity of populations and provides a clearer understanding of treatment efficacy. This approach also minimizes bias and enhances the validity of the findings by focusing on true non-response rather than adverse effects.

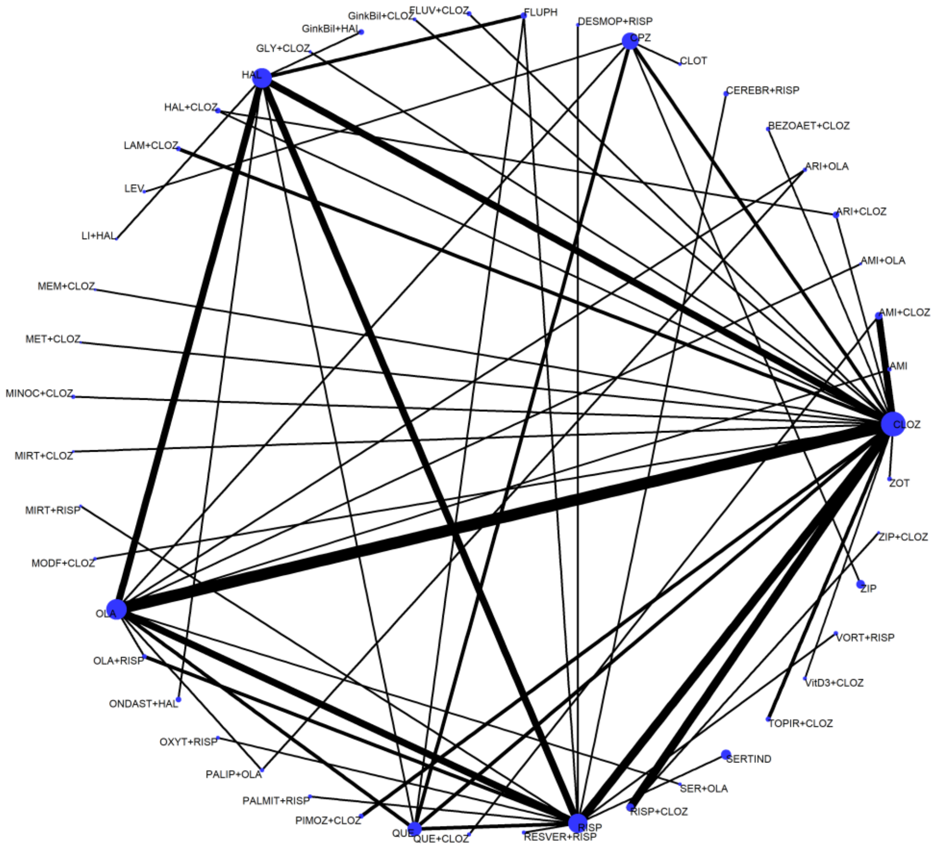

Figure S 16.4a. Network plot for the exclusion of RCTs including intolerant patients
AMI: amisulpride; ARI: aripiprazole; BEZOAET: benzoate sodium; CEREBR: cerebrolysin; CI: confidence intervals; CLOT: clotiapine; CLOZ: clozapine; CPZ: chlorpromazine; DESMOP: desmopressin; FLUPH: fluphenazine; FLUV: fluvoxamine; GinkBil: Ginkgo biloba; GLY: glycine; HAL: haloperidol; LAM: lamotrigine; LEV: levomepromazine; LI: lithium; MEM: memantine; MET: metformin; MINOC: minocycline; MIRT: mirtazapine; MODF: modafinil; OLA: olanzapine; ONDAST: ondansetron; OXYT: oxytocin; PALIP: paliperidone; PALMIT: palmitoylethanolamide; PIMOZ: pimozide; QUE: quetiapine; RESVER: resveratrol; RISP: risperidone; SER: sertraline; SERTIND: sertindole; SMD: standard mean difference; SUL: sulpiride; TOPIR: topiramate; VitD3: vitamin D3; VORT: vortioxetine; ZIP: ziprasidone; ZOT: zotepine.

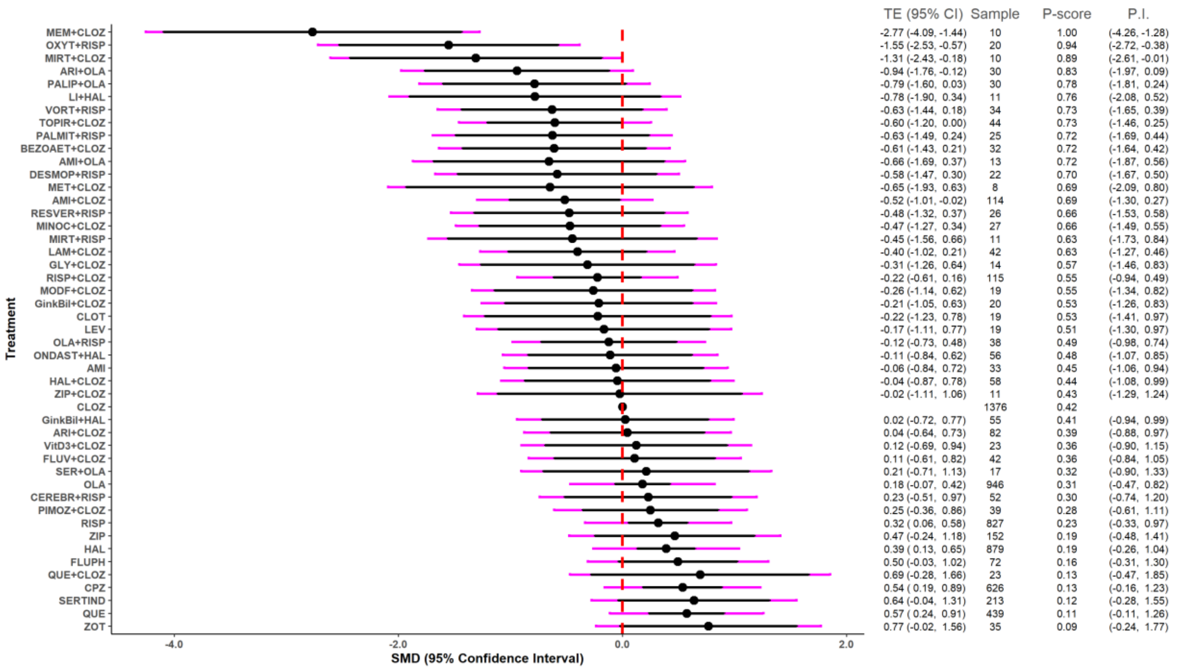

Figure S 16.4b. Forest plot with prediction intervals for the exclusion of RCTs including intolerant patients

Reference comparator: Clozapine. AMI: amisulpride; ARI: aripiprazole; BEZOAET: benzoate sodium; CEREBR: cerebrolysin; CI: confidence intervals; CLOT: clotiapine; CLOZ: clozapine; CPZ: chlorpromazine; DESMOP: desmopressin; FLUPH: fluphenazine; FLUV: fluvoxamine; GinkBil: Ginkgo biloba; GLY: glycine; HAL: haloperidol; LAM: lamotrigine; LEV: levomepromazine; LI: lithium; MEM: memantine; MET: metformin; MINOC: minocycline; MIRT: mirtazapine; MODF: modafinil; OLA: olanzapine; ONDAST: ondansetron; OXYT: oxytocin; PALIP: paliperidone; PALMIT: palmitoylethanolamide; PIMOZ: pimozide; QUE: quetiapine; RESVER: resveratrol; RISP: risperidone; SER: sertraline; SERTIND: sertindole; SMD: standardized mean difference; SUL: sulpiride; TOPIR: topiramate; VitD3: vitamin D3; VORT: vortioxetine; ZIP: ziprasidone; ZOT: zotepine.

16.5. Exclusion of randomised control trials with high risk of bias (Figure S 16.5a; Figure S 16.5b)

High-risk studies may skew the results and reduce the validity of the meta-analysis. Excluding studies rated as having a high risk of bias ensures that the findings are based on the most reliable evidence and reduces the risk of biased conclusions.

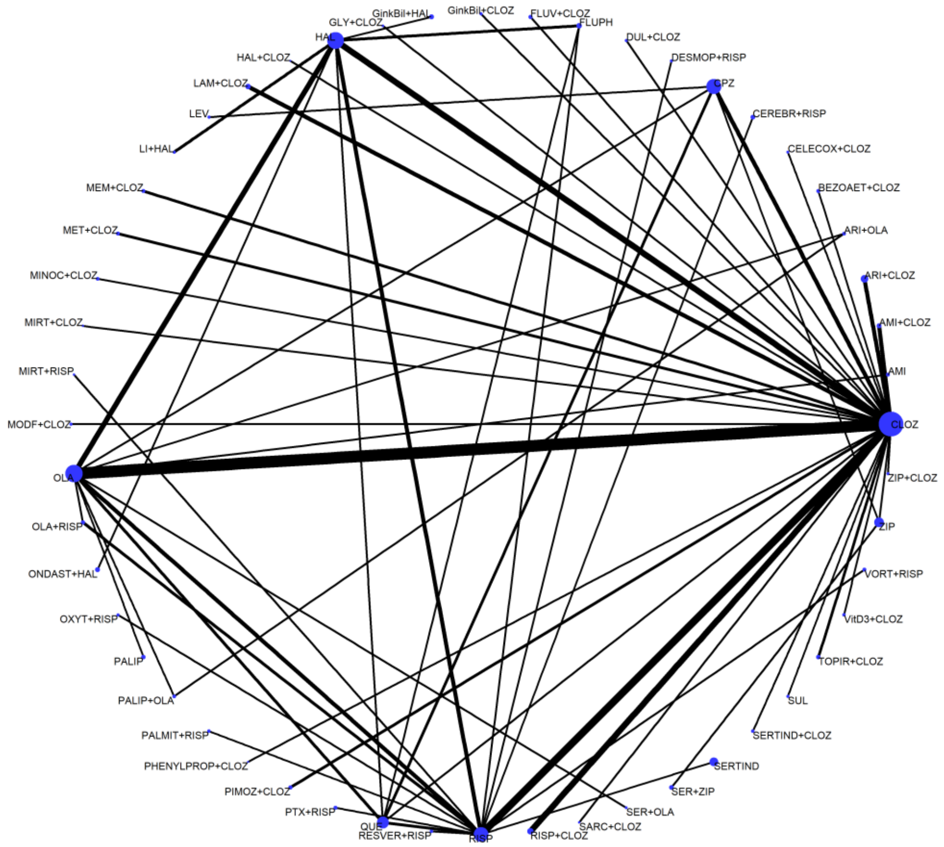

Figure S 16.5a. Network plot for the exclusion of the exclusion of RCTs with high risk of bias
AMI: amisulpride; ARI: aripiprazole; BEZOAET: benzoate sodium; CELECOX: celecoxib; CEREBR: cerebrolysin; CLOZ: clozapine; CPZ: chlorpromazine; DESMOP: desmopressin; DUL: duloxetine; FLUPH: fluphenazine; FLUV: fluvoxamine; GinkBil: Ginkgo biloba; GLY: glycine; HAL: haloperidol; LAM: lamotrigine; LEV: levomepromazine; LI: lithium; MEM: memantine; MET: metformin; MINOC: minocycline; MIRT: mirtazapine; MODF: modafinil; OLA: olanzapine; ONDAST: ondansetron; OXYT: oxytocin; PALIP: paliperidone; PALMIT: palmitoylethanolamide; PIMOZ: pimozide; PTX: pentoxifylline; QUE: quetiapine; RESVER: resveratrol; RISP: risperidone; SARC: sarcosine; SER: sertraline; SERTIND: sertindole; SUL: sulpiride; TOPIR: topiramate; VitD3: vitamin D3; VORT: vortioxetine; ZIP: ziprasidone.

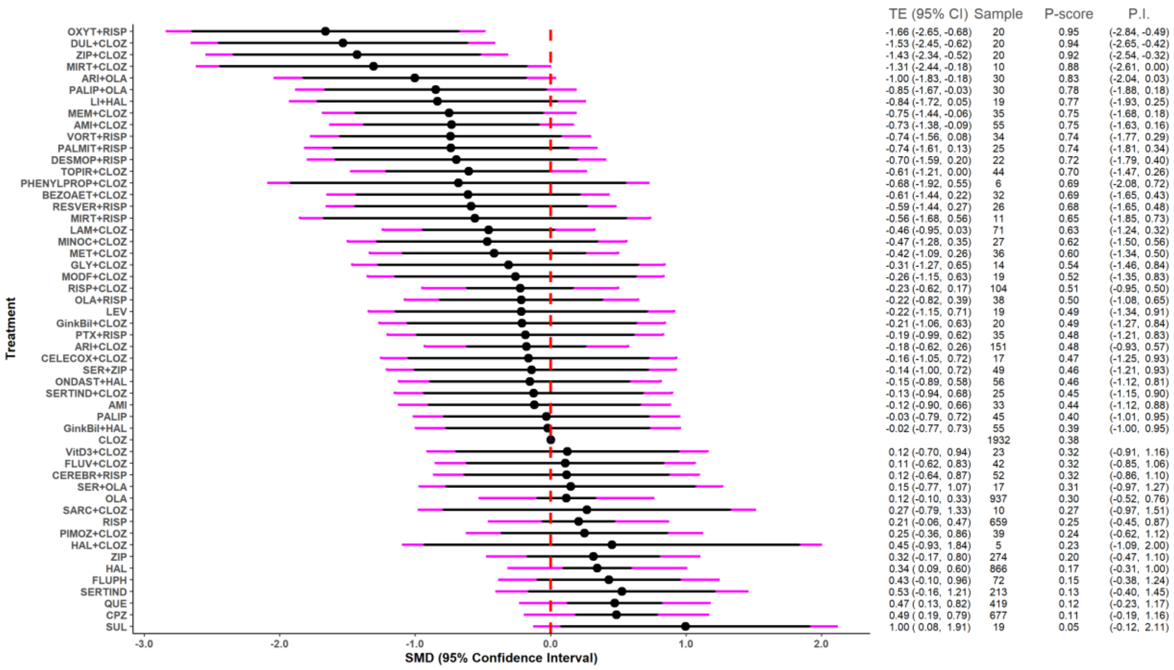

Figure S 16.5b. Forest plot with prediction intervals for the exclusion of RCTs with high risk of bias
Reference comparator: Clozapine. AMI: amisulpride; ARI: aripiprazole; BEZOAET: benzoate sodium; CELECOX: celecoxib; CEREBR: cerebrolysin; CI: confidence intervals; CLOZ: clozapine; CPZ: chlorpromazine; DESMOP: desmopressin; DUL: duloxetine; FLUPH: fluphenazine; FLUV: fluvoxamine; GinkBil: Ginkgo biloba; GLY: glycine; HAL: haloperidol; LAM: lamotrigine; LEV: levomepromazine; LI: lithium; MEM: memantine; MET: metformin; MINOC: minocycline; MIRT: mirtazapine; MODF: modafinil; OLA: olanzapine; ONDAST: ondansetron; OXYT: oxytocin; PALIP: paliperidone; PALMIT: palmitoylethanolamide; PIMOZ: pimozide; PTX: pentoxifylline; QUE: quetiapine; RESVER: resveratrol; RISP: risperidone; SARC: sarcosine; SER: sertraline; SERTIND: sertindole; SMD: standardized mean difference; SUL: sulpiride; TOPIR: topiramate; VitD3: vitamin D3; VORT: vortioxetine; ZIP: ziprasidone.

16.6. Assumption of missing data (Figure S 16.6a; Figure S 16.6b)

Assumptions about missing data can introduce bias. Excluding studies that presented only completers data helps ensure that the analysis is not skewed by incomplete or selective reporting. This approach reduces the risk of overestimating treatment effects and provides a more accurate representation of the true outcomes.

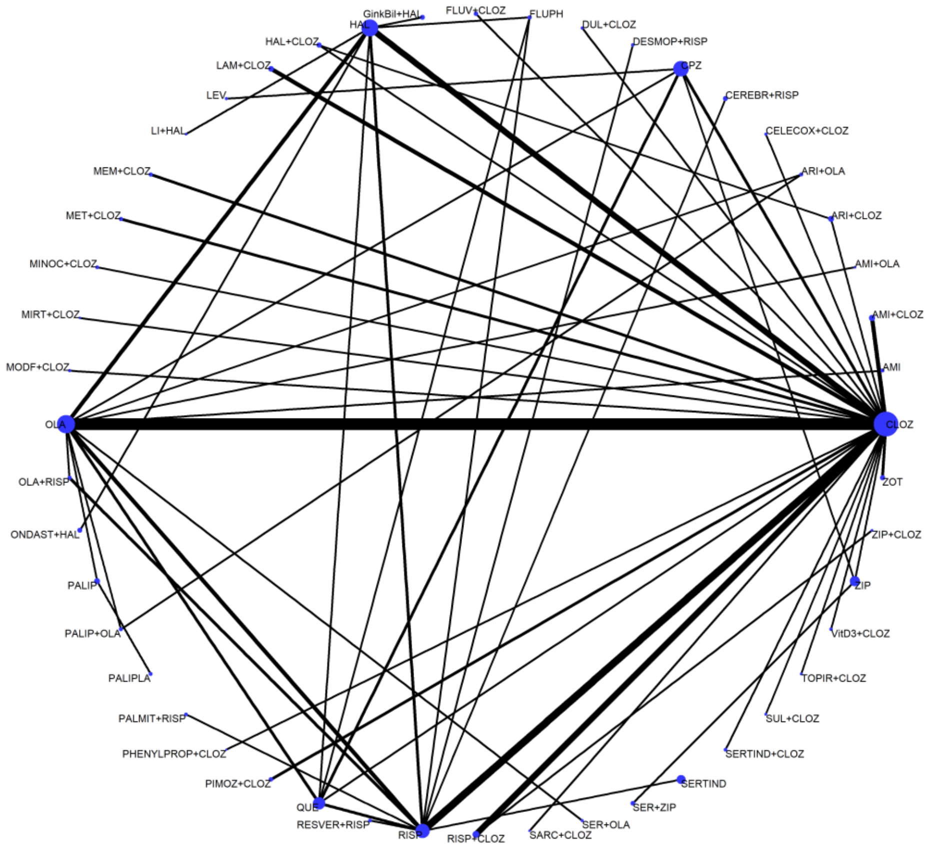

Figure S 16.6a. Network plot for the assumption of missing data
AMI: amisulpride; ARI: aripiprazole; BEZOAET: benzoate sodium; CELECOX: celecoxib; CEREBR: cerebrolysin; CLOZ: clozapine; CPZ: chlorpromazine; DESMOP: desmopressin; DUL: duloxetine; FLUPH: fluphenazine; FLUV: fluvoxamine; GinkBil: Ginkgo biloba; GLY: glycine; HAL: haloperidol; LAM: lamotrigine; LEV: levomepromazine; LI: lithium; MEM: memantine; MET: metformin; MINOC: minocycline; MIRT: mirtazapine; MODF: modafinil; OLA: olanzapine; ONDAST: ondansetron; OXYT: oxytocin; PALIP: paliperidone; PALMIT: palmitoylethanolamide; PHENYLPROP: phenylpropanolamine; PIMOZ: pimozide; QUE: quetiapine; RESVER: resveratrol; RISP: risperidone; SARC: sarcosine; SER: sertraline; SERTIND: sertindole; SUL: sulpiride; TOPIR: topiramate; VitD3: vitamin D3; VORT: vortioxetine; ZIP: ziprasidone; ZOT: zotepine.

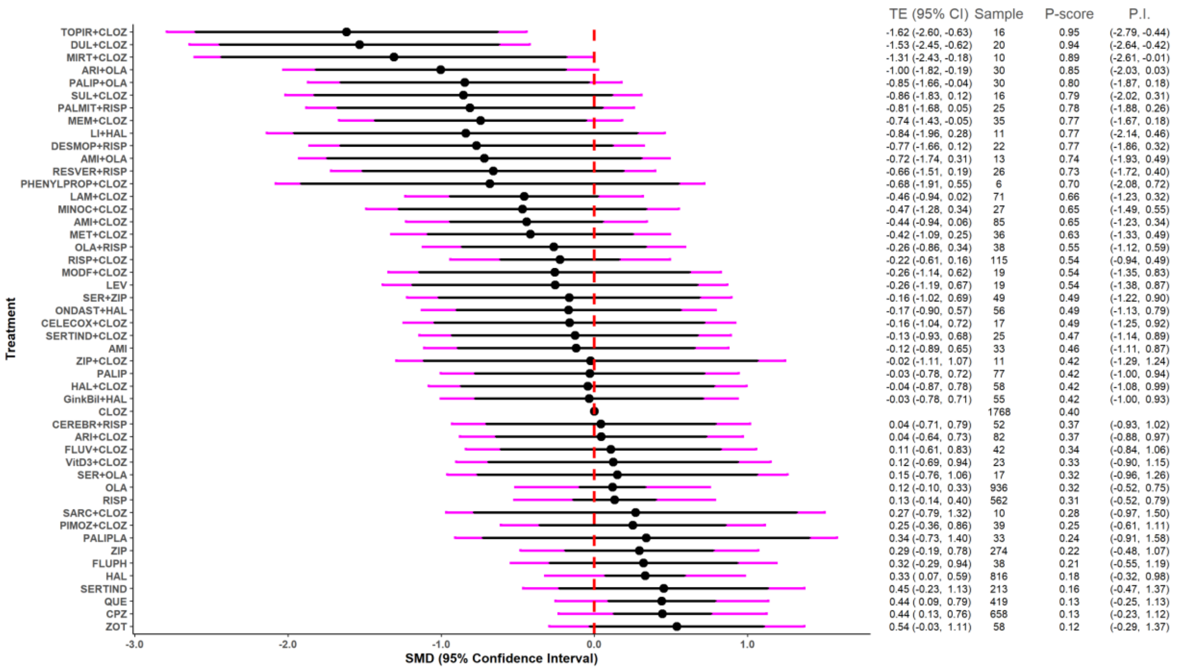

Figure S 16.6b. Forest plot with prediction intervals for the assumption of missing data

Reference comparator: Clozapine. AMI: amisulpride; ARI: aripiprazole; BEZOAET: benzoate sodium; CELECOX: celecoxib; CEREBR: cerebrolysin; CI: confidence intervals; CLOZ: clozapine; CPZ: chlorpromazine; DESMOP: desmopressin; DUL: duloxetine; FLUPH: fluphenazine; FLUV: fluvoxamine; GinkBil: Ginkgo biloba; GLY: glycine; HAL: haloperidol; LAM: lamotrigine; LEV: levomepromazine; LI: lithium; MEM: memantine; MET: metformin; MINOC: minocycline; MIRT: mirtazapine; MODF: modafinil; OLA: olanzapine; ONDAST: ondansetron; OXYT: oxytocin; PALIP: paliperidone; PALMIT: palmitoylethanolamide; PHENYLPROP: phenylpropanolamine; PIMOZ: pimozide; QUE: quetiapine; RESVER: resveratrol; RISP: risperidone; SARC: sarcosine; SER: sertraline; SERTIND: sertindole; SMD: standardized mean difference; SUL: sulpiride; TOPIR: topiramate; VitD3: vitamin D3; VORT: vortioxetine; ZIP: ziprasidone; ZOT: zotepine.

16.7. Country: Trials from developed countries only (Figure S 16.7a; Figure S 16.7b)

Studies conducted in less developed countries might exaggerate treatment effects and often lack methodological rigor, potentially compromising the quality of the findings. Excluding such studies can help ensure the validity and reliability of the results by minimizing the influence of biased or low-quality evidence.

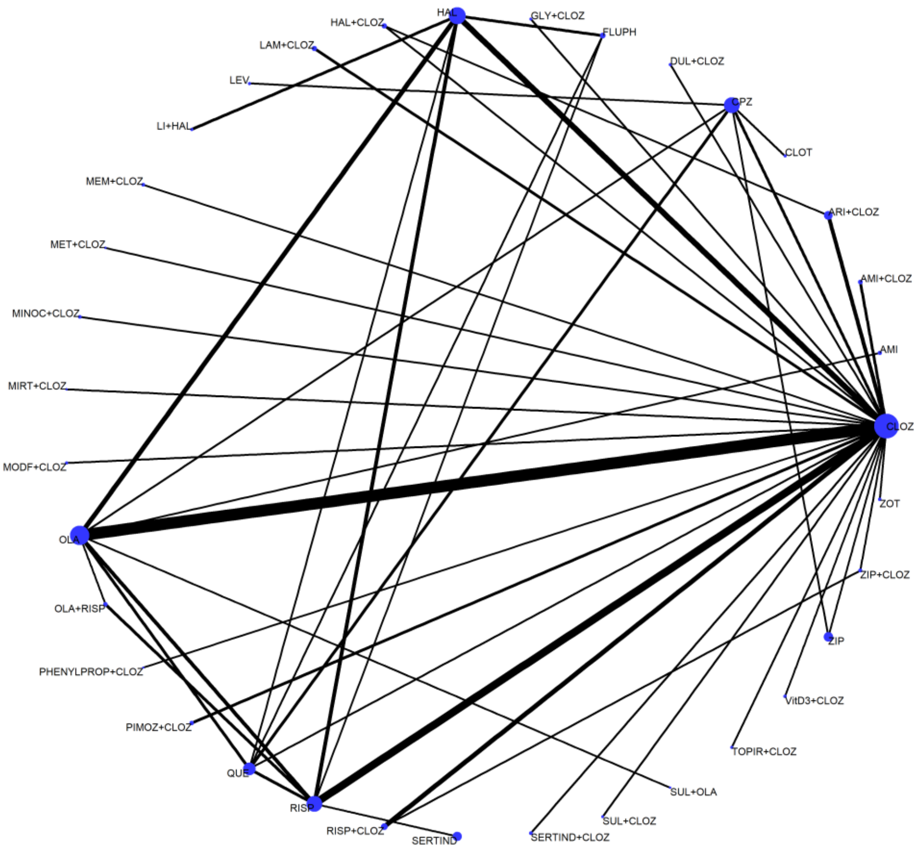

Figure S 16.7a. Network plot for country: Trials from developed countries only
AMI: amisulpride; ARI: aripiprazole; CLOT: clotiapine; CLOZ: clozapine; CPZ: chlorpromazine; DUL: duloxetine; FLUPH: fluphenazine; GLY: glycine; HAL: haloperidol; LAM: lamotrigine; LEV: levomepromazine; LI: lithium; MEM: memantine; MET: metformin; MINOC: minocycline; MIRT: mirtazapine; MODF: modafinil; OLA: olanzapine; PHENYLPROP: phenylpropanolamine; PIMOZ: pimozide; QUE: quetiapine; RISP: risperidone; SERTIND: sertindole; SUL: sulpiride; TOPIR: topiramate; VitD3: vitamin D3; VORT: vortioxetine; ZIP: ziprasidone; ZOT: zotepine.

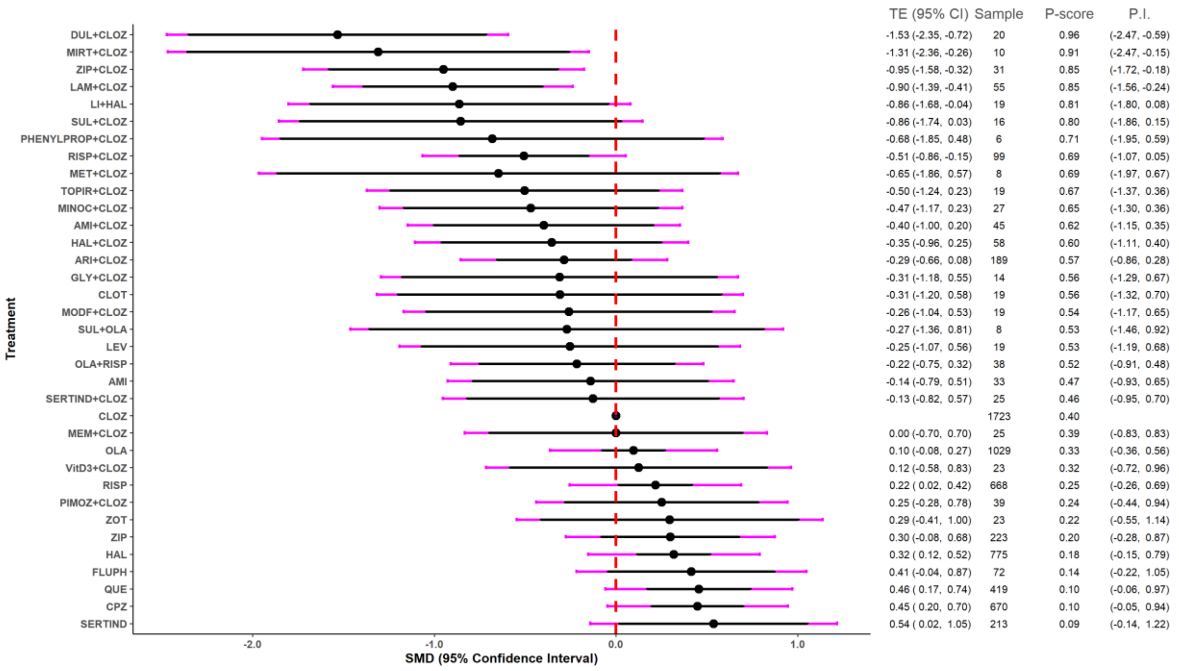

Figure S 16.7b. Forest plot with prediction intervals for country: Trials from developed countries only
Reference comparator: Clozapine. AMI: amisulpride; ARI: aripiprazole; CI: confidence intervals; CLOT: clotiapine; CLOZ: clozapine; CPZ: chlorpromazine; DUL: duloxetine; FLUPH: fluphenazine; GLY: glycine; HAL: haloperidol; LAM: lamotrigine; LEV: levomepromazine; LI: lithium; MEM: memantine; MET: metformin; MINOC: minocycline; MIRT: mirtazapine; MODF: modafinil; OLA: olanzapine; PHENYLPROP: phenylpropanolamine; PIMOZ: pimozide; QUE: quetiapine; RISP: risperidone; SERTIND: sertindole; SMD: standardized mean difference; SUL: sulpiride; TOPIR: topiramate; VitD3: vitamin D3; VORT: vortioxetine; ZIP: ziprasidone; ZOT: zotepine.

16.8. Common-effect model (Figure S 16.8)

Repeating the analysis using a common-effect (fixed-effect) model instead of a random-effects model tests whether the choice of statistical model affects the results. Common-effect models assume no heterogeneity, while random-effects models account for it.

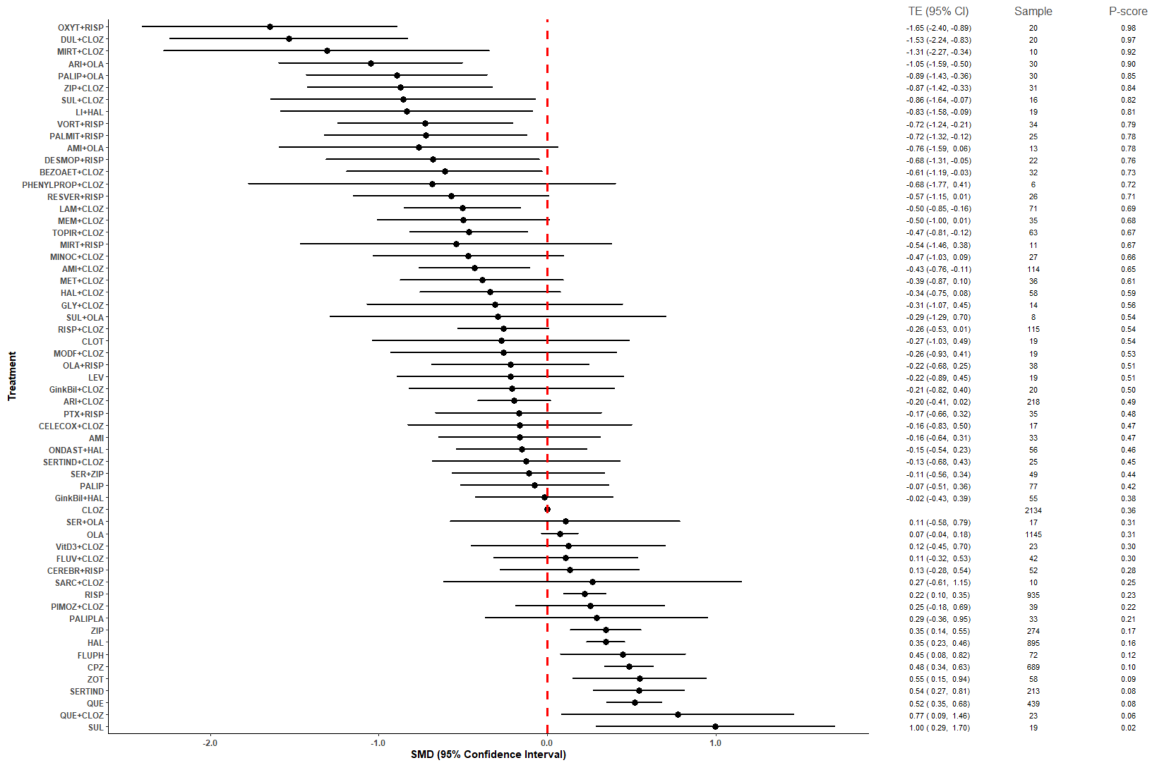

Figure S 16.8. Forest plot with prediction intervals using a common-effect model
Reference comparator: Clozapine. AMI: amisulpride; ARI: aripiprazole; BEZOAET: benzoate sodium; CELECOX: celecoxib; CEREBR: cerebrolysin; CI: confidence intervals; CLOZ: clozapine; CPZ: chlorpromazine; DESMOP: desmopressin; DUL: duloxetine; FLUPH: fluphenazine; FLUV: fluvoxamine; GinkBil: Ginkgo biloba; GLY: glycine; HAL: haloperidol; LAM: lamotrigine; LEV: levomepromazine; LI: lithium; MEM: memantine; MET: metformin; MINOC: minocycline; MIRT: mirtazapine; MODF: modafinil; OLA: olanzapine; ONDAST: ondansetron; OXYT: oxytocin; PALIP: paliperidone; PALMIT: palmitoylethanolamide; PHENYLPROP: phenylpropanolamine; PIMOZ: pimozide; PTX: pentoxifylline; QUE: quetiapine; RESVER: resveratrol; RISP: risperidone; SARC: sarcosine; SER: sertraline; SERTIND: sertindole; SMD: standardized mean difference; SUL: sulpiride; TOPIR: topiramate; VitD3: vitamin D3; VORT: vortioxetine; ZIP: ziprasidone; ZOT: zotepine.

16.9. Extreme sensitivity analysis excluding the following studies: Open-label, intolerant patients, and low and medium stringency of resistance criteria (Figure S 16.9a; Figure S 16.9b)

Studies with open-label designs (not blinded), intolerant patients, and low or medium stringency of resistance criteria were excluded. This stringent approach was taken to test whether the results remain consistent even after removing studies with potential methodological weaknesses or variability in patient populations. By doing so, the analysis ensures the robustness and reliability of the findings, minimizing the influence of lower-quality or heterogeneous studies.

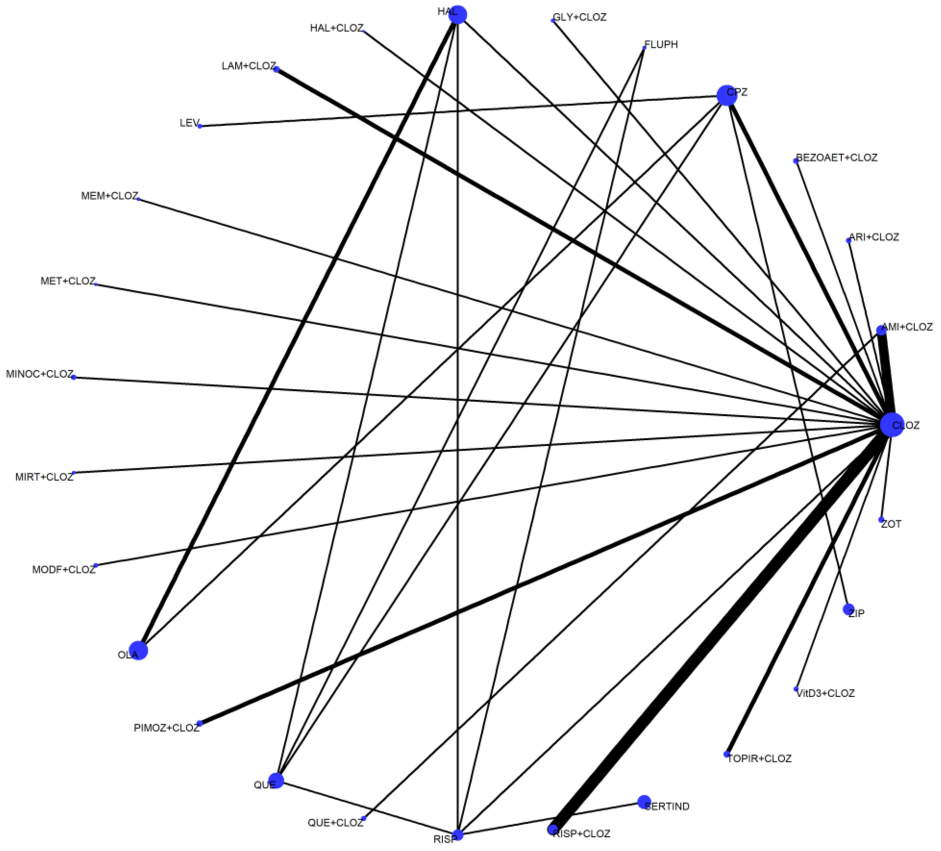

Figure S 16.9a. Network plot for extreme sensitivity analysis excluding (1) open-label studies, (2) studies with intolerant patients, (3) studies with low and medium stringency of resistance criteria

AMI: amisulpride; ARI: aripiprazole; BEZOAET: benzoate sodium; CLOZ: clozapine; CPZ: chlorpromazine;: fluphenazine; FLUV: fluvoxamine; GLY: glycine; HAL: haloperidol; LAM: lamotrigine; LEV: levomepromazine; MEM: memantine; MET: metformin; MINOC: minocycline; MIRT: mirtazapine; MODF: modafinil; OLA: olanzapine; PIMOZ: pimozide; QUE: quetiapine; RISP: risperidone; SERTIND: sertindole; TOPIR: topiramate; VitD3: vitamin D3; VORT: vortioxetine; ZIP: ziprasidone; ZOT: zotepine.

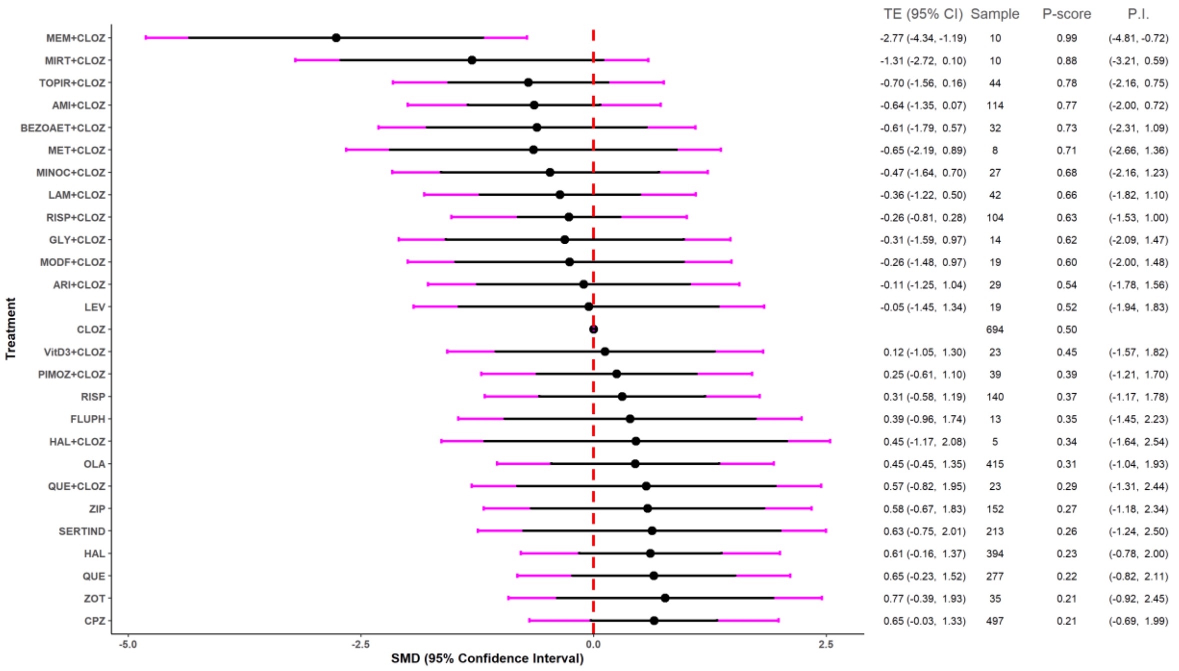

Figure S 16.9b. Forest plot with prediction intervals extreme sensitivity analysis excluding (1) open-label studies, (2) studies with intolerant patients, (3) studies with low and medium stringency of resistance criteria

Reference comparator: Clozapine. AMI: amisulpride; ARI: aripiprazole; BEZOAET: benzoate sodium; CI: confidence intervals; CLOZ: clozapine; CPZ: chlorpromazine;: fluphenazine; FLUV: fluvoxamine; GLY: glycine; HAL: haloperidol; LAM: lamotrigine; LEV: levomepromazine; MEM: memantine; MET: metformin; MINOC: minocycline; MIRT: mirtazapine; MODF: modafinil; OLA: olanzapine; PIMOZ: pimozide; QUE: quetiapine; RISP: risperidone; SERTIND: sertindole; SMD: standardized mean difference; TOPIR: topiramate; VitD3: vitamin D3; VORT: vortioxetine; ZIP: ziprasidone; ZOT: zotepine**.**

Appendix 17. Pairwise meta-analysis of drug groups

17.1. Combination of clozapine with another antipsychotic vs clozapine monotherapy

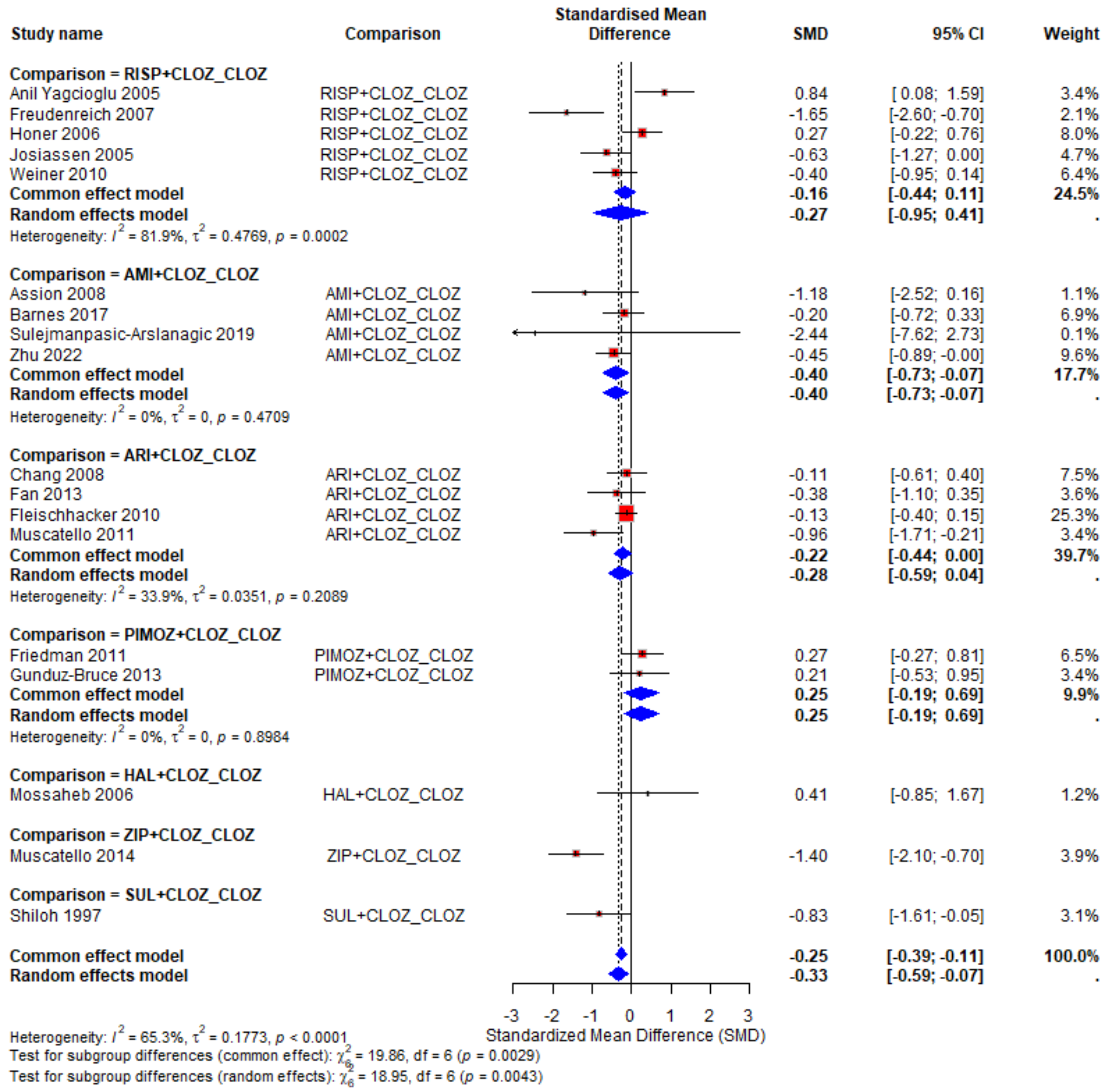

Figure S 17.1. Combination of clozapine with another antipsychotic vs clozapine monotherapy
AMI: amisulpride; ARI: aripiprazole; CI: confidence intervals; CLOZ: clozapine; HAL: haloperidol; PIMOZ: pimozide; RISP: risperidone; SUL: sulpiride; ZIP: ziprasidone.

17.2. Combination of olanzapine with another antipsychotic versus antipsychotic monotherapy

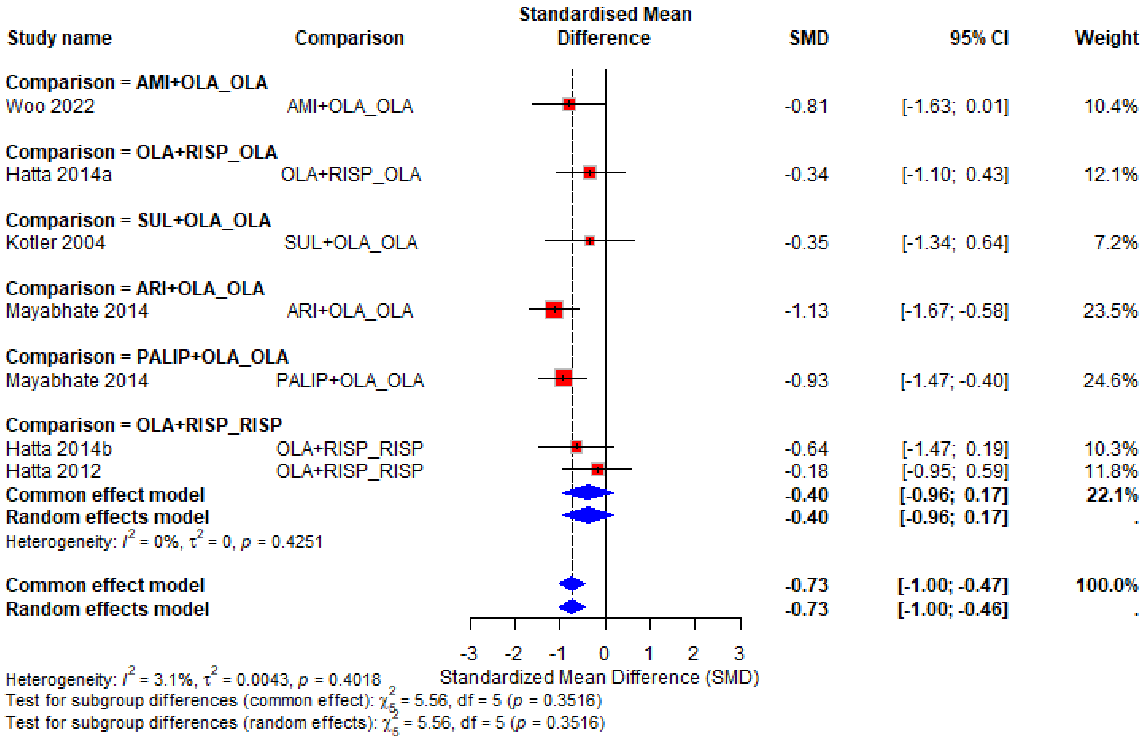

Figure S 17.2. Combination of olanzapine with another antipsychotic versus antipsychotic monotherapy
AMI: amisulpride; ARI: aripiprazole; CI: confidence intervals; PALIP: paliperidone; OLA: olanzapine; RISP: risperidone; SUL: sulpiride.

17.3. Antipsychotics augmentation with antiepileptics versus antipsychotic monotherapy

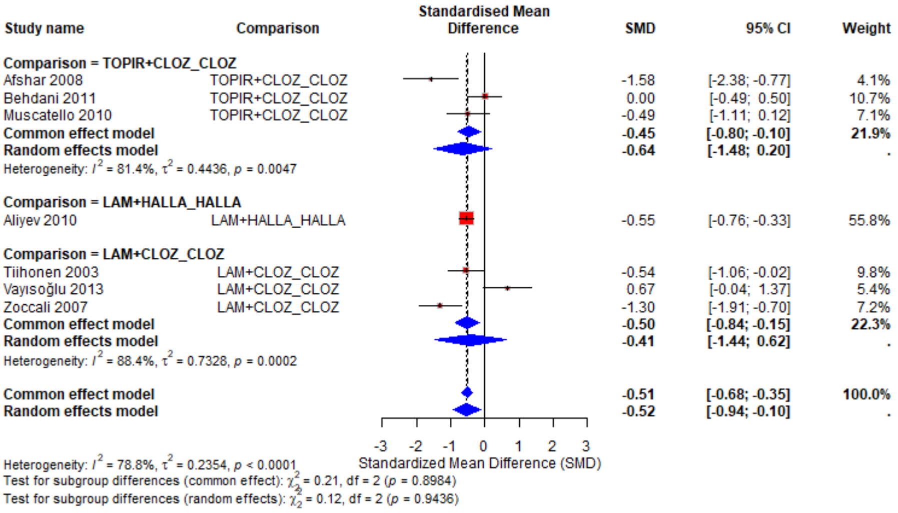

Figure S 17.3. Antipsychotics augmentation with antiepileptics vs antipsychotics monotherapy
CI: confidence intervals; CLOZ: clozapine; HALLA: haloperidol long-acting; LAM: lamotrigine; TOPIR: topiramate.

Appendix 18. Assessment of publication bias: Contour-enhanced funnel plot

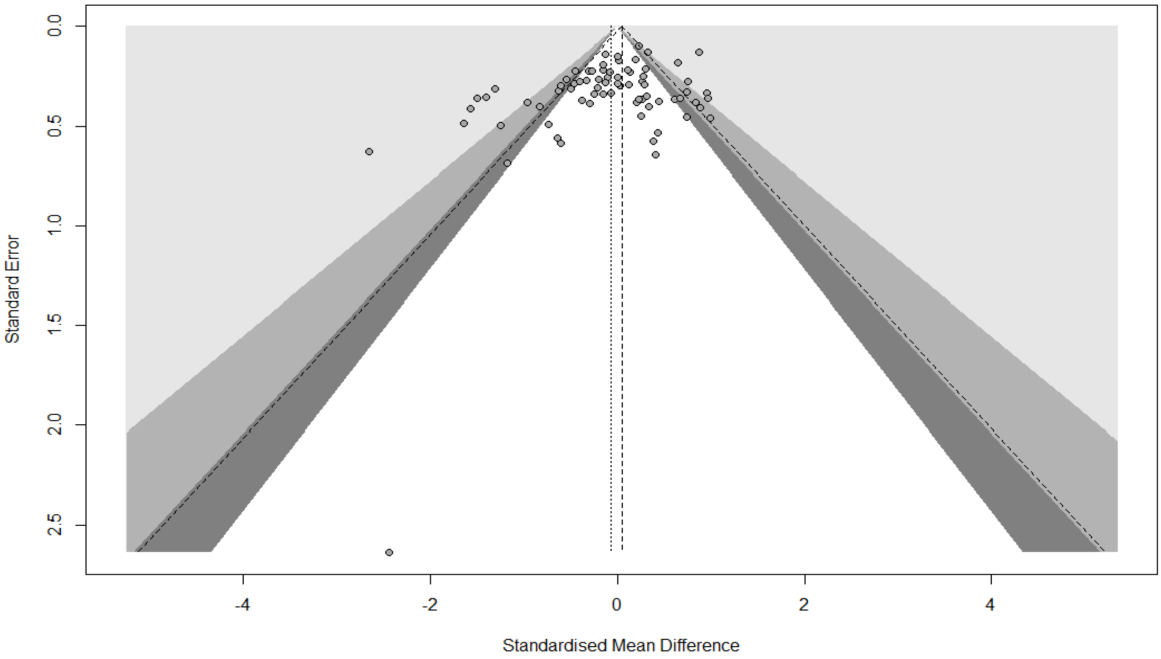

Egger's test result: t = -3.10, df = 72, p-value = 0.0027

Bias estimate: -1.5999 (SE = 0.5157)

Figure S 18. Contour-enhanced funnel plot for all antipsychotics compared to clozapine
Change in overall symptoms for all antipsychotics compared to clozapine. The circles indicate the effect sizes of individual studies, measured as standardized mean differences.

Appendix 19. References

1. Huhn M, Nikolakopoulou A, Schneider-Thoma J, et al. Comparative efficacy and tolerability of 32 oral antipsychotics for the acute treatment of adults with multi-episode schizophrenia: A systematic review and network meta-analysis. *Lancet Lond Engl*. 2019;394(10202):939-951. doi:10.1016/S0140-6736(19)31135-3

2. Samara MT, Nikolakopoulou A, Salanti G, Leucht S. How Many Patients With Schizophrenia Do Not Respond to Antipsychotic Drugs in the Short Term? An Analysis Based on Individual Patient Data From Randomized Controlled Trials. *Schizophr Bull*. 2019;45(3):639-646. doi:10.1093/schbul/sby095

3. Demjaha A, Lappin JM, Stahl D, et al. Antipsychotic treatment resistance in first-episode psychosis: prevalence, subtypes and predictors. *Psychol Med*. 2017;47(11):1981-1989. doi:10.1017/S0033291717000435

4. Lally J, Ajnakina O, Di Forti M, et al. Two distinct patterns of treatment resistance: clinical predictors of treatment resistance in first-episode schizophrenia spectrum psychoses. *Psychol Med*. 2016;46(15):3231-3240. doi:10.1017/S0033291716002014

5. Kane JM, Agid O, Baldwin ML, et al. Clinical Guidance on the Identification and Management of Treatment-Resistant Schizophrenia. *J Clin Psychiatry*. 2019;80(2):18com12123. doi:10.4088/JCP.18com12123

6. Diniz E, Fonseca L, Rocha D, et al. Treatment resistance in schizophrenia: a meta-analysis of prevalence and correlates. *Braz J Psychiatry*. 2023;45(5):448-458. doi:10.47626/1516-4446-2023-3126

7. Samara MT, Dold M, Gianatsi M, et al. Efficacy, acceptability, and tolerability of antipsychotics in treatment-resistant schizophrenia: A network meta-analysis. *JAMA Psychiatry*. 2016;73(3):199-210. doi:10.1001/jamapsychiatry.2015.2955

8. Warnez S, Alessi-Severini S. Clozapine: a review of clinical practice guidelines and prescribing trends. *BMC Psychiatry*. 2014;14(1):102. doi:10.1186/1471-244X-14-102

9. Lagreula J, De Timary P, Elens L, Dalleur O. Antipsychotic polypharmacy and clozapine prescribing patterns: evolution and correlates before and after a psychiatric hospitalisation. *Ther Adv Psychopharmacol*. 2022;12:20451253221112587. doi:10.1177/20451253221112587

10. Tiihonen J, Taipale H, Mehtälä J, Vattulainen P, Correll CU, Tanskanen A. Association of Antipsychotic Polypharmacy vs Monotherapy With Psychiatric Rehospitalization Among Adults With Schizophrenia. *JAMA Psychiatry*. 2019;76(5):499-507. doi:10.1001/jamapsychiatry.2018.4320

11. Dong S, Schneider-Thoma J, Bighelli I, et al. A network meta-analysis of efficacy, acceptability, and tolerability of antipsychotics in treatment-resistant schizophrenia. *Eur Arch Psychiatry Clin Neurosci*. 2024;274(4):917-928. doi:10.1007/s00406-023-01654-2

12. Jones PB, Barnes TRE, Davies L, et al. Randomized Controlled Trial of the Effect on Quality of Life of Second- vs First-Generation Antipsychotic Drugs in Schizophrenia: Cost Utility of the Latest Antipsychotic Drugs in Schizophrenia Study (CUtLASS 1). *Arch Gen Psychiatry*. 2006;63(10):1079-1087. doi:10.1001/archpsyc.63.10.1079

13. Lieberman JA, Stroup TS, McEvoy JP, et al. Effectiveness of Antipsychotic Drugs in Patients with Chronic Schizophrenia. *N Engl J Med*. 2005;353(12):1209-1223. doi:10.1056/NEJMoa051688

14. Galling B, Roldán A, Hagi K, et al. Antipsychotic augmentation vs. monotherapy in schizophrenia: systematic review, meta-analysis and meta-regression analysis. *World Psychiatry*. 2017;16(1):77-89. doi:10.1002/wps.20387

15. Yeh TC, Correll CU, Yang FC, et al. Pharmacological and nonpharmacological augmentation treatments for clozapine-resistant schizophrenia: A systematic review and network meta-analysis with normalized entropy assessment. *Asian J Psychiatry*. 2023;79:103375. doi:10.1016/j.ajp.2022.103375

16. Zheng M, Liu R, Ni Z, Yu Z. Efficiency, effectiveness and treatment stability of clear aligners: A systematic review and meta-analysis. *Orthod Craniofac Res*. 2017;20(3):127-133. doi:10.1111/ocr.12177

17. Howes OD, McCutcheon R, Agid O, et al. Treatment-Resistant Schizophrenia: Treatment Response and Resistance in Psychosis (TRRIP) Working Group Consensus Guidelines on Diagnosis and Terminology. *Am J Psychiatry*. 2017;174(3):216-229. doi:10.1176/appi.ajp.2016.16050503

18. Kane J. Clozapine for the Treatment-Resistant Schizophrenic: A Double-blind Comparison With Chlorpromazine. *Arch Gen Psychiatry*. 1988;45(9):789. doi:10.1001/archpsyc.1988.01800330013001

19. McAdam MK, Baldessarini RJ, Murphy AL, Gardner DM. Second International Consensus Study of Antipsychotic Dosing (ICSAD-2). *J Psychopharmacol Oxf Engl*. 2023;37(10):982-991. doi:10.1177/02698811231205688

20. Ahlfors UG, Dencker SJ, Gravem A, Remvig J. Clopenthixol decanoate and perhenazine enanthate in schizophrenic patients: A double-blind Nordic multicentre trial. *Acta Psychiatr Scand*. 1980;61(S279):77-91. doi:10.1111/j.1600-0447.1980.tb07085.x

21. Altamura AC, Velonà I, Curreli R, Mundo E, Bravi D. Is olanzapine better than haloperidol in resistant schizophrenia? A double-blind study in partial responders. *Int J Psychiatry Clin Pract*. 2002;6(2):107-111. doi:10.1080/136515002753724117

22. AstraZeneca. *A Multicenter, Double-Blind, Randomized, Comparison of Quetiapine (SEROQUEL) and Chlorpromazine in the Treatment of Subjects with Treatment-Resistant Schizophrenia (5077IL/0031)*.; 2005. https://www.astrazenecaclinicaltrials.com/study/5077IL%2F0031/

23. AstraZeneca. *A Multicentre, Double-Blind, Randomised Trial to Compare the Effects of SEROQUEL and Chlorpromazine in Patients with Treatment Resistant Schizophrenia (5077IL/0054 [TRESS])*.; 2000. https://www.astrazenecaclinicaltrials.com/study/5077IL%2F0054/

24. Azorin JM, Spiegel R, Remington G, et al. A double-blind comparative study of clozapine and risperidone in the management of severe chronic schizophrenia. *Am J Psychiatry*. 2001;158(8):1305-1313. doi:10.1176/appi.ajp.158.8.1305

25. Bitter I, Dossenbach MRK, Brook S, et al. Olanzapine versus clozapine in treatment-resistant or treatment-intolerant schizophrenia. *Prog Neuropsychopharmacol Biol Psychiatry*. 2004;28(1):173-180. doi:10.1016/j.pnpbp.2003.09.033

26. Bondolfi G, Dufour H, Patris M, et al. Risperidone versus clozapine in treatment-resistant chronic schizophrenia: a randomized double-blind study. The Risperidone Study Group. *Am J Psychiatry*. 1998;155(4):499-504. doi:10.1176/ajp.155.4.499

27. Bozzatello P, Bellino S, Mancini I, Sandei L, Zanalda E, Rocca P. Effects on Satisfaction and Service Engagement of Paliperidone Palmitate Compared with Oral Paliperidone in Patients with Schizophrenia: An Open Label Randomized Controlled Trial. *Clin Drug Investig*. 2019;39(2):169-178. doi:10.1007/s40261-018-0734-1

28. Breier AF, Malhotra AK, Su TP, et al. Clozapine and risperidone in chronic schizophrenia: effects on symptoms, parkinsonian side effects, and neuroendocrine response. *Am J Psychiatry*. 1999;156(2):294-298. doi:10.1176/ajp.156.2.294

29. Breier A, Hamilton SH. Comparative efficacy of olanzapine and haloperidol for patients with treatment-resistant schizophrenia. *Biol Psychiatry*. 1999;45(4):403-411. doi:10.1016/s0006-3223(98)00291-1

30. Browne FW, Cooper SJ, Wilson R, King DJ. Serum haloperidol levels and clinical response in chronic, treatment-resistant schizophrenic patients. *J Psychopharmacol Oxf Engl*. 1988;2(2):94-103. doi:10.1177/026988118800200204

31. Feighner JP, Robins E, Guze SB, Woodruff RA, Winokur G, Munoz R. Diagnostic Criteria for Use in Psychiatric Research. *Arch Gen Psychiatry*. 1972;26(1):57. doi:10.1001/archpsyc.1972.01750190059011

32. Buchanan RW, Breier A, Kirkpatrick B, Ball P, Carpenter WT. Positive and negative symptom response to clozapine in schizophrenic patients with and without the deficit syndrome. *Am J Psychiatry*. 1998;155(6):751-760. doi:10.1176/ajp.155.6.751

33. Buchanan RW, Ball MP, Weiner E, et al. Olanzapine treatment of residual positive and negative symptoms. *Am J Psychiatry*. 2005;162(1):124-129. doi:10.1176/appi.ajp.162.1.124

34. Chen JJ, Chan HY, Chen CH, Gau SSF, Hwu HG. Risperidone and olanzapine versus another first generation antipsychotic in patients with schizophrenia inadequately responsive to first generation antipsychotics. *Pharmacopsychiatry*. 2012;45(2):64-71. doi:10.1055/s-0031-1291293

35. Chowdhury AN, Mukherjee A, Ghosh K, Chowdhury S, Das Sen K. Horizon of a new hope: Recovery of schizophrenia in India. *Int Med J*. 1999;6(3):181-185.

36. Claus A, Bollen J, De Cuyper H, et al. Risperidone versus haloperidol in the treatment of chronic schizophrenic inpatients: a multicentre double-blind comparative study. *Acta Psychiatr Scand*. 1992;85(4):295-305. doi:10.1111/j.1600-0447.1992.tb01473.x

37. Conley RR, Tamminga CA, Bartko JJ, et al. Olanzapine compared with chlorpromazine in treatment-resistant schizophrenia. *Am J Psychiatry*. 1998;155(7):914-920. doi:10.1176/ajp.155.7.914

38. Conley RR, Kelly DL, Richardson CM, Tamminga CA, Carpenter WT. The efficacy of high-dose olanzapine versus clozapine in treatment-resistant schizophrenia: a double-blind crossover study. *J Clin Psychopharmacol*. 2003;23(6):668-671. doi:10.1097/01.jcp.0000096246.29231.73

39. Conley RR, Kelly DL, Nelson MW, et al. Risperidone, quetiapine, and fluphenazine in the treatment of patients with therapy-refractory schizophrenia. *Clin Neuropharmacol*. 2005;28(4):163-168. doi:10.1097/01.wnf.0000172993.89879.0f

40. Daniel DG, Goldberg TE, Weinberger DR, et al. Different side effect profiles of risperidone and clozapine in 20 outpatients with schizophrenia or schizoaffective disorder: a pilot study. *Am J Psychiatry*. 1996;153(3):417-419. doi:10.1176/ajp.153.3.417

41. Dean EF, Buker S. Schizophrenia treated with and without chlorpromazine. *Rocky Mt Med J*. 1958;55(4):47-50.

42. Emsley RA, Raniwalla J, Bailey PJ, Jones AM. A comparison of the effects of quetiapine ('seroquel') and haloperidol in schizophrenic patients with a history of and a demonstrated, partial response to conventional antipsychotic treatment. PRIZE Study Group. *Int Clin Psychopharmacol*. 2000;15(3):121-131. doi:10.1097/00004850-200015030-00001

43. Geller V, Gorzaltsan I, Shleifer T, Belmaker RH, Bersudsky Y. Clotiapine compared with chlorpromazine in chronic schizophrenia. *Schizophr Res*. 2005;80(2-3):343-347. doi:10.1016/j.schres.2005.07.007

44. Hall WB, Vestre ND, Schiele BC, Zimmermann R. A controlled comparison of haloperidol and fluphenazine in chronic treatment-resistant schizophrenics. *Dis Nerv Syst*. 1968;29(6):405-408.

45. Herken H, Kaya N, Beşiroğlu L, Derman H, Özkan I. Kronik şizofreni hastalarında klozapin ve sulpiridin etkinliğinin karşılaştırılması. *Bull Clin Psychopharmacol*. 1999;9(3):148-151.

46. Heres S, Cordes J, Feyerabend S, et al. Changing the Antipsychotic in Early Nonimprovers to Amisulpride or Olanzapine: Randomized, Double-Blind Trial in Patients With Schizophrenia. *Schizophr Bull*. 2022;48(6):1273-1283. doi:10.1093/schbul/sbac068

47. Hong CJ, Chen JY, Chiu HJ, Sim CB. A double-blind comparative study of clozapine versus chlorpromazine on Chinese patients with treatment-refractory schizophrenia. *Int Clin Psychopharmacol*. 1997;12(3):123-130. doi:10.1097/00004850-199705000-00001

48. Honigfeld G, Patin J, Singer J. Clozapine: Antipsychotic activity in treatment-resistant schizophrenics. *Adv Ther*. 1984;1(2):77-97.

49. Howard JS. Haloperidol for chronically hospitalized psychotics: A double-blind comparison with thiothixene and placebo; a follow-up open evaluation. *Dis Nerv Syst*. 1974;35(10):458-463.

50. Kahn RS, Winter van Rossum I, Leucht S, et al. Amisulpride and olanzapine followed by open-label treatment with clozapine in first-episode schizophrenia and schizophreniform disorder (OPTiMiSE): a three-phase switching study. *Lancet Psychiatry*. 2018;5(10):797-807. doi:10.1016/S2215-0366(18)30252-9

51. Kane JM, Honigfeld G, Singer J, Meltzer H. Clozapine for the treatment-resistant schizophrenic. A double-blind comparison with chlorpromazine. *Arch Gen Psychiatry*. 1988;45(9):789-796. doi:10.1001/archpsyc.1988.01800330013001

52. Kane JM, Marder SR, Schooler NR, et al. Clozapine and haloperidol in moderately refractory schizophrenia: A 6-month randomized and double-blind comparison. *Arch Gen Psychiatry*. 2001;58(10):965-972. doi:10.1001/archpsyc.58.10.965

53. Kane JM, Khanna S, Rajadhyaksha S, Giller E. Efficacy and tolerability of ziprasidone in patients with treatment-resistant schizophrenia. *Int Clin Psychopharmacol*. 2006;21(1):21-28. doi:10.1097/01.yic.0000182114.65134.81

54. Kane JM, Meltzer HY, Carson WH, et al. Aripiprazole for treatment-resistant schizophrenia: results of a multicenter, randomized, double-blind, comparison study versus perphenazine. *J Clin Psychiatry*. 2007;68(2):213-223.

55. Kane JM, Potkin SG, Daniel DG, Buckley PF. A double-blind, randomized study comparing the efficacy and safety of sertindole and risperidone in patients with treatment-resistant schizophrenia. *J Clin Psychiatry*. 2011;72(2):194-204. doi:10.4088/JCP.07m03733yel

56. Kinon BJ, Kane JM, Johns C, et al. Treatment of neuroleptic-resistant schizophrenic relapse. *Psychopharmacol Bull*. 1993;29(2):309-314.

57. Kinon BJ, Chen L, Ascher-Svanum H, et al. Early response to antipsychotic drug therapy as a clinical marker of subsequent response in the treatment of schizophrenia. *Neuropsychopharmacol Off Publ Am Coll Neuropsychopharmacol*. 2010;35(2):581-590. doi:10.1038/npp.2009.164

58. Kluge M, Schuld A, Himmerich H, et al. Clozapine and Olanzapine Are Associated With Food Craving and Binge Eating: Results From A Randomized Double-Blind Study. *J Clin Psychopharmacol*. 2007;27(6):662-666. doi:10.1097/jcp.0b013e31815a8872

59. Kumar M, Chavan BS, Sidana A, Das S. Efficacy and tolerability of clozapine versus quetiapine in treatment-resistant schizophrenia. *Indian J Psychol Med*. 2017;39(6):770-776. doi:10.4103/IJPSYM.IJPSYM_111_17

60. Kumra S, Frazier JA, Jacobsen LK, et al. Childhood-onset schizophrenia. A double-blind clozapine-haloperidol comparison. *Arch Gen Psychiatry*. 1996;53(12):1090-1097. doi:10.1001/archpsyc.1996.01830120020005

61. Kumra S, Kranzler H, Gerbino-Rosen G, et al. Clozapine versus “high-dose” olanzapine in refractory early-onset schizophrenia: An open-label extension study. *J Child Adolesc Psychopharmacol*. 2008;18(4):307-316. doi:10.1089/cap.2007.0089

62. Lal S, Thavundayil JX, Nair NPV, et al. Levomepromazine versus chlorpromazine in treatment-resistant schizophrenia: a double-blind randomized trial. *J Psychiatry Neurosci JPN*. 2006;31(4):271-279.

63. Lin CC, Chiu HJ, Chen JY, et al. Switching From Clozapine to Zotepine in Patients With Schizophrenia: A 12-Week Prospective, Randomized, Rater Blind, and Parallel Study. *J Clin Psychopharmacol*. 2013;33(2):211-214. doi:10.1097/JCP.0b013e31828700c7

64. Marjerrison G, Irvine D, Stewart CN, Williams R, Matheu H, Demay M. Withdrawal of long-term phenothiazines from chronically hospitalized psychiatric patients. *Can Psychiatr Assoc J*. 1964;9(4):290-298. doi:10.1177/070674376400900404

65. McGurk SR, Carter C, Goldman R, et al. The effects of clozapine and risperidone on spatial working memory in schizophrenia. *Am J Psychiatry*. 2005;162(5):1013-1016. doi:10.1176/appi.ajp.162.5.1013

66. Schooler NR, Marder SR, Chengappa KNR, et al. Clozapine and risperidone in moderately refractory schizophrenia: a 6-month randomized double-blind comparison. *J Clin Psychiatry*. 2016;77(5):628-634. doi:10.4088/JCP.13m08351

67. McCreadie RG, MacDonald IM. High dosage haloperidol in chronic schizophrenia. *Br J Psychiatry*. 1977;131(3):310-316. doi:10.1192/bjp.131.3.310

68. McEvoy JP, Lieberman JA, Stroup TS, et al. Effectiveness of clozapine versus olanzapine, quetiapine, and risperidone in patients with chronic schizophrenia who did not respond to prior atypical antipsychotic treatment. *Am J Psychiatry*. 2006;163(4):600-610. doi:10.1176/ajp.2006.163.4.600

69. Stroup TS, McEvoy JP, Swartz MS, et al. The National Institute of Mental Health Clinical Antipsychotic Trials of Intervention Effectiveness (CATIE) Project: Schizophrenia Trial Design and Protocol Development. *Schizophr Bull*. 2003;29(1):15-31. doi:10.1093/oxfordjournals.schbul.a006986

70. Nakajima S, Takeuchi H, Fervaha G, et al. Comparative efficacy between clozapine and other atypical antipsychotics on depressive symptoms in patients with schizophrenia: Analysis of the CATIE phase 2E data. *Schizophr Res*. 2015;161(2-3):429-433. doi:10.1016/j.schres.2014.12.024

71. Meltzer HY, Bobo WV, Roy A, et al. A randomized, double-blind comparison of clozapine and high-dose olanzapine in treatment-resistant patients with schizophrenia. *J Clin Psychiatry*. 2008;69(2):274-285. doi:10.4088/jcp.v69n0214

72. Mercer G, Finlayson A, Johnstone EC, Murray C, Owens DG. A study of enhanced management in patients with treatment-resistant schizophrenia. *J Psychopharmacol Oxf Engl*. 1997;11(4):349-356. doi:10.1177/026988119701100411

73. Meyer-Lindenberg A, Gruppe H, Bauer U, Lis S, Krieger S, Gallhofer B. Improvement of cognitive function in schizophrenic patients receiving clozapine or zotepine: results from a double-blind study. *Pharmacopsychiatry*. 1997;30(2):35-42. doi:10.1055/s-2007-979481

74. Moresco RM, Cavallaro R, Messa C, et al. Cerebral D2 and 5-HT2 receptor occupancy in Schizophrenic patients treated with olanzapine or clozapine. *J Psychopharmacol Oxf Engl*. 2004;18(3):355-365. doi:10.1177/026988110401800306

75. Naber D, Riedel M, Klimke A, et al. Randomized double blind comparison of olanzapine vs. clozapine on subjective well-being and clinical outcome in patients with schizophrenia. *Acta Psychiatr Scand*. 2005;111(2):106-115. doi:10.1111/j.1600-0447.2004.00486.x

76. Rosenheck R, Cramer J, Xu W, et al. A comparison of clozapine and haloperidol in hospitalized patients with refractory schizophrenia. Department of Veterans Affairs Cooperative Study Group on Clozapine in Refractory Schizophrenia. *N Engl J Med*. 1997;337(12):809-815. doi:10.1056/NEJM199709183371202

77. Sacchetti E, Galluzzo A, Valsecchi P, Romeo F, Gorini B, Warrington L. Ziprasidone vs clozapine in schizophrenia patients refractory to multiple antipsychotic treatments: The MOZART study. *Schizophr Res*. 2009;113(1):112-121. doi:10.1016/j.schres.2009.05.002

78. Schiele BC, Vestre ND, Stein KE. A comparison of thioridazine, trifluoperazine, chlorpromazine, and placebo: a double-blind controlled study on the treatment of chronic, hospitalized, schizophrenic patients. *J Clin Exp Psychopathol Q Rev Psychiatry Neurol*. 1961;22:151-162.

79. Schlosberg A, Shadmi Weber M. A comparative controlled study of two long-acting phenothiazines: Pipotiazine palmitate and fluphenazine decanoate. *Curr Ther Res*. 1978;23(5):642-654.

80. See RE, Fido AA, Maurice M, Ibrahim MM, Salama GM. Risperidone-induced increase of plasma norepinephrine is not correlated with symptom improvement in chronic schizophrenia. *Biol Psychiatry*. 1999;45(12):1653-1656. doi:10.1016/s0006-3223(98)00199-1

81. Shalev A, Hermesh H, Rothberg J, Munitz H. Poor neuroleptic response in acutely exacerbated schizophrenic patients. *Acta Psychiatr Scand*. 1993;87(2):86-91. doi:10.1111/j.1600-0447.1993.tb03335.x

82. Shaw P, Sporn A, Gogtay N, et al. Childhood-onset schizophrenia: A double-blind, randomized clozapine-olanzapine comparison. *Arch Gen Psychiatry*. 2006;63(7):721-730. doi:10.1001/archpsyc.63.7.721

83. Sirota P, Pannet I, Koren A, Tchernichovsky E. Quetiapine versus olanzapine for the treatment of negative symptoms in patients with schizophrenia. *Hum Psychopharmacol*. 2006;21(4):227-234. doi:10.1002/hup.763

84. Smith RC, Infante M, Singh A, Khandat A. The effects of olanzapine on neurocognitive functioning in medication-refractory schizophrenia. *Int J Neuropsychopharmacol*. 2001;4(3):239-250. doi:10.1017/S146114570100253X

85. Suzuki T, Uchida H, Watanabe K, et al. How effective is it to sequentially switch among Olanzapine, Quetiapine and Risperidone?—A randomized, open-label study of algorithm-based antipsychotic treatment to patients with symptomatic schizophrenia in the real-world clinical setting. *Psychopharmacology (Berl)*. 2007;195(2):285-295. doi:10.1007/s00213-007-0872-2

86. Tollefson GD, Birkett MA, Kiesler GM, Wood AJ, Lilly Resistant Schizophrenia Study Group. Double-blind comparison of olanzapine versus clozapine in schizophrenic patients clinically eligible for treatment with clozapine. *Biol Psychiatry*. 2001;49(1):52-63. doi:10.1016/s0006-3223(00)01026-x

87. Toru M, Shimazono Y, Miyasaka M, Kokubo T, Mori Y, Nasu T. A double-blind comparison of sulpiride with chlorpromazine in chronic schizophrenia. *J Clin Pharmacol New Drugs*. 1972;12(5-6):221-229. doi:10.1002/j.1552-4604.1972.tb00166.x

88. Volavka J, Czobor P, Sheitman B, et al. Clozapine, olanzapine, risperidone, and haloperidol in the treatment of patients with chronic schizophrenia and schizoaffective disorder. *Am J Psychiatry*. 2002;159(2):255-262. doi:10.1176/appi.ajp.159.2.255

89. Wahlbeck K, Cheine M, Tuisku K, Ahokas A, Joffe G, Rimón R. Risperidone versus clozapine in treatment-resistant schizophrenia: a randomized pilot study. *Prog Neuropsychopharmacol Biol Psychiatry*. 2000;24(6):911-922. doi:10.1016/s0278-5846(00)00118-4

90. Wang D, Wei N, Hu F, et al. Paliperidone Extended Release Versus Olanzapine in Treatment-Resistant Schizophrenia: A Randomized, Double-Blind, Multicenter Study. *J Clin Psychopharmacol*. 2022;42(4):383-390. doi:10.1097/JCP.0000000000001573

91. Wirshing DA, Marshall, Jr. BD, Green MF, Mintz J, Marder SR, Wirshing WC. Risperidone in Treatment-Refractory Schizophrenia. *Am J Psychiatry*. 1999;156(9):1374-1379. doi:10.1176/ajp.156.9.1374

92. Afshar H, Roohafza H, Mousavi G, et al. Topiramate add-on treatment in schizophrenia: a randomised, double-blind, placebo-controlled clinical trial. *J Psychopharmacol (Oxf)*. 2009;23(2):157-162. doi:10.1177/0269881108089816

93. Aliyev NA, Aliyev NZ. P03-360 - The Effect of Lamotrigine Augmentation to Haloperidol Deaconate in the Treatment of Resistant Schizophrenia: Randomized, Double-Blind, Placebo-Controlled, Study. *Eur Psychiatry*. 2010;25(S1):25-E966. doi:10.1016/S0924-9338(10)70966-4

94. Anil Yağcioğlu AE, Kivircik Akdede BB, Turgut TI, et al. A double-blind controlled study of adjunctive treatment with risperidone in schizophrenic patients partially responsive to clozapine: efficacy and safety. *J Clin Psychiatry*. 2005;66(1):63-72. doi:10.4088/jcp.v66n0109

95. Assion HJ, Reinbold H, Lemanski S, Basilowski M, Juckel G. Amisulpride Augmentation in Patients with Schizophrenia Partially Responsive or Unresponsive to Clozapine. A Randomized, Double-Blind, Placebo-Controlled Trial. *Pharmacopsychiatry*. 2008;41(1):24-28. doi:10.1055/s-2007-993209

96. Barnes TR, Leeson VC, Paton C, et al. Amisulpride augmentation in clozapine-unresponsive schizophrenia (AMICUS): a double-blind, placebo-controlled, randomised trial of clinical effectiveness and cost-effectiveness. *Health Technol Assess*. 2017;21(49):1-56. doi:10.3310/hta21490

97. Behdani F, Hebrani P, Rezaei Ardani A, Rafee E. Effect of topiramate augmentation in chronic schizophrenia: a placebo-controlled trial. *Arch Iran Med*. 2011;14(4):270-275.

98. Biederman J, Lerner Y, Belmaker RH. Combination of lithium carbonate and haloperidol in schizo-affective disorder: a controlled study. *Arch Gen Psychiatry*. 1979;36(3):327-333. doi:10.1001/archpsyc.1979.01780030093009

99. Boggeto F, Fonzo V, Glusoppe M, Ravizza L. Adjuctive Fluoxetine or Amisulpride Improves Schizophrenic Negative Symptoms. *Eur J Psychiatry*. 1995;9(2):119-127.

100. Borovicka MC, Fuller MA, Konicki PE, White JC, Steele VM, Jaskiw GE. Phenylpropanolamine appears not to promote weight loss in patients with schizophrenia who have gained weight during clozapine treatment. *J Clin Psychiatry*. 2002;63(4):345-348. doi:10.4088/jcp.v63n0412

101. Buchanan RW, Kirkpatrick B, Bryant N, Ball P, Breier A. Fluoxetine augmentation of clozapine treatment in patients with schizophrenia. *Am J Psychiatry*. 1996;153(12):1625-1627. doi:10.1176/ajp.153.12.1625

102. Carpenter WT, Breier A, Buchanan RW, Kirkpatrick B, Shepard P, Weiner E. Mazindol treatment of negative symptoms. *Neuropsychopharmacol Off Publ Am Coll Neuropsychopharmacol*. 2000;23(4):365-374. doi:10.1016/S0893-133X(00)00115-9

103. Chang JS, Ahn YM, Park HJ, et al. Aripiprazole augmentation in clozapine-treated patients with refractory schizophrenia: an 8-week, randomized, double-blind, placebo-controlled trial. *J Clin Psychiatry*. 2008;69(5):720-731. doi:10.4088/jcp.v69n0505

104. Chen CH, Huang MC, Kao CF, et al. Effects of adjunctive metformin on metabolic traits in nondiabetic clozapine-treated patients with schizophrenia and the effect of metformin discontinuation on body weight: A 24-week, randomized, double-blind, placebo-controlled study. *J Clin Psychiatry*. 2013;74(05):e424-e430. doi:10.4088/JCP.12m08186

105. Cipriani A, Accordini S, Nosè M, et al. Aripiprazole Versus Haloperidol in Combination With Clozapine for Treatment-Resistant Schizophrenia: A 12-Month, Randomized, Naturalistic Trial. *J Clin Psychopharmacol*. 2013;33(4):533-537. doi:10.1097/JCP.0b013e318296884f

106. Barbui C, Accordini S, Nosè M, et al. Aripiprazole Versus Haloperidol in Combination With Clozapine for Treatment-Resistant Schizophrenia in Routine Clinical Care: A Randomized, Controlled Trial. *J Clin Psychopharmacol*. 2011;31(3):266-273. doi:10.1097/JCP.0b013e318219cba3

107. de Lucena D, Fernandes BS, Berk M, et al. Improvement of negative and positive symptoms in treatment-refractory schizophrenia: a double-blind, randomized, placebo-controlled trial with memantine as add-on therapy to clozapine. *J Clin Psychiatry*. 2009;70(10):1416-1423. doi:10.4088/JCP.08m04935gry

108. Doruk A, Uzun O, Ozşahin A. A placebo-controlled study of extract of ginkgo biloba added to clozapine in patients with treatment-resistant schizophrenia. *Int Clin Psychopharmacol*. 2008;23(4):223-227. doi:10.1097/YIC.0b013e3282fcff2f

109. Evins AE, Fitzgerald SM, Wine L, Rosselli R, Goff DC. Placebo-controlled trial of glycine added to clozapine in schizophrenia. *Am J Psychiatry*. 2000;157(5):826-828. doi:10.1176/appi.ajp.157.5.826

110. Fan X, Borba CPC, Copeland P, et al. Metabolic effects of adjunctive aripiprazole in clozapine-treated patients with schizophrenia. *Acta Psychiatr Scand*. 2013;127(3):217-226. doi:10.1111/acps.12009

111. Fleischhacker WW, Heikkinen ME, Olié JP, et al. Effects of adjunctive treatment with aripiprazole on body weight and clinical efficacy in schizophrenia patients treated with clozapine: a randomized, double-blind, placebo-controlled trial. *Int J Neuropsychopharmacol*. 2010;13(08):1115-1125. doi:10.1017/S1461145710000490

112. Freudenreich O, Henderson DC, Walsh JP, Culhane MA, Goff DC. Risperidone augmentation for schizophrenia partially responsive to clozapine: A double-blind, placebo-controlled trial. *Schizophr Res*. 2007;92(1-3):90-94. doi:10.1016/j.schres.2006.12.030

113. Freudenreich O, Henderson DC, Macklin EA, et al. Modafinil for Clozapine-Treated Schizophrenia Patients: A Double-Blind, Placebo-Controlled Pilot Trial. *J Clin Psychiatry*. 2009;70(12):1674-1680. doi:10.4088/JCP.08m04683

114. Friedman JI, Lindenmayer JP, Alcantara F, et al. Pimozide augmentation of clozapine inpatients with schizophrenia and schizoaffective disorder unresponsive to clozapine monotherapy. *Neuropsychopharmacol Off Publ Am Coll Neuropsychopharmacol*. 2011;36(6):1289-1295. doi:10.1038/npp.2011.14

115. Genç Y, Taner E, Candansayar S. Comparison of clozapine-amisulpride and clozapine-quetiapine combinations for patients with schizophrenia who are partially responsive to clozapine: a single-blind randomized study. *Adv Ther*. 2007;24(1):1-13. doi:10.1007/BF02849987

116. Goff DC, Henderson DC, Evins AE, Amico E. A placebo-controlled crossover trial of D-cycloserine added to clozapine in patients with schizophrenia. *Biol Psychiatry*. 1999;45(4):512-514. doi:10.1016/s0006-3223(98)00367-9

117. Gunduz-Bruce H, Oliver S, Gueorguieva R, et al. Efficacy of pimozide augmentation for clozapine partial responders with schizophrenia. *Schizophr Res*. 2013;143(2-3):344-347. doi:10.1016/j.schres.2012.11.008

118. Hatta K, Otachi T, Sudo Y, et al. A comparison between augmentation with olanzapine and increased risperidone dose in acute schizophrenia patients showing early non-response to risperidone. *Psychiatry Res*. 2012;198(2):194-201. doi:10.1016/j.psychres.2012.01.006

119. Hatta K, Otachi T, Fujita K, et al. Antipsychotic switching versus augmentation among early non-responders to risperidone or olanzapine in acute-phase schizophrenia. *Schizophr Res*. 2014;158(1-3):213-222. doi:10.1016/j.schres.2014.07.015

120. Honer WG, Thornton AE, Chen EYH, et al. Clozapine alone versus clozapine and risperidone with refractory schizophrenia. *N Engl J Med*. 2006;354(5):472-482. doi:10.1056/NEJMoa053222

121. Hosseini SMR, Farokhnia M, Rezaei F, et al. Intranasal desmopressin as an adjunct to risperidone for negative symptoms of schizophrenia: A randomized, double-blind, placebo-controlled, clinical trial. *Eur Neuropsychopharmacol*. 2014;24(6):846-855. doi:10.1016/j.euroneuro.2014.02.001

122. Ibrahim I, Tobar S, Fathi W, et al. Randomized controlled trial of adjunctive Valproate for cognitive remediation in early course schizophrenia. *J Psychiatr Res*. 2019;118:66-72. doi:10.1016/j.jpsychires.2019.08.011

123. Josiassen RC, Joseph A, Kohegyi E, et al. Clozapine augmented with risperidone in the treatment of schizophrenia: a randomized, double-blind, placebo-controlled trial. *Am J Psychiatry*. 2005;162(1):130-136. doi:10.1176/appi.ajp.162.1.130

124. Kelly DL, Sullivan KM, McEvoy JP, et al. Adjunctive Minocycline in Clozapine-Treated Schizophrenia Patients With Persistent Symptoms. *J Clin Psychopharmacol*. 2015;35(4):374-381. doi:10.1097/JCP.0000000000000345

125. Kotler M, Strous RD, Reznik I, Shwartz S, Weizman A, Spivak B. Sulpiride augmentation of olanzapine in the management of treatment-resistant chronic schizophrenia: evidence for improvement of mood symptomatology. *Int Clin Psychopharmacol*. 2004;19(1):23-26. doi:10.1097/00004850-200401000-00004

126. Krivoy A, Onn R, Vilner Y, et al. Vitamin D Supplementation in Chronic Schizophrenia Patients Treated with Clozapine: A Randomized, Double-Blind, Placebo-controlled Clinical Trial. *EBioMedicine*. 2017;26:138-145. doi:10.1016/j.ebiom.2017.11.027

127. Lane HY, Huang CL, Wu PL, et al. Glycine Transporter I Inhibitor, N-methylglycine (Sarcosine), Added to Clozapine for the Treatment of Schizophrenia. *Biol Psychiatry*. 2006;60(6):645-649. doi:10.1016/j.biopsych.2006.04.005

128. Lang X, Zang X, Yu F, Xiu M. Effects of low-dose combined olanzapine and sertraline on negative and depressive symptoms in treatment-resistant outpatients with acute exacerbated schizophrenia. *Front Pharmacol*. 2023;14:1166507. doi:10.3389/fphar.2023.1166507

129. Cho SJ, Yook K, Kim B, et al. Mirtazapine augmentation enhances cognitive and reduces negative symptoms in schizophrenia patients treated with risperidone: A randomized

controlled trial. *Prog Neuropsychopharmacol Biol Psychiatry*. 2011;35(1):208-211. doi:10.1016/j.pnpbp.2010.11.006

130. Lin CH, Lin CH, Chang YC, et al. Sodium Benzoate, a D-Amino Acid Oxidase Inhibitor, Added to Clozapine for the Treatment of Schizophrenia: A Randomized, Double-Blind, Placebo-Controlled Trial. *Biol Psychiatry*. 2018;84(6):422-432. doi:10.1016/j.biopsych.2017.12.006

131. Lu ML, Lane HY, Lin SK, Chen KP, Chang WH. Adjunctive fluvoxamine inhibits clozapine-related weight gain and metabolic disturbances. *J Clin Psychiatry*. 2004;65(6):766-771. doi:10.4088/jcp.v65n0607

132. Lu ML, Chen TT, Kuo PH, Hsu CC, Chen CH. Effects of adjunctive fluvoxamine on metabolic parameters and psychopathology in clozapine-treated patients with schizophrenia: A 12-week, randomized, double-blind, placebo-controlled study. *Schizophr Res*. 2018;193:126-133. doi:10.1016/j.schres.2017.06.030

133. Mayabhate M, Badar V, Waradkar P, Somani A. Cognitive and psychomotor effects of adjunctive aripiprazole or paliperidone in patients of schizophrenia receiving olanzapine: a double blind placebo controlled clinical study. *Int J Basic Clin Pharmacol*. 2014;3(1):130. doi:10.5455/2319-2003.ijbcp20140216

134. Mico' U, Bruno A, Pandolfo G, et al. Duloxetine as adjunctive treatment to clozapine in patients with schizophrenia: a randomized, placebo-controlled trial. *Int Clin Psychopharmacol*. 2011;26(6):303-310. doi:10.1097/YIC.0b013e32834bbc0d

135. Moazen-Zadeh E, Bayanati S, Ziafat K, Rezaei F, Mesgarpour B, Akhondzadeh S. Vortioxetine as adjunctive therapy to risperidone for treatment of patients with chronic schizophrenia: A randomised, double-blind, placebo-controlled clinical trial. *J Psychopharmacol Oxf Engl*. 2020;34(5):506-513. doi:10.1177/0269881120909416

136. Modabbernia A, Rezaei F, Salehi B, et al. Intranasal oxytocin as an adjunct to risperidone in patients with schizophrenia : an 8-week, randomized, double-blind, placebo-controlled study. *CNS Drugs*. 2013;27(1):57-65. doi:10.1007/s40263-012-0022-1

137. Mossaheb N, Sacher J, Wiesegger G, et al. P.3.c.037 Haloperidol in combination with clozapine in treatment-refractory patients with schizophrenia. *Eur Neuropsychopharmacol*. 2006;16(4):S416. doi:10.1016/S0924-977X(06)70524-7

138. Muscatello M, Bruno A, Pandolfo G, et al. Topiramate augmentation of clozapine in schizophrenia: a double-blind, placebo-controlled study. *J Psychopharmacol (Oxf)*. 2010;25(5):667-674. doi:10.1177/0269881110372548

139. Muscatello MRA, Bruno A, Pandolfo G, et al. Effect of aripiprazole augmentation of clozapine in schizophrenia: A double-blind, placebo-controlled study. *Schizophr Res*. 2011;127(1-3):93-99. doi:10.1016/j.schres.2010.12.011

140. Muscatello MRA, Pandolfo G, Micò U, et al. Augmentation of Clozapine With Ziprasidone in Refractory Schizophrenia: A Double-Blind, Placebo-Controlled Study. *J Clin Psychopharmacol*. 2014;34(1):129-133. doi:10.1097/JCP.0000000000000042

141. Neill E, Rossell SL, Yolland C, et al. N-Acetylcysteine (NAC) in Schizophrenia Resistant to Clozapine: A Double-Blind, Randomized, Placebo-Controlled Trial Targeting Negative Symptoms. *Schizophr Bull*. 2022;48(6):1263-1272. doi:10.1093/schbul/sbac065

142. Nielsen J, Emborg C, Gydesen S, et al. Augmenting Clozapine With Sertindole: A Double-Blind, Randomized, Placebo-Controlled Study. *J Clin Psychopharmacol*. 2012;32(2):173-178. doi:10.1097/JCP.0b013e318248dfb8

143. Potkin SG, Jin Y, Bunney BG, Costa J, Gulasekaram B. Effect of clozapine and adjunctive high-dose glycine in treatment-resistant schizophrenia. *Am J Psychiatry*. 1999;156(1):145-147. doi:10.1176/ajp.156.1.145

144. Repo-Tiihonen E, Hallikainen T, Kivistö P, Tiihonen J. Antipsychotic polypharmacy in clozapine resistant schizophrenia: a randomized controlled trial of tapering antipsychotic co-treatment. *Ment Illn*. 2012;4(1):1-4. doi:10.4081/mi.2012.e1

145. Samadi R, Akhoundpour Manteghi A, Baghban Haghighi M, Azhari A, Assari S. Low Dose Celecoxib Combined with Clozapine for Treatment of Schizophrenia: A Double Blind Randomized Clinical Trial. *Int J Travel Med Glob Health*. 2015;3(1):11-17. doi:10.20286/ijtmgh-030111

146. Samadi R, Soluti S, Daneshmand R, Assari S, Manteghi AA. Efficacy of Risperidone Augmentation with Ondansetron in the Treatment of Negative and Depressive Symptoms in Schizophrenia: A Randomized Clinical Trial. *Iran J Med Sci*. 2017;42(1):14-23.

147. Samaei A, Moradi K, Bagheri S, et al. Resveratrol Adjunct Therapy for Negative Symptoms in Patients With Stable Schizophrenia: A Double-Blind, Randomized Placebo-Controlled Trial. *Int J Neuropsychopharmacol*. 2020;23(12):775-782. doi:10.1093/ijnp/pyaa006

148. Salehi A, Namaei P, TaghaviZanjani F, et al. Adjuvant palmitoylethanolamide therapy with risperidone improves negative symptoms in patients with schizophrenia: A randomized, double-blinded, placebo-controlled trial. *Psychiatry Res*. 2022;316:114737. doi:10.1016/j.psychres.2022.114737

149. Schulz SC, Thompson PA, Jacobs M, et al. Lithium augmentation fails to reduce symptoms in poorly responsive schizophrenic outpatients. *J Clin Psychiatry*. 1999;60(6):366-372.

150. Shamabadi A, Rafiei-Tabatabaei ES, Kazemzadeh K, et al. Pentoxifylline adjunct to risperidone for negative symptoms of stable schizophrenia: a randomized, double-blind, placebo-controlled trial. *Int J Neuropsychopharmacol*. 2024;28(1):pyae051. doi:10.1093/ijnp/pyae051

151. Shi H, Xu J, Lang X, Wu HE, Xiu MH, Zhang XY. Comparison of Efficacy and Safety Between Low-Dose Ziprasidone in Combination With Sertraline and Ziprasidone Monotherapy for Treatment-Resistant Patients With Acute Exacerbation Schizophrenia: A Randomized Controlled Trial. *Front Pharmacol*. 2022;13:863588. doi:10.3389/fphar.2022.863588

152. Shiloh R, Zemishlany Z, Aizenberg D, et al. Sulpiride augmentation in people with schizophrenia partially responsive to clozapine. A double-blind, placebo-controlled study. *Br J Psychiatry J Ment Sci*. 1997;171:569-573. doi:10.1192/bjp.171.6.569

153. Shoja-Shafti S. Augmentation of aripiprazole by flupenthixol decanoate in poorly responsive schizophrenia: a randomized clinical study. *Psychiatry Clin Psychopharmacol*. 2017;27:235-242.

154. Siris SG, Bermanzohn PC, Gonzalez A, Mason SE, White CV, Shuwall MA. The use of antidepressants for negative symptoms in a subset of schizophrenic patients. *Psychopharmacol Bull*. 1991;27(3):331-335.

155. Siskind D, Russell AW, Suetani S, et al. CoMET: a randomised controlled trial of co-commencement of metformin versus placebo as an adjunctive treatment to attenuate weight gain in patients with schizophrenia newly commenced on clozapine. *Ther Adv Psychopharmacol*. 2021;11:20451253211045248. doi:10.1177/20451253211045248

156. Stryjer R, Strous R, Bar F, et al. Donepezil augmentation of clozapine monotherapy in schizophrenia patients: a double blind cross-over study. *Hum Psychopharmacol*. 2004;19(5):343-346. doi:10.1002/hup.595

157. Sulejmanpasic G, Bise S. Clozapine augmented with amisulpride in treatment-resistant schizophrenia. *Eur Neuropsychopharmacol*. 2019;29(3):S121-S122. doi:10.1016/j.euroneuro.2018.11.229

158. Talbot DR. Are tranquilizer combinations more effective than a single tranquilizer? *Am J Psychiatry*. 1964;121(6):597-600. doi:10.1176/ajp.121.6.597

159. Tiihonen J, Hallikainen T, Ryynänen OP, et al. Lamotrigine in treatment-resistant schizophrenia: a randomized placebo-controlled crossover trial. *Biol Psychiatry*. 2003;54(11):1241-1248. doi:10.1016/s0006-3223(03)00524-9

160. Tsai GE, Yang P, Chung LC, Tsai IC, Tsai CW, Coyle JT. D-serine added to clozapine for the treatment of schizophrenia. *Am J Psychiatry*. 1999;156(11):1822-1825. doi:10.1176/ajp.156.11.1822

161. Vayısoğlu S, Anıl Yağcıoğlu AE, Yağcıoğlu S, et al. Lamotrigine augmentation in patients with schizophrenia who show partial response to clozapine treatment. *Schizophr Res*. 2013;143(1):207-214. doi:10.1016/j.schres.2012.11.006

162. Veerman SRT, Schulte PFJ, Smith JD, de Haan L. Memantine augmentation in clozapine-refractory schizophrenia: a randomized, double-blind, placebo-controlled crossover study. *Psychol Med*. 2016;46(9):1909-1921. doi:10.1017/S0033291716000398

163. Weiner E, Conley RR, Ball MP, et al. Adjunctive risperidone for partially responsive people with schizophrenia treated with clozapine. *Neuropsychopharmacol Off Publ Am Coll Neuropsychopharmacol*. 2010;35(11):2274-2283. doi:10.1038/npp.2010.101

164. Wilson WH. Addition of lithium to haloperidol in non-affective, antipsychotic non-responsive schizophrenia: a double blind, placebo controlled, parallel design clinical trial. *Psychopharmacology (Berl)*. 1993;111(3):359-366. doi:10.1007/BF02244953

165. Woo YS, Park SY, Yoon BH, Choi WS, Wang SM, Bahk WM. Amisulpride Augmentation in Schizophrenia Patients with Poor Response to Olanzapine: A 4-week, Randomized, Rater-Blind, Controlled, Pilot Study. *Clin Psychopharmacol Neurosci Off Sci J Korean Coll Neuropsychopharmacol*. 2022;20(3):567-572. doi:10.9758/cpn.2022.20.3.567

166. Xiao S, Xue H, Li G, et al. Therapeutic effects of cerebrolysin added to risperidone in patients with schizophrenia dominated by negative symptoms. *Aust N Z J Psychiatry*. 2012;46(2):153-160. doi:10.1177/0004867411433213

167. Zhang XY, Zhou DF, Su JM, Zhang PY. The effect of extract of ginkgo biloba added to haloperidol on superoxide dismutase in inpatients with chronic schizophrenia. *J Clin Psychopharmacol*. 2001;21(1):85-88. doi:10.1097/00004714-200102000-00015

168. Zhang XY, Zhou DF, Zhang PY, Wu GY, Su JM, Cao LY. A double-blind, placebo-controlled trial of extract of Ginkgo biloba added to haloperidol in treatment-resistant patients with schizophrenia. *J Clin Psychiatry*. 2001;62(11):878-883. doi:10.4088/jcp.v62n1107

169. Zhang ZJ, Kang WH, Li Q, Wang XY, Yao SM, Ma AQ. Beneficial effects of ondansetron as an adjunct to haloperidol for chronic, treatment-resistant schizophrenia: a double-blind, randomized, placebo-controlled study. *Schizophr Res*. 2006;88(1-3):102-110. doi:10.1016/j.schres.2006.07.010

170. Zhou D, Zhang X, Su J, et al. The effects of classic antipsychotic haloperidol plus the extract of ginkgo biloba on superoxide dismutase in patients with chronic refractory schizophrenia. *Chin Med J (Engl)*. 1999;112(12):1093-1096.

171. Zhu MH, Liu ZJ, Hu QY, et al. Amisulpride augmentation therapy improves cognitive performance and psychopathology in clozapine-resistant treatment-refractory schizophrenia: a 12-week randomized, double-blind, placebo-controlled trial. *Mil Med Res*. 2022;9(1):59. doi:10.1186/s40779-022-00420-0

172. Zink M, Kuwilsky A, Krumm B, Dressing H. Efficacy and tolerability of ziprasidone versus risperidone as augmentation in patients partially responsive to clozapine: a randomised controlled clinical trial. *J Psychopharmacol Oxf Engl*. 2009;23(3):305-314. doi:10.1177/0269881108089593

173. Zoccali R, Muscatello MR, Bruno A, et al. The effect of lamotrigine augmentation of clozapine in a sample of treatment-resistant schizophrenic patients: a double-blind, placebo-controlled study. *Schizophr Res*. 2007;93(1-3):109-116. doi:10.1016/j.schres.2007.02.009

174. Evrenoglou T, White IR, Afach S, Mavridis D, Chaimani A. Network meta-analysis of rare events using penalized likelihood regression. *Stat Med*. 2022;41(26):5203-5219. doi:10.1002/sim.9562

175. Efthimiou O, Rücker G, Schwarzer G, Higgins JPT, Egger M, Salanti G. Network meta-analysis of rare events using the Mantel-Haenszel method. *Stat Med*. 2019;38(16):2992-3012. doi:10.1002/sim.8158

176. Rücker G, Schwarzer G. Ranking treatments in frequentist network meta-analysis works without resampling methods. *BMC Med Res Methodol*. 2015;15:58. doi:10.1186/s12874-015-0060-8

177. Dias S, Welton NJ, Caldwell DM, Ades AE. Checking consistency in mixed treatment comparison meta-analysis. *Stat Med*. 2010;29(7-8):932-944. doi:10.1002/sim.3767

178. Higgins JPT, Jackson D, Barrett JK, Lu G, Ades AE, White IR. Consistency and inconsistency in network meta-analysis: concepts and models for multi-arm studies. *Res Synth Methods*. 2012;3(2):98-110. doi:10.1002/jrsm.1044

179. Krahn U, Binder H, König J. A graphical tool for locating inconsistency in network meta-analyses. *BMC Med Res Methodol*. 2013;13(1):35. doi:10.1186/1471-2288-13-35

180. R Core Team. _R: A Language and Environment for Statistical Computing_. Published online 2024. <https://www.R-project.org/>

181. Balduzzi S, Rücker G, Nikolakopoulou A, et al. **netmeta** : An *R* Package for Network Meta-Analysis Using Frequentist Methods. *J Stat Softw*. 2023;106(2):1-40. doi:10.18637/jss.v106.i02

182. Phillippo DM. multinma: Bayesian Network Meta-Analysis of Individual and Aggregate Data. Published online 2024. doi:10.5281/ZENODO.3904454

183. Nikolakopoulou A, Higgins JPT, Papakonstantinou T, et al. CINeMA: An approach for assessing confidence in the results of a network meta-analysis. *PLOS Med*. 2020;17(4):e1003082. doi:10.1371/journal.pmed.1003082

184. Shen X, Xia J, Adams CE. Flupenthixol versus placebo for schizophrenia. Cochrane Schizophrenia Group, ed. *Cochrane Database Syst Rev*. 2012;2012(11). doi:10.1002/14651858.CD009777.pub2

185. Rhodes KM, Turner RM, Higgins JPT. Predictive distributions were developed for the extent of heterogeneity in meta-analyses of continuous outcome data. *J Clin Epidemiol*. 2015;68(1):52-60. doi:10.1016/j.jclinepi.2014.08.012

186. Dickersin K, Berlin JA. Meta-analysis: State-of-the-Science. *Epidemiol Rev*. 1992;14(1):154-176. doi:10.1093/oxfordjournals.epirev.a036084

187. Higgins JPT, Thompson SG, Deeks JJ, Altman DG. Measuring inconsistency in meta-analyses. *BMJ*. 2003;327(7414):557-560. doi:10.1136/bmj.327.7414.557

188. Furukawa TA, Salanti G, Atkinson LZ, et al. Comparative efficacy and acceptability of first-generation and second-generation antidepressants in the acute treatment of major depression: protocol for a network meta-analysis. *BMJ Open*. 2016;6(7):e010919. doi:10.1136/bmjopen-2015-010919

189. Chiocchia V, Nikolakopoulou A, Higgins JPT, et al. ROB-MEN: a tool to assess risk of bias due to missing evidence in network meta-analysis. *BMC Med*. 2021;19(1):304. doi:10.1186/s12916-021-02166-3

190. Borenstein M. Avoiding common mistakes in meta-analysis: Understanding the distinct roles of *Q* , *I* -squared, tau-squared, and the prediction interval in reporting heterogeneity. *Res Synth Methods*. 2024;15(2):354-368. doi:10.1002/jrsm.1678

191. Puhan MA, Schunemann HJ, Murad MH, et al. A GRADE Working Group approach for rating the quality of treatment effect estimates from network meta-analysis. *BMJ*. 2014;349(sep24 5):g5630-g5630. doi:10.1136/bmj.g5630

192. Schneider-Thoma J, Chalkou K, Dörries C, et al. Comparative efficacy and tolerability of 32 oral and long-acting injectable antipsychotics for the maintenance treatment of adults with schizophrenia: a systematic review and network meta-analysis. *The Lancet*. 2022;399(10327):824-836. doi:10.1016/S0140-6736(21)01997-8
